# Meta-Analysis of Genome-Wide Association Studies for Abdominal Aortic Aneurysm Identifies Four New Disease-Specific Risk Loci

**DOI:** 10.1161/CIRCRESAHA.116.308765

**Published:** 2017-01-19

**Authors:** Gregory T. Jones, Gerard Tromp, Helena Kuivaniemi, Solveig Gretarsdottir, Annette F. Baas, Betti Giusti, Ewa Strauss, Femke N.G. van‘t Hof, Thomas R. Webb, Robert Erdman, Marylyn D. Ritchie, James R. Elmore, Anurag Verma, Sarah Pendergrass, Iftikhar J. Kullo, Zi Ye, Peggy L. Peissig, Omri Gottesman, Shefali S. Verma, Jennifer Malinowski, Laura J. Rasmussen-Torvik, Kenneth M. Borthwick, Diane T. Smelser, David R. Crosslin, Mariza de Andrade, Evan J. Ryer, Catherine A. McCarty, Erwin P. Böttinger, Jennifer A. Pacheco, Dana C. Crawford, David S. Carrell, Glenn S. Gerhard, David P. Franklin, David J. Carey, Victoria L. Phillips, Michael J.A. Williams, Wenhua Wei, Ross Blair, Andrew A. Hill, Thodor M. Vasudevan, David R. Lewis, Ian A. Thomson, Jo Krysa, Geraldine B. Hill, Justin Roake, Tony R. Merriman, Grzegorz Oszkinis, Silvia Galora, Claudia Saracini, Rosanna Abbate, Raffaele Pulli, Carlo Pratesi, Athanasios Saratzis, Ana R. Verissimo, Suzannah Bumpstead, Stephen A. Badger, Rachel E. Clough, Gillian Cockerill, Hany Hafez, D. Julian A. Scott, T. Simon Futers, Simon P.R. Romaine, Katherine Bridge, Kathryn J. Griffin, Marc A. Bailey, Alberto Smith, Matthew M. Thompson, Frank M. van Bockxmeer, Stefan E. Matthiasson, Gudmar Thorleifsson, Unnur Thorsteinsdottir, Jan D. Blankensteijn, Joep A.W. Teijink, Cisca Wijmenga, Jacqueline de Graaf, Lambertus A. Kiemeney, Jes S. Lindholt, Anne Hughes, Declan T. Bradley, Kathleen Stirrups, Jonathan Golledge, Paul E. Norman, Janet T. Powell, Steve E. Humphries, Stephen E. Hamby, Alison H. Goodall, Christopher P. Nelson, Natzi Sakalihasan, Audrey Courtois, Robert E. Ferrell, Per Eriksson, Lasse Folkersen, Anders Franco-Cereceda, John D. Eicher, Andrew D. Johnson, Christer Betsholtz, Arno Ruusalepp, Oscar Franzén, Eric E. Schadt, Johan L.M. Björkegren, Leonard Lipovich, Anne M. Drolet, Eric L. Verhoeven, Clark J. Zeebregts, Robert H. Geelkerken, Marc R. van Sambeek, Steven M. van Sterkenburg, Jean-Paul de Vries, Kari Stefansson, John R. Thompson, Paul I.W. de Bakker, Panos Deloukas, Robert D. Sayers, Seamus C. Harrison, Andre M. van Rij, Nilesh J. Samani, Matthew J. Bown, The Cardiogenics Consortium, The International Consortium for Blood Pressure

**Affiliations:** For the author affiliations, please see the Appendix.

**Keywords:** aortic aneurysm, abdominal, computational biology, genetics, genome-wide association study, matrix metalloproteinases, meta-analysis

## Abstract

**Rationale:**

Abdominal aortic aneurysm (AAA) is a complex disease with both genetic and environmental risk factors. Together, 6 previously identified risk loci only explain a small proportion of the heritability of AAA.

**Objective:**

To identify additional AAA risk loci using data from all available genome-wide association studies.

**Methods and Results:**

Through a meta-analysis of 6 genome-wide association study data sets and a validation study totaling 10 204 cases and 107 766 controls, we identified 4 new AAA risk loci: 1q32.3 (*SMYD2*), 13q12.11 (*LINC00540*), 20q13.12 (near *PCIF1*/*MMP9*/*ZNF335*), and 21q22.2 (*ERG*). In various database searches, we observed no new associations between the lead AAA single nucleotide polymorphisms and coronary artery disease, blood pressure, lipids, or diabetes mellitus. Network analyses identified *ERG*, *IL6R*, and *LDLR* as modifiers of *MMP9*, with a direct interaction between *ERG* and *MMP9*.

**Conclusions:**

The 4 new risk loci for AAA seem to be specific for AAA compared with other cardiovascular diseases and related traits suggesting that traditional cardiovascular risk factor management may only have limited value in preventing the progression of aneurysmal disease.

## Abstract 2

Supplemental Digital Content is available in the text.

Abdominal aortic aneurysms (AAAs; MIM100070) are a significant cause of mortality and morbidity in the Western world. Although much less common than ischemic heart disease or stroke, AAA is responsible for ≈11 000 deaths/y in the United States, with no clinical treatment other than expensive, high-risk surgery.^1^ The US Preventative Services taskforce recommends AAA screening by ultrasound for all men aged 65 to 75 years who have ever smoked.^2^ The UK NHS AAA Screening Program screens all men at the age of 65 years irrespective of smoking history yielding a prevalence of AAA (>29 mm) of 1.2%.^3^

**Editorial, see p 259**

AAA is an enigmatic complex disease. Although sharing risk factors for, and often coexisting with atherosclerosis, AAA can be considered to be a distinct entity from atherosclerosis. Smoking, a positive family history of AAA, and male sex have been consistently identified as the strongest risk factors for AAA. There is uncertainty over the influence of other traditional cardiovascular risk markers such as hypertension and hyperlipidemia. Furthermore, diabetes mellitus has been found to be negatively associated with AAA and is strongly protective against disease progression (AAA growth).^1^

Heritability of AAA is >0.7,^4^ and individuals with a first-degree relative with AAA have a 2-fold higher risk of developing an AAA.^5^ Genome-wide association studies (GWAS) have identified 3 AAA risk loci on chromosomes 9 (*DAB2IP*^6^ [DAB2 interacting protein]), 12 (*LRP1*^7^ [low-density lipoprotein receptor related protein 1]), and 19 (*LDLR*^8^ [low-density lipoprotein receptor]). Further AAA risk loci on chromosomes 1 (*SORT1*^9^ [sortilin 1] and *IL6R*^10^ [interleukin 6 receptor]) and 9 (*CDKN2BAS1/ANRIL*^11^ [also known as CDKN2B-AS1, CDKN2B antisense RNA 1]) were identified by candidate gene/locus approaches. Together, these explain only a small proportion of the heritability of AAA.

Overall, the high heritability estimates for AAA and the small number of loci identified suggest that there are further risk loci yet to be found. In the current study, we performed a meta-analysis of 6 available GWAS data sets for AAA on 4972 cases and 99 858 controls and confirmed the findings within validation data sets of 5232 cases and 7908 controls. This resulted in identification of 4 novel validated loci for AAA. We followed up positive results with extensive bioinformatics analyses and used data available from various databases to elucidate the potential biological significance of our findings to the pathobiology of AAA.

## Methods

Detailed Methods are available in the Online Data Supplement.

### Expanded Aneurysm Consortium

All known studies with AAA genome-wide genotyping (Online Methods; Online Table I) were invited to join the International Aneurysm Consortium. Additional samples (Online Methods; Online Table II) were used for the validation study. All AAA cases had an infrarenal aortic diameter of >30 mm. AAAs secondary to connective tissue diseases were excluded. The use of the samples in each study cohort was approved by local Ethics Committees or Institutional Review Boards.

### Meta-Analysis

The discovery phase of the meta-GWAS was conducted using the METAL (a tool for meta-analysis of genome-wide association scans) software package^12^ on the 6 cohorts detailed in Online Table I, comprising 4972 AAA cases and 99 858 controls. An effective sample number (N_eff_) weighted analysis^12^ was conducted because of case/control asymmetry within some of the contributing cohorts. Quality control included assessments for population stratification in each data set and adjustment was performed if necessary. The analysis of each contributing GWAS had been performed independently, and there was therefore no uniform analysis plan across all data sets. The individual GWAS data sets from Iceland and the Netherlands were adjusted for genomic inflation before inclusion in the meta-analysis. The overall meta-analysis was then adjusted for genomic inflation (λ; Online Table I; Online Figure I). An initial (λ-adjusted) discovery threshold of *P*<5×10^−^^6^ was used to identify single nucleotide polymorphisms (SNPs) for subsequent validation genotyping. SNPs with high heterogeneity (*P*_het_ <0.005 or *I*^2^>70%) were not taken forward for validation.

The lead SNPs [or their proxies in high linkage disequilibrium], identified in the discovery analyses, were then genotyped in a further 8 independent cohorts with 5,232 cases and 7,908 controls (Online Table II). Allele association analysis of each individual validation study cohort was carried out using the SHEsis (software platform for analyses of linkage disequilibrium, haplotype construction, and genetic association at polymorphism loci) web-based software package.^13^ A combined (discovery-validation) fixed effect meta-analysis was performed using a Maentel–Haenzel method with the genome-wide *P*-value significance threshold being set at 5×10^−^^8^. Random-effects (Han-Eskin method^14^) meta-analysis was also performed to determine whether any results were sensitive to between-study heterogeneity.

### SNP Lookup in GWAS for Other Traits Associated With AAA

GWAS data sets for other traits were searched for associations with the AAA-associated SNPs to determine whether the associations were unique to AAA or related to generalized cardiovascular disease. Results were obtained from meta-analyses of multiple primary GWAS data sets for each trait. Summary data for each AAA associated SNP (*P* value and effect size) were extracted. *P* values <5×10^−^^8^ were considered to be significant. Results were available for type 2 diabetes mellitus^15^ (DIAGRAM [a consortium called DIAbetes Genetics Replication And Meta-analysis] consortium; http://www.diagram-consortium.org/index.html), coronary artery disease (CAD; CARDIoGRAM consortium (a consortium called Coronary ARtery DIsease Genome wide Replication and Meta-analysis)^16^; www.CARDIOGRAMPLUSC4D.ORG), lipids (the Global Lipids Genetics Consortium^17^; http://csg.sph.umich.edu/abecasis/public/lipids2013), and blood pressure (the International Consortium for Blood Pressure^18^; http://www.ncbi.nlm.nih.gov/projects/gap/cgi-bin/study.cgi?study_id=phs000585.v1.p1).

### Search for Other Associated Traits and Diseases Using GWAS Databases

The Phenotype-Genotype Integrator^19^ (http://www.ncbi.nlm.nih.gov/gap/phegeni#GenomeView), the GWAS catalog (http://www.gwascentral.org/index), and the NHLBI GRASP (The Genome-wide Repository of Associations between SNPs and Phenotypes) catalog (GRASP v2.0; http://grasp.nhlbi.nih.gov/Overview.aspx)^20^ were searched for diseases and traits associated with the lead SNPs at the AAA loci.

### Phenome-Wide Association Study Analysis

We performed a phenome-wide association study (PheWAS)^21,22^ exploring associations between the 9 AAA-associated SNPs and an extensive group of diagnoses to identify novel associations and uncover potential pleiotropy. For the PheWAS, we used data from the eMERGE (electronic Medical Records and Genomics) Network^23^ with a total of 27 077 unrelated patients of European ancestry aged >19 years. We divided these samples into 2 data sets by proportional sampling based on eMERGE site, sex, and genotyping platform (13 559 and 13 518 individuals in sets 1 and 2, respectively). We calculated associations between the 9 AAA-associated SNPs and case or control status based on the extensive set of 9th edition of the *International Statistical Classification of Diseases* and Related Health Problems diagnoses (2408 and 2385 in sets 1 and 2, respectively) where for a specific diagnosis, individuals with the diagnosis are considered cases. Associations were adjusted for sex, site, genotyping platform, and the first 3 principal components to account for global ancestry.

### Annotation of AAA Associated SNPs Using the University of California Santa Cruz Genome Browser, Pupasuite, and GWAS3D

Confirmed AAA-associated loci were manually annotated using the University of California Santa Cruz Genome Browser (http://genome.ucsc.edu/cgi-bin/hgGateway) on the hg19 human genome assembly. For the Pupasuite analyses SNPs in linkage disequilibrium (*r*^2^>0.5) and with lead SNPs at the novel AAA risk loci identified were extracted from the 1000 Genomes data and then entered into Pupasuite v3.1.^24^ In addition, all known (novel and previously identified) AAA-associated SNPs were entered into the GWAS3D (bioinformatics tool detecting human regulatory variants by integrative analysis of genome-wide associations, chromosome interactions, and histone modifications)^25^ web-portal (http://jjwanglab.org/gwas3d) to identify functional SNPs.

### Bioinformatic Identification of Candidate AAA Genes and Pathways Using DEPICT (Data-Driven Expression-Prioritized Integration for Complex Traits)

An integrated gene function analysis was performed using the DEPICT tool (version 1.1).^26^ Two separate runs were performed using either all independent SNPs with discovery meta-GWAS *P*<5×10^−^^6^ or just those 9 SNPs that reached *P*<5×10^−^^8^ in the combined analysis. Both nominal *P* values and false discovery rates were calculated.

### Experimental Evidence for Functional Variants at AAA Loci

SNPs at loci confirmed to be associated with AAA were examined for functional effects using multiple methods (Online Methods). (1) To search for evidence of functional effects of SNPs at AAA associated loci 2 expression quantitative trait locus (eQTL) data sets based on publically available data, and a broad range of tissues with relatively large sample sizes were examined. First, index and proxy SNPs were queried in a collected database of published expressed SNP results. The collected expressed SNP results met criteria for statistical thresholds for association with gene transcript levels as described in the original publications. Second, additional eQTL data were integrated from online sources including ScanDB (SNP and CNV Annotation Database), the Broad Institute The Genotype-Tissue Expression browser, and the Pritchard Laboratory (eqtl.uchicago.edu). (2) To search for vascular tissue-specific effects, eQTL data were also obtained from the ASAP (Advanced Study of Aortic Pathology) data set^27^ and RNA-seq (whole-genome RNA-sequence generated by high-throughput methods) data were from the Stockholm-Tartu Atherosclerosis Reverse Network Engineering Task (STARNET) database^28^ (http://www.mountsinai.org/profiles/johan-bjorkegren). (3) Because some genes at AAA loci were associated with monocyte function and AAA is known to be an inflammatory disease,^29^ data from an eQTL analysis of peripheral blood monocytes were obtained from the Cardiogenics Consortium (http://www.cardiogramplusc4d.org/). (4) Finally to search for effects in AAA tissue specifically, mRNA expression profiles of all the GWAS3D predicted distal targets, as well as SNP proximity implicated genes, were examined using a previously published genome-wide expression data set on human aorta (GSE57691),^30^ from which 49 AAA samples were compared with 10 organ donor control aortic samples. Transcription factor (TF) binding data were also obtained from a previous study,^31^ which described chromatin-immunoprecipitation (ChIP)-chip for TFs ELF1, ETS2, RUNX1, and STAT5 using human aortic tissue in AAAs and healthy control aorta.

### Network Analysis

We investigated whether most of the loci could be connected into a single network through intermediate nodes and interactions. A network integrating most of the loci would suggest mechanisms by which the loci could act in concert, whether synergistically or antagonistically, to affect the phenotype. The network(s) would also provide hypotheses for future investigation. Using the genes harboring AAA-associated SNPs as a starting set, we analyzed potential interactions between the proteins and known intermediates (proteins, noncoding RNA, and metabolites) using 2 independent analysis tools, Ingenuity Pathway Analysis (IPA) tool version 9.0 (Qiagen’s Ingenuity Systems, Redwood City, CA; www.ingenuity.com) and Consensus PathDB (http://cpdb.molgen.mpg.de/CPDB).^32,33^ The analyzed gene set had 14 genes because 2 of the 9 AAA loci included clusters of 3 genes and tumor necrosis factor (TNF) was added because of the recent literature demonstrating the strong effect of SMYD2 (SET and MYND domain containing 2 [SET domain-containing proteins, such as catalyze lysine methylation]) on interleukin-6 (IL6) and TNF production^34,35^ (see Online Table XIV for SNP annotations and Online Methods).

## Results

### Meta-Analysis of 6 GWAS Data sets for AAA Followed by a Validation Study Reveals 4 New AAA Susceptibility Loci

**Figure 1.**
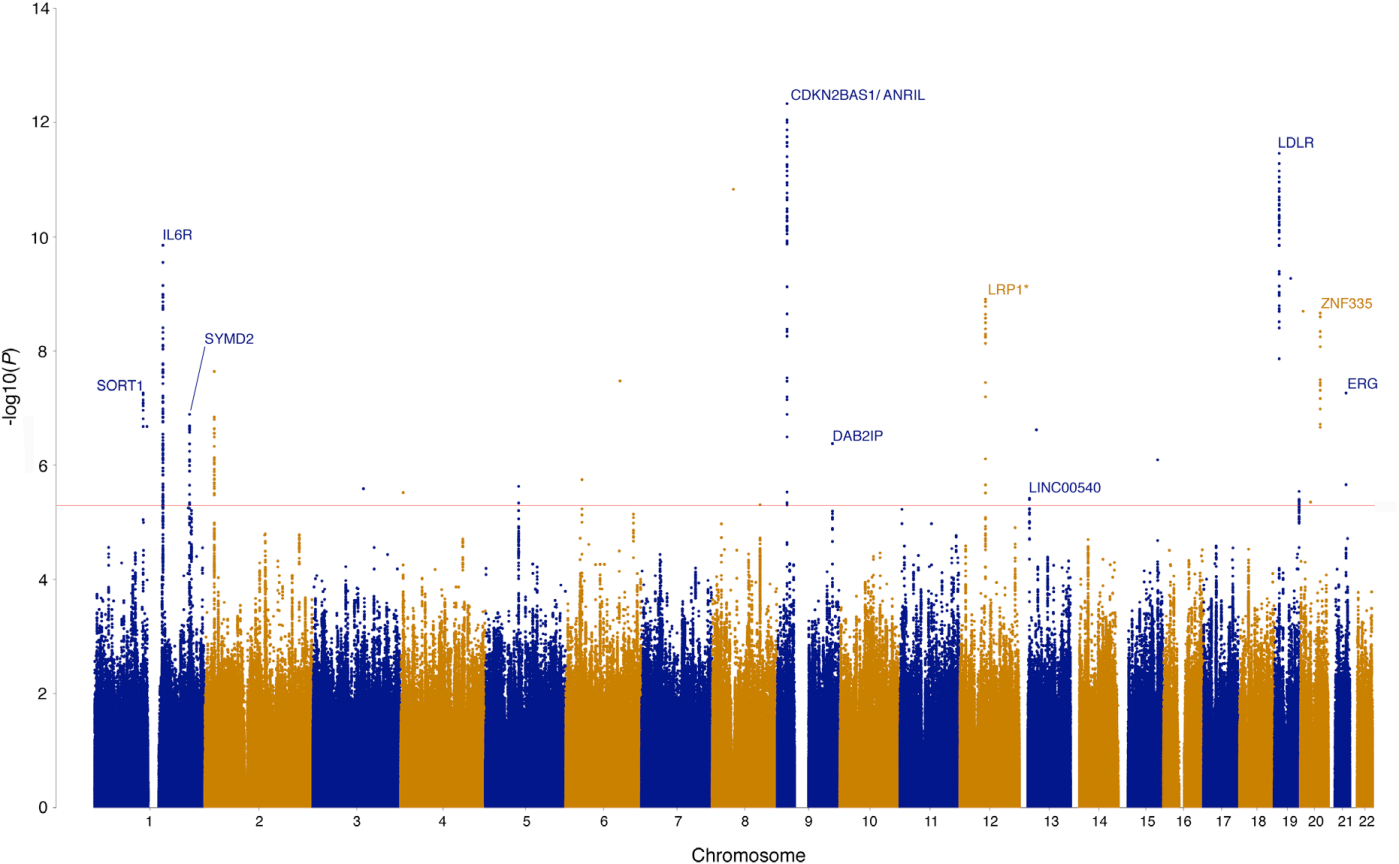
**Whole-genome association plot for the primary meta-analysis of genome-wide association studies of abdominal aortic aneurysm (AAA**). Data represent a meta-analysis of 4972 AAA cases and 99 858 controls. The horizontal line indicates the *P* value threshold of 5×10^−^^6^ used to select loci for validation studies. The 9 subsequently validated AAA loci are indicated along with the previously identified *LRP1* locus, which fell to *P*=6.4×10^–7^ in the combined discovery/ validation analysis (Online Tables III and IV).

**Figure 2.**
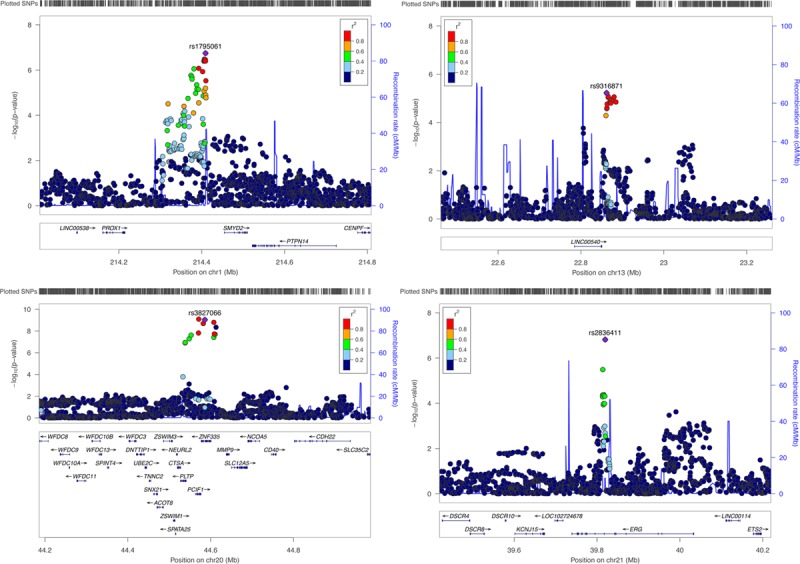
**Regional association plots for 4 new abdominal aortic aneurysm (AAA**) **genome-wide significant loci at 1q32.3, 13q12.11, 20q13.12, and 21q22.2.** New AAA genome-wide significant loci at 1q32.3 (near *SMYD2*), 13q12.11 (*LINC00540*), 20q13.12 (near *MMP9/ZNF335*), and 21q22.2 (*ERG*). −log_10_ (*P*_fixed_) values for single nucleotide polymorphisms (SNPs) from the AAA discovery meta-analysis of 4972 cases and 99 858 controls were plotted against their genomic positions using LocusZoom (1000Genomes, EUR, November 2014). The peak SNP in each region is labeled (purple diamond), whereas the color indicates LD (*r*^2^) with the peak.

The meta-analysis of 6 GWAS data sets (4972 AAA cases; 99 858 controls; Online Table I) revealed 19 loci of interest (*P*<1×10^−^^6^, Online Tables III and IV; Figure 1). Lead SNPs from these loci, including the 6 AAA risk loci reported previously, were analyzed in a validation study of 5232 AAA cases and 7908 controls (Online Tables II, V, VI, and VII). Four new loci were independently significant (*P*<0.05) in the validation cohort, had a direction of effect consistent with the discovery cohort and when combined with the discovery cohort had a *P* value that surpassed a genome-wide significance (5×10^−^^8^): 1q32.3 (*SMYD2*), 13q12.11 (*LINC00540* [long intergenic nonprotein coding RNA 540]), 20q13.12 (near *PCIF1* [C-terminal inhibiting factor 1 of a protein called pancreatic and duodenal homeobox 1]/*MMP9* [matrix metalloproteinase 9]/*ZNF335* [zinc finger protein 335]), and 21q22.2 (*ERG* [v-ets avian erythroblastosis virus E26 oncogene homolog]; Table 1; Online Tables V, VI, and VII; Figure 2). All previously reported associations with AAA were confirmed at genome-wide significance (Table 1; Online Table VII; Online Figure II) with the exception of 12q13.3 (*LRP1*), where the lead SNP identified in this meta-analysis and tested in our validation study only demonstrated a borderline association with AAA in the combined analysis (*P*=6.4×10^−^^7^). There was evidence of significant heterogeneity in the results observed for rs1795061 (near *SMYD2*) and rs2836411 (*ERG*) (Online Table VII). A random-effects model sensitivity analysis (Han-Eskin^14^ method) demonstrated minimal effect on the results for these 2 loci (Online Table VIII). The lead SNPs at 2 loci that were both below the threshold for genome-wide significance under the fixed-effects model (rs6516091, 20p12.3, near *FERMT1* and rs5954362, Xq27.2, *SPANXA1*) were significant in the random-effects model. However, because both demonstrated extreme heterogeneity (*I*^2^ ≥ 0.7), we did not consider these to be newly identified loci for AAA and these were excluded from further analysis.

**Table 1.**
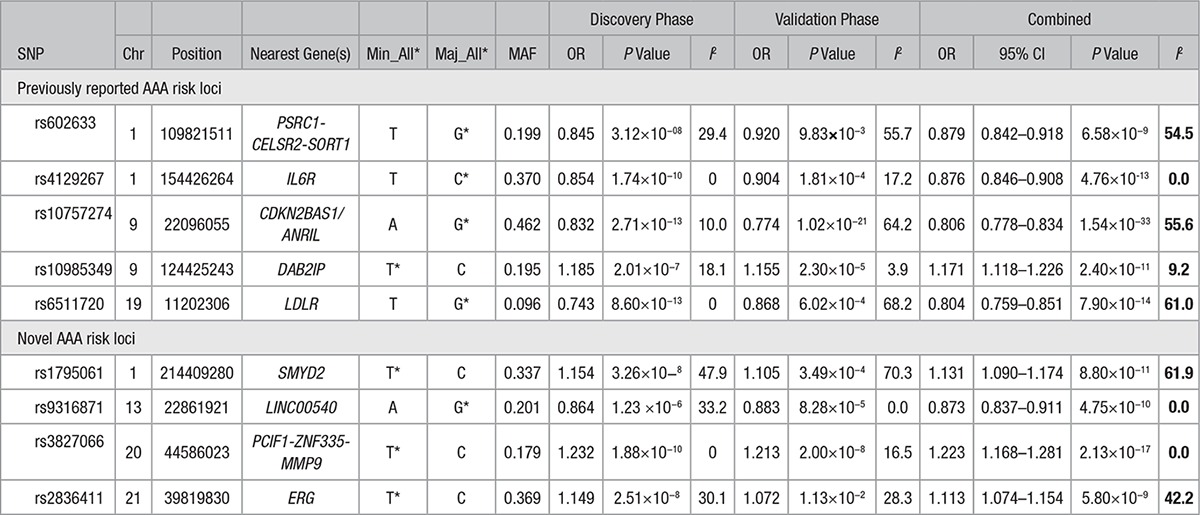
List of AAA Associated Loci Surpassing a Genome-Wide Significance Threshold After Combining GWAS data (4972 cases and 99 858 controls) and Validation Data (5232 cases and 7908 controls)

### New AAA Loci Seem to be Specific for AAA

**Figure 3.**
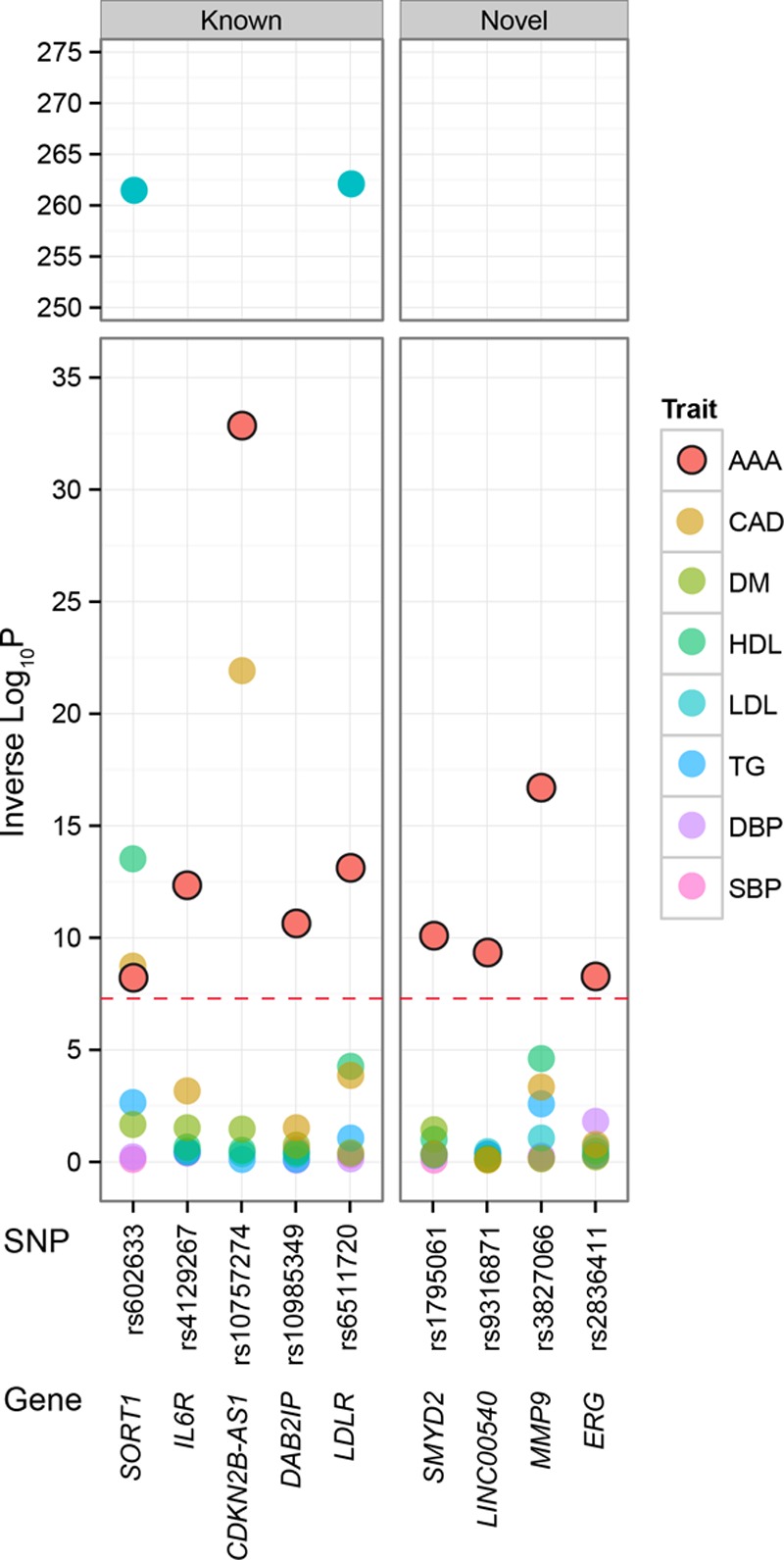
**Association between the lead single nucleotide polymorphisms (SNP) at the abdominal aortic aneurysm risk loci and association *P* values for other cardiovascular risk factors/traits (Online Table IX).** CAD indicates coronary artery disease; DBP, diastolic blood pressure; DM, diabetes mellitus; HDL, high-density lipoprotein; LDL, low-density lipoprotein; SBP, systolic blood pressure; and TG, triglyceride.

To assess whether the loci identified in our meta-analysis were specific to AAA or were also associated with diseases or risk factors known to be associated with AAA, we looked up results from GWAS of CAD,^6^ hypertension,^18^ and lipid traits.^17^ We also obtained results for diabetes mellitus^15^ to determine whether there was a reverse effect at these loci because diabetes mellitus is a negative risk factor for AAA and negatively influences AAA growth.^1^ Other than the known associations at 1p13.3 (*SORT1*), 9p21 (*CDKN2BAS1/ANRIL*) with CAD, 1p13.3 (*SORT1*) with high-density lipoprotein/LDL, and 19p13.2 (*LDLR*) with LDL, we observed no new associations between the lead SNPs at any of the AAA risk loci we had identified and these traits (Figure 3; Online Table IX). In particular, no association was observed between diabetes mellitus and these SNPs. Literature searching revealed an association between rs4845625 at 1q21.3 (*IL6R*) and CAD, but this was not in high linkage disequilibrium with the lead SNP genotyped in our study at this locus (*R*^2^=0.54).^36^

We also searched GWAS Central (database proving integrative visualization of and access to GWAS data) and Phenotype-Genotype Integrator and performed a GRASP^37^ analysis for any associations of the lead AAA SNPs with traits other than those listed above. We identified additional genome-wide significant associations between 1q21.3/*IL6R* (rs4129267) and C-reactive protein/asthma, and nominal associations between 1p13.3/*SORT1* (rs602633), 21q22.2/*ERG* (rs2836411), and 19p13.2/*LDLR* (rs6511720) and height (Online Tables X, XI, and XII), a potential risk factor for AAA.^38^

We also performed a PheWAS^21,22^ in the eMERGE data sets exploring the association between the 9 AAA-associated SNPs and an extensive group of diagnoses to identify novel associations and uncover potential pleiotropy. We considered identification of previously known associations, such as rs602633 associated with hyperglyceridemia and rs10757274 associated with CAD, to be indications that the PheWAS approach was robust. The PheWAS results demonstrated the known associations with CAD and lipid levels but did not identify any novel disease associations (Online Table XIII).

### Annotation of SNPs at AAA Loci

**Figure 4.**
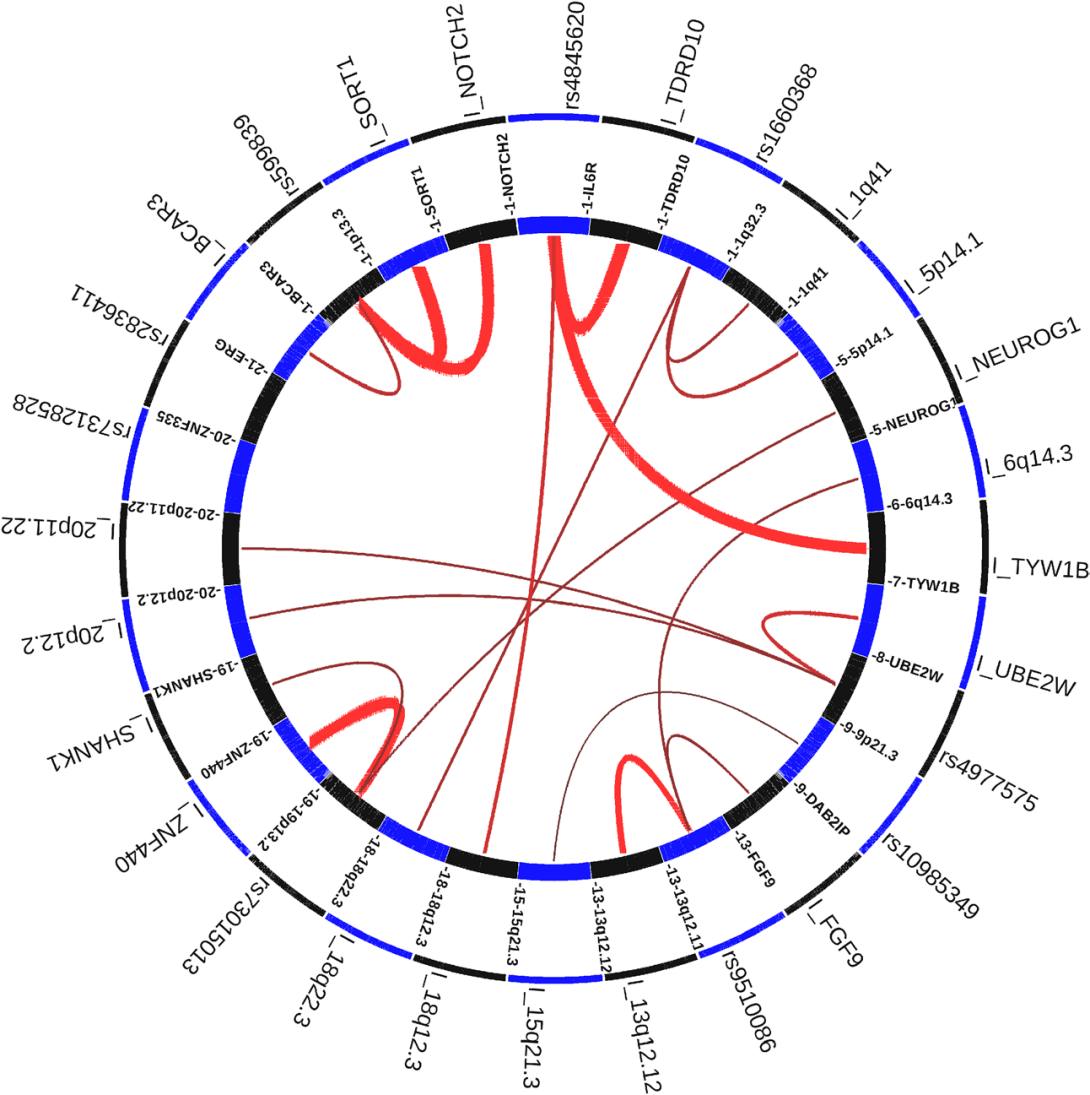
**Circle plot showing the lead single nucleotide polymorphism (SNP) distal interaction regions based on the 9 replicated abdominal aortic aneurysm genome-wide association study SNPs.** Top variants with highest regulatory signals and distal interaction regions are shown on the outer circle (significant regulatory variants are labeled with I). The inner circle shows genes and genomic loci, whereas the distal interactive signals are shown with red lines (width corresponds to intensity of interaction). Note the long-range interactions, such as that between variants associated with *IL6R* (rs4845620, 1q21.3) and *TYW1B* (7q11.23).

Annotation did not identify any nonsynonymous variants in high linkage disequilibrium (*R*^2^>0.5) with the lead SNPs at the AAA risk loci (Online Tables XIV and XV). Based on GWAS3D analysis, all 9 lead SNPs were associated with TF-binding site affinity variants (Online Tables XVI and XVII). Eight SNPs had potential long-range interactions with distal genomic regions (Figure 4). GWAS3D analysis also provided potential mechanistic insight for intergenic AAA variants such as rs9316871 (13q12.11) that had significant predicted regulatory variant interaction with *FGF9* (fibroblast growth factor 9; 13q12.11). In addition, although the AAA association with rs599839 (1p13.3) showed strong long-range chromatin interaction with SORT1 (as previously reported specifically in AAA^9^), it also had predicted distal interactions with other genes including *BCAR3* (breast cancer antiestrogen resistance 3; 1p22.1) and *NOTCH2* (notch 2 member of type 1 transmembrane protein family; 1p12-p11).

### DEPICT Gene Pathway Prediction

DEPICT identified 633 and 482 gene enrichment sets with nominal *P*<0.05 using the discovery meta-GWAS SNP set (*P*<5×10^−^^6^) and top 9 SNPs from the combined analysis, respectively. Only one of the gene sets (decreased long bone epiphyseal plate size) had a false discovery rate of <0.2. Gene set descriptions included multiple functional classes relevant to vascular biology, ie, transforming growth factor-β regulation, lipoprotein metabolism, inflammation-induced extracellular matrix remodeling (regulatory factor X1), vascular smooth muscle cell function, vascular injury including hemorrhage, immune cell function (particularly T and B cells), acute phase response including IL6 secretion, apoptosis, hyperglycemia and the phosphatidylinositol-4,5-bisphosphate 3-kinase catalytic subunit alpha, c-Jun N-terminal kinase, and mitogen-activated kinase-like protein cascades. In addition, there were multiple gene sets associated with long bone size and epiphyseal plate formation (Table 2; Online Table XVIII and Online Data File).

**Table 2.**
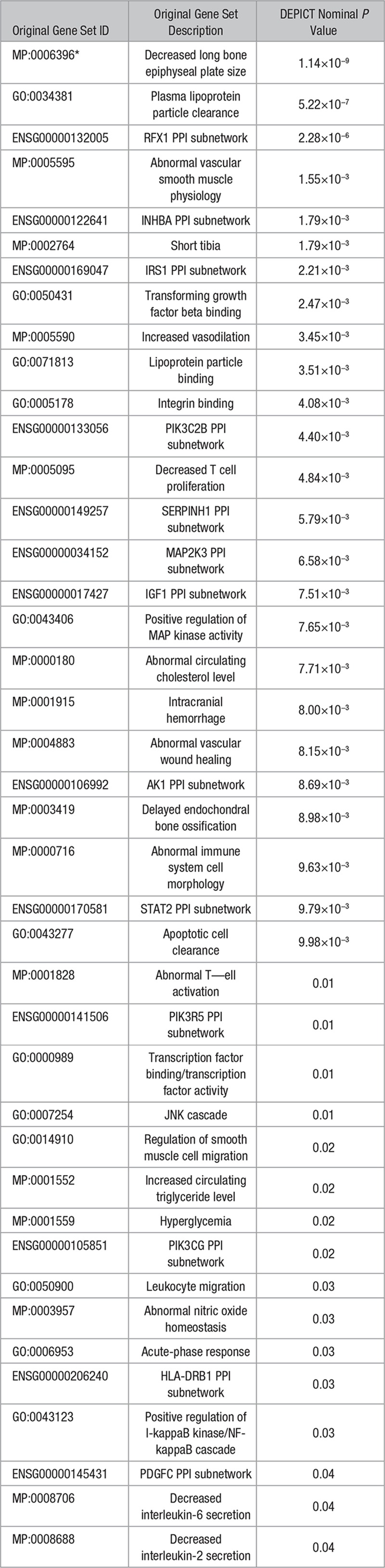
DEPICT Gene Enrichment Sets Based on the Top 10 Validated Loci

### Functional Effects of SNPs at AAA Loci

The lookup of SNPs at AAA loci in studies of functional effects included multitissue eQTL studies, vascular/monocyte-specific eQTL, and AAA-specific studies (mRNA expression and chromatin-immunoprecipitation-chip). These analyses revealed several potential functional associations (Online Tables XI, XX, XXI, and XXII; Online Figure III).^27,39^ Of most relevance to AAA, eQTLs were observed for rs3827066 (20q13.3) and *PLTP* (phospholipid transfer protein) expression in aortic tissue and for rs4129267 (1q21.3) and *IL6R* expression in mammary artery. RNA-Seq data also demonstrated independent eQTLs in mammary artery for 2 of the novel AAA associations we have identified: rs2836411 and *ERG* expression and rs9316871 and *FGF9* expression. All eQTLs, with the exception of rs9316871 and *FGF9* were also seen in tissues other than arterial samples.

Several GWAS3D-predicted distal interacting genes had significantly different mRNA expression between AAA and control samples (Table 3; Online Table XXIII and Figure IV).^30^ For example, *BCAR3* had decreased mRNA expression in AAA tissue (as did *SORT1* itself). In addition, although the closest gene to rs9316871, a long intergenic noncoding RNA (*LINC00540*), was not part of the mRNA data set, the predicted distal target *FGF9* had significantly increased mRNA expression in AAA tissue (Online Table XXIII).

**Table 3.**
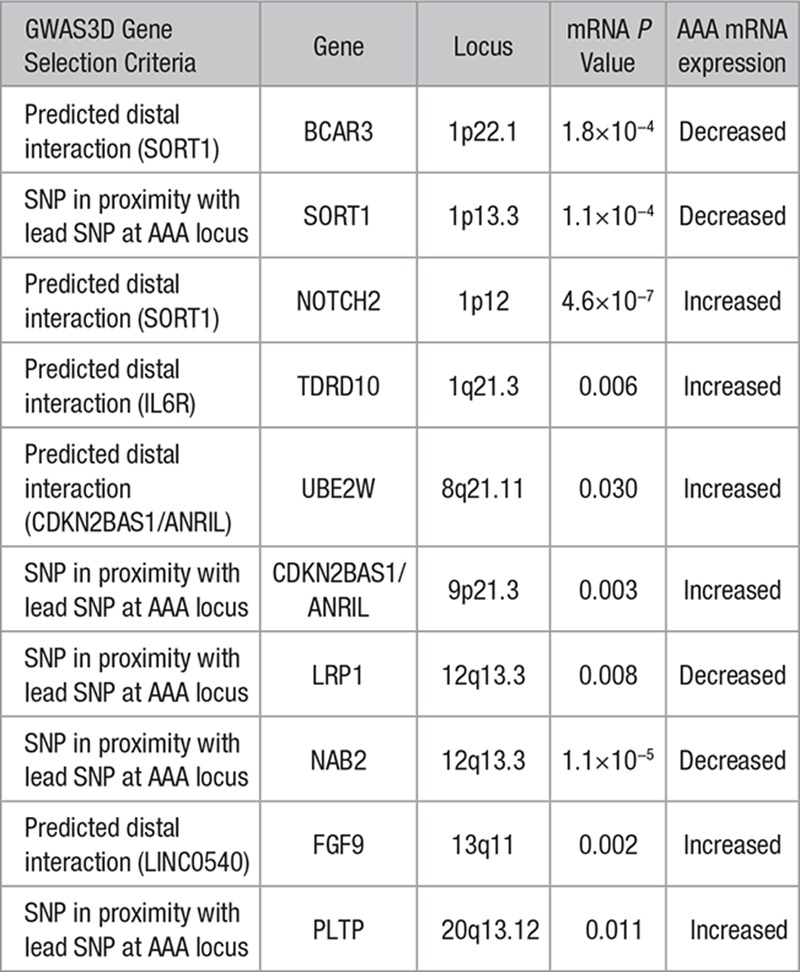
Genes Predicted by GWAS3D Analysis to Be Associated With Putative AAA Loci Identified in the Discovery Study Demonstrating Significantly Different mRNA Expression in Aneurysmal Aortic Wall Samples From 49 Patients With AAA Compared With 10 Organ Donor Control Aortic Samples

Chromatin-immunoprecipitation-chip data from human AAA tissue^31^ revealed TF-binding sites in 5 genes (*SMYD2, SORT1, CDKN2BAS1/ANRIL*, *ERG*, and *DAB2IP*), which harbor AAA risk loci, but none of these binding sites included the lead SNP tested for association with AAA (Online Table XXIV).

### Network Analysis Reveals a Central Role for Matrix Metalloproteinase 9

Network analysis using both IPA and Consensus PathDB demonstrated similar results (Online Figures V and VI). Both analyses revealed a central role for MMP9 in AAA, with IPA identifying direct interactions (physical contact between 2 molecules such as binding or phosphorylation) between ERG, IL6R and LDLR, and MMP9, and Consensus PathDB identifying a direct interaction between ERG and MMP9 with secondary interactions (interactions without physical contact, such as signaling events) between both SMYD2 and LDLR, and MMP9. On removing TNF from the analysis (which had been added based on the strong effect of SMYD2 on IL6 and TNF production^34,35^), the genes at AAA loci each remained in independent subnetworks. Inclusion of transforming growth factor-B1, implicated in thoracic aneurysms and Marfan syndrome, instead of TNF failed to coalesce the subnetworks. The long noncoding RNA ANRIL (*CDKN2BAS1*), our strongest hit in the genome (Figure 1), has been reported in numerous studies as a GWAS hotspot and a candidate gene for CAD, intracranial aneurysms, and diverse cardiometabolic disorders^40^; however, this was not represented in either the IPA or Consensus PathDB networks.

## Discussion

The present study is the largest genetic association study of AAA performed to date, utilizing 6 GWAS data sets for AAA with a total of 4972 cases and 99 858 controls. Furthermore, we used an independent validation set of 5232 AAA cases and 7908 controls and then performed a pooled analysis of all 10 204 cases and 107 766 controls. We confirmed the association of 5 previously reported loci and identified 4 new loci associated with AAA at genome-wide levels of significance. In contrast to previously identified loci, lead SNPs at the newly identified loci did not demonstrate evidence of cross-phenotype association with other cardiometabolic phenotypes. In summary, the genetic evidence to date mirrors that seen in the epidemiological literature where it is clear that AAA and other forms of cardiovascular diseases are seen as distinct but overlapping phenotypes.

Previous genetic discoveries in AAA have pointed to inflammation and immune function (*IL6R* and *CDKN2BAS1/ANRIL*) and low-density lipoprotein metabolism (*SORT1* and *LDLR*) as important mediators of AAA development. The genes at the novel AAA loci identified here are relevant to aneurysm biology, but their precise roles require further investigation. *MMP9* is within the 20q13.12 locus and matrix degradation via MMP9 is known to play a key role in the development of AAA, evidenced by the observation of high levels of MMP9 in end-stage disease specimens.^41^ This is also an important finding given the development of novel pharmacotherapies that target inflammation and matrix degradation pathways such as tofacitinib (a novel Janus kinase inhibitor). Although it is tempting to assume that MMP9 is the causal association at this locus, there are, however, other candidate genes at this locus. Examination of the region and the association pattern with AAA (Figure 2) shows that the strongest signals are seen upstream of *MMP9* and are separated from *MMP9* by a recombination hotspot. Closer to the strongest association signal are *ZNF335* and *PCIF1*. There is no literature evidence for any potential link for ZNF335 to AAA and the only identified genetic association of *ZNF335* is with celiac disease.^42^ Although rs181914932 is upstream and more proximal to PCIF1, it has been associated with the activity of PLTP,^43^ an adjacent gene in the same locus. Our eQTL analyses demonstrated an association between the lead SNP we assessed at this locus (rs3827066) and PLTP expression in aortic tissue (Online Tables XX and XXI). We have also shown that PLTP expression is significantly higher in aneurysmal aortic tissue than in control aorta (Online Table XXIII and Figure IV). PLTP plays a role in cholesterol transport. These data strengthen the evidence, particularly when taken together with the *SORT1* and *LDLR* associations confirmed here, that aberrations of lipid metabolism play a key role in the development of AAA.

The other novel AAA loci identified here contain *LINC00540*, *ERG*, and *SMYD2*. *LINC00540* is a long noncoding RNA with no currently known function; however, both our GWAS3D and eQTL analyses independently suggested an association with FGF9, which was also differentially expressed within AAA tissue. *ERG* encodes a TF that is normally present in hematopoietic and endothelial cells. ERG has a role in vascular endothelial growth factor/mitogen-activated kinase-like protein–mediated vascular development,^44^ as well as regulating angiogenesis, which is known to play a role in the development of AAA.^45,46^ ERG also plays a role in the embryonic development of the aorta,^44^ and it has been hypothesized that in utero aortic development has a role in the later development of an AAA.^47^ In prostate cancer, ERG has been shown to regulate the expression of MMP9.^48^ Taken together this limited evidence points to several potential roles by which ERG may influence the development of AAA and, along with our significant eQTL observations, strongly suggest that further work in this area is warranted.

The role of SMYD2 in AAA is less clear. SMYD2 regulates *HSP90* (heat shock protein 90) methylation,^49^ and the inhibition of heat shock protein 90 has been shown to reduce AAA formation in murine models,^50^ suggesting this as a possible link between SMYD2 and AAA. SMYD2 also plays a role in the differentiation of embryonic stem cells,^51^ again suggesting a possible role for aberrations of in utero aortic development influencing the risk of aortic disease later in life.

The integrated gene function analysis tool DEPICT identified numerous pathways that are potentially relevant to aneurysm pathogenesis (Table 2). In particular, we note with interest that the strongest predicted set was associated with long bone epiphyseal plate formation, which is possibly consistent with previous studies reporting tall stature as a risk factor for AAA^52^ and conversely short stature with occlusive CAD.^53,54^

Our network analyses using 2 different bioinformatics tools also revealed a central role for MMP9 in AAA, with IPA identifying direct interactions between ERG, IL6R and LDLR, and MMP9, and Consensus PathDB identifying a direct interaction between ERG and MMP9 with secondary interactions between both SMYD2 and LDLR and MMP9. These results suggest that the novel loci could act in concert, either synergistically or antagonistically, to affect the AAA phenotype, and provide hypotheses for future investigation using animal and cell culture models.

In this study, we did not replicate the association previously identified between *LRP1* and AAA.^7^ The samples from the original study that identified this association were included in this analysis, suggesting that this may have been a false-positive association. However, there is evidence supporting LRP1 as a biologically plausible candidate pathway for AAA.^55,56^ Variants at, or close to, LRP1 are also associated with other vascular/related phenotypes (aortic dissection,^57^ migraine,^58^ and lipid traits^17^). Because we observed a degree of heterogeneity at this locus in our analysis (Online Table VII), we consider that further investigation of this locus remains warranted despite our findings.

Our GWAS3D genome analysis predicted potential novel biological pathways in AAA pathogenesis. For example, *FGF9* was shown to have a possible distal interaction with the intergenic SNP rs9316871. FGF9, although not previously considered a strong candidate in AAA pathogenesis, was nevertheless at least partially validated by its increased mRNA expression in AAA tissue (Table 3). In AAA, both the medial and adventitial layers of the vessel wall are significantly more vascularized compared with nonaneurysmal tissue,^59^ and it is therefore interesting to note that FGF9 has been shown to enhance angiogenesis and neovascularization within mouse models of myocardial infarction.^60^

The main strength of this study is the inclusion of all currently available worldwide GWAS data sets for AAA and formation of an expanded International Aneurysm Consortium. We acknowledge several limitations in our work. The overall numbers of samples included in our analysis are lower than for more common traits such as diabetes mellitus^15^ and CAD.^16^ We also did not have an adequate number of females in our sample set to perform sex-specific analyses that may have been informative given the strong sexual dimorphism exhibited by AAA.^61^ We recognize this limitation, but the current focus of AAA screening programs on men alone^2,3^ and the much reduced prevalence of AAA in women means that collecting adequate samples for such analyses is likely to be challenging. Some of the contributing GWAS studies such as the Aneurysm Consortium GWAS were derived from multicenter sample collections that led to intercohort heterogeneity in clinical phenotyping of the case groups. Together with the limited covariate data available for the control groups in the GWAS studies that used population control samples, this led to an inability to reliably adjust for clinical covariates in our overall analysis. Given these limitations, and in particular about the numbers of samples available for analysis in AAA, alternative approaches for investigating the genetic cause of AAA need to be considered. The natural history of AAA with a long latent period (if detected early), during which patients are monitored by serial imaging studies, offers the opportunity to study disease progression as a continuous trait, leveraging additional power over discrete trait approaches for the limited sample sizes available.^37^

In conclusion, our meta-GWAS and the bioinformatics analyses, applying multiple techniques, has highlighted several potentially novel mechanisms of AAA pathobiology. These will require direct investigation in future studies to confirm their role in the development and progression of AAA.

## Acknowledgments

The Abdominal Aortic Aneurysm Consortium made use of the Welcome Trust Case Control Consortium data. The full list of investigators is available at www.wtccc.org.uk. Data on coronary artery disease/myocardial infarction were contributed by CARDIoGRAMplusC4D (Coronary ARtery DIsease Genome wide Replication and Meta-analysis) investigators and were downloaded from www.CARDIOGRAMPLUSC4D.ORG. Data on Blood Pressure were contributed by the International Consortium for Blood Pressure. Contributing members of all consortia are listed in the Online Supplement.

## Sources of Funding

The Welcome Trust Case Control Consortium project was funded by the Wellcome Trust (awards 076113 and 085475). The New Zealand project was funded by the Health Research Council of New Zealand (08–75, 14–155). Recruitment of abdominal aortic aneurysm patients and controls in Belgium, Canada, and Pittsburgh, USA, was funded in part by the National Heart, Lung, and Blood Institute, National Institutes of Health (HL064310 and HL044682). The Geisinger sample collection was funded in part by the Pennsylvania Commonwealth Universal Research Enhancement program, the Geisinger Clinical Research Fund, the American Heart Association, and the Ben Franklin Technology Development Fund of Pennsylvania. The Barts and the Leicester Cardiovascular Biomedical Research Units are funded by the National Institute for Health Research. The eMERGE (electronic Medical Records and Genomics) Network is funded by the National Human Genome Research Institute, with additional funding from the National Institute of General Medical Sciences through the following grants: U01HG004438 to Johns Hopkins University; U01HG004424 to The Broad Institute; U01HG004438 to CIDR; U01HG004610 and U01HG006375 to Group Health Cooperative; U01HG004608 to Marshfield Clinic; U01HG006389 to Essentia Institute of Rural Health; U01HG04599 and U01HG006379 to Mayo Clinic; U01HG004609 and U01HG006388 to Northwestern University; U01HG04603 and U01HG006378 to Vanderbilt University; U01HG006385 to the Coordinating Center; U01HG006382 to Geisinger Health System; U01HG006380 to Icahn School of Medicine Mount Sinai. The generation and management of genome-wide association study (GWAS) data for the Rotterdam Study (control samples for the Dutch GWAS) is supported by the Netherlands Organization of Scientific Research (NWO) Investments (175.010.2005.011, 911-03-012). This study is funded by the Research Institute for Diseases in the Elderly (014-93-015; RIDE2), the Netherlands Genomics Initiative/NWO project nr. 050-060-810. The Italian sample collection were funded by grants from Ente Cassa di Risparmio di Firenze to Fiorgen Foundation, Florence, Italy, and from the Italian Ministry of Health. Sample collections from Poland were funded in part by the National Science Centre in Poland (6P05A03921, NN403250440). The Mayo Vascular Disease Biorepository was funded by a Marriot Award for Individualized Medicine and an Award from the Mayo Center of Individualized Medicine. The Vanderbilt data set(s) were obtained from Vanderbilt University Medical Center’s BioVU supported by institutional funding and by the National Center for Research Resources (UL1 RR024975-01, which is now at the National Center for Advancing Translational Sciences, UL1 TR000445-06). The ASAP study (Advanced Study of Aortic Pathology) was supported by the Swedish Research Council, the Swedish Heart-Lung Foundation, the Leducq Foundation (MIBAVA), and a donation by Fredrik Lundberg. S.E. Humphries holds a Chair funded by the British Heart Foundation, and is supported by the British Heart Foundation (BHF; PG08/008) and by the National Institute for Health Research University College London Hospitals Biomedical Research Centre. The Cardiogenics project was supported by the European Union 6th Framework Programme (LSHM-CT-2006–037593). S.C. Harrison was funded by a BHF clinical training fellowship (FS/11/16/28696). The Stockholm-Tartu Atherosclerosis Reverse Network Engineering Task biobank and the generation of the RNASeq data set was funded by Astra-Zeneca Translational Science Centre-Karolinska Institutet, the University of Tartu (SP1GVARENG), the Estonian Research Council (ETF 8853), the Torsten and Ragnar Söderberg Foundation, the Knut and Alice Wallenberg Foundation, the American Heart Association (A14SFRN20840000) and by the National Institute of Health (R01HL71207).

## Appendix

From the Surgery Department (G.T.J., V.L.P., W.W., I.A.T., J.K., G.B.H., A.M.v.R.), Medicine Department (M.J.A.W.), and Biochemistry Department (T.R.M.), University of Otago, Dunedin, New Zealand; The Sigfried and Janet Weis Center for Research (G.T., H.K., R.E., K.M.B., D.T.S., D.J.C.) and Biomedical and Translational Informatics (M.D.R., S.P.), Geisinger Health System, Danville, PA; Division of Molecular Biology and Human Genetics, Department of Biomedical Sciences, Faculty of Medicine and Health Sciences, Stellenbosch University, Tygerberg, South Africa (G.T., H.K.); deCODE/Amgen, Reykjavik, Iceland (S.G., G.T., U.T., A.R., K.S.); Department of Medical Genetics (A.F.B., P.I.W.d.B.), and Department of Epidemiology, Julius Center for Health Sciences and Primary Care (P.I.W.d.B.), University Medical Center Utrecht, The Netherlands; Atherothrombotic Disease Center, Department of Experimental and Clinical Medicine (B.G., S.G., C.S., R.A., A.C.) and Vascular Surgery Unit, Department of Experimental and Clinical Medicine (R.P., C.P.), University of Florence, Careggi Hospital, Florence, Italy; Institute of Human Genetics, Polish Academy of Sciences, Faculty of Nucleic Acid Function, Poznan, Poland (E.S.); Department of General and Vascular Surgery, Poznan University of Medical Sciences, Poland (E.S., G.O.); Department of Cardiovascular Sciences (T.R.W., A.S., A.R.V., S.P.R.R., S.E.H., A.H.G., C.P.N., R.D.S., S.C.H., N.J.S., M.J.B.) and Department of Health Sciences (J.R.T.), University of Leicester, United Kingdom; NIHR Leicester Cardiovascular Biomedical Research Unit, Glenfield General Hospital, Leicester, United Kingdom (T.R.W., A.S., A.R.V., S.E.H., A.H.G., C.P.N., N.J.S., M.J.B.); The Pennsylvania State University, University Park, PA (M.D.R., A.V., S.P., S.S.V.); The Department of Vascular Surgery at Geisinger Medical Center, Danville, PA (J.R.E., E.J.R.); Mayo Clinic Rochester, MN (I.J.K., Z.Y., M.d.A.); Marshfield Clinic Research Foundation, WI (P.L.P.); Icahn School of Medicine at Mount Sinai (O.G., E.P.B.) and Department of Genetics and Genomic Sciences, Institute of Genomics and Multiscale Biology (O.F., E.E.S., J.L.M.B.), Icahn School of Medicine at Mount Sinai, New York, NY; Vanderbilt University Nashville, TN (J.M.); Northwestern University Feinberg School of Medicine, Chicago, IL (L.J.R.-T., J.A.P.); Department of Biomedical Informatics and Medical Education, University of Washington, Seattle (D.R.C.); Research Division, Essentia Institute of Rural Health, Duluth, MN (C.A.M.); Case Western Reserve University, Cleveland, OH (D.C.C.); Group Health Research Institute, Seattle, WA (D.S.C.); Department of Medical Genetics and Molecular Biochemistry, Temple University School of Medicine, PA (G.S.G.); Mission Clinic, Mission Health System, Asheville, NC (D.P.F.); Waikato Hospital, Hamilton, New Zealand (R.B., T.M.V.); Auckland City Hospital, New Zealand (A.A.H.); Surgery Department, University of Otago, Christchurch, New Zealand (D.R.L., J.R.); Genetics of Complex Traits in Humans Group, Wellcome Trust Sanger Institute, Cambridge, United Kingdom (S.B.), School of Medicine (S.A.B.) and Centre for Public Health (D.T.B.), Queens University Belfast, United Kingdom; Department of Vascular Surgery (R.E.C.) and Department of Vascular Surgery, Cardiovascular Division/British Heart Foundation Centre of Research Excellence (A.S.), King’s College London, United Kingdom; Department of Vascular Surgery, St George’s University of London, United Kingdom (G.C., M.M.T.); King Faisal Specialist Hospital and Research Centre, Jeddah, Saudi Arabia (H.H.); The Leeds Institute of Cardiovascular and Metabolic Medicine, University of Leeds, United Kingdom (D.J.A.S., T.S.F., S.P.R.R., K.B., K.J.G., M.A.B.); Department of Surgery, University of Western Australia, Crawley, Australia (F.M.v.B., P.E.N.); Laekning Medical Clinics, Reykjavik, Iceland (S.E.M.); University of Iceland, Faculty of Medicine, Reykjavik (U.T., K.S; Department of Vascular Surgery, VU Medical Center, Amsterdam, The Netherlands (J.D.B.); CAPHRI Research School, University Maastricht, Eindhoven, The Netherlands (J.A.W.T.); Department of Vascular Surgery, Catharina Ziekenhuis, Eindhoven, The Netherlands (J.A.W.T., M.R.v.S.); Department of Genetics, UMC Groningen, The Netherlands (C.W.); Radboud University Medical Centre, Radboud Institute for Health Sciences, Nijmegen, The Netherlands (J.d.G., L.A.K.); Elitary Research Centre of Individualized Medicine in Arterial Disease (CIMA), Department of Cardiothoracic and Vascular Surgery, Odense University Hospital, Denmark (J.S.L.); Belfast, United Kingdom (A.H.); William Harvey Research Institute, Barts and The London School of Medicine and Dentistry, Queen Mary University of London, United Kingdom (K.S., P.D.); Department of Haematology, University of Cambridge, United Kingdom (K.S.); Vascular Biology Unit, Queensland Research Centre for Peripheral Vascular Disease and the Department of Vascular and Endovascular Surgery, James Cook University and Townsville Hospital, Australia (J.G.); Department of Surgery and Cancer, Imperial College London, United Kingdom (J.T.P.); Cardiovascular Genetics, Institute of Cardiovascular Science, University College London, United Kingdom (S.E.H.); Surgical Research Center GIGA-Cardiovascular Science Unit, University of Liège, Belgium (N.S.); Department of Human Genetics, University of Pittsburgh School of Public Health, PA (R.E.F.); Atherosclerosis Research Unit, Center for Molecular Medicine, Department of Medicine (P.E., L.F.), Cardiothoracic Surgery Unit, Department of Molecular Medicine and Surgery (A.F.-C.), Department of Medical Biochemistry and Biophysics, Vascular Biology Unit (C.B.), Department of Medical Biochemistry and Biophysics (J.L.M.B.), Karolinska Institutet, Stockholm, Sweden; Center for Biological Sequence Analysis, Technical University of Denmark, Copenhagen, Denmark (L.F.); Center for Population Studies, National Heart, Lung, and Blood Institute, The Framingham Heart Study, MA (J.D.E., A.D.J.); Department of Immunology, Genetics and Pathology, Rudbeck Laboratory, Uppsala University, Sweden (C.B.); Department of Physiology, Institute of Biomedicine and Translation Medicine, University of Tartu, Estonia (J.L.M.B.); Department of Cardiac Surgery, Tartu University Hospital, Estonia (A.R.); Clinical Gene Networks AB, Stockholm, Sweden (A.R., O.F., J.L.M.B.); Department of Neurology (L.L.) and Center for Molecular Medicine and Genetics (A.M.D.), Wayne State University, Detroit, MI; Department of Vascular and Endovascular Surgery, Paracelsus Medical University Nuremberg, Germany (E.L.V.); Department of Surgery (Division of Vascular Surgery), University Medical Center Groningen, University of Groningen, The Netherlands (C.J.Z.); Chirurgencoöperatie Oost Nederland, Enschede, The Netherlands (R.H.G.); Department of Surgery, TweeSteden Hospital, Tilburg, The Netherlands (S.M.v.S.); Department of Vascular Surgery, Rijnstate Ziekenhuis, Arnhem, The Netherlands (S.M.v.S.); Department of Vascular Surgery, St. Antonius Hospital, Nieuwegein, The Netherlands (J.P.d.V.); and The Princess Al-Jawhara Al-Brahim Centre of Excellence in Research of Hereditary Disorders (PACER-HD), King Abdulaziz University, Jeddah, Saudi Arabia (P.D.).

## Disclosures

Johan L.M. Björkegren is founder and major shareholder in Clinical Gene Networks AB (CGN) together with Arno Ruusalepp. Björkegren, Ruusalepp, and Eric E Schadt are members of the board of directors. Clinical Gene Networks AB has an invested interest in the Stockholm-Tartu Atherosclerosis Reverse Network Engineering Task biobank and data set.

## Supplementary Material

## Novelty and Significance

### What Is Known?

- Abdominal aortic aneurysm (AAA) has a prevalence of ≈1.5% in men aged >65 years.
- Positive family history of AAA is a strong risk factor for AAA; however, only 6 robust and independently validated AAA genetic loci have been identified to date.

## References

[1] Lederle FA (2009). In the clinic. Abdominal aortic aneurysm.. Ann Intern Med.

[2] Guirguis-Blake JM, Beil TL, Senger CA, Whitlock EP (2014). Ultrasonography screening for abdominal aortic aneurysms: a systematic evidence review for the U.S. Preventive Services Task Force.. Ann Intern Med.

[3] Abdominal aortic aneurysm screening: 2014 to 2015 data. The NHS AAA Screening Programme.. https://www.gov.uk/government/publications/abdominal-aortic-aneurysm-screening-2014-to-2015-data.

[4] Wahlgren CM, Larsson E, Magnusson PK, Hultgren R, Swedenborg J (2010). Genetic and environmental contributions to abdominal aortic aneurysm development in a twin population.. J Vasc Surg.

[5] Larsson E, Granath F, Swedenborg J, Hultgren R (2009). A population-based case-control study of the familial risk of abdominal aortic aneurysm.. J Vasc Surg.

[6] Gretarsdottir S, Baas AF, Thorleifsson G (2010). Genome-wide association study identifies a sequence variant within the DAB2IP gene conferring susceptibility to abdominal aortic aneurysm.. Nat Genet.

[7] Bown MJ, Jones GT, Harrison SC, CARDIoGRAM Consortium; Global BPgen Consortium; DIAGRAM Consortium; VRCNZ Consortium (2011). Abdominal aortic aneurysm is associated with a variant in low-density lipoprotein receptor-related protein 1.. Am J Hum Genet.

[8] Bradley DT, Hughes AE, Badger SA (2013). A variant in LDLR is associated with abdominal aortic aneurysm.. Circ Cardiovasc Genet.

[9] Jones GT, Bown MJ, Gretarsdottir S (2013). A sequence variant associated with sortilin-1 (SORT1) on 1p13.3 is independently associated with abdominal aortic aneurysm.. Hum Mol Genet.

[10] Harrison SC, Smith AJ, Jones GT, Aneurysm Consortium (2013). Interleukin-6 receptor pathways in abdominal aortic aneurysm.. Eur Heart J.

[11] Helgadottir A, Thorleifsson G, Magnusson KP (2008). The same sequence variant on 9p21 associates with myocardial infarction, abdominal aortic aneurysm and intracranial aneurysm.. Nat Genet.

[12] Willer CJ, Li Y, Abecasis GR (2010). METAL: fast and efficient meta-analysis of genomewide association scans.. Bioinformatics.

[13] Shi YY, He L (2005). SHEsis, a powerful software platform for analyses of linkage disequilibrium, haplotype construction, and genetic association at polymorphism loci.. Cell Res.

[14] Han B, Eskin E (2011). Random-effects model aimed at discovering associations in meta-analysis of genome-wide association studies.. Am J Hum Genet.

[15] Morris AP, Voight BF, Teslovich TM, Wellcome Trust Case Control Consortium; Meta-Analyses of Glucose and Insulin-related traits Consortium (MAGIC) Investigators; Genetic Investigation of ANthropometric Traits (GIANT) Consortium; Asian Genetic Epidemiology Network–Type 2 Diabetes (AGEN-T2D) Consortium; South Asian Type 2 Diabetes (SAT2D) Consortium; DIAbetes Genetics Replication And Meta-analysis (DIAGRAM) Consortium (2012). Large-scale association analysis provides insights into the genetic architecture and pathophysiology of type 2 diabetes.. Nat Genet.

[16] Schunkert H, König IR, Kathiresan S, Cardiogenics; CARDIoGRAM Consortium (2011). Large-scale association analysis identifies 13 new susceptibility loci for coronary artery disease.. Nat Genet.

[17] Willer CJ, Schmidt EM, Sengupta S (2013). Discovery and refinement of loci associated with lipid levels.. Nat Genet.

[18] Wain LV, Verwoert GC, O’Reilly PF, LifeLines Cohort Study; EchoGen consortium; AortaGen Consortium; CHARGE Consortium Heart Failure Working Group; KidneyGen consortium; CKDGen consortium; Cardiogenics consortium; CardioGram (2011). Genome-wide association study identifies six new loci influencing pulse pressure and mean arterial pressure.. Nat Genet.

[19] Ramos EM, Hoffman D, Junkins HA, Maglott D, Phan L, Sherry ST, Feolo M, Hindorff LA (2014). Phenotype-Genotype Integrator (PheGenI): synthesizing genome-wide association study (GWAS) data with existing genomic resources.. Eur J Hum Genet.

[20] Leslie R, O’Donnell CJ, Johnson AD (2014). GRASP: analysis of genotype-phenotype results from 1390 genome-wide association studies and corresponding open access database.. Bioinformatics.

[21] Denny JC, Ritchie MD, Basford MA, Pulley JM, Bastarache L, Brown-Gentry K, Wang D, Masys DR, Roden DM, Crawford DC (2010). PheWAS: demonstrating the feasibility of a phenome-wide scan to discover gene-disease associations.. Bioinformatics.

[22] Pendergrass SA, Brown-Gentry K, Dudek S (2013). Phenome-wide association study (PheWAS) for detection of pleiotropy within the Population Architecture using Genomics and Epidemiology (PAGE) Network.. PLoS Genet.

[23] Gottesman O, Kuivaniemi H, Tromp G, eMERGE Network (2013). The Electronic Medical Records and Genomics (eMERGE) Network: past, present, and future.. Genet Med.

[24] Reumers J, Conde L, Medina I, Maurer-Stroh S, Van Durme J, Dopazo J, Rousseau F, Schymkowitz J (2008). Joint annotation of coding and non-coding single nucleotide polymorphisms and mutations in the SNPeffect and PupaSuite databases.. Nucleic Acids Res.

[25] Li MJ, Wang LY, Xia Z, Sham PC, Wang J (2013). GWAS3D: Detecting human regulatory variants by integrative analysis of genome-wide associations, chromosome interactions and histone modifications.. Nucleic Acids Res.

[26] Pers TH, Karjalainen JM, Chan Y, Genetic Investigation of ANthropometric Traits (GIANT) Consortium (2015). Biological interpretation of genome-wide association studies using predicted gene functions.. Nat Commun.

[27] Folkersen L, van’t Hooft F, Chernogubova E, Agardh HE, Hansson GK, Hedin U, Liska J, Syvänen AC, Paulsson-Berne G, Paulssson-Berne G, Franco-Cereceda A, Hamsten A, Gabrielsen A, Eriksson P, BiKE and ASAP Study Groups (2010). Association of genetic risk variants with expression of proximal genes identifies novel susceptibility genes for cardiovascular disease.. Circ Cardiovasc Genet.

[28] Björkegren JL, Kovacic JC, Dudley JT, Schadt EE (2015). Genome-wide significant loci: how important are they? Systems genetics to understand heritability of coronary artery disease and other common complex disorders.. J Am Coll Cardiol.

[29] Kuivaniemi H, Platsoucas CD, Tilson MD (2008). Aortic aneurysms: an immune disease with a strong genetic component.. Circulation.

[30] Biros E, Gäbel G, Moran CS, Schreurs C, Lindeman JH, Walker PJ, Nataatmadja M, West M, Holdt LM, Hinterseher I, Pilarsky C, Golledge J (2015). Differential gene expression in human abdominal aortic aneurysm and aortic occlusive disease.. Oncotarget.

[31] Pahl MC, Erdman R, Kuivaniemi H, Lillvis JH, Elmore JR, Tromp G (2015). Transcriptional (ChIP-Chip) analysis of ELF1, ETS2, RUNX1 and STAT5 in human abdominal aortic aneurysm.. Int J Mol Sci.

[32] Kamburov A, Stelzl U, Lehrach H, Herwig R (2013). The ConsensusPathDB interaction database: 2013 update.. Nucleic Acids Res.

[33] Pentchev K, Ono K, Herwig R, Ideker T, Kamburov A (2010). Evidence mining and novelty assessment of protein-protein interactions with the ConsensusPathDB plugin for Cytoscape.. Bioinformatics.

[34] Nguyen H, Allali-Hassani A, Antonysamy S (2015). LLY-507, a cell-active, potent, and selective inhibitor of protein-lysine methyltransferase SMYD2.. J Biol Chem.

[35] Xu G, Liu G, Xiong S, Liu H, Chen X, Zheng B (2015). The histone methyltransferase Smyd2 is a negative regulator of macrophage activation by suppressing interleukin 6 (IL-6) and tumor necrosis factor α (TNF-α) production.. J Biol Chem.

[36] Deloukas P, Kanoni S, Willenborg C (2013). Large-scale association analysis identifies new risk loci for coronary artery disease.. Nat Genet.

[37] Eicher JD, Landowski C, Stackhouse B, Sloan A, Chen W, Jensen N, Lien JP, Leslie R, Johnson AD (2015). GRASP v2.0: an update on the Genome-Wide Repository of Associations between SNPs and phenotypes.. Nucleic Acids Res.

[38] Smelser DT, Tromp G, Elmore JR, Kuivaniemi H, Franklin DP, Kirchner HL, Carey DJ (2014). Population risk factor estimates for abdominal aortic aneurysm from electronic medical records: a case control study.. BMC Cardiovasc Disord.

[39] Zhang X, Gierman HJ, Levy D, Plump A, Dobrin R, Goring HH, Curran JE, Johnson MP, Blangero J, Kim SK, O’Donnell CJ, Emilsson V, Johnson AD (2014). Synthesis of 53 tissue and cell line expression QTL datasets reveals master eQTLs.. BMC Genomics.

[40] Hannou SA, Wouters K, Paumelle R, Staels B (2015). Functional genomics of the CDKN2A/B locus in cardiovascular and metabolic disease: what have we learned from GWASs?. Trends Endocrinol Metab.

[41] Pearce WH, Shively VP (2006). Abdominal aortic aneurysm as a complex multifactorial disease: interactions of polymorphisms of inflammatory genes, features of autoimmunity, and current status of MMPs.. Ann N Y Acad Sci.

[42] Coleman C, Quinn EM, Ryan AW (2016). Common polygenic variation in coeliac disease and confirmation of ZNF335 and NIFA as disease susceptibility loci.. Eur J Hum Genet.

[43] Kim DS, Burt AA, Ranchalis JE, Vuletic S, Vaisar T, Li WF, Rosenthal EA, Dong W, Eintracht JF, Motulsky AG, Brunzell JD, Albers JJ, Furlong CE, Jarvik GP (2015). PLTP activity inversely correlates with CAAD: effects of PON1 enzyme activity and genetic variants on PLTP activity.. J Lipid Res.

[44] Wythe JD, Dang LT, Devine WP, Boudreau E, Artap ST, He D, Schachterle W, Stainier DY, Oettgen P, Black BL, Bruneau BG, Fish JE (2013). ETS factors regulate Vegf-dependent arterial specification.. Dev Cell.

[45] Choke E, Cockerill GW, Dawson J, Wilson RW, Jones A, Loftus IM, Thompson MM (2006). Increased angiogenesis at the site of abdominal aortic aneurysm rupture.. Ann N Y Acad Sci.

[46] Choke E, Thompson MM, Dawson J, Wilson WR, Sayed S, Loftus IM, Cockerill GW (2006). Abdominal aortic aneurysm rupture is associated with increased medial neovascularization and overexpression of proangiogenic cytokines.. Arterioscler Thromb Vasc Biol.

[47] Norman PE, Powell JT (2010). Site specificity of aneurysmal disease.. Circulation.

[48] Tian TV, Tomavo N, Huot L, Flourens A, Bonnelye E, Flajollet S, Hot D, Leroy X, de Launoit Y, Duterque-Coquillaud M (2014). Identification of novel TMPRSS2:ERG mechanisms in prostate cancer metastasis: involvement of MMP9 and PLXNA2.. Oncogene.

[49] Du SJ, Tan X, Zhang J (2014). SMYD proteins: key regulators in skeletal and cardiac muscle development and function.. Anat Rec (Hoboken).

[50] Qi J, Yang P, Yi B, Huo Y, Chen M, Zhang J, Sun J (2015). Heat shock protein 90 inhibition by 17-DMAG attenuates abdominal aortic aneurysm formation in mice.. Am J Physiol Heart Circ Physiol.

[51] Sesé B, Barrero MJ, Fabregat MC, Sander V, Izpisua Belmonte JC (2013). SMYD2 is induced during cell differentiation and participates in early development.. Int J Dev Biol.

[52] Reed D, Reed C, Stemmermann G, Hayashi T (1992). Are aortic aneurysms caused by atherosclerosis?. Circulation.

[53] Paajanen TA, Oksala NK, Kuukasjärvi P, Karhunen PJ (2010). Short stature is associated with coronary heart disease: a systematic review of the literature and a meta-analysis.. Eur Heart J.

[54] Nelson CP, Hamby SE, Saleheen D, CARDIoGRAM+C4D Consortium (2015). Genetically determined height and coronary artery disease.. N Engl J Med.

[55] Chan CY, Chan YC, Cheuk BL, Cheng SW (2013). A pilot study on low-density lipoprotein receptor-related protein-1 in Chinese patients with abdominal aortic aneurysm.. Eur J Vasc Endovasc Surg.

[56] Muratoglu SC, Belgrave S, Hampton B, Migliorini M, Coksaygan T, Chen L, Mikhailenko I, Strickland DK (2013). LRP1 protects the vasculature by regulating levels of connective tissue growth factor and HtrA1.. Arterioscler Thromb Vasc Biol.

[57] Guo DC, Grove ML, Prakash SK, GenTAC Investigators; BAVCon Investigators (2016). Genetic variants in LRP1 and ULK4 are associated with acute aortic dissections.. Am J Hum Genet.

[58] Chasman DI, Schürks M, Anttila V (2011). Genome-wide association study reveals three susceptibility loci for common migraine in the general population.. Nat Genet.

[59] Jones GT, Grundmann R (2011). The pathohistology of abdominal aortic aneurysm.. In: Diagnosis, Screening and Treatment of Abdominal, Thoracoabdominal and Thoracic Aortic Aneurysms.

[60] Singla D, Wang J (2016). Fibroblast growth factor-9 activates c-Kit progenitor cells and enhances angiogenesis in the infarcted diabetic heart.. Oxid Med Cell Longev.

[61] Bloomer LD, Bown MJ, Tomaszewski M (2012). Sexual dimorphism of abdominal aortic aneurysms: a striking example of “male disadvantage” in cardiovascular disease.. Atherosclerosis.

