# Supplementary material for: Meta-Analysis of Genome-Wide Association Studies for Abdominal Aortic Aneurysm Identifies Four New Disease-Specific Risk Loci
Source: Circ Res. 2017 Jan 19;120(2):341–53. doi: 10.1161/CIRCRESAHA.116.308765 (PMC5253231; doi:10.1161/CIRCRESAHA.116.308765)
Supplement: Supplementary file 1 [file res-120-341-s001.pdf]

Original gene set ID	Original gene set description	Nominal P value
MP:0006396	decreased long bone epiphyseal plate size	1.14E-09
GO:0034381	plasma lipoprotein particle clearance	5.22E-07
ENSG00000205250	E2F4 PPI subnetwork	1.27E-06
ENSG00000132005	RFX1 PPI subnetwork	2.28E-06
MP:0000708	thymus hyperplasia	6.32E-06
ENSG00000167553	TUBA1C PPI subnetwork	3.18E-05
ENSG00000170421	KRT8 PPI subnetwork	9.59E-05
MP:0003645	increased pancreatic beta cell number	1.12E-04
ENSG00000166866	MYO1A PPI subnetwork	2.32E-04
REACTOME_APOPTOTIC_EXECUTION__PHASE	REACTOME_APOPTOTIC_EXECUTION__PHASE	2.92E-04
MP:0008182	decreased marginal zone B cell number	3.06E-04
GO:0008375	acetylglucosaminyltransferase activity	3.62E-04
ENSG00000131941	RHPN2 PPI subnetwork	3.75E-04
ENSG00000169710	FASN PPI subnetwork	4.55E-04
REACTOME_APOPTOTIC_CLEAVAGE_OF_CELLULAR_PROTEINS	REACTOME_APOPTOTIC_CLEAVAGE_OF_CELLULAR_PROTEINS	5.46E-04
ENSG00000013297	CLDN11 PPI subnetwork	6.28E-04
ENSG00000070159	PTPN3 PPI subnetwork	6.56E-04
ENSG00000091409	ITGA6 PPI subnetwork	7.26E-04
ENSG00000178209	PLEC PPI subnetwork	8.57E-04
REACTOME_P75_NTR_RECEPTOR:MEDIATED_SIGNALLING	REACTOME_P75_NTR_RECEPTOR:MEDIATED_SIGNALLING	1.07E-03
GO:0001890	placenta development	1.08E-03
ENSG00000164344	KLKB1 PPI subnetwork	1.09E-03
MP:0002136	abnormal kidney physiology	1.17E-03
MP:0002655	abnormal keratinocyte morphology	1.45E-03
ENSG00000143375	CGN PPI subnetwork	1.48E-03
MP:0005595	abnormal vascular smooth muscle physiology	1.55E-03
ENSG00000122641	INHBA PPI subnetwork	1.79E-03
MP:0002764	short tibia	1.79E-03
MP:0003662	abnormal long bone epiphyseal plate proliferative zone	2.01E-03
ENSG00000169047	IRS1 PPI subnetwork	2.21E-03
ENSG00000125503	PPP1R12C PPI subnetwork	2.23E-03
MP:0001179	thick pulmonary interalveolar septum	2.30E-03
GO:0043256	laminin complex	2.33E-03
ENSG00000116809	ZBTB17 PPI subnetwork	2.46E-03
GO:0050431	transforming growth factor beta binding	2.47E-03
ENSG00000039560	RAI14 PPI subnetwork	2.60E-03
ENSG00000164733	CTSB PPI subnetwork	2.64E-03
ENSG00000139567	ACVRL1 PPI subnetwork	2.75E-03
MP:0005590	increased vasodilation	3.45E-03
GO:0071813	lipoprotein particle binding	3.51E-03
GO:0071814	protein-lipid complex binding	3.51E-03
MP:0002082	postnatal lethality	3.53E-03
GO:0071902	positive regulation of protein serine/threonine kinase activity	3.87E-03
ENSG00000130147	SH3BP4 PPI subnetwork	3.91E-03
GO:0005178	integrin binding	4.08E-03
ENSG00000133056	PIK3C2B PPI subnetwork	4.40E-03
ENSG00000172725	CORO1B PPI subnetwork	4.45E-03
ENSG00000136286	MYO1G PPI subnetwork	4.65E-03
ENSG00000078142	PIK3C3 PPI subnetwork	4.72E-03

Original gene set ID	Original gene set description	Nominal P value
MP:0005095	decreased T cell proliferation	4.84E-03
ENSG00000145715	RASA1 PPI subnetwork	4.96E-03
ENSG00000104725	ENSG00000104725 PPI subnetwork	5.08E-03
KEGG_PATHWAYS_IN_CANCER	KEGG_PATHWAYS_IN_CANCER	5.17E-03
GO:0008194	UDP-glycosyltransferase activity	5.46E-03
ENSG00000078747	ITCH PPI subnetwork	5.48E-03
ENSG00000149257	SERPINH1 PPI subnetwork	5.79E-03
ENSG00000114062	UBE3A PPI subnetwork	5.85E-03
ENSG00000139144	PIK3C2G PPI subnetwork	5.85E-03
ENSG00000143393	PI4KB PPI subnetwork	5.87E-03
ENSG00000148498	PARD3 PPI subnetwork	6.00E-03
ENSG00000196455	PIK3R4 PPI subnetwork	6.19E-03
ENSG00000148660	CAMK2G PPI subnetwork	6.48E-03
ENSG00000034152	MAP2K3 PPI subnetwork	6.58E-03
ENSG00000123124	WWP1 PPI subnetwork	6.95E-03
MP:0008813	decreased common myeloid progenitor cell number	7.37E-03
ENSG00000204175	GPRIN2 PPI subnetwork	7.39E-03
GO:0001772	immunological synapse	7.40E-03
REACTOME_CASPASE:MEDIATED_CLEAVAGE_OF_CYTOSKELETAL_PROTEINS	REACTOME_CASPASE:MEDIATED_CLEAVAGE_OF_CYTOSKELETAL_PROTEINS	7.46E-03
ENSG00000017427	IGF1 PPI subnetwork	7.51E-03
MP:0001954	respiratory distress	7.56E-03
GO:0016051	carbohydrate biosynthetic process	7.65E-03
GO:0043406	positive regulation of MAP kinase activity	7.65E-03
REACTOME_CELL_DEATH_SIGNALLING_VIA_NRAGE_NRIF_AND_NADE	REACTOME_CELL_DEATH_SIGNALLING_VIA_NRAGE_NRIF_AND_NADE	7.67E-03
MP:0000180	abnormal circulating cholesterol level	7.71E-03
ENSG00000170759	KIF5B PPI subnetwork	7.79E-03
ENSG00000180530	NRIP1 PPI subnetwork	7.86E-03
ENSG00000138771	SHROOM3 PPI subnetwork	7.89E-03
ENSG00000065882	TBC1D1 PPI subnetwork	7.97E-03
ENSG00000138592	USP8 PPI subnetwork	7.99E-03
MP:0001915	intracranial hemorrhage	8.00E-03
ENSG00000131746	TNS4 PPI subnetwork	8.01E-03
MP:0004883	abnormal vascular wound healing	8.15E-03
ENSG00000091073	ENSG00000091073 PPI subnetwork	8.22E-03
ENSG00000081189	MEF2C PPI subnetwork	8.24E-03
ENSG00000154415	PPP1R3A PPI subnetwork	8.33E-03
ENSG00000188313	PLSCR1 PPI subnetwork	8.55E-03
MP:0004933	abnormal epididymis epithelium morphology	8.61E-03
ENSG00000147065	MSN PPI subnetwork	8.64E-03
ENSG00000165409	TSHR PPI subnetwork	8.64E-03
ENSG00000106992	AK1 PPI subnetwork	8.69E-03
GO:0007292	female gamete generation	8.93E-03
ENSG00000144061	NPHP1 PPI subnetwork	8.95E-03
MP:0003419	delayed endochondral bone ossification	8.98E-03
ENSG00000110880	CORO1C PPI subnetwork	9.04E-03
ENSG00000197879	MYO1C PPI subnetwork	9.22E-03
ENSG00000176476	CCDC101 PPI subnetwork	9.31E-03
ENSG00000176108	CHMP6 PPI subnetwork	9.44E-03
REACTOME_INTEGRIN_CELL_SURFACE_INTERACTIONS	REACTOME_INTEGRIN_CELL_SURFACE_INTERACTIONS	9.47E-03

Original gene set ID	Original gene set description	Nominal P value
GO:0016758	transferase activity, transferring hexosyl groups	9.49E-03
ENSG00000103197	TSC2 PPI subnetwork	9.49E-03
MP:0003909	increased eating behavior	9.57E-03
MP:0000716	abnormal immune system cell morphology	9.63E-03
MP:0008803	abnormal placental labyrinth vasculature morphology	9.67E-03
ENSG00000137801	THBS1 PPI subnetwork	9.71E-03
ENSG00000130522	JUND PPI subnetwork	9.72E-03
GO:0005088	Ras guanyl-nucleotide exchange factor activity	9.78E-03
ENSG00000170581	STAT2 PPI subnetwork	9.79E-03
ENSG00000173757	STAT5B PPI subnetwork	9.84E-03
GO:0043277	apoptotic cell clearance	9.98E-03
GO:0006917	induction of apoptosis	0.01
MP:0001828	abnormal T cell activation	0.01
ENSG00000171241	SHCBP1 PPI subnetwork	0.01
REACTOME_APOPTOSIS	REACTOME_APOPTOSIS	0.01
MP:0011106	partial embryonic lethality before somite formation	0.01
GO:0030169	low-density lipoprotein particle binding	0.01
MP:0003731	abnormal retinal outer nuclear layer morphology	0.01
MP:0009400	decreased skeletal muscle fiber size	0.01
ENSG00000137693	YAP1 PPI subnetwork	0.01
REACTOME_NRAGE_SIGNALS_DEATH_THROUGH_JNK	REACTOME_NRAGE_SIGNALS_DEATH_THROUGH_JNK	0.01
ENSG00000145794	MEGF10 PPI subnetwork	0.01
MP:0008478	increased spleen white pulp amount	0.01
MP:0005079	defective cytotoxic T cell cytolysis	0.01
ENSG00000141506	PIK3R5 PPI subnetwork	0.01
GO:0000989	transcription factor binding transcription factor activity	0.01
MP:0002161	abnormal fertility/fecundity	0.01
GO:0040029	regulation of gene expression, epigenetic	0.01
ENSG00000115963	RND3 PPI subnetwork	0.01
GO:0043236	laminin binding	0.01
ENSG00000211660	ENSG00000211660 PPI subnetwork	0.01
ENSG00000211653	ENSG00000211653 PPI subnetwork	0.01
ENSG00000160310	PRMT2 PPI subnetwork	0.01
ENSG00000127688	GAN PPI subnetwork	0.01
ENSG00000167711	SERPINF2 PPI subnetwork	0.01
GO:0043491	protein kinase B signaling cascade	0.01
ENSG00000136068	FLNB PPI subnetwork	0.01
GO:0002020	protease binding	0.01
ENSG00000198053	SIRPA PPI subnetwork	0.01
ENSG00000182319	SGK223 PPI subnetwork	0.01
ENSG00000174292	TNK1 PPI subnetwork	0.01
ENSG00000132825	PPP1R3D PPI subnetwork	0.01
GO:0051015	actin filament binding	0.01
ENSG00000140443	IGF1R PPI subnetwork	0.01
MP:0000281	abnormal interventricular septum morphology	0.01
ENSG00000067560	RHOA PPI subnetwork	0.01
MP:0006094	increased fat cell size	0.01
ENSG00000197555	SIPA1L1 PPI subnetwork	0.01
ENSG00000183386	FHL3 PPI subnetwork	0.01

Original gene set ID	Original gene set description	Nominal P value
MP:0003229	abnormal vitelline vasculature morphology	0.01
MP:0001231	abnormal epidermis stratum basale morphology	0.01
MP:0000511	abnormal intestinal mucosa morphology	0.01
KEGG_ACUTE_MYELOID_LEUKEMIA	KEGG_ACUTE_MYELOID_LEUKEMIA	0.01
GO:0007254	JNK cascade	0.01
GO:0008624	induction of apoptosis by extracellular signals	0.01
GO:0000988	protein binding transcription factor activity	0.02
MP:0002452	abnormal antigen presenting cell physiology	0.02
ENSG00000185950	IRS2 PPI subnetwork	0.02
KEGG_LEUKOCYTE_TRANSENDOTHELIAL_MIGRATION	KEGG_LEUKOCYTE_TRANSENDOTHELIAL_MIGRATION	0.02
GO:0051568	histone H3-K4 methylation	0.02
ENSG00000165516	KLHDC2 PPI subnetwork	0.02
REACTOME_CELL_SURFACE_INTERACTIONS_AT_THE_VASCULAR_WALL	REACTOME_CELL_SURFACE_INTERACTIONS_AT_THE_VASCULAR_WALL	0.02
ENSG00000198838	RYR3 PPI subnetwork	0.02
MP:0001711	abnormal placenta morphology	0.02
GO:0014910	regulation of smooth muscle cell migration	0.02
ENSG00000104960	PTOV1 PPI subnetwork	0.02
GO:0001968	fibronectin binding	0.02
GO:0012502	induction of programmed cell death	0.02
ENSG00000100364	KIAA0930 PPI subnetwork	0.02
ENSG00000165410	CFL2 PPI subnetwork	0.02
MP:0004994	abnormal brain wave pattern	0.02
MP:0001552	increased circulating triglyceride leve	0.02
ENSG00000116141	MARK1 PPI subnetwork	0.02
GO:0032403	protein complex binding	0.02
ENSG00000179364	PACS2 PPI subnetwork	0.02
MP:0001559	hyperglycemia	0.02
ENSG00000105851	PIK3CG PPI subnetwork	0.02
MP:0004031	insulitis	0.02
MP:0010124	decreased bone mineral content	0.02
ENSG00000165197	FIGF PPI subnetwork	0.02
ENSG00000126561	STAT5A PPI subnetwork	0.02
ENSG00000136156	ITM2B PPI subnetwork	0.02
ENSG00000164327	RICTOR PPI subnetwork	0.02
ENSG00000159166	LAD1 PPI subnetwork	0.02
MP:0001716	abnormal placenta labyrinth morphology	0.02
MP:0002427	disproportionate dwarf	0.02
ENSG00000105371	ICAM4 PPI subnetwork	0.02
ENSG00000165476	REEP3 PPI subnetwork	0.02
GO:0046625	sphingolipid binding	0.02
MP:0000585	kinked tail	0.02
MP:0000889	abnormal cerebellar molecular layer	0.02
ENSG00000105699	LSR PPI subnetwork	0.02
GO:0005545	1-phosphatidylinositol binding	0.02
MP:0001134	absent corpus luteum	0.02
ENSG00000100097	LGALS1 PPI subnetwork	0.02
MP:0002079	increased circulating insulin level	0.02
ENSG00000150093	ITGB1 PPI subnetwork	0.02
GO:0001871	pattern binding	0.02

Original gene set ID	Original gene set description	Nominal P value
GO:0030247	polysaccharide binding	0.02
ENSG00000126934	MAP2K2 PPI subnetwork	0.02
ENSG00000110395	CBL PPI subnetwork	0.02
ENSG00000179151	EDC3 PPI subnetwork	0.02
ENSG00000154162	CDH12 PPI subnetwork	0.02
ENSG00000184363	PKP3 PPI subnetwork	0.02
ENSG00000020577	SAMD4A PPI subnetwork	0.02
MP:0004139	abnormal gastric parietal cell morphology	0.02
ENSG00000168476	REEP4 PPI subnetwork	0.02
ENSG00000110651	CD81 PPI subnetwork	0.02
ENSG00000134184	GSTM1 PPI subnetwork	0.02
ENSG00000105376	ICAM5 PPI subnetwork	0.02
ENSG00000196954	CASP4 PPI subnetwork	0.02
MP:0003704	abnormal hair follicle development	0.02
ENSG00000050820	BCAR1 PPI subnetwork	0.02
ENSG00000151748	SAV1 PPI subnetwork	0.02
GO:0003714	transcription corepressor activity	0.02
ENSG00000115904	SOS1 PPI subnetwork	0.02
ENSG00000175793	SFN PPI subnetwork	0.02
ENSG00000100345	MYH9 PPI subnetwork	0.02
GO:0035091	phosphatidylinositol binding	0.02
ENSG00000149930	TAOK2 PPI subnetwork	0.02
GO:0042054	histone methyltransferase activity	0.02
MP:0000689	abnormal spleen morphology	0.02
GO:0001892	embryonic placenta development	0.02
ENSG00000130294	KIF1A PPI subnetwork	0.02
ENSG00000148965	SAA4 PPI subnetwork	0.02
GO:0034774	secretory granule lumen	0.02
ENSG00000166483	WEE1 PPI subnetwork	0.02
ENSG00000110237	ARHGEF17 PPI subnetwork	0.02
GO:0032608	interferon-beta production	0.02
ENSG00000152518	ZFP36L2 PPI subnetwork	0.02
MP:0010792	abnormal stomach mucosa morphology	0.02
ENSG00000189319	FAM53B PPI subnetwork	0.02
ENSG00000117461	PIK3R3 PPI subnetwork	0.02
GO:0034362	low-density lipoprotein particle	0.02
ENSG00000134072	CAMK1 PPI subnetwork	0.02
ENSG00000163362	C1orf106 PPI subnetwork	0.02
MP:0002816	colitis	0.02
GO:0050900	leukocyte migration	0.03
GO:0044304	main axon	0.03
ENSG00000071909	MYO3B PPI subnetwork	0.03
ENSG00000100714	MTHFD1 PPI subnetwork	0.03
ENSG00000198836	OPA1 PPI subnetwork	0.03
ENSG00000197442	MAP3K5 PPI subnetwork	0.03
ENSG00000206306	HLA-DRB1 PPI subnetwork	0.03
ENSG00000206240	HLA-DRB1 PPI subnetwork	0.03
GO:0031983	vesicle lumen	0.03
KEGG_REGULATION_OF_ACTIN_CYTOSKELETON	KEGG_REGULATION_OF_ACTIN_CYTOSKELETON	0.03

Original gene set ID	Original gene set description	Nominal P value
GO:0004713	protein tyrosine kinase activity	0.03
GO:0006953	acute-phase response	0.03
GO:0003712	transcription cofactor activity	0.03
MP:0000295	trabecula carnea hypoplasia	0.03
ENSG00000105647	PIK3R2 PPI subnetwork	0.03
GO:0060205	cytoplasmic membrane-bounded vesicle lumen	0.03
ENSG00000107566	ERLIN1 PPI subnetwork	0.03
ENSG00000114270	COL7A1 PPI subnetwork	0.03
ENSG00000135930	EIF4E2 PPI subnetwork	0.03
MP:0006413	increased T cell apoptosis	0.03
ENSG00000211949	ENSG00000211949 PPI subnetwork	0.03
ENSG00000125731	SH2D3A PPI subnetwork	0.03
MP:0000414	alopecia	0.03
ENSG00000160691	SHC1 PPI subnetwork	0.03
MP:0001282	short vibrissae	0.03
MP:0003996	clonic seizures	0.03
ENSG00000019991	HGF PPI subnetwork	0.03
MP:0010025	decreased total body fat amount	0.03
GO:0007568	aging	0.03
GO:0042809	vitamin D receptor binding	0.03
MP:0005331	insulin resistance	0.03
GO:0045682	regulation of epidermis development	0.03
MP:0001923	reduced female fertility	0.03
MP:0001219	thick epidermis	0.03
ENSG00000068615	REEP1 PPI subnetwork	0.03
ENSG00000171219	CDC42BPG PPI subnetwork	0.03
MP:0009583	increased keratinocyte proliferatior	0.03
ENSG00000105810	CDK6 PPI subnetwork	0.03
ENSG00000105662	CRTC1 PPI subnetwork	0.03
MP:0003957	abnormal nitric oxide homeostasis	0.03
KEGG_SMALL_CELL_LUNG_CANCER	KEGG_SMALL_CELL_LUNG_CANCER	0.03
GO:0030669	clathrin-coated endocytic vesicle membrane	0.03
ENSG00000100030	MAPK1 PPI subnetwork	0.03
GO:0046328	regulation of JNK cascade	0.03
GO:0014070	response to organic cyclic compound	0.03
GO:0033500	carbohydrate homeostasis	0.03
GO:0042593	glucose homeostasis	0.03
REACTOME_PTM_GAMMA_CARBOXYLATION_HYPUSINE_FORMATION_AND_AF	REACTOME_PTM_GAMMA_CARBOXYLATION_HYPUSINE_FORMATION_AND_ARYLS	0.03
REACTOME_REGULATION_OF_SIGNALING_BY_CBL	REACTOME_REGULATION_OF_SIGNALING_BY_CBL	0.03
MP:0002418	increased susceptibility to viral infectior	0.03
MP:0003721	increased tumor growth/size	0.03
GO:0071845	cellular component disassembly at cellular leve	0.03
GO:0030518	intracellular steroid hormone receptor signaling pathway	0.03
ENSG00000116824	CD2 PPI subnetwork	0.03
MP:0003566	abnormal cell adhesion	0.03
GO:0034061	DNA polymerase activity	0.03
ENSG00000141968	VAV1 PPI subnetwork	0.03
GO:0001701	in utero embryonic development	0.03
MP:0000166	abnormal chondrocyte morphology	0.03

Original gene set ID	Original gene set description	Nominal P value
MP:0003400	kinked neural tube	0.03
GO:0000790	nuclear chromatin	0.03
ENSG00000197102	DYNC1H1 PPI subnetwork	0.03
GO:0043566	structure-specific DNA binding	0.03
ENSG00000075413	MARK3 PPI subnetwork	0.03
GO:0000271	polysaccharide biosynthetic process	0.03
REACTOME_CELL:CELL_COMMUNICATION	REACTOME_CELL:CELL_COMMUNICATION	0.03
MP:0000410	waved hair	0.03
ENSG00000154556	SORBS2 PPI subnetwork	0.03
ENSG00000104368	PLAT PPI subnetwork	0.03
GO:0043123	positive regulation of I-kappaB kinase/NF-kappaB cascade	0.03
ENSG00000156127	BATF PPI subnetwork	0.03
ENSG00000132470	ITGB4 PPI subnetwork	0.03
GO:0038024	cargo receptor activity	0.03
ENSG00000100014	SPECC1L PPI subnetwork	0.03
ENSG00000163083	INHBB PPI subnetwork	0.03
ENSG00000110931	CAMKK2 PPI subnetwork	0.03
MP:0002088	abnormal embryonic growth/weight/body size	0.03
GO:0016571	histone methylation	0.03
GO:0033559	unsaturated fatty acid metabolic process	0.03
MP:0005350	increased susceptibility to autoimmune disorder	0.03
ENSG00000136111	TBC1D4 PPI subnetwork	0.03
REACTOME_NEPHRIN_INTERACTIONS	REACTOME_NEPHRIN_INTERACTIONS	0.03
ENSG00000182195	LDOC1 PPI subnetwork	0.03
ENSG00000123685	BATF3 PPI subnetwork	0.03
ENSG00000215699	ENSG00000215699 PPI subnetwork	0.03
GO:0005720	nuclear heterochromatin	0.03
ENSG00000092969	TGFB2 PPI subnetwork	0.03
KEGG_ECM_RECEPTOR_INTERACTION	KEGG_ECM_RECEPTOR_INTERACTION	0.03
MP:0001201	translucent skin	0.03
GO:0016278	lysine N-methyltransferase activity	0.03
GO:0016279	protein-lysine N-methyltransferase activity	0.03
ENSG00000196586	MYO6 PPI subnetwork	0.03
GO:0004702	receptor signaling protein serine/threonine kinase activity	0.03
ENSG00000072518	MARK2 PPI subnetwork	0.03
ENSG00000165025	SYK PPI subnetwork	0.03
MP:0002109	abnormal limb morphology	0.03
ENSG00000157764	BRAF PPI subnetwork	0.03
ENSG00000152256	PDK1 PPI subnetwork	0.03
ENSG00000065618	COL17A1 PPI subnetwork	0.04
ENSG00000169220	RGS14 PPI subnetwork	0.04
ENSG00000100311	PDGFB PPI subnetwork	0.04
ENSG00000134202	GSTM3 PPI subnetwork	0.04
ENSG00000142515	KLK3 PPI subnetwork	0.04
MP:0002619	abnormal lymphocyte morphology	0.04
ENSG00000161395	PGAP3 PPI subnetwork	0.04
ENSG00000145431	PDGFC PPI subnetwork	0.04
ENSG00000170962	PDGFD PPI subnetwork	0.04
ENSG00000153879	CEBPG PPI subnetwork	0.04

Original gene set ID	Original gene set description	Nominal P value
ENSG00000077380	DYNC1I2 PPI subnetwork	0.04
ENSG00000197122	SRC PPI subnetwork	0.04
MP:0004399	abnormal cochlear outer hair cell morphology	0.04
ENSG00000174996	KLC2 PPI subnetwork	0.04
MP:0002376	abnormal dendritic cell physiology	0.04
MP:0000709	enlarged thymus	0.04
MP:0008706	decreased interleukin-6 secretion	0.04
MP:0004686	decreased length of long bones	0.04
GO:0050810	regulation of steroid biosynthetic process	0.04
ENSG00000138396	ENSG00000138396 PPI subnetwork	0.04
ENSG00000148400	NOTCH1 PPI subnetwork	0.04
ENSG00000137171	KLC4 PPI subnetwork	0.04
ENSG00000196396	PTPN1 PPI subnetwork	0.04
ENSG00000148672	GLUD1 PPI subnetwork	0.04
GO:0000975	regulatory region DNA binding	0.04
GO:0001067	regulatory region nucleic acid binding	0.04
GO:0022411	cellular component disassembly	0.04
ENSG00000026025	VIM PPI subnetwork	0.04
ENSG00000061273	HDAC7 PPI subnetwork	0.04
ENSG00000104067	TJP1 PPI subnetwork	0.04
MP:0004813	absent linear vestibular evoked potentia	0.04
ENSG00000091136	LAMB1 PPI subnetwork	0.04
KEGG_RENAL_CELL_CARCINOMA	KEGG_RENAL_CELL_CARCINOMA	0.04
KEGG_FOCAL_ADHESION	KEGG_FOCAL_ADHESION	0.04
GO:0031581	hemidesmosome assembly	0.04
ENSG00000141068	KSR1 PPI subnetwork	0.04
MP:0004214	abnormal long bone diaphysis morphology	0.04
ENSG00000123836	PFKFB2 PPI subnetwork	0.04
ENSG00000168090	COPS6 PPI subnetwork	0.04
ENSG00000132356	PRKAA1 PPI subnetwork	0.04
GO:0031093	platelet alpha granule lumen	0.04
GO:0048545	response to steroid hormone stimulus	0.04
MP:0003109	short femur	0.04
ENSG00000113758	DBN1 PPI subnetwork	0.04
GO:0008276	protein methyltransferase activity	0.04
MP:0003383	abnormal gluconeogenesis	0.04
ENSG00000162614	NEXN PPI subnetwork	0.04
ENSG00000162614	NEXN PPI subnetwork	0.04
ENSG00000169641	LUZP1 PPI subnetwork	0.04
MP:0002152	abnormal brain morphology	0.04
ENSG00000204257	HLA-DMA PPI subnetwork	0.04
ENSG00000206229	ENSG00000206229 PPI subnetwork	0.04
ENSG00000206293	ENSG00000206293 PPI subnetwork	0.04
ENSG00000138439	FAM117B PPI subnetwork	0.04
GO:0006636	unsaturated fatty acid biosynthetic process	0.04
ENSG00000176444	CLK2 PPI subnetwork	0.04
MP:0000703	abnormal thymus morphology	0.04
REACTOME_ZINC_TRANSPORTERS	REACTOME_ZINC_TRANSPORTERS	0.04
ENSG00000125952	MAX PPI subnetwork	0.04

Original gene set ID	Original gene set description	Nominal P value
GO:0046456	icosanoid biosynthetic process	0.04
ENSG00000132964	CDK8 PPI subnetwork	0.04
MP:0008688	decreased interleukin-2 secretion	0.04
ENSG00000196218	RYR1 PPI subnetwork	0.04
MP:0004770	abnormal synaptic vesicle recycling	0.04
ENSG00000121879	PIK3CA PPI subnetwork	0.04
ENSG00000196735	HLA-DQA1 PPI subnetwork	0.04
MP:0009355	increased liver triglyceride level	0.04
MP:0009399	increased skeletal muscle fiber size	0.04
ENSG00000160678	S100A1 PPI subnetwork	0.04
ENSG00000064999	ANKS1A PPI subnetwork	0.04
ENSG00000173327	MAP3K11 PPI subnetwork	0.04
GO:0051183	vitamin transporter activity	0.04
GO:0006690	icosanoid metabolic process	0.04
ENSG00000134363	FST PPI subnetwork	0.04
GO:0060053	neurofilament cytoskeleton	0.04
ENSG00000151914	DST PPI subnetwork	0.04
ENSG00000189079	ARID2 PPI subnetwork	0.04
ENSG00000065559	MAP2K4 PPI subnetwork	0.05
ENSG00000120709	FAM53C PPI subnetwork	0.05
MP:0002110	abnormal digit morphology	0.05
GO:0005976	polysaccharide metabolic process	0.05
ENSG00000054523	KIF1B PPI subnetwork	0.05
ENSG00000100906	NFKBIA PPI subnetwork	0.05
ENSG00000136518	ACTL6A PPI subnetwork	0.05
GO:0004709	MAP kinase kinase kinase activity	0.05
GO:0060711	labyrinthine layer development	0.05
KEGG_CIRCADIAN_RHYTHM_MAMMAL	KEGG_CIRCADIAN_RHYTHM_MAMMAL	0.05
REACTOME_CLASSICAL_ANTIBODY:MEDIATED_COMPLEMENT_ACTIVATION	REACTOME_CLASSICAL_ANTIBODY:MEDIATED_COMPLEMENT_ACTIVATION	0.05
ENSG00000211979	ENSG00000211979 PPI subnetwork	0.05
ENSG00000211973	ENSG00000211973 PPI subnetwork	0.05
ENSG00000172534	HCFC1 PPI subnetwork	0.05
ENSG00000136270	TBRG4 PPI subnetwork	0.05
GO:0032648	regulation of interferon-beta production	0.05
GO:0034375	high-density lipoprotein particle remodeling	0.05
ENSG00000185811	IKZF1 PPI subnetwork	0.05
ENSG00000198802	ENSG00000198802 PPI subnetwork	0.05
MP:0006262	testis tumor	0.05
ENSG00000171992	SYNPO PPI subnetwork	0.05
ENSG00000213341	CHUK PPI subnetwork	0.05
ENSG00000175197	DDIT3 PPI subnetwork	0.05
MP:0005150	cachexia	0.05
GO:0043122	regulation of I-kappaB kinase/NF-kappaB cascade	0.05
GO:0097006	regulation of plasma lipoprotein particle levels	0.05
ENSG00000162772	ATF3 PPI subnetwork	0.05
GO:0000122	negative regulation of transcription from RNA polymerase II promoter	0.05
GO:0005858	axonemal dynein complex	0.05
ENSG00000051382	PIK3CB PPI subnetwork	0.05
GO:0043405	regulation of MAP kinase activity	0.05

Original gene set ID	Original gene set description	Nominal P value
MP:0008722	abnormal chemokine secretion	0.05
KEGG_CHRONIC_MYELOID_LEUKEMIA	KEGG_CHRONIC_MYELOID_LEUKEMIA	0.05
REACTOME_REGULATED_PROTEOLYSIS_OF_P75NTR	REACTOME_REGULATED_PROTEOLYSIS_OF_P75NTR	0.05
GO:0043588	skin development	0.05
GO:0010627	regulation of intracellular protein kinase cascade	0.05
GO:0044212	transcription regulatory region DNA binding	0.05
GO:0030027	lamellipodium	0.05
ENSG00000105976	MET PPI subnetwork	0.05
MP:0002792	abnormal retinal vasculature morphology	0.05
MP:0000069	kyphoscoliosis	0.05
GO:0034339	regulation of transcription from RNA polymerase II promoter by nuclear hormone re	0.05
ENSG00000141551	CSNK1D PPI subnetwork	0.05
MP:0005108	abnormal ulna morphology	0.05
MP:0002419	abnormal innate immunity	0.05
GO:0016757	transferase activity, transferring glycosyl groups	0.05
ENSG00000161800	RACGAP1 PPI subnetwork	0.05
MP:0006387	abnormal T cell number	0.05
GO:0005089	Rho guanyl-nucleotide exchange factor activity	0.05
ENSG00000117984	CTSD PPI subnetwork	0.05
ENSG00000105971	CAV2 PPI subnetwork	0.05
ENSG00000115085	ZAP70 PPI subnetwork	0.05
MP:0004609	vertebral fusion	0.05
ENSG00000135862	LAMC1 PPI subnetwork	0.05
MP:0003449	abnormal intestinal goblet cell morphology	0.05
MP:0002687	oligozoospermia	0.05
MP:0000714	increased thymocyte number	0.05
ENSG00000133030	MPRIP PPI subnetwork	0.05
ENSG00000079841	RIMS1 PPI subnetwork	0.05
ENSG00000130638	ATXN10 PPI subnetwork	0.05
MP:0002656	abnormal keratinocyte differentiation	0.05
ENSG00000129691	ASH2L PPI subnetwork	0.05
MP:0002650	abnormal ameloblast morphology	0.05
ENSG00000135503	ACVR1B PPI subnetwork	0.05
GO:0004715	non-membrane spanning protein tyrosine kinase activity	0.05
ENSG00000001497	LAS1L PPI subnetwork	0.05
GO:0018024	histone-lysine N-methyltransferase activity	0.05
GO:0000792	heterochromatin	0.05
ENSG00000111961	SASH1 PPI subnetwork	0.05
MP:0008840	abnormal spike wave discharge	0.05
ENSG00000139514	SLC7A1 PPI subnetwork	0.05
GO:0007249	I-kappaB kinase/NF-kappaB cascade	0.05
MP:0009886	failure of palatal shelf elevation	0.06
ENSG00000144668	ITGA9 PPI subnetwork	0.06
MP:0001798	impaired macrophage phagocytosis	0.06
MP:0004148	increased compact bone thickness	0.06
GO:0034637	cellular carbohydrate biosynthetic process	0.06
REACTOME_PLATELET_SENSITIZATION_BY_LDL	REACTOME_PLATELET_SENSITIZATION_BY_LDL	0.06
GO:0008170	N-methyltransferase activity	0.06
GO:0046890	regulation of lipid biosynthetic process	0.06

Original gene set ID	Original gene set description	Nominal P value
ENSG00000173511	VEGFB PPI subnetwork	0.06
REACTOME_ACTIVATION_OF_CHAPERONE_GENES_BY_XBP1S	REACTOME_ACTIVATION_OF_CHAPERONE_GENES_BY_XBP1S	0.06
GO:0009925	basal plasma membrane	0.06
ENSG00000163486	SRGAP2 PPI subnetwork	0.06
REACTOME_CREATION_OF_C4_AND_C2_ACTIVATORS	REACTOME_CREATION_OF_C4_AND_C2_ACTIVATORS	0.06
GO:0007044	cell-substrate junction assembly	0.06
ENSG00000042429	MED17 PPI subnetwork	0.06
GO:0044445	cytosolic part	0.06
ENSG00000166949	SMAD3 PPI subnetwork	0.06
GO:0009311	oligosaccharide metabolic process	0.06
MP:0009549	decreased platelet aggregation	0.06
MP:0001510	abnormal coat appearance	0.06
GO:0005911	cell-cell junction	0.06
GO:0070302	regulation of stress-activated protein kinase signaling cascade	0.06
GO:0001776	leukocyte homeostasis	0.06
MP:0008752	abnormal tumor necrosis factor level	0.06
GO:0003887	DNA-directed DNA polymerase activity	0.06
ENSG00000173156	RHOD PPI subnetwork	0.06
ENSG00000121966	CXCR4 PPI subnetwork	0.06
ENSG00000095637	SORBS1 PPI subnetwork	0.06
GO:0071702	organic substance transport	0.06
GO:0030374	ligand-dependent nuclear receptor transcription coactivator activity	0.06
ENSG00000137154	RPS6 PPI subnetwork	0.06
ENSG00000113721	PDGFRB PPI subnetwork	0.06
GO:0042800	histone methyltransferase activity (H3-K4 specific)	0.06
ENSG00000091436	ENSG00000091436 PPI subnetwork	0.06
ENSG00000164050	PLXNB1 PPI subnetwork	0.06
GO:0034968	histone lysine methylation	0.06
GO:0006921	cellular component disassembly involved in apoptotic process	0.06
ENSG00000168502	CCDC165 PPI subnetwork	0.06
MP:0006267	abnormal intercalated disc morphology	0.06
ENSG00000206294	ENSG00000206294 PPI subnetwork	0.06
GO:0043616	keratinocyte proliferation	0.06
GO:0005543	phospholipid binding	0.06
ENSG00000120693	SMAD9 PPI subnetwork	0.06
ENSG00000006747	SCIN PPI subnetwork	0.06
GO:0008013	beta-catenin binding	0.06
ENSG00000008735	MAPK8IP2 PPI subnetwork	0.06
ENSG00000184371	CSF1 PPI subnetwork	0.06
ENSG00000041982	TNC PPI subnetwork	0.06
MP:0000547	short limbs	0.06
MP:0008079	decreased CD8-positive T cell number	0.06
ENSG00000057294	PKP2 PPI subnetwork	0.06
ENSG00000150630	VEGFC PPI subnetwork	0.06
ENSG00000101213	PTK6 PPI subnetwork	0.06
MP:0000512	intestinal ulcer	0.06
REACTOME_ZINC_INFLUX_INTO_CELLS_BY_THE_SLC39_GENE_FAMILY	REACTOME_ZINC_INFLUX_INTO_CELLS_BY_THE_SLC39_GENE_FAMILY	0.06
MP:0005342	abnormal intestinal lipid absorption	0.06
GO:0071371	cellular response to gonadotropin stimulus	0.06

Original gene set ID	Original gene set description	Nominal P value
MP:0004047	abnormal milk composition	0.06
MP:0005670	abnormal white adipose tissue physiology	0.06
ENSG00000132906	CASP9 PPI subnetwork	0.06
ENSG00000167601	AXL PPI subnetwork	0.06
ENSG00000136731	UGGT1 PPI subnetwork	0.06
GO:0015926	glucosidase activity	0.06
ENSG00000107560	RAB11FIP2 PPI subnetwork	0.06
ENSG00000179344	HLA-DQB1 PPI subnetwork	0.06
MP:0008973	decreased erythroid progenitor cell number	0.06
GO:0060350	endochondral bone morphogenesis	0.06
ENSG00000136160	EDNRB PPI subnetwork	0.06
ENSG00000070882	OSBPL3 PPI subnetwork	0.06
GO:0046942	carboxylic acid transport	0.06
ENSG00000106991	ENG PPI subnetwork	0.06
ENSG00000204673	AKT1S1 PPI subnetwork	0.06
ENSG00000145349	CAMK2D PPI subnetwork	0.06
MP:0000552	abnormal radius morphology	0.06
GO:0033692	cellular polysaccharide biosynthetic process	0.06
ENSG00000164078	MST1R PPI subnetwork	0.06
ENSG00000160271	RALGDS PPI subnetwork	0.06
GO:0031663	lipopolysaccharide-mediated signaling pathway	0.06
MP:0002444	abnormal T cell physiology	0.06
ENSG00000169714	CNBP PPI subnetwork	0.06
GO:0048185	activin binding	0.06
ENSG00000197694	SPTAN1 PPI subnetwork	0.06
MP:0004255	abnormal spongiotrophoblast layer morphology	0.06
GO:0004712	protein serine/threonine/tyrosine kinase activity	0.06
ENSG00000171094	ALK PPI subnetwork	0.06
GO:0003706	ligand-regulated transcription factor activity	0.06
ENSG00000104892	KLC3 PPI subnetwork	0.07
REACTOME_APOPTOTIC_CLEAVAGE_OF_CELL_ADHESION__PROTEINS	REACTOME_APOPTOTIC_CLEAVAGE_OF_CELL_ADHESION__PROTEINS	0.07
ENSG00000077514	POLD3 PPI subnetwork	0.07
ENSG00000116455	WDR77 PPI subnetwork	0.07
GO:0030183	B cell differentiation	0.07
MP:0000609	abnormal liver physiology	0.07
MP:0004792	abnormal synaptic vesicle number	0.07
GO:0042303	molting cycle	0.07
GO:0042633	hair cycle	0.07
MP:0000558	abnormal tibia morphology	0.07
REACTOME_ACTIVATION_OF_CHAPERONES_BY_IRE1ALPHA	REACTOME_ACTIVATION_OF_CHAPERONES_BY_IRE1ALPHA	0.07
ENSG00000185122	HSF1 PPI subnetwork	0.07
ENSG00000125686	MED1 PPI subnetwork	0.07
ENSG00000115415	STAT1 PPI subnetwork	0.07
ENSG00000158195	WASF2 PPI subnetwork	0.07
MP:0000559	abnormal femur morphology	0.07
ENSG00000136153	LMO7 PPI subnetwork	0.07
ENSG00000132780	NASP PPI subnetwork	0.07
ENSG00000174744	BRMS1 PPI subnetwork	0.07
ENSG00000198793	MTOR PPI subnetwork	0.07

Original gene set ID	Original gene set description	Nominal P value
MP:0001846	increased inflammatory response	0.07
MP:0005178	increased circulating cholesterol leve	0.07
ENSG00000165219	GAPVD1 PPI subnetwork	0.07
ENSG00000104695	PPP2CB PPI subnetwork	0.07
MP:0001539	decreased caudal vertebrae number	0.07
REACTOME_BMAL1CLOCKNPAS2_ACTIVATES_GENE_EXPRESSION	REACTOME_BMAL1CLOCKNPAS2_ACTIVATES_GENE_EXPRESSION	0.07
ENSG00000164597	COG5 PPI subnetwork	0.07
ENSG00000122679	RAMP3 PPI subnetwork	0.07
MP:0008563	decreased interferon-alpha secretion	0.07
ENSG00000203747	FCGR3A PPI subnetwork	0.07
ENSG00000129250	KIF1C PPI subnetwork	0.07
MP:0005018	decreased T cell number	0.07
REACTOME_TRAF6_MEDIATED_NF:KB_ACTIVATION	REACTOME_TRAF6_MEDIATED_NF:KB_ACTIVATION	0.07
ENSG00000144021	CIAO1 PPI subnetwork	0.07
ENSG00000184967	NOC4L PPI subnetwork	0.07
ENSG00000105245	NUMBL PPI subnetwork	0.07
MP:0002401	abnormal lymphopoiesis	0.07
REACTOME_SIGNALLING_BY_NGF	REACTOME_SIGNALLING_BY_NGF	0.07
MP:0001973	increased thermal nociceptive threshold	0.07
GO:0033077	T cell differentiation in thymus	0.07
MP:0003723	abnormal long bone morphology	0.07
ENSG00000133997	MED6 PPI subnetwork	0.07
ENSG00000007968	E2F2 PPI subnetwork	0.07
GO:0030286	dynein complex	0.07
GO:0002526	acute inflammatory response	0.07
ENSG00000182901	RGS7 PPI subnetwork	0.07
ENSG00000214485	ENSG00000214485 PPI subnetwork	0.07
GO:0035097	histone methyltransferase complex	0.07
GO:0034708	methyltransferase complex	0.07
ENSG00000171608	PIK3CD PPI subnetwork	0.07
ENSG00000149923	PPP4C PPI subnetwork	0.07
MP:0001922	reduced male fertility	0.07
GO:0005416	cation:amino acid symporter activity	0.07
MP:0001129	impaired ovarian folliculogenesis	0.07
MP:0003231	abnormal placenta vasculature	0.07
ENSG00000055332	EIF2AK2 PPI subnetwork	0.07
GO:0015849	organic acid transport	0.07
GO:0048011	nerve growth factor receptor signaling pathway	0.07
GO:0005605	basal lamina	0.07
MP:0001858	intestinal inflammation	0.07
GO:0003690	double-stranded DNA binding	0.07
ENSG00000180879	SSR4 PPI subnetwork	0.07
REACTOME_OTHER_SEMAPHORIN_INTERACTIONS	REACTOME_OTHER_SEMAPHORIN_INTERACTIONS	0.07
GO:0043408	regulation of MAPK cascade	0.07
MP:0008479	decreased spleen white pulp amount	0.07
ENSG00000185963	BICD2 PPI subnetwork	0.07
GO:0046969	NAD-dependent histone deacetylase activity (H3-K9 specific)	0.07
GO:0032129	histone deacetylase activity (H3-K9 specific)	0.07
ENSG00000105723	GSK3A PPI subnetwork	0.07

Original gene set ID	Original gene set description	Nominal P value
ENSG00000151422	FER PPI subnetwork	0.07
MP:0005031	abnormal trophoblast layer morphology	0.07
ENSG00000171150	SOCS5 PPI subnetwork	0.07
MP:0002957	intestinal adenocarcinoma	0.07
ENSG00000137831	UACA PPI subnetwork	0.07
ENSG00000102678	FGF9 PPI subnetwork	0.07
GO:0005083	small GTPase regulator activity	0.07
ENSG00000100479	POLE2 PPI subnetwork	0.07
MP:0000849	abnormal cerebellum morphology	0.07
ENSG00000072110	ACTN1 PPI subnetwork	0.07
REACTOME_CALNEXINCALRETICULIN_CYCLE	REACTOME_CALNEXINCALRETICULIN_CYCLE	0.07
ENSG00000206266	ENSG00000206266 PPI subnetwork	0.07
ENSG00000204348	DOM3Z PPI subnetwork	0.07
ENSG00000206346	DOM3Z PPI subnetwork	0.07
MP:0009417	skeletal muscle atrophy	0.07
MP:0005293	impaired glucose tolerance	0.07
MP:0000693	spleen hyperplasia	0.07
GO:0070670	response to interleukin-4	0.07
ENSG00000087266	SH3BP2 PPI subnetwork	0.08
GO:0022804	active transmembrane transporter activity	0.08
ENSG00000175866	BAIAP2 PPI subnetwork	0.08
MP:0001926	female infertility	0.08
MP:0000221	decreased leukocyte cell number	0.08
GO:0045178	basal part of cell	0.08
MP:0000352	decreased cell proliferation	0.08
ENSG00000105357	MYH14 PPI subnetwork	0.08
MP:0001239	abnormal epidermis stratum granulosum morphology	0.08
ENSG00000078061	ARAF PPI subnetwork	0.08
ENSG00000102755	FLT1 PPI subnetwork	0.08
ENSG00000145675	PIK3R1 PPI subnetwork	0.08
ENSG00000099381	SETD1A PPI subnetwork	0.08
ENSG00000018408	WWTR1 PPI subnetwork	0.08
REACTOME_P38MAPK_EVENTS	REACTOME_P38MAPK_EVENTS	0.08
MP:0005241	abnormal retinal ganglion layer morphology	0.08
ENSG00000156675	RAB11FIP1 PPI subnetwork	0.08
GO:0035591	signaling adaptor activity	0.08
GO:0045296	cadherin binding	0.08
MP:0005592	abnormal vascular smooth muscle morphology	0.08
MP:0001153	small seminiferous tubules	0.08
ENSG00000173210	ABLIM3 PPI subnetwork	0.08
ENSG00000185338	SOCS1 PPI subnetwork	0.08
MP:0000260	abnormal angiogenesis	0.08
ENSG00000127191	TRAF2 PPI subnetwork	0.08
GO:0003823	antigen binding	0.08
GO:0060348	bone development	0.08
GO:0030166	proteoglycan biosynthetic process	0.08
GO:0030522	intracellular receptor mediated signaling pathway	0.08
ENSG00000117676	RPS6KA1 PPI subnetwork	0.08
ENSG00000166888	STAT6 PPI subnetwork	0.08

Original gene set ID	Original gene set description	Nominal P value
MP:0005439	decreased glycogen level	0.08
MP:0000493	rectal prolapse	0.08
REACTOME_CIRCADIAN_CLOCK	REACTOME_CIRCADIAN_CLOCK	0.08
ENSG00000182511	FES PPI subnetwork	0.08
ENSG00000065054	SLC9A3R2 PPI subnetwork	0.08
REACTOME_TRANSCRIPTIONAL_REGULATION_OF_WHITE_ADIPOCYTE_DIFFERE	REACTOME_TRANSCRIPTIONAL_REGULATION_OF_WHITE_ADIPOCYTE_DIFFERENTI/	0.08
KEGG_DORSO_VENTRAL_AXIS_FORMATION	KEGG_DORSO_VENTRAL_AXIS_FORMATION	0.08
ENSG00000158402	CDC25C PPI subnetwork	0.08
ENSG00000107186	MPDZ PPI subnetwork	0.08
ENSG00000117395	EBNA1BP2 PPI subnetwork	0.08
GO:0032606	type I interferon production	0.08
ENSG00000101057	MYBL2 PPI subnetwork	0.08
ENSG00000163629	PTPN13 PPI subnetwork	0.08
REACTOME_METABOLISM_OF_WATER:SOLUBLE_VITAMINS_AND_COFACTORS	REACTOME_METABOLISM_OF_WATER:SOLUBLE_VITAMINS_AND_COFACTORS	0.08
REACTOME_METABOLISM_OF_VITAMINS_AND_COFACTORS	REACTOME_METABOLISM_OF_VITAMINS_AND_COFACTORS	0.08
ENSG00000133895	MEN1 PPI subnetwork	0.08
ENSG00000197451	HNRNPAB PPI subnetwork	0.08
GO:0043029	T cell homeostasis	0.08
GO:0031252	cell leading edge	0.08
GO:0009101	glycoprotein biosynthetic process	0.08
MP:0004616	lumbar vertebral transformation	0.08
MP:0003892	abnormal gastric gland morphology	0.08
MP:0002740	heart hypoplasia	0.08
MP:0000133	abnormal long bone metaphysis morphology	0.08
ENSG00000140009	ESR2 PPI subnetwork	0.08
KEGG_PANCREATIC_CANCER	KEGG_PANCREATIC_CANCER	0.08
MP:0001209	spontaneous skin ulceration	0.08
MP:0005017	decreased B cell number	0.08
ENSG00000107262	BAG1 PPI subnetwork	0.08
ENSG00000118260	CREB1 PPI subnetwork	0.08
ENSG00000169967	MAP3K2 PPI subnetwork	0.08
GO:0050840	extracellular matrix binding	0.08
ENSG00000198286	CARD11 PPI subnetwork	0.08
GO:0005085	guanyl-nucleotide exchange factor activity	0.08
ENSG00000126001	CEP250 PPI subnetwork	0.08
ENSG00000203879	GDI1 PPI subnetwork	0.08
GO:0046970	NAD-dependent histone deacetylase activity (H4-K16 specific)	0.08
GO:0034739	histone deacetylase activity (H4-K16 specific)	0.08
GO:0031078	histone deacetylase activity (H3-K14 specific)	0.08
GO:0032041	NAD-dependent histone deacetylase activity (H3-K14 specific)	0.08
REACTOME_INTERFERON_ALPHABETA_SIGNALING	REACTOME_INTERFERON_ALPHABETA_SIGNALING	0.08
MP:0000951	sporadic seizures	0.08
ENSG00000119401	TRIM32 PPI subnetwork	0.08
ENSG00000080815	PSEN1 PPI subnetwork	0.08
GO:0033674	positive regulation of kinase activity	0.08
ENSG00000102898	NUTF2 PPI subnetwork	0.08
GO:0008378	galactosyltransferase activity	0.08
MP:0000751	myopathy	0.08
ENSG00000119630	PGF PPI subnetwork	0.08

Original gene set ID	Original gene set description	Nominal P value
REACTOME_N:GLYCAN_TRIMMING_IN_THE_ER_AND_CALNEXINCALRETICULIN_	REACTOME_N:GLYCAN_TRIMMING_IN_THE_ER_AND_CALNEXINCALRETICULIN_CYC	0.08
GO:0030728	ovulation	0.08
GO:0002577	regulation of antigen processing and presentation	0.08
GO:0031100	organ regeneration	0.08
ENSG00000063046	EIF4B PPI subnetwork	0.08
REACTOME_IMMUNOREGULATORY_INTERACTIONS_BETWEEN_A_LYMPHOID_A	REACTOME_IMMUNOREGULATORY_INTERACTIONS_BETWEEN_A_LYMPHOID_AND_	0.08
MP:0001745	increased circulating corticosterone level	0.08
MP:0003087	absent allantois	0.08
ENSG00000198959	TGM2 PPI subnetwork	0.08
ENSG00000154380	ENAH PPI subnetwork	0.08
GO:0051347	positive regulation of transferase activity	0.08
ENSG00000099308	MAST3 PPI subnetwork	0.08
GO:0035250	UDP-galactosyltransferase activity	0.08
ENSG00000129682	FGF13 PPI subnetwork	0.08
MP:0008577	increased circulating interferon-gamma level	0.08
ENSG00000170579	DLGAP1 PPI subnetwork	0.08
GO:0005539	glycosaminoglycan binding	0.08
MP:0005090	increased double-negative T cell number	0.08
REACTOME_PEPTIDE_HORMONE_BIOSYNTHESIS	REACTOME_PEPTIDE_HORMONE_BIOSYNTHESIS	0.08
GO:0071900	regulation of protein serine/threonine kinase activity	0.09
GO:0045723	positive regulation of fatty acid biosynthetic process	0.09
GO:0016298	lipase activity	0.09
GO:0035770	ribonucleoprotein granule	0.09
ENSG00000085721	RRN3 PPI subnetwork	0.09
ENSG00000136250	AOAH PPI subnetwork	0.09
ENSG00000081237	PTPRC PPI subnetwork	0.09
ENSG00000143621	ILF2 PPI subnetwork	0.09
GO:0031098	stress-activated protein kinase signaling cascade	0.09
KEGG_STEROID_BIOSYNTHESIS	KEGG_STEROID_BIOSYNTHESIS	0.09
GO:0015936	coenzyme A metabolic process	0.09
ENSG00000212981	ENSG00000212981 PPI subnetwork	0.09
MP:0008750	abnormal interferon level	0.09
ENSG00000206297	TAP1 PPI subnetwork	0.09
ENSG00000206233	ENSG00000206233 PPI subnetwork	0.09
ENSG00000168394	TAP1 PPI subnetwork	0.09
GO:0031929	TOR signaling cascade	0.09
ENSG00000068305	MEF2A PPI subnetwork	0.09
ENSG00000090339	ICAM1 PPI subnetwork	0.09
ENSG00000049618	ARID1B PPI subnetwork	0.09
MP:0002665	decreased circulating corticosterone level	0.09
GO:0005796	Golgi lumen	0.09
REACTOME_INITIAL_TRIGGERING_OF_COMPLEMENT	REACTOME_INITIAL_TRIGGERING_OF_COMPLEMENT	0.09
GO:0006305	DNA alkylation	0.09
GO:0006306	DNA methylation	0.09
GO:0045061	thymic T cell selection	0.09
ENSG00000198734	F5 PPI subnetwork	0.09
ENSG00000130762	ARHGEF16 PPI subnetwork	0.09
ENSG00000166913	YWHAB PPI subnetwork	0.09
ENSG00000099917	MED15 PPI subnetwork	0.09

Original gene set ID	Original gene set description	Nominal P value
GO:0042755	eating behavior	0.09
GO:0045334	clathrin-coated endocytic vesicle	0.09
ENSG00000133104	SPG20 PPI subnetwork	0.09
GO:0008213	protein alkylation	0.09
GO:0006479	protein methylation	0.09
GO:0010740	positive regulation of intracellular protein kinase cascade	0.09
GO:0008584	male gonad development	0.09
MP:0002722	abnormal immune system organ morphology	0.09
ENSG00000120509	PDZD11 PPI subnetwork	0.09
ENSG00000204361	FAM55B PPI subnetwork	0.09
MP:0001655	multifocal hepatic necrosis	0.09
MP:0004374	bowed radius	0.09
MP:0000764	abnormal tongue epithelium morphology	0.09
ENSG00000185634	SHC4 PPI subnetwork	0.09
ENSG00000140368	PSTPIP1 PPI subnetwork	0.09
GO:0008610	lipid biosynthetic process	0.09
ENSG00000109471	IL2 PPI subnetwork	0.09
GO:0005637	nuclear inner membrane	0.09
ENSG00000169131	ZNF354A PPI subnetwork	0.09
GO:0046943	carboxylic acid transmembrane transporter activity	0.09
MP:0002086	abnormal extraembryonic tissue morphology	0.09
MP:0005325	abnormal renal glomerulus morphology	0.09
GO:0042110	T cell activation	0.09
ENSG00000107338	SHB PPI subnetwork	0.09
GO:0045860	positive regulation of protein kinase activity	0.09
GO:0002920	regulation of humoral immune response	0.09
ENSG00000106052	TAX1BP1 PPI subnetwork	0.09
ENSG00000118690	ARMC2 PPI subnetwork	0.09
ENSG00000142273	CBLC PPI subnetwork	0.09
MP:0001236	abnormal epidermis stratum spinosum morphology	0.09
GO:0009100	glycoprotein metabolic process	0.09
ENSG00000056972	TRAF3IP2 PPI subnetwork	0.09
MP:0004852	decreased testis weight	0.09
GO:0044272	sulfur compound biosynthetic process	0.09
GO:0042605	peptide antigen binding	0.09
GO:0000187	activation of MAPK activity	0.09
GO:0006029	proteoglycan metabolic process	0.09
GO:0002080	acrosomal membrane	0.09
GO:0031293	membrane protein intracellular domain proteolysis	0.09
ENSG00000110092	CCND1 PPI subnetwork	0.09
MP:0000473	abnormal stomach glandular epithelium morphology	0.09
GO:0031258	lamellipodium membrane	0.09
GO:0006694	steroid biosynthetic process	0.09
MP:0000592	short tail	0.09
GO:0010608	posttranscriptional regulation of gene expression	0.09
ENSG00000198911	SREBF2 PPI subnetwork	0.09
GO:0045446	endothelial cell differentiation	0.09
GO:0005035	death receptor activity	0.09
GO:0051346	negative regulation of hydrolase activity	0.09

Original gene set ID	Original gene set description	Nominal P value
ENSG00000136169	SETDB2 PPI subnetwork	0.09
GO:0002260	lymphocyte homeostasis	0.09
MP:0003339	decreased pancreatic beta cell number	0.09
REACTOME_COMPLEMENT_CASCADE	REACTOME_COMPLEMENT_CASCADE	0.09
MP:0001792	impaired wound healing	0.09
GO:0008406	gonad development	0.09
ENSG00000171720	HDAC3 PPI subnetwork	0.09
GO:0004620	phospholipase activity	0.09
ENSG00000101266	CSNK2A1 PPI subnetwork	0.09
ENSG00000067191	CACNB1 PPI subnetwork	0.09
KEGG_FC_GAMMA_R_MEDIATED_PHAGOCYTOSIS	KEGG_FC_GAMMA_R_MEDIATED_PHAGOCYTOSIS	0.09
REACTOME_CHYLOMICRON:MEDIATED_LIPID_TRANSPORT	REACTOME_CHYLOMICRON:MEDIATED_LIPID_TRANSPORT	0.09
MP:0002780	decreased circulating testosterone leve	0.09
MP:0001216	abnormal epidermal layer morphology	0.09
GO:0045058	T cell selection	0.09
ENSG00000215440	NPEPL1 PPI subnetwork	0.09
GO:0007565	female pregnancy	0.09
GO:0010638	positive regulation of organelle organization	0.09
MP:0001147	small testis	0.1
ENSG00000101210	EEF1A2 PPI subnetwork	0.1
MP:0005294	abnormal heart ventricle morphology	0.1
GO:0030323	respiratory tube development	0.1
GO:0005542	folic acid binding	0.1
ENSG00000141646	SMAD4 PPI subnetwork	0.1
ENSG00000100888	CHD8 PPI subnetwork	0.1
ENSG00000072062	PRKACA PPI subnetwork	0.1
ENSG00000134853	PDGFRA PPI subnetwork	0.1
ENSG00000135069	PSAT1 PPI subnetwork	0.1
GO:0000118	histone deacetylase complex	0.1
GO:0071887	leukocyte apoptotic process	0.1
MP:0008271	abnormal bone ossification	0.1
GO:0008201	heparin binding	0.1
ENSG00000124222	STX16 PPI subnetwork	0.1
GO:0030299	intestinal cholesterol absorption	0.1
ENSG00000122194	PLG PPI subnetwork	0.1
MP:0001951	abnormal breathing pattern	0.1
ENSG00000105369	CD79A PPI subnetwork	0.1
ENSG00000170889	RPS9 PPI subnetwork	0.1
ENSG00000136149	ENSG00000136149 PPI subnetwork	0.1
GO:0043410	positive regulation of MAPK cascade	0.1
MP:0000642	enlarged adrenal glands	0.1
MP:0003795	abnormal bone structure	0.1
MP:0003648	abnormal radial glial cell morphology	0.1
MP:0004151	decreased circulating iron level	0.1
REACTOME_TRANSPORT_OF_INORGANIC_CATIONSANIONS_AND_AMINO_ACID	REACTOME_TRANSPORT_OF_INORGANIC_CATIONSANIONS_AND_AMINO_ACIDSOL	0.1
ENSG00000154727	GABPA PPI subnetwork	0.1
MP:0003427	parakeratosis	0.1
GO:0031300	intrinsic to organelle membrane	0.1
MP:0002098	abnormal vibrissa morphology	0.1

Original gene set ID	Original gene set description	Nominal P value
MP:0000702	enlarged lymph nodes	0.1
ENSG00000142675	CNKSR1 PPI subnetwork	0.1
GO:0000217	DNA secondary structure binding	0.1
MP:0003453	abnormal keratinocyte physiology	0.1
GO:0006730	one-carbon metabolic process	0.1
ENSG00000099204	ABLIM1 PPI subnetwork	0.1
ENSG00000179335	CLK3 PPI subnetwork	0.1
GO:0034358	plasma lipoprotein particle	0.1
GO:0032994	protein-lipid complex	0.1
GO:0005938	cell cortex	0.1
GO:0003158	endothelium development	0.1
MP:0004974	decreased regulatory T cell number	0.1
MP:0005278	abnormal cholesterol homeostasis	0.1
GO:0030132	clathrin coat of coated pit	0.1
MP:0005167	abnormal blood-brain barrier function	0.1
ENSG00000118971	CCND2 PPI subnetwork	0.1
GO:0005024	transforming growth factor beta-activated receptor activity	0.1
MP:0000445	short snout	0.1
REACTOME_P130CAS_LINKAGE_TO_MAPK_SIGNALING_FOR_INTEGRINS	REACTOME_P130CAS_LINKAGE_TO_MAPK_SIGNALING_FOR_INTEGRINS	0.1
GO:0022405	hair cycle process	0.1
GO:0001942	hair follicle development	0.1
GO:0022404	molting cycle process	0.1
REACTOME_P75NTR_SIGNALS_VIA_NF:KB	REACTOME_P75NTR_SIGNALS_VIA_NF:KB	0.1
ENSG00000163932	PRKCD PPI subnetwork	0.1
GO:0043691	reverse cholesterol transport	0.1
ENSG00000007866	TEAD3 PPI subnetwork	0.1
GO:0070613	regulation of protein processing	0.1
ENSG00000107643	MAPK8 PPI subnetwork	0.1
MP:0001196	shiny skin	0.1
ENSG00000009954	BAZ1B PPI subnetwork	0.1
ENSG00000182953	ENSG00000182953 PPI subnetwork	0.1
REACTOME_SLC:MEDIATED_TRANSMEMBRANE_TRANSPORT	REACTOME_SLC:MEDIATED_TRANSMEMBRANE_TRANSPORT	0.1
MP:0003050	abnormal sacral vertebrae morphology	0.1
ENSG00000151617	EDNRA PPI subnetwork	0.1
GO:0009595	detection of biotic stimulus	0.1
MP:0005458	increased percent body fat	0.1
MP:0008751	abnormal interleukin level	0.1
REACTOME_INTEGRIN_ALPHAIIB_BETA3_SIGNALING	REACTOME_INTEGRIN_ALPHAIIB_BETA3_SIGNALING	0.1
ENSG00000119699	TGFB3 PPI subnetwork	0.1
ENSG00000147383	NSDHL PPI subnetwork	0.1
MP:0009674	decreased birth weight	0.1
GO:0000165	MAPK cascade	0.1
ENSG00000096060	FKBP5 PPI subnetwork	0.1
GO:0006790	sulfur compound metabolic process	0.1
ENSG00000134215	VAV3 PPI subnetwork	0.1
REACTOME_INTERLEUKIN:6_SIGNALING	REACTOME_INTERLEUKIN:6_SIGNALING	0.1
MP:0001786	skin edema	0.1
ENSG00000134333	LDHA PPI subnetwork	0.1
GO:0044264	cellular polysaccharide metabolic process	0.1

Original gene set ID	Original gene set description	Nominal P value
GO:0032607	interferon-alpha production	0.1
GO:0032647	regulation of interferon-alpha production	0.1
MP:0004358	bowed tibia	0.1
GO:0008544	epidermis development	0.1
ENSG00000101224	CDC25B PPI subnetwork	0.1
ENSG00000106144	CASP2 PPI subnetwork	0.1
MP:0001208	blistering	0.1
REACTOME_INTERLEUKIN:7_SIGNALING	REACTOME_INTERLEUKIN:7_SIGNALING	0.1
REACTOME_GAMMA:CARBOXYLATION_TRANSPORT_AND_AMINO:TERMINAL_(	REACTOME_GAMMA:CARBOXYLATION_TRANSPORT_AND_AMINO:TERMINAL_CLEA	0.1
ENSG00000107562	CXCL12 PPI subnetwork	0.1
GO:0005342	organic acid transmembrane transporter activity	0.1
GO:0005385	zinc ion transmembrane transporter activity	0.1
MP:0008883	abnormal enterocyte proliferation	0.1
GO:0048020	CCR chemokine receptor binding	0.1
ENSG00000172936	MYD88 PPI subnetwork	0.1
MP:0002375	abnormal thymus medulla morphology	0.1
GO:0003774	motor activity	0.1
GO:0071356	cellular response to tumor necrosis factor	0.11
MP:0003954	abnormal Reichert's membrane morphology	0.11
MP:0004592	small mandible	0.11
MP:0008111	abnormal granulocyte differentiation	0.11
GO:0030055	cell-substrate junction	0.11
MP:0000628	abnormal mammary gland development	0.11
ENSG00000158560	DYNC1I1 PPI subnetwork	0.11
GO:0019955	cytokine binding	0.11
GO:0034385	triglyceride-rich lipoprotein particle	0.11
GO:0034361	very-low-density lipoprotein particle	0.11
MP:0002759	abnormal caudal vertebrae morphology	0.11
GO:0055038	recycling endosome membrane	0.11
ENSG00000119139	TJP2 PPI subnetwork	0.11
REACTOME_PLATELET_DEGRANULATION	REACTOME_PLATELET_DEGRANULATION	0.11
GO:0007159	leukocyte cell-cell adhesion	0.11
ENSG00000096968	JAK2 PPI subnetwork	0.11
MP:0008050	decreased memory T cell number	0.11
GO:0006413	translational initiation	0.11
MP:0001393	ataxia	0.11
ENSG00000089009	RPL6 PPI subnetwork	0.11
REACTOME_NF:KB_IS_ACTIVATED_AND_SIGNALS_SURVIVAL	REACTOME_NF:KB_IS_ACTIVATED_AND_SIGNALS_SURVIVAL	0.11
ENSG00000089737	DDX24 PPI subnetwork	0.11
ENSG00000091831	ESR1 PPI subnetwork	0.11
GO:0034340	response to type I interferon	0.11
ENSG00000147507	ENSG00000147507 PPI subnetwork	0.11
MP:0004521	abnormal cochlear hair cell stereociliary bundle morphology	0.11
GO:0032354	response to follicle-stimulating hormone stimulus	0.11
ENSG00000108953	YWHAE PPI subnetwork	0.11
ENSG00000213923	CSNK1E PPI subnetwork	0.11
ENSG00000170017	ALCAM PPI subnetwork	0.11
GO:0016863	intramolecular oxidoreductase activity, transposing C=C bonds	0.11
ENSG00000146729	GBAS PPI subnetwork	0.11

Original gene set ID	Original gene set description	Nominal P value
MP:0002730	head shaking	0.11
GO:0008203	cholesterol metabolic process	0.11
ENSG00000171148	TADA3 PPI subnetwork	0.11
MP:0001157	small seminal vesicle	0.11
GO:0060349	bone morphogenesis	0.11
KEGG_ABC_TRANSPORTERS	KEGG_ABC_TRANSPORTERS	0.11
GO:0032479	regulation of type I interferon production	0.11
MP:0002111	abnormal tail morphology	0.11
ENSG00000197043	ANXA6 PPI subnetwork	0.11
ENSG00000172809	RPL38 PPI subnetwork	0.11
ENSG00000140575	IQGAP1 PPI subnetwork	0.11
GO:0043046	DNA methylation involved in gamete generation	0.11
ENSG00000084073	ZMPSTE24 PPI subnetwork	0.11
ENSG00000143801	PSEN2 PPI subnetwork	0.11
ENSG00000187672	ERC2 PPI subnetwork	0.11
GO:0051093	negative regulation of developmental process	0.11
ENSG00000165699	TSC1 PPI subnetwork	0.11
ENSG00000170847	ENSG00000170847 PPI subnetwork	0.11
ENSG00000173534	ENSG00000173534 PPI subnetwork	0.11
ENSG00000146950	SHROOM2 PPI subnetwork	0.11
ENSG00000157456	CCNB2 PPI subnetwork	0.11
REACTOME_REGULATION_OF_GENE_EXPRESSION_IN_BETA_CELLS	REACTOME_REGULATION_OF_GENE_EXPRESSION_IN_BETA_CELLS	0.11
GO:0071357	cellular response to type I interferon	0.11
GO:0060337	type I interferon-mediated signaling pathway	0.11
ENSG00000167657	DAPK3 PPI subnetwork	0.11
ENSG00000132693	CRP PPI subnetwork	0.11
ENSG00000005339	CREBBP PPI subnetwork	0.11
ENSG00000075945	KIFAP3 PPI subnetwork	0.11
ENSG00000120833	SOCS2 PPI subnetwork	0.11
GO:0003713	transcription coactivator activity	0.11
MP:0000218	increased leukocyte cell number	0.11
MP:0001222	epidermal hyperplasia	0.11
ENSG00000105221	AKT2 PPI subnetwork	0.11
ENSG00000167526	RPL13 PPI subnetwork	0.11
GO:0015718	monocarboxylic acid transport	0.11
ENSG00000164403	SHROOM1 PPI subnetwork	0.11
ENSG00000102804	TSC22D1 PPI subnetwork	0.11
ENSG00000074181	NOTCH3 PPI subnetwork	0.11
GO:0022626	cytosolic ribosome	0.11
ENSG00000010810	FYN PPI subnetwork	0.11
ENSG00000204592	HLA-E PPI subnetwork	0.11
REACTOME_A_THIRD_PROTEOLYTIC_CLEAVAGE_RELEASES_NICD	REACTOME_A_THIRD_PROTEOLYTIC_CLEAVAGE_RELEASES_NICD	0.11
ENSG00000143815	LBR PPI subnetwork	0.11
MP:0002497	increased IgE level	0.11
GO:0005905	coated pit	0.11
GO:0002579	positive regulation of antigen processing and presentation	0.11
ENSG00000150867	PIP4K2A PPI subnetwork	0.11
ENSG00000075426	FOSL2 PPI subnetwork	0.11
MP:0002408	abnormal double-positive T cell morphology	0.11

Original gene set ID	Original gene set description	Nominal P value
ENSG00000181827	RFX7 PPI subnetwork	0.11
ENSG00000116285	ERRFI1 PPI subnetwork	0.11
ENSG00000105193	RPS16 PPI subnetwork	0.11
ENSG00000093167	LRRFIP2 PPI subnetwork	0.11
KEGG_N_GLYCAN_BIOSYNTHESIS	KEGG_N_GLYCAN_BIOSYNTHESIS	0.11
ENSG00000113580	NR3C1 PPI subnetwork	0.11
ENSG00000058335	RASGRF1 PPI subnetwork	0.11
ENSG00000100815	TRIP11 PPI subnetwork	0.11
ENSG00000001630	CYP51A1 PPI subnetwork	0.11
ENSG00000166986	MARS PPI subnetwork	0.11
GO:0044403	symbiosis, encompassing mutualism through parasitism	0.11
MP:0008246	abnormal leukocyte morphology	0.12
MP:0003884	decreased macrophage cell number	0.12
REACTOME_DSCAM_INTERACTIONS	REACTOME_DSCAM_INTERACTIONS	0.12
ENSG00000126749	ENSG00000126749 PPI subnetwork	0.12
ENSG00000106665	CLIP2 PPI subnetwork	0.12
ENSG00000179776	CDH5 PPI subnetwork	0.12
ENSG00000142534	RPS11 PPI subnetwork	0.12
ENSG00000170677	SOCS6 PPI subnetwork	0.12
GO:0005272	sodium channel activity	0.12
MP:0008074	increased CD4-positive T cell number	0.12
ENSG00000077943	ITGA8 PPI subnetwork	0.12
GO:0016125	sterol metabolic process	0.12
GO:0071345	cellular response to cytokine stimulus	0.12
GO:0004004	ATP-dependent RNA helicase activity	0.12
MP:0005179	decreased circulating cholesterol leve	0.12
GO:0031301	integral to organelle membrane	0.12
ENSG00000010610	CD4 PPI subnetwork	0.12
ENSG00000056345	ENSG00000056345 PPI subnetwork	0.12
ENSG00000100181	ENSG00000100181 PPI subnetwork	0.12
GO:0001078	RNA polymerase II core promoter proximal region sequence-specific DNA binding tr	0.12
ENSG00000130402	ACTN4 PPI subnetwork	0.12
ENSG00000084207	GSTP1 PPI subnetwork	0.12
ENSG00000183305	MAGEA2B PPI subnetwork	0.12
ENSG00000034971	MYOC PPI subnetwork	0.12
MP:0004471	short nasal bone	0.12
MP:0003304	large intestinal inflammation	0.12
ENSG00000112078	KCTD20 PPI subnetwork	0.12
MP:0000565	oligodactyly	0.12
GO:0044419	interspecies interaction between organisms	0.12
MP:0001177	atelectasis	0.12
GO:0017136	NAD-dependent histone deacetylase activity	0.12
GO:0034979	NAD-dependent protein deacetylase activity	0.12
GO:0008585	female gonad development	0.12
ENSG00000105173	CCNE1 PPI subnetwork	0.12
ENSG00000105974	CAV1 PPI subnetwork	0.12
MP:0005296	abnormal humerus morphology	0.12
REACTOME_CHOLESTEROL_BIOSYNTHESIS	REACTOME_CHOLESTEROL_BIOSYNTHESIS	0.12
KEGG_GLYCOSPHINGOLIPID_BIOSYNTHESIS_LACTO_AND_NEOLACTO_SERIES	KEGG_GLYCOSPHINGOLIPID_BIOSYNTHESIS_LACTO_AND_NEOLACTO_SERIES	0.12

Original gene set ID	Original gene set description	Nominal P value
ENSG00000126218	F10 PPI subnetwork	0.12
MP:0005425	increased macrophage cell number	0.12
ENSG00000182010	RTKN2 PPI subnetwork	0.12
MP:0002191	abnormal artery morphology	0.12
GO:0051180	vitamin transport	0.12
MP:0000116	abnormal tooth development	0.12
GO:0002275	myeloid cell activation involved in immune response	0.12
MP:0011109	partial lethality throughout fetal growth and development	0.12
MP:0000383	abnormal hair follicle orientation	0.12
GO:0006094	gluconeogenesis	0.12
ENSG00000058404	CAMK2B PPI subnetwork	0.12
MP:0001967	deafness	0.12
MP:0008735	increased susceptibility to endotoxin shock	0.12
GO:0006957	complement activation, alternative pathway	0.12
GO:0080008	CUL4 RING ubiquitin ligase complex	0.12
GO:0019212	phosphatase inhibitor activity	0.12
ENSG00000182754	ENSG00000182754 PPI subnetwork	0.12
ENSG00000163682	RPL9 PPI subnetwork	0.12
MP:0001192	scaly skin	0.12
MP:0003809	abnormal hair shaft morphology	0.12
ENSG00000105568	PPP2R1A PPI subnetwork	0.12
GO:0006702	androgen biosynthetic process	0.12
ENSG00000177606	JUN PPI subnetwork	0.12
ENSG00000213380	COG8 PPI subnetwork	0.12
GO:0070003	threonine-type peptidase activity	0.12
GO:0004298	threonine-type endopeptidase activity	0.12
GO:0032933	SREBP-mediated signaling pathway	0.12
ENSG00000123612	ACVR1C PPI subnetwork	0.12
ENSG00000112049	ENSG00000112049 PPI subnetwork	0.12
GO:0005925	focal adhesion	0.12
ENSG00000004975	DVL2 PPI subnetwork	0.12
GO:0051017	actin filament bundle assembly	0.12
ENSG00000078043	PIAS2 PPI subnetwork	0.12
MP:0005306	abnormal phalanx morphology	0.12
ENSG00000176105	YES1 PPI subnetwork	0.12
GO:0001666	response to hypoxia	0.12
GO:0019221	cytokine-mediated signaling pathway	0.12
GO:0004553	hydrolase activity, hydrolyzing O-glycosyl compounds	0.12
ENSG00000164305	CASP3 PPI subnetwork	0.12
GO:0045022	early endosome to late endosome transport	0.12
MP:0005547	abnormal Muller cell morphology	0.12
MP:0008561	decreased tumor necrosis factor secretion	0.12
ENSG00000112033	PPARD PPI subnetwork	0.12
ENSG00000198909	MAP3K3 PPI subnetwork	0.12
MP:0008670	decreased interleukin-12b secretion	0.12
REACTOME_STEROID_HORMONES	REACTOME_STEROID_HORMONES	0.12
MP:0004810	decreased hematopoietic stem cell number	0.12
ENSG00000132382	MYBBP1A PPI subnetwork	0.12
ENSG00000134909	ARHGAP32 PPI subnetwork	0.12

Original gene set ID	Original gene set description	Nominal P value
ENSG00000197535	MYO5A PPI subnetwork	0.12
KEGG_PROGESTERONE_MEDIATED_OOCYTE_MATURATION	KEGG_PROGESTERONE_MEDIATED_OOCYTE_MATURATION	0.12
GO:0005924	cell-substrate adherens junction	0.12
MP:0002187	abnormal fibula morphology	0.12
GO:0030301	cholesterol transport	0.12
REACTOME_PEPTIDE_CHAIN_ELONGATION	REACTOME_PEPTIDE_CHAIN_ELONGATION	0.12
ENSG00000182326	C1S PPI subnetwork	0.12
ENSG00000136997	MYC PPI subnetwork	0.12
ENSG00000162302	RPS6KA4 PPI subnetwork	0.12
ENSG00000179262	RAD23A PPI subnetwork	0.12
GO:0009303	rRNA transcription	0.12
ENSG00000185507	IRF7 PPI subnetwork	0.13
GO:0008134	transcription factor binding	0.13
REACTOME_LIPID_DIGESTION_MOBILIZATION_AND_TRANSPORT	REACTOME_LIPID_DIGESTION_MOBILIZATION_AND_TRANSPORT	0.13
ENSG00000111859	NEDD9 PPI subnetwork	0.13
ENSG00000144895	EIF2A PPI subnetwork	0.13
MP:0008565	decreased interferon-beta secretion	0.13
GO:0005160	transforming growth factor beta receptor binding	0.13
ENSG00000107807	TLX1 PPI subnetwork	0.13
ENSG00000114423	CBLB PPI subnetwork	0.13
ENSG00000076641	PAG1 PPI subnetwork	0.13
ENSG00000178184	PARD6G PPI subnetwork	0.13
GO:0001944	vasculature development	0.13
ENSG00000140650	PMM2 PPI subnetwork	0.13
GO:0016202	regulation of striated muscle tissue development	0.13
MP:0002781	increased circulating testosterone leve	0.13
ENSG00000007312	CD79B PPI subnetwork	0.13
MP:0003058	increased insulin secretion	0.13
MP:0000691	enlarged spleen	0.13
GO:0005547	phosphatidylinositol-3,4,5-trisphosphate binding	0.13
ENSG00000109814	UGDH PPI subnetwork	0.13
ENSG00000155329	ZCCHC10 PPI subnetwork	0.13
GO:0008360	regulation of cell shape	0.13
ENSG00000162298	SYVN1 PPI subnetwork	0.13
ENSG00000138798	EGF PPI subnetwork	0.13
GO:0006968	cellular defense response	0.13
ENSG00000008838	MED24 PPI subnetwork	0.13
MP:0002818	abnormal dentin morphology	0.13
MP:0003091	abnormal cell migration	0.13
MP:0004921	decreased placenta weight	0.13
GO:0006695	cholesterol biosynthetic process	0.13
MP:0005621	abnormal cell physiology	0.13
KEGG_COMPLEMENT_AND_COAGULATION_CASCADES	KEGG_COMPLEMENT_AND_COAGULATION_CASCADES	0.13
GO:0022602	ovulation cycle process	0.13
MP:0008088	abnormal T-helper 1 cell differentiation	0.13
ENSG00000072135	PTPN18 PPI subnetwork	0.13
ENSG00000109475	RPL34 PPI subnetwork	0.13
GO:0010906	regulation of glucose metabolic process	0.13
ENSG00000012124	CD22 PPI subnetwork	0.13

Original gene set ID	Original gene set description	Nominal P value
MP:0003850	abnormal thymocyte activation	0.13
ENSG00000213741	RPS29 PPI subnetwork	0.13
ENSG00000198000	NOL8 PPI subnetwork	0.13
ENSG00000160087	UBE2J2 PPI subnetwork	0.13
ENSG00000125695	STRADA PPI subnetwork	0.13
ENSG00000186895	FGF3 PPI subnetwork	0.13
REACTOME_G_ALPHA_1213_SIGNALLING_EVENTS	REACTOME_G_ALPHA_1213_SIGNALLING_EVENTS	0.13
GO:0000981	sequence-specific DNA binding RNA polymerase II transcription factor activity	0.13
KEGG_ADIPOCYTOKINE_SIGNALING_PATHWAY	KEGG_ADIPOCYTOKINE_SIGNALING_PATHWAY	0.13
GO:0061039	ovum-producing ovary development	0.13
ENSG00000165733	BMS1 PPI subnetwork	0.13
MP:0005201	abnormal retinal pigment epithelium morphology	0.13
GO:0016126	sterol biosynthetic process	0.13
ENSG00000101331	C20orf160 PPI subnetwork	0.13
KEGG_INSULIN_SIGNALING_PATHWAY	KEGG_INSULIN_SIGNALING_PATHWAY	0.13
MP:0001860	liver inflammation	0.13
GO:0015291	secondary active transmembrane transporter activity	0.13
ENSG00000140464	PML PPI subnetwork	0.13
ENSG00000089157	RPLP0 PPI subnetwork	0.13
REACTOME_EUKARYOTIC_TRANSLATION_ELONGATION	REACTOME_EUKARYOTIC_TRANSLATION_ELONGATION	0.13
GO:0031091	platelet alpha granule	0.13
ENSG00000197386	HTT PPI subnetwork	0.13
ENSG00000174485	DENND4A PPI subnetwork	0.13
GO:0032330	regulation of chondrocyte differentiation	0.13
GO:0005912	adherens junction	0.13
ENSG00000168399	ENSG00000168399 PPI subnetwork	0.13
ENSG00000204273	ENSG00000204273 PPI subnetwork	0.13
ENSG00000206284	WDR46 PPI subnetwork	0.13
ENSG00000204221	WDR46 PPI subnetwork	0.13
GO:0048634	regulation of muscle organ development	0.13
ENSG00000144744	UBA3 PPI subnetwork	0.13
ENSG00000136634	IL10 PPI subnetwork	0.13
ENSG00000165702	GFI1B PPI subnetwork	0.13
MP:0008190	decreased transitional stage B cell number	0.13
ENSG00000126768	TIMM17B PPI subnetwork	0.13
ENSG00000168610	STAT3 PPI subnetwork	0.13
MP:0001805	decreased IgG level	0.13
ENSG00000133794	ARNTL PPI subnetwork	0.13
ENSG00000136021	SCYL2 PPI subnetwork	0.13
MP:0000379	decreased hair follicle number	0.13
MP:0001575	cyanosis	0.13
GO:0031984	organelle subcompartment	0.13
ENSG00000106028	SSBP1 PPI subnetwork	0.13
GO:0051119	sugar transmembrane transporter activity	0.13
GO:0016323	basolateral plasma membrane	0.13
MP:0009356	decreased liver triglyceride level	0.13
GO:0016486	peptide hormone processing	0.13
ENSG00000159216	RUNX1 PPI subnetwork	0.13
ENSG00000114686	MRPL3 PPI subnetwork	0.13

Original gene set ID	Original gene set description	Nominal P value
GO:0035194	posttranscriptional gene silencing by RNA	0.13
GO:0016441	posttranscriptional gene silencing	0.13
ENSG00000120899	PTK2B PPI subnetwork	0.13
ENSG00000106683	LIMK1 PPI subnetwork	0.13
MP:0000427	abnormal hair cycle	0.13
GO:0015399	primary active transmembrane transporter activity	0.13
GO:0015405	P-P-bond-hydrolysis-driven transmembrane transporter activity	0.13
ENSG00000136999	NOV PPI subnetwork	0.13
GO:0034623	cellular macromolecular complex disassembly	0.13
GO:0006304	DNA modification	0.13
ENSG00000112658	SRF PPI subnetwork	0.13
GO:0043414	macromolecule methylation	0.13
ENSG00000114126	TFDP2 PPI subnetwork	0.13
GO:0045103	intermediate filament-based process	0.13
KEGG_MELANOMA	KEGG_MELANOMA	0.13
MP:0005076	abnormal cell differentiation	0.13
ENSG00000168487	BMP1 PPI subnetwork	0.13
MP:0001676	abnormal apical ectodermal ridge morphology	0.13
GO:0034698	response to gonadotropin stimulus	0.13
GO:0003777	microtubule motor activity	0.13
GO:0048365	Rac GTPase binding	0.13
ENSG00000138069	RAB1A PPI subnetwork	0.13
MP:0001240	abnormal epidermis stratum corneum morphology	0.13
MP:0002060	abnormal skin morphology	0.14
ENSG00000141434	MEP1B PPI subnetwork	0.14
MP:0011099	complete lethality throughout fetal growth and development	0.14
ENSG00000198755	RPL10A PPI subnetwork	0.14
ENSG00000135424	ITGA7 PPI subnetwork	0.14
ENSG00000197303	ENSG00000197303 PPI subnetwork	0.14
GO:0050764	regulation of phagocytosis	0.14
MP:0001847	brain inflammation	0.14
ENSG00000080839	RBL1 PPI subnetwork	0.14
MP:0002404	increased intestinal adenoma incidence	0.14
MP:0008470	abnormal spleen B cell follicle morphology	0.14
GO:0019218	regulation of steroid metabolic process	0.14
GO:0003724	RNA helicase activity	0.14
MP:0003436	decreased susceptibility to induced arthritis	0.14
MP:0009009	absent estrous cycle	0.14
ENSG00000135144	DTX1 PPI subnetwork	0.14
ENSG00000185432	METTL7A PPI subnetwork	0.14
GO:0015278	calcium-release channel activity	0.14
GO:0005604	basement membrane	0.14
ENSG00000117242	ENSG00000117242 PPI subnetwork	0.14
ENSG00000099399	MAGEB2 PPI subnetwork	0.14
GO:0014909	smooth muscle cell migration	0.14
ENSG00000185386	MAPK11 PPI subnetwork	0.14
ENSG00000150527	CTAGE5 PPI subnetwork	0.14
GO:0043624	cellular protein complex disassembly	0.14
GO:0051607	defense response to virus	0.14

Original gene set ID	Original gene set description	Nominal P value
GO:0007276	gamete generation	0.14
ENSG00000163519	TRAT1 PPI subnetwork	0.14
ENSG00000174748	RPL15 PPI subnetwork	0.14
MP:0006317	decreased urine sodium level	0.14
GO:0045104	intermediate filament cytoskeleton organization	0.14
MP:0001284	absent vibrissae	0.14
ENSG00000185499	MUC1 PPI subnetwork	0.14
MP:0008127	decreased dendritic cell number	0.14
ENSG00000134242	PTPN22 PPI subnetwork	0.14
GO:0005044	scavenger receptor activity	0.14
ENSG00000087245	MMP2 PPI subnetwork	0.14
MP:0002727	decreased circulating insulin level	0.14
GO:0015012	heparan sulfate proteoglycan biosynthetic process	0.14
GO:0031965	nuclear membrane	0.14
GO:0005792	microsome	0.14
ENSG00000161835	GRASP PPI subnetwork	0.14
MP:0005089	decreased double-negative T cell number	0.14
REACTOME_CD28_DEPENDENT_PI3KAKT_SIGNALING	REACTOME_CD28_DEPENDENT_PI3KAKT_SIGNALING	0.14
GO:0051604	protein maturation	0.14
GO:0050691	regulation of defense response to virus by host	0.14
MP:0010465	aberrant origin of the right subclavian artery	0.14
REACTOME_REGULATION_OF_LIPID_METABOLISM_BY_PEROXISOME_PROLIFER	REACTOME_REGULATION_OF_LIPID_METABOLISM_BY_PEROXISOME_PROLIFERATO	0.14
REACTOME_P75NTR_RECRUITS_SIGNALLING_COMPLEXES	REACTOME_P75NTR_RECRUITS_SIGNALLING_COMPLEXES	0.14
REACTOME_NUCLEAR_RECEPTOR_TRANSCRIPTION_PATHWAY	REACTOME_NUCLEAR_RECEPTOR_TRANSCRIPTION_PATHWAY	0.14
ENSG00000139318	DUSP6 PPI subnetwork	0.14
MP:0003396	abnormal embryonic hematopoiesis	0.14
GO:0007346	regulation of mitotic cell cycle	0.14
GO:0045596	negative regulation of cell differentiation	0.14
GO:0007584	response to nutrient	0.14
GO:0048194	Golgi vesicle budding	0.14
REACTOME_METABOLISM_OF_PROTEINS	REACTOME_METABOLISM_OF_PROTEINS	0.14
MP:0003156	abnormal leukocyte migration	0.14
GO:0050654	chondroitin sulfate proteoglycan metabolic process	0.14
ENSG00000177731	FLII PPI subnetwork	0.14
GO:0071339	MLL1 complex	0.14
MP:0000598	abnormal liver morphology	0.14
GO:0002673	regulation of acute inflammatory response	0.14
ENSG00000130956	HABP4 PPI subnetwork	0.14
ENSG00000171490	RSL1D1 PPI subnetwork	0.14
ENSG00000169896	ITGAM PPI subnetwork	0.14
REACTOME_RESPONSE_TO_ELEVATED_PLATELET_CYTOSOLIC_CA2	REACTOME_RESPONSE_TO_ELEVATED_PLATELET_CYTOSOLIC_CA2	0.14
GO:0014911	positive regulation of smooth muscle cell migration	0.14
MP:0001953	respiratory failure	0.14
ENSG00000169398	PTK2 PPI subnetwork	0.14
ENSG00000182636	NDN PPI subnetwork	0.14
MP:0008209	decreased pre-B cell number	0.14
REACTOME_POST:TRANSLATIONAL_PROTEIN_MODIFICATION	REACTOME_POST:TRANSLATIONAL_PROTEIN_MODIFICATION	0.14
MP:0002113	abnormal skeleton development	0.14
ENSG00000171862	PTEN PPI subnetwork	0.14

Original gene set ID	Original gene set description	Nominal P value
GO:0008158	hedgehog receptor activity	0.14
GO:0006414	translational elongation	0.14
GO:0042598	vesicular fraction	0.14
ENSG00000213949	ITGA1 PPI subnetwork	0.14
GO:0042446	hormone biosynthetic process	0.14
GO:0008186	RNA-dependent ATPase activity	0.14
ENSG00000155506	LARP1 PPI subnetwork	0.14
ENSG00000127947	PTPN12 PPI subnetwork	0.14
ENSG00000166333	ILK PPI subnetwork	0.14
REACTOME_FACILITATIVE_NA:INDEPENDENT_GLUCOSE_TRANSPORTERS	REACTOME_FACILITATIVE_NA:INDEPENDENT_GLUCOSE_TRANSPORTERS	0.14
GO:0002831	regulation of response to biotic stimulus	0.14
GO:0009103	lipopolysaccharide biosynthetic process	0.14
ENSG00000158092	NCK1 PPI subnetwork	0.14
GO:0030324	lung development	0.14
GO:0070001	aspartic-type peptidase activity	0.14
GO:0004190	aspartic-type endopeptidase activity	0.14
REACTOME_METABOLISM_OF_LIPIDS_AND_LIPOPROTEINS	REACTOME_METABOLISM_OF_LIPIDS_AND_LIPOPROTEINS	0.14
ENSG00000143537	ADAM15 PPI subnetwork	0.15
ENSG00000052802	MSMO1 PPI subnetwork	0.15
GO:0002437	inflammatory response to antigenic stimulus	0.15
ENSG00000143398	PIP5K1A PPI subnetwork	0.15
GO:0019902	phosphatase binding	0.15
ENSG00000105329	TGFB1 PPI subnetwork	0.15
GO:0006909	phagocytosis	0.15
ENSG00000184557	SOCS3 PPI subnetwork	0.15
ENSG00000073614	KDM5A PPI subnetwork	0.15
ENSG00000212664	ENSG00000212664 PPI subnetwork	0.15
MP:0002216	abnormal seminiferous tubule morphology	0.15
ENSG00000120235	IFNA6 PPI subnetwork	0.15
ENSG00000134318	ROCK2 PPI subnetwork	0.15
GO:0001816	cytokine production	0.15
ENSG00000083642	PDS5B PPI subnetwork	0.15
MP:0008037	abnormal T cell morphology	0.15
ENSG00000102189	EEA1 PPI subnetwork	0.15
ENSG00000175189	INHBC PPI subnetwork	0.15
ENSG00000139239	ENSG00000139239 PPI subnetwork	0.15
GO:0015179	L-amino acid transmembrane transporter activity	0.15
GO:0003730	mRNA 3'-UTR binding	0.15
ENSG00000118046	STK11 PPI subnetwork	0.15
GO:0003707	steroid hormone receptor activity	0.15
GO:0001958	endochondral ossification	0.15
GO:0006090	pyruvate metabolic process	0.15
REACTOME_REGULATION_OF_BETA:CELL_DEVELOPMENT	REACTOME_REGULATION_OF_BETA:CELL_DEVELOPMENT	0.15
ENSG00000138794	CASP6 PPI subnetwork	0.15
ENSG00000104973	MED25 PPI subnetwork	0.15
ENSG00000180008	SOCS4 PPI subnetwork	0.15
ENSG00000087303	NID2 PPI subnetwork	0.15
MP:0003911	increased drinking behavior	0.15
MP:0000240	extramedullary hematopoiesis	0.15

Original gene set ID	Original gene set description	Nominal P value
ENSG00000206088	ENSG00000206088 PPI subnetwork	0.15
MP:0008215	decreased immature B cell number	0.15
ENSG00000154262	ABCA6 PPI subnetwork	0.15
GO:0070161	anchoring junction	0.15
GO:0030203	glycosaminoglycan metabolic process	0.15
ENSG00000029725	RABEP1 PPI subnetwork	0.15
GO:0016458	gene silencing	0.15
ENSG00000134987	WDR36 PPI subnetwork	0.15
ENSG00000118058	MLL PPI subnetwork	0.15
MP:0000812	abnormal dentate gyrus morphology	0.15
GO:0000922	spindle pole	0.15
ENSG00000147604	RPL7 PPI subnetwork	0.15
MP:0002864	abnormal ocular fundus morphology	0.15
ENSG00000136488	CSH1 PPI subnetwork	0.15
ENSG00000082805	ERC1 PPI subnetwork	0.15
GO:0050868	negative regulation of T cell activation	0.15
GO:0019900	kinase binding	0.15
ENSG00000180182	MED14 PPI subnetwork	0.15
ENSG00000115053	NCL PPI subnetwork	0.15
ENSG00000137500	CCDC90B PPI subnetwork	0.15
ENSG00000141510	TP53 PPI subnetwork	0.15
ENSG00000172766	NAA16 PPI subnetwork	0.15
ENSG00000111676	ATN1 PPI subnetwork	0.15
REACTOME_ABC:FAMILY_PROTEINS_MEDIATED_TRANSPORT	REACTOME_ABC:FAMILY_PROTEINS_MEDIATED_TRANSPORT	0.15
ENSG00000047936	ROS1 PPI subnetwork	0.15
GO:0032984	macromolecular complex disassembly	0.15
GO:0006085	acetyl-CoA biosynthetic process	0.15
GO:0003682	chromatin binding	0.15
ENSG00000196363	WDR5 PPI subnetwork	0.15
ENSG00000173402	DAG1 PPI subnetwork	0.15
MP:0000160	kyphosis	0.15
GO:0019838	growth factor binding	0.15
MP:0008475	intermingled spleen red and white pulp	0.15
MP:0003887	increased hepatocyte apoptosis	0.15
ENSG00000136244	IL6 PPI subnetwork	0.15
GO:0042826	histone deacetylase binding	0.15
ENSG00000211592	ENSG00000211592 PPI subnetwork	0.15
GO:0033157	regulation of intracellular protein transport	0.15
ENSG00000171208	NETO2 PPI subnetwork	0.15
ENSG00000115232	ITGA4 PPI subnetwork	0.15
ENSG00000163346	PBXIP1 PPI subnetwork	0.15
ENSG00000104549	SQLE PPI subnetwork	0.15
ENSG00000117222	RBBP5 PPI subnetwork	0.15
MP:0009238	coiled sperm flagellum	0.15
GO:0006024	glycosaminoglycan biosynthetic process	0.15
ENSG00000137757	CASP5 PPI subnetwork	0.15
ENSG00000185591	SP1 PPI subnetwork	0.15
ENSG00000108504	ENSG00000108504 PPI subnetwork	0.15
MP:0003055	abnormal long bone epiphyseal plate morphology	0.15

Original gene set ID	Original gene set description	Nominal P value
GO:0003705	RNA polymerase II distal enhancer sequence-specific DNA binding transcription fact	0.15
GO:0001570	vasculogenesis	0.15
MP:0002731	megacolon	0.15
ENSG00000147403	RPL10 PPI subnetwork	0.15
ENSG00000175592	FOSL1 PPI subnetwork	0.15
ENSG00000174697	LEP PPI subnetwork	0.15
MP:0001919	abnormal reproductive system physiology	0.15
ENSG00000136574	GATA4 PPI subnetwork	0.15
ENSG00000162891	IL20 PPI subnetwork	0.15
MP:0000321	increased bone marrow cell number	0.15
GO:0046966	thyroid hormone receptor binding	0.15
GO:0019319	hexose biosynthetic process	0.15
GO:0032580	Golgi cisterna membrane	0.15
MP:0003638	abnormal response/metabolism to endogenous compounds	0.15
GO:0030947	regulation of vascular endothelial growth factor receptor signaling pathway	0.15
ENSG00000120948	TARDBP PPI subnetwork	0.15
ENSG00000166285	ENSG00000166285 PPI subnetwork	0.15
ENSG00000204359	CFB PPI subnetwork	0.15
GO:0008653	lipopolysaccharide metabolic process	0.15
GO:0042803	protein homodimerization activity	0.15
ENSG00000037241	RPL26L1 PPI subnetwork	0.15
GO:0048514	blood vessel morphogenesis	0.15
MP:0000416	sparse hair	0.15
ENSG00000128487	SPECC1 PPI subnetwork	0.15
REACTOME_GTP_HYDROLYSIS_AND_JOINING_OF_THE_60S_RIBOSOMAL_SUBU	REACTOME_GTP_HYDROLYSIS_AND_JOINING_OF_THE_60S_RIBOSOMAL_SUBUNIT	0.15
ENSG00000189403	HMGB1 PPI subnetwork	0.15
ENSG00000148297	MED22 PPI subnetwork	0.15
GO:0006916	anti-apoptosis	0.15
ENSG00000117408	IPO13 PPI subnetwork	0.15
ENSG00000156273	BACH1 PPI subnetwork	0.16
MP:0000454	abnormal jaw morphology	0.16
ENSG00000213625	LEPROT PPI subnetwork	0.16
GO:0005313	L-glutamate transmembrane transporter activity	0.16
GO:0050650	chondroitin sulfate proteoglycan biosynthetic process	0.16
GO:0005179	hormone activity	0.16
MP:0002493	increased IgG level	0.16
GO:0004806	triglyceride lipase activity	0.16
GO:0015103	inorganic anion transmembrane transporter activity	0.16
GO:0070482	response to oxygen levels	0.16
ENSG00000196700	ZNF512B PPI subnetwork	0.16
ENSG00000134588	USP26 PPI subnetwork	0.16
GO:0001568	blood vessel development	0.16
GO:0016585	chromatin remodeling complex	0.16
REACTOME_RHO_GTPASE_CYCLE	REACTOME_RHO_GTPASE_CYCLE	0.16
REACTOME_SIGNALING_BY_RHO_GTPASES	REACTOME_SIGNALING_BY_RHO_GTPASES	0.16
GO:0043449	cellular alkene metabolic process	0.16
GO:0006691	leukotriene metabolic process	0.16
GO:0030098	lymphocyte differentiation	0.16
ENSG00000072195	SPEG PPI subnetwork	0.16

Original gene set ID	Original gene set description	Nominal P value
ENSG00000160791	CCR5 PPI subnetwork	0.16
ENSG00000215778	ENSG00000215778 PPI subnetwork	0.16
MP:0008719	impaired neutrophil recruitment	0.16
MP:0004016	decreased bone mass	0.16
MP:0002192	hydrops fetalis	0.16
MP:0002356	abnormal spleen red pulp morphology	0.16
GO:0031060	regulation of histone methylation	0.16
ENSG00000149084	HSD17B12 PPI subnetwork	0.16
ENSG00000086061	DNAJA1 PPI subnetwork	0.16
GO:0008202	steroid metabolic process	0.16
ENSG00000144867	SRPRB PPI subnetwork	0.16
ENSG00000198780	FAM169A PPI subnetwork	0.16
MP:0009858	abnormal cellular extravasation	0.16
GO:0000323	lytic vacuole	0.16
GO:0005764	lysosome	0.16
ENSG00000140481	CCDC33 PPI subnetwork	0.16
ENSG00000129048	CCRL1 PPI subnetwork	0.16
ENSG00000212695	ENSG00000212695 PPI subnetwork	0.16
GO:0032924	activin receptor signaling pathway	0.16
REACTOME_N:GLYCAN_ANTENNAE_ELONGATION	REACTOME_N:GLYCAN_ANTENNAE_ELONGATION	0.16
MP:0005006	abnormal osteoblast physiology	0.16
ENSG00000105397	TYK2 PPI subnetwork	0.16
ENSG00000077942	FBLN1 PPI subnetwork	0.16
GO:0015923	mannosidase activity	0.16
MP:0008254	increased megakaryocyte cell number	0.16
GO:0032006	regulation of TOR signaling cascade	0.16
ENSG00000144648	CCBP2 PPI subnetwork	0.16
GO:0003729	mRNA binding	0.16
MP:0002754	dilated heart right ventricle	0.16
MP:0002833	increased heart weight	0.16
GO:0042383	sarcolemma	0.16
ENSG00000138376	BARD1 PPI subnetwork	0.16
MP:0008553	increased circulating tumor necrosis factor leve	0.16
ENSG00000188687	SLC4A5 PPI subnetwork	0.16
KEGG_PRIMARY_IMMUNODEFICIENCY	KEGG_PRIMARY_IMMUNODEFICIENCY	0.16
ENSG00000138107	ACTR1A PPI subnetwork	0.16
MP:0010027	increased liver cholesterol level	0.16
GO:0048487	beta-tubulin binding	0.16
ENSG00000134419	RPS15A PPI subnetwork	0.16
GO:0043450	alkene biosynthetic process	0.16
GO:0019370	leukotriene biosynthetic process	0.16
GO:0033619	membrane protein proteolysis	0.16
GO:0043241	protein complex disassembly	0.16
GO:0032728	positive regulation of interferon-beta productior	0.16
ENSG00000111679	PTPN6 PPI subnetwork	0.16
MP:0000600	liver hypoplasia	0.16
ENSG00000171791	BCL2 PPI subnetwork	0.16
ENSG00000146677	ENSG00000146677 PPI subnetwork	0.16
GO:0050863	regulation of T cell activation	0.16

Original gene set ID	Original gene set description	Nominal P value
GO:0016814	hydrolase activity, acting on carbon-nitrogen (but not peptide) bonds, in cyclic amid	0.16
ENSG00000104365	IKBKB PPI subnetwork	0.16
MP:0004905	decreased uterus weight	0.16
GO:0005790	smooth endoplasmic reticulum	0.16
GO:0008028	monocarboxylic acid transmembrane transporter activity	0.16
ENSG00000170871	KIAA0232 PPI subnetwork	0.16
ENSG00000206340	C4A PPI subnetwork	0.16
ENSG00000172172	MRPL13 PPI subnetwork	0.16
MP:0002932	abnormal joint morphology	0.16
GO:0015909	long-chain fatty acid transport	0.16
GO:0060537	muscle tissue development	0.16
MP:0008075	decreased CD4-positive T cell number	0.16
ENSG00000147955	SIGMAR1 PPI subnetwork	0.16
GO:0006023	aminoglycan biosynthetic process	0.16
GO:0034405	response to fluid shear stress	0.16
MP:0001874	acanthosis	0.16
ENSG00000206413	ENSG00000206413 PPI subnetwork	0.16
ENSG00000206493	HLA-E PPI subnetwork	0.16
MP:0011320	abnormal glomerular capillary morphology	0.16
MP:0001156	abnormal spermatogenesis	0.16
GO:0019198	transmembrane receptor protein phosphatase activity	0.16
GO:0005001	transmembrane receptor protein tyrosine phosphatase activity	0.16
GO:0001726	ruffle	0.16
GO:0031047	gene silencing by RNA	0.16
MP:0005232	abnormal mesenteric lymph node morphology	0.16
ENSG00000071537	SEL1L PPI subnetwork	0.16
ENSG00000189037	DUSP21 PPI subnetwork	0.16
GO:0033209	tumor necrosis factor-mediated signaling pathway	0.16
GO:0030658	transport vesicle membrane	0.16
ENSG00000065989	PDE4A PPI subnetwork	0.16
ENSG00000175333	ENSG00000175333 PPI subnetwork	0.16
ENSG00000177283	FZD8 PPI subnetwork	0.16
GO:0035725	sodium ion transmembrane transport	0.16
MP:0001732	postnatal growth retardation	0.16
GO:0018195	peptidyl-arginine modification	0.16
GO:0001893	maternal placenta development	0.16
REACTOME_PPARA_ACTIVATES_GENE_EXPRESSION	REACTOME_PPARA_ACTIVATES_GENE_EXPRESSION	0.16
ENSG00000127329	PTPRB PPI subnetwork	0.16
MP:0011143	thick lung-associated mesenchyme	0.16
ENSG00000121989	ACVR2A PPI subnetwork	0.16
MP:0005015	increased T cell number	0.16
GO:0001501	skeletal system development	0.16
ENSG00000111241	FGF6 PPI subnetwork	0.16
ENSG00000107831	FGF8 PPI subnetwork	0.16
ENSG00000158815	FGF17 PPI subnetwork	0.16
ENSG00000162344	FGF19 PPI subnetwork	0.16
ENSG00000156427	FGF18 PPI subnetwork	0.16
ENSG00000070388	FGF22 PPI subnetwork	0.16
ENSG00000183337	BCOR PPI subnetwork	0.17

Original gene set ID	Original gene set description	Nominal P value
GO:0008654	phospholipid biosynthetic process	0.17
ENSG00000115268	RPS15 PPI subnetwork	0.17
GO:0010469	regulation of receptor activity	0.17
GO:0042632	cholesterol homeostasis	0.17
GO:0055092	sterol homeostasis	0.17
ENSG00000163714	U2SURP PPI subnetwork	0.17
ENSG00000132664	POLR3F PPI subnetwork	0.17
ENSG00000186660	ZFP91 PPI subnetwork	0.17
GO:0035313	wound healing, spreading of epidermal cells	0.17
GO:0034706	sodium channel complex	0.17
MP:0000377	abnormal hair follicle morphology	0.17
GO:0005874	microtubule	0.17
GO:0031345	negative regulation of cell projection organizatior	0.17
REACTOME_LIPOPROTEIN_METABOLISM	REACTOME_LIPOPROTEIN_METABOLISM	0.17
ENSG00000052749	RRP12 PPI subnetwork	0.17
MP:0000121	failure of tooth eruption	0.17
GO:0001829	trophectodermal cell differentiation	0.17
ENSG00000145777	TSLP PPI subnetwork	0.17
GO:0016765	transferase activity, transferring alkyl or aryl (other than methyl) groups	0.17
MP:0000947	convulsive seizures	0.17
GO:0006656	phosphatidylcholine biosynthetic process	0.17
ENSG00000164754	RAD21 PPI subnetwork	0.17
REACTOME_NRIF_SIGNALS_CELL_DEATH_FROM_THE_NUCLEUS	REACTOME_NRIF_SIGNALS_CELL_DEATH_FROM_THE_NUCLEUS	0.17
ENSG00000122861	PLAU PPI subnetwork	0.17
MP:0002177	abnormal outer ear morphology	0.17
KEGG_GLYCOSAMINOGLYCAN_DEGRADATION	KEGG_GLYCOSAMINOGLYCAN_DEGRADATION	0.17
GO:0005913	cell-cell adherens junction	0.17
ENSG00000160801	PTH1R PPI subnetwork	0.17
GO:0015918	sterol transport	0.17
ENSG00000095752	IL11 PPI subnetwork	0.17
REACTOME_GLUTAMATE_NEUROTRANSMITTER_RELEASE_CYCLE	REACTOME_GLUTAMATE_NEUROTRANSMITTER_RELEASE_CYCLE	0.17
ENSG00000171401	KRT13 PPI subnetwork	0.17
ENSG00000102882	MAPK3 PPI subnetwork	0.17
GO:0007160	cell-matrix adhesion	0.17
GO:0016798	hydrolase activity, acting on glycosyl bonds	0.17
GO:0043299	leukocyte degranulation	0.17
MP:0000130	abnormal trabecular bone morphology	0.17
GO:0004879	ligand-activated sequence-specific DNA binding RNA polymerase II transcription fact	0.17
ENSG00000100823	APEX1 PPI subnetwork	0.17
MP:0003409	decreased width of hypertrophic chondrocyte zone	0.17
ENSG00000162244	RPL29 PPI subnetwork	0.17
MP:0001200	thick skin	0.17
ENSG00000065361	ERBB3 PPI subnetwork	0.17
GO:0035195	gene silencing by miRNA	0.17
ENSG00000211896	ENSG00000211896 PPI subnetwork	0.17
ENSG00000148843	PDCD11 PPI subnetwork	0.17
MP:0001750	increased circulating follicle stimulating hormone leve	0.17
MP:0008097	increased plasma cell number	0.17
ENSG00000136715	SAP130 PPI subnetwork	0.17

Original gene set ID	Original gene set description	Nominal P value
GO:0043900	regulation of multi-organism process	0.17
GO:0033865	nucleoside bisphosphate metabolic process	0.17
ENSG00000007816	ENSG00000007816 PPI subnetwork	0.17
GO:0043500	muscle adaptation	0.17
ENSG00000086598	TMED2 PPI subnetwork	0.17
GO:0044241	lipid digestion	0.17
ENSG00000137285	TUBB2B PPI subnetwork	0.17
MP:0001191	abnormal skin condition	0.17
MP:0000367	abnormal coat/ hair morphology	0.17
ENSG00000120156	TEK PPI subnetwork	0.17
GO:0016529	sarcoplasmic reticulum	0.17
GO:0046661	male sex differentiation	0.17
GO:0046527	glucosyltransferase activity	0.17
MP:0008873	increased physiological sensitivity to xenobiotic	0.17
ENSG00000005884	ITGA3 PPI subnetwork	0.17
GO:0033273	response to vitamin	0.17
ENSG00000197045	GMFB PPI subnetwork	0.17
ENSG00000071539	TRIP13 PPI subnetwork	0.17
GO:0032012	regulation of ARF protein signal transduction	0.17
REACTOME_GLYCOSPHINGOLIPID_METABOLISM	REACTOME_GLYCOSPHINGOLIPID_METABOLISM	0.17
MP:0006354	abnormal fourth branchial arch artery morphology	0.17
KEGG_GALACTOSE_METABOLISM	KEGG_GALACTOSE_METABOLISM	0.17
ENSG00000087269	NOP14 PPI subnetwork	0.17
ENSG00000123416	TUBA1B PPI subnetwork	0.17
GO:0071706	tumor necrosis factor superfamily cytokine production	0.17
GO:0005903	brush border	0.17
GO:0006641	triglyceride metabolic process	0.17
MP:0008208	decreased pro-B cell number	0.17
ENSG00000166598	HSP90B1 PPI subnetwork	0.17
ENSG00000118705	RPN2 PPI subnetwork	0.17
ENSG00000129214	SHBG PPI subnetwork	0.17
GO:0046889	positive regulation of lipid biosynthetic process	0.17
GO:0019888	protein phosphatase regulator activity	0.17
ENSG00000108264	TADA2A PPI subnetwork	0.17
ENSG00000132196	HSD17B7 PPI subnetwork	0.17
MP:0008280	male germ cell apoptosis	0.17
GO:0051250	negative regulation of lymphocyte activation	0.17
GO:0008299	isoprenoid biosynthetic process	0.17
ENSG00000055957	ITIH1 PPI subnetwork	0.17
GO:0046128	purine ribonucleoside metabolic process	0.17
ENSG00000002330	BAD PPI subnetwork	0.17
GO:0016570	histone modification	0.17
ENSG00000141522	ARHGDIA PPI subnetwork	0.17
MP:0000358	abnormal cell morphology	0.17
ENSG00000107242	PIP5K1B PPI subnetwork	0.17
GO:0019748	secondary metabolic process	0.17
ENSG00000167244	IGF2 PPI subnetwork	0.17
GO:0000139	Golgi membrane	0.17
ENSG00000138675	FGF5 PPI subnetwork	0.17

Original gene set ID	Original gene set description	Nominal P value
REACTOME_REGULATION_OF_PYRUVATE_DEHYDROGENASE_PDH_COMPLEX	REACTOME_REGULATION_OF_PYRUVATE_DEHYDROGENASE_PDH_COMPLEX	0.17
ENSG00000169136	ATF5 PPI subnetwork	0.17
MP:0006397	disorganized long bone epiphyseal plate	0.17
GO:0046546	development of primary male sexual characteristics	0.17
ENSG00000127318	IL22 PPI subnetwork	0.17
MP:0003998	decreased thermal nociceptive threshold	0.17
MP:0008782	increased B cell apoptosis	0.17
ENSG00000033327	GAB2 PPI subnetwork	0.17
GO:0015172	acidic amino acid transmembrane transporter activity	0.17
ENSG00000110492	MDK PPI subnetwork	0.17
GO:0010741	negative regulation of intracellular protein kinase cascade	0.17
GO:0005251	delayed rectifier potassium channel activity	0.17
GO:0042158	lipoprotein biosynthetic process	0.17
MP:0003140	dilated heart atrium	0.17
ENSG00000012660	ELOVL5 PPI subnetwork	0.17
REACTOME_HEMOSTASIS	REACTOME_HEMOSTASIS	0.17
ENSG00000127511	SIN3B PPI subnetwork	0.17
ENSG00000150281	CTF1 PPI subnetwork	0.17
ENSG00000125998	FAM83C PPI subnetwork	0.17
MP:0001190	reddish skin	0.17
GO:0043687	post-translational protein modification	0.17
GO:0006415	translational termination	0.18
GO:0032312	regulation of ARF GTPase activity	0.18
GO:0030198	extracellular matrix organization	0.18
GO:0043062	extracellular structure organization	0.18
GO:0046777	protein autophosphorylation	0.18
ENSG00000175221	MED16 PPI subnetwork	0.18
ENSG00000167721	TSR1 PPI subnetwork	0.18
GO:0019210	kinase inhibitor activity	0.18
GO:0022625	cytosolic large ribosomal subunit	0.18
MP:0001925	male infertility	0.18
ENSG00000129219	PLD2 PPI subnetwork	0.18
ENSG00000058729	RIOK2 PPI subnetwork	0.18
GO:0015450	P-P-bond-hydrolysis-driven protein transmembrane transporter activity	0.18
ENSG00000103742	IGDCC4 PPI subnetwork	0.18
MP:0002447	abnormal erythrocyte morphology	0.18
REACTOME_CELL_JUNCTION_ORGANIZATION	REACTOME_CELL_JUNCTION_ORGANIZATION	0.18
ENSG00000161970	RPL26 PPI subnetwork	0.18
ENSG00000147873	IFNA5 PPI subnetwork	0.18
ENSG00000186803	IFNA10 PPI subnetwork	0.18
ENSG00000120247	ENSG00000120247 PPI subnetwork	0.18
ENSG00000147877	ENSG00000147877 PPI subnetwork	0.18
ENSG00000188379	IFNA2 PPI subnetwork	0.18
ENSG00000186809	ENSG00000186809 PPI subnetwork	0.18
ENSG00000147885	IFNA16 PPI subnetwork	0.18
ENSG00000137080	IFNA21 PPI subnetwork	0.18
ENSG00000120242	IFNA8 PPI subnetwork	0.18
GO:0043967	histone H4 acetylation	0.18
MP:0002904	increased circulating parathyroid hormone leve	0.18

Original gene set ID	Original gene set description	Nominal P value
GO:0046697	decidualization	0.18
MP:0008115	abnormal dendritic cell differentiation	0.18
ENSG00000086189	DIMT1 PPI subnetwork	0.18
ENSG00000113889	KNG1 PPI subnetwork	0.18
MP:0005146	decreased circulating VLDL cholesterol leve	0.18
MP:0005136	decreased growth hormone level	0.18
ENSG00000188130	MAPK12 PPI subnetwork	0.18
GO:0016779	nucleotidyltransferase activity	0.18
GO:0010907	positive regulation of glucose metabolic process	0.18
ENSG00000100842	EFS PPI subnetwork	0.18
REACTOME_METAL_ION_SLC_TRANSPORTERS	REACTOME_METAL_ION_SLC_TRANSPORTERS	0.18
GO:0006829	zinc ion transport	0.18
GO:0090263	positive regulation of canonical Wnt receptor signaling pathway	0.18
KEGG_TERPENOID_BACKBONE_BIOSYNTHESIS	KEGG_TERPENOID_BACKBONE_BIOSYNTHESIS	0.18
MP:0000187	abnormal triglyceride level	0.18
GO:0030246	carbohydrate binding	0.18
GO:0017048	Rho GTPase binding	0.18
MP:0000259	abnormal vascular development	0.18
MP:0008844	decreased subcutaneous adipose tissue amount	0.18
KEGG_BASAL_CELL_CARCINOMA	KEGG_BASAL_CELL_CARCINOMA	0.18
GO:0050839	cell adhesion molecule binding	0.18
GO:0015145	monosaccharide transmembrane transporter activity	0.18
GO:0004181	metallocarboxypeptidase activity	0.18
ENSG00000155957	TMBIM4 PPI subnetwork	0.18
GO:0006412	translation	0.18
REACTOME_GPVI:MEDIATED_ACTIVATION_CASCADE	REACTOME_GPVI:MEDIATED_ACTIVATION_CASCADE	0.18
MP:0008658	decreased interleukin-1 beta secretion	0.18
ENSG00000163956	LRPAP1 PPI subnetwork	0.18
GO:0016528	sarcoplasm	0.18
ENSG00000082781	ITGB5 PPI subnetwork	0.18
GO:0045069	regulation of viral genome replication	0.18
MP:0005048	thrombosis	0.18
ENSG00000206308	HLA-DRA PPI subnetwork	0.18
GO:0005635	nuclear envelope	0.18
GO:0044420	extracellular matrix part	0.18
REACTOME_INTERFERON_SIGNALING	REACTOME_INTERFERON_SIGNALING	0.18
MP:0000706	small thymus	0.18
GO:0051569	regulation of histone H3-K4 methylation	0.18
ENSG00000204983	PRSS1 PPI subnetwork	0.18
MP:0004841	abnormal small intestine crypts of Lieberkuhn morphology	0.18
ENSG00000115866	DARS PPI subnetwork	0.18
GO:0001825	blastocyst formation	0.18
MP:0009414	skeletal muscle fiber necrosis	0.18
ENSG00000173039	RELA PPI subnetwork	0.18
KEGG_ENDOCYTOSIS	KEGG_ENDOCYTOSIS	0.18
ENSG00000171223	JUNB PPI subnetwork	0.18
ENSG00000136110	LECT1 PPI subnetwork	0.18
GO:0005537	mannose binding	0.18
ENSG00000138326	RPS24 PPI subnetwork	0.18

Original gene set ID	Original gene set description	Nominal P value
ENSG00000183311	TUBB PPI subnetwork	0.18
ENSG00000196230	TUBB PPI subnetwork	0.18
ENSG00000137379	ENSG00000137379 PPI subnetwork	0.18
GO:0004659	prenyltransferase activity	0.18
MP:0004229	abnormal embryonic erythropoiesis	0.18
MP:0000554	abnormal carpal bone morphology	0.18
ENSG00000133511	ENSG00000133511 PPI subnetwork	0.18
REACTOME_PLATELET_ACTIVATION_SIGNALING_AND_AGGREGATION	REACTOME_PLATELET_ACTIVATION_SIGNALING_AND_AGGREGATION	0.18
GO:0070085	glycosylation	0.18
ENSG00000133935	C14orf1 PPI subnetwork	0.18
REACTOME_NF:KB_ACTIVATION_THROUGH_FADDRIP:1_PATHWAY_MEDIATED_	REACTOME_NF:KB_ACTIVATION_THROUGH_FADDRIP:1_PATHWAY_MEDIATED_BY_	0.18
MP:0002724	enhanced wound healing	0.18
ENSG00000110169	HPX PPI subnetwork	0.18
REACTOME_PLATELET_AGGREGATION_PLUG_FORMATION	REACTOME_PLATELET_AGGREGATION_PLUG_FORMATION	0.18
MP:0009743	preaxial polydactyly	0.18
ENSG00000188223	LIN37 PPI subnetwork	0.18
ENSG00000182774	RPS17L PPI subnetwork	0.18
ENSG00000184779	RPS17 PPI subnetwork	0.18
GO:0004675	transmembrane receptor protein serine/threonine kinase activity	0.18
MP:0009940	abnormal hippocampus pyramidal cell morphology	0.18
MP:0000928	incomplete cephalic closure	0.18
MP:0002666	increased circulating aldosterone leve	0.18
REACTOME_TERMINATION_OF_O:GLYCAN_BIOSYNTHESIS	REACTOME_TERMINATION_OF_O:GLYCAN_BIOSYNTHESIS	0.18
GO:0015924	mannosyl-oligosaccharide mannosidase activity	0.18
ENSG00000133805	AMPD3 PPI subnetwork	0.18
ENSG00000106125	FAM188B PPI subnetwork	0.18
ENSG00000157168	NRG1 PPI subnetwork	0.18
MP:0005025	abnormal response to infection	0.18
ENSG00000183405	ENSG00000183405 PPI subnetwork	0.18
ENSG00000182446	NPLOC4 PPI subnetwork	0.18
MP:0001819	abnormal immune cell physiology	0.18
MP:0003408	increased width of hypertrophic chondrocyte zone	0.18
MP:0009142	decreased prepulse inhibition	0.18
REACTOME_CYTOKINE_SIGNALING_IN_IMMUNE_SYSTEM	REACTOME_CYTOKINE_SIGNALING_IN_IMMUNE_SYSTEM	0.18
GO:0032680	regulation of tumor necrosis factor production	0.18
GO:0032640	tumor necrosis factor production	0.18
MP:0004808	abnormal hematopoietic stem cell morphology	0.18
GO:0043330	response to exogenous dsRNA	0.18
GO:0002922	positive regulation of humoral immune response	0.18
MP:0002432	abnormal CD4-positive T cell morphology	0.18
ENSG00000188529	SRSF10 PPI subnetwork	0.18
ENSG00000160213	CSTB PPI subnetwork	0.18
MP:0010067	increased red blood cell distribution width	0.18
ENSG00000111845	PAK1IP1 PPI subnetwork	0.18
ENSG00000198467	TPM2 PPI subnetwork	0.18
ENSG00000163513	TGFBR2 PPI subnetwork	0.18
ENSG00000075539	FRYL PPI subnetwork	0.18
MP:0006059	decreased susceptibility to ischemic brain injury	0.18
ENSG00000186468	RPS23 PPI subnetwork	0.18

Original gene set ID	Original gene set description	Nominal P value
GO:0003697	single-stranded DNA binding	0.18
ENSG00000155926	SLA PPI subnetwork	0.18
ENSG00000136891	TEX10 PPI subnetwork	0.18
MP:0011108	partial embryonic lethality during organogenesis	0.18
GO:0032008	positive regulation of TOR signaling cascade	0.18
ENSG00000198265	HELZ PPI subnetwork	0.18
ENSG00000106617	PRKAG2 PPI subnetwork	0.18
GO:0051129	negative regulation of cellular component organization	0.18
ENSG00000121653	MAPK8IP1 PPI subnetwork	0.18
GO:0072659	protein localization in plasma membrane	0.18
ENSG00000176273	SLC35G1 PPI subnetwork	0.19
GO:0043413	macromolecule glycosylation	0.19
GO:0006486	protein glycosylation	0.19
MP:0003799	impaired macrophage chemotaxis	0.19
MP:0002644	decreased circulating triglyceride level	0.19
ENSG00000205813	ENSG00000205813 PPI subnetwork	0.19
ENSG00000109272	PF4V1 PPI subnetwork	0.19
GO:0002456	T cell mediated immunity	0.19
ENSG00000161202	DVL3 PPI subnetwork	0.19
GO:0015144	carbohydrate transmembrane transporter activity	0.19
ENSG00000197561	ELANE PPI subnetwork	0.19
MP:0001879	abnormal lymphatic vessel morphology	0.19
GO:0046982	protein heterodimerization activity	0.19
ENSG00000168214	RBPJ PPI subnetwork	0.19
MP:0001698	decreased embryo size	0.19
GO:0045834	positive regulation of lipid metabolic process	0.19
ENSG00000103510	KAT8 PPI subnetwork	0.19
REACTOME_REGULATION_OF_IFNA_SIGNALING	REACTOME_REGULATION_OF_IFNA_SIGNALING	0.19
ENSG00000122406	RPL5 PPI subnetwork	0.19
ENSG00000111615	KRR1 PPI subnetwork	0.19
ENSG00000185479	KRT6B PPI subnetwork	0.19
ENSG00000137807	KIF23 PPI subnetwork	0.19
MP:0000440	domed cranium	0.19
REACTOME_ETHANOL_OXIDATION	REACTOME_ETHANOL_OXIDATION	0.19
GO:0010830	regulation of myotube differentiation	0.19
MP:0004351	short humerus	0.19
ENSG00000158417	EIF5B PPI subnetwork	0.19
GO:0030695	GTPase regulator activity	0.19
GO:0004198	calcium-dependent cysteine-type endopeptidase activity	0.19
GO:0060541	respiratory system development	0.19
MP:0008083	decreased single-positive T cell number	0.19
MP:0002461	increased immunoglobulin level	0.19
GO:0017119	Golgi transport complex	0.19
GO:0005875	microtubule associated complex	0.19
MP:0000060	delayed bone ossification	0.19
REACTOME_SIGNALING_BY_INTERLEUKINS	REACTOME_SIGNALING_BY_INTERLEUKINS	0.19
GO:0008209	androgen metabolic process	0.19
REACTOME_ASPARAGINE_N:LINKED_GLYCOSYLATION	REACTOME_ASPARAGINE_N:LINKED_GLYCOSYLATION	0.19
GO:0045987	positive regulation of smooth muscle contraction	0.19

Original gene set ID	Original gene set description	Nominal P value
GO:0031985	Golgi cisterna	0.19
ENSG00000118402	ELOVL4 PPI subnetwork	0.19
ENSG00000104969	SGTA PPI subnetwork	0.19
MP:0003984	embryonic growth retardation	0.19
KEGG_CELL_CYCLE	KEGG_CELL_CYCLE	0.19
ENSG00000145781	COMMD10 PPI subnetwork	0.19
GO:0043492	ATPase activity, coupled to movement of substances	0.19
ENSG00000169083	AR PPI subnetwork	0.19
ENSG00000115718	PROC PPI subnetwork	0.19
ENSG00000083845	RPS5 PPI subnetwork	0.19
ENSG00000197959	DNM3 PPI subnetwork	0.19
GO:0043401	steroid hormone mediated signaling pathway	0.19
MP:0003216	absence seizures	0.19
MP:0001289	persistence of hyaloid vascular system	0.19
ENSG00000064012	CASP8 PPI subnetwork	0.19
ENSG00000119408	NEK6 PPI subnetwork	0.19
MP:0001622	abnormal vasculogenesis	0.19
ENSG00000113520	IL4 PPI subnetwork	0.19
ENSG00000196656	ENSG00000196656 PPI subnetwork	0.19
ENSG00000197728	RPS26 PPI subnetwork	0.19
ENSG00000117410	ATP6V0B PPI subnetwork	0.19
REACTOME_METABOLISM_OF_STEROID_HORMONES_AND_VITAMINS_A_AND_	REACTOME_METABOLISM_OF_STEROID_HORMONES_AND_VITAMINS_A_AND_D	0.19
ENSG00000164022	AIMP1 PPI subnetwork	0.19
MP:0001127	small ovary	0.19
MP:0000291	enlarged pericardium	0.19
ENSG00000163347	CLDN1 PPI subnetwork	0.19
ENSG00000112118	MCM3 PPI subnetwork	0.19
ENSG00000163050	ADCK3 PPI subnetwork	0.19
GO:0032480	negative regulation of type I interferon production	0.19
GO:0090179	planar cell polarity pathway involved in neural tube closure	0.19
GO:0090178	regulation of establishment of planar polarity involved in neural tube closure	0.19
MP:0001601	abnormal myelopoiesis	0.19
ENSG00000106462	EZH2 PPI subnetwork	0.19
REACTOME_RNA_POLYMERASE_III_ABORTIVE_AND_RETRACTIVE_INITIATION	REACTOME_RNA_POLYMERASE_III_ABORTIVE_AND_RETRACTIVE_INITIATION	0.19
REACTOME_RNA_POLYMERASE_III_TRANSCRIPTION	REACTOME_RNA_POLYMERASE_III_TRANSCRIPTION	0.19
ENSG00000196365	LONP1 PPI subnetwork	0.19
MP:0002078	abnormal glucose homeostasis	0.19
ENSG00000006062	MAP3K14 PPI subnetwork	0.19
ENSG00000077150	NFKB2 PPI subnetwork	0.19
ENSG00000166197	NOLC1 PPI subnetwork	0.19
ENSG00000133101	CCNA1 PPI subnetwork	0.19
GO:0014706	striated muscle tissue development	0.19
MP:0010300	increased skin tumor incidence	0.19
GO:0009749	response to glucose stimulus	0.19
ENSG00000013441	CLK1 PPI subnetwork	0.19
ENSG00000120800	UTP20 PPI subnetwork	0.19
ENSG00000175387	SMAD2 PPI subnetwork	0.19
ENSG00000124006	OBSL1 PPI subnetwork	0.19
GO:0032494	response to peptidoglycan	0.19

Original gene set ID	Original gene set description	Nominal P value
REACTOME_GLUCOSE_METABOLISM	REACTOME_GLUCOSE_METABOLISM	0.19
ENSG00000198517	MAFK PPI subnetwork	0.19
MP:0005185	decreased circulating progesterone level	0.19
GO:0055017	cardiac muscle tissue growth	0.19
MP:0002543	brachyphalangia	0.19
GO:0007266	Rho protein signal transduction	0.19
MP:0001802	arrested B cell differentiation	0.19
ENSG00000182393	IL29 PPI subnetwork	0.19
ENSG00000111536	IL26 PPI subnetwork	0.19
ENSG00000142224	IL19 PPI subnetwork	0.19
ENSG00000145839	IL9 PPI subnetwork	0.19
ENSG00000183709	IL28A PPI subnetwork	0.19
ENSG00000162892	IL24 PPI subnetwork	0.19
ENSG00000104432	IL7 PPI subnetwork	0.19
ENSG00000177047	IFNW1 PPI subnetwork	0.19
ENSG00000164136	IL15 PPI subnetwork	0.19
ENSG00000147896	IFNK PPI subnetwork	0.19
ENSG00000138684	IL21 PPI subnetwork	0.19
ENSG00000128342	LIF PPI subnetwork	0.19
ENSG00000184995	IFNE PPI subnetwork	0.19
ENSG00000197110	IL28B PPI subnetwork	0.19
GO:0003779	actin binding	0.19
ENSG00000104938	CLEC4M PPI subnetwork	0.19
GO:0000803	sex chromosome	0.19
GO:0017153	sodium:dicarboxylate symporter activity	0.19
ENSG00000110944	IL23A PPI subnetwork	0.19
GO:0007530	sex determination	0.19
ENSG00000188994	ZNF292 PPI subnetwork	0.19
MP:0011506	glomerular crescent	0.19
MP:0000572	abnormal autopod morphology	0.19
KEGG_COLORECTAL_CANCER	KEGG_COLORECTAL_CANCER	0.19
MP:0008102	lymph node hyperplasia	0.19
GO:0007548	sex differentiation	0.19
ENSG00000213764	ENSG00000213764 PPI subnetwork	0.2
ENSG00000196459	TRAPPC2 PPI subnetwork	0.2
ENSG00000105202	FBL PPI subnetwork	0.2
GO:0030159	receptor signaling complex scaffold activity	0.2
ENSG00000010278	CD9 PPI subnetwork	0.2
GO:0002690	positive regulation of leukocyte chemotaxis	0.2
ENSG00000107282	APBA1 PPI subnetwork	0.2
KEGG_NOTCH_SIGNALING_PATHWAY	KEGG_NOTCH_SIGNALING_PATHWAY	0.2
ENSG00000198646	NCOA6 PPI subnetwork	0.2
ENSG00000131051	RBM39 PPI subnetwork	0.2
ENSG00000154134	ROBO3 PPI subnetwork	0.2
MP:0008347	decreased gamma-delta T cell number	0.2
ENSG00000130396	MLLT4 PPI subnetwork	0.2
ENSG00000145192	AHSG PPI subnetwork	0.2
ENSG00000170027	YWHAG PPI subnetwork	0.2
ENSG00000132603	NIP7 PPI subnetwork	0.2

Original gene set ID	Original gene set description	Nominal P value
ENSG00000156482	RPL30 PPI subnetwork	0.2
ENSG00000103266	STUB1 PPI subnetwork	0.2
GO:0050776	regulation of immune response	0.2
ENSG00000116962	NID1 PPI subnetwork	0.2
ENSG00000143520	FLG2 PPI subnetwork	0.2
ENSG00000138722	MMRN1 PPI subnetwork	0.2
ENSG00000090861	AARS PPI subnetwork	0.2
GO:0015908	fatty acid transport	0.2
GO:0008017	microtubule binding	0.2
ENSG00000082641	NFE2L1 PPI subnetwork	0.2
REACTOME_EUKARYOTIC_TRANSLATION_INITIATION	REACTOME_EUKARYOTIC_TRANSLATION_INITIATION	0.2
REACTOME_CAP:DEPENDENT_TRANSLATION_INITIATION	REACTOME_CAP:DEPENDENT_TRANSLATION_INITIATION	0.2
ENSG00000163162	RNF149 PPI subnetwork	0.2
GO:0001817	regulation of cytokine production	0.2
REACTOME_HORMONE_LIGAND:BINDING_RECEPTORS	REACTOME_HORMONE_LIGAND:BINDING_RECEPTORS	0.2
GO:0015813	L-glutamate transport	0.2
GO:0006869	lipid transport	0.2
REACTOME_GROWTH_HORMONE_RECEPTOR_SIGNALING	REACTOME_GROWTH_HORMONE_RECEPTOR_SIGNALING	0.2
GO:0008088	axon cargo transport	0.2
GO:0002444	myeloid leukocyte mediated immunity	0.2
ENSG00000166167	BTRC PPI subnetwork	0.2
ENSG00000074071	MRPS34 PPI subnetwork	0.2
MP:0001195	flaky skin	0.2
GO:0010717	regulation of epithelial to mesenchymal transition	0.2
ENSG00000161203	AP2M1 PPI subnetwork	0.2
ENSG00000105127	AKAP8 PPI subnetwork	0.2
ENSG00000213044	ENSG00000213044 PPI subnetwork	0.2
GO:0001913	T cell mediated cytotoxicity	0.2
ENSG00000030110	BAK1 PPI subnetwork	0.2
MP:0001194	dermatitis	0.2
ENSG00000172179	PRL PPI subnetwork	0.2
ENSG00000122026	RPL21 PPI subnetwork	0.2
ENSG00000164924	YWHAZ PPI subnetwork	0.2
GO:0016500	protein-hormone receptor activity	0.2
ENSG00000156976	EIF4A2 PPI subnetwork	0.2
MP:0000652	enlarged sebaceous gland	0.2
GO:0002253	activation of immune response	0.2
ENSG00000100603	SNW1 PPI subnetwork	0.2
GO:0043434	response to peptide hormone stimulus	0.2
MP:0001258	decreased body length	0.2
MP:0004607	abnormal cervical atlas morphology	0.2
ENSG00000179409	GEMIN4 PPI subnetwork	0.2
MP:0000167	decreased chondrocyte cell number	0.2
ENSG00000033011	ALG1 PPI subnetwork	0.2
ENSG00000169992	NLGN2 PPI subnetwork	0.2
GO:0012507	ER to Golgi transport vesicle membrane	0.2
MP:0001154	seminiferous tubule degeneration	0.2
GO:0051184	cofactor transporter activity	0.2
REACTOME_RNA_POLYMERASE_III_TRANSCRIPTION_TERMINATION	REACTOME_RNA_POLYMERASE_III_TRANSCRIPTION_TERMINATION	0.2

Original gene set ID	Original gene set description	Nominal P value
GO:0071383	cellular response to steroid hormone stimulus	0.2
ENSG00000113407	TARS PPI subnetwork	0.2
ENSG00000124228	DDX27 PPI subnetwork	0.2
MP:0003227	abnormal vascular branching morphogenesis	0.2
GO:0045351	type I interferon biosynthetic process	0.2
GO:2000106	regulation of leukocyte apoptotic process	0.2
REACTOME_GLYCOLYSIS	REACTOME_GLYCOLYSIS	0.2
ENSG00000102391	ENSG00000102391 PPI subnetwork	0.2
ENSG00000035403	VCL PPI subnetwork	0.2
ENSG00000164134	NAA15 PPI subnetwork	0.2
ENSG00000164399	IL3 PPI subnetwork	0.2
GO:0043901	negative regulation of multi-organism process	0.2
MP:0000825	dilated lateral ventricles	0.2
ENSG00000119285	HEATR1 PPI subnetwork	0.2
ENSG00000011243	AKAP8L PPI subnetwork	0.2
GO:0016485	protein processing	0.2
ENSG00000160285	LSS PPI subnetwork	0.2
ENSG00000127022	CANX PPI subnetwork	0.2
MP:0000462	abnormal digestive system morphology	0.2
ENSG00000005961	ITGA2B PPI subnetwork	0.2
GO:0061097	regulation of protein tyrosine kinase activity	0.2
GO:0048770	pigment granule	0.2
GO:0042470	melanosome	0.2
ENSG00000144713	RPL32 PPI subnetwork	0.2
REACTOME_3_:UTR:MEDIATED_TRANSLATIONAL_REGULATION	REACTOME_3_:UTR:MEDIATED_TRANSLATIONAL_REGULATION	0.2
REACTOME_L13A:MEDIATED_TRANSLATIONAL_SILENCING_OF_CERULOPLASMI	REACTOME_L13A:MEDIATED_TRANSLATIONAL_SILENCING_OF_CERULOPLASMIN_E	0.2
ENSG00000114391	RPL24 PPI subnetwork	0.2
GO:0042278	purine nucleoside metabolic process	0.2
KEGG_MTOR_SIGNALING_PATHWAY	KEGG_MTOR_SIGNALING_PATHWAY	0.2
ENSG00000215412	ENSG00000215412 PPI subnetwork	0.2
ENSG00000198563	DDX39B PPI subnetwork	0.2
ENSG00000215425	DDX39B PPI subnetwork	0.2
GO:0050921	positive regulation of chemotaxis	0.2
ENSG00000123159	GIPC1 PPI subnetwork	0.2
ENSG00000169194	IL13 PPI subnetwork	0.2
ENSG00000038002	AGA PPI subnetwork	0.2
MP:0005091	increased double-positive T cell number	0.2
MP:0003720	abnormal neural tube closure	0.2
MP:0001260	increased body weight	0.2
GO:0006342	chromatin silencing	0.2
GO:0006006	glucose metabolic process	0.2
GO:0055102	lipase inhibitor activity	0.2
REACTOME_TRAF3:DEPENDENT_IRF_ACTIVATION_PATHWAY	REACTOME_TRAF3:DEPENDENT_IRF_ACTIVATION_PATHWAY	0.2
MP:0008511	thin retinal inner nuclear layer	0.2
MP:0000715	decreased thymocyte number	0.2
GO:0008289	lipid binding	0.2
ENSG00000131069	ACSS2 PPI subnetwork	0.2
ENSG00000205246	RPSAP58 PPI subnetwork	0.2
GO:0008593	regulation of Notch signaling pathway	0.2

Original gene set ID	Original gene set description	Nominal P value
REACTOME_EUKARYOTIC_TRANSLATION_TERMINATION	REACTOME_EUKARYOTIC_TRANSLATION_TERMINATION	0.2
ENSG00000112818	MEP1A PPI subnetwork	0.2
GO:0002367	cytokine production involved in immune response	0.21
ENSG00000171855	IFNB1 PPI subnetwork	0.21
MP:0009546	absent gastric milk in neonates	0.21
ENSG00000115461	IGFBP5 PPI subnetwork	0.21
ENSG00000073009	IKBKG PPI subnetwork	0.21
ENSG00000131469	RPL27 PPI subnetwork	0.21
GO:0009881	photoreceptor activity	0.21
ENSG00000092098	RNF31 PPI subnetwork	0.21
ENSG00000105364	MRPL4 PPI subnetwork	0.21
ENSG00000171105	INSR PPI subnetwork	0.21
GO:0000030	mannosyltransferase activity	0.21
ENSG00000063438	AHRR PPI subnetwork	0.21
MP:0004110	transposition of great arteries	0.21
ENSG00000153391	INO80C PPI subnetwork	0.21
ENSG00000077809	GTF2I PPI subnetwork	0.21
GO:0044454	nuclear chromosome part	0.21
GO:0034612	response to tumor necrosis factor	0.21
MP:0008042	abnormal NK T cell physiology	0.21
MP:0011089	complete perinatal lethality	0.21
MP:0001613	abnormal vasodilation	0.21
ENSG00000108107	RPL28 PPI subnetwork	0.21
ENSG00000144218	AFF3 PPI subnetwork	0.21
MP:0000165	abnormal long bone hypertrophic chondrocyte zone	0.21
ENSG00000134259	NGF PPI subnetwork	0.21
MP:0002896	abnormal bone mineralization	0.21
GO:0006506	GPI anchor biosynthetic process	0.21
GO:0005343	organic acid:sodium symporter activity	0.21
ENSG00000136531	SCN2A PPI subnetwork	0.21
ENSG00000121152	NCAPH PPI subnetwork	0.21
ENSG00000196681	ENSG00000196681 PPI subnetwork	0.21
ENSG00000176534	ENSG00000176534 PPI subnetwork	0.21
ENSG00000198637	ENSG00000198637 PPI subnetwork	0.21
GO:0005031	tumor necrosis factor-activated receptor activity	0.21
GO:0009167	purine ribonucleoside monophosphate metabolic process	0.21
GO:0009126	purine nucleoside monophosphate metabolic process	0.21
MP:0003567	abnormal fetal cardiomyocyte proliferation	0.21
ENSG00000143190	POU2F1 PPI subnetwork	0.21
ENSG00000108342	CSF3 PPI subnetwork	0.21
ENSG00000105639	JAK3 PPI subnetwork	0.21
ENSG00000155363	MOV10 PPI subnetwork	0.21
ENSG00000174444	RPL4 PPI subnetwork	0.21
ENSG00000119729	RHOQ PPI subnetwork	0.21
ENSG00000136942	RPL35 PPI subnetwork	0.21
REACTOME_CELL:EXTRACELLULAR_MATRIX_INTERACTIONS	REACTOME_CELL:EXTRACELLULAR_MATRIX_INTERACTIONS	0.21
GO:0035088	establishment or maintenance of apical/basal cell polarity	0.21
GO:0061245	establishment or maintenance of bipolar cell polarity	0.21
ENSG00000206509	HLA-F PPI subnetwork	0.21

Original gene set ID	Original gene set description	Nominal P value
REACTOME_PYRUVATE_METABOLISM	REACTOME_PYRUVATE_METABOLISM	0.21
MP:0009050	dilated proximal convoluted tubules	0.21
GO:0005283	sodium:amino acid symporter activity	0.21
MP:0001511	disheveled coat	0.21
ENSG00000168884	TNIP2 PPI subnetwork	0.21
ENSG00000184634	MED12 PPI subnetwork	0.21
GO:0004860	protein kinase inhibitor activity	0.21
MP:0004969	pale kidney	0.21
GO:0009755	hormone-mediated signaling pathway	0.21
MP:0008578	decreased circulating interferon-gamma level	0.21
KEGG_TIGHT_JUNCTION	KEGG_TIGHT_JUNCTION	0.21
GO:0030204	chondroitin sulfate metabolic process	0.21
MP:0011097	complete embryonic lethality before turning of embryo	0.21
GO:0023019	signal transduction involved in regulation of gene expression	0.21
MP:0006400	decreased molar number	0.21
ENSG00000134308	YWHAQ PPI subnetwork	0.21
ENSG00000174123	TLR10 PPI subnetwork	0.21
GO:0006022	aminoglycan metabolic process	0.21
ENSG00000077235	GTF3C1 PPI subnetwork	0.21
REACTOME_AMINO_ACID_AND_OLIGOPEPTIDE_SLC_TRANSPORTERS	REACTOME_AMINO_ACID_AND_OLIGOPEPTIDE_SLC_TRANSPORTERS	0.21
MP:0001212	skin lesions	0.21
ENSG00000111424	VDR PPI subnetwork	0.21
MP:0000470	abnormal stomach morphology	0.21
MP:0000857	abnormal cerebellar foliation	0.21
ENSG00000114841	DNAH1 PPI subnetwork	0.21
GO:0008211	glucocorticoid metabolic process	0.21
MP:0008947	increased neuron number	0.21
GO:0030134	ER to Golgi transport vesicle	0.21
GO:0002687	positive regulation of leukocyte migration	0.21
GO:0010466	negative regulation of peptidase activity	0.21
MP:0004737	absent distortion product otoacoustic emissions	0.21
ENSG00000124614	RPS10 PPI subnetwork	0.21
GO:0042578	phosphoric ester hydrolase activity	0.21
ENSG00000173848	NET1 PPI subnetwork	0.21
ENSG00000130427	EPO PPI subnetwork	0.21
MP:0000183	decreased circulating LDL cholesterol leve	0.21
ENSG00000163938	GNL3 PPI subnetwork	0.21
MP:0001683	absent mesoderm	0.21
MP:0002981	increased liver weight	0.21
GO:0071495	cellular response to endogenous stimulus	0.21
GO:0033043	regulation of organelle organization	0.21
ENSG00000130702	LAMA5 PPI subnetwork	0.21
GO:0001702	gastrulation with mouth forming second	0.21
ENSG00000070756	PABPC1 PPI subnetwork	0.21
MP:0004740	sensorineural hearing loss	0.21
ENSG00000070371	CLTCL1 PPI subnetwork	0.21
GO:0042107	cytokine metabolic process	0.21
ENSG00000153113	CAST PPI subnetwork	0.21
GO:0016810	hydrolase activity, acting on carbon-nitrogen (but not peptide) bonds	0.21

Original gene set ID	Original gene set description	Nominal P value
MP:0000694	spleen hypoplasia	0.21
ENSG00000177200	CHD9 PPI subnetwork	0.21
ENSG00000136718	IMP4 PPI subnetwork	0.21
ENSG00000164163	ABCE1 PPI subnetwork	0.22
MP:0006144	increased systemic arterial systolic blood pressure	0.22
ENSG00000164220	F2RL2 PPI subnetwork	0.22
MP:0005602	decreased angiogenesis	0.22
MP:0000465	gastrointestinal hemorrhage	0.22
GO:0002709	regulation of T cell mediated immunity	0.22
MP:0003659	abnormal lymph circulation	0.22
ENSG00000164751	PEX2 PPI subnetwork	0.22
GO:0010951	negative regulation of endopeptidase activity	0.22
MP:0005169	abnormal male meiosis	0.22
MP:0002467	impaired neutrophil phagocytosis	0.22
ENSG00000188846	RPL14 PPI subnetwork	0.22
ENSG00000108256	NUFIP2 PPI subnetwork	0.22
KEGG_MATURITY_ONSET_DIABETES_OF_THE_YOUNG	KEGG_MATURITY_ONSET_DIABETES_OF_THE_YOUNG	0.22
ENSG00000115414	FN1 PPI subnetwork	0.22
ENSG00000136869	TLR4 PPI subnetwork	0.22
ENSG00000108270	AATF PPI subnetwork	0.22
GO:0018200	peptidyl-glutamic acid modification	0.22
GO:0002230	positive regulation of defense response to virus by host	0.22
KEGG_RIBOSOME	KEGG_RIBOSOME	0.22
ENSG00000142541	RPL13A PPI subnetwork	0.22
GO:0008757	S-adenosylmethionine-dependent methyltransferase activity	0.22
GO:0008217	regulation of blood pressure	0.22
GO:0006417	regulation of translation	0.22
ENSG00000075391	RASAL2 PPI subnetwork	0.22
ENSG00000138442	WDR12 PPI subnetwork	0.22
ENSG00000099797	TECR PPI subnetwork	0.22
GO:0003727	single-stranded RNA binding	0.22
MP:0000601	small liver	0.22
MP:0002784	abnormal Sertoli cell morphology	0.22
GO:0008060	ARF GTPase activator activity	0.22
MP:0005244	hemopericardium	0.22
MP:0005559	increased circulating glucose level	0.22
GO:0019865	immunoglobulin binding	0.22
ENSG00000186141	POLR3C PPI subnetwork	0.22
GO:0032095	regulation of response to food	0.22
GO:0007018	microtubule-based movement	0.22
ENSG00000075643	MOCOS PPI subnetwork	0.22
MP:0002836	abnormal chorion morphology	0.22
ENSG00000185010	F8 PPI subnetwork	0.22
ENSG00000179958	DCTPP1 PPI subnetwork	0.22
GO:0015631	tubulin binding	0.22
ENSG00000149480	MTA2 PPI subnetwork	0.22
GO:0042771	DNA damage response, signal transduction by p53 class mediator resulting in induct	0.22
GO:0032760	positive regulation of tumor necrosis factor productior	0.22
GO:0044319	wound healing, spreading of cells	0.22

Original gene set ID	Original gene set description	Nominal P value
GO:0015721	bile acid and bile salt transport	0.22
ENSG00000082512	TRAF5 PPI subnetwork	0.22
KEGG_NEUROTROPHIN_SIGNALING_PATHWAY	KEGG_NEUROTROPHIN_SIGNALING_PATHWAY	0.22
MP:0008892	abnormal sperm flagellum morphology	0.22
ENSG00000134882	UBAC2 PPI subnetwork	0.22
ENSG00000140992	PDPK1 PPI subnetwork	0.22
GO:0071359	cellular response to dsRNA	0.22
ENSG00000103275	UBE2I PPI subnetwork	0.22
ENSG00000187899	ENSG00000187899 PPI subnetwork	0.22
MP:0008026	abnormal brain white matter morphology	0.22
MP:0008495	decreased IgG1 level	0.22
MP:0003044	impaired basement membrane formation	0.22
GO:0015935	small ribosomal subunit	0.22
GO:0006661	phosphatidylinositol biosynthetic process	0.22
ENSG00000113302	IL12B PPI subnetwork	0.22
GO:0015800	acidic amino acid transport	0.22
ENSG00000135845	PIGC PPI subnetwork	0.22
ENSG00000170727	BOP1 PPI subnetwork	0.22
ENSG00000114739	ACVR2B PPI subnetwork	0.22
GO:0034762	regulation of transmembrane transport	0.22
ENSG00000115816	CEBPZ PPI subnetwork	0.22
ENSG00000123064	DDX54 PPI subnetwork	0.22
MP:0000443	abnormal snout morphology	0.22
MP:0009763	increased sensitivity to induced morbidity/mortality	0.22
GO:0021700	developmental maturation	0.22
MP:0002217	small lymph nodes	0.22
GO:0001945	lymph vessel development	0.22
MP:0000250	abnormal vasoconstriction	0.22
GO:0007176	regulation of epidermal growth factor-activated receptor activity	0.22
ENSG00000146648	EGFR PPI subnetwork	0.22
GO:0021697	cerebellar cortex formation	0.22
ENSG00000198373	WWP2 PPI subnetwork	0.22
ENSG00000188976	NOC2L PPI subnetwork	0.22
ENSG00000184863	RBM33 PPI subnetwork	0.22
GO:0060038	cardiac muscle cell proliferation	0.22
ENSG00000111605	CPSF6 PPI subnetwork	0.22
ENSG00000162407	PPAP2B PPI subnetwork	0.22
ENSG00000135047	CTSL1 PPI subnetwork	0.22
GO:0004089	carbonate dehydratase activity	0.22
ENSG00000106397	PLOD3 PPI subnetwork	0.22
GO:0046466	membrane lipid catabolic process	0.22
GO:0030149	sphingolipid catabolic process	0.22
MP:0003983	decreased cholesterol level	0.22
GO:0050778	positive regulation of immune response	0.22
KEGG_VALINE_LEUCINE_AND_ISOLEUCINE_BIOSYNTHESIS	KEGG_VALINE_LEUCINE_AND_ISOLEUCINE_BIOSYNTHESIS	0.22
MP:0001547	abnormal lipid level	0.22
ENSG00000184185	KCNJ12 PPI subnetwork	0.22
ENSG00000168907	PLA2G4F PPI subnetwork	0.22
ENSG00000131791	PRKAB2 PPI subnetwork	0.22

Original gene set ID	Original gene set description	Nominal P value
ENSG00000166313	APBB1 PPI subnetwork	0.22
GO:0022415	viral reproductive process	0.22
ENSG00000135090	TAOK3 PPI subnetwork	0.22
MP:0004014	abnormal uterine environment	0.22
MP:0002001	blindness	0.22
MP:0002689	abnormal molar morphology	0.22
GO:0004683	calmodulin-dependent protein kinase activity	0.22
ENSG00000145833	DDX46 PPI subnetwork	0.22
ENSG00000118513	MYB PPI subnetwork	0.22
GO:0016569	covalent chromatin modification	0.22
ENSG00000097007	ABL1 PPI subnetwork	0.22
MP:0001577	anemia	0.22
MP:0002551	abnormal blood coagulation	0.22
ENSG00000179222	MAGED1 PPI subnetwork	0.22
ENSG00000138398	PPIG PPI subnetwork	0.22
GO:0002252	immune effector process	0.22
MP:0001242	hyperkeratosis	0.22
GO:0050688	regulation of defense response to virus	0.22
MP:0001722	pale yolk sac	0.22
GO:0042476	odontogenesis	0.22
MP:0001274	curly vibrissae	0.22
GO:0060338	regulation of type I interferon-mediated signaling pathway	0.22
ENSG00000117266	CDK18 PPI subnetwork	0.22
GO:0072509	divalent inorganic cation transmembrane transporter activity	0.22
MP:0004362	cochlear hair cell degeneration	0.22
GO:0048730	epidermis morphogenesis	0.22
GO:0048732	gland development	0.22
GO:0016884	carbon-nitrogen ligase activity, with glutamine as amido-N-donor	0.23
GO:0005520	insulin-like growth factor binding	0.23
MP:0004989	decreased osteoblast cell number	0.23
ENSG00000180340	FZD2 PPI subnetwork	0.23
ENSG00000049759	NEDD4L PPI subnetwork	0.23
REACTOME_FORMATION_OF_A_POOL_OF_FREE_40S_SUBUNITS	REACTOME_FORMATION_OF_A_POOL_OF_FREE_40S_SUBUNITS	0.23
GO:0045823	positive regulation of heart contraction	0.23
GO:0007566	embryo implantation	0.23
MP:0008211	decreased mature B cell number	0.23
REACTOME_ANTIVIRAL_MECHANISM_BY_IFN:STIMULATED_GENES	REACTOME_ANTIVIRAL_MECHANISM_BY_IFN:STIMULATED_GENES	0.23
REACTOME_ISG15_ANTIVIRAL_MECHANISM	REACTOME_ISG15_ANTIVIRAL_MECHANISM	0.23
GO:0030662	coated vesicle membrane	0.23
ENSG00000034053	APBA2 PPI subnetwork	0.23
GO:0032481	positive regulation of type I interferon production	0.23
ENSG00000160293	VAV2 PPI subnetwork	0.23
MP:0008603	decreased circulating interleukin-4 level	0.23
ENSG00000013364	MVP PPI subnetwork	0.23
ENSG00000197766	CFD PPI subnetwork	0.23
ENSG00000140986	RPL3L PPI subnetwork	0.23
ENSG00000187498	COL4A1 PPI subnetwork	0.23
ENSG00000012983	MAP4K5 PPI subnetwork	0.23
MP:0002272	abnormal nervous system electrophysiology	0.23

Original gene set ID	Original gene set description	Nominal P value
GO:0004252	serine-type endopeptidase activity	0.23
REACTOME_ABCA_TRANSPORTERS_IN_LIPID_HOMEOSTASIS	REACTOME_ABCA_TRANSPORTERS_IN_LIPID_HOMEOSTASIS	0.23
ENSG00000124587	PEX6 PPI subnetwork	0.23
GO:0015171	amino acid transmembrane transporter activity	0.23
GO:0001569	patterning of blood vessels	0.23
MP:0006398	increased long bone epiphyseal plate size	0.23
ENSG00000186350	RXRA PPI subnetwork	0.23
ENSG00000140988	RPS2 PPI subnetwork	0.23
GO:0045746	negative regulation of Notch signaling pathway	0.23
MP:0000223	decreased monocyte cell number	0.23
GO:0071219	cellular response to molecule of bacterial origin	0.23
GO:0002448	mast cell mediated immunity	0.23
ENSG00000011422	PLAUR PPI subnetwork	0.23
GO:0042439	ethanolamine-containing compound metabolic process	0.23
GO:0019901	protein kinase binding	0.23
ENSG00000141837	CACNA1A PPI subnetwork	0.23
MP:0001891	hydroencephaly	0.23
ENSG00000175505	CLCF1 PPI subnetwork	0.23
GO:0060589	nucleoside-triphosphatase regulator activity	0.23
ENSG00000198641	ENSG00000198641 PPI subnetwork	0.23
ENSG00000134070	IRAK2 PPI subnetwork	0.23
GO:0072657	protein localization in membrane	0.23
GO:0035272	exocrine system development	0.23
GO:0010510	regulation of acetyl-CoA biosynthetic process from pyruvate	0.23
GO:0006086	acetyl-CoA biosynthetic process from pyruvate	0.23
ENSG00000101361	NOP56 PPI subnetwork	0.23
GO:0031667	response to nutrient levels	0.23
ENSG00000100448	CTSG PPI subnetwork	0.23
ENSG00000174469	CNTNAP2 PPI subnetwork	0.23
ENSG00000172531	PPP1CA PPI subnetwork	0.23
GO:0009086	methionine biosynthetic process	0.23
GO:0030176	integral to endoplasmic reticulum membrane	0.23
GO:0051650	establishment of vesicle localization	0.23
ENSG00000143851	PTPN7 PPI subnetwork	0.23
ENSG00000119812	FAM98A PPI subnetwork	0.23
ENSG00000116711	PLA2G4A PPI subnetwork	0.23
ENSG00000170145	SIK2 PPI subnetwork	0.23
ENSG00000164934	DCAF13 PPI subnetwork	0.23
ENSG00000150337	FCGR1A PPI subnetwork	0.23
ENSG00000171863	RPS7 PPI subnetwork	0.23
MP:0001004	abnormal retinal photoreceptor morphology	0.23
ENSG00000033800	PIAS1 PPI subnetwork	0.23
ENSG00000112715	VEGFA PPI subnetwork	0.23
ENSG00000064703	DDX20 PPI subnetwork	0.23
GO:0035019	somatic stem cell maintenance	0.23
GO:0033673	negative regulation of kinase activity	0.23
GO:0031227	intrinsic to endoplasmic reticulum membrane	0.23
ENSG00000076248	UNG PPI subnetwork	0.23
GO:0009792	embryo development ending in birth or egg hatching	0.23

Original gene set ID	Original gene set description	Nominal P value
GO:0004602	glutathione peroxidase activity	0.23
GO:0005765	lysosomal membrane	0.23
GO:0031018	endocrine pancreas development	0.23
ENSG00000163879	DNALI1 PPI subnetwork	0.23
GO:0002467	germinal center formation	0.23
ENSG00000111276	CDKN1B PPI subnetwork	0.23
ENSG00000088035	ALG6 PPI subnetwork	0.23
GO:0071222	cellular response to lipopolysaccharide	0.23
REACTOME_TAK1_ACTIVATES_NFKB_BY_PHOSPHORYLATION_AND_ACTIVATIOI	REACTOME_TAK1_ACTIVATES_NFKB_BY_PHOSPHORYLATION_AND_ACTIVATION_O	0.23
GO:0019953	sexual reproduction	0.23
ENSG00000155868	MED7 PPI subnetwork	0.23
ENSG00000160307	S100B PPI subnetwork	0.23
ENSG00000070193	FGF10 PPI subnetwork	0.23
MP:0001106	abnormal Schwann cell morphology	0.23
MP:0001125	abnormal oocyte morphology	0.23
GO:0032870	cellular response to hormone stimulus	0.23
GO:0015711	organic anion transport	0.23
MP:0001829	increased activated T cell number	0.23
ENSG00000213023	SYT3 PPI subnetwork	0.23
GO:0005868	cytoplasmic dynein complex	0.23
GO:0070371	ERK1 and ERK2 cascade	0.23
GO:0035265	organ growth	0.23
MP:0004804	decreased susceptibility to autoimmune diabetes	0.23
MP:0008723	impaired eosinophil recruitment	0.23
ENSG00000111707	SUDS3 PPI subnetwork	0.23
GO:0060041	retina development in camera-type eye	0.23
MP:0003402	decreased liver weight	0.24
GO:0005811	lipid particle	0.24
REACTOME_FATTY_ACYL:COA_BIOSYNTHESIS	REACTOME_FATTY_ACYL:COA_BIOSYNTHESIS	0.24
ENSG00000108298	RPL19 PPI subnetwork	0.24
GO:0043009	chordate embryonic development	0.24
GO:0050866	negative regulation of cell activation	0.24
GO:0031114	regulation of microtubule depolymerization	0.24
ENSG00000101680	LAMA1 PPI subnetwork	0.24
KEGG_NITROGEN_METABOLISM	KEGG_NITROGEN_METABOLISM	0.24
MP:0003009	abnormal cytokine secretion	0.24
MP:0003755	abnormal palate morphology	0.24
REACTOME_INHIBITION_OF_REPLICATION_INITIATION_OF_DAMAGED_DNA_BY	REACTOME_INHIBITION_OF_REPLICATION_INITIATION_OF_DAMAGED_DNA_BY_RE	0.24
GO:0032259	methylation	0.24
ENSG00000186081	KRT5 PPI subnetwork	0.24
ENSG00000211614	ENSG00000211614 PPI subnetwork	0.24
ENSG00000141736	ERBB2 PPI subnetwork	0.24
ENSG00000165458	INPPL1 PPI subnetwork	0.24
MP:0008721	abnormal chemokine level	0.24
MP:0003071	decreased vascular permeability	0.24
MP:0005087	decreased acute inflammation	0.24
ENSG00000100294	MCAT PPI subnetwork	0.24
MP:0009789	decreased susceptibility to bacterial infection induced morbidity/mortality	0.24
MP:0008080	abnormal CD8-positive T cell differentiation	0.24

Original gene set ID	Original gene set description	Nominal P value
GO:0007026	negative regulation of microtubule depolymerization	0.24
ENSG00000053372	MRTO4 PPI subnetwork	0.24
ENSG00000141101	NOB1 PPI subnetwork	0.24
GO:0032386	regulation of intracellular transport	0.24
ENSG00000163166	IWS1 PPI subnetwork	0.24
MP:0003542	abnormal vascular endothelial cell development	0.24
ENSG00000124181	PLCG1 PPI subnetwork	0.24
ENSG00000138378	STAT4 PPI subnetwork	0.24
GO:0002576	platelet degranulation	0.24
MP:0004091	abnormal Z lines	0.24
MP:0001859	kidney inflammation	0.24
MP:0001132	absent mature ovarian follicles	0.24
GO:0034097	response to cytokine stimulus	0.24
MP:0003732	abnormal retinal outer plexiform layer morphology	0.24
ENSG00000129824	RPS4Y1 PPI subnetwork	0.24
MP:0001145	abnormal male reproductive system morphology	0.24
ENSG00000161638	ITGA5 PPI subnetwork	0.24
ENSG00000069869	NEDD4 PPI subnetwork	0.24
ENSG00000082701	GSK3B PPI subnetwork	0.24
GO:0018205	peptidyl-lysine modification	0.24
GO:0055088	lipid homeostasis	0.24
GO:0045137	development of primary sexual characteristics	0.24
ENSG00000065268	WDR18 PPI subnetwork	0.24
ENSG00000128052	KDR PPI subnetwork	0.24
REACTOME_ACTIVATED_TAK1_MEDIATES_P38_MAPK_ACTIVATION	REACTOME_ACTIVATED_TAK1_MEDIATES_P38_MAPK_ACTIVATION	0.24
MP:0003308	abnormal cochlear sensory epithelium morphology	0.24
ENSG00000167658	EEF2 PPI subnetwork	0.24
MP:0005181	decreased circulating estradiol level	0.24
REACTOME_GLUTATHIONE_CONJUGATION	REACTOME_GLUTATHIONE_CONJUGATION	0.24
MP:0004392	abnormal CD8-positive T cell physiology	0.24
GO:0042089	cytokine biosynthetic process	0.24
GO:0043499	eukaryotic cell surface binding	0.24
ENSG00000105640	RPL18A PPI subnetwork	0.24
REACTOME_RIBOSOMAL_SCANNING_AND_START_CODON_RECOGNITION	REACTOME_RIBOSOMAL_SCANNING_AND_START_CODON_RECOGNITION	0.24
ENSG00000175899	A2M PPI subnetwork	0.24
GO:0002695	negative regulation of leukocyte activation	0.24
ENSG00000137547	MRPL15 PPI subnetwork	0.24
GO:0004180	carboxypeptidase activity	0.24
ENSG00000145604	SKP2 PPI subnetwork	0.24
MP:0009504	abnormal mammary gland epithelium morphology	0.24
GO:0031490	chromatin DNA binding	0.24
MP:0009409	abnormal skeletal muscle fiber type ratio	0.24
MP:0003853	dry skin	0.24
MP:0000243	myoclonus	0.24
GO:0045577	regulation of B cell differentiation	0.24
GO:0043409	negative regulation of MAPK cascade	0.24
GO:0032101	regulation of response to external stimulus	0.24
MP:0002855	abnormal cochlear ganglion morphology	0.24
GO:0050308	sugar-phosphatase activity	0.24

Original gene set ID	Original gene set description	Nominal P value
GO:0019203	carbohydrate phosphatase activity	0.24
GO:0030217	T cell differentiation	0.24
REACTOME_RIG:IMDA5_MEDIATED_INDUCTION_OF_IFN:ALPHABETA_PATHWA	REACTOME_RIG:IMDA5_MEDIATED_INDUCTION_OF_IFN:ALPHABETA_PATHWAYS	0.24
MP:0000162	lordosis	0.24
GO:0046649	lymphocyte activation	0.24
ENSG00000170248	PDCD6IP PPI subnetwork	0.24
ENSG00000089597	GANAB PPI subnetwork	0.24
ENSG00000185630	PBX1 PPI subnetwork	0.24
GO:0002886	regulation of myeloid leukocyte mediated immunity	0.24
ENSG00000044524	EPHA3 PPI subnetwork	0.24
GO:0006497	protein lipidation	0.24
GO:0033017	sarcoplasmic reticulum membrane	0.24
REACTOME_TRAF6_MEDIATED_IRF7_ACTIVATION	REACTOME_TRAF6_MEDIATED_IRF7_ACTIVATION	0.24
ENSG00000164362	TERT PPI subnetwork	0.24
ENSG00000148082	SHC3 PPI subnetwork	0.24
GO:0044452	nucleolar part	0.24
GO:0005200	structural constituent of cytoskeleton	0.24
MP:0000784	forebrain hypoplasia	0.24
REACTOME_TRANSPORT_OF_VITAMINS_NUCLEOSIDES_AND_RELATED_MOLEC	REACTOME_TRANSPORT_OF_VITAMINS_NUCLEOSIDES_AND_RELATED_MOLECULE!	0.24
GO:0008138	protein tyrosine/serine/threonine phosphatase activity	0.24
ENSG00000173406	DAB1 PPI subnetwork	0.24
GO:0042056	chemoattractant activity	0.24
MP:0010825	abnormal lung saccule morphology	0.24
ENSG00000075388	FGF4 PPI subnetwork	0.24
ENSG00000008988	RPS20 PPI subnetwork	0.24
GO:0006270	DNA-dependent DNA replication initiation	0.24
ENSG00000122484	RPAP2 PPI subnetwork	0.24
GO:0048608	reproductive structure development	0.24
ENSG00000152556	PFKM PPI subnetwork	0.24
ENSG00000104267	CA2 PPI subnetwork	0.24
ENSG00000168028	RPSA PPI subnetwork	0.24
MP:0005508	abnormal skeleton morphology	0.24
GO:0003229	ventricular cardiac muscle tissue development	0.24
ENSG00000133703	KRAS PPI subnetwork	0.24
MP:0001304	cataracts	0.24
MP:0002765	short fibula	0.24
ENSG00000005844	ITGAL PPI subnetwork	0.24
MP:0003641	small lung	0.24
ENSG00000160741	CRTC2 PPI subnetwork	0.24
GO:0046474	glycerophospholipid biosynthetic process	0.24
GO:0006633	fatty acid biosynthetic process	0.24
ENSG00000169067	ACTBL2 PPI subnetwork	0.24
MP:0002416	abnormal proerythroblast morphology	0.24
ENSG00000158874	APOA2 PPI subnetwork	0.24
ENSG00000204843	DCTN1 PPI subnetwork	0.24
ENSG00000064601	CTSA PPI subnetwork	0.24
MP:0008135	small Peyer's patches	0.24
MP:0009655	abnormal secondary palate development	0.24
ENSG00000164251	F2RL1 PPI subnetwork	0.25

Original gene set ID	Original gene set description	Nominal P value
GO:0034505	tooth mineralization	0.25
ENSG00000110700	RPS13 PPI subnetwork	0.25
ENSG00000168454	TXNDC2 PPI subnetwork	0.25
GO:0006469	negative regulation of protein kinase activity	0.25
REACTOME_VIRAL_MRNA_TRANSLATION	REACTOME_VIRAL_MRNA_TRANSLATION	0.25
MP:0005503	abnormal tendon morphology	0.25
GO:0035924	cellular response to vascular endothelial growth factor stimulus	0.25
ENSG00000100302	RASD2 PPI subnetwork	0.25
MP:0000276	heart right ventricle hypertrophy	0.25
ENSG00000167978	SRRM2 PPI subnetwork	0.25
GO:0000097	sulfur amino acid biosynthetic process	0.25
MP:0008720	impaired neutrophil chemotaxis	0.25
GO:0001525	angiogenesis	0.25
MP:0008133	decreased Peyer's patch number	0.25
GO:2001014	regulation of skeletal muscle cell differentiation	0.25
GO:0008236	serine-type peptidase activity	0.25
GO:0003743	translation initiation factor activity	0.25
ENSG00000162409	PRKAA2 PPI subnetwork	0.25
ENSG00000105197	TIMM50 PPI subnetwork	0.25
ENSG00000164338	UTP15 PPI subnetwork	0.25
MP:0005168	abnormal female meiosis	0.25
MP:0002563	shortened circadian period	0.25
ENSG00000166862	CACNG2 PPI subnetwork	0.25
ENSG00000110987	BCL7A PPI subnetwork	0.25
MP:0003945	abnormal lymphocyte physiology	0.25
ENSG00000082175	PGR PPI subnetwork	0.25
GO:0001725	stress fiber	0.25
GO:0002521	leukocyte differentiation	0.25
GO:0006475	internal protein amino acid acetylation	0.25
GO:0042306	regulation of protein import into nucleus	0.25
GO:0005902	microvillus	0.25
MP:0001245	thick dermal layer	0.25
ENSG00000197498	RPF2 PPI subnetwork	0.25
GO:0019229	regulation of vasoconstriction	0.25
MP:0001211	wrinkled skin	0.25
ENSG00000134398	ERN2 PPI subnetwork	0.25
MP:0005093	decreased B cell proliferation	0.25
ENSG00000067842	ATP2B3 PPI subnetwork	0.25
GO:0016592	mediator complex	0.25
MP:0001712	abnormal placenta development	0.25
ENSG00000161016	RPL8 PPI subnetwork	0.25
ENSG00000136485	DCAF7 PPI subnetwork	0.25
MP:0000762	abnormal tongue morphology	0.25
GO:0006903	vesicle targeting	0.25
GO:0043596	nuclear replication fork	0.25
ENSG00000215467	ENSG00000215467 PPI subnetwork	0.25
GO:0051261	protein depolymerization	0.25
MP:0005058	abnormal lysosome morphology	0.25
GO:0005773	vacuole	0.25

Original gene set ID	Original gene set description	Nominal P value
GO:0004364	glutathione transferase activity	0.25
GO:0043242	negative regulation of protein complex disassembly	0.25
ENSG00000166441	RPL27A PPI subnetwork	0.25
GO:0042304	regulation of fatty acid biosynthetic process	0.25
MP:0003205	testicular atrophy	0.25
ENSG00000198821	CD247 PPI subnetwork	0.25
GO:0001819	positive regulation of cytokine production	0.25
MP:0002723	abnormal immune serum protein physiology	0.25
GO:0005581	collagen	0.25
MP:0000018	small ears	0.25
GO:0002064	epithelial cell development	0.25
ENSG00000121481	RNF2 PPI subnetwork	0.25
ENSG00000178363	CALML3 PPI subnetwork	0.25
KEGG_APOPTOSIS	KEGG_APOPTOSIS	0.25
MP:0006280	abnormal digit development	0.25
MP:0001306	small lens	0.25
KEGG_T_CELL_RECEPTOR_SIGNALING_PATHWAY	KEGG_T_CELL_RECEPTOR_SIGNALING_PATHWAY	0.25
ENSG00000130939	UBE4B PPI subnetwork	0.25
ENSG00000025796	SEC63 PPI subnetwork	0.25
GO:0001076	RNA polymerase II transcription factor binding transcription factor activity	0.25
MP:0008664	decreased interleukin-12 secretion	0.25
MP:0000229	abnormal megakaryocyte differentiation	0.25
GO:0034364	high-density lipoprotein particle	0.25
GO:0015807	L-amino acid transport	0.25
GO:0043331	response to dsRNA	0.25
ENSG00000102225	CDK16 PPI subnetwork	0.25
MP:0000042	abnormal organ of Corti morphology	0.25
ENSG00000102145	GATA1 PPI subnetwork	0.25
REACTOME_TRANSMEMBRANE_TRANSPORT_OF_SMALL_MOLECULES	REACTOME_TRANSMEMBRANE_TRANSPORT_OF_SMALL_MOLECULES	0.25
MP:0000219	increased neutrophil cell number	0.25
REACTOME_TIGHT_JUNCTION_INTERACTIONS	REACTOME_TIGHT_JUNCTION_INTERACTIONS	0.25
ENSG00000049246	PER3 PPI subnetwork	0.25
ENSG00000012048	BRCA1 PPI subnetwork	0.25
GO:0003006	developmental process involved in reproduction	0.25
ENSG00000188021	UBQLN2 PPI subnetwork	0.25
GO:0010770	positive regulation of cell morphogenesis involved in differentiation	0.25
GO:0006692	prostanoid metabolic process	0.25
MP:0006362	abnormal male germ cell morphology	0.25
ENSG00000067533	RRP15 PPI subnetwork	0.25
GO:0010876	lipid localization	0.25
GO:0030864	cortical actin cytoskeleton	0.25
REACTOME_COPI_MEDIATED_TRANSPORT	REACTOME_COPI_MEDIATED_TRANSPORT	0.25
REACTOME_GOLGI_TO_ER_RETROGRADE_TRANSPORT	REACTOME_GOLGI_TO_ER_RETROGRADE_TRANSPORT	0.25
GO:0050771	negative regulation of axonogenesis	0.25
REACTOME_NONSENSE:MEDIATED_DECAY	REACTOME_NONSENSE:MEDIATED_DECAY	0.25
REACTOME_NONSENSE_MEDIATED_DECAY_ENHANCED_BY_THE_EXON_JUNCT	REACTOME_NONSENSE_MEDIATED_DECAY_ENHANCED_BY_THE_EXON_JUNCTION	0.25
MP:0002100	abnormal tooth morphology	0.25
GO:0009991	response to extracellular stimulus	0.25
MP:0001696	failure to gastrulate	0.25

Original gene set ID	Original gene set description	Nominal P value
GO:0006639	acylglycerol metabolic process	0.25
ENSG00000108061	SHOC2 PPI subnetwork	0.25
ENSG00000145592	RPL37 PPI subnetwork	0.25
ENSG00000197746	PSAP PPI subnetwork	0.25
MP:0011101	partial prenatal lethality	0.25
ENSG00000165271	NOL6 PPI subnetwork	0.25
ENSG00000108262	GIT1 PPI subnetwork	0.25
ENSG00000070759	TESK2 PPI subnetwork	0.25
GO:0007219	Notch signaling pathway	0.25
ENSG00000106799	TGFBR1 PPI subnetwork	0.25
MP:0008082	increased single-positive T cell number	0.25
MP:0008519	thin retinal outer plexiform layer	0.25
GO:0001914	regulation of T cell mediated cytotoxicity	0.25
GO:0055123	digestive system development	0.25
ENSG00000109472	CPE PPI subnetwork	0.25
GO:0004012	phospholipid-translocating ATPase activity	0.25
GO:0006865	amino acid transport	0.25
GO:0042698	ovulation cycle	0.25
ENSG00000126522	ASL PPI subnetwork	0.25
ENSG00000056678	ENSG00000056678 PPI subnetwork	0.25
ENSG00000204197	KIFC1 PPI subnetwork	0.25
GO:0014855	striated muscle cell proliferation	0.25
GO:0022627	cytosolic small ribosomal subunit	0.25
ENSG00000205571	SMN2 PPI subnetwork	0.25
ENSG00000172062	SMN1 PPI subnetwork	0.25
ENSG00000168811	IL12A PPI subnetwork	0.25
ENSG00000118181	RPS25 PPI subnetwork	0.25
GO:0005901	caveola	0.25
GO:0060419	heart growth	0.25
GO:0071436	sodium ion export	0.25
MP:0003935	abnormal craniofacial development	0.25
ENSG00000105953	OGDH PPI subnetwork	0.25
REACTOME_SPHINGOLIPID_METABOLISM	REACTOME_SPHINGOLIPID_METABOLISM	0.25
ENSG00000177301	KCNA2 PPI subnetwork	0.25
MP:0008070	absent T cells	0.25
ENSG00000140284	SLC27A2 PPI subnetwork	0.25
GO:0001917	photoreceptor inner segment	0.25
ENSG00000089159	PXN PPI subnetwork	0.25
MP:0005236	abnormal olfactory nerve morphology	0.26
GO:0042626	ATPase activity, coupled to transmembrane movement of substances	0.26
GO:0019058	viral infectious cycle	0.26
GO:0008340	determination of adult lifespan	0.26
GO:0043543	protein acylation	0.26
MP:0000607	abnormal hepatocyte morphology	0.26
GO:0004867	serine-type endopeptidase inhibitor activity	0.26
ENSG00000135372	NAT10 PPI subnetwork	0.26
KEGG_BIOSYNTHESIS_OF_UNSATURATED_FATTY_ACIDS	KEGG_BIOSYNTHESIS_OF_UNSATURATED_FATTY_ACIDS	0.26
GO:0015749	monosaccharide transport	0.26
ENSG00000101773	RBBP8 PPI subnetwork	0.26

Original gene set ID	Original gene set description	Nominal P value
ENSG00000082146	STRADB PPI subnetwork	0.26
ENSG00000137713	PPP2R1B PPI subnetwork	0.26
ENSG00000117133	RPF1 PPI subnetwork	0.26
GO:0016581	NuRD complex	0.26
GO:0048705	skeletal system morphogenesis	0.26
REACTOME_SIGNALING_BY_NOTCH	REACTOME_SIGNALING_BY_NOTCH	0.26
ENSG00000116473	RAP1A PPI subnetwork	0.26
ENSG00000174791	RIN1 PPI subnetwork	0.26
GO:0045940	positive regulation of steroid metabolic process	0.26
MP:0002026	leukemia	0.26
GO:0048200	Golgi transport vesicle coating	0.26
GO:0035964	COPI-coated vesicle budding	0.26
GO:0048205	COPI coating of Golgi vesicle	0.26
MP:0008537	increased susceptibility to induced colitis	0.26
GO:0042157	lipoprotein metabolic process	0.26
MP:0000596	abnormal liver development	0.26
REACTOME_MITOCHONDRIAL_TRNA_AMINOACYLATION	REACTOME_MITOCHONDRIAL_TRNA_AMINOACYLATION	0.26
REACTOME_FATTY_ACID_TRIACYLGLYCEROL_AND_KETONE_BODY_METABOLISI	REACTOME_FATTY_ACID_TRIACYLGLYCEROL_AND_KETONE_BODY_METABOLISM	0.26
ENSG00000136383	ALPK3 PPI subnetwork	0.26
ENSG00000121274	PAPD5 PPI subnetwork	0.26
ENSG00000115170	ACVR1 PPI subnetwork	0.26
ENSG00000128731	HERC2 PPI subnetwork	0.26
MP:0011086	partial postnatal lethality	0.26
GO:0004725	protein tyrosine phosphatase activity	0.26
MP:0000091	short premaxilla	0.26
ENSG00000069399	BCL3 PPI subnetwork	0.26
MP:0002980	abnormal postural reflex	0.26
ENSG00000164404	GDF9 PPI subnetwork	0.26
GO:0008376	acetylgalactosaminyltransferase activity	0.26
GO:0005247	voltage-gated chloride channel activity	0.26
ENSG00000166025	AMOTL1 PPI subnetwork	0.26
ENSG00000108821	COL1A1 PPI subnetwork	0.26
ENSG00000078304	PPP2R5C PPI subnetwork	0.26
ENSG00000103502	CDIPT PPI subnetwork	0.26
GO:0045321	leukocyte activation	0.26
GO:0005614	interstitial matrix	0.26
ENSG00000196549	MME PPI subnetwork	0.26
ENSG00000124788	ATXN1 PPI subnetwork	0.26
ENSG00000166603	MC4R PPI subnetwork	0.26
REACTOME_ADAPTIVE_IMMUNE_SYSTEM	REACTOME_ADAPTIVE_IMMUNE_SYSTEM	0.26
ENSG00000120690	ELF1 PPI subnetwork	0.26
MP:0005036	diarrhea	0.26
ENSG00000198785	GRIN3A PPI subnetwork	0.26
ENSG00000162736	NCSTN PPI subnetwork	0.26
GO:0030173	integral to Golgi membrane	0.26
MP:0010402	ventricular septal defect	0.26
GO:0007229	integrin-mediated signaling pathway	0.26
GO:0006700	C21-steroid hormone biosynthetic process	0.26
ENSG00000106366	SERPINE1 PPI subnetwork	0.26

Original gene set ID	Original gene set description	Nominal P value
MP:0009230	abnormal sperm head morphology	0.26
ENSG00000091127	PUS7 PPI subnetwork	0.26
ENSG00000065427	KARS PPI subnetwork	0.26
GO:0005057	receptor signaling protein activity	0.26
ENSG00000128050	PAICS PPI subnetwork	0.26
ENSG00000198242	RPL23A PPI subnetwork	0.26
ENSG00000075886	TUBA3D PPI subnetwork	0.26
ENSG00000198033	TUBA3C PPI subnetwork	0.26
GO:0006473	protein acetylation	0.26
ENSG00000186832	KRT16 PPI subnetwork	0.26
KEGG_JAK_STAT_SIGNALING_PATHWAY	KEGG_JAK_STAT_SIGNALING_PATHWAY	0.26
ENSG00000108312	UBTF PPI subnetwork	0.26
MP:0002874	decreased hemoglobin content	0.26
MP:0000467	abnormal esophagus morphology	0.26
ENSG00000108510	MED13 PPI subnetwork	0.26
MP:0008476	increased spleen red pulp amount	0.26
MP:0008525	decreased cranium height	0.26
ENSG00000101421	CHMP4B PPI subnetwork	0.26
MP:0004794	increased anti-nuclear antigen antibody leve	0.26
ENSG00000166233	ARIH1 PPI subnetwork	0.26
ENSG00000116044	NFE2L2 PPI subnetwork	0.26
GO:0004859	phospholipase inhibitor activity	0.26
ENSG00000103089	FA2H PPI subnetwork	0.26
MP:0003717	pallor	0.26
GO:0015149	hexose transmembrane transporter activity	0.26
ENSG00000205609	EIF3CL PPI subnetwork	0.26
GO:0034062	RNA polymerase activity	0.26
GO:0003899	DNA-directed RNA polymerase activity	0.26
MP:0008499	increased IgG1 level	0.26
ENSG00000105141	CASP14 PPI subnetwork	0.26
ENSG00000100934	SEC23A PPI subnetwork	0.26
ENSG00000054267	ARID4B PPI subnetwork	0.26
GO:0034367	macromolecular complex remodeling	0.26
GO:0034368	protein-lipid complex remodeling	0.26
GO:0034369	plasma lipoprotein particle remodeling	0.26
ENSG00000099985	OSM PPI subnetwork	0.26
GO:0033764	steroid dehydrogenase activity, acting on the CH-OH group of donors, NAD or NADP	0.26
ENSG00000186831	ENSG00000186831 PPI subnetwork	0.26
ENSG00000188763	FZD9 PPI subnetwork	0.26
REACTOME_INNATE_IMMUNE_SYSTEM	REACTOME_INNATE_IMMUNE_SYSTEM	0.26
ENSG00000128245	YWHAH PPI subnetwork	0.26
REACTOME_NUCLEAR_EVENTS_KINASE_AND_TRANSCRIPTION_FACTOR_ACTIV/	REACTOME_NUCLEAR_EVENTS_KINASE_AND_TRANSCRIPTION_FACTOR_ACTIVATIC	0.26
MP:0002344	abnormal lymph node B cell domain morphology	0.26
ENSG00000111652	COPS7A PPI subnetwork	0.26
ENSG00000155760	FZD7 PPI subnetwork	0.26
GO:0002218	activation of innate immune response	0.26
MP:0000550	abnormal forelimb morphology	0.26
MP:0001272	increased metastatic potential	0.26
ENSG00000186716	BCR PPI subnetwork	0.26

Original gene set ID	Original gene set description	Nominal P value
GO:0031295	T cell costimulation	0.26
GO:0031294	lymphocyte costimulation	0.26
MP:0008101	lymph node hypoplasia	0.26
GO:0046332	SMAD binding	0.26
GO:0009897	external side of plasma membrane	0.26
MP:0004787	abnormal dorsal aorta morphology	0.26
GO:0000080	G1 phase of mitotic cell cycle	0.26
ENSG00000155508	CNOT8 PPI subnetwork	0.27
GO:0001738	morphogenesis of a polarized epithelium	0.27
KEGG_RIG_I_LIKE_RECEPTOR_SIGNALING_PATHWAY	KEGG_RIG_I_LIKE_RECEPTOR_SIGNALING_PATHWAY	0.27
GO:0007164	establishment of tissue polarity	0.27
GO:0032592	integral to mitochondrial membrane	0.27
ENSG00000107404	DVL1 PPI subnetwork	0.27
REACTOME_FORMATION_OF_FIBRIN_CLOT_CLOTTING_CASCADE	REACTOME_FORMATION_OF_FIBRIN_CLOT_CLOTTING_CASCADE	0.27
GO:0043244	regulation of protein complex disassembly	0.27
ENSG00000162337	LRP5 PPI subnetwork	0.27
ENSG00000054611	TBC1D22A PPI subnetwork	0.27
ENSG00000189369	GSPT2 PPI subnetwork	0.27
ENSG00000007264	MATK PPI subnetwork	0.27
GO:0043300	regulation of leukocyte degranulation	0.27
REACTOME_COMMON_PATHWAY	REACTOME_COMMON_PATHWAY	0.27
GO:0021533	cell differentiation in hindbrain	0.27
ENSG00000066032	CTNNA2 PPI subnetwork	0.27
ENSG00000119041	GTF3C3 PPI subnetwork	0.27
ENSG00000090863	GLG1 PPI subnetwork	0.27
GO:0002688	regulation of leukocyte chemotaxis	0.27
ENSG00000121892	PDS5A PPI subnetwork	0.27
ENSG00000177971	IMP3 PPI subnetwork	0.27
ENSG00000096696	DSP PPI subnetwork	0.27
GO:0032947	protein complex scaffold	0.27
GO:0005072	transforming growth factor beta receptor, cytoplasmic mediator activity	0.27
ENSG00000072694	FCGR2B PPI subnetwork	0.27
GO:0045111	intermediate filament cytoskeleton	0.27
ENSG00000167768	KRT1 PPI subnetwork	0.27
GO:0004896	cytokine receptor activity	0.27
ENSG00000126883	NUP214 PPI subnetwork	0.27
ENSG00000055044	NOP58 PPI subnetwork	0.27
ENSG00000196776	CD47 PPI subnetwork	0.27
GO:0006890	retrograde vesicle-mediated transport, Golgi to ER	0.27
GO:0045933	positive regulation of muscle contraction	0.27
GO:0002366	leukocyte activation involved in immune response	0.27
GO:0002263	cell activation involved in immune response	0.27
ENSG00000087903	RFX2 PPI subnetwork	0.27
GO:0016289	CoA hydrolase activity	0.27
ENSG00000164342	TLR3 PPI subnetwork	0.27
GO:0008168	methyltransferase activity	0.27
ENSG00000159479	MED8 PPI subnetwork	0.27
KEGG_GLYCOSAMINOGLYCAN_BIOSYNTHESIS_CHONDROITIN_SULFATE	KEGG_GLYCOSAMINOGLYCAN_BIOSYNTHESIS_CHONDROITIN_SULFATE	0.27
GO:0032925	regulation of activin receptor signaling pathway	0.27

Original gene set ID	Original gene set description	Nominal P value
MP:0001152	Leydig cell hyperplasia	0.27
ENSG00000113712	CSNK1A1 PPI subnetwork	0.27
ENSG00000125508	SRMS PPI subnetwork	0.27
ENSG00000060140	STYK1 PPI subnetwork	0.27
GO:0007596	blood coagulation	0.27
GO:0045638	negative regulation of myeloid cell differentiatior	0.27
ENSG00000106355	LSM5 PPI subnetwork	0.27
GO:0045089	positive regulation of innate immune response	0.27
ENSG00000132155	RAF1 PPI subnetwork	0.27
GO:0048635	negative regulation of muscle organ development	0.27
ENSG00000119689	DLST PPI subnetwork	0.27
GO:0016493	C-C chemokine receptor activity	0.27
ENSG00000105993	DNAJB6 PPI subnetwork	0.27
GO:0005112	Notch binding	0.27
MP:0003135	increased erythroid progenitor cell number	0.27
REACTOME_RECYCLING_PATHWAY_OF_L1	REACTOME_RECYCLING_PATHWAY_OF_L1	0.27
GO:0071565	nBAF complex	0.27
ENSG00000186111	PIP5K1C PPI subnetwork	0.27
ENSG00000106344	RBM28 PPI subnetwork	0.27
GO:0009168	purine ribonucleoside monophosphate biosynthetic process	0.27
GO:0009127	purine nucleoside monophosphate biosynthetic process	0.27
GO:0050817	coagulation	0.27
GO:0042310	vasoconstriction	0.27
MP:0000854	abnormal cerebellum development	0.27
ENSG00000111731	KIAA0528 PPI subnetwork	0.27
ENSG00000132589	FLOT2 PPI subnetwork	0.27
ENSG00000130669	PAK4 PPI subnetwork	0.27
MP:0008770	decreased survivor rate	0.27
MP:0002796	impaired skin barrier function	0.27
GO:0045445	myoblast differentiation	0.27
GO:0006446	regulation of translational initiation	0.27
GO:0005355	glucose transmembrane transporter activity	0.27
ENSG00000101040	ZMYND8 PPI subnetwork	0.27
GO:0046470	phosphatidylcholine metabolic process	0.27
GO:0045619	regulation of lymphocyte differentiation	0.27
MP:0005309	increased circulating ammonia level	0.27
GO:0070325	lipoprotein particle receptor binding	0.27
GO:0071577	zinc ion transmembrane transport	0.27
ENSG00000105438	KDELR1 PPI subnetwork	0.27
ENSG00000197756	RPL37A PPI subnetwork	0.27
MP:0000026	abnormal inner ear morphology	0.27
GO:0016805	dipeptidase activity	0.27
ENSG00000023191	RNH1 PPI subnetwork	0.27
ENSG00000169682	SPNS1 PPI subnetwork	0.27
ENSG00000165280	VCP PPI subnetwork	0.27
GO:0019199	transmembrane receptor protein kinase activity	0.27
GO:0002683	negative regulation of immune system process	0.27
GO:0007599	hemostasis	0.27
ENSG00000100393	EP300 PPI subnetwork	0.27

Original gene set ID	Original gene set description	Nominal P value
ENSG00000104517	UBR5 PPI subnetwork	0.27
GO:0022829	wide pore channel activity	0.27
ENSG00000092208	GEMIN2 PPI subnetwork	0.27
MP:0008597	decreased circulating interleukin-6 level	0.27
MP:0008395	abnormal osteoblast differentiation	0.27
ENSG00000140319	SRP14 PPI subnetwork	0.27
ENSG00000137818	RPLP1 PPI subnetwork	0.27
ENSG00000198034	RPS4X PPI subnetwork	0.27
REACTOME_NONSENSE_MEDIATED_DECAY_INDEPENDENT_OF_THE_EXON_JUN	REACTOME_NONSENSE_MEDIATED_DECAY_INDEPENDENT_OF_THE_EXON_JUNCTI	0.27
MP:0004952	increased spleen weight	0.27
GO:0009161	ribonucleoside monophosphate metabolic process	0.27
GO:0006367	transcription initiation from RNA polymerase II promoter	0.27
REACTOME_TRAF6_MEDIATED_INDUCTION_OF_NFKB_AND_MAP_KINASES_UP	REACTOME_TRAF6_MEDIATED_INDUCTION_OF_NFKB_AND_MAP_KINASES_UPON_	0.27
REACTOME_TOLL_LIKE_RECEPTOR_78_TLR78_CASCADE	REACTOME_TOLL_LIKE_RECEPTOR_78_TLR78_CASCADE	0.27
REACTOME_MYD88_DEPENDENT_CASCADE_INITIATED_ON_ENDOSOME	REACTOME_MYD88_DEPENDENT_CASCADE_INITIATED_ON_ENDOSOME	0.27
GO:0019208	phosphatase regulator activity	0.27
MP:0003077	abnormal cell cycle	0.27
ENSG00000139626	ITGB7 PPI subnetwork	0.27
ENSG00000184787	UBE2G2 PPI subnetwork	0.27
GO:0030949	positive regulation of vascular endothelial growth factor receptor signaling pathway	0.27
MP:0001533	abnormal skeleton physiology	0.27
MP:0002075	abnormal coat/hair pigmentation	0.27
ENSG00000145912	NHP2 PPI subnetwork	0.27
GO:0055010	ventricular cardiac muscle tissue morphogenesis	0.27
GO:0006814	sodium ion transport	0.27
ENSG00000131368	MRPS25 PPI subnetwork	0.27
GO:0045120	pronucleus	0.27
MP:0005092	decreased double-positive T cell number	0.27
ENSG00000078808	SDF4 PPI subnetwork	0.27
ENSG00000198873	GRK5 PPI subnetwork	0.27
ENSG00000160584	SIK3 PPI subnetwork	0.27
KEGG_PYRUVATE_METABOLISM	KEGG_PYRUVATE_METABOLISM	0.27
MP:0003068	enlarged kidney	0.27
GO:0051591	response to cAMP	0.27
ENSG00000116030	SUMO1 PPI subnetwork	0.27
GO:0031012	extracellular matrix	0.27
ENSG00000150990	DHX37 PPI subnetwork	0.27
ENSG00000075618	FSCN1 PPI subnetwork	0.27
GO:0045814	negative regulation of gene expression, epigenetic	0.27
GO:0015893	drug transport	0.27
ENSG00000115705	TPO PPI subnetwork	0.27
ENSG00000185920	PTCH1 PPI subnetwork	0.27
ENSG00000115221	ITGB6 PPI subnetwork	0.28
GO:0007422	peripheral nervous system development	0.28
ENSG00000031698	SARS PPI subnetwork	0.28
MP:0003270	intestinal obstruction	0.28
GO:0048534	hemopoietic or lymphoid organ development	0.28
ENSG00000150760	DOCK1 PPI subnetwork	0.28
GO:0016741	transferase activity, transferring one-carbon groups	0.28

Original gene set ID	Original gene set description	Nominal P value
GO:0042974	retinoic acid receptor binding	0.28
MP:0001259	abnormal body weight	0.28
MP:0004993	decreased bone resorption	0.28
GO:0000228	nuclear chromosome	0.28
MP:0008481	increased spleen germinal center number	0.28
GO:0007431	salivary gland development	0.28
ENSG00000198554	WDHD1 PPI subnetwork	0.28
ENSG00000100324	TAB1 PPI subnetwork	0.28
ENSG00000160469	BRSK1 PPI subnetwork	0.28
GO:0045668	negative regulation of osteoblast differentiation	0.28
MP:0008174	decreased follicular B cell number	0.28
ENSG00000043462	LCP2 PPI subnetwork	0.28
ENSG00000188153	COL4A5 PPI subnetwork	0.28
ENSG00000067057	PFKP PPI subnetwork	0.28
GO:0005518	collagen binding	0.28
ENSG00000092847	EIF2C1 PPI subnetwork	0.28
ENSG00000180210	F2 PPI subnetwork	0.28
ENSG00000163251	FZD5 PPI subnetwork	0.28
ENSG00000078295	ADCY2 PPI subnetwork	0.28
ENSG00000149806	FAU PPI subnetwork	0.28
ENSG00000129460	NGDN PPI subnetwork	0.28
GO:0019083	viral transcription	0.28
GO:0019080	viral genome expression	0.28
ENSG00000118579	MED28 PPI subnetwork	0.28
ENSG00000101412	E2F1 PPI subnetwork	0.28
GO:2000116	regulation of cysteine-type endopeptidase activity	0.28
MP:0006325	impaired hearing	0.28
GO:0070328	triglyceride homeostasis	0.28
GO:0007250	activation of NF-kappaB-inducing kinase activity	0.28
REACTOME_FGFR3_LIGAND_BINDING_AND_ACTIVATION	REACTOME_FGFR3_LIGAND_BINDING_AND_ACTIVATION	0.28
REACTOME_FGFR3C_LIGAND_BINDING_AND_ACTIVATION	REACTOME_FGFR3C_LIGAND_BINDING_AND_ACTIVATION	0.28
ENSG00000007047	MARK4 PPI subnetwork	0.28
ENSG00000112304	ACOT13 PPI subnetwork	0.28
ENSG00000125691	RPL23 PPI subnetwork	0.28
MP:0004800	decreased susceptibility to experimental autoimmune encephalomyelitis	0.28
GO:0052547	regulation of peptidase activity	0.28
ENSG00000107625	DDX50 PPI subnetwork	0.28
MP:0000711	thymus cortex hypoplasia	0.28
GO:0015295	solute:hydrogen symporter activity	0.28
ENSG00000164104	HMGB2 PPI subnetwork	0.28
ENSG00000163902	RPN1 PPI subnetwork	0.28
GO:0008329	pattern recognition receptor activity	0.28
MP:0011098	complete embryonic lethality during organogenesis	0.28
MP:0002389	abnormal Peyer's patch follicle morphology	0.28
MP:0001399	hyperactivity	0.28
KEGG_ADHERENS_JUNCTION	KEGG_ADHERENS_JUNCTION	0.28
MP:0004259	small placenta	0.28
GO:0022618	ribonucleoprotein complex assembly	0.28
MP:0002578	impaired ability to fire action potentials	0.28

Original gene set ID	Original gene set description	Nominal P value
REACTOME_POST:TRANSLATIONAL_MODIFICATION_SYNTHESIS_OF_GPI:ANCHC	REACTOME_POST:TRANSLATIONAL_MODIFICATION_SYNTHESIS_OF_GPI:ANCHOREI	0.28
MP:0000267	abnormal heart development	0.28
ENSG00000205659	LIN52 PPI subnetwork	0.28
ENSG00000136352	NKX2-1 PPI subnetwork	0.28
ENSG00000108848	LUC7L3 PPI subnetwork	0.28
ENSG00000079335	CDC14A PPI subnetwork	0.28
MP:0003628	abnormal leukocyte adhesion	0.28
GO:0061008	hepaticobiliary system development	0.28
GO:0090287	regulation of cellular response to growth factor stimulus	0.28
MP:0006042	increased apoptosis	0.28
MP:0002144	abnormal B cell differentiation	0.28
ENSG00000107863	ARHGAP21 PPI subnetwork	0.28
ENSG00000196924	FLNA PPI subnetwork	0.28
ENSG00000160999	SH2B2 PPI subnetwork	0.28
GO:0051091	positive regulation of sequence-specific DNA binding transcription factor activity	0.28
ENSG00000132424	PNISR PPI subnetwork	0.28
GO:0017171	serine hydrolase activity	0.28
ENSG00000029534	ANK1 PPI subnetwork	0.28
REACTOME_SEMA4D_IN_SEMAPHORIN_SIGNALING	REACTOME_SEMA4D_IN_SEMAPHORIN_SIGNALING	0.28
ENSG00000160633	SAFB PPI subnetwork	0.28
GO:0050870	positive regulation of T cell activation	0.28
ENSG00000196498	NCOR2 PPI subnetwork	0.28
ENSG00000113460	BRIX1 PPI subnetwork	0.28
GO:0002703	regulation of leukocyte mediated immunity	0.28
ENSG00000198625	MDM4 PPI subnetwork	0.28
ENSG00000178585	CTNNBIP1 PPI subnetwork	0.28
GO:0004435	phosphatidylinositol phospholipase C activity	0.28
KEGG_MAPK_SIGNALING_PATHWAY	KEGG_MAPK_SIGNALING_PATHWAY	0.28
GO:0002685	regulation of leukocyte migration	0.28
GO:0052548	regulation of endopeptidase activity	0.28
MP:0004200	decreased fetal size	0.28
GO:0045923	positive regulation of fatty acid metabolic process	0.28
MP:0004830	short incisors	0.28
MP:0002566	abnormal sexual interaction	0.28
GO:0015114	phosphate ion transmembrane transporter activity	0.28
GO:0050777	negative regulation of immune response	0.28
GO:0031589	cell-substrate adhesion	0.28
ENSG00000187778	MCRS1 PPI subnetwork	0.28
ENSG00000065154	OAT PPI subnetwork	0.28
ENSG00000182255	KCNA4 PPI subnetwork	0.28
GO:0005801	cis-Golgi network	0.28
ENSG00000109606	DHX15 PPI subnetwork	0.28
MP:0004773	abnormal bile composition	0.28
MP:0002417	abnormal megakaryocyte morphology	0.28
GO:0007129	synapsis	0.28
GO:0006544	glycine metabolic process	0.28
GO:0000785	chromatin	0.28
ENSG00000145907	G3BP1 PPI subnetwork	0.28
REACTOME_NEGATIVE_REGULATORS_OF_RIG:IMDA5_SIGNALING	REACTOME_NEGATIVE_REGULATORS_OF_RIG:IMDA5_SIGNALING	0.28

Original gene set ID	Original gene set description	Nominal P value
MP:0002411	decreased susceptibility to bacterial infectior	0.28
ENSG00000145414	NAF1 PPI subnetwork	0.28
GO:0014902	myotube differentiation	0.28
ENSG00000115207	GTF3C2 PPI subnetwork	0.28
ENSG00000138768	USO1 PPI subnetwork	0.28
ENSG00000162434	JAK1 PPI subnetwork	0.28
MP:0001672	abnormal embryogenesis/ development	0.28
ENSG00000113525	IL5 PPI subnetwork	0.28
GO:0048010	vascular endothelial growth factor receptor signaling pathway	0.28
ENSG00000125810	CD93 PPI subnetwork	0.28
GO:0017038	protein import	0.28
GO:0006638	neutral lipid metabolic process	0.28
ENSG00000101365	IDH3B PPI subnetwork	0.29
MP:0005441	increased urine calcium level	0.29
ENSG00000164985	PSIP1 PPI subnetwork	0.29
MP:0005423	abnormal somatic nervous system physiology	0.29
MP:0009435	abnormal miniature inhibitory postsynaptic currents	0.29
ENSG00000173545	ZNF622 PPI subnetwork	0.29
ENSG00000139921	TMX1 PPI subnetwork	0.29
GO:0042113	B cell activation	0.29
GO:0010675	regulation of cellular carbohydrate metabolic process	0.29
ENSG00000064547	LPAR2 PPI subnetwork	0.29
GO:0018393	internal peptidyl-lysine acetylation	0.29
GO:0033202	DNA helicase complex	0.29
GO:0031011	Ino80 complex	0.29
ENSG00000167004	PDIA3 PPI subnetwork	0.29
GO:0005097	Rab GTPase activator activity	0.29
REACTOME_TRANSPORT_TO_THE_GOLGI_AND_SUBSEQUENT_MODIFICATION	REACTOME_TRANSPORT_TO_THE_GOLGI_AND_SUBSEQUENT_MODIFICATION	0.29
MP:0004803	increased susceptibility to autoimmune diabetes	0.29
ENSG00000170365	SMAD1 PPI subnetwork	0.29
GO:0008239	dipeptidyl-peptidase activity	0.29
GO:0043574	peroxisomal transport	0.29
GO:0042035	regulation of cytokine biosynthetic process	0.29
GO:0044144	modulation of growth of symbiont involved in interaction with host	0.29
GO:0044126	regulation of growth of symbiont in host	0.29
GO:0044116	growth of symbiont involved in interaction with host	0.29
GO:0044117	growth of symbiont in host	0.29
GO:0044146	negative regulation of growth of symbiont involved in interaction with host	0.29
GO:0044130	negative regulation of growth of symbiont in host	0.29
GO:0044110	growth involved in symbiotic interaction	0.29
ENSG00000163399	ATP1A1 PPI subnetwork	0.29
ENSG00000186868	MAPT PPI subnetwork	0.29
ENSG00000198001	IRAK4 PPI subnetwork	0.29
GO:0070374	positive regulation of ERK1 and ERK2 cascade	0.29
KEGG_TYPE_II_DIABETES_MELLITUS	KEGG_TYPE_II_DIABETES_MELLITUS	0.29
MP:0008554	decreased circulating tumor necrosis factor leve	0.29
GO:0005881	cytoplasmic microtubule	0.29
GO:0006954	inflammatory response	0.29
ENSG00000125740	FOSB PPI subnetwork	0.29

Original gene set ID	Original gene set description	Nominal P value
GO:0033280	response to vitamin D	0.29
REACTOME_RAP1_SIGNALLING	REACTOME_RAP1_SIGNALLING	0.29
GO:0005246	calcium channel regulator activity	0.29
MP:0004423	abnormal squamosal bone morphology	0.29
GO:0006399	tRNA metabolic process	0.29
GO:0019216	regulation of lipid metabolic process	0.29
REACTOME_NUCLEOTIDE:BINDING_DOMAIN_LEUCINE_RICH_REPEAT_CONTAIN	REACTOME_NUCLEOTIDE:BINDING_DOMAIN_LEUCINE_RICH_REPEAT_CONTAINING	0.29
ENSG00000131023	LATS1 PPI subnetwork	0.29
REACTOME_EFFECTS_OF_PIP2_HYDROLYSIS	REACTOME_EFFECTS_OF_PIP2_HYDROLYSIS	0.29
ENSG00000148606	POLR3A PPI subnetwork	0.29
MP:0001314	corneal opacity	0.29
GO:0035914	skeletal muscle cell differentiation	0.29
ENSG00000124383	MPHOSPH10 PPI subnetwork	0.29
GO:0045907	positive regulation of vasoconstriction	0.29
REACTOME_TRIGLYCERIDE_BIOSYNTHESIS	REACTOME_TRIGLYCERIDE_BIOSYNTHESIS	0.29
ENSG00000107882	SUFU PPI subnetwork	0.29
ENSG00000136982	DSCC1 PPI subnetwork	0.29
GO:0043966	histone H3 acetylation	0.29
ENSG00000082397	EPB41L3 PPI subnetwork	0.29
MP:0005013	increased lymphocyte cell number	0.29
GO:0051272	positive regulation of cellular component movement	0.29
GO:0015238	drug transmembrane transporter activity	0.29
ENSG00000206412	GNL1 PPI subnetwork	0.29
ENSG00000204590	GNL1 PPI subnetwork	0.29
ENSG00000206492	GNL1 PPI subnetwork	0.29
REACTOME_VITAMIN_B5_PANTOTHENATE_METABOLISM	REACTOME_VITAMIN_B5_PANTOTHENATE_METABOLISM	0.29
ENSG00000184922	FMNL1 PPI subnetwork	0.29
GO:0005070	SH3/SH2 adaptor activity	0.29
ENSG00000164930	FZD6 PPI subnetwork	0.29
ENSG00000111432	FZD10 PPI subnetwork	0.29
ENSG00000057593	F7 PPI subnetwork	0.29
ENSG00000155966	AFF2 PPI subnetwork	0.29
GO:0016820	hydrolase activity, acting on acid anhydrides, catalyzing transmembrane movement	0.29
GO:0006400	tRNA modification	0.29
GO:0001755	neural crest cell migration	0.29
ENSG00000113263	ITK PPI subnetwork	0.29
GO:0009746	response to hexose stimulus	0.29
GO:0000977	RNA polymerase II regulatory region sequence-specific DNA binding	0.29
GO:0000932	cytoplasmic mRNA processing body	0.29
ENSG00000120705	ETF1 PPI subnetwork	0.29
ENSG00000188536	HBA2 PPI subnetwork	0.29
ENSG00000206172	HBA1 PPI subnetwork	0.29
MP:0001806	decreased IgM level	0.29
MP:0001501	abnormal sleep pattern	0.29
GO:0045087	innate immune response	0.29
MP:0001325	abnormal retina morphology	0.29
ENSG00000145425	RPS3A PPI subnetwork	0.29
ENSG00000138018	EPT1 PPI subnetwork	0.29
ENSG00000166794	PPIB PPI subnetwork	0.29

Original gene set ID	Original gene set description	Nominal P value
ENSG00000134248	HBXIP PPI subnetwork	0.29
ENSG00000130985	UBA1 PPI subnetwork	0.29
ENSG00000117480	FAAH PPI subnetwork	0.29
MP:0002657	chondrodystrophy	0.29
GO:0016791	phosphatase activity	0.29
ENSG00000112964	GHR PPI subnetwork	0.29
ENSG00000107937	GTPBP4 PPI subnetwork	0.29
MP:0000808	abnormal hippocampus development	0.29
MP:0011087	complete neonatal lethality	0.29
MP:0009657	failure of chorioallantoic fusion	0.29
GO:0032102	negative regulation of response to external stimulus	0.29
GO:0048872	homeostasis of number of cells	0.29
GO:0001818	negative regulation of cytokine production	0.29
GO:0016568	chromatin modification	0.29
GO:0006906	vesicle fusion	0.29
GO:0042641	actomyosin	0.29
ENSG00000111641	NOP2 PPI subnetwork	0.29
ENSG00000130810	PPAN PPI subnetwork	0.29
GO:0051648	vesicle localization	0.29
ENSG00000149269	PAK1 PPI subnetwork	0.29
ENSG00000088205	DDX18 PPI subnetwork	0.29
MP:0000875	abnormal cerebellar Purkinje cell layer	0.29
MP:0005597	decreased susceptibility to type I hypersensitivity reaction	0.29
GO:0000979	RNA polymerase II core promoter sequence-specific DNA binding	0.29
ENSG00000141141	DDX52 PPI subnetwork	0.29
REACTOME_TRANSLATION	REACTOME_TRANSLATION	0.29
GO:0043281	regulation of cysteine-type endopeptidase activity involved in apoptotic process	0.29
GO:2000241	regulation of reproductive process	0.29
ENSG00000005022	SLC25A5 PPI subnetwork	0.29
ENSG00000132153	DHX30 PPI subnetwork	0.29
ENSG00000064393	HIPK2 PPI subnetwork	0.29
GO:0043903	regulation of symbiosis, encompassing mutualism through parasitism	0.29
GO:0050892	intestinal absorption	0.29
GO:0006555	methionine metabolic process	0.29
GO:0051656	establishment of organelle localization	0.29
MP:0002652	thin myocardium	0.29
GO:0010718	positive regulation of epithelial to mesenchymal transition	0.29
ENSG00000188404	SELL PPI subnetwork	0.29
MP:0000872	abnormal cerebellum external granule cell layer morphology	0.29
ENSG00000074054	CLASP1 PPI subnetwork	0.29
GO:0017187	peptidyl-glutamic acid carboxylation	0.29
GO:0018214	protein carboxylation	0.29
ENSG00000118523	CTGF PPI subnetwork	0.3
MP:0002207	abnormal long term potentiation	0.3
GO:0005795	Golgi stack	0.3
ENSG00000068793	CYFIP1 PPI subnetwork	0.3
ENSG00000102158	MAGT1 PPI subnetwork	0.3
GO:0030133	transport vesicle	0.3
ENSG00000116544	DLGAP3 PPI subnetwork	0.3

Original gene set ID	Original gene set description	Nominal P value
ENSG00000157873	TNFRSF14 PPI subnetwork	0.3
ENSG00000139219	COL2A1 PPI subnetwork	0.3
ENSG00000104290	FZD3 PPI subnetwork	0.3
GO:0018394	peptidyl-lysine acetylation	0.3
GO:0015293	symporter activity	0.3
ENSG00000125538	IL1B PPI subnetwork	0.3
ENSG00000070785	EIF2B3 PPI subnetwork	0.3
GO:0007052	mitotic spindle organization	0.3
GO:0060090	binding, bridging	0.3
ENSG00000100811	YY1 PPI subnetwork	0.3
GO:0005669	transcription factor TFIID complex	0.3
ENSG00000078269	SYNJ2 PPI subnetwork	0.3
ENSG00000131323	TRAF3 PPI subnetwork	0.3
ENSG00000009830	POMT2 PPI subnetwork	0.3
REACTOME_TRAF6_MEDIATED_INDUCTION_OF_PROINFLAMMATORY_CYTOKIN	REACTOME_TRAF6_MEDIATED_INDUCTION_OF_PROINFLAMMATORY_CYTOKINES	0.3
ENSG00000156453	PCDH1 PPI subnetwork	0.3
ENSG00000134697	GNL2 PPI subnetwork	0.3
MP:0003724	increased susceptibility to induced arthritis	0.3
MP:0005023	abnormal wound healing	0.3
GO:0015298	solute:cation antiporter activity	0.3
MP:0003974	abnormal endocardium morphology	0.3
ENSG00000196415	PRTN3 PPI subnetwork	0.3
GO:0007281	germ cell development	0.3
MP:0011024	abnormal branching involved in lung morphogenesis	0.3
MP:0008451	retinal rod cell degeneration	0.3
GO:0009119	ribonucleoside metabolic process	0.3
ENSG00000130164	LDLR PPI subnetwork	0.3
GO:0071216	cellular response to biotic stimulus	0.3
MP:0008049	increased memory T cell number	0.3
ENSG00000127445	PIN1 PPI subnetwork	0.3
MP:0000125	absent incisors	0.3
ENSG00000171858	RPS21 PPI subnetwork	0.3
ENSG00000113360	DROSHA PPI subnetwork	0.3
ENSG00000151498	ACAD8 PPI subnetwork	0.3
ENSG00000151090	THRB PPI subnetwork	0.3
ENSG00000172602	RND1 PPI subnetwork	0.3
ENSG00000170522	ELOVL6 PPI subnetwork	0.3
GO:0005978	glycogen biosynthetic process	0.3
GO:0009250	glucan biosynthetic process	0.3
MP:0001881	abnormal mammary gland physiology	0.3
MP:0000280	thin ventricular wall	0.3
ENSG00000142676	RPL11 PPI subnetwork	0.3
GO:0031526	brush border membrane	0.3
GO:0045785	positive regulation of cell adhesion	0.3
GO:0005929	cilium	0.3
GO:0005996	monosaccharide metabolic process	0.3
GO:0015491	cation:cation antiporter activity	0.3
ENSG00000141076	CIRH1A PPI subnetwork	0.3
ENSG00000169957	ZNF768 PPI subnetwork	0.3

Original gene set ID	Original gene set description	Nominal P value
ENSG00000074201	CLNS1A PPI subnetwork	0.3
ENSG00000112242	E2F3 PPI subnetwork	0.3
REACTOME_MYD88_CASCADE_INITIATED_ON_PLASMA_MEMBRANE	REACTOME_MYD88_CASCADE_INITIATED_ON_PLASMA_MEMBRANE	0.3
REACTOME_TOLL_LIKE_RECEPTOR_10_TLR10_CASCADE	REACTOME_TOLL_LIKE_RECEPTOR_10_TLR10_CASCADE	0.3
REACTOME_TOLL_LIKE_RECEPTOR_5_TLR5_CASCADE	REACTOME_TOLL_LIKE_RECEPTOR_5_TLR5_CASCADE	0.3
GO:0004143	diacylglycerol kinase activity	0.3
GO:0050852	T cell receptor signaling pathway	0.3
GO:0051270	regulation of cellular component movement	0.3
ENSG00000166908	PIP4K2C PPI subnetwork	0.3
MP:0002460	decreased immunoglobulin level	0.3
GO:0007507	heart development	0.3
GO:0007265	Ras protein signal transduction	0.3
ENSG00000187391	MAGI2 PPI subnetwork	0.3
ENSG00000178913	TAF7 PPI subnetwork	0.3
REACTOME_TRNA_AMINOACYLATION	REACTOME_TRNA_AMINOACYLATION	0.3
MP:0005292	improved glucose tolerance	0.3
REACTOME_TOLL_LIKE_RECEPTOR_4_TLR4_CASCADE	REACTOME_TOLL_LIKE_RECEPTOR_4_TLR4_CASCADE	0.3
GO:0045773	positive regulation of axon extension	0.3
GO:0045637	regulation of myeloid cell differentiation	0.3
ENSG00000132507	EIF5A PPI subnetwork	0.3
GO:0004521	endoribonuclease activity	0.3
MP:0008040	decreased NK T cell number	0.3
MP:0002463	abnormal neutrophil physiology	0.3
GO:0032432	actin filament bundle	0.3
ENSG00000136936	XPA PPI subnetwork	0.3
REACTOME_TOLL_RECEPTOR_CASCADES	REACTOME_TOLL_RECEPTOR_CASCADES	0.3
ENSG00000182718	ANXA2 PPI subnetwork	0.3
KEGG_GLYCOSAMINOGLYCAN_BIOSYNTHESIS_HEPARAN_SULFATE	KEGG_GLYCOSAMINOGLYCAN_BIOSYNTHESIS_HEPARAN_SULFATE	0.3
GO:0008320	protein transmembrane transporter activity	0.3
GO:0022884	macromolecule transmembrane transporter activity	0.3
ENSG00000152147	GEMIN6 PPI subnetwork	0.3
GO:0055024	regulation of cardiac muscle tissue development	0.3
GO:0008637	apoptotic mitochondrial changes	0.3
ENSG00000108296	CWC25 PPI subnetwork	0.3
GO:0070227	lymphocyte apoptotic process	0.3
GO:0002675	positive regulation of acute inflammatory response	0.3
GO:0001104	RNA polymerase II transcription cofactor activity	0.3
ENSG00000196781	TLE1 PPI subnetwork	0.3
ENSG00000177105	RHOG PPI subnetwork	0.3
GO:0006109	regulation of carbohydrate metabolic process	0.3
ENSG00000120071	KIAA1267 PPI subnetwork	0.3
ENSG00000106804	C5 PPI subnetwork	0.3
GO:0008373	sialyltransferase activity	0.3
ENSG00000187109	NAP1L1 PPI subnetwork	0.3
GO:0090329	regulation of DNA-dependent DNA replication	0.3
ENSG00000198742	SMURF1 PPI subnetwork	0.3
GO:0045646	regulation of erythrocyte differentiation	0.3
GO:0032655	regulation of interleukin-12 production	0.3
ENSG00000204319	ENSG00000204319 PPI subnetwork	0.3

Original gene set ID	Original gene set description	Nominal P value
MP:0002945	abnormal inhibitory postsynaptic currents	0.3
ENSG00000161939	C17orf49 PPI subnetwork	0.3
MP:0009403	increased variability of skeletal muscle fiber size	0.3
GO:0005372	water transmembrane transporter activity	0.3
GO:0031099	regeneration	0.3
ENSG00000196405	EVL PPI subnetwork	0.3
ENSG00000105216	ENSG00000105216 PPI subnetwork	0.3
GO:0072332	signal transduction by p53 class mediator resulting in induction of apoptosis	0.3
ENSG00000086205	FOLH1 PPI subnetwork	0.3
REACTOME_SHC:MEDIATED_CASCADE	REACTOME_SHC:MEDIATED_CASCADE	0.31
GO:0033267	axon part	0.31
GO:0000123	histone acetyltransferase complex	0.31
GO:0031016	pancreas development	0.31
REACTOME_N:GLYCAN_ANTENNAE_ELONGATION_IN_THE_MEDIALTRANS:GOL	REACTOME_N:GLYCAN_ANTENNAE_ELONGATION_IN_THE_MEDIALTRANS:GOLGI	0.31
ENSG00000148296	SURF6 PPI subnetwork	0.31
GO:0051495	positive regulation of cytoskeleton organization	0.31
ENSG00000138448	ITGAV PPI subnetwork	0.31
ENSG00000137497	NUMA1 PPI subnetwork	0.31
GO:0004721	phosphoprotein phosphatase activity	0.31
MP:0002743	glomerulonephritis	0.31
MP:0010024	increased total body fat amount	0.31
ENSG00000135903	PAX3 PPI subnetwork	0.31
MP:0002625	heart left ventricle hypertrophy	0.31
MP:0003089	decreased skin tensile strength	0.31
ENSG00000196591	HDAC2 PPI subnetwork	0.31
GO:0043086	negative regulation of catalytic activity	0.31
ENSG00000127564	PKMYT1 PPI subnetwork	0.31
GO:0032313	regulation of Rab GTPase activity	0.31
GO:0032483	regulation of Rab protein signal transduction	0.31
GO:0016775	phosphotransferase activity, nitrogenous group as acceptor	0.31
ENSG00000138594	TMOD3 PPI subnetwork	0.31
ENSG00000132170	PPARG PPI subnetwork	0.31
MP:0002092	abnormal eye morphology	0.31
GO:0045060	negative thymic T cell selection	0.31
ENSG00000124641	MED20 PPI subnetwork	0.31
GO:0043038	amino acid activation	0.31
GO:0043039	tRNA aminoacylation	0.31
ENSG00000137275	RIPK1 PPI subnetwork	0.31
ENSG00000171681	ATF7IP PPI subnetwork	0.31
ENSG00000136238	RAC1 PPI subnetwork	0.31
GO:0017124	SH3 domain binding	0.31
GO:0003073	regulation of systemic arterial blood pressure	0.31
MP:0004157	interrupted aortic arch	0.31
ENSG00000181856	SLC2A4 PPI subnetwork	0.31
ENSG00000162702	ZNF281 PPI subnetwork	0.31
GO:0005732	small nucleolar ribonucleoprotein complex	0.31
MP:0005215	abnormal pancreatic islet morphology	0.31
ENSG00000063177	RPL18 PPI subnetwork	0.31
ENSG00000011260	UTP18 PPI subnetwork	0.31

Original gene set ID	Original gene set description	Nominal P value
GO:0006606	protein import into nucleus	0.31
MP:0002145	abnormal T cell differentiation	0.31
GO:0051427	hormone receptor binding	0.31
ENSG00000173369	C1QB PPI subnetwork	0.31
ENSG00000167193	CRK PPI subnetwork	0.31
GO:0017148	negative regulation of translation	0.31
GO:0030291	protein serine/threonine kinase inhibitor activity	0.31
ENSG00000141378	PTRH2 PPI subnetwork	0.31
GO:0031056	regulation of histone modification	0.31
REACTOME_CYCLIN_D_ASSOCIATED_EVENTS_IN_G1	REACTOME_CYCLIN_D_ASSOCIATED_EVENTS_IN_G1	0.31
REACTOME_G1_PHASE	REACTOME_G1_PHASE	0.31
GO:0043601	nuclear replisome	0.31
GO:0030894	replisome	0.31
ENSG00000163605	PPP4R2 PPI subnetwork	0.31
ENSG00000049323	LTBP1 PPI subnetwork	0.31
GO:0008285	negative regulation of cell proliferation	0.31
MP:0001146	abnormal testis morphology	0.31
ENSG00000147689	FAM83A PPI subnetwork	0.31
ENSG00000004660	CAMKK1 PPI subnetwork	0.31
REACTOME_BILE_SALT_AND_ORGANIC_ANION_SLC_TRANSPORTERS	REACTOME_BILE_SALT_AND_ORGANIC_ANION_SLC_TRANSPORTERS	0.31
GO:0021872	forebrain generation of neurons	0.31
ENSG00000112851	ERBB2IP PPI subnetwork	0.31
GO:0048520	positive regulation of behavior	0.31
REACTOME_TOLL_LIKE_RECEPTOR_2_TLR2_CASCADE	REACTOME_TOLL_LIKE_RECEPTOR_2_TLR2_CASCADE	0.31
REACTOME_TOLL_LIKE_RECEPTOR_TLR6TLR2_CASCADE	REACTOME_TOLL_LIKE_RECEPTOR_TLR6TLR2_CASCADE	0.31
REACTOME_MYD88MAL_CASCADE_INITIATED_ON_PLASMA_MEMBRANE	REACTOME_MYD88MAL_CASCADE_INITIATED_ON_PLASMA_MEMBRANE	0.31
REACTOME_TOLL_LIKE_RECEPTOR_TLR1TLR2_CASCADE	REACTOME_TOLL_LIKE_RECEPTOR_TLR1TLR2_CASCADE	0.31
GO:0030673	axolemma	0.31
GO:0030097	hemopoiesis	0.31
REACTOME_INTERLEUKIN:3_5_AND_GM:CSF_SIGNALING	REACTOME_INTERLEUKIN:3_5_AND_GM:CSF_SIGNALING	0.31
GO:0006312	mitotic recombination	0.31
ENSG00000085117	CD82 PPI subnetwork	0.31
GO:0048742	regulation of skeletal muscle fiber development	0.31
GO:0043303	mast cell degranulation	0.31
ENSG00000173281	PPP1R3B PPI subnetwork	0.31
REACTOME_SRP:DEPENDENT_COTRANSLATIONAL_PROTEIN_TARGETING_TO_M	REACTOME_SRP:DEPENDENT_COTRANSLATIONAL_PROTEIN_TARGETING_TO_MEM	0.31
ENSG00000161956	SENP3 PPI subnetwork	0.31
ENSG00000102753	KPNA3 PPI subnetwork	0.31
GO:0048641	regulation of skeletal muscle tissue development	0.31
GO:0005882	intermediate filament	0.31
ENSG00000164442	CITED2 PPI subnetwork	0.31
MP:0000239	absent common myeloid progenitor cells	0.31
ENSG00000212802	ENSG00000212802 PPI subnetwork	0.31
ENSG00000174766	ENSG00000174766 PPI subnetwork	0.31
MP:0002114	abnormal axial skeleton morphology	0.31
ENSG00000198933	TBKBP1 PPI subnetwork	0.31
ENSG00000129255	MPDU1 PPI subnetwork	0.31
GO:0006720	isoprenoid metabolic process	0.31
ENSG00000204271	SPIN3 PPI subnetwork	0.31

Original gene set ID	Original gene set description	Nominal P value
ENSG00000186787	SPIN2B PPI subnetwork	0.31
MP:0004816	abnormal class switch recombination	0.31
MP:0002410	decreased susceptibility to viral infection	0.31
ENSG00000183093	ENSG00000183093 PPI subnetwork	0.31
ENSG00000077454	LRCH4 PPI subnetwork	0.31
ENSG00000205307	SAP25 PPI subnetwork	0.31
ENSG00000197263	OR8D2 PPI subnetwork	0.31
ENSG00000143514	TP53BP2 PPI subnetwork	0.31
MP:0004028	chromosome breakage	0.31
ENSG00000160208	RRP1B PPI subnetwork	0.31
ENSG00000089693	MLF2 PPI subnetwork	0.31
MP:0001429	dehydration	0.31
GO:0005667	transcription factor complex	0.31
REACTOME_PURINE_RIBONUCLEOSIDE_MONOPHOSPHATE_BIOSYNTHESIS	REACTOME_PURINE_RIBONUCLEOSIDE_MONOPHOSPHATE_BIOSYNTHESIS	0.31
GO:0002757	immune response-activating signal transduction	0.31
MP:0000284	double outlet heart right ventricle	0.31
GO:0007179	transforming growth factor beta receptor signaling pathway	0.31
ENSG00000112936	C7 PPI subnetwork	0.31
MP:0009254	disorganized pancreatic islets	0.31
GO:0001012	RNA polymerase II regulatory region DNA binding	0.31
MP:0004502	decreased incidence of chemically-induced tumors	0.31
GO:0034660	ncRNA metabolic process	0.31
ENSG00000164107	HAND2 PPI subnetwork	0.31
ENSG00000119616	FCF1 PPI subnetwork	0.31
GO:0080135	regulation of cellular response to stress	0.31
GO:0042393	histone binding	0.31
GO:0034284	response to monosaccharide stimulus	0.31
GO:0045095	keratin filament	0.31
ENSG00000104833	TUBB4A PPI subnetwork	0.31
ENSG00000162889	MAPKAPK2 PPI subnetwork	0.31
ENSG00000101665	SMAD7 PPI subnetwork	0.31
GO:0016272	prefoldin complex	0.31
ENSG00000128602	SMO PPI subnetwork	0.31
GO:0007051	spindle organization	0.31
ENSG00000070814	TCOF1 PPI subnetwork	0.31
REACTOME_SIGNAL_REGULATORY_PROTEIN_SIRP_FAMILY_INTERACTIONS	REACTOME_SIGNAL_REGULATORY_PROTEIN_SIRP_FAMILY_INTERACTIONS	0.31
MP:0008008	early cellular replicative senescence	0.31
GO:0060333	interferon-gamma-mediated signaling pathway	0.31
GO:0042249	establishment of planar polarity of embryonic epithelium	0.31
GO:0002822	regulation of adaptive immune response based on somatic recombination of immur	0.31
ENSG00000087088	BAX PPI subnetwork	0.31
ENSG00000172216	CEBPB PPI subnetwork	0.31
MP:0002108	abnormal muscle morphology	0.31
ENSG00000186416	NKRF PPI subnetwork	0.31
GO:0048863	stem cell differentiation	0.31
MP:0008875	abnormal xenobiotic pharmacokinetics	0.32
ENSG00000114767	RRP9 PPI subnetwork	0.32
MP:0004762	increased anti-double stranded DNA antibody leve	0.32
GO:0051321	meiotic cell cycle	0.32

Original gene set ID	Original gene set description	Nominal P value
ENSG00000172594	SMPDL3A PPI subnetwork	0.32
MP:0001556	increased circulating HDL cholesterol leve	0.32
ENSG00000099882	ENSG00000099882 PPI subnetwork	0.32
ENSG00000179632	MAF1 PPI subnetwork	0.32
GO:0005275	amine transmembrane transporter activity	0.32
GO:0051249	regulation of lymphocyte activation	0.32
MP:0004613	fusion of vertebral arches	0.32
GO:0006470	protein dephosphorylation	0.32
GO:0009071	serine family amino acid catabolic process	0.32
ENSG00000157240	FZD1 PPI subnetwork	0.32
GO:0090177	establishment of planar polarity involved in neural tube closure	0.32
ENSG00000114784	EIF1B PPI subnetwork	0.32
GO:0070372	regulation of ERK1 and ERK2 cascade	0.32
ENSG00000124151	NCOA3 PPI subnetwork	0.32
ENSG00000185883	ATP6V0C PPI subnetwork	0.32
GO:0000794	condensed nuclear chromosome	0.32
MP:0000471	abnormal stomach epithelium morphology	0.32
MP:0000418	focal hair loss	0.32
ENSG00000175220	ARHGAP1 PPI subnetwork	0.32
GO:0010721	negative regulation of cell development	0.32
MP:0004401	increased cochlear outer hair cell number	0.32
GO:0030128	clathrin coat of endocytic vesicle	0.32
MP:0004946	abnormal regulatory T cell physiology	0.32
MP:0004768	abnormal axonal transport	0.32
REACTOME_PROTEIN_FOLDING	REACTOME_PROTEIN_FOLDING	0.32
ENSG00000112208	BAG2 PPI subnetwork	0.32
REACTOME_TCR_SIGNALING	REACTOME_TCR_SIGNALING	0.32
REACTOME_TRANSLATION_INITIATION_COMPLEX_FORMATION	REACTOME_TRANSLATION_INITIATION_COMPLEX_FORMATION	0.32
REACTOME_DEADENYLATION_OF_MRNA	REACTOME_DEADENYLATION_OF_MRNA	0.32
REACTOME_THE_NLRP3_INFLAMMASOME	REACTOME_THE_NLRP3_INFLAMMASOME	0.32
ENSG00000127824	TUBA4A PPI subnetwork	0.32
GO:0001889	liver development	0.32
REACTOME_TOLL_LIKE_RECEPTOR_9_TLR9_CASCADE	REACTOME_TOLL_LIKE_RECEPTOR_9_TLR9_CASCADE	0.32
ENSG00000159063	ALG8 PPI subnetwork	0.32
MP:0011260	abnormal head mesenchyme morphology	0.32
GO:0045843	negative regulation of striated muscle tissue development	0.32
GO:0006418	tRNA aminoacylation for protein translation	0.32
GO:0030835	negative regulation of actin filament depolymerization	0.32
MP:0011085	complete postnatal lethality	0.32
ENSG00000100029	PES1 PPI subnetwork	0.32
ENSG00000149273	RPS3 PPI subnetwork	0.32
GO:0050715	positive regulation of cytokine secretion	0.32
GO:0015629	actin cytoskeleton	0.32
KEGG_CYTOSOLIC_DNA_SENSING_PATHWAY	KEGG_CYTOSOLIC_DNA_SENSING_PATHWAY	0.32
ENSG00000173530	TNFRSF10D PPI subnetwork	0.32
ENSG00000106546	AHR PPI subnetwork	0.32
ENSG00000152944	MED21 PPI subnetwork	0.32
ENSG00000133398	MED10 PPI subnetwork	0.32
GO:0007019	microtubule depolymerization	0.32

Original gene set ID	Original gene set description	Nominal P value
MP:0004173	abnormal intervertebral disk morphology	0.32
ENSG00000125084	WNT1 PPI subnetwork	0.32
GO:0003215	cardiac right ventricle morphogenesis	0.32
ENSG00000149968	MMP3 PPI subnetwork	0.32
ENSG00000099389	ENSG00000099389 PPI subnetwork	0.32
ENSG00000118972	FGF23 PPI subnetwork	0.32
ENSG00000162521	RBBP4 PPI subnetwork	0.32
ENSG00000113916	BCL6 PPI subnetwork	0.32
ENSG00000037280	FLT4 PPI subnetwork	0.32
ENSG00000103653	CSK PPI subnetwork	0.32
ENSG00000111364	DDX55 PPI subnetwork	0.32
REACTOME_FRS2:MEDIATED_CASCADE	REACTOME_FRS2:MEDIATED_CASCADE	0.32
GO:0031228	intrinsic to Golgi membrane	0.32
MP:0006379	abnormal spermatocyte morphology	0.32
ENSG00000184678	HIST2H2BE PPI subnetwork	0.32
GO:0051240	positive regulation of multicellular organismal process	0.32
GO:0050321	tau-protein kinase activity	0.32
GO:0051635	bacterial cell surface binding	0.32
MP:0000364	abnormal vascular regression	0.32
ENSG00000104980	TIMM44 PPI subnetwork	0.32
GO:0042475	odontogenesis of dentin-containing tooth	0.32
REACTOME_PD:1_SIGNALING	REACTOME_PD:1_SIGNALING	0.32
GO:0006693	prostaglandin metabolic process	0.32
GO:0007259	JAK-STAT cascade	0.32
ENSG00000215301	DDX3X PPI subnetwork	0.32
GO:0016893	endonuclease activity, active with either ribo- or deoxyribonucleic acids and produci	0.32
ENSG00000175390	EIF3F PPI subnetwork	0.32
GO:0008235	metalloexopeptidase activity	0.32
GO:0002764	immune response-regulating signaling pathway	0.32
MP:0002891	increased insulin sensitivity	0.32
GO:0051567	histone H3-K9 methylation	0.32
ENSG00000120158	RCL1 PPI subnetwork	0.32
KEGG_OTHER_GLYCAN_DEGRADATION	KEGG_OTHER_GLYCAN_DEGRADATION	0.32
GO:0051348	negative regulation of transferase activity	0.32
ENSG00000159348	CYB5R1 PPI subnetwork	0.32
GO:0002706	regulation of lymphocyte mediated immunity	0.32
ENSG00000196981	WDR5B PPI subnetwork	0.32
ENSG00000185736	ADARB2 PPI subnetwork	0.32
MP:0010254	nuclear cataracts	0.32
MP:0008078	increased CD8-positive T cell number	0.32
GO:0004629	phospholipase C activity	0.32
ENSG00000099194	SCD PPI subnetwork	0.32
ENSG00000112578	BYSL PPI subnetwork	0.32
ENSG00000164587	RPS14 PPI subnetwork	0.32
ENSG00000136271	DDX56 PPI subnetwork	0.32
REACTOME_ACTIVATION_OF_THE_MRNA_UPON_BINDING_OF_THE_CAP:BINDI	REACTOME_ACTIVATION_OF_THE_MRNA_UPON_BINDING_OF_THE_CAP:BINDING_	0.32
GO:0046620	regulation of organ growth	0.32
GO:0070411	I-SMAD binding	0.32
ENSG00000176165	FOXG1 PPI subnetwork	0.32

Original gene set ID	Original gene set description	Nominal P value
MP:0011096	complete embryonic lethality before somite formation	0.32
MP:0001261	obese	0.32
GO:0052689	carboxylic ester hydrolase activity	0.32
MP:0003355	decreased ovulation rate	0.32
ENSG00000215760	ENSG00000215760 PPI subnetwork	0.32
ENSG00000187325	TAF9B PPI subnetwork	0.32
ENSG00000167670	CHAF1A PPI subnetwork	0.32
GO:0034330	cell junction organization	0.32
ENSG00000151276	MAGI1 PPI subnetwork	0.32
ENSG00000160282	FTCD PPI subnetwork	0.32
ENSG00000084676	NCOA1 PPI subnetwork	0.33
ENSG00000108654	DDX5 PPI subnetwork	0.33
ENSG00000152137	HSPB8 PPI subnetwork	0.33
REACTOME_SIGNALING_BY_NODAL	REACTOME_SIGNALING_BY_NODAL	0.33
ENSG00000163349	HIPK1 PPI subnetwork	0.33
GO:0032637	interleukin-8 production	0.33
GO:0008015	blood circulation	0.33
MP:0005319	abnormal enzyme/ coenzyme level	0.33
ENSG00000111642	CHD4 PPI subnetwork	0.33
GO:0045017	glycerolipid biosynthetic process	0.33
GO:0019207	kinase regulator activity	0.33
ENSG00000073536	NLE1 PPI subnetwork	0.33
ENSG00000138031	ADCY3 PPI subnetwork	0.33
MP:0002940	variable body spotting	0.33
GO:0005578	proteinaceous extracellular matrix	0.33
ENSG00000153563	CD8A PPI subnetwork	0.33
ENSG00000184897	H1FX PPI subnetwork	0.33
GO:0032615	interleukin-12 production	0.33
ENSG00000108590	MED31 PPI subnetwork	0.33
ENSG00000138835	RGS3 PPI subnetwork	0.33
ENSG00000197586	ENTPD6 PPI subnetwork	0.33
GO:0016860	intramolecular oxidoreductase activity	0.33
ENSG00000159217	IGF2BP1 PPI subnetwork	0.33
GO:2000147	positive regulation of cell motility	0.33
MP:0000696	abnormal Peyer's patch morphology	0.33
MP:0000024	lowered ear position	0.33
REACTOME_BETA:CATENIN_PHOSPHORYLATION_CASCADE	REACTOME_BETA:CATENIN_PHOSPHORYLATION_CASCADE	0.33
ENSG00000101017	CD40 PPI subnetwork	0.33
ENSG00000149357	LAMTOR1 PPI subnetwork	0.33
ENSG00000138180	CEP55 PPI subnetwork	0.33
ENSG00000177791	MYOZ1 PPI subnetwork	0.33
GO:0006505	GPI anchor metabolic process	0.33
GO:0043368	positive T cell selection	0.33
GO:0031347	regulation of defense response	0.33
GO:0051170	nuclear import	0.33
GO:0060445	branching involved in salivary gland morphogenesis	0.33
MP:0008033	impaired lipolysis	0.33
ENSG00000138802	SEC24B PPI subnetwork	0.33
ENSG00000101109	STK4 PPI subnetwork	0.33

Original gene set ID	Original gene set description	Nominal P value
ENSG00000181061	HIGD1A PPI subnetwork	0.33
GO:0005201	extracellular matrix structural constituent	0.33
ENSG00000140564	FURIN PPI subnetwork	0.33
REACTOME_METABOLISM_OF_CARBOHYDRATES	REACTOME_METABOLISM_OF_CARBOHYDRATES	0.33
MP:0005669	increased circulating leptin level	0.33
MP:0006126	abnormal outflow tract development	0.33
GO:0048565	digestive tract development	0.33
GO:0016577	histone demethylation	0.33
MP:0008826	abnormal splenic cell ratio	0.33
ENSG00000170345	FOS PPI subnetwork	0.33
ENSG00000127586	CHTF18 PPI subnetwork	0.33
GO:0002455	humoral immune response mediated by circulating immunoglobulin	0.33
ENSG00000057663	ATG5 PPI subnetwork	0.33
ENSG00000068024	HDAC4 PPI subnetwork	0.33
ENSG00000130772	MED18 PPI subnetwork	0.33
MP:0003932	abnormal molar crown morphology	0.33
GO:0051019	mitogen-activated protein kinase binding	0.33
ENSG00000163823	CCR1 PPI subnetwork	0.33
REACTOME_EXTRINSIC_PATHWAY_FOR_APOPTOSIS	REACTOME_EXTRINSIC_PATHWAY_FOR_APOPTOSIS	0.33
REACTOME_DEATH_RECEPTOR__SIGNALLING	REACTOME_DEATH_RECEPTOR__SIGNALLING	0.33
ENSG00000108773	KAT2A PPI subnetwork	0.33
GO:0048525	negative regulation of viral reproduction	0.33
GO:0045071	negative regulation of viral genome replication	0.33
GO:0007126	meiosis	0.33
GO:0051327	M phase of meiotic cell cycle	0.33
REACTOME_REMOVAL_OF_THE_FLAP_INTERMEDIATE	REACTOME_REMOVAL_OF_THE_FLAP_INTERMEDIATE	0.33
REACTOME_CLASS_C3_METABOTROPIC_GLUTAMATEPHEROMONE_RECEPTORS	REACTOME_CLASS_C3_METABOTROPIC_GLUTAMATEPHEROMONE_RECEPTORS	0.33
ENSG00000118503	TNFAIP3 PPI subnetwork	0.33
GO:0035336	long-chain fatty-acyl-CoA metabolic process	0.33
GO:0035338	long-chain fatty-acyl-CoA biosynthetic process	0.33
ENSG00000186340	THBS2 PPI subnetwork	0.33
GO:0016638	oxidoreductase activity, acting on the CH-NH2 group of donors	0.33
ENSG00000206211	ENSG00000206211 PPI subnetwork	0.33
ENSG00000204220	PFDN6 PPI subnetwork	0.33
ENSG00000206283	PFDN6 PPI subnetwork	0.33
ENSG00000171557	FGG PPI subnetwork	0.33
GO:0050792	regulation of viral reproduction	0.33
MP:0003743	abnormal facial morphology	0.33
MP:0005253	abnormal eye physiology	0.33
ENSG00000161980	POLR3K PPI subnetwork	0.33
GO:0008238	exopeptidase activity	0.33
MP:0003982	increased cholesterol level	0.33
MP:0000079	abnormal basioccipital bone morphology	0.33
GO:0005977	glycogen metabolic process	0.33
MP:0010418	perimembraneous ventricular septal defect	0.33
GO:0019827	stem cell maintenance	0.33
KEGG_CELL_ADHESION_MOLECULES_CAMS	KEGG_CELL_ADHESION_MOLECULES_CAMS	0.33
MP:0008284	abnormal hippocampus pyramidal cell layer	0.33
ENSG00000167674	ENSG00000167674 PPI subnetwork	0.33

Original gene set ID	Original gene set description	Nominal P value
MP:0002831	absent Peyer's patches	0.33
ENSG00000178568	ERBB4 PPI subnetwork	0.33
GO:0000272	polysaccharide catabolic process	0.33
GO:0034377	plasma lipoprotein particle assembly	0.33
GO:0065005	protein-lipid complex assembly	0.33
MP:0005358	abnormal incisor morphology	0.33
GO:0043296	apical junction complex	0.33
MP:0008438	abnormal cutaneous collagen fibril morphology	0.33
GO:0045597	positive regulation of cell differentiation	0.33
GO:0019717	synaptosome	0.33
ENSG00000102878	HSF4 PPI subnetwork	0.33
GO:0030427	site of polarized growth	0.33
KEGG_WNT_SIGNALING_PATHWAY	KEGG_WNT_SIGNALING_PATHWAY	0.33
ENSG00000078328	RBFOX1 PPI subnetwork	0.33
ENSG00000198231	DDX42 PPI subnetwork	0.33
GO:0007586	digestion	0.33
GO:0016558	protein import into peroxisome matrix	0.33
GO:0043022	ribosome binding	0.33
ENSG00000003402	CFLAR PPI subnetwork	0.33
MP:0001825	arrested T cell differentiation	0.33
ENSG00000174177	CTU2 PPI subnetwork	0.33
ENSG00000108592	FTSJ3 PPI subnetwork	0.33
GO:0071826	ribonucleoprotein complex subunit organization	0.33
GO:0002443	leukocyte mediated immunity	0.33
MP:0009395	increased nucleated erythrocyte cell number	0.33
ENSG00000181852	RNF41 PPI subnetwork	0.33
GO:0030879	mammary gland development	0.33
MP:0004901	decreased male germ cell number	0.33
ENSG00000170486	KRT72 PPI subnetwork	0.34
GO:0034504	protein localization to nucleus	0.34
ENSG00000164758	MED30 PPI subnetwork	0.34
REACTOME_NUCLEAR_SIGNALING_BY_ERBB4	REACTOME_NUCLEAR_SIGNALING_BY_ERBB4	0.34
ENSG00000175634	RPS6KB2 PPI subnetwork	0.34
ENSG00000146143	ENSG00000146143 PPI subnetwork	0.34
MP:0004948	abnormal neuronal precursor proliferation	0.34
MP:0008827	abnormal thymus cell ratio	0.34
MP:0002875	decreased erythrocyte cell number	0.34
GO:0005840	ribosome	0.34
MP:0009703	decreased birth body size	0.34
ENSG00000180138	CSNK1A1L PPI subnetwork	0.34
MP:0011083	complete lethality at weaning	0.34
ENSG00000139637	C12orf10 PPI subnetwork	0.34
GO:0071347	cellular response to interleukin-1	0.34
GO:0018904	organic ether metabolic process	0.34
ENSG00000143153	ATP1B1 PPI subnetwork	0.34
ENSG00000143622	RIT1 PPI subnetwork	0.34
ENSG00000188488	SERPINA5 PPI subnetwork	0.34
GO:0008146	sulfotransferase activity	0.34
GO:0016338	calcium-independent cell-cell adhesion	0.34

Original gene set ID	Original gene set description	Nominal P value
ENSG00000017260	ATP2C1 PPI subnetwork	0.34
GO:0008601	protein phosphatase type 2A regulator activity	0.34
ENSG00000149948	HMGA2 PPI subnetwork	0.34
ENSG00000160220	ENSG00000160220 PPI subnetwork	0.34
ENSG00000123384	LRP1 PPI subnetwork	0.34
MP:0008126	increased dendritic cell number	0.34
GO:0008022	protein C-terminus binding	0.34
GO:0007127	meiosis I	0.34
GO:0006897	endocytosis	0.34
GO:0010324	membrane invagination	0.34
ENSG00000134250	NOTCH2 PPI subnetwork	0.34
ENSG00000150459	SAP18 PPI subnetwork	0.34
ENSG00000213588	ZBTB9 PPI subnetwork	0.34
GO:0006073	cellular glucan metabolic process	0.34
GO:0044042	glucan metabolic process	0.34
ENSG00000147677	EIF3H PPI subnetwork	0.34
KEGG_ARRHYTHMOGENIC_RIGHT_VENTRICULAR_CARDIOMYOPATHY_ARVC	KEGG_ARRHYTHMOGENIC_RIGHT_VENTRICULAR_CARDIOMYOPATHY_ARVC	0.34
ENSG00000138433	CIR1 PPI subnetwork	0.34
MP:0001726	abnormal allantois morphology	0.34
ENSG00000106263	EIF3B PPI subnetwork	0.34
MP:0010263	total cataracts	0.34
REACTOME_SEMAPHORIN_INTERACTIONS	REACTOME_SEMAPHORIN_INTERACTIONS	0.34
GO:0002819	regulation of adaptive immune response	0.34
MP:0003993	abnormal ventral spinal root morphology	0.34
MP:0002705	dilated renal tubules	0.34
ENSG00000197249	SERPINA1 PPI subnetwork	0.34
ENSG00000152268	ENSG00000152268 PPI subnetwork	0.34
REACTOME_HDL:MEDIATED_LIPID_TRANSPORT	REACTOME_HDL:MEDIATED_LIPID_TRANSPORT	0.34
MP:0006027	impaired lung alveolus development	0.34
MP:0010249	lactation failure	0.34
GO:0004623	phospholipase A2 activity	0.34
REACTOME_SIGNALING_BY_TGF_BETA	REACTOME_SIGNALING_BY_TGF_BETA	0.34
GO:0001736	establishment of planar polarity	0.34
ENSG00000006638	TBXA2R PPI subnetwork	0.34
GO:0016875	ligase activity, forming carbon-oxygen bonds	0.34
GO:0004812	aminoacyl-tRNA ligase activity	0.34
GO:0016876	ligase activity, forming aminoacyl-tRNA and related compounds	0.34
ENSG00000196374	HIST1H2BM PPI subnetwork	0.34
MP:0004359	short ulna	0.34
MP:0000109	abnormal parietal bone morphology	0.34
MP:0005103	abnormal retinal pigmentation	0.34
MP:0005289	increased oxygen consumption	0.34
REACTOME_UNWINDING_OF_DNA	REACTOME_UNWINDING_OF_DNA	0.34
GO:0030175	filopodium	0.34
MP:0000639	abnormal adrenal gland morphology	0.34
MP:0002499	chronic inflammation	0.34
GO:0048568	embryonic organ development	0.34
ENSG00000173674	EIF1AX PPI subnetwork	0.34
GO:0015020	glucuronosyltransferase activity	0.34

Original gene set ID	Original gene set description	Nominal P value
ENSG00000108559	NUP88 PPI subnetwork	0.34
GO:0006084	acetyl-CoA metabolic process	0.34
GO:0050920	regulation of chemotaxis	0.34
MP:0000333	decreased bone marrow cell number	0.34
GO:0003725	double-stranded RNA binding	0.34
MP:0010403	atrial septal defect	0.34
GO:0005921	gap junction	0.34
GO:0003013	circulatory system process	0.34
ENSG00000102871	TRADD PPI subnetwork	0.34
GO:0046134	pyrimidine nucleoside biosynthetic process	0.34
GO:0032103	positive regulation of response to external stimulus	0.34
GO:0045055	regulated secretory pathway	0.34
GO:0043189	H4/H2A histone acetyltransferase complex	0.34
ENSG00000156697	UTP14A PPI subnetwork	0.34
GO:0045182	translation regulator activity	0.34
ENSG00000080824	HSP90AA1 PPI subnetwork	0.34
GO:0002753	cytoplasmic pattern recognition receptor signaling pathway	0.34
GO:0070423	nucleotide-binding oligomerization domain containing signaling pathway	0.34
GO:0035872	nucleotide-binding domain, leucine rich repeat containing receptor signaling pathwa	0.34
MP:0011093	complete embryonic lethality at implantation	0.34
GO:0015934	large ribosomal subunit	0.34
ENSG00000134376	CRB1 PPI subnetwork	0.34
ENSG00000131269	ABCB7 PPI subnetwork	0.34
ENSG00000155438	MKI67IP PPI subnetwork	0.34
MP:0000217	abnormal leukocyte cell number	0.34
REACTOME_NOTCH:HLH_TRANSCRIPTION_PATHWAY	REACTOME_NOTCH:HLH_TRANSCRIPTION_PATHWAY	0.34
REACTOME_NICD_TRAFFICS_TO_NUCLEUS	REACTOME_NICD_TRAFFICS_TO_NUCLEUS	0.34
ENSG00000198918	RPL39 PPI subnetwork	0.34
MP:0000141	abnormal vertebral body morphology	0.34
GO:0050768	negative regulation of neurogenesis	0.34
GO:0046545	development of primary female sexual characteristics	0.34
GO:0007589	body fluid secretion	0.34
ENSG00000131462	TUBG1 PPI subnetwork	0.34
ENSG00000026508	CD44 PPI subnetwork	0.34
ENSG00000066926	FECH PPI subnetwork	0.34
MP:0000063	decreased bone mineral density	0.34
GO:0002684	positive regulation of immune system process	0.34
MP:0003052	omphalocele	0.34
GO:0009395	phospholipid catabolic process	0.34
ENSG00000064300	NGFR PPI subnetwork	0.34
ENSG00000185745	IFIT1 PPI subnetwork	0.34
ENSG00000171314	PGAM1 PPI subnetwork	0.34
GO:0045576	mast cell activation	0.34
GO:0015296	anion:cation symporter activity	0.34
ENSG00000125485	DDX31 PPI subnetwork	0.34
ENSG00000114978	MOB1A PPI subnetwork	0.34
ENSG00000197063	MAFG PPI subnetwork	0.35
ENSG00000076003	MCM6 PPI subnetwork	0.35
REACTOME_GLYCOGEN_BREAKDOWN_GLYCOGENOLYSIS	REACTOME_GLYCOGEN_BREAKDOWN_GLYCOGENOLYSIS	0.35

Original gene set ID	Original gene set description	Nominal P value
ENSG00000078579	FGF20 PPI subnetwork	0.35
MP:0004231	abnormal calcium ion homeostasis	0.35
ENSG00000142252	GEMIN7 PPI subnetwork	0.35
ENSG00000111450	STX2 PPI subnetwork	0.35
GO:0042277	peptide binding	0.35
GO:0042623	ATPase activity, coupled	0.35
GO:0016327	apicolateral plasma membrane	0.35
ENSG00000008710	PKD1 PPI subnetwork	0.35
MP:0006055	abnormal vascular endothelial cell morphology	0.35
ENSG00000196504	PRPF40A PPI subnetwork	0.35
MP:0008450	retinal photoreceptor degeneration	0.35
ENSG00000095015	MAP3K1 PPI subnetwork	0.35
MP:0000474	abnormal foregut morphology	0.35
GO:0008135	translation factor activity, nucleic acid binding	0.35
ENSG00000130726	TRIM28 PPI subnetwork	0.35
MP:0001914	hemorrhage	0.35
MP:0011348	abnormal renal glomerulus basement membrane morphology	0.35
ENSG00000147044	CASK PPI subnetwork	0.35
GO:0043627	response to estrogen stimulus	0.35
ENSG00000037042	TUBG2 PPI subnetwork	0.35
ENSG00000164692	COL1A2 PPI subnetwork	0.35
MP:0005348	increased T cell proliferation	0.35
ENSG00000100697	DICER1 PPI subnetwork	0.35
MP:0001326	retinal degeneration	0.35
ENSG00000047056	WDR37 PPI subnetwork	0.35
MP:0003997	tonic-clonic seizures	0.35
ENSG00000173889	PHC3 PPI subnetwork	0.35
MP:0000921	demyelination	0.35
GO:0045088	regulation of innate immune response	0.35
ENSG00000110245	APOC3 PPI subnetwork	0.35
ENSG00000215755	ENSG00000215755 PPI subnetwork	0.35
KEGG_NATURAL_KILLER_CELL_MEDIATED_CYTOTOXICITY	KEGG_NATURAL_KILLER_CELL_MEDIATED_CYTOTOXICITY	0.35
GO:0002429	immune response-activating cell surface receptor signaling pathway	0.35
GO:0004835	tubulin-tyrosine ligase activity	0.35
ENSG00000130520	LSM4 PPI subnetwork	0.35
REACTOME_FORMATION_OF_THE_TERNARY_COMPLEX_AND_SUBSEQUENTLY_	REACTOME_FORMATION_OF_THE_TERNARY_COMPLEX_AND_SUBSEQUENTLY_THE	0.35
GO:0045621	positive regulation of lymphocyte differentiation	0.35
REACTOME_ACTIVATED_TLR4_SIGNALLING	REACTOME_ACTIVATED_TLR4_SIGNALLING	0.35
ENSG00000086232	EIF2AK1 PPI subnetwork	0.35
REACTOME_TRANSLOCATION_OF_ZAP:70_TO_IMMUNOLOGICAL_SYNAPSE	REACTOME_TRANSLOCATION_OF_ZAP:70_TO_IMMUNOLOGICAL_SYNAPSE	0.35
MP:0001844	autoimmune response	0.35
GO:0030335	positive regulation of cell migration	0.35
ENSG00000114737	CISH PPI subnetwork	0.35
GO:0006820	anion transport	0.35
GO:0007088	regulation of mitosis	0.35
GO:0051783	regulation of nuclear division	0.35
MP:0008071	absent B cells	0.35
GO:0042098	T cell proliferation	0.35
REACTOME_GAP_JUNCTION_ASSEMBLY	REACTOME_GAP_JUNCTION_ASSEMBLY	0.35

Original gene set ID	Original gene set description	Nominal P value
GO:0042481	regulation of odontogenesis	0.35
GO:0008643	carbohydrate transport	0.35
ENSG00000099725	ENSG00000099725 PPI subnetwork	0.35
GO:0018410	C-terminal protein amino acid modification	0.35
ENSG00000157227	MMP14 PPI subnetwork	0.35
MP:0001158	abnormal prostate gland morphology	0.35
ENSG00000105085	MED26 PPI subnetwork	0.35
ENSG00000212908	ENSG00000212908 PPI subnetwork	0.35
ENSG00000213416	KRTAP4-12 PPI subnetwork	0.35
GO:0001782	B cell homeostasis	0.35
ENSG00000115946	PNO1 PPI subnetwork	0.35
GO:0071305	cellular response to vitamin D	0.35
GO:0006107	oxaloacetate metabolic process	0.35
GO:0070988	demethylation	0.35
GO:0018279	protein N-linked glycosylation via asparagine	0.35
GO:0018196	peptidyl-asparagine modification	0.35
ENSG00000118985	ELL2 PPI subnetwork	0.35
ENSG00000206212	ENSG00000206212 PPI subnetwork	0.35
ENSG00000182498	ENSG00000182498 PPI subnetwork	0.35
ENSG00000096150	RPS18 PPI subnetwork	0.35
GO:0046847	filopodium assembly	0.35
ENSG00000135387	CAPRIN1 PPI subnetwork	0.35
MP:0011090	partial perinatal lethality	0.35
GO:0032369	negative regulation of lipid transport	0.35
GO:0002821	positive regulation of adaptive immune response	0.35
ENSG00000111537	IFNG PPI subnetwork	0.35
ENSG00000135404	CD63 PPI subnetwork	0.35
GO:0009156	ribonucleoside monophosphate biosynthetic process	0.35
ENSG00000123091	RNF11 PPI subnetwork	0.35
GO:0051905	establishment of pigment granule localization	0.35
MP:0002357	abnormal spleen white pulp morphology	0.35
GO:0045582	positive regulation of T cell differentiation	0.35
GO:0051098	regulation of binding	0.35
GO:0005839	proteasome core complex	0.35
MP:0004704	short vertebral column	0.35
REACTOME_PHOSPHORYLATION_OF_CD3_AND_TCR_ZETA_CHAINS	REACTOME_PHOSPHORYLATION_OF_CD3_AND_TCR_ZETA_CHAINS	0.35
ENSG00000167552	TUBA1A PPI subnetwork	0.35
ENSG00000136931	NR5A1 PPI subnetwork	0.35
ENSG00000119953	SMNDC1 PPI subnetwork	0.35
GO:0017053	transcriptional repressor complex	0.35
ENSG00000130203	APOE PPI subnetwork	0.35
GO:0015662	ATPase activity, coupled to transmembrane movement of ions, phosphorylative me	0.35
ENSG00000145423	SFRP2 PPI subnetwork	0.35
GO:0071375	cellular response to peptide hormone stimulus	0.35
GO:0030318	melanocyte differentiation	0.35
GO:0030595	leukocyte chemotaxis	0.35
GO:0030036	actin cytoskeleton organization	0.35
ENSG00000139515	PDX1 PPI subnetwork	0.35
ENSG00000006075	CCL3 PPI subnetwork	0.35

Original gene set ID	Original gene set description	Nominal P value
MP:0001785	edema	0.35
GO:0006956	complement activation	0.35
REACTOME_L1CAM_INTERACTIONS	REACTOME_L1CAM_INTERACTIONS	0.35
REACTOME_GAP_JUNCTION_TRAFFICKING_AND_REGULATION	REACTOME_GAP_JUNCTION_TRAFFICKING_AND_REGULATION	0.35
ENSG00000142892	PIGK PPI subnetwork	0.35
KEGG_TOLL_LIKE_RECEPTOR_SIGNALING_PATHWAY	KEGG_TOLL_LIKE_RECEPTOR_SIGNALING_PATHWAY	0.35
ENSG00000088356	PDRG1 PPI subnetwork	0.35
MP:0004608	abnormal cervical axis morphology	0.36
MP:0006029	abnormal sclerotome morphology	0.36
ENSG00000077097	TOP2B PPI subnetwork	0.36
MP:0006303	abnormal retinal nerve fiber layer morphology	0.36
ENSG00000184083	FAM120C PPI subnetwork	0.36
MP:0002857	cochlear ganglion degeneration	0.36
ENSG00000004799	PDK4 PPI subnetwork	0.36
ENSG00000108826	MRPL27 PPI subnetwork	0.36
ENSG00000138346	DNA2 PPI subnetwork	0.36
ENSG00000178562	CD28 PPI subnetwork	0.36
ENSG00000143748	NVL PPI subnetwork	0.36
MP:0003051	curly tail	0.36
MP:0006279	abnormal limb development	0.36
REACTOME_MITOTIC_G2:G2M_PHASES	REACTOME_MITOTIC_G2:G2M_PHASES	0.36
ENSG00000136573	BLK PPI subnetwork	0.36
GO:0046928	regulation of neurotransmitter secretion	0.36
ENSG00000117091	CD48 PPI subnetwork	0.36
GO:0035257	nuclear hormone receptor binding	0.36
GO:0034142	toll-like receptor 4 signaling pathway	0.36
ENSG00000084674	APOB PPI subnetwork	0.36
GO:0006767	water-soluble vitamin metabolic process	0.36
REACTOME_DOWNSTREAM_TCR_SIGNALING	REACTOME_DOWNSTREAM_TCR_SIGNALING	0.36
ENSG00000104722	NEFM PPI subnetwork	0.36
MP:0001175	abnormal lung morphology	0.36
REACTOME_ACTIVATION_OF_THE_PRE:REPLICATIVE_COMPLEX	REACTOME_ACTIVATION_OF_THE_PRE:REPLICATIVE_COMPLEX	0.36
ENSG00000172845	SP3 PPI subnetwork	0.36
GO:0035085	cilium axoneme	0.36
MP:0011091	complete prenatal lethality	0.36
ENSG00000126247	CAPNS1 PPI subnetwork	0.36
MP:0008567	decreased interferon-gamma secretion	0.36
ENSG00000134954	ETS1 PPI subnetwork	0.36
GO:0043586	tongue development	0.36
ENSG00000085511	MAP3K4 PPI subnetwork	0.36
GO:0004175	endopeptidase activity	0.36
GO:0016605	PML body	0.36
ENSG00000093000	NUP50 PPI subnetwork	0.36
ENSG00000165637	VDAC2 PPI subnetwork	0.36
ENSG00000168918	INPP5D PPI subnetwork	0.36
ENSG00000120802	TMPO PPI subnetwork	0.36
REACTOME_TRIF_MEDIATED_TLR3_SIGNALING	REACTOME_TRIF_MEDIATED_TLR3_SIGNALING	0.36
REACTOME_TOLL_LIKE_RECEPTOR_3_TLR3_CASCADE	REACTOME_TOLL_LIKE_RECEPTOR_3_TLR3_CASCADE	0.36
REACTOME_RNA_POLYMERASE_I_RNA_POLYMERASE_III_AND_MITOCHONDRIA	REACTOME_RNA_POLYMERASE_I_RNA_POLYMERASE_III_AND_MITOCHONDRIAL_T	0.36

Original gene set ID	Original gene set description	Nominal P value
MP:0001392	abnormal locomotor behavior	0.36
GO:0043218	compact myelin	0.36
KEGG_AMINOACYL_TRNA_BIOSYNTHESIS	KEGG_AMINOACYL_TRNA_BIOSYNTHESIS	0.36
ENSG00000168542	COL3A1 PPI subnetwork	0.36
ENSG00000163558	PRKCI PPI subnetwork	0.36
ENSG00000179295	PTPN11 PPI subnetwork	0.36
GO:0070192	chromosome organization involved in meiosis	0.36
ENSG00000117906	RCN2 PPI subnetwork	0.36
GO:0007606	sensory perception of chemical stimulus	0.36
GO:0048066	developmental pigmentation	0.36
MP:0005016	decreased lymphocyte cell number	0.36
MP:0005065	abnormal neutrophil morphology	0.36
ENSG00000158691	ZSCAN12 PPI subnetwork	0.36
ENSG00000124214	STAU1 PPI subnetwork	0.36
ENSG00000100413	POLR3H PPI subnetwork	0.36
GO:0006959	humoral immune response	0.36
GO:0071827	plasma lipoprotein particle organization	0.36
GO:0071825	protein-lipid complex subunit organization	0.36
ENSG00000075415	SLC25A3 PPI subnetwork	0.36
MP:0002962	increased urine protein level	0.36
ENSG00000111266	DUSP16 PPI subnetwork	0.36
REACTOME_INFLUENZA_VIRAL_RNA_TRANSCRIPTION_AND_REPLICATION	REACTOME_INFLUENZA_VIRAL_RNA_TRANSCRIPTION_AND_REPLICATION	0.36
GO:0019903	protein phosphatase binding	0.36
ENSG00000166484	MAPK7 PPI subnetwork	0.36
GO:0016891	endoribonuclease activity, producing 5'-phosphomonoesters	0.36
GO:0050727	regulation of inflammatory response	0.36
REACTOME_UNFOLDED_PROTEIN_RESPONSE	REACTOME_UNFOLDED_PROTEIN_RESPONSE	0.36
ENSG00000105939	ZC3HAV1 PPI subnetwork	0.36
MP:0004796	increased anti-histone antibody level	0.36
ENSG00000109072	SEBOX PPI subnetwork	0.36
ENSG00000132703	APCS PPI subnetwork	0.36
REACTOME_GLUCONEOGENESIS	REACTOME_GLUCONEOGENESIS	0.36
ENSG00000100316	RPL3 PPI subnetwork	0.36
ENSG00000154096	THY1 PPI subnetwork	0.36
ENSG00000129083	COPB1 PPI subnetwork	0.36
GO:0005096	GTPase activator activity	0.36
GO:0046965	retinoid X receptor binding	0.36
ENSG00000206506	HLA-G PPI subnetwork	0.36
ENSG00000206443	ENSG00000206443 PPI subnetwork	0.36
ENSG00000204632	HLA-G PPI subnetwork	0.36
MP:0001504	abnormal posture	0.36
ENSG00000168488	ATXN2L PPI subnetwork	0.36
REACTOME_ERKMAPK_TARGETS	REACTOME_ERKMAPK_TARGETS	0.36
ENSG00000062038	CDH3 PPI subnetwork	0.36
ENSG00000005007	UPF1 PPI subnetwork	0.36
ENSG00000115977	AAK1 PPI subnetwork	0.36
MP:0008279	arrest of spermiogenesis	0.36
ENSG00000105610	KLF1 PPI subnetwork	0.36
ENSG00000196943	C14orf21 PPI subnetwork	0.36

Original gene set ID	Original gene set description	Nominal P value
GO:0045672	positive regulation of osteoclast differentiation	0.36
GO:0005625	soluble fraction	0.36
GO:0006487	protein N-linked glycosylation	0.36
MP:0008582	short photoreceptor inner segment	0.36
ENSG00000147649	MTDH PPI subnetwork	0.36
GO:0046486	glycerolipid metabolic process	0.36
KEGG_GLYCOSPHINGOLIPID_BIOSYNTHESIS_GLOBO_SERIES	KEGG_GLYCOSPHINGOLIPID_BIOSYNTHESIS_GLOBO_SERIES	0.36
ENSG00000174175	SELP PPI subnetwork	0.36
GO:0005922	connexon complex	0.36
GO:0006493	protein O-linked glycosylation	0.36
ENSG00000162613	FUBP1 PPI subnetwork	0.36
ENSG00000162924	REL PPI subnetwork	0.36
GO:0090257	regulation of muscle system process	0.36
MP:0009937	abnormal neuron differentiation	0.36
MP:0000164	abnormal cartilage development	0.36
MP:0004982	abnormal osteoclast morphology	0.36
REACTOME_NFKB_AND_MAP_KINASES_ACTIVATION_MEDIATED_BY_TLR4_SIGI	REACTOME_NFKB_AND_MAP_KINASES_ACTIVATION_MEDIATED_BY_TLR4_SIGNALI	0.36
GO:0008656	cysteine-type endopeptidase activator activity involved in apoptotic process	0.36
GO:0018298	protein-chromophore linkage	0.36
ENSG00000112282	MED23 PPI subnetwork	0.36
ENSG00000114209	PDCD10 PPI subnetwork	0.36
GO:0061035	regulation of cartilage development	0.36
MP:0008641	increased circulating interleukin-1 beta level	0.36
KEGG_GLYCEROPHOSPHOLIPID_METABOLISM	KEGG_GLYCEROPHOSPHOLIPID_METABOLISM	0.36
GO:0050931	pigment cell differentiation	0.36
GO:0009408	response to heat	0.36
REACTOME_ENDOGENOUS_STEROLS	REACTOME_ENDOGENOUS_STEROLS	0.36
GO:0001916	positive regulation of T cell mediated cytotoxicity	0.36
GO:0046949	fatty-acyl-CoA biosynthetic process	0.36
GO:0035337	fatty-acyl-CoA metabolic process	0.36
REACTOME_CONVERSION_FROM_APCCCDC20_TO_APCCCDH1_IN_LATE_ANAPI	REACTOME_CONVERSION_FROM_APCCCDC20_TO_APCCCDH1_IN_LATE_ANAPHASI	0.36
GO:0007141	male meiosis I	0.36
ENSG00000153395	LPCAT1 PPI subnetwork	0.36
MP:0008540	abnormal cerebrum morphology	0.36
REACTOME_MYD88:INDEPENDENT_CASCADE_INITIATED_ON_PLASMA_MEMBF	REACTOME_MYD88:INDEPENDENT_CASCADE_INITIATED_ON_PLASMA_MEMBRANI	0.37
GO:0070936	protein K48-linked ubiquitination	0.37
REACTOME_REGULATION_OF_INSULIN:LIKE_GROWTH_FACTOR_IGF_ACTIVITY_	REACTOME_REGULATION_OF_INSULIN:LIKE_GROWTH_FACTOR_IGF_ACTIVITY_BY_	0.37
MP:0003651	abnormal axon outgrowth	0.37
GO:0005923	tight junction	0.37
GO:0070160	occluding junction	0.37
GO:0008037	cell recognition	0.37
GO:0070193	synaptonemal complex organization	0.37
ENSG00000165630	PRPF18 PPI subnetwork	0.37
ENSG00000184110	EIF3C PPI subnetwork	0.37
ENSG00000149187	CELF1 PPI subnetwork	0.37
ENSG00000066336	SPI1 PPI subnetwork	0.37
GO:0005516	calmodulin binding	0.37
GO:0003016	respiratory system process	0.37
ENSG00000185236	RAB11B PPI subnetwork	0.37

Original gene set ID	Original gene set description	Nominal P value
MP:0005105	abnormal middle ear ossicle morphology	0.37
ENSG00000113569	NUP155 PPI subnetwork	0.37
MP:0002490	abnormal immunoglobulin level	0.37
MP:0009788	increased susceptibility to bacterial infection induced morbidity/mortality	0.37
GO:0017147	Wnt-protein binding	0.37
ENSG00000001167	NFYA PPI subnetwork	0.37
GO:0042558	pteridine-containing compound metabolic process	0.37
ENSG00000034693	PEX3 PPI subnetwork	0.37
ENSG00000142875	PRKACB PPI subnetwork	0.37
MP:0008151	increased diameter of long bones	0.37
ENSG00000107779	BMPR1A PPI subnetwork	0.37
MP:0001488	increased startle reflex	0.37
REACTOME_HEXOSE_TRANSPORT	REACTOME_HEXOSE_TRANSPORT	0.37
REACTOME_SIGNALLING_TO_ERKS	REACTOME_SIGNALLING_TO_ERKS	0.37
MP:0005078	abnormal cytotoxic T cell physiology	0.37
ENSG00000101966	XIAP PPI subnetwork	0.37
MP:0002948	abnormal neuron specification	0.37
ENSG00000170035	UBE2E3 PPI subnetwork	0.37
ENSG00000160917	CPSF4 PPI subnetwork	0.37
ENSG00000198010	DLGAP2 PPI subnetwork	0.37
ENSG00000068438	FTSJ1 PPI subnetwork	0.37
GO:0042166	acetylcholine binding	0.37
MP:0001780	decreased brown adipose tissue amount	0.37
GO:0010212	response to ionizing radiation	0.37
REACTOME_SYNTHESIS_OF_VERY_LONG:CHAIN_FATTY_ACYL:COAS	REACTOME_SYNTHESIS_OF_VERY_LONG:CHAIN_FATTY_ACYL:COAS	0.37
GO:0042101	T cell receptor complex	0.37
GO:0006984	ER-nucleus signaling pathway	0.37
ENSG00000182568	SATB1 PPI subnetwork	0.37
ENSG00000128272	ATF4 PPI subnetwork	0.37
GO:0005788	endoplasmic reticulum lumen	0.37
MP:0008572	abnormal Purkinje cell dendrite morphology	0.37
GO:0007220	Notch receptor processing	0.37
GO:0031349	positive regulation of defense response	0.37
ENSG00000173145	NOC3L PPI subnetwork	0.37
ENSG00000101473	ACOT8 PPI subnetwork	0.37
ENSG00000110876	SELPLG PPI subnetwork	0.37
REACTOME_RETROGRADE_NEUROTROPHIN_SIGNALLING	REACTOME_RETROGRADE_NEUROTROPHIN_SIGNALLING	0.37
MP:0001648	abnormal apoptosis	0.37
GO:0004861	cyclin-dependent protein kinase inhibitor activity	0.37
ENSG00000112592	TBP PPI subnetwork	0.37
GO:0002224	toll-like receptor signaling pathway	0.37
ENSG00000084754	HADHA PPI subnetwork	0.37
ENSG00000168959	GRM5 PPI subnetwork	0.37
GO:0006261	DNA-dependent DNA replication	0.37
ENSG00000071082	RPL31 PPI subnetwork	0.37
GO:0003735	structural constituent of ribosome	0.37
ENSG00000127993	RBM48 PPI subnetwork	0.37
MP:0001257	increased body length	0.37
ENSG00000062822	POLD1 PPI subnetwork	0.37

Original gene set ID	Original gene set description	Nominal P value
ENSG00000184489	PTP4A3 PPI subnetwork	0.37
ENSG00000161618	ALDH16A1 PPI subnetwork	0.37
ENSG00000100304	TTLL12 PPI subnetwork	0.37
MP:0003934	abnormal pancreas development	0.37
GO:0071564	npBAF complex	0.37
KEGG_ALDOSTERONE_REGULATED_SODIUM_REABSORPTION	KEGG_ALDOSTERONE_REGULATED_SODIUM_REABSORPTION	0.37
ENSG00000111344	RASAL1 PPI subnetwork	0.37
MP:0008006	increased stomach pH	0.37
ENSG00000133740	E2F5 PPI subnetwork	0.37
ENSG00000067715	SYT1 PPI subnetwork	0.37
KEGG_ENDOMETRIAL_CANCER	KEGG_ENDOMETRIAL_CANCER	0.37
ENSG00000148303	RPL7A PPI subnetwork	0.37
GO:0006385	transcription elongation from RNA polymerase III promoter	0.37
GO:0006386	termination of RNA polymerase III transcription	0.37
ENSG00000160447	PKN3 PPI subnetwork	0.37
GO:0071901	negative regulation of protein serine/threonine kinase activity	0.37
ENSG00000131759	RARA PPI subnetwork	0.37
GO:0055012	ventricular cardiac muscle cell differentiation	0.37
GO:0051196	regulation of coenzyme metabolic process	0.37
GO:0051193	regulation of cofactor metabolic process	0.37
GO:0060042	retina morphogenesis in camera-type eye	0.37
MP:0006009	abnormal neuronal migration	0.37
ENSG00000164885	CDK5 PPI subnetwork	0.37
MP:0004130	abnormal muscle cell glucose uptake	0.37
ENSG00000078053	AMPH PPI subnetwork	0.37
GO:0008033	tRNA processing	0.37
ENSG00000145386	CCNA2 PPI subnetwork	0.37
ENSG00000109062	SLC9A3R1 PPI subnetwork	0.37
ENSG00000137955	RABGGTB PPI subnetwork	0.37
ENSG00000105325	FZR1 PPI subnetwork	0.37
ENSG00000147140	NONO PPI subnetwork	0.37
ENSG00000101003	GINS1 PPI subnetwork	0.37
ENSG00000188419	CHM PPI subnetwork	0.37
ENSG00000076928	ARHGEF1 PPI subnetwork	0.37
ENSG00000073584	SMARCE1 PPI subnetwork	0.37
MP:0003910	decreased eating behavior	0.37
ENSG00000074966	TXK PPI subnetwork	0.37
MP:0001823	thymus hypoplasia	0.37
GO:0016878	acid-thiol ligase activity	0.37
REACTOME_METABOLISM_OF_MRNA	REACTOME_METABOLISM_OF_MRNA	0.37
REACTOME_SEMA3A_PAK_DEPENDENT_AXON_REPULSION	REACTOME_SEMA3A_PAK_DEPENDENT_AXON_REPULSION	0.37
GO:0009986	cell surface	0.37
MP:0008515	thin retinal outer nuclear layer	0.37
GO:0005544	calcium-dependent phospholipid binding	0.37
GO:0051090	regulation of sequence-specific DNA binding transcription factor activity	0.37
ENSG00000157557	ETS2 PPI subnetwork	0.37
GO:0006198	cAMP catabolic process	0.37
GO:0072663	establishment of protein localization to peroxisome	0.37
GO:0072662	protein localization to peroxisome	0.37

Original gene set ID	Original gene set description	Nominal P value
GO:0006625	protein targeting to peroxisome	0.37
ENSG00000206452	HLA-C PPI subnetwork	0.37
GO:0070304	positive regulation of stress-activated protein kinase signaling cascade	0.37
ENSG00000148334	PTGES2 PPI subnetwork	0.37
ENSG00000188612	SUMO2 PPI subnetwork	0.37
ENSG00000148719	DNAJB12 PPI subnetwork	0.38
MP:0004045	abnormal cell cycle checkpoint function	0.38
ENSG00000133316	WDR74 PPI subnetwork	0.38
REACTOME_POST:CHAPERONIN_TUBULIN_FOLDING_PATHWAY	REACTOME_POST:CHAPERONIN_TUBULIN_FOLDING_PATHWAY	0.38
GO:2000145	regulation of cell motility	0.38
ENSG00000100599	RIN3 PPI subnetwork	0.38
MP:0000662	abnormal branching of the mammary ductal tree	0.38
GO:0035102	PRC1 complex	0.38
MP:0008866	chromosomal instability	0.38
ENSG00000168646	AXIN2 PPI subnetwork	0.38
ENSG00000204394	VARS PPI subnetwork	0.38
ENSG00000096171	VARS PPI subnetwork	0.38
MP:0002095	abnormal skin pigmentation	0.38
ENSG00000136653	RASSF5 PPI subnetwork	0.38
GO:0031143	pseudopodium	0.38
GO:0016538	cyclin-dependent protein kinase regulator activity	0.38
GO:0016324	apical plasma membrane	0.38
ENSG00000144554	FANCD2 PPI subnetwork	0.38
ENSG00000104825	NFKBIB PPI subnetwork	0.38
KEGG_CYTOKINE_CYTOKINE_RECEPTOR_INTERACTION	KEGG_CYTOKINE_CYTOKINE_RECEPTOR_INTERACTION	0.38
ENSG00000159692	CTBP1 PPI subnetwork	0.38
MP:0005281	increased fatty acid level	0.38
ENSG00000143226	FCGR2A PPI subnetwork	0.38
GO:0035586	purinergic receptor activity	0.38
ENSG00000152942	RAD17 PPI subnetwork	0.38
MP:0004771	increased anti-single stranded DNA antibody leve	0.38
MP:0001505	hunched posture	0.38
REACTOME_INTERACTION_BETWEEN_L1_AND_ANKYRINS	REACTOME_INTERACTION_BETWEEN_L1_AND_ANKYRINS	0.38
GO:0021879	forebrain neuron differentiation	0.38
ENSG00000115484	CCT4 PPI subnetwork	0.38
ENSG00000084774	CAD PPI subnetwork	0.38
ENSG00000198336	MYL4 PPI subnetwork	0.38
ENSG00000171444	MCC PPI subnetwork	0.38
ENSG00000129347	KRI1 PPI subnetwork	0.38
REACTOME_SIGNALLING_TO_RAS	REACTOME_SIGNALLING_TO_RAS	0.38
GO:0030334	regulation of cell migration	0.38
MP:0008642	decreased circulating interleukin-1 beta leve	0.38
MP:0011092	complete embryonic lethality	0.38
REACTOME_CTLA4_INHIBITORY_SIGNALING	REACTOME_CTLA4_INHIBITORY_SIGNALING	0.38
GO:0019432	triglyceride biosynthetic process	0.38
ENSG00000130332	LSM7 PPI subnetwork	0.38
MP:0003729	abnormal photoreceptor outer segment morphology	0.38
ENSG00000126778	SIX1 PPI subnetwork	0.38
GO:0031461	cullin-RING ubiquitin ligase complex	0.38

Original gene set ID	Original gene set description	Nominal P value
GO:0048589	developmental growth	0.38
ENSG00000100347	SAMM50 PPI subnetwork	0.38
MP:0004751	increased length of allograft survival	0.38
GO:0042562	hormone binding	0.38
MP:0004618	thoracic vertebral transformation	0.38
MP:0005070	impaired NK cell cytolysis	0.38
ENSG00000169783	LINGO1 PPI subnetwork	0.38
ENSG00000171530	TBCA PPI subnetwork	0.38
ENSG00000164053	ATRIP PPI subnetwork	0.38
ENSG00000139083	ETV6 PPI subnetwork	0.38
REACTOME_SEMA4D_INDUCED_CELL_MIGRATION_AND_GROWTH:CONE_COLL	REACTOME_SEMA4D_INDUCED_CELL_MIGRATION_AND_GROWTH:CONE_COLLAPS	0.38
GO:0042493	response to drug	0.38
ENSG00000101084	C20orf24 PPI subnetwork	0.38
REACTOME_INSULIN_RECEPTOR_SIGNALLING_CASCADE	REACTOME_INSULIN_RECEPTOR_SIGNALLING_CASCADE	0.38
GO:0043560	insulin receptor substrate binding	0.38
GO:0007339	binding of sperm to zona pellucida	0.38
MP:0008168	decreased B-1a cell number	0.38
ENSG00000137841	PLCB2 PPI subnetwork	0.38
ENSG00000150995	ITPR1 PPI subnetwork	0.38
GO:0050851	antigen receptor-mediated signaling pathway	0.38
REACTOME_CHAPERONIN:MEDIATED_PROTEIN_FOLDING	REACTOME_CHAPERONIN:MEDIATED_PROTEIN_FOLDING	0.38
GO:0034433	steroid esterification	0.38
GO:0034435	cholesterol esterification	0.38
GO:0034434	sterol esterification	0.38
GO:0009108	coenzyme biosynthetic process	0.38
MP:0005616	decreased susceptibility to type IV hypersensitivity reaction	0.38
GO:0031527	filopodium membrane	0.38
MP:0004007	abnormal lung vasculature morphology	0.38
ENSG00000205022	PABPN1L PPI subnetwork	0.38
ENSG00000138696	BMPR1B PPI subnetwork	0.38
ENSG00000142657	PGD PPI subnetwork	0.38
MP:0002451	abnormal macrophage physiology	0.38
GO:0042445	hormone metabolic process	0.38
MP:0008261	arrest of male meiosis	0.38
GO:0000146	microfilament motor activity	0.38
MP:0000564	syndactyly	0.38
MP:0002339	abnormal lymph node morphology	0.38
MP:0004919	abnormal positive T cell selection	0.38
ENSG00000196083	IL1RAP PPI subnetwork	0.38
ENSG00000142871	CYR61 PPI subnetwork	0.38
GO:0009988	cell-cell recognition	0.38
ENSG00000143466	IKBKE PPI subnetwork	0.38
ENSG00000148175	STOM PPI subnetwork	0.38
GO:0048864	stem cell development	0.38
ENSG00000143768	LEFTY2 PPI subnetwork	0.38
GO:0010564	regulation of cell cycle process	0.38
REACTOME_GLUCOSE_TRANSPORT	REACTOME_GLUCOSE_TRANSPORT	0.38
GO:0002279	mast cell activation involved in immune response	0.38
GO:0001667	ameboidal cell migration	0.38

Original gene set ID	Original gene set description	Nominal P value
ENSG00000100612	DHRS7 PPI subnetwork	0.38
ENSG00000149182	ARFGAP2 PPI subnetwork	0.38
ENSG00000114353	GNAI2 PPI subnetwork	0.38
GO:0030426	growth cone	0.38
REACTOME_PECAM1_INTERACTIONS	REACTOME_PECAM1_INTERACTIONS	0.38
MP:0002442	abnormal leukocyte physiology	0.38
MP:0008673	decreased interleukin-13 secretion	0.38
MP:0003564	abnormal insulin secretion	0.38
MP:0003447	decreased tumor growth/size	0.38
ENSG00000148248	SURF4 PPI subnetwork	0.38
ENSG00000104131	EIF3J PPI subnetwork	0.38
GO:0051056	regulation of small GTPase mediated signal transduction	0.38
ENSG00000124571	XPO5 PPI subnetwork	0.38
MP:0004527	abnormal outer hair cell stereociliary bundle morphology	0.38
REACTOME_PROSTANOID_METABOLISM	REACTOME_PROSTANOID_METABOLISM	0.38
ENSG00000121022	COPS5 PPI subnetwork	0.38
ENSG00000153233	PTPRR PPI subnetwork	0.38
ENSG00000115761	NOL10 PPI subnetwork	0.39
MP:0000035	abnormal membranous labyrinth morphology	0.39
ENSG00000172379	ARNT2 PPI subnetwork	0.39
ENSG00000142539	SPIB PPI subnetwork	0.39
ENSG00000184937	WT1 PPI subnetwork	0.39
GO:0005319	lipid transporter activity	0.39
ENSG00000170860	LSM3 PPI subnetwork	0.39
ENSG00000164400	CSF2 PPI subnetwork	0.39
ENSG00000161647	MPP3 PPI subnetwork	0.39
GO:0005154	epidermal growth factor receptor binding	0.39
ENSG00000109906	ZBTB16 PPI subnetwork	0.39
ENSG00000141252	VPS53 PPI subnetwork	0.39
REACTOME_MAPK_TARGETS_NUCLEAR_EVENTS_MEDIATED_BY_MAP_KINASES	REACTOME_MAPK_TARGETS_NUCLEAR_EVENTS_MEDIATED_BY_MAP_KINASES	0.39
GO:0050731	positive regulation of peptidyl-tyrosine phosphorylation	0.39
GO:0051693	actin filament capping	0.39
REACTOME_DIABETES_PATHWAYS	REACTOME_DIABETES_PATHWAYS	0.39
GO:0005583	fibrillar collagen	0.39
ENSG00000152583	SPARCL1 PPI subnetwork	0.39
ENSG00000129625	REEP5 PPI subnetwork	0.39
ENSG00000109819	PPARGC1A PPI subnetwork	0.39
ENSG00000124783	SSR1 PPI subnetwork	0.39
ENSG00000215719	ENSG00000215719 PPI subnetwork	0.39
ENSG00000205155	PSENEN PPI subnetwork	0.39
MP:0008725	enlarged heart atrium	0.39
GO:0035295	tube development	0.39
MP:0010029	abnormal basicranium morphology	0.39
ENSG00000181656	GPR88 PPI subnetwork	0.39
ENSG00000011405	PIK3C2A PPI subnetwork	0.39
MP:0004136	abnormal tongue muscle morphology	0.39
GO:0015175	neutral amino acid transmembrane transporter activity	0.39
ENSG00000179715	FAM113B PPI subnetwork	0.39
MP:0001651	necrosis	0.39

Original gene set ID	Original gene set description	Nominal P value
REACTOME_GRB2SOS_PROVIDES_LINKAGE_TO_MAPK_SIGNALING_FOR_INTER(	REACTOME_GRB2SOS_PROVIDES_LINKAGE_TO_MAPK_SIGNALING_FOR_INTERGRII	0.39
GO:0007608	sensory perception of smell	0.39
MP:0000220	increased monocyte cell number	0.39
GO:0022604	regulation of cell morphogenesis	0.39
MP:0002407	abnormal double-negative T cell morphology	0.39
GO:0030834	regulation of actin filament depolymerization	0.39
GO:0033522	histone H2A ubiquitination	0.39
MP:0000039	abnormal otic capsule morphology	0.39
ENSG00000116213	WRAP73 PPI subnetwork	0.39
MP:0003123	paternal imprinting	0.39
MP:0006355	abnormal sixth branchial arch artery morphology	0.39
MP:0011104	partial embryonic lethality before implantatior	0.39
GO:0032868	response to insulin stimulus	0.39
GO:0051303	establishment of chromosome localization	0.39
GO:0050000	chromosome localization	0.39
GO:0051875	pigment granule localization	0.39
GO:0007031	peroxisome organization	0.39
ENSG00000010803	SCMH1 PPI subnetwork	0.39
GO:0001750	photoreceptor outer segment	0.39
GO:0030674	protein binding, bridging	0.39
GO:0016628	oxidoreductase activity, acting on the CH-CH group of donors, NAD or NADP as acce	0.39
MP:0008584	photoreceptor outer segment degeneration	0.39
ENSG00000063322	MED29 PPI subnetwork	0.39
MP:0000495	abnormal colon morphology	0.39
ENSG00000015475	BID PPI subnetwork	0.39
MP:0000556	abnormal hindlimb morphology	0.39
ENSG00000174804	FZD4 PPI subnetwork	0.39
ENSG00000170653	ATF7 PPI subnetwork	0.39
REACTOME_INTRINSIC_PATHWAY_FOR_APOPTOSIS	REACTOME_INTRINSIC_PATHWAY_FOR_APOPTOSIS	0.39
GO:0045580	regulation of T cell differentiatior	0.39
GO:0017046	peptide hormone binding	0.39
KEGG_STARCH_AND_SUCROSE_METABOLISM	KEGG_STARCH_AND_SUCROSE_METABOLISM	0.39
GO:0006501	C-terminal protein lipidation	0.39
ENSG00000162105	SHANK2 PPI subnetwork	0.39
ENSG00000084623	EIF3I PPI subnetwork	0.39
KEGG_MELANOGENESIS	KEGG_MELANOGENESIS	0.39
ENSG00000115947	ORC4 PPI subnetwork	0.39
ENSG00000110107	PRPF19 PPI subnetwork	0.39
MP:0002358	abnormal spleen periarteriolar lymphoid sheath morphology	0.39
ENSG00000105404	RABAC1 PPI subnetwork	0.39
GO:0016922	ligand-dependent nuclear receptor binding	0.39
ENSG00000131043	C20orf4 PPI subnetwork	0.39
MP:0002455	abnormal dendritic cell antigen presentatior	0.39
GO:0002708	positive regulation of lymphocyte mediated immunity	0.39
GO:0002705	positive regulation of leukocyte mediated immunity	0.39
ENSG00000123349	PFDN5 PPI subnetwork	0.39
MP:0005657	abnormal neural plate morphology	0.39
GO:0030672	synaptic vesicle membrane	0.39
GO:0046822	regulation of nucleocytoplasmic transport	0.39

Original gene set ID	Original gene set description	Nominal P value
GO:0002768	immune response-regulating cell surface receptor signaling pathway	0.39
ENSG00000120949	TNFRSF8 PPI subnetwork	0.39
ENSG00000189308	LIN54 PPI subnetwork	0.39
MP:0008098	decreased plasma cell number	0.39
ENSG00000101255	TRIB3 PPI subnetwork	0.39
ENSG00000108604	SMARCD2 PPI subnetwork	0.39
REACTOME_FGFR_LIGAND_BINDING_AND_ACTIVATION	REACTOME_FGFR_LIGAND_BINDING_AND_ACTIVATION	0.39
GO:0016573	histone acetylation	0.39
ENSG00000168283	BMI1 PPI subnetwork	0.39
GO:0006518	peptide metabolic process	0.39
GO:0001909	leukocyte mediated cytotoxicity	0.39
GO:0005980	glycogen catabolic process	0.39
ENSG00000198176	TFDP1 PPI subnetwork	0.39
GO:0019079	viral genome replication	0.39
ENSG00000198301	SDAD1 PPI subnetwork	0.39
GO:0016877	ligase activity, forming carbon-sulfur bonds	0.39
GO:0002694	regulation of leukocyte activation	0.39
ENSG00000196535	MYO18A PPI subnetwork	0.39
GO:0033189	response to vitamin A	0.39
MP:0002801	abnormal long term object recognition memory	0.39
GO:0005763	mitochondrial small ribosomal subunit	0.39
GO:0000314	organellar small ribosomal subunit	0.39
ENSG00000122140	MRPS2 PPI subnetwork	0.39
GO:0006401	RNA catabolic process	0.39
ENSG00000102900	NUP93 PPI subnetwork	0.39
ENSG00000120697	ALG5 PPI subnetwork	0.39
ENSG00000068903	SIRT2 PPI subnetwork	0.39
ENSG00000175482	POLD4 PPI subnetwork	0.39
ENSG00000154518	ATP5G3 PPI subnetwork	0.39
GO:0008630	DNA damage response, signal transduction resulting in induction of apoptosis	0.39
MP:0000961	abnormal dorsal root ganglion morphology	0.39
ENSG00000166582	CENPV PPI subnetwork	0.39
MP:0002211	abnormal primary sex determination	0.39
GO:0006839	mitochondrial transport	0.39
ENSG00000103319	EEF2K PPI subnetwork	0.39
ENSG00000177602	GSG2 PPI subnetwork	0.39
GO:0030705	cytoskeleton-dependent intracellular transport	0.39
ENSG00000164776	PHKG1 PPI subnetwork	0.39
GO:0048284	organelle fusion	0.39
ENSG00000168495	POLR3D PPI subnetwork	0.39
ENSG00000090020	SLC9A1 PPI subnetwork	0.39
ENSG00000132341	RAN PPI subnetwork	0.39
MP:0001005	abnormal retinal rod cell morphology	0.39
REACTOME_ACETYLCHOLINE_NEUROTRANSMITTER_RELEASE_CYCLE	REACTOME_ACETYLCHOLINE_NEUROTRANSMITTER_RELEASE_CYCLE	0.39
MP:0008056	abnormal retinal ganglion cell morphology	0.39
KEGG_DNA_REPLICATION	KEGG_DNA_REPLICATION	0.39
MP:0003105	abnormal heart atrium morphology	0.39
GO:0015838	betaine transport	0.39
GO:0015879	carnitine transport	0.39

Original gene set ID	Original gene set description	Nominal P value
ENSG00000108797	CNTNAP1 PPI subnetwork	0.39
MP:0000743	muscle spasm	0.39
ENSG00000175216	CKAP5 PPI subnetwork	0.39
GO:0040017	positive regulation of locomotion	0.39
ENSG00000183943	PRKX PPI subnetwork	0.39
GO:0045880	positive regulation of smoothened signaling pathway	0.39
MP:0004567	decreased myocardial fiber number	0.39
MP:0002599	increased mean platelet volume	0.4
GO:0008305	integrin complex	0.4
ENSG00000174125	TLR1 PPI subnetwork	0.4
MP:0008596	increased circulating interleukin-6 level	0.4
MP:0002258	abnormal cricoid cartilage morphology	0.4
GO:0043304	regulation of mast cell degranulation	0.4
ENSG00000172795	DCP2 PPI subnetwork	0.4
ENSG00000105649	RAB3A PPI subnetwork	0.4
REACTOME_CENTROSOME_MATURATION	REACTOME_CENTROSOME_MATURATION	0.4
REACTOME_RECRUITMENT_OF_MITOTIC_CENTROSOME_PROTEINS_AND_COM	REACTOME_RECRUITMENT_OF_MITOTIC_CENTROSOME_PROTEINS_AND_COMPLE	0.4
GO:0030218	erythrocyte differentiation	0.4
MP:0000717	abnormal lymphocyte cell number	0.4
GO:0034138	toll-like receptor 3 signaling pathway	0.4
REACTOME_G2M_TRANSITION	REACTOME_G2M_TRANSITION	0.4
GO:0045787	positive regulation of cell cycle	0.4
GO:0030010	establishment of cell polarity	0.4
KEGG_VEGF_SIGNALING_PATHWAY	KEGG_VEGF_SIGNALING_PATHWAY	0.4
MP:0001385	pup cannibalization	0.4
GO:0033002	muscle cell proliferation	0.4
GO:0061061	muscle structure development	0.4
ENSG00000112306	RPS12 PPI subnetwork	0.4
GO:0007423	sensory organ development	0.4
GO:0009067	aspartate family amino acid biosynthetic process	0.4
GO:0005730	nucleolus	0.4
REACTOME_INFLUENZA_INFECTION	REACTOME_INFLUENZA_INFECTION	0.4
KEGG_BLADDER_CANCER	KEGG_BLADDER_CANCER	0.4
ENSG00000156508	EEF1A1 PPI subnetwork	0.4
ENSG00000004897	CDC27 PPI subnetwork	0.4
ENSG00000077080	ACTL6B PPI subnetwork	0.4
KEGG_METABOLISM_OF_XENOBIOTICS_BY_CYTOCHROME_P450	KEGG_METABOLISM_OF_XENOBIOTICS_BY_CYTOCHROME_P450	0.4
GO:0032587	ruffle membrane	0.4
ENSG00000115310	RTN4 PPI subnetwork	0.4
GO:0003208	cardiac ventricle morphogenesis	0.4
GO:0002697	regulation of immune effector process	0.4
ENSG00000141456	ENSG00000141456 PPI subnetwork	0.4
ENSG00000003400	CASP10 PPI subnetwork	0.4
GO:0048041	focal adhesion assembly	0.4
ENSG00000158373	HIST1H2BD PPI subnetwork	0.4
ENSG00000101246	ARFRP1 PPI subnetwork	0.4
MP:0008277	abnormal sternum ossification	0.4
ENSG00000136504	KAT7 PPI subnetwork	0.4
ENSG00000100767	PAPLN PPI subnetwork	0.4

Original gene set ID	Original gene set description	Nominal P value
GO:0042813	Wnt-activated receptor activity	0.4
REACTOME_MEIOTIC_SYNAPSIS	REACTOME_MEIOTIC_SYNAPSIS	0.4
GO:0002460	adaptive immune response based on somatic recombination of immune receptors b	0.4
ENSG00000147145	LPAR4 PPI subnetwork	0.4
GO:0032392	DNA geometric change	0.4
GO:0032508	DNA duplex unwinding	0.4
ENSG00000071462	WBSCR22 PPI subnetwork	0.4
ENSG00000182899	RPL35A PPI subnetwork	0.4
MP:0000322	increased granulocyte number	0.4
ENSG00000183751	TBL3 PPI subnetwork	0.4
MP:0009890	cleft secondary palate	0.4
ENSG00000132646	PCNA PPI subnetwork	0.4
ENSG00000100325	ASCC2 PPI subnetwork	0.4
GO:0006662	glycerol ether metabolic process	0.4
ENSG00000116750	UCHL5 PPI subnetwork	0.4
GO:0007140	male meiosis	0.4
MP:0001751	increased circulating luteinizing hormone leve	0.4
ENSG00000168040	FADD PPI subnetwork	0.4
GO:0009214	cyclic nucleotide catabolic process	0.4
REACTOME_INTERLEUKIN:2_SIGNALING	REACTOME_INTERLEUKIN:2_SIGNALING	0.4
GO:0050865	regulation of cell activation	0.4
MP:0004769	abnormal synaptic vesicle morphology	0.4
ENSG00000133026	MYH10 PPI subnetwork	0.4
ENSG00000058600	POLR3E PPI subnetwork	0.4
ENSG00000137574	TGS1 PPI subnetwork	0.4
ENSG00000165806	CASP7 PPI subnetwork	0.4
MP:0006092	abnormal olfactory neuron morphology	0.4
MP:0008045	decreased NK cell number	0.4
ENSG00000163586	FABP1 PPI subnetwork	0.4
MP:0004042	decreased susceptibility to kidney reperfusion injury	0.4
GO:0043010	camera-type eye development	0.4
MP:0005014	increased B cell number	0.4
GO:0044441	cilium part	0.4
MP:0002260	abnormal thyroid cartilage morphology	0.4
GO:0003333	amino acid transmembrane transport	0.4
ENSG00000196136	SERPINA3 PPI subnetwork	0.4
GO:0015294	solute:cation symporter activity	0.4
ENSG00000109458	GAB1 PPI subnetwork	0.4
MP:0009146	abnormal pancreatic acinar cell morphology	0.4
ENSG00000067177	PHKA1 PPI subnetwork	0.4
MP:0000820	abnormal choroid plexus morphology	0.4
GO:0050853	B cell receptor signaling pathway	0.4
GO:0003407	neural retina development	0.4
ENSG00000116001	TIA1 PPI subnetwork	0.4
ENSG00000187790	FANCM PPI subnetwork	0.4
GO:0007435	salivary gland morphogenesis	0.4
ENSG00000065183	WDR3 PPI subnetwork	0.4
ENSG00000177600	RPLP2 PPI subnetwork	0.4
GO:0005242	inward rectifier potassium channel activity	0.4

Original gene set ID	Original gene set description	Nominal P value
ENSG00000204628	GNB2L1 PPI subnetwork	0.4
ENSG00000142156	COL6A1 PPI subnetwork	0.4
GO:0017022	myosin binding	0.4
ENSG00000141026	MED9 PPI subnetwork	0.4
GO:0016502	nucleotide receptor activity	0.4
GO:0001614	purinergic nucleotide receptor activity	0.4
ENSG00000117450	PRDX1 PPI subnetwork	0.4
MP:0002168	other aberrant phenotype	0.4
ENSG00000123358	NR4A1 PPI subnetwork	0.4
GO:0044247	cellular polysaccharide catabolic process	0.4
GO:0009251	glucan catabolic process	0.4
ENSG00000129282	MRM1 PPI subnetwork	0.4
ENSG00000196277	GRM7 PPI subnetwork	0.4
ENSG00000124762	CDKN1A PPI subnetwork	0.4
ENSG00000010671	BTK PPI subnetwork	0.4
ENSG00000159189	C1QC PPI subnetwork	0.4
GO:0030042	actin filament depolymerization	0.4
ENSG00000130312	MRPL34 PPI subnetwork	0.4
GO:0021799	cerebral cortex radially oriented cell migration	0.4
MP:0000531	right pulmonary isomerism	0.4
ENSG00000197283	SYNGAP1 PPI subnetwork	0.4
GO:0030496	midbody	0.4
ENSG00000140396	NCOA2 PPI subnetwork	0.4
GO:0017002	activin-activated receptor activity	0.4
MP:0002116	abnormal craniofacial bone morphology	0.4
GO:0002824	positive regulation of adaptive immune response based on somatic recombination c	0.4
GO:0032653	regulation of interleukin-10 production	0.4
MP:0001633	poor circulation	0.4
ENSG00000061676	NCKAP1 PPI subnetwork	0.4
ENSG00000100170	SLC5A1 PPI subnetwork	0.4
GO:0032401	establishment of melanosome localization	0.4
MP:0008586	disorganized photoreceptor outer segment	0.4
GO:0005099	Ras GTPase activator activity	0.4
ENSG00000162367	TAL1 PPI subnetwork	0.4
GO:0001540	beta-amyloid binding	0.4
GO:0032355	response to estradiol stimulus	0.4
MP:0003674	oxidative stress	0.4
ENSG00000118785	SPP1 PPI subnetwork	0.4
GO:0035254	glutamate receptor binding	0.4
GO:0006605	protein targeting	0.4
GO:0000795	synaptonemal complex	0.4
MP:0004617	sacral vertebral transformation	0.4
ENSG00000124635	HIST1H2BJ PPI subnetwork	0.4
ENSG00000067048	DDX3Y PPI subnetwork	0.4
ENSG00000152684	PELO PPI subnetwork	0.4
GO:0035383	thioester metabolic process	0.4
GO:0006637	acyl-CoA metabolic process	0.4
MP:0004837	abnormal neural fold formation	0.4
GO:0030057	desmosome	0.4

Original gene set ID	Original gene set description	Nominal P value
ENSG00000196470	SIAH1 PPI subnetwork	0.4
ENSG00000133961	NUMB PPI subnetwork	0.4
GO:0046873	metal ion transmembrane transporter activity	0.4
MP:0009888	palatal shelves fail to meet at midline	0.41
ENSG00000186951	PPARA PPI subnetwork	0.41
GO:0006509	membrane protein ectodomain proteolysis	0.41
ENSG00000114030	KPNA1 PPI subnetwork	0.41
ENSG00000184588	PDE4B PPI subnetwork	0.41
ENSG00000110330	BIRC2 PPI subnetwork	0.41
ENSG00000144891	AGTR1 PPI subnetwork	0.41
GO:0051216	cartilage development	0.41
ENSG00000101343	CRNKL1 PPI subnetwork	0.41
MP:0001861	lung inflammation	0.41
MP:0010766	abnormal NK cell physiology	0.41
MP:0009115	abnormal fat cell morphology	0.41
ENSG00000166128	RAB8B PPI subnetwork	0.41
ENSG00000149925	ALDOA PPI subnetwork	0.41
ENSG00000112249	ASCC3 PPI subnetwork	0.41
GO:0035267	NuA4 histone acetyltransferase complex	0.41
GO:0045745	positive regulation of G-protein coupled receptor protein signaling pathway	0.41
GO:0006476	protein deacetylation	0.41
ENSG00000111725	PRKAB1 PPI subnetwork	0.41
ENSG00000121031	ENSG00000121031 PPI subnetwork	0.41
ENSG00000204389	HSPA1A PPI subnetwork	0.41
ENSG00000204388	HSPA1B PPI subnetwork	0.41
ENSG00000212866	HSPA1B PPI subnetwork	0.41
ENSG00000215292	ENSG00000215292 PPI subnetwork	0.41
ENSG00000212860	ENSG00000212860 PPI subnetwork	0.41
GO:0048009	insulin-like growth factor receptor signaling pathway	0.41
REACTOME_BASIGIN_INTERACTIONS	REACTOME_BASIGIN_INTERACTIONS	0.41
GO:0005871	kinesin complex	0.41
MP:0006072	abnormal retinal apoptosis	0.41
ENSG00000131381	ZFYVE20 PPI subnetwork	0.41
ENSG00000102606	ARHGEF7 PPI subnetwork	0.41
MP:0004174	abnormal spine curvature	0.41
GO:0031231	intrinsic to peroxisomal membrane	0.41
GO:0005779	integral to peroxisomal membrane	0.41
GO:0008509	anion transmembrane transporter activity	0.41
GO:0003231	cardiac ventricle development	0.41
MP:0005282	decreased fatty acid level	0.41
ENSG00000134243	SORT1 PPI subnetwork	0.41
MP:0006410	abnormal common myeloid progenitor cell morphology	0.41
MP:0003886	abnormal embryonic epiblast morphology	0.41
GO:0000083	regulation of transcription involved in G1/S phase of mitotic cell cycle	0.41
MP:0004509	abnormal pelvic girdle bone morphology	0.41
ENSG00000131910	NR0B2 PPI subnetwork	0.41
ENSG00000109971	HSPA8 PPI subnetwork	0.41
ENSG00000131236	CAP1 PPI subnetwork	0.41
REACTOME_AMINO_ACID_TRANSPORT_ACROSS_THE_PLASMA_MEMBRANE	REACTOME_AMINO_ACID_TRANSPORT_ACROSS_THE_PLASMA_MEMBRANE	0.41

Original gene set ID	Original gene set description	Nominal P value
MP:0005030	absent amnion	0.41
GO:0032098	regulation of appetite	0.41
ENSG00000183072	NKX2-5 PPI subnetwork	0.41
MP:0001469	abnormal contextual conditioning behavior	0.41
ENSG00000140285	FGF7 PPI subnetwork	0.41
GO:0005793	endoplasmic reticulum-Golgi intermediate compartment	0.41
ENSG00000196501	ENSG00000196501 PPI subnetwork	0.41
GO:0072376	protein activation cascade	0.41
GO:0032461	positive regulation of protein oligomerization	0.41
ENSG00000197597	ENSG00000197597 PPI subnetwork	0.41
MP:0004981	decreased neuronal precursor cell number	0.41
ENSG00000105229	PIAS4 PPI subnetwork	0.41
GO:0002756	MyD88-independent toll-like receptor signaling pathway	0.41
GO:0019867	outer membrane	0.41
GO:0043113	receptor clustering	0.41
MP:0000351	increased cell proliferation	0.41
GO:0050663	cytokine secretion	0.41
ENSG00000103351	CLUAP1 PPI subnetwork	0.41
ENSG00000113643	RARS PPI subnetwork	0.41
GO:0005768	endosome	0.41
GO:0019318	hexose metabolic process	0.41
MP:0008210	increased mature B cell number	0.41
GO:0071897	DNA biosynthetic process	0.41
GO:0016328	lateral plasma membrane	0.41
ENSG00000174197	MGA PPI subnetwork	0.41
ENSG00000170004	CHD3 PPI subnetwork	0.41
ENSG00000164045	CDC25A PPI subnetwork	0.41
ENSG00000206450	HLA-B PPI subnetwork	0.41
ENSG00000174227	PIGG PPI subnetwork	0.41
GO:0015758	glucose transport	0.41
GO:0008645	hexose transport	0.41
ENSG00000111640	GAPDH PPI subnetwork	0.41
ENSG00000154229	PRKCA PPI subnetwork	0.41
GO:0002761	regulation of myeloid leukocyte differentiation	0.41
ENSG00000072832	CRMP1 PPI subnetwork	0.41
MP:0002795	dilated cardiomyopathy	0.41
ENSG00000163466	ARPC2 PPI subnetwork	0.41
MP:0009746	enhanced behavioral response to xenobiotic	0.41
GO:0048771	tissue remodeling	0.41
ENSG00000135341	MAP3K7 PPI subnetwork	0.41
ENSG00000108094	CUL2 PPI subnetwork	0.41
GO:0009225	nucleotide-sugar metabolic process	0.41
ENSG00000198899	MT-ATP6 PPI subnetwork	0.41
ENSG00000070010	UFD1L PPI subnetwork	0.41
ENSG00000143878	RHOB PPI subnetwork	0.41
GO:0046930	pore complex	0.41
ENSG00000087111	PIGS PPI subnetwork	0.41
GO:0000082	G1/S transition of mitotic cell cycle	0.41
ENSG00000105401	CDC37 PPI subnetwork	0.41

Original gene set ID	Original gene set description	Nominal P value
MP:0000048	abnormal stria vascularis morphology	0.41
GO:0006516	glycoprotein catabolic process	0.41
ENSG00000108688	CCL7 PPI subnetwork	0.41
ENSG00000102024	PLS3 PPI subnetwork	0.41
GO:0001046	core promoter sequence-specific DNA binding	0.41
GO:0060326	cell chemotaxis	0.41
ENSG00000142798	HSPG2 PPI subnetwork	0.41
ENSG00000197448	GSTK1 PPI subnetwork	0.41
ENSG00000171723	GPHN PPI subnetwork	0.41
ENSG00000101336	HCK PPI subnetwork	0.41
GO:0061180	mammary gland epithelium development	0.41
MP:0001178	pulmonary hypoplasia	0.41
GO:0001503	ossification	0.41
MP:0000934	abnormal telencephalon development	0.41
ENSG00000109534	GAR1 PPI subnetwork	0.41
GO:0002758	innate immune response-activating signal transduction	0.41
ENSG00000122034	GTF3A PPI subnetwork	0.41
REACTOME_NGF_SIGNALLING_VIA_TRKA_FROM_THE_PLASMA_MEMBRANE	REACTOME_NGF_SIGNALLING_VIA_TRKA_FROM_THE_PLASMA_MEMBRANE	0.41
GO:0008361	regulation of cell size	0.41
GO:2000243	positive regulation of reproductive process	0.41
GO:0030168	platelet activation	0.41
ENSG00000130741	EIF2S3 PPI subnetwork	0.41
ENSG00000177425	PAWR PPI subnetwork	0.41
GO:0032526	response to retinoic acid	0.41
GO:0060323	head morphogenesis	0.41
GO:0048199	vesicle targeting, to, from or within Golgi	0.41
ENSG00000101849	TBL1X PPI subnetwork	0.41
MP:0004187	cardia bifida	0.41
ENSG00000142937	RPS8 PPI subnetwork	0.42
KEGG_DRUG_METABOLISM_OTHER_ENZYMES	KEGG_DRUG_METABOLISM_OTHER_ENZYMES	0.42
MP:0000846	abnormal medulla oblongata morphology	0.42
ENSG00000121486	TRMT1L PPI subnetwork	0.42
REACTOME_DEVELOPMENTAL_BIOLOGY	REACTOME_DEVELOPMENTAL_BIOLOGY	0.42
GO:0033993	response to lipid	0.42
GO:0046915	transition metal ion transmembrane transporter activity	0.42
ENSG00000148408	CACNA1B PPI subnetwork	0.42
ENSG00000171603	CLSTN1 PPI subnetwork	0.42
GO:0034101	erythrocyte homeostasis	0.42
MP:0005416	abnormal circulating protein level	0.42
GO:0045620	negative regulation of lymphocyte differentiation	0.42
MP:0000062	increased bone mineral density	0.42
MP:0002840	abnormal lens fiber morphology	0.42
ENSG00000184226	PCDH9 PPI subnetwork	0.42
ENSG00000136842	TMOD1 PPI subnetwork	0.42
ENSG00000136938	ANP32B PPI subnetwork	0.42
ENSG00000157933	SKI PPI subnetwork	0.42
MP:0008214	increased immature B cell number	0.42
MP:0009172	small pancreatic islets	0.42
ENSG00000000971	CFH PPI subnetwork	0.42

Original gene set ID	Original gene set description	Nominal P value
ENSG00000117020	AKT3 PPI subnetwork	0.42
GO:0033344	cholesterol efflux	0.42
ENSG00000153006	SREK1IP1 PPI subnetwork	0.42
GO:0005774	vacuolar membrane	0.42
MP:0008617	increased circulating interleukin-12 level	0.42
ENSG00000156711	MAPK13 PPI subnetwork	0.42
MP:0003871	abnormal myelin sheath morphology	0.42
ENSG00000118965	WDR35 PPI subnetwork	0.42
GO:0005104	fibroblast growth factor receptor binding	0.42
MP:0001940	testis hypoplasia	0.42
ENSG00000138468	SENP7 PPI subnetwork	0.42
ENSG00000118640	VAMP8 PPI subnetwork	0.42
ENSG00000105663	ENSG00000105663 PPI subnetwork	0.42
MP:0000292	distended pericardium	0.42
MP:0001790	abnormal immune system physiology	0.42
MP:0001554	increased circulating free fatty acid leve	0.42
GO:0030099	myeloid cell differentiation	0.42
MP:0003633	abnormal nervous system physiology	0.42
GO:0016053	organic acid biosynthetic process	0.42
GO:0046394	carboxylic acid biosynthetic process	0.42
MP:0004696	abnormal thyroid follicle morphology	0.42
ENSG00000100353	EIF3D PPI subnetwork	0.42
GO:0051153	regulation of striated muscle cell differentiation	0.42
ENSG00000196611	MMP1 PPI subnetwork	0.42
ENSG00000213024	NUP62 PPI subnetwork	0.42
ENSG00000118007	STAG1 PPI subnetwork	0.42
ENSG00000106554	CHCHD3 PPI subnetwork	0.42
ENSG00000080608	KIAA0020 PPI subnetwork	0.42
GO:0031110	regulation of microtubule polymerization or depolymerization	0.42
GO:0035384	thioester biosynthetic process	0.42
GO:0071616	acyl-CoA biosynthetic process	0.42
REACTOME_IRS:RELATED_EVENTS	REACTOME_IRS:RELATED_EVENTS	0.42
REACTOME_IRS:MEDIATED_SIGNALLING	REACTOME_IRS:MEDIATED_SIGNALLING	0.42
GO:0042559	pteridine-containing compound biosynthetic process	0.42
REACTOME_METABOLISM_OF_RNA	REACTOME_METABOLISM_OF_RNA	0.42
GO:0005529	GO:0005529	0.42
ENSG00000089902	RCOR1 PPI subnetwork	0.42
MP:0009404	centrally nucleated skeletal muscle fibers	0.42
REACTOME_ASSOCIATION_OF_TRICCCT_WITH_TARGET_PROTEINS_DURING_BI	REACTOME_ASSOCIATION_OF_TRICCCT_WITH_TARGET_PROTEINS_DURING_BIOSY	0.42
ENSG00000146587	RBAK PPI subnetwork	0.42
ENSG00000081248	CACNA1S PPI subnetwork	0.42
ENSG00000007402	CACNA2D2 PPI subnetwork	0.42
MP:0000822	abnormal brain ventricle morphology	0.42
GO:0045776	negative regulation of blood pressure	0.42
GO:0009266	response to temperature stimulus	0.42
GO:0006940	regulation of smooth muscle contraction	0.42
GO:0031111	negative regulation of microtubule polymerization or depolymerization	0.42
MP:0002118	abnormal lipid homeostasis	0.42
ENSG00000177565	TBL1XR1 PPI subnetwork	0.42

Original gene set ID	Original gene set description	Nominal P value
ENSG00000182944	EWSR1 PPI subnetwork	0.42
ENSG00000173349	SFT2D3 PPI subnetwork	0.42
GO:0034694	response to prostaglandin stimulus	0.42
ENSG00000090776	EFNB1 PPI subnetwork	0.42
ENSG00000102010	BMX PPI subnetwork	0.42
ENSG00000100385	IL2RB PPI subnetwork	0.42
GO:0006626	protein targeting to mitochondrion	0.42
ENSG00000138297	TIMM23 PPI subnetwork	0.42
ENSG00000172399	MYOZ2 PPI subnetwork	0.42
GO:0051318	G1 phase	0.42
GO:0016229	steroid dehydrogenase activity	0.42
ENSG00000108651	UTP6 PPI subnetwork	0.42
MP:0000740	impaired smooth muscle contractility	0.42
MP:0000477	abnormal intestine morphology	0.42
MP:0000222	decreased neutrophil cell number	0.42
ENSG00000164167	LSM6 PPI subnetwork	0.42
REACTOME_SIGNALING_BY_ERBB4	REACTOME_SIGNALING_BY_ERBB4	0.42
MP:0000088	short mandible	0.42
ENSG00000136628	EPRS PPI subnetwork	0.42
ENSG00000164171	ITGA2 PPI subnetwork	0.42
ENSG00000182809	CRIP2 PPI subnetwork	0.42
ENSG00000149091	DGKZ PPI subnetwork	0.42
GO:0032787	monocarboxylic acid metabolic process	0.42
GO:0003684	damaged DNA binding	0.42
REACTOME_PROCESSIVE_SYNTHESIS_ON_THE_LAGGING_STRAND	REACTOME_PROCESSIVE_SYNTHESIS_ON_THE_LAGGING_STRAND	0.42
GO:0030660	Golgi-associated vesicle membrane	0.42
REACTOME_RECYCLING_OF_BILE_ACIDS_AND_SALTS	REACTOME_RECYCLING_OF_BILE_ACIDS_AND_SALTS	0.42
ENSG00000196712	NF1 PPI subnetwork	0.42
GO:0007260	tyrosine phosphorylation of STAT protein	0.42
ENSG00000160094	ZNF362 PPI subnetwork	0.42
GO:0007517	muscle organ development	0.42
GO:0035094	response to nicotine	0.42
ENSG00000131508	UBE2D2 PPI subnetwork	0.42
GO:0017075	syntaxin-1 binding	0.42
ENSG00000166889	PATL1 PPI subnetwork	0.42
ENSG00000163554	SPTA1 PPI subnetwork	0.43
MP:0001327	decreased retinal photoreceptor cell number	0.43
GO:0001047	core promoter binding	0.43
GO:0000086	G2/M transition of mitotic cell cycle	0.43
ENSG00000127955	GNAI1 PPI subnetwork	0.43
ENSG00000078900	TP73 PPI subnetwork	0.43
GO:0035601	protein deacylation	0.43
REACTOME_INTRINSIC_PATHWAY	REACTOME_INTRINSIC_PATHWAY	0.43
GO:0035329	hippo signaling cascade	0.43
GO:0034134	toll-like receptor 2 signaling pathway	0.43
MP:0003122	maternal imprinting	0.43
GO:0006338	chromatin remodeling	0.43
GO:0060443	mammary gland morphogenesis	0.43
ENSG00000100485	SOS2 PPI subnetwork	0.43

Original gene set ID	Original gene set description	Nominal P value
GO:0005540	hyaluronic acid binding	0.43
MP:0004678	split xiphoid process	0.43
REACTOME_GENERIC_TRANSCRIPTION_PATHWAY	REACTOME_GENERIC_TRANSCRIPTION_PATHWAY	0.43
GO:0008214	protein dealkylation	0.43
GO:0006482	protein demethylation	0.43
GO:0010565	regulation of cellular ketone metabolic process	0.43
ENSG00000148143	ZNF462 PPI subnetwork	0.43
GO:0051310	metaphase plate congression	0.43
ENSG00000184985	SORCS2 PPI subnetwork	0.43
MP:0000428	abnormal craniofacial morphology	0.43
ENSG00000123975	CKS2 PPI subnetwork	0.43
ENSG00000156136	DCK PPI subnetwork	0.43
ENSG00000160200	CBS PPI subnetwork	0.43
REACTOME_PI:3K_CASCADE	REACTOME_PI:3K_CASCADE	0.43
MP:0002412	increased susceptibility to bacterial infection	0.43
GO:0090100	positive regulation of transmembrane receptor protein serine/threonine kinase sign	0.43
ENSG00000066044	ELAVL1 PPI subnetwork	0.43
ENSG00000104689	TNFRSF10A PPI subnetwork	0.43
ENSG00000112237	CCNC PPI subnetwork	0.43
ENSG00000197943	PLCG2 PPI subnetwork	0.43
REACTOME_DOWNSTREAM_SIGNALING_OF_ACTIVATED_FGFR	REACTOME_DOWNSTREAM_SIGNALING_OF_ACTIVATED_FGFR	0.43
ENSG00000043591	ADRB1 PPI subnetwork	0.43
REACTOME_GENERATION_OF_SECOND_MESSENGER_MOLECULES	REACTOME_GENERATION_OF_SECOND_MESSENGER_MOLECULES	0.43
REACTOME_INFLUENZA_LIFE_CYCLE	REACTOME_INFLUENZA_LIFE_CYCLE	0.43
GO:0016763	transferase activity, transferring pentosyl groups	0.43
GO:0014812	muscle cell migration	0.43
ENSG00000124782	RREB1 PPI subnetwork	0.43
ENSG00000113356	POLR3G PPI subnetwork	0.43
MP:0001395	bidirectional circling	0.43
GO:0002718	regulation of cytokine production involved in immune response	0.43
REACTOME_NEGATIVE_REGULATION_OF_FGFR_SIGNALING	REACTOME_NEGATIVE_REGULATION_OF_FGFR_SIGNALING	0.43
ENSG00000106105	GARS PPI subnetwork	0.43
GO:0001522	pseudouridine synthesis	0.43
ENSG00000129315	CCNT1 PPI subnetwork	0.43
GO:0002250	adaptive immune response	0.43
REACTOME_PI3K_CASCADE	REACTOME_PI3K_CASCADE	0.43
GO:0030141	secretory granule	0.43
MP:0003139	patent ductus arteriosus	0.43
ENSG00000100387	RBX1 PPI subnetwork	0.43
GO:0007213	G-protein coupled acetylcholine receptor signaling pathway	0.43
ENSG00000145220	LYAR PPI subnetwork	0.43
GO:0030665	clathrin coated vesicle membrane	0.43
MP:0006395	abnormal epiphyseal plate morphology	0.43
KEGG_GLYCOSYLPHOSPHATIDYLINOSITOL_GPI_ANCHOR_BIOSYNTHESIS	KEGG_GLYCOSYLPHOSPHATIDYLINOSITOL_GPI_ANCHOR_BIOSYNTHESIS	0.43
ENSG00000142166	IFNAR1 PPI subnetwork	0.43
ENSG00000130826	DKC1 PPI subnetwork	0.43
GO:0004197	cysteine-type endopeptidase activity	0.43
ENSG00000124217	MOCS3 PPI subnetwork	0.43
GO:0022612	gland morphogenesis	0.43

Original gene set ID	Original gene set description	Nominal P value
MP:0000157	abnormal sternum morphology	0.43
ENSG00000173473	SMARCC1 PPI subnetwork	0.43
MP:0000837	abnormal hypothalamus morphology	0.43
MP:0011094	complete embryonic lethality before implantation	0.43
MP:0001614	abnormal blood vessel morphology	0.43
ENSG00000117385	LEPRE1 PPI subnetwork	0.43
MP:0003725	increased autoantibody level	0.43
ENSG00000109320	NFKB1 PPI subnetwork	0.43
GO:0002407	dendritic cell chemotaxis	0.43
ENSG00000105220	GPI PPI subnetwork	0.43
MP:0010872	increased trabecular bone mass	0.43
MP:0008682	decreased interleukin-17 secretion	0.43
MP:0005606	increased bleeding time	0.43
GO:0045839	negative regulation of mitosis	0.43
GO:0051784	negative regulation of nuclear division	0.43
MP:0000136	abnormal microglial cell morphology	0.43
GO:2000311	regulation of alpha-amino-3-hydroxy-5-methyl-4-isoxazole propionate selective glut	0.43
MP:0001312	abnormal cornea morphology	0.43
ENSG00000113312	TTC1 PPI subnetwork	0.43
GO:0034329	cell junction assembly	0.43
GO:0070307	lens fiber cell development	0.43
ENSG00000099331	MYO9B PPI subnetwork	0.43
GO:0060076	excitatory synapse	0.43
ENSG00000181929	PRKAG1 PPI subnetwork	0.43
ENSG00000197961	ZNF121 PPI subnetwork	0.43
ENSG00000137876	RSL24D1 PPI subnetwork	0.43
GO:0016782	transferase activity, transferring sulfur-containing groups	0.43
MP:0002631	abnormal epididymis morphology	0.43
GO:0046006	regulation of activated T cell proliferation	0.43
MP:0004076	abnormal vitelline vascular remodeling	0.43
MP:0001800	abnormal humoral immune response	0.43
ENSG00000182367	ENSG00000182367 PPI subnetwork	0.43
MP:0003048	abnormal cervical vertebrae morphology	0.43
ENSG00000143498	TAF1A PPI subnetwork	0.43
MP:0000685	abnormal immune system morphology	0.43
MP:0005011	increased eosinophil cell number	0.43
GO:0009152	purine ribonucleotide biosynthetic process	0.43
GO:0007264	small GTPase mediated signal transduction	0.43
REACTOME_RNA_POLYMERASE_I_TRANSCRIPTION_TERMINATION	REACTOME_RNA_POLYMERASE_I_TRANSCRIPTION_TERMINATION	0.43
ENSG00000134255	CEPT1 PPI subnetwork	0.43
ENSG00000197616	MYH6 PPI subnetwork	0.43
ENSG00000139613	SMARCC2 PPI subnetwork	0.43
GO:0045028	G-protein coupled purinergic nucleotide receptor activity	0.43
GO:0001608	G-protein coupled nucleotide receptor activity	0.43
ENSG00000186153	WWOX PPI subnetwork	0.43
ENSG00000155111	CDK19 PPI subnetwork	0.43
ENSG00000163635	ATXN7 PPI subnetwork	0.43
ENSG00000154767	XPC PPI subnetwork	0.43
MP:0010019	liver vascular congestion	0.43

Original gene set ID	Original gene set description	Nominal P value
GO:0050673	epithelial cell proliferation	0.43
MP:0002574	increased vertical activity	0.43
MP:0005421	loose skin	0.43
GO:0048638	regulation of developmental growth	0.43
GO:0009116	nucleoside metabolic process	0.43
GO:0032970	regulation of actin filament-based process	0.43
ENSG00000154764	WNT7A PPI subnetwork	0.43
ENSG00000105855	ITGB8 PPI subnetwork	0.43
ENSG00000070423	RNF126 PPI subnetwork	0.43
ENSG00000124588	NQO2 PPI subnetwork	0.43
GO:0005161	platelet-derived growth factor receptor binding	0.44
ENSG00000076604	TRAF4 PPI subnetwork	0.44
MP:0003718	maternal effect	0.44
ENSG00000065485	PDIA5 PPI subnetwork	0.44
ENSG00000065135	GNAI3 PPI subnetwork	0.44
ENSG00000100227	POLDIP3 PPI subnetwork	0.44
MP:0005353	abnormal patella morphology	0.44
MP:0008501	increased IgG2b level	0.44
MP:0004029	spontaneous chromosome breakage	0.44
GO:0032613	interleukin-10 production	0.44
MP:0000182	increased circulating LDL cholesterol leve	0.44
MP:0004783	abnormal cardinal vein morphology	0.44
GO:0001518	voltage-gated sodium channel complex	0.44
ENSG00000165030	NFIL3 PPI subnetwork	0.44
KEGG_P53_SIGNALING_PATHWAY	KEGG_P53_SIGNALING_PATHWAY	0.44
MP:0005463	abnormal CD4-positive T cell physiology	0.44
GO:0048738	cardiac muscle tissue development	0.44
GO:0032869	cellular response to insulin stimulus	0.44
ENSG00000105695	MAG PPI subnetwork	0.44
GO:0048246	macrophage chemotaxis	0.44
GO:0040008	regulation of growth	0.44
ENSG00000171346	KRT15 PPI subnetwork	0.44
GO:0000070	mitotic sister chromatid segregation	0.44
GO:0042129	regulation of T cell proliferation	0.44
MP:0008593	increased circulating interleukin-10 level	0.44
GO:0002478	antigen processing and presentation of exogenous peptide antiger	0.44
ENSG00000204642	HLA-F PPI subnetwork	0.44
MP:0000030	abnormal tympanic ring morphology	0.44
ENSG00000104312	RIPK2 PPI subnetwork	0.44
ENSG00000035928	RFC1 PPI subnetwork	0.44
KEGG_GLYCOLYSIS_GLUCONEOGENESIS	KEGG_GLYCOLYSIS_GLUCONEOGENESIS	0.44
ENSG00000132432	SEC61G PPI subnetwork	0.44
GO:0043484	regulation of RNA splicing	0.44
ENSG00000002745	WNT16 PPI subnetwork	0.44
MP:0006043	decreased apoptosis	0.44
ENSG00000144580	RQCD1 PPI subnetwork	0.44
ENSG00000116251	RPL22 PPI subnetwork	0.44
MP:0003131	increased erythrocyte cell number	0.44
MP:0004703	abnormal vertebral column morphology	0.44

Original gene set ID	Original gene set description	Nominal P value
GO:0044283	small molecule biosynthetic process	0.44
GO:0003281	ventricular septum development	0.44
GO:0016407	acetyltransferase activity	0.44
MP:0004154	renal tubular necrosis	0.44
MP:0008522	abnormal lymph node germinal center morphology	0.44
ENSG00000093009	CDC45 PPI subnetwork	0.44
ENSG00000105963	ADAP1 PPI subnetwork	0.44
GO:0000096	sulfur amino acid metabolic process	0.44
GO:0046578	regulation of Ras protein signal transduction	0.44
GO:0005184	neuropeptide hormone activity	0.44
GO:0005086	ARF guanyl-nucleotide exchange factor activity	0.44
ENSG00000100297	MCM5 PPI subnetwork	0.44
GO:0000079	regulation of cyclin-dependent protein kinase activity	0.44
ENSG00000089234	BRAP PPI subnetwork	0.44
GO:0046660	female sex differentiation	0.44
REACTOME_VOLTAGE_GATED_POTASSIUM_CHANNELS	REACTOME_VOLTAGE_GATED_POTASSIUM_CHANNELS	0.44
GO:0007613	memory	0.44
GO:0042531	positive regulation of tyrosine phosphorylation of STAT protein	0.44
ENSG00000158042	MRPL17 PPI subnetwork	0.44
GO:0050954	sensory perception of mechanical stimulus	0.44
MP:0004272	abnormal basement membrane morphology	0.44
GO:0005775	vacuolar lumen	0.44
GO:0008063	Toll signaling pathway	0.44
ENSG00000066379	ZNRD1 PPI subnetwork	0.44
ENSG00000206502	ZNRD1 PPI subnetwork	0.44
ENSG00000206429	ENSG00000206429 PPI subnetwork	0.44
ENSG00000106571	GLI3 PPI subnetwork	0.44
MP:0003179	decreased platelet cell number	0.44
REACTOME_NOD12_SIGNALING_PATHWAY	REACTOME_NOD12_SIGNALING_PATHWAY	0.44
ENSG00000096433	ITPR3 PPI subnetwork	0.44
MP:0001353	increased aggression towards mice	0.44
MP:0002736	abnormal nociception after inflammation	0.44
GO:0043383	negative T cell selection	0.44
ENSG00000050748	MAPK9 PPI subnetwork	0.44
ENSG00000126562	WNK4 PPI subnetwork	0.44
ENSG00000205572	SERF1B PPI subnetwork	0.44
ENSG00000172058	SERF1A PPI subnetwork	0.44
GO:0044437	vacuolar part	0.44
GO:0007183	SMAD protein complex assembly	0.44
GO:0035270	endocrine system development	0.44
KEGG_LYSOSOME	KEGG_LYSOSOME	0.44
GO:0004857	enzyme inhibitor activity	0.44
GO:0022616	DNA strand elongation	0.44
ENSG00000133103	COG6 PPI subnetwork	0.44
GO:0046460	neutral lipid biosynthetic process	0.44
GO:0046463	acylglycerol biosynthetic process	0.44
GO:2000242	negative regulation of reproductive process	0.44
GO:0030029	actin filament-based process	0.44
GO:0070888	E-box binding	0.44

Original gene set ID	Original gene set description	Nominal P value
KEGG_GLIOMA	KEGG_GLIOMA	0.44
GO:0048471	perinuclear region of cytoplasm	0.44
GO:0051494	negative regulation of cytoskeleton organizatior	0.44
GO:0070925	organelle assembly	0.44
ENSG00000137218	FRS3 PPI subnetwork	0.44
GO:0017069	snRNA binding	0.44
MP:0000688	lymphoid hyperplasia	0.44
ENSG00000171634	BPTF PPI subnetwork	0.44
GO:0017015	regulation of transforming growth factor beta receptor signaling pathwa	0.44
GO:0004714	transmembrane receptor protein tyrosine kinase activit	0.44
ENSG00000120889	TNFRSF10B PPI subnetwork	0.44
ENSG00000196975	ANXA4 PPI subnetwork	0.44
ENSG00000121858	TNFSF10 PPI subnetwork	0.44
GO:0002449	lymphocyte mediated immunity	0.44
GO:0005212	structural constituent of eye lens	0.44
GO:0048745	smooth muscle tissue development	0.44
GO:0022613	ribonucleoprotein complex biogenesis	0.44
MP:0010373	myeloid hyperplasia	0.44
MP:0000036	absent semicircular canals	0.44
ENSG00000132485	ZRANB2 PPI subnetwork	0.44
ENSG00000106123	EPHB6 PPI subnetwork	0.44
GO:0042254	ribosome biogenesis	0.44
MP:0005102	abnormal iris pigmentation	0.44
ENSG00000206156	ENSG00000206156 PPI subnetwork	0.44
ENSG00000105372	RPS19 PPI subnetwork	0.44
MP:0005140	decreased cardiac muscle contractility	0.44
ENSG00000081019	RSBN1 PPI subnetwork	0.44
ENSG00000101439	CST3 PPI subnetwork	0.44
MP:0008186	increased pro-B cell number	0.44
GO:0032400	melanosome localization	0.44
ENSG00000154143	PANX3 PPI subnetwork	0.44
GO:0046902	regulation of mitochondrial membrane permeability	0.44
GO:0000159	protein phosphatase type 2A complex	0.44
MP:0005154	increased B cell proliferation	0.44
GO:0016705	oxidoreductase activity, acting on paired donors, with incorporation or reduction of	0.44
MP:0001384	abnormal pup retrieval	0.44
GO:0014047	glutamate secretion	0.44
MP:0001939	secondary sex reversal	0.44
GO:0016641	oxidoreductase activity, acting on the CH-NH2 group of donors, oxygen as accepto	0.45
MP:0001273	decreased metastatic potential	0.45
ENSG00000071655	MBD3 PPI subnetwork	0.45
ENSG00000136854	STXBP1 PPI subnetwork	0.45
ENSG00000172071	EIF2AK3 PPI subnetwork	0.45
ENSG00000160563	MED27 PPI subnetwork	0.45
ENSG00000080802	CNOT4 PPI subnetwork	0.45
ENSG00000078018	MAP2 PPI subnetwork	0.45
GO:0007005	mitochondrion organization	0.45
ENSG00000100726	TELO2 PPI subnetwork	0.45
ENSG00000182541	LIMK2 PPI subnetwork	0.45

Original gene set ID	Original gene set description	Nominal P value
GO:0070412	R-SMAD binding	0.45
ENSG00000188739	RBM34 PPI subnetwork	0.45
GO:0035176	social behavior	0.45
ENSG00000144597	EAF1 PPI subnetwork	0.45
GO:0042987	amyloid precursor protein catabolic process	0.45
ENSG00000196226	HIST1H2BB PPI subnetwork	0.45
ENSG00000150907	FOXO1 PPI subnetwork	0.45
ENSG00000147536	GINS4 PPI subnetwork	0.45
REACTOME_ANTIGEN_PRESENTATION_FOLDING_ASSEMBLY_AND_PEPTIDE_LO	REACTOME_ANTIGEN_PRESENTATION_FOLDING_ASSEMBLY_AND_PEPTIDE_LOADII	0.45
ENSG00000117335	CD46 PPI subnetwork	0.45
ENSG00000135018	UBQLN1 PPI subnetwork	0.45
GO:0032956	regulation of actin cytoskeleton organization	0.45
MP:0001634	internal hemorrhage	0.45
ENSG00000124813	RUNX2 PPI subnetwork	0.45
GO:0043025	neuronal cell body	0.45
ENSG00000104332	SFRP1 PPI subnetwork	0.45
ENSG00000120057	SFRP5 PPI subnetwork	0.45
ENSG00000106483	SFRP4 PPI subnetwork	0.45
REACTOME_PHOSPHOLIPASE_C:MEDIATED_CASCADE	REACTOME_PHOSPHOLIPASE_C:MEDIATED_CASCADE	0.45
MP:0003360	abnormal depression-related behavior	0.45
GO:0060363	cranial suture morphogenesis	0.45
GO:0097094	craniofacial suture morphogenesis	0.45
GO:0043367	CD4-positive, alpha-beta T cell differentiation	0.45
GO:0000982	RNA polymerase II core promoter proximal region sequence-specific DNA binding tr	0.45
MP:0001719	absent vitelline blood vessels	0.45
GO:0016725	oxidoreductase activity, acting on CH or CH2 groups	0.45
ENSG00000145555	MYO10 PPI subnetwork	0.45
KEGG_ONE_CARBON_POOL_BY_FOLATE	KEGG_ONE_CARBON_POOL_BY_FOLATE	0.45
MP:0005410	abnormal fertilization	0.45
GO:0016746	transferase activity, transferring acyl groups	0.45
ENSG00000178982	EIF3K PPI subnetwork	0.45
GO:0016311	dephosphorylation	0.45
GO:0046112	nucleobase biosynthetic process	0.45
ENSG00000153187	HNRNPU PPI subnetwork	0.45
ENSG00000014138	POLA2 PPI subnetwork	0.45
ENSG00000112739	PRPF4B PPI subnetwork	0.45
ENSG00000132849	INADL PPI subnetwork	0.45
MP:0008560	increased tumor necrosis factor secretion	0.45
ENSG00000068976	PYGM PPI subnetwork	0.45
MP:0002928	abnormal bile duct morphology	0.45
GO:0031253	cell projection membrane	0.45
GO:0007163	establishment or maintenance of cell polarity	0.45
ENSG00000117528	ABCD3 PPI subnetwork	0.45
MP:0000603	pale liver	0.45
MP:0003944	abnormal T cell subpopulation ratio	0.45
MP:0002458	abnormal B cell number	0.45
ENSG00000214026	MRPL23 PPI subnetwork	0.45
ENSG00000156603	MED19 PPI subnetwork	0.45
GO:0007605	sensory perception of sound	0.45

Original gene set ID	Original gene set description	Nominal P value
ENSG00000178105	DDX10 PPI subnetwork	0.45
ENSG00000156931	VPS8 PPI subnetwork	0.45
ENSG00000141985	SH3GL1 PPI subnetwork	0.45
ENSG00000108819	ENSG00000108819 PPI subnetwork	0.45
GO:0070306	lens fiber cell differentiation	0.45
ENSG00000102144	PGK1 PPI subnetwork	0.45
GO:0006958	complement activation, classical pathway	0.45
REACTOME_LOSS_OF_PROTEINS_REQUIRED_FOR_INTERPHASE_MICROTUBULE_	REACTOME_LOSS_OF_PROTEINS_REQUIRED_FOR_INTERPHASE_MICROTUBULE_OR	0.45
REACTOME_LOSS_OF_NLP_FROM_MITOTIC_CENTROSOMES	REACTOME_LOSS_OF_NLP_FROM_MITOTIC_CENTROSOMES	0.45
GO:0032663	regulation of interleukin-2 production	0.45
GO:0035258	steroid hormone receptor binding	0.45
GO:0030178	negative regulation of Wnt receptor signaling pathway	0.45
ENSG00000166710	B2M PPI subnetwork	0.45
GO:0042058	regulation of epidermal growth factor receptor signaling pathway	0.45
GO:0031968	organelle outer membrane	0.45
ENSG00000071051	NCK2 PPI subnetwork	0.45
ENSG00000163516	ANKZF1 PPI subnetwork	0.45
REACTOME_GAP_JUNCTION_TRAFFICKING	REACTOME_GAP_JUNCTION_TRAFFICKING	0.45
ENSG00000136044	APPL2 PPI subnetwork	0.45
ENSG00000125868	DSTN PPI subnetwork	0.45
ENSG00000095564	BTAF1 PPI subnetwork	0.45
ENSG00000196890	HIST3H2BB PPI subnetwork	0.45
GO:0000793	condensed chromosome	0.45
ENSG00000101442	ACTR5 PPI subnetwork	0.45
MP:0000753	paralysis	0.45
MP:0006020	decreased tympanic ring size	0.45
GO:0006760	folic acid-containing compound metabolic process	0.45
ENSG00000165632	TAF3 PPI subnetwork	0.45
GO:0048844	artery morphogenesis	0.45
ENSG00000128739	SNRPN PPI subnetwork	0.45
GO:0006664	glycolipid metabolic process	0.45
GO:0043209	myelin sheath	0.45
ENSG00000087191	PSMC5 PPI subnetwork	0.45
ENSG00000175536	LIPT2 PPI subnetwork	0.45
GO:0014704	intercalated disc	0.45
ENSG00000106682	EIF4H PPI subnetwork	0.45
GO:0002889	regulation of immunoglobulin mediated immune response	0.45
GO:0042104	positive regulation of activated T cell proliferation	0.45
ENSG00000148377	IDI2 PPI subnetwork	0.45
GO:0043094	cellular metabolic compound salvage	0.45
ENSG00000196220	SRGAP3 PPI subnetwork	0.45
MP:0008518	retinal outer nuclear layer degeneration	0.45
ENSG00000116957	TBCE PPI subnetwork	0.45
MP:0000163	abnormal cartilage morphology	0.45
ENSG00000184009	ACTG1 PPI subnetwork	0.45
ENSG00000169032	MAP2K1 PPI subnetwork	0.45
GO:0008156	negative regulation of DNA replication	0.45
GO:0044433	cytoplasmic vesicle part	0.45
MP:0006058	decreased cerebral infarction size	0.45

Original gene set ID	Original gene set description	Nominal P value
ENSG00000172053	QARS PPI subnetwork	0.45
GO:0000959	mitochondrial RNA metabolic process	0.45
GO:0045598	regulation of fat cell differentiation	0.45
MP:0008474	absent spleen germinal center	0.45
MP:0008143	abnormal dendrite morphology	0.45
ENSG00000100784	RPS6KA5 PPI subnetwork	0.45
ENSG00000126005	ENSG00000126005 PPI subnetwork	0.45
ENSG00000069974	RAB27A PPI subnetwork	0.45
ENSG00000111669	TPI1 PPI subnetwork	0.45
KEGG_AMYOTROPHIC_LATERAL_SCLEROSIS_ALS	KEGG_AMYOTROPHIC_LATERAL_SCLEROSIS_ALS	0.45
MP:0009887	abnormal palatal shelf fusion at midline	0.45
GO:0001669	acrosomal vesicle	0.45
ENSG00000134460	IL2RA PPI subnetwork	0.45
ENSG00000166033	HTRA1 PPI subnetwork	0.45
REACTOME_CHROMOSOME_MAINTENANCE	REACTOME_CHROMOSOME_MAINTENANCE	0.46
ENSG00000099250	NRP1 PPI subnetwork	0.46
MP:0000926	absent floor plate	0.46
ENSG00000182866	LCK PPI subnetwork	0.46
ENSG00000155380	SLC16A1 PPI subnetwork	0.46
GO:0048524	positive regulation of viral reproduction	0.46
ENSG00000168066	SF1 PPI subnetwork	0.46
ENSG00000076864	RAP1GAP PPI subnetwork	0.46
MP:0001718	abnormal visceral yolk sac morphology	0.46
GO:0006363	termination of RNA polymerase I transcription	0.46
ENSG00000198612	COPS8 PPI subnetwork	0.46
MP:0001303	abnormal lens morphology	0.46
GO:0004497	monooxygenase activity	0.46
ENSG00000125484	GTF3C4 PPI subnetwork	0.46
ENSG00000197971	MBP PPI subnetwork	0.46
GO:0004519	endonuclease activity	0.46
ENSG00000105176	URI1 PPI subnetwork	0.46
GO:0004722	protein serine/threonine phosphatase activity	0.46
ENSG00000128833	MYO5C PPI subnetwork	0.46
GO:0005819	spindle	0.46
ENSG00000196540	ENSG00000196540 PPI subnetwork	0.46
GO:0017127	cholesterol transporter activity	0.46
GO:0035710	CD4-positive, alpha-beta T cell activation	0.46
GO:0042542	response to hydrogen peroxide	0.46
MP:0005438	abnormal glycogen homeostasis	0.46
ENSG00000150768	DLAT PPI subnetwork	0.46
GO:0002221	pattern recognition receptor signaling pathway	0.46
GO:0050911	detection of chemical stimulus involved in sensory perception of smel	0.46
GO:0070461	SAGA-type complex	0.46
GO:0034399	nuclear periphery	0.46
ENSG00000179071	CCDC89 PPI subnetwork	0.46
MP:0000746	weakness	0.46
ENSG00000135999	EPC2 PPI subnetwork	0.46
KEGG_PROSTATE_CANCER	KEGG_PROSTATE_CANCER	0.46
MP:0002576	abnormal enamel morphology	0.46

Original gene set ID	Original gene set description	Nominal P value
ENSG00000162735	PEX19 PPI subnetwork	0.46
ENSG00000009335	UBE3C PPI subnetwork	0.46
MP:0001675	abnormal ectoderm development	0.46
ENSG00000113558	SKP1 PPI subnetwork	0.46
ENSG00000042980	ADAM28 PPI subnetwork	0.46
MP:0001870	salivary gland inflammation	0.46
ENSG00000167461	RAB8A PPI subnetwork	0.46
ENSG00000163599	CTLA4 PPI subnetwork	0.46
GO:0060420	regulation of heart growth	0.46
MP:0002546	mydriasis	0.46
MP:0002495	increased IgA level	0.46
ENSG00000104613	INTS10 PPI subnetwork	0.46
MP:0000135	decreased compact bone thickness	0.46
ENSG00000132109	TRIM21 PPI subnetwork	0.46
GO:0034130	toll-like receptor 1 signaling pathway	0.46
ENSG00000099860	GADD45B PPI subnetwork	0.46
GO:0005769	early endosome	0.46
MP:0002184	abnormal innervation	0.46
GO:0070588	calcium ion transmembrane transport	0.46
MP:0003215	renal interstitial fibrosis	0.46
ENSG00000105048	TNNT1 PPI subnetwork	0.46
MP:0008828	abnormal lymph node cell ratio	0.46
GO:0051084	'de novo' posttranslational protein folding	0.46
GO:0008250	oligosaccharyltransferase complex	0.46
GO:0006271	DNA strand elongation involved in DNA replication	0.46
MP:0002884	abnormal branchial arch morphology	0.46
ENSG00000083520	DIS3 PPI subnetwork	0.46
ENSG00000164061	BSN PPI subnetwork	0.46
MP:0002080	prenatal lethality	0.46
MP:0000886	abnormal cerebellar granule layer	0.46
ENSG00000009790	TRAF3IP3 PPI subnetwork	0.46
ENSG00000211799	ENSG00000211799 PPI subnetwork	0.46
ENSG00000211810	ENSG00000211810 PPI subnetwork	0.46
ENSG00000211739	ENSG00000211739 PPI subnetwork	0.46
ENSG00000211735	ENSG00000211735 PPI subnetwork	0.46
GO:0043407	negative regulation of MAP kinase activity	0.46
GO:0045815	positive regulation of gene expression, epigenetic	0.46
ENSG00000175104	TRAF6 PPI subnetwork	0.46
ENSG00000139505	MTMR6 PPI subnetwork	0.46
ENSG00000133313	CNDP2 PPI subnetwork	0.46
REACTOME_INTERFERON_GAMMA_SIGNALING	REACTOME_INTERFERON_GAMMA_SIGNALING	0.46
GO:0046887	positive regulation of hormone secretion	0.46
KEGG_STEROID_HORMONE_BIOSYNTHESIS	KEGG_STEROID_HORMONE_BIOSYNTHESIS	0.46
GO:0050707	regulation of cytokine secretion	0.46
ENSG00000148053	NTRK2 PPI subnetwork	0.46
GO:0031109	microtubule polymerization or depolymerization	0.46
MP:0002421	abnormal cell-mediated immunity	0.46
MP:0004448	abnormal presphenoid bone morphology	0.46
MP:0002641	anisopoikilocytosis	0.46

Original gene set ID	Original gene set description	Nominal P value
GO:0051092	positive regulation of NF-kappaB transcription factor activity	0.46
GO:0015697	quaternary ammonium group transport	0.46
REACTOME_TETRAHYDROBIOPTERIN_BH4_SYNTHESIS_RECYCLING_SALVAGE_A	REACTOME_TETRAHYDROBIOPTERIN_BH4_SYNTHESIS_RECYCLING_SALVAGE_AND_	0.46
MP:0000065	abnormal bone marrow cavity morphology	0.46
GO:0002286	T cell activation involved in immune response	0.46
MP:0002672	abnormal branchial arch artery morphology	0.46
MP:0003990	decreased neurotransmitter release	0.46
GO:0019637	organophosphate metabolic process	0.46
REACTOME_NEUROTRANSMITTER_RELEASE_CYCLE	REACTOME_NEUROTRANSMITTER_RELEASE_CYCLE	0.46
GO:0008047	enzyme activator activity	0.46
REACTOME_FGFR2C_LIGAND_BINDING_AND_ACTIVATION	REACTOME_FGFR2C_LIGAND_BINDING_AND_ACTIVATION	0.46
GO:0003206	cardiac chamber morphogenesis	0.46
MP:0002275	abnormal type II pneumocyte morphology	0.46
ENSG00000196419	XRCC6 PPI subnetwork	0.46
MP:0008217	abnormal B cell activation	0.46
GO:0006096	glycolysis	0.46
GO:0005916	fascia adherens	0.46
GO:0005248	voltage-gated sodium channel activity	0.46
KEGG_LONG_TERM_DEPRESSION	KEGG_LONG_TERM_DEPRESSION	0.46
MP:0004418	small parietal bone	0.46
ENSG00000187079	TEAD1 PPI subnetwork	0.46
ENSG00000165156	ZHX1 PPI subnetwork	0.46
ENSG00000131788	PIAS3 PPI subnetwork	0.46
ENSG00000155229	MMS19 PPI subnetwork	0.46
GO:0060411	cardiac septum morphogenesis	0.46
GO:0000819	sister chromatid segregation	0.46
GO:0060412	ventricular septum morphogenesis	0.46
GO:0050909	sensory perception of taste	0.46
GO:0043584	nose development	0.46
GO:0006026	aminoglycan catabolic process	0.46
ENSG00000145692	BHMT PPI subnetwork	0.46
GO:0048665	neuron fate specification	0.46
GO:0031519	PcG protein complex	0.46
ENSG00000125249	RAP2A PPI subnetwork	0.46
GO:0048610	cellular process involved in reproduction	0.46
ENSG00000039319	ZFYVE16 PPI subnetwork	0.46
ENSG00000124207	CSE1L PPI subnetwork	0.46
ENSG00000147010	SH3KBP1 PPI subnetwork	0.46
ENSG00000143867	OSR1 PPI subnetwork	0.46
ENSG00000141447	OSBPL1A PPI subnetwork	0.46
ENSG00000154310	TNIK PPI subnetwork	0.46
GO:0060444	branching involved in mammary gland duct morphogenesis	0.46
ENSG00000180855	ZNF443 PPI subnetwork	0.46
ENSG00000077549	CAPZB PPI subnetwork	0.46
GO:0015108	chloride transmembrane transporter activity	0.46
MP:0011088	partial neonatal lethality	0.46
MP:0002702	decreased circulating free fatty acid leve	0.46
ENSG00000174307	PHLDA3 PPI subnetwork	0.47
ENSG00000130758	MAP3K10 PPI subnetwork	0.47

Original gene set ID	Original gene set description	Nominal P value
ENSG00000110799	VWF PPI subnetwork	0.47
REACTOME_PURINE_METABOLISM	REACTOME_PURINE_METABOLISM	0.47
ENSG00000126458	RRAS PPI subnetwork	0.47
MP:0001529	abnormal vocalization	0.47
MP:0000599	enlarged liver	0.47
MP:0003560	osteoarthritis	0.47
ENSG00000145817	YIPF5 PPI subnetwork	0.47
GO:0001502	cartilage condensation	0.47
ENSG00000164708	PGAM2 PPI subnetwork	0.47
ENSG00000089154	GCN1L1 PPI subnetwork	0.47
GO:0042059	negative regulation of epidermal growth factor receptor signaling pathway	0.47
ENSG00000105447	GRWD1 PPI subnetwork	0.47
MP:0001364	decreased anxiety-related response	0.47
REACTOME_RNA_POLYMERASE_III_TRANSCRIPTION_INITIATION_FROM_TYPE_1	REACTOME_RNA_POLYMERASE_III_TRANSCRIPTION_INITIATION_FROM_TYPE_1_PI	0.47
ENSG00000183520	UTP11L PPI subnetwork	0.47
REACTOME_FGFR4_LIGAND_BINDING_AND_ACTIVATION	REACTOME_FGFR4_LIGAND_BINDING_AND_ACTIVATION	0.47
ENSG00000130561	SAG PPI subnetwork	0.47
ENSG00000173011	TADA2B PPI subnetwork	0.47
ENSG00000197892	KIF13B PPI subnetwork	0.47
ENSG00000198851	CD3E PPI subnetwork	0.47
ENSG00000135547	HEY2 PPI subnetwork	0.47
MP:0008212	absent mature B cells	0.47
GO:0015837	amine transport	0.47
GO:0006749	glutathione metabolic process	0.47
ENSG00000150455	TIRAP PPI subnetwork	0.47
ENSG00000183117	CSMD1 PPI subnetwork	0.47
ENSG00000069275	NUCKS1 PPI subnetwork	0.47
ENSG00000108515	ENO3 PPI subnetwork	0.47
GO:0060840	artery development	0.47
ENSG00000178028	DMAP1 PPI subnetwork	0.47
ENSG00000163602	RYBP PPI subnetwork	0.47
ENSG00000125991	ERGIC3 PPI subnetwork	0.47
ENSG00000144029	MRPS5 PPI subnetwork	0.47
REACTOME_FRS2:MEDIATED_ACTIVATION	REACTOME_FRS2:MEDIATED_ACTIVATION	0.47
MP:0000067	osteopetrosis	0.47
ENSG00000040199	PHLPP2 PPI subnetwork	0.47
ENSG00000135338	LCA5 PPI subnetwork	0.47
ENSG00000144566	RAB5A PPI subnetwork	0.47
KEGG_PATHOGENIC_ESCHERICHIA_COLI_INFECTION	KEGG_PATHOGENIC_ESCHERICHIA_COLI_INFECTION	0.47
ENSG00000072210	ALDH3A2 PPI subnetwork	0.47
GO:0032365	intracellular lipid transport	0.47
MP:0004024	aneuploidy	0.47
REACTOME_HORMONE:SENSITIVE_LIPASE_HSL:MEDIATED_TRIACYLGLYCEROL_	REACTOME_HORMONE:SENSITIVE_LIPASE_HSL:MEDIATED_TRIACYLGLYCEROL_HYD	0.47
KEGG_SPHINGOLIPID_METABOLISM	KEGG_SPHINGOLIPID_METABOLISM	0.47
ENSG00000214265	SNURF PPI subnetwork	0.47
GO:0032269	negative regulation of cellular protein metabolic process	0.47
ENSG00000007237	GAS7 PPI subnetwork	0.47
MP:0002643	poikilocytosis	0.47
GO:0001783	B cell apoptotic process	0.47

Original gene set ID	Original gene set description	Nominal P value
ENSG00000073111	MCM2 PPI subnetwork	0.47
ENSG00000181191	PJA1 PPI subnetwork	0.47
ENSG00000169251	NMD3 PPI subnetwork	0.47
GO:0030259	lipid glycosylation	0.47
ENSG00000117000	RLF PPI subnetwork	0.47
ENSG00000204227	RING1 PPI subnetwork	0.47
ENSG00000206287	RING1 PPI subnetwork	0.47
ENSG00000206215	ENSG00000206215 PPI subnetwork	0.47
GO:0048017	inositol lipid-mediated signaling	0.47
GO:0048015	phosphatidylinositol-mediated signaling	0.47
GO:2000104	negative regulation of DNA-dependent DNA replication	0.47
REACTOME_NUCLEOTIDE:LIKE_PURINERGIC_RECEPTORS	REACTOME_NUCLEOTIDE:LIKE_PURINERGIC_RECEPTORS	0.47
ENSG00000211790	ENSG00000211790 PPI subnetwork	0.47
ENSG00000189162	ENSG00000189162 PPI subnetwork	0.47
ENSG00000181163	NPM1 PPI subnetwork	0.47
ENSG00000137413	TAF8 PPI subnetwork	0.47
REACTOME_AMINE:DERIVED_HORMONES	REACTOME_AMINE:DERIVED_HORMONES	0.47
GO:0034470	ncRNA processing	0.47
ENSG00000214021	TTLL3 PPI subnetwork	0.47
ENSG00000092820	EZR PPI subnetwork	0.47
GO:0005244	voltage-gated ion channel activity	0.47
GO:0022832	voltage-gated channel activity	0.47
REACTOME_DNA_STRAND_ELONGATION	REACTOME_DNA_STRAND_ELONGATION	0.47
ENSG00000137709	POU2F3 PPI subnetwork	0.47
ENSG00000108179	PPIF PPI subnetwork	0.47
MP:0001053	abnormal neuromuscular synapse morphology	0.47
REACTOME_O:LINKED_GLYCOSYLATION_OF_MUCINS	REACTOME_O:LINKED_GLYCOSYLATION_OF_MUCINS	0.47
ENSG00000160654	CD3G PPI subnetwork	0.47
ENSG00000074266	EED PPI subnetwork	0.47
GO:0042976	activation of Janus kinase activity	0.47
GO:0002274	myeloid leukocyte activation	0.47
GO:0003205	cardiac chamber development	0.47
KEGG_PYRIMIDINE_METABOLISM	KEGG_PYRIMIDINE_METABOLISM	0.47
MP:0008657	increased interleukin-1 beta secretion	0.47
ENSG00000100664	EIF5 PPI subnetwork	0.47
ENSG00000015171	ZMYND11 PPI subnetwork	0.47
GO:0000151	ubiquitin ligase complex	0.47
GO:0005643	nuclear pore	0.47
MP:0001853	heart inflammation	0.47
GO:0010970	microtubule-based transport	0.47
ENSG00000148308	GTF3C5 PPI subnetwork	0.47
ENSG00000138081	FBXO11 PPI subnetwork	0.47
ENSG00000175324	LSM1 PPI subnetwork	0.47
ENSG00000100902	PSMA6 PPI subnetwork	0.47
GO:0050678	regulation of epithelial cell proliferation	0.47
MP:0008713	abnormal cytokine level	0.47
GO:0071774	response to fibroblast growth factor stimulus	0.47
GO:0044344	cellular response to fibroblast growth factor stimulus	0.47
GO:0022603	regulation of anatomical structure morphogenesis	0.47

Original gene set ID	Original gene set description	Nominal P value
ENSG00000177688	SUMO4 PPI subnetwork	0.47
GO:0030552	cAMP binding	0.47
KEGG_VASOPRESSIN_REGULATED_WATER_REABSORPTION	KEGG_VASOPRESSIN_REGULATED_WATER_REABSORPTION	0.47
ENSG00000064419	TNPO3 PPI subnetwork	0.47
GO:0000722	telomere maintenance via recombination	0.47
ENSG00000157193	LRP8 PPI subnetwork	0.47
GO:0060070	canonical Wnt receptor signaling pathway	0.47
MP:0002871	albuminuria	0.47
ENSG00000128595	CALU PPI subnetwork	0.47
GO:0006458	'de novo' protein folding	0.47
ENSG00000196154	S100A4 PPI subnetwork	0.47
ENSG00000198898	CAPZA2 PPI subnetwork	0.47
MP:0004025	polyploidy	0.47
ENSG00000043355	ZIC2 PPI subnetwork	0.48
ENSG00000182093	WRB PPI subnetwork	0.48
GO:0051225	spindle assembly	0.48
MP:0009454	impaired contextual conditioning behavior	0.48
ENSG00000169727	GPS1 PPI subnetwork	0.48
ENSG00000196730	DAPK1 PPI subnetwork	0.48
MP:0004310	small otic vesicle	0.48
MP:0006271	abnormal involution of the mammary gland	0.48
KEGG_DRUG_METABOLISM_CYTOCHROME_P450	KEGG_DRUG_METABOLISM_CYTOCHROME_P450	0.48
GO:0001764	neuron migration	0.48
GO:0061135	endopeptidase regulator activity	0.48
ENSG00000198561	CTNND1 PPI subnetwork	0.48
ENSG00000105135	ILVBL PPI subnetwork	0.48
ENSG00000173801	JUP PPI subnetwork	0.48
MP:0001243	abnormal dermal layer morphology	0.48
GO:0035239	tube morphogenesis	0.48
ENSG00000179041	RRS1 PPI subnetwork	0.48
MP:0008140	podocyte foot process effacement	0.48
MP:0000120	malocclusion	0.48
GO:2000602	regulation of interphase of mitotic cell cycle	0.48
MP:0001155	arrest of spermatogenesis	0.48
GO:0051493	regulation of cytoskeleton organization	0.48
ENSG00000102241	HTATSF1 PPI subnetwork	0.48
ENSG00000089250	NOS1 PPI subnetwork	0.48
ENSG00000065328	MCM10 PPI subnetwork	0.48
GO:0014020	primary neural tube formation	0.48
MP:0009936	abnormal dendritic spine morphology	0.48
GO:0009913	epidermal cell differentiation	0.48
ENSG00000070808	CAMK2A PPI subnetwork	0.48
GO:0044291	cell-cell contact zone	0.48
ENSG00000107623	GDF10 PPI subnetwork	0.48
MP:0005222	abnormal somite size	0.48
ENSG00000115211	EIF2B4 PPI subnetwork	0.48
GO:0090311	regulation of protein deacetylation	0.48
MP:0002459	abnormal B cell physiology	0.48
ENSG00000136950	ARPC5L PPI subnetwork	0.48

Original gene set ID	Original gene set description	Nominal P value
MP:0000681	abnormal thyroid gland morphology	0.48
GO:0015929	hexosaminidase activity	0.48
ENSG00000125352	RNF113A PPI subnetwork	0.48
ENSG00000187840	EIF4EBP1 PPI subnetwork	0.48
ENSG00000115966	ATF2 PPI subnetwork	0.48
ENSG00000160844	GATS PPI subnetwork	0.48
GO:0060759	regulation of response to cytokine stimulus	0.48
ENSG00000140332	TLE3 PPI subnetwork	0.48
GO:0000075	cell cycle checkpoint	0.48
GO:0051101	regulation of DNA binding	0.48
ENSG00000108561	C1QBP PPI subnetwork	0.48
ENSG00000108424	KPNB1 PPI subnetwork	0.48
ENSG00000138032	PPM1B PPI subnetwork	0.48
ENSG00000135097	MSI1 PPI subnetwork	0.48
GO:0044297	cell body	0.48
GO:0000289	nuclear-transcribed mRNA poly(A) tail shortening	0.48
ENSG00000115274	INO80B PPI subnetwork	0.48
ENSG00000173207	CKS1B PPI subnetwork	0.48
GO:0003279	cardiac septum development	0.48
ENSG00000018236	CNTN1 PPI subnetwork	0.48
MP:0002359	abnormal spleen germinal center morphology	0.48
ENSG00000164889	SLC4A2 PPI subnetwork	0.48
ENSG00000044574	HSPA5 PPI subnetwork	0.48
ENSG00000179348	GATA2 PPI subnetwork	0.48
MP:0002391	abnormal Peyer's patch germinal center morphology	0.48
MP:0001404	no spontaneous movement	0.48
ENSG00000170876	TMEM43 PPI subnetwork	0.48
GO:0016079	synaptic vesicle exocytosis	0.48
ENSG00000105880	DLX5 PPI subnetwork	0.48
GO:0045851	pH reduction	0.48
ENSG00000135333	EPHA7 PPI subnetwork	0.48
ENSG00000114166	KAT2B PPI subnetwork	0.48
GO:0002712	regulation of B cell mediated immunity	0.48
REACTOME_FGFR2_LIGAND_BINDING_AND_ACTIVATION	REACTOME_FGFR2_LIGAND_BINDING_AND_ACTIVATION	0.48
GO:0001910	regulation of leukocyte mediated cytotoxicity	0.48
ENSG00000157349	DDX19B PPI subnetwork	0.48
MP:0004057	thin myocardium compact layer	0.48
GO:0031214	biomineral tissue development	0.48
MP:0003675	kidney cysts	0.48
GO:0001578	microtubule bundle formation	0.48
ENSG00000140400	MAN2C1 PPI subnetwork	0.48
ENSG00000102893	PHKB PPI subnetwork	0.48
GO:0006687	glycosphingolipid metabolic process	0.48
ENSG00000008056	SYN1 PPI subnetwork	0.48
MP:0000755	hindlimb paralysis	0.48
MP:0002362	abnormal spleen marginal zone morphology	0.48
GO:0032623	interleukin-2 production	0.48
ENSG00000143373	ZNF687 PPI subnetwork	0.48
MP:0001929	abnormal gametogenesis	0.48

Original gene set ID	Original gene set description	Nominal P value
MP:0002826	tonic seizures	0.48
ENSG00000047410	TPR PPI subnetwork	0.48
ENSG00000185130	HIST1H2BL PPI subnetwork	0.48
REACTOME_G0_AND_EARLY_G1	REACTOME_G0_AND_EARLY_G1	0.48
GO:0006644	phospholipid metabolic process	0.48
ENSG00000130589	RP4-697K14.7 PPI subnetwork	0.48
MP:0000274	enlarged heart	0.48
ENSG00000141582	CBX4 PPI subnetwork	0.48
ENSG00000050405	LIMA1 PPI subnetwork	0.48
ENSG00000108852	MPP2 PPI subnetwork	0.48
GO:0000287	magnesium ion binding	0.48
GO:0007283	spermatogenesis	0.48
GO:0048232	male gamete generation	0.48
MP:0004251	failure of heart looping	0.48
ENSG00000108100	CCNY PPI subnetwork	0.48
MP:0001730	embryonic growth arrest	0.48
MP:0000939	decreased motor neuron number	0.48
MP:0005312	pericardial effusion	0.48
ENSG00000007174	DNAH9 PPI subnetwork	0.48
ENSG00000180370	PAK2 PPI subnetwork	0.48
MP:0009866	abnormal aorta wall morphology	0.48
ENSG00000215021	PHB2 PPI subnetwork	0.48
ENSG00000117118	SDHB PPI subnetwork	0.48
GO:0007178	transmembrane receptor protein serine/threonine kinase signaling pathway	0.48
ENSG00000134046	MBD2 PPI subnetwork	0.48
MP:0005405	axon degeneration	0.48
MP:0000186	decreased circulating HDL cholesterol leve	0.48
MP:0000074	abnormal neurocranium morphology	0.48
GO:0032371	regulation of sterol transport	0.48
GO:0032374	regulation of cholesterol transport	0.48
ENSG00000111602	TIMELESS PPI subnetwork	0.49
REACTOME_COSTIMULATION_BY_THE_CD28_FAMILY	REACTOME_COSTIMULATION_BY_THE_CD28_FAMILY	0.49
REACTOME_DOWNSTREAM_SIGNAL_TRANSDUCTION	REACTOME_DOWNSTREAM_SIGNAL_TRANSDUCTION	0.49
GO:0032201	telomere maintenance via semi-conservative replication	0.49
GO:0007405	neuroblast proliferation	0.49
GO:0060026	convergent extension	0.49
ENSG00000113658	SMAD5 PPI subnetwork	0.49
ENSG00000160695	ENSG00000160695 PPI subnetwork	0.49
GO:0071705	nitrogen compound transport	0.49
ENSG00000130811	EIF3G PPI subnetwork	0.49
GO:0071843	cellular component biogenesis at cellular leve	0.49
GO:0030165	PDZ domain binding	0.49
ENSG00000009307	CSDE1 PPI subnetwork	0.49
ENSG00000132963	POMP PPI subnetwork	0.49
GO:0001974	blood vessel remodeling	0.49
ENSG00000167286	CD3D PPI subnetwork	0.49
MP:0000852	small cerebellum	0.49
GO:0016052	carbohydrate catabolic process	0.49
ENSG00000014216	CAPN1 PPI subnetwork	0.49

Original gene set ID	Original gene set description	Nominal P value
KEGG_GLYCEROLIPID_METABOLISM	KEGG_GLYCEROLIPID_METABOLISM	0.49
MP:0005010	abnormal CD8-positive T cell morphology	0.49
ENSG00000162236	STX5 PPI subnetwork	0.49
ENSG00000157500	APPL1 PPI subnetwork	0.49
MP:0002270	abnormal pulmonary alveolus morphology	0.49
GO:0048511	rhythmic process	0.49
GO:0050820	positive regulation of coagulation	0.49
GO:0016247	channel regulator activity	0.49
GO:0016853	isomerase activity	0.49
ENSG00000075624	ACTB PPI subnetwork	0.49
GO:0003014	renal system process	0.49
MP:0001882	abnormal lactation	0.49
GO:0043370	regulation of CD4-positive, alpha-beta T cell differentiation	0.49
GO:0061311	cell surface receptor signaling pathway involved in heart development	0.49
GO:0031418	L-ascorbic acid binding	0.49
ENSG00000171735	CAMTA1 PPI subnetwork	0.49
MP:0001721	absent visceral yolk sac blood islands	0.49
ENSG00000157152	ENSG00000157152 PPI subnetwork	0.49
ENSG00000177728	KIAA0195 PPI subnetwork	0.49
MP:0002856	abnormal vestibular ganglion morphology	0.49
GO:0033006	regulation of mast cell activation involved in immune response	0.49
GO:0046637	regulation of alpha-beta T cell differentiation	0.49
GO:0072594	establishment of protein localization to organelle	0.49
GO:0001654	eye development	0.49
GO:0002711	positive regulation of T cell mediated immunity	0.49
ENSG00000168497	SDPR PPI subnetwork	0.49
GO:0015248	sterol transporter activity	0.49
KEGG_ASCORBATE_AND_ALDARATE_METABOLISM	KEGG_ASCORBATE_AND_ALDARATE_METABOLISM	0.49
MP:0008809	increased spleen iron level	0.49
ENSG00000085733	CTTN PPI subnetwork	0.49
ENSG00000164346	NSA2 PPI subnetwork	0.49
MP:0000745	tremors	0.49
ENSG00000134769	DTNA PPI subnetwork	0.49
MP:0002912	abnormal excitatory postsynaptic potential	0.49
ENSG00000198356	ASNA1 PPI subnetwork	0.49
ENSG00000104976	SNAPC2 PPI subnetwork	0.49
GO:0022037	metencephalon development	0.49
ENSG00000196262	PPIA PPI subnetwork	0.49
ENSG00000198618	ENSG00000198618 PPI subnetwork	0.49
GO:0032318	regulation of Ras GTPase activity	0.49
GO:0040012	regulation of locomotion	0.49
GO:0043542	endothelial cell migration	0.49
ENSG00000147168	IL2RG PPI subnetwork	0.49
MP:0010701	fusion of atlas and odontoid process	0.49
MP:0000336	decreased mast cell number	0.49
GO:0004003	ATP-dependent DNA helicase activity	0.49
GO:0009593	detection of chemical stimulus	0.49
REACTOME_REGULATION_OF_IFNG_SIGNALING	REACTOME_REGULATION_OF_IFNG_SIGNALING	0.49
MP:0001935	decreased litter size	0.49

Original gene set ID	Original gene set description	Nominal P value
ENSG00000125818	PSMF1 PPI subnetwork	0.49
GO:0045123	cellular extravasation	0.49
ENSG00000078967	UBE2D4 PPI subnetwork	0.49
ENSG00000129351	ILF3 PPI subnetwork	0.49
ENSG00000149131	SERPING1 PPI subnetwork	0.49
ENSG00000104408	EIF3E PPI subnetwork	0.49
ENSG00000152795	HNRPDL PPI subnetwork	0.49
GO:0033627	cell adhesion mediated by integrin	0.49
ENSG00000211889	ENSG00000211889 PPI subnetwork	0.49
ENSG00000137975	CLCA2 PPI subnetwork	0.49
ENSG00000157514	TSC22D3 PPI subnetwork	0.49
ENSG00000100284	TOM1 PPI subnetwork	0.49
ENSG00000124145	SDC4 PPI subnetwork	0.49
ENSG00000147082	CCNB3 PPI subnetwork	0.49
ENSG00000078699	CBFA2T2 PPI subnetwork	0.49
ENSG00000184216	IRAK1 PPI subnetwork	0.49
ENSG00000115942	ORC2 PPI subnetwork	0.49
ENSG00000168924	LETM1 PPI subnetwork	0.49
ENSG00000079246	XRCC5 PPI subnetwork	0.49
REACTOME_SIGNALING_BY_FGFR	REACTOME_SIGNALING_BY_FGFR	0.49
KEGG_PRION_DISEASES	KEGG_PRION_DISEASES	0.49
REACTOME_PYRUVATE_METABOLISM_AND_CITRIC_ACID_TCA_CYCLE	REACTOME_PYRUVATE_METABOLISM_AND_CITRIC_ACID_TCA_CYCLE	0.49
ENSG00000137309	HMGA1 PPI subnetwork	0.49
ENSG00000072803	FBXW11 PPI subnetwork	0.49
MP:0000920	abnormal myelination	0.49
MP:0009764	decreased sensitivity to induced morbidity/mortality	0.49
ENSG00000105989	WNT2 PPI subnetwork	0.49
ENSG00000075290	WNT8B PPI subnetwork	0.49
ENSG00000143816	WNT9A PPI subnetwork	0.49
ENSG00000169884	WNT10B PPI subnetwork	0.49
ENSG00000135925	WNT10A PPI subnetwork	0.49
ENSG00000158955	WNT9B PPI subnetwork	0.49
ENSG00000085741	WNT11 PPI subnetwork	0.49
ENSG00000061492	WNT8A PPI subnetwork	0.49
ENSG00000146109	ABT1 PPI subnetwork	0.49
ENSG00000175063	UBE2C PPI subnetwork	0.49
MP:0002774	small prostate gland	0.49
ENSG00000116560	SFPQ PPI subnetwork	0.49
GO:0022803	passive transmembrane transporter activity	0.49
GO:0015267	channel activity	0.49
GO:0061138	morphogenesis of a branching epithelium	0.49
ENSG00000100201	DDX17 PPI subnetwork	0.49
ENSG00000104814	MAP4K1 PPI subnetwork	0.49
ENSG00000166225	FRS2 PPI subnetwork	0.49
ENSG00000145901	TNIP1 PPI subnetwork	0.49
ENSG00000185245	GP1BA PPI subnetwork	0.5
MP:0001045	abnormal enteric ganglia morphology	0.5
ENSG00000158796	DEDD PPI subnetwork	0.5
MP:0000435	shortened head	0.5

Original gene set ID	Original gene set description	Nominal P value
GO:0045005	maintenance of fidelity involved in DNA-dependent DNA replicatior	0.5
REACTOME_EXTENSION_OF_TELOMERES	REACTOME_EXTENSION_OF_TELOMERES	0.5
GO:0060603	mammary gland duct morphogenesis	0.5
REACTOME_PURINE_SALVAGE	REACTOME_PURINE_SALVAGE	0.5
ENSG00000161570	CCL5 PPI subnetwork	0.5
ENSG00000203283	ENSG00000203283 PPI subnetwork	0.5
GO:0042108	positive regulation of cytokine biosynthetic process	0.5
GO:0002285	lymphocyte activation involved in immune response	0.5
GO:0000725	recombinational repair	0.5
ENSG00000061337	LZTS1 PPI subnetwork	0.5
GO:0006582	melanin metabolic process	0.5
ENSG00000115596	WNT6 PPI subnetwork	0.5
ENSG00000101132	PFDN4 PPI subnetwork	0.5
ENSG00000142507	PSMB6 PPI subnetwork	0.5
GO:0051640	organelle localization	0.5
ENSG00000074047	GLI2 PPI subnetwork	0.5
GO:0014823	response to activity	0.5
MP:0003049	abnormal lumbar vertebrae morphology	0.5
MP:0008496	decreased IgG2a level	0.5
ENSG00000101557	USP14 PPI subnetwork	0.5
KEGG_GNRH_SIGNALING_PATHWAY	KEGG_GNRH_SIGNALING_PATHWAY	0.5
MP:0002059	abnormal seminal vesicle morphology	0.5
ENSG00000108231	LGI1 PPI subnetwork	0.5
ENSG00000149428	HYOU1 PPI subnetwork	0.5
ENSG00000139352	ASCL1 PPI subnetwork	0.5
MP:0010724	thick interventricular septum	0.5
GO:0050680	negative regulation of epithelial cell proliferatior	0.5
KEGG_THYROID_CANCER	KEGG_THYROID_CANCER	0.5
GO:0008635	activation of cysteine-type endopeptidase activity involved in apoptotic process by c	0.5
ENSG00000184203	PPP1R2 PPI subnetwork	0.5
ENSG00000187558	ENSG00000187558 PPI subnetwork	0.5
ENSG00000107341	UBE2R2 PPI subnetwork	0.5
GO:0030666	endocytic vesicle membrane	0.5
MP:0001324	abnormal eye pigmentation	0.5
MP:0002761	abnormal hippocampal mossy fiber morphology	0.5
ENSG00000088926	F11 PPI subnetwork	0.5
ENSG00000134453	RBM17 PPI subnetwork	0.5
GO:0042398	cellular modified amino acid biosynthetic process	0.5
GO:0016887	ATPase activity	0.5
GO:0002040	sprouting angiogenesis	0.5
GO:0051329	interphase of mitotic cell cycle	0.5
GO:0030315	T-tubule	0.5
ENSG00000155097	ATP6V1C1 PPI subnetwork	0.5
MP:0002640	reticulocytosis	0.5
GO:0031256	leading edge membrane	0.5
ENSG00000141480	ARRB2 PPI subnetwork	0.5
GO:0071156	regulation of cell cycle arrest	0.5
GO:0000724	double-strand break repair via homologous recombinatior	0.5
ENSG00000125753	VASP PPI subnetwork	0.5

Original gene set ID	Original gene set description	Nominal P value
KEGG_PANTOTHENATE_AND_COA_BIOSYNTHESIS	KEGG_PANTOTHENATE_AND_COA_BIOSYNTHESIS	0.5
ENSG00000106089	STX1A PPI subnetwork	0.5
GO:0051248	negative regulation of protein metabolic process	0.5
GO:0045639	positive regulation of myeloid cell differentiation	0.5
GO:0048029	monosaccharide binding	0.5
MP:0000438	abnormal cranium morphology	0.5
GO:0043235	receptor complex	0.5
REACTOME_RNA_POLYMERASE_III_TRANSCRIPTION_INITIATION	REACTOME_RNA_POLYMERASE_III_TRANSCRIPTION_INITIATION	0.5
REACTOME_IRAK2_MEDIATED_ACTIVATION_OF_TAK1_COMPLEX_UPON_TLR78	REACTOME_IRAK2_MEDIATED_ACTIVATION_OF_TAK1_COMPLEX_UPON_TLR78_OF	0.5
GO:0007131	reciprocal meiotic recombination	0.5
GO:0035825	reciprocal DNA recombination	0.5
GO:0006301	postreplication repair	0.5
GO:0046530	photoreceptor cell differentiation	0.5
ENSG00000139182	CLSTN3 PPI subnetwork	0.5
MP:0002027	lung adenocarcinoma	0.5
GO:0008081	phosphoric diester hydrolase activity	0.5
ENSG00000112651	MRPL2 PPI subnetwork	0.5
ENSG00000198216	CACNA1E PPI subnetwork	0.5
KEGG_PHOSPHATIDYLINOSITOL_SIGNALING_SYSTEM	KEGG_PHOSPHATIDYLINOSITOL_SIGNALING_SYSTEM	0.5
GO:0051004	regulation of lipoprotein lipase activity	0.5
MP:0004763	absent brainstem auditory evoked potential	0.5
GO:0007062	sister chromatid cohesion	0.5
ENSG00000011465	DCN PPI subnetwork	0.5
ENSG00000182359	KBTBD3 PPI subnetwork	0.5
ENSG00000082516	GEMIN5 PPI subnetwork	0.5
GO:0008286	insulin receptor signaling pathway	0.5
GO:0032635	interleukin-6 production	0.5
GO:0032675	regulation of interleukin-6 production	0.5
ENSG00000119414	PPP6C PPI subnetwork	0.5
ENSG00000104064	GABPB1 PPI subnetwork	0.5
ENSG00000131828	PDHA1 PPI subnetwork	0.5
ENSG00000161920	MED11 PPI subnetwork	0.5
ENSG00000163811	WDR43 PPI subnetwork	0.5
MP:0004522	abnormal orientation of cochlear hair cell stereociliary bundles	0.5
REACTOME_CD28_CO:STIMULATION	REACTOME_CD28_CO:STIMULATION	0.5
ENSG00000137710	RDX PPI subnetwork	0.5
ENSG00000130041	ENSG00000130041 PPI subnetwork	0.5
ENSG00000177954	RPS27 PPI subnetwork	0.5
ENSG00000183814	LIN9 PPI subnetwork	0.5
REACTOME_SIGNALING_BY_WNT	REACTOME_SIGNALING_BY_WNT	0.5
REACTOME_DEGRADATION_OF_BETA:CATENIN_BY_THE_DESTRUCTION_COMP	REACTOME_DEGRADATION_OF_BETA:CATENIN_BY_THE_DESTRUCTION_COMPLEX	0.5
ENSG00000204523	ENSG00000204523 PPI subnetwork	0.5
ENSG00000174405	LIG4 PPI subnetwork	0.5
GO:0051271	negative regulation of cellular component movement	0.5
GO:0032024	positive regulation of insulin secretion	0.5
MP:0005562	decreased mean corpuscular hemoglobin	0.5
GO:0033549	MAP kinase phosphatase activity	0.5
ENSG00000140451	PIF1 PPI subnetwork	0.5
GO:0003018	vascular process in circulatory system	0.5

Original gene set ID	Original gene set description	Nominal P value
GO:0031670	cellular response to nutrient	0.5
MP:0004726	abnormal nasal capsule morphology	0.5
ENSG00000055208	TAB2 PPI subnetwork	0.5
GO:0002573	myeloid leukocyte differentiation	0.5
ENSG00000196497	IPO4 PPI subnetwork	0.5
GO:0015081	sodium ion transmembrane transporter activity	0.5
ENSG00000130803	ZNF317 PPI subnetwork	0.5
MP:0005145	increased circulating VLDL cholesterol leve	0.5
ENSG00000148943	LIN7C PPI subnetwork	0.5
MP:0005630	increased lung weight	0.5
GO:0006754	ATP biosynthetic process	0.5
ENSG00000082014	SMARCD3 PPI subnetwork	0.5
MP:0002397	abnormal bone marrow morphology	0.5
ENSG00000090054	SPTLC1 PPI subnetwork	0.5
ENSG00000158186	MRAS PPI subnetwork	0.5
MP:0005659	decreased susceptibility to diet-induced obesity	0.5
GO:0030897	HOPS complex	0.5
GO:0042074	cell migration involved in gastrulation	0.5
GO:0010639	negative regulation of organelle organization	0.5
GO:0050880	regulation of blood vessel size	0.5
ENSG00000153107	ANAPC1 PPI subnetwork	0.5
ENSG00000166147	FBN1 PPI subnetwork	0.5
ENSG00000174233	ADCY6 PPI subnetwork	0.5
REACTOME_RAS_ACTIVATION_UOPN_CA2_INFUX_THROUGH_NMDA_RECEPTO	REACTOME_RAS_ACTIVATION_UOPN_CA2_INFUX_THROUGH_NMDA_RECEPTOR	0.5
GO:0035036	sperm-egg recognition	0.5
ENSG00000117500	TMED5 PPI subnetwork	0.5
ENSG00000127914	AKAP9 PPI subnetwork	0.51
GO:0050798	activated T cell proliferation	0.51
ENSG00000117360	PRPF3 PPI subnetwork	0.51
ENSG00000173372	C1QA PPI subnetwork	0.51
ENSG00000204301	NOTCH4 PPI subnetwork	0.51
REACTOME_MAP_KINASE_ACTIVATION_IN_TLR_CASCADE	REACTOME_MAP_KINASE_ACTIVATION_IN_TLR_CASCADE	0.51
GO:0043186	P granule	0.51
GO:0045495	pole plasm	0.51
GO:0060293	germ plasm	0.51
GO:0048016	inositol phosphate-mediated signaling	0.51
MP:0002024	T cell derived lymphoma	0.51
GO:0055037	recycling endosome	0.51
MP:0005172	reduced eye pigmentation	0.51
GO:0004866	endopeptidase inhibitor activity	0.51
MP:0003194	abnormal frequency of paradoxical sleep	0.51
MP:0005543	corneal thinning	0.51
ENSG00000071127	WDR1 PPI subnetwork	0.51
ENSG00000117322	CR2 PPI subnetwork	0.51
ENSG00000065675	PRKCQ PPI subnetwork	0.51
MP:0002747	abnormal aortic valve morphology	0.51
GO:0001948	glycoprotein binding	0.51
GO:0043473	pigmentation	0.51
GO:0050867	positive regulation of cell activation	0.51

Original gene set ID	Original gene set description	Nominal P value
ENSG00000165029	ABCA1 PPI subnetwork	0.51
GO:0009163	nucleoside biosynthetic process	0.51
MP:0005560	decreased circulating glucose level	0.51
REACTOME_SMOOTH_MUSCLE_CONTRACTION	REACTOME_SMOOTH_MUSCLE_CONTRACTION	0.51
GO:0006688	glycosphingolipid biosynthetic process	0.51
ENSG00000128829	EIF2AK4 PPI subnetwork	0.51
ENSG00000006715	VPS41 PPI subnetwork	0.51
ENSG00000105671	DDX49 PPI subnetwork	0.51
ENSG00000170632	ARMC10 PPI subnetwork	0.51
GO:0000976	transcription regulatory region sequence-specific DNA binding	0.51
GO:0019217	regulation of fatty acid metabolic process	0.51
GO:0043568	positive regulation of insulin-like growth factor receptor signaling pathway	0.51
MP:0001541	abnormal osteoclast physiology	0.51
ENSG00000127334	DYRK2 PPI subnetwork	0.51
ENSG00000183495	EP400 PPI subnetwork	0.51
ENSG00000174775	HRAS PPI subnetwork	0.51
ENSG00000133265	HSPBP1 PPI subnetwork	0.51
MP:0001081	abnormal cranial ganglia morphology	0.51
ENSG00000143933	CALM2 PPI subnetwork	0.51
ENSG00000160014	CALM3 PPI subnetwork	0.51
ENSG00000198668	CALM1 PPI subnetwork	0.51
ENSG00000013503	POLR3B PPI subnetwork	0.51
ENSG00000123066	MED13L PPI subnetwork	0.51
GO:0042737	drug catabolic process	0.51
GO:0030126	COPI vesicle coat	0.51
ENSG00000145391	SETD7 PPI subnetwork	0.51
ENSG00000061987	MON2 PPI subnetwork	0.51
GO:0045428	regulation of nitric oxide biosynthetic process	0.51
ENSG00000203814	HIST2H2BF PPI subnetwork	0.51
MP:0005094	abnormal T cell proliferation	0.51
GO:0009260	ribonucleotide biosynthetic process	0.51
GO:0070035	purine NTP-dependent helicase activity	0.51
GO:0008026	ATP-dependent helicase activity	0.51
GO:0043174	nucleoside salvage	0.51
ENSG00000120129	DUSP1 PPI subnetwork	0.51
GO:0051053	negative regulation of DNA metabolic process	0.51
ENSG00000113140	SPARC PPI subnetwork	0.51
ENSG00000135218	CD36 PPI subnetwork	0.51
ENSG00000185057	ENSG00000185057 PPI subnetwork	0.51
GO:0032715	negative regulation of interleukin-6 production	0.51
ENSG00000134686	PHC2 PPI subnetwork	0.51
GO:0015698	inorganic anion transport	0.51
ENSG00000064313	TAF2 PPI subnetwork	0.51
ENSG00000215328	HSPA1A PPI subnetwork	0.51
ENSG00000113494	PRLR PPI subnetwork	0.51
GO:0046504	glycerol ether biosynthetic process	0.51
GO:0008206	bile acid metabolic process	0.51
ENSG00000070018	LRP6 PPI subnetwork	0.51
ENSG00000139496	NUPL1 PPI subnetwork	0.51

Original gene set ID	Original gene set description	Nominal P value
GO:0006631	fatty acid metabolic process	0.51
GO:0048546	digestive tract morphogenesis	0.51
GO:0045444	fat cell differentiation	0.51
REACTOME_GLUTATHIONE_SYNTHESIS_AND_RECYCLING	REACTOME_GLUTATHIONE_SYNTHESIS_AND_RECYCLING	0.51
ENSG00000169375	SIN3A PPI subnetwork	0.51
ENSG00000147854	UHRF2 PPI subnetwork	0.51
ENSG00000173876	TUBB8 PPI subnetwork	0.51
MP:0000049	abnormal middle ear morphology	0.51
GO:0051222	positive regulation of protein transport	0.51
ENSG00000119917	IFIT3 PPI subnetwork	0.51
GO:0060113	inner ear receptor cell differentiation	0.51
GO:0060219	camera-type eye photoreceptor cell differentiation	0.51
GO:0035051	cardiac cell differentiation	0.51
ENSG00000115750	TAF1B PPI subnetwork	0.51
MP:0001516	abnormal motor coordination/ balance	0.51
GO:0019835	cytolysis	0.51
MP:0002273	abnormal pulmonary alveolus epithelial cell morphology	0.51
GO:0050864	regulation of B cell activation	0.51
MP:0003132	increased pre-B cell number	0.51
GO:0060828	regulation of canonical Wnt receptor signaling pathway	0.51
REACTOME_SEROTONIN_NEUROTRANSMITTER_RELEASE_CYCLE	REACTOME_SEROTONIN_NEUROTRANSMITTER_RELEASE_CYCLE	0.51
REACTOME_DOPAMINE_NEUROTRANSMITTER_RELEASE_CYCLE	REACTOME_DOPAMINE_NEUROTRANSMITTER_RELEASE_CYCLE	0.51
GO:0008629	induction of apoptosis by intracellular signals	0.51
ENSG00000167513	CDT1 PPI subnetwork	0.51
GO:0051251	positive regulation of lymphocyte activation	0.51
MP:0001092	abnormal trigeminal ganglion morphology	0.51
GO:0051147	regulation of muscle cell differentiation	0.51
REACTOME_RNA_POLYMERASE_III_TRANSCRIPTION_INITIATION_FROM_TYPE_2	REACTOME_RNA_POLYMERASE_III_TRANSCRIPTION_INITIATION_FROM_TYPE_2_PI	0.51
ENSG00000131459	GFPT2 PPI subnetwork	0.51
ENSG00000008294	SPAG9 PPI subnetwork	0.51
GO:0040014	regulation of multicellular organism growth	0.51
KEGG_GLYCOSPHINGOLIPID_BIOSYNTHESIS_GANGLIO_SERIES	KEGG_GLYCOSPHINGOLIPID_BIOSYNTHESIS_GANGLIO_SERIES	0.51
GO:0030194	positive regulation of blood coagulation	0.51
MP:0006298	abnormal platelet activation	0.51
ENSG00000156313	RPGR PPI subnetwork	0.51
ENSG00000078399	HOXA9 PPI subnetwork	0.51
GO:0030837	negative regulation of actin filament polymerization	0.51
REACTOME_INTERACTIONS_OF_VPR_WITH_HOST_CELLULAR_PROTEINS	REACTOME_INTERACTIONS_OF_VPR_WITH_HOST_CELLULAR_PROTEINS	0.51
GO:0060021	palate development	0.51
GO:0008180	signalosome	0.51
REACTOME_CREB_PHOSPHORYLATION_THROUGH_THE_ACTIVATION_OF_RAS	REACTOME_CREB_PHOSPHORYLATION_THROUGH_THE_ACTIVATION_OF_RAS	0.51
ENSG00000153914	SREK1 PPI subnetwork	0.51
MP:0004098	abnormal cerebellar granule cell morphology	0.51
GO:0014031	mesenchymal cell development	0.52
GO:2000514	regulation of CD4-positive, alpha-beta T cell activation	0.52
ENSG00000139372	TDG PPI subnetwork	0.52
MP:0005362	abnormal Langerhans cell physiology	0.52
REACTOME_RNA_POLYMERASE_III_CHAIN_ELONGATION	REACTOME_RNA_POLYMERASE_III_CHAIN_ELONGATION	0.52
GO:0070201	regulation of establishment of protein localization	0.52

Original gene set ID	Original gene set description	Nominal P value
GO:0005657	replication fork	0.52
ENSG00000081479	LRP2 PPI subnetwork	0.52
ENSG00000071243	ING3 PPI subnetwork	0.52
GO:0007043	cell-cell junction assembly	0.52
MP:0003938	abnormal ear development	0.52
ENSG00000100722	ZC3H14 PPI subnetwork	0.52
ENSG00000135679	MDM2 PPI subnetwork	0.52
ENSG00000116106	EPHA4 PPI subnetwork	0.52
REACTOME_HIGHLY_CALCIUM_PERMEABLE_POSTSYNAPTIC_NICOTINIC_ACETY	REACTOME_HIGHLY_CALCIUM_PERMEABLE_POSTSYNAPTIC_NICOTINIC_ACETYLCH	0.52
GO:0006937	regulation of muscle contraction	0.52
ENSG00000165732	DDX21 PPI subnetwork	0.52
GO:0019438	aromatic compound biosynthetic process	0.52
ENSG00000175073	VCPIP1 PPI subnetwork	0.52
GO:0009113	purine base biosynthetic process	0.52
GO:0072655	establishment of protein localization in mitochondrion	0.52
GO:0044269	glycerol ether catabolic process	0.52
GO:0046464	acylglycerol catabolic process	0.52
GO:0046461	neutral lipid catabolic process	0.52
MP:0003627	abnormal leukocyte tethering or rolling	0.52
KEGG_TGF_BETA_SIGNALING_PATHWAY	KEGG_TGF_BETA_SIGNALING_PATHWAY	0.52
GO:0046148	pigment biosynthetic process	0.52
ENSG00000215754	ENSG00000215754 PPI subnetwork	0.52
ENSG00000156802	ATAD2 PPI subnetwork	0.52
REACTOME_SYNTHESIS_OF_GLYCOSYLPHOSPHATIDYLINOSITOL_GPI	REACTOME_SYNTHESIS_OF_GLYCOSYLPHOSPHATIDYLINOSITOL_GPI	0.52
ENSG00000185024	BRF1 PPI subnetwork	0.52
ENSG00000149970	CNKSR2 PPI subnetwork	0.52
GO:0016579	protein deubiquitination	0.52
GO:0043021	ribonucleoprotein complex binding	0.52
MP:0006082	CNS inflammation	0.52
GO:0006362	transcription elongation from RNA polymerase I promoter	0.52
ENSG00000197238	HIST1H4J PPI subnetwork	0.52
ENSG00000183941	HIST2H4A PPI subnetwork	0.52
ENSG00000197914	HIST1H4K PPI subnetwork	0.52
ENSG00000198518	HIST1H4E PPI subnetwork	0.52
ENSG00000197837	HIST4H4 PPI subnetwork	0.52
ENSG00000182217	HIST2H4B PPI subnetwork	0.52
ENSG00000158406	HIST1H4H PPI subnetwork	0.52
ENSG00000198558	HIST1H4L PPI subnetwork	0.52
ENSG00000124529	HIST1H4B PPI subnetwork	0.52
ENSG00000197061	HIST1H4C PPI subnetwork	0.52
ENSG00000188987	HIST1H4D PPI subnetwork	0.52
ENSG00000198339	HIST1H4I PPI subnetwork	0.52
ENSG00000198327	HIST1H4F PPI subnetwork	0.52
ENSG00000196176	HIST1H4A PPI subnetwork	0.52
ENSG00000104738	MCM4 PPI subnetwork	0.52
GO:0005930	axoneme	0.52
ENSG00000168374	ARF4 PPI subnetwork	0.52
GO:0002696	positive regulation of leukocyte activation	0.52
MP:0006138	congestive heart failure	0.52

Original gene set ID	Original gene set description	Nominal P value
ENSG00000135269	TES PPI subnetwork	0.52
MP:0000102	abnormal nasal bone morphology	0.52
ENSG00000108679	LGALS3BP PPI subnetwork	0.52
ENSG00000111229	ARPC3 PPI subnetwork	0.52
GO:0031105	septin complex	0.52
GO:0032156	septin cytoskeleton	0.52
GO:0015297	antiporter activity	0.52
GO:0045665	negative regulation of neuron differentiation	0.52
ENSG00000166266	CUL5 PPI subnetwork	0.52
GO:0050860	negative regulation of T cell receptor signaling pathway	0.52
GO:0050858	negative regulation of antigen receptor-mediated signaling pathway	0.52
GO:0072378	blood coagulation, fibrin clot formation	0.52
GO:0070848	response to growth factor stimulus	0.52
ENSG00000145191	EIF2B5 PPI subnetwork	0.52
GO:0061134	peptidase regulator activity	0.52
GO:0020027	hemoglobin metabolic process	0.52
ENSG00000145782	ATG12 PPI subnetwork	0.52
MP:0000876	Purkinje cell degeneration	0.52
GO:0042612	MHC class I protein complex	0.52
ENSG00000171421	MRPL36 PPI subnetwork	0.52
ENSG00000104856	RELB PPI subnetwork	0.52
GO:0060341	regulation of cellular localization	0.52
MP:0009660	abnormal induced retinal neovascularization	0.52
GO:0030140	trans-Golgi network transport vesicle	0.52
ENSG00000135972	MRPS9 PPI subnetwork	0.52
ENSG00000100852	ARHGAP5 PPI subnetwork	0.52
GO:0005680	anaphase-promoting complex	0.52
ENSG00000171552	BCL2L1 PPI subnetwork	0.52
REACTOME_TELOMERE_C:STRAND_LAGGING_STRAND_SYNTHESIS	REACTOME_TELOMERE_C:STRAND_LAGGING_STRAND_SYNTHESIS	0.52
MP:0005671	abnormal response to transplant	0.52
GO:0060284	regulation of cell development	0.52
GO:0005243	gap junction channel activity	0.52
MP:0005598	decreased ventricle muscle contractility	0.52
GO:0007519	skeletal muscle tissue development	0.52
GO:0030155	regulation of cell adhesion	0.52
GO:0034707	chloride channel complex	0.52
GO:0016459	myosin complex	0.52
GO:0006144	purine base metabolic process	0.52
MP:0000208	decreased hematocrit	0.52
GO:0048729	tissue morphogenesis	0.52
ENSG00000100994	PYGB PPI subnetwork	0.52
ENSG00000070770	CSNK2A2 PPI subnetwork	0.52
GO:0007597	blood coagulation, intrinsic pathway	0.52
REACTOME_RECRUITMENT_OF_NUMA_TO_MITOTIC_CENTROSOMES	REACTOME_RECRUITMENT_OF_NUMA_TO_MITOTIC_CENTROSOMES	0.52
KEGG_ANTIGEN_PROCESSING_AND_PRESENTATION	KEGG_ANTIGEN_PROCESSING_AND_PRESENTATION	0.52
ENSG00000206234	ENSG00000206234 PPI subnetwork	0.52
ENSG00000204264	PSMB8 PPI subnetwork	0.52
ENSG00000206298	PSMB8 PPI subnetwork	0.52
GO:0009247	glycolipid biosynthetic process	0.52

Original gene set ID	Original gene set description	Nominal P value
GO:0019932	second-messenger-mediated signaling	0.52
GO:0000188	inactivation of MAPK activity	0.52
ENSG00000163512	AZI2 PPI subnetwork	0.52
ENSG00000113013	HSPA9 PPI subnetwork	0.52
ENSG00000114854	TNNC1 PPI subnetwork	0.52
GO:0009451	RNA modification	0.52
GO:0006383	transcription from RNA polymerase III promoter	0.52
ENSG00000120694	HSPH1 PPI subnetwork	0.52
MP:0001845	abnormal inflammatory response	0.52
ENSG00000151320	AKAP6 PPI subnetwork	0.52
ENSG00000056661	PCGF2 PPI subnetwork	0.52
ENSG00000151846	PABPC3 PPI subnetwork	0.52
GO:0016363	nuclear matrix	0.52
ENSG00000198104	OR2T6 PPI subnetwork	0.52
REACTOME_PHASE_1_:_FUNCTIONALIZATION_OF_COMPOUNDS	REACTOME_PHASE_1_:_FUNCTIONALIZATION_OF_COMPOUNDS	0.52
GO:0035253	ciliary rootlet	0.52
ENSG00000215769	ENSG00000215769 PPI subnetwork	0.52
ENSG00000182481	KPNA2 PPI subnetwork	0.52
KEGG_HEDGEHOG_SIGNALING_PATHWAY	KEGG_HEDGEHOG_SIGNALING_PATHWAY	0.52
ENSG00000114670	NEK11 PPI subnetwork	0.52
ENSG00000110713	NUP98 PPI subnetwork	0.52
ENSG00000107968	MAP3K8 PPI subnetwork	0.52
MP:0008396	abnormal osteoclast differentiation	0.52
ENSG00000026103	FAS PPI subnetwork	0.52
REACTOME_REV:MEDIATED_NUCLEAR_EXPORT_OF_HIV:1_RNA	REACTOME_REV:MEDIATED_NUCLEAR_EXPORT_OF_HIV:1_RNA	0.52
GO:0042119	neutrophil activation	0.53
ENSG00000143627	PKLR PPI subnetwork	0.53
GO:0019897	extrinsic to plasma membrane	0.53
GO:0072012	glomerulus vasculature development	0.53
MP:0001436	abnormal suckling behavior	0.53
MP:0010377	abnormal gut flora balance	0.53
GO:0021517	ventral spinal cord development	0.53
ENSG00000161960	EIF4A1 PPI subnetwork	0.53
KEGG_INTESTINAL_IMMUNE_NETWORK_FOR_IGA_PRODUCTION	KEGG_INTESTINAL_IMMUNE_NETWORK_FOR_IGA_PRODUCTION	0.53
ENSG00000105618	PRPF31 PPI subnetwork	0.53
REACTOME_MEIOSIS	REACTOME_MEIOSIS	0.53
ENSG00000124198	ARFGEF2 PPI subnetwork	0.53
GO:0022838	substrate-specific channel activity	0.53
ENSG00000119866	BCL11A PPI subnetwork	0.53
MP:0000107	abnormal frontal bone morphology	0.53
ENSG00000166170	BAG5 PPI subnetwork	0.53
ENSG00000141655	TNFRSF11A PPI subnetwork	0.53
ENSG00000115325	DOK1 PPI subnetwork	0.53
ENSG00000132305	IMMT PPI subnetwork	0.53
ENSG00000168036	CTNNB1 PPI subnetwork	0.53
GO:0010975	regulation of neuron projection development	0.53
ENSG00000131437	KIF3A PPI subnetwork	0.53
MP:0009339	decreased splenocyte number	0.53
GO:0000245	spliceosomal complex assembly	0.53

Original gene set ID	Original gene set description	Nominal P value
GO:0051324	prophase	0.53
GO:0031498	chromatin disassembly	0.53
GO:0032986	protein-DNA complex disassembly	0.53
GO:0006337	nucleosome disassembly	0.53
MP:0001394	circling	0.53
MP:0004542	impaired acrosome reaction	0.53
ENSG00000077782	FGFR1 PPI subnetwork	0.53
ENSG00000006125	AP2B1 PPI subnetwork	0.53
MP:0002413	abnormal megakaryocyte progenitor cell morphology	0.53
MP:0001006	abnormal retinal cone cell morphology	0.53
ENSG00000015479	MATR3 PPI subnetwork	0.53
ENSG00000198130	HIBCH PPI subnetwork	0.53
GO:0031058	positive regulation of histone modification	0.53
GO:0016050	vesicle organization	0.53
MP:0000692	small spleen	0.53
ENSG00000144158	ENSG00000144158 PPI subnetwork	0.53
GO:0030529	ribonucleoprotein complex	0.53
ENSG00000069956	MAPK6 PPI subnetwork	0.53
REACTOME_PREFOLDIN_MEDIATED_TRANSFER_OF_SUBSTRATE__TO_CCTTRIC	REACTOME_PREFOLDIN_MEDIATED_TRANSFER_OF_SUBSTRATE__TO_CCTTRIC	0.53
REACTOME_COOPERATION_OF_PREFOLDIN_AND_TRICCCT__IN_ACTIN_AND_TI	REACTOME_COOPERATION_OF_PREFOLDIN_AND_TRICCCT__IN_ACTIN_AND_TUBU	0.53
ENSG00000169592	INO80E PPI subnetwork	0.53
ENSG00000162552	WNT4 PPI subnetwork	0.53
ENSG00000133027	PEMT PPI subnetwork	0.53
ENSG00000185627	PSMD13 PPI subnetwork	0.53
GO:0030424	axon	0.53
KEGG_FC_EPSILON_RI_SIGNALING_PATHWAY	KEGG_FC_EPSILON_RI_SIGNALING_PATHWAY	0.53
GO:0002755	MyD88-dependent toll-like receptor signaling pathway	0.53
ENSG00000081052	COL4A4 PPI subnetwork	0.53
ENSG00000067596	DHX8 PPI subnetwork	0.53
MP:0000248	macrocytosis	0.53
REACTOME_GABA_SYNTHESIS_RELEASE_REUPTAKE_AND_DEGRADATION	REACTOME_GABA_SYNTHESIS_RELEASE_REUPTAKE_AND_DEGRADATION	0.53
ENSG00000160712	IL6R PPI subnetwork	0.53
GO:0005852	eukaryotic translation initiation factor 3 complex	0.53
GO:0048598	embryonic morphogenesis	0.53
ENSG00000001626	CFTR PPI subnetwork	0.53
ENSG00000005175	RPAP3 PPI subnetwork	0.53
GO:0042461	photoreceptor cell development	0.53
GO:0007128	meiotic prophase I	0.53
GO:0031063	regulation of histone deacetylation	0.53
GO:0001085	RNA polymerase II transcription factor binding	0.53
ENSG00000149557	FEZ1 PPI subnetwork	0.53
ENSG00000049540	ELN PPI subnetwork	0.53
ENSG00000206279	DAXX PPI subnetwork	0.53
ENSG00000206206	DAXX PPI subnetwork	0.53
ENSG00000204209	DAXX PPI subnetwork	0.53
GO:0090175	regulation of establishment of planar polarity	0.53
GO:0060071	Wnt receptor signaling pathway, planar cell polarity pathway	0.53
GO:0001843	neural tube closure	0.53
ENSG00000163877	SNIP1 PPI subnetwork	0.53

Original gene set ID	Original gene set description	Nominal P value
ENSG00000153487	ING1 PPI subnetwork	0.53
GO:0005261	cation channel activity	0.53
MP:0005330	cardiomyopathy	0.53
ENSG00000134245	WNT2B PPI subnetwork	0.53
REACTOME_ANTIGEN_PROCESSING:CROSS_PRESENTATION	REACTOME_ANTIGEN_PROCESSING:CROSS_PRESENTATION	0.53
GO:0060325	face morphogenesis	0.53
MP:0004485	increased response of heart to induced stress	0.53
ENSG00000185637	ENSG00000185637 PPI subnetwork	0.53
MP:0000131	abnormal long bone epiphysis morphology	0.53
ENSG00000130699	TAF4 PPI subnetwork	0.53
ENSG00000211762	ENSG00000211762 PPI subnetwork	0.53
GO:0006732	coenzyme metabolic process	0.53
ENSG00000075340	ADD2 PPI subnetwork	0.53
ENSG00000116641	DOCK7 PPI subnetwork	0.53
ENSG00000137752	CASP1 PPI subnetwork	0.53
ENSG00000091106	NLRC4 PPI subnetwork	0.53
MP:0000458	abnormal mandible morphology	0.53
ENSG00000000938	FGR PPI subnetwork	0.53
ENSG00000151576	QTRTD1 PPI subnetwork	0.53
ENSG00000161681	SHANK1 PPI subnetwork	0.53
MP:0002428	abnormal semicircular canal morphology	0.53
ENSG00000118137	APOA1 PPI subnetwork	0.53
ENSG00000215756	ENSG00000215756 PPI subnetwork	0.53
MP:0004322	abnormal sternebra morphology	0.53
MP:0008808	decreased spleen iron level	0.53
MP:0004409	abnormal crista ampullaris neuroepithelium morphology	0.53
MP:0002500	granulomatous inflammation	0.53
GO:0000502	proteasome complex	0.53
ENSG00000198042	MAK16 PPI subnetwork	0.53
REACTOME_SIGNALING_BY_SCF:KIT	REACTOME_SIGNALING_BY_SCF:KIT	0.53
MP:0005322	abnormal serotonin level	0.53
ENSG00000121083	DYNLL2 PPI subnetwork	0.53
MP:0008585	absent photoreceptor outer segment	0.53
MP:0004875	increased mean systemic arterial blood pressure	0.53
MP:0005122	increased circulating thyroid-stimulating hormone leve	0.53
ENSG00000100129	EIF3L PPI subnetwork	0.53
ENSG00000166401	SERPINB8 PPI subnetwork	0.53
GO:0000279	M phase	0.53
ENSG00000179218	CALR PPI subnetwork	0.53
REACTOME_FGFR1C_LIGAND_BINDING_AND_ACTIVATION	REACTOME_FGFR1C_LIGAND_BINDING_AND_ACTIVATION	0.53
ENSG00000163520	FBLN2 PPI subnetwork	0.53
ENSG00000146701	MDH2 PPI subnetwork	0.53
GO:0051188	cofactor biosynthetic process	0.53
GO:0044275	cellular carbohydrate catabolic process	0.53
REACTOME_SIGNALING_BY_VEGF	REACTOME_SIGNALING_BY_VEGF	0.54
REACTOME_VEGF_LIGAND:RECEPTOR_INTERACTIONS	REACTOME_VEGF_LIGAND:RECEPTOR_INTERACTIONS	0.54
ENSG00000162511	LAPTM5 PPI subnetwork	0.54
GO:0001837	epithelial to mesenchymal transition	0.54
MP:0010018	pulmonary vascular congestion	0.54

Original gene set ID	Original gene set description	Nominal P value
GO:0051904	pigment granule transport	0.54
GO:0001649	osteoblast differentiation	0.54
ENSG00000094880	CDC23 PPI subnetwork	0.54
GO:0004540	ribonuclease activity	0.54
REACTOME_SYNTHESIS_SECRETION_AND_DEACYLATION_OF_GHRELIN	REACTOME_SYNTHESIS_SECRETION_AND_DEACYLATION_OF_GHRELIN	0.54
MP:0002786	abnormal Leydig cell morphology	0.54
MP:0000925	abnormal floor plate morphology	0.54
ENSG00000164494	PDSS2 PPI subnetwork	0.54
REACTOME_INTERLEUKIN_RECEPTOR_SHC_SIGNALING	REACTOME_INTERLEUKIN_RECEPTOR_SHC_SIGNALING	0.54
GO:0071173	spindle assembly checkpoint	0.54
REACTOME_ACTIVATION_OF_THE_AP:1_FAMILY_OF_TRANSCRIPTION_FACTORS	REACTOME_ACTIVATION_OF_THE_AP:1_FAMILY_OF_TRANSCRIPTION_FACTORS	0.54
MP:0002908	delayed wound healing	0.54
GO:0007269	neurotransmitter secretion	0.54
ENSG00000124802	EEF1E1 PPI subnetwork	0.54
GO:0045121	membrane raft	0.54
REACTOME_FGFR1_LIGAND_BINDING_AND_ACTIVATION	REACTOME_FGFR1_LIGAND_BINDING_AND_ACTIVATION	0.54
GO:0019433	triglyceride catabolic process	0.54
MP:0001302	eyelids open at birth	0.54
ENSG00000198062	POTEH PPI subnetwork	0.54
ENSG00000164609	SLU7 PPI subnetwork	0.54
GO:0045926	negative regulation of growth	0.54
REACTOME_METABOLISM_OF_NUCLEOTIDES	REACTOME_METABOLISM_OF_NUCLEOTIDES	0.54
ENSG00000079785	DDX1 PPI subnetwork	0.54
MP:0005465	abnormal T-helper 1 physiology	0.54
KEGG_ALPHA_LINOLENIC_ACID_METABOLISM	KEGG_ALPHA_LINOLENIC_ACID_METABOLISM	0.54
GO:0035150	regulation of tube size	0.54
KEGG_LINOLEIC_ACID_METABOLISM	KEGG_LINOLEIC_ACID_METABOLISM	0.54
ENSG00000099942	CRKL PPI subnetwork	0.54
MP:0006000	abnormal corneal epithelium morphology	0.54
ENSG00000101868	POLA1 PPI subnetwork	0.54
GO:0009112	nucleobase metabolic process	0.54
ENSG00000039650	PNKP PPI subnetwork	0.54
ENSG00000174827	PDZK1 PPI subnetwork	0.54
KEGG_LEISHMANIA_INFECTION	KEGG_LEISHMANIA_INFECTION	0.54
KEGG_OOCYTE_MEIOSIS	KEGG_OOCYTE_MEIOSIS	0.54
GO:0009968	negative regulation of signal transduction	0.54
ENSG00000120885	CLU PPI subnetwork	0.54
MP:0003634	abnormal glial cell morphology	0.54
ENSG00000165417	GTF2A1 PPI subnetwork	0.54
ENSG00000172115	CYCS PPI subnetwork	0.54
GO:0006891	intra-Golgi vesicle-mediated transport	0.54
MP:0002843	decreased systemic arterial blood pressure	0.54
MP:0001524	impaired limb coordination	0.54
MP:0005311	abnormal circulating amino acid level	0.54
ENSG00000075673	ATP12A PPI subnetwork	0.54
GO:0008543	fibroblast growth factor receptor signaling pathway	0.54
MP:0003936	abnormal reproductive system development	0.54
MP:0002465	abnormal eosinophil physiology	0.54
ENSG00000119535	CSF3R PPI subnetwork	0.54

Original gene set ID	Original gene set description	Nominal P value
ENSG00000139618	BRCA2 PPI subnetwork	0.54
ENSG00000100239	PPP6R2 PPI subnetwork	0.54
ENSG00000112159	MDN1 PPI subnetwork	0.54
ENSG00000100109	TFIP11 PPI subnetwork	0.54
ENSG00000187990	HIST1H2BG PPI subnetwork	0.54
ENSG00000168242	HIST1H2BI PPI subnetwork	0.54
ENSG00000180596	HIST1H2BC PPI subnetwork	0.54
ENSG00000197846	HIST1H2BF PPI subnetwork	0.54
ENSG00000126261	UBA2 PPI subnetwork	0.54
ENSG00000011485	PPP5C PPI subnetwork	0.54
ENSG00000101146	RAE1 PPI subnetwork	0.54
ENSG00000163810	TGM4 PPI subnetwork	0.54
ENSG00000142208	AKT1 PPI subnetwork	0.54
ENSG00000170312	CDK1 PPI subnetwork	0.54
KEGG_PORPHYRIN_AND_CHLOROPHYLL_METABOLISM	KEGG_PORPHYRIN_AND_CHLOROPHYLL_METABOLISM	0.54
GO:0010948	negative regulation of cell cycle process	0.54
GO:0070555	response to interleukin-1	0.54
GO:0009982	pseudouridine synthase activity	0.54
GO:0030934	anchoring collagen	0.54
GO:0005253	anion channel activity	0.54
GO:0019841	retinol binding	0.54
GO:0006885	regulation of pH	0.54
MP:0002887	decreased susceptibility to pharmacologically induced seizures	0.54
ENSG00000163069	SGCB PPI subnetwork	0.54
MP:0008807	increased liver iron level	0.54
GO:0043202	lysosomal lumen	0.54
ENSG00000197903	HIST1H2BK PPI subnetwork	0.54
ENSG00000164086	DUSP7 PPI subnetwork	0.54
GO:0008287	protein serine/threonine phosphatase complex	0.54
ENSG00000174989	FBXW8 PPI subnetwork	0.54
GO:0034483	heparan sulfate sulfotransferase activity	0.54
ENSG00000177879	AP3S1 PPI subnetwork	0.54
GO:0021549	cerebellum development	0.54
GO:0008175	tRNA methyltransferase activity	0.54
KEGG_GRAFT_VERSUS_HOST_DISEASE	KEGG_GRAFT_VERSUS_HOST_DISEASE	0.54
GO:0031577	spindle checkpoint	0.54
GO:0008366	axon ensheathment	0.54
GO:0007272	ensheathment of neurons	0.54
GO:0016339	calcium-dependent cell-cell adhesion	0.54
ENSG00000129993	CBFA2T3 PPI subnetwork	0.54
MP:0001363	increased anxiety-related response	0.54
ENSG00000126067	PSMB2 PPI subnetwork	0.54
ENSG00000066933	MYO9A PPI subnetwork	0.54
GO:0071855	neuropeptide receptor binding	0.54
REACTOME_SIGNAL_AMPLIFICATION	REACTOME_SIGNAL_AMPLIFICATION	0.54
ENSG00000006468	ETV1 PPI subnetwork	0.54
GO:0071295	cellular response to vitamin	0.54
ENSG00000111348	ARHGDIB PPI subnetwork	0.54
ENSG00000143437	ARNT PPI subnetwork	0.54

Original gene set ID	Original gene set description	Nominal P value
ENSG00000160255	ITGB2 PPI subnetwork	0.54
GO:0071941	nitrogen cycle metabolic process	0.54
MP:0001270	distended abdomen	0.54
ENSG00000180185	FAHD1 PPI subnetwork	0.54
MP:0000460	mandible hypoplasia	0.54
KEGG_PROXIMAL_TUBULE_BICARBONATE_RECLAMATION	KEGG_PROXIMAL_TUBULE_BICARBONATE_RECLAMATION	0.54
ENSG00000184117	NIPSNAP1 PPI subnetwork	0.54
ENSG00000146232	NFKBIE PPI subnetwork	0.54
MP:0005104	abnormal tarsal bone morphology	0.54
GO:0050795	regulation of behavior	0.54
MP:0004753	abnormal miniature excitatory postsynaptic currents	0.54
ENSG00000113810	SMC4 PPI subnetwork	0.55
ENSG00000130706	ADRM1 PPI subnetwork	0.55
MP:0001489	decreased startle reflex	0.55
MP:0001762	polyuria	0.55
ENSG00000140600	SH3GL3 PPI subnetwork	0.55
GO:0045737	positive regulation of cyclin-dependent protein kinase activity	0.55
ENSG00000096063	SRPK1 PPI subnetwork	0.55
ENSG00000120087	HOXB7 PPI subnetwork	0.55
MP:0002123	abnormal hematopoiesis	0.55
ENSG00000177485	ZBTB33 PPI subnetwork	0.55
MP:0005466	abnormal T-helper 2 physiology	0.55
GO:0007130	synaptonemal complex assembly	0.55
GO:0010562	positive regulation of phosphorus metabolic process	0.55
GO:0045937	positive regulation of phosphate metabolic process	0.55
GO:0046634	regulation of alpha-beta T cell activation	0.55
GO:0044057	regulation of system process	0.55
GO:0061077	chaperone-mediated protein folding	0.55
GO:0005507	copper ion binding	0.55
ENSG00000175467	SART1 PPI subnetwork	0.55
GO:0008633	activation of pro-apoptotic gene products	0.55
GO:0019887	protein kinase regulator activity	0.55
GO:0030902	hindbrain development	0.55
GO:0005267	potassium channel activity	0.55
GO:0032535	regulation of cellular component size	0.55
MP:0009790	decreased susceptibility to viral infection induced morbidity/mortality	0.55
GO:0030867	rough endoplasmic reticulum membrane	0.55
GO:0043603	cellular amide metabolic process	0.55
GO:0046631	alpha-beta T cell activation	0.55
MP:0003606	kidney failure	0.55
MP:0001523	impaired righting response	0.55
GO:0032320	positive regulation of Ras GTPase activity	0.55
ENSG00000084234	APLP2 PPI subnetwork	0.55
GO:0007398	ectoderm development	0.55
ENSG00000125378	BMP4 PPI subnetwork	0.55
ENSG00000165059	PRKACG PPI subnetwork	0.55
GO:0045086	positive regulation of interleukin-2 biosynthetic process	0.55
MP:0003890	abnormal embryonic-extraembryonic boundary morphology	0.55
ENSG00000198791	CNOT7 PPI subnetwork	0.55

Original gene set ID	Original gene set description	Nominal P value
ENSG00000008083	JARID2 PPI subnetwork	0.55
MP:0005658	increased susceptibility to diet-induced obesity	0.55
ENSG00000136243	NUPL2 PPI subnetwork	0.55
ENSG00000051128	HOMER3 PPI subnetwork	0.55
ENSG00000100591	AHSA1 PPI subnetwork	0.55
MP:0001046	abnormal enteric neuron morphology	0.55
ENSG00000166716	ZNF592 PPI subnetwork	0.55
ENSG00000074319	TSG101 PPI subnetwork	0.55
GO:0016337	cell-cell adhesion	0.55
GO:0051130	positive regulation of cellular component organization	0.55
ENSG00000143549	TPM3 PPI subnetwork	0.55
GO:0016408	C-acyltransferase activity	0.55
GO:0051592	response to calcium ion	0.55
ENSG00000103126	AXIN1 PPI subnetwork	0.55
GO:0046425	regulation of JAK-STAT cascade	0.55
GO:0051403	stress-activated MAPK cascade	0.55
ENSG00000159110	IFNAR2 PPI subnetwork	0.55
ENSG00000117400	MPL PPI subnetwork	0.55
GO:0035023	regulation of Rho protein signal transduction	0.55
ENSG00000146674	IGFBP3 PPI subnetwork	0.55
REACTOME_CD28_DEPENDENT_VAV1_PATHWAY	REACTOME_CD28_DEPENDENT_VAV1_PATHWAY	0.55
ENSG00000157110	RBPMS PPI subnetwork	0.55
GO:0008417	fucosyltransferase activity	0.55
GO:0030414	peptidase inhibitor activity	0.55
ENSG00000072778	ACADVL PPI subnetwork	0.55
GO:0004177	aminopeptidase activity	0.55
ENSG00000141384	TAF4B PPI subnetwork	0.55
MP:0001513	limb grasping	0.55
MP:0002022	increased lymphoma incidence	0.55
ENSG00000137656	BUD13 PPI subnetwork	0.55
GO:0001912	positive regulation of leukocyte mediated cytotoxicity	0.55
MP:0000780	abnormal corpus callosum morphology	0.55
ENSG00000152582	SPEF2 PPI subnetwork	0.55
ENSG00000143947	RPS27A PPI subnetwork	0.55
ENSG00000047249	ATP6V1H PPI subnetwork	0.55
GO:0021846	cell proliferation in forebrain	0.55
ENSG00000138750	NUP54 PPI subnetwork	0.55
ENSG00000119718	EIF2B2 PPI subnetwork	0.55
GO:0043101	purine-containing compound salvage	0.55
GO:0005216	ion channel activity	0.55
GO:0010595	positive regulation of endothelial cell migration	0.55
MP:0004978	decreased B-1 B cell number	0.55
ENSG00000167508	MVD PPI subnetwork	0.55
ENSG00000072401	UBE2D1 PPI subnetwork	0.55
MP:0000864	abnormal cerebellum vermis morphology	0.55
MP:0010375	increased kidney iron level	0.55
ENSG00000139687	RB1 PPI subnetwork	0.55
MP:0002544	brachydactyly	0.55
MP:0005627	increased circulating potassium level	0.55

Original gene set ID	Original gene set description	Nominal P value
ENSG00000104419	NDRG1 PPI subnetwork	0.55
MP:0000245	abnormal erythropoiesis	0.55
ENSG00000117601	SERPINC1 PPI subnetwork	0.55
GO:0060485	mesenchyme development	0.55
GO:0042575	DNA polymerase complex	0.55
MP:0001120	abnormal uterus morphology	0.55
ENSG00000108055	SMC3 PPI subnetwork	0.55
ENSG00000102974	CTCF PPI subnetwork	0.55
MP:0004939	abnormal B cell morphology	0.55
GO:0005100	Rho GTPase activator activity	0.55
MP:0008528	polycystic kidney	0.55
MP:0009862	abnormal aorta elastic tissue morphology	0.55
MP:0004876	decreased mean systemic arterial blood pressure	0.55
GO:0007017	microtubule-based process	0.55
GO:0003007	heart morphogenesis	0.55
ENSG00000111262	KCNA1 PPI subnetwork	0.56
MP:0005159	azoospermia	0.56
ENSG00000180628	PCGF5 PPI subnetwork	0.56
ENSG00000177469	PTRF PPI subnetwork	0.56
ENSG00000122965	RBM19 PPI subnetwork	0.56
GO:0046658	anchored to plasma membrane	0.56
GO:0042625	ATPase activity, coupled to transmembrane movement of ions	0.56
ENSG00000162946	DISC1 PPI subnetwork	0.56
ENSG00000085231	TAF9 PPI subnetwork	0.56
ENSG00000069431	ABCC9 PPI subnetwork	0.56
GO:0006182	cGMP biosynthetic process	0.56
ENSG00000123374	CDK2 PPI subnetwork	0.56
REACTOME_CYTOSOLIC_TRNA_AMINOACYLATION	REACTOME_CYTOSOLIC_TRNA_AMINOACYLATION	0.56
REACTOME_SCFSKP2:MEDIATED_DEGRADATION_OF_P27P21	REACTOME_SCFSKP2:MEDIATED_DEGRADATION_OF_P27P21	0.56
MP:0011100	complete preweaning lethality	0.56
MP:0002176	increased brain weight	0.56
REACTOME_CGMP_EFFECTS	REACTOME_CGMP_EFFECTS	0.56
ENSG00000092203	TOX4 PPI subnetwork	0.56
ENSG00000165527	ARF6 PPI subnetwork	0.56
ENSG00000067704	IARS2 PPI subnetwork	0.56
GO:0090312	positive regulation of protein deacetylation	0.56
GO:2000027	regulation of organ morphogenesis	0.56
GO:0045177	apical part of cell	0.56
MP:0004779	abnormal production of surfactant	0.56
ENSG00000106976	DNM1 PPI subnetwork	0.56
ENSG00000106348	IMPDH1 PPI subnetwork	0.56
ENSG00000174446	SNAPC5 PPI subnetwork	0.56
ENSG00000167751	KLK2 PPI subnetwork	0.56
ENSG00000102572	STK24 PPI subnetwork	0.56
GO:0003950	NAD+ ADP-ribosyltransferase activity	0.56
ENSG00000159840	ZYX PPI subnetwork	0.56
ENSG00000105664	COMP PPI subnetwork	0.56
ENSG00000165684	SNAPC4 PPI subnetwork	0.56
GO:0061387	regulation of extent of cell growth	0.56

Original gene set ID	Original gene set description	Nominal P value
REACTOME_MITOTIC_SPINDLE_CHECKPOINT	REACTOME_MITOTIC_SPINDLE_CHECKPOINT	0.56
REACTOME_UNBLOCKING_OF_NMDA_RECEPTOR_GLUTAMATE_BINDING_AND_	REACTOME_UNBLOCKING_OF_NMDA_RECEPTOR_GLUTAMATE_BINDING_AND_AC	0.56
MP:0002621	delayed neural tube closure	0.56
ENSG00000101558	VAPA PPI subnetwork	0.56
GO:0007040	lysosome organization	0.56
ENSG00000097046	CDC7 PPI subnetwork	0.56
MP:0008703	decreased interleukin-5 secretion	0.56
MP:0004486	decreased response of heart to induced stress	0.56
ENSG00000101189	C20orf20 PPI subnetwork	0.56
REACTOME_G1S:SPECIFIC_TRANSCRIPTION	REACTOME_G1S:SPECIFIC_TRANSCRIPTION	0.56
ENSG00000099341	PSMD8 PPI subnetwork	0.56
GO:0043534	blood vessel endothelial cell migration	0.56
ENSG00000106211	HSPB1 PPI subnetwork	0.56
ENSG00000198380	GFPT1 PPI subnetwork	0.56
GO:0005254	chloride channel activity	0.56
ENSG00000171867	PRNP PPI subnetwork	0.56
GO:0032368	regulation of lipid transport	0.56
ENSG00000206232	ENSG00000206232 PPI subnetwork	0.56
ENSG00000204261	ENSG00000204261 PPI subnetwork	0.56
ENSG00000206296	ENSG00000206296 PPI subnetwork	0.56
GO:0016776	phosphotransferase activity, phosphate group as acceptor	0.56
ENSG00000129559	NEDD8 PPI subnetwork	0.56
ENSG00000152661	GJA1 PPI subnetwork	0.56
GO:0016709	oxidoreductase activity, acting on paired donors, with incorporation or reduction of	0.56
GO:0035050	embryonic heart tube development	0.56
ENSG00000175084	DES PPI subnetwork	0.56
GO:0007588	excretion	0.56
GO:0050770	regulation of axonogenesis	0.56
ENSG00000114251	WNT5A PPI subnetwork	0.56
MP:0000111	cleft palate	0.56
ENSG00000155561	NUP205 PPI subnetwork	0.56
ENSG00000198018	ENTPD7 PPI subnetwork	0.56
REACTOME_P2Y_RECEPTORS	REACTOME_P2Y_RECEPTORS	0.56
GO:0043498	cell surface binding	0.56
MP:0008189	increased transitional stage B cell number	0.56
GO:0007059	chromosome segregation	0.56
GO:0008023	transcription elongation factor complex	0.56
MP:0005324	ascites	0.56
KEGG_FRUCTOSE_AND_MANNOSE_METABOLISM	KEGG_FRUCTOSE_AND_MANNOSE_METABOLISM	0.56
ENSG00000167930	ITFG3 PPI subnetwork	0.56
GO:0016514	SWI/SNF complex	0.56
GO:0016055	Wnt receptor signaling pathway	0.56
MP:0005334	abnormal fat pad morphology	0.56
ENSG00000108379	WNT3 PPI subnetwork	0.56
MP:0005464	abnormal platelet physiology	0.56
GO:0016064	immunoglobulin mediated immune response	0.56
GO:0032609	interferon-gamma production	0.56
GO:0035162	embryonic hemopoiesis	0.56
ENSG00000103168	TAF1C PPI subnetwork	0.56

Original gene set ID	Original gene set description	Nominal P value
MP:0006269	abnormal mammary gland growth during pregnancy	0.56
REACTOME_PHOSPHORYLATION_OF_THE_APCC	REACTOME_PHOSPHORYLATION_OF_THE_APCC	0.56
MP:0004090	abnormal sarcomere morphology	0.56
ENSG00000172794	RAB37 PPI subnetwork	0.56
GO:0042733	embryonic digit morphogenesis	0.56
REACTOME_DNA_REPLICATION_PRE:INITIATION	REACTOME_DNA_REPLICATION_PRE:INITIATION	0.56
REACTOME_MG1_TRANSITION	REACTOME_MG1_TRANSITION	0.56
ENSG00000134982	APC PPI subnetwork	0.56
GO:0045980	negative regulation of nucleotide metabolic process	0.56
GO:0008483	transaminase activity	0.56
MP:0001714	absent trophoblast giant cells	0.56
MP:0002950	abnormal neural crest cell migration	0.56
ENSG00000172116	CD8B PPI subnetwork	0.56
ENSG00000111186	WNT5B PPI subnetwork	0.56
GO:0015485	cholesterol binding	0.56
ENSG00000130414	NDUFA10 PPI subnetwork	0.56
GO:0003197	endocardial cushion development	0.56
GO:0008016	regulation of heart contraction	0.56
ENSG00000131238	PPT1 PPI subnetwork	0.56
MP:0009331	absent primitive node	0.56
GO:0032755	positive regulation of interleukin-6 production	0.56
ENSG00000198925	ATG9A PPI subnetwork	0.57
MP:0005326	abnormal podocyte morphology	0.57
GO:0005506	iron ion binding	0.57
ENSG00000123737	EXOSC9 PPI subnetwork	0.57
GO:0070646	protein modification by small protein remova	0.57
ENSG00000010030	ETV7 PPI subnetwork	0.57
ENSG00000197321	SVIL PPI subnetwork	0.57
ENSG00000197697	HIST1H2BE PPI subnetwork	0.57
ENSG00000138741	TRPC3 PPI subnetwork	0.57
GO:0001710	mesodermal cell fate commitment	0.57
ENSG00000124789	NUP153 PPI subnetwork	0.57
GO:0030100	regulation of endocytosis	0.57
GO:0051325	interphase	0.57
ENSG00000092964	DPYSL2 PPI subnetwork	0.57
GO:0015370	solute:sodium symporter activity	0.57
GO:0004576	oligosaccharyl transferase activity	0.57
REACTOME_VPU_MEDIATED_DEGRADATION_OF_CD4	REACTOME_VPU_MEDIATED_DEGRADATION_OF_CD4	0.57
KEGG_HOMOLOGOUS_RECOMBINATION	KEGG_HOMOLOGOUS_RECOMBINATION	0.57
GO:0016010	dystrophin-associated glycoprotein complex	0.57
REACTOME_CREB_PHOSPHORYLATION_THROUGH_THE_ACTIVATION_OF_CAM	REACTOME_CREB_PHOSPHORYLATION_THROUGH_THE_ACTIVATION_OF_CAMKII	0.57
GO:0042770	signal transduction in response to DNA damage	0.57
MP:0000913	abnormal brain development	0.57
ENSG00000136754	ABI1 PPI subnetwork	0.57
GO:0042692	muscle cell differentiation	0.57
REACTOME_KINESINS	REACTOME_KINESINS	0.57
ENSG00000178607	ERN1 PPI subnetwork	0.57
GO:0042805	actinin binding	0.57
GO:0006297	nucleotide-excision repair, DNA gap filling	0.57

Original gene set ID	Original gene set description	Nominal P value
ENSG00000122884	P4HA1 PPI subnetwork	0.57
GO:0003143	embryonic heart tube morphogenesis	0.57
ENSG00000153234	NR4A2 PPI subnetwork	0.57
ENSG00000135363	LMO2 PPI subnetwork	0.57
GO:0019894	kinesin binding	0.57
GO:0042440	pigment metabolic process	0.57
GO:0060538	skeletal muscle organ development	0.57
GO:0006275	regulation of DNA replication	0.57
ENSG00000100079	LGALS2 PPI subnetwork	0.57
REACTOME_ACTIVATION_OF_CHAPERONES_BY_ATF6:ALPHA	REACTOME_ACTIVATION_OF_CHAPERONES_BY_ATF6:ALPHA	0.57
GO:0016604	nuclear body	0.57
MP:0002199	abnormal brain commissure morphology	0.57
ENSG00000165996	PTPLA PPI subnetwork	0.57
GO:0004115	3',5'-cyclic-AMP phosphodiesterase activity	0.57
ENSG00000175792	RUVBL1 PPI subnetwork	0.57
ENSG00000111752	PHC1 PPI subnetwork	0.57
MP:0002085	abnormal embryonic tissue morphology	0.57
ENSG00000114982	KANSL3 PPI subnetwork	0.57
GO:0010745	negative regulation of macrophage derived foam cell differentiation	0.57
GO:0007094	mitotic cell cycle spindle assembly checkpoint	0.57
ENSG00000108823	SGCA PPI subnetwork	0.57
GO:0008395	steroid hydroxylase activity	0.57
GO:0001754	eye photoreceptor cell differentiation	0.57
GO:0032393	MHC class I receptor activity	0.57
REACTOME_CELL_CYCLE_MITOTIC	REACTOME_CELL_CYCLE_MITOTIC	0.57
GO:0030193	regulation of blood coagulation	0.57
GO:0019239	deaminase activity	0.57
MP:0005341	decreased susceptibility to atherosclerosis	0.57
ENSG00000102054	RBBP7 PPI subnetwork	0.57
MP:0001182	lung hemorrhage	0.57
GO:0021904	dorsal/ventral neural tube patterning	0.57
ENSG00000166478	ZNF143 PPI subnetwork	0.57
KEGG_BASAL_TRANSCRIPTION_FACTORS	KEGG_BASAL_TRANSCRIPTION_FACTORS	0.57
GO:0048566	embryonic digestive tract development	0.57
MP:0000788	abnormal cerebral cortex morphology	0.57
ENSG00000144285	SCN1A PPI subnetwork	0.57
MP:0000562	polydactyly	0.57
REACTOME_ION_TRANSPORT_BY_P:TYPE_ATPASES	REACTOME_ION_TRANSPORT_BY_P:TYPE_ATPASES	0.57
ENSG00000105287	PRKD2 PPI subnetwork	0.57
ENSG00000181610	MRPS23 PPI subnetwork	0.57
ENSG00000116478	HDAC1 PPI subnetwork	0.57
GO:0030901	midbrain development	0.57
ENSG00000158517	NCF1 PPI subnetwork	0.57
ENSG00000152818	UTRN PPI subnetwork	0.57
MP:0008076	abnormal CD4-positive T cell differentiation	0.57
KEGG_LYSINE_DEGRADATION	KEGG_LYSINE_DEGRADATION	0.57
MP:0011346	renal tubule atrophy	0.57
ENSG00000142599	RERE PPI subnetwork	0.57
REACTOME_METABOLISM_OF_NON:CODING_RNA	REACTOME_METABOLISM_OF_NON:CODING_RNA	0.57

Original gene set ID	Original gene set description	Nominal P value
REACTOME_SNRNP_ASSEMBLY	REACTOME_SNRNP_ASSEMBLY	0.57
ENSG00000159199	ATP5G1 PPI subnetwork	0.57
KEGG_CALCIUM_SIGNALING_PATHWAY	KEGG_CALCIUM_SIGNALING_PATHWAY	0.57
GO:0002039	p53 binding	0.57
ENSG00000148798	INA PPI subnetwork	0.57
GO:0030551	cyclic nucleotide binding	0.57
ENSG00000150347	ARID5B PPI subnetwork	0.57
GO:0032147	activation of protein kinase activity	0.57
ENSG00000188064	WNT7B PPI subnetwork	0.57
MP:0005307	head tossing	0.58
GO:0060047	heart contraction	0.58
ENSG00000163288	GABRB1 PPI subnetwork	0.58
GO:0051048	negative regulation of secretion	0.58
ENSG00000012223	LTF PPI subnetwork	0.58
GO:0045765	regulation of angiogenesis	0.58
ENSG00000089094	KDM2B PPI subnetwork	0.58
GO:0002504	antigen processing and presentation of peptide or polysaccharide antigen via MHC c	0.58
ENSG00000186676	ENSG00000186676 PPI subnetwork	0.58
MP:0001876	decreased inflammatory response	0.58
GO:0046632	alpha-beta T cell differentiation	0.58
ENSG00000109339	MAPK10 PPI subnetwork	0.58
ENSG00000171564	FGB PPI subnetwork	0.58
GO:0010165	response to X-ray	0.58
ENSG00000135365	PHF21A PPI subnetwork	0.58
MP:0000633	abnormal pituitary gland morphology	0.58
MP:0000334	decreased granulocyte number	0.58
GO:0004984	olfactory receptor activity	0.58
GO:0034446	substrate adhesion-dependent cell spreading	0.58
ENSG00000136930	PSMB7 PPI subnetwork	0.58
ENSG00000139190	VAMP1 PPI subnetwork	0.58
ENSG00000075884	ARHGAP15 PPI subnetwork	0.58
REACTOME_BIOLOGICAL_OXIDATIONS	REACTOME_BIOLOGICAL_OXIDATIONS	0.58
ENSG00000115641	FHL2 PPI subnetwork	0.58
GO:0016409	palmitoyltransferase activity	0.58
MP:0002230	abnormal primitive streak formation	0.58
GO:0048306	calcium-dependent protein binding	0.58
GO:0001934	positive regulation of protein phosphorylation	0.58
ENSG00000115361	ACADL PPI subnetwork	0.58
REACTOME_VPR:MEDIATED_NUCLEAR_IMPORT_OF_PICS	REACTOME_VPR:MEDIATED_NUCLEAR_IMPORT_OF_PICS	0.58
GO:0030593	neutrophil chemotaxis	0.58
MP:0002651	abnormal sciatic nerve morphology	0.58
MP:0002233	abnormal nose morphology	0.58
ENSG00000075089	ACTR6 PPI subnetwork	0.58
ENSG00000107581	EIF3A PPI subnetwork	0.58
REACTOME_INTERACTIONS_OF_REV_WITH_HOST_CELLULAR_PROTEINS	REACTOME_INTERACTIONS_OF_REV_WITH_HOST_CELLULAR_PROTEINS	0.58
GO:0015116	sulfate transmembrane transporter activity	0.58
ENSG00000095794	CREM PPI subnetwork	0.58
GO:0010769	regulation of cell morphogenesis involved in differentiation	0.58
GO:0071363	cellular response to growth factor stimulus	0.58

Original gene set ID	Original gene set description	Nominal P value
MP:0004405	absent cochlear hair cells	0.58
GO:0043583	ear development	0.58
MP:0004261	abnormal embryonic neuroepithelium morphology	0.58
REACTOME_ANTIGEN_PROCESSING_UBIQUITINATION__PROTEASOME_DEGRAI	REACTOME_ANTIGEN_PROCESSING_UBIQUITINATION__PROTEASOME_DEGRADATI	0.58
GO:0031514	motile cilium	0.58
GO:0046415	urate metabolic process	0.58
ENSG00000114867	EIF4G1 PPI subnetwork	0.58
ENSG00000198677	TTC37 PPI subnetwork	0.58
ENSG00000116717	GADD45A PPI subnetwork	0.58
REACTOME_CLASS_I_MHC_MEDIATED_ANTIGEN_PROCESSING__PRESENTATIOI	REACTOME_CLASS_I_MHC_MEDIATED_ANTIGEN_PROCESSING__PRESENTATION	0.58
GO:0033119	negative regulation of RNA splicing	0.58
MP:0000914	exencephaly	0.58
GO:0048659	smooth muscle cell proliferation	0.58
GO:0060271	cilium morphogenesis	0.58
MP:0004774	abnormal bile salt level	0.58
MP:0001695	abnormal gastrulation	0.58
ENSG00000131504	DIAPH1 PPI subnetwork	0.58
GO:0048762	mesenchymal cell differentiation	0.58
REACTOME_ACTIVATION_OF_BH3:ONLY_PROTEINS	REACTOME_ACTIVATION_OF_BH3:ONLY_PROTEINS	0.58
ENSG00000073969	NSF PPI subnetwork	0.58
GO:0005548	phospholipid transporter activity	0.58
ENSG00000215120	ENSG00000215120 PPI subnetwork	0.58
ENSG00000134086	VHL PPI subnetwork	0.58
ENSG00000134058	CDK7 PPI subnetwork	0.58
MP:0004924	abnormal behavior	0.58
ENSG00000163531	NFASC PPI subnetwork	0.58
REACTOME_CYTOCHROME_P450_:_ARRANGED_BY_SUBSTRATE_TYPE	REACTOME_CYTOCHROME_P450_:_ARRANGED_BY_SUBSTRATE_TYPE	0.58
ENSG00000103051	COG4 PPI subnetwork	0.58
ENSG00000137345	MOG PPI subnetwork	0.58
ENSG00000206456	ENSG00000206456 PPI subnetwork	0.58
ENSG00000204655	MOG PPI subnetwork	0.58
GO:0019915	lipid storage	0.58
MP:0001654	hepatic necrosis	0.58
MP:0001328	disorganized retinal layers	0.58
GO:0000726	non-recombinational repair	0.58
ENSG00000185787	MORF4L1 PPI subnetwork	0.58
MP:0006011	abnormal endolymphatic duct morphology	0.58
REACTOME_ER:PHAGOSOME_PATHWAY	REACTOME_ER:PHAGOSOME_PATHWAY	0.58
ENSG00000123080	CDKN2C PPI subnetwork	0.58
REACTOME_SYNTHESIS_OF_DNA	REACTOME_SYNTHESIS_OF_DNA	0.58
GO:0035145	exon-exon junction complex	0.58
GO:0015079	potassium ion transmembrane transporter activity	0.58
GO:0050730	regulation of peptidyl-tyrosine phosphorylatior	0.58
MP:0008388	hypochromic microcytic anemia	0.58
GO:0055008	cardiac muscle tissue morphogenesis	0.58
GO:0045664	regulation of neuron differentiatior	0.58
ENSG00000168685	IL7R PPI subnetwork	0.58
KEGG_UBIQUITIN_MEDIATED_PROTEOLYSIS	KEGG_UBIQUITIN_MEDIATED_PROTEOLYSIS	0.58
ENSG00000044115	CTNNA1 PPI subnetwork	0.58

Original gene set ID	Original gene set description	Nominal P value
ENSG00000123338	NCKAP1L PPI subnetwork	0.58
GO:0042611	MHC protein complex	0.58
ENSG00000065613	SLK PPI subnetwork	0.58
ENSG00000133318	RTN3 PPI subnetwork	0.58
GO:0030818	negative regulation of cAMP biosynthetic process	0.58
GO:0030815	negative regulation of cAMP metabolic process	0.58
GO:0003015	heart process	0.58
GO:0032321	positive regulation of Rho GTPase activity	0.58
ENSG00000182533	CAV3 PPI subnetwork	0.58
ENSG00000143632	ACTA1 PPI subnetwork	0.58
ENSG00000008277	ADAM22 PPI subnetwork	0.58
REACTOME_EXPORT_OF_VIRAL_RIBONUCLEOPROTEINS_FROM_NUCLEUS	REACTOME_EXPORT_OF_VIRAL_RIBONUCLEOPROTEINS_FROM_NUCLEUS	0.58
GO:0045807	positive regulation of endocytosis	0.58
GO:0018212	peptidyl-tyrosine modification	0.58
GO:0034374	low-density lipoprotein particle remodeling	0.58
GO:0070851	growth factor receptor binding	0.58
GO:0006607	NLS-bearing substrate import into nucleus	0.59
ENSG00000131711	MAP1B PPI subnetwork	0.59
GO:0070585	protein localization in mitochondrion	0.59
REACTOME_MTOR_SIGNALLING	REACTOME_MTOR_SIGNALLING	0.59
MP:0010763	abnormal hematopoietic stem cell physiology	0.59
ENSG00000198087	CD2AP PPI subnetwork	0.59
REACTOME_MITOTIC_G1:G1S_PHASES	REACTOME_MITOTIC_G1:G1S_PHASES	0.59
GO:0031346	positive regulation of cell projection organization	0.59
ENSG00000023608	SNAPC1 PPI subnetwork	0.59
ENSG00000183735	TBK1 PPI subnetwork	0.59
ENSG00000112312	GMNN PPI subnetwork	0.59
GO:0016049	cell growth	0.59
GO:0001558	regulation of cell growth	0.59
GO:0046875	ephrin receptor binding	0.59
ENSG00000077238	IL4R PPI subnetwork	0.59
ENSG00000196305	IARS PPI subnetwork	0.59
ENSG00000136738	STAM PPI subnetwork	0.59
GO:0032649	regulation of interferon-gamma production	0.59
GO:0002294	CD4-positive, alpha-beta T cell differentiation involved in immune response	0.59
GO:0042093	T-helper cell differentiation	0.59
ENSG00000185359	HGS PPI subnetwork	0.59
GO:0030111	regulation of Wnt receptor signaling pathway	0.59
KEGG_INOSITOL_PHOSPHATE_METABOLISM	KEGG_INOSITOL_PHOSPHATE_METABOLISM	0.59
GO:0048538	thymus development	0.59
ENSG00000171566	PLRG1 PPI subnetwork	0.59
GO:0030120	vesicle coat	0.59
ENSG00000197459	HIST1H2BH PPI subnetwork	0.59
ENSG00000167645	YIF1B PPI subnetwork	0.59
MP:0002693	abnormal pancreas physiology	0.59
GO:0051259	protein oligomerization	0.59
GO:0000302	response to reactive oxygen species	0.59
ENSG00000170142	UBE2E1 PPI subnetwork	0.59
ENSG00000185619	PCGF3 PPI subnetwork	0.59

Original gene set ID	Original gene set description	Nominal P value
ENSG00000160007	ARHGAP35 PPI subnetwork	0.59
KEGG_PPAR_SIGNALING_PATHWAY	KEGG_PPAR_SIGNALING_PATHWAY	0.59
ENSG00000175602	CCDC85B PPI subnetwork	0.59
MP:0000233	abnormal blood flow velocity	0.59
MP:0006113	abnormal heart septum morphology	0.59
KEGG_PROPANOATE_METABOLISM	KEGG_PROPANOATE_METABOLISM	0.59
GO:0009743	response to carbohydrate stimulus	0.59
MP:0001263	weight loss	0.59
GO:0048660	regulation of smooth muscle cell proliferation	0.59
GO:0070830	tight junction assembly	0.59
GO:0018108	peptidyl-tyrosine phosphorylation	0.59
MP:0003733	abnormal retinal inner nuclear layer morphology	0.59
GO:0050818	regulation of coagulation	0.59
GO:0048934	peripheral nervous system neuron differentiation	0.59
GO:0048935	peripheral nervous system neuron development	0.59
ENSG00000086619	ERO1LB PPI subnetwork	0.59
ENSG00000198700	IPO9 PPI subnetwork	0.59
ENSG00000130208	APOC1 PPI subnetwork	0.59
ENSG00000067606	PRKCZ PPI subnetwork	0.59
GO:0003179	heart valve morphogenesis	0.59
REACTOME_CYCLIN_ACDK2:ASSOCIATED_EVENTS_AT_S_PHASE_ENTRY	REACTOME_CYCLIN_ACDK2:ASSOCIATED_EVENTS_AT_S_PHASE_ENTRY	0.59
GO:0005761	mitochondrial ribosome	0.59
GO:0000313	organellar ribosome	0.59
GO:0043198	dendritic shaft	0.59
ENSG00000151164	RAD9B PPI subnetwork	0.59
ENSG00000117594	HSD11B1 PPI subnetwork	0.59
ENSG00000157087	ATP2B2 PPI subnetwork	0.59
MP:0000848	abnormal pons morphology	0.59
GO:0042327	positive regulation of phosphorylation	0.59
ENSG00000113575	PPP2CA PPI subnetwork	0.59
MP:0005566	decreased blood urea nitrogen level	0.59
ENSG00000077522	ACTN2 PPI subnetwork	0.59
REACTOME_INHIBITION_OF_THE_PROTEOLYTIC_ACTIVITY_OF_APCC_REQUIRED	REACTOME_INHIBITION_OF_THE_PROTEOLYTIC_ACTIVITY_OF_APCC_REQUIRED_FO	0.59
REACTOME_INACTIVATION_OF_APCC_VIA_DIRECT_INHIBITION_OF_THE_APCC_	REACTOME_INACTIVATION_OF_APCC_VIA_DIRECT_INHIBITION_OF_THE_APCC_CO	0.59
ENSG00000088833	NSFL1C PPI subnetwork	0.59
KEGG_AUTOIMMUNE_THYROID_DISEASE	KEGG_AUTOIMMUNE_THYROID_DISEASE	0.59
ENSG00000106829	TLE4 PPI subnetwork	0.59
GO:0019884	antigen processing and presentation of exogenous antigen	0.59
GO:0030135	coated vesicle	0.59
MP:0003644	thymus atrophy	0.59
ENSG00000173566	NUDT18 PPI subnetwork	0.59
GO:0015645	fatty acid ligase activity	0.59
ENSG00000099960	SLC7A4 PPI subnetwork	0.59
ENSG00000013293	SLC7A14 PPI subnetwork	0.59
ENSG00000173786	CNP PPI subnetwork	0.59
GO:0050679	positive regulation of epithelial cell proliferation	0.59
GO:0046427	positive regulation of JAK-STAT cascade	0.59
ENSG00000162692	VCAM1 PPI subnetwork	0.59
GO:0007350	blastoderm segmentation	0.59

Original gene set ID	Original gene set description	Nominal P value
KEGG_PROTEASOME	KEGG_PROTEASOME	0.59
ENSG00000197860	SGTB PPI subnetwork	0.59
REACTOME_LIGAND:GATED_ION_CHANNEL_TRANSPORT	REACTOME_LIGAND:GATED_ION_CHANNEL_TRANSPORT	0.59
REACTOME_RNA_POLYMERASE_I_TRANSCRIPTION_INITIATION	REACTOME_RNA_POLYMERASE_I_TRANSCRIPTION_INITIATION	0.59
GO:0015276	ligand-gated ion channel activity	0.59
GO:0022834	ligand-gated channel activity	0.59
ENSG00000172780	RAB43 PPI subnetwork	0.59
ENSG00000174547	MRPL11 PPI subnetwork	0.59
GO:0008652	cellular amino acid biosynthetic process	0.59
KEGG_PHENYLALANINE_METABOLISM	KEGG_PHENYLALANINE_METABOLISM	0.59
GO:0030863	cortical cytoskeleton	0.59
GO:0019842	vitamin binding	0.59
ENSG00000160867	FGFR4 PPI subnetwork	0.59
ENSG00000135597	REPS1 PPI subnetwork	0.59
MP:0003722	absent ureter	0.59
GO:0009411	response to UV	0.59
ENSG00000113240	CLK4 PPI subnetwork	0.59
ENSG00000168118	RAB4A PPI subnetwork	0.59
ENSG00000137462	TLR2 PPI subnetwork	0.59
ENSG00000134352	IL6ST PPI subnetwork	0.59
ENSG00000151532	VTI1A PPI subnetwork	0.59
GO:0048333	mesodermal cell differentiation	0.59
ENSG00000197956	S100A6 PPI subnetwork	0.59
GO:0032733	positive regulation of interleukin-10 production	0.59
ENSG00000125835	SNRPB PPI subnetwork	0.59
MP:0002572	abnormal emotion/affect behavior	0.59
ENSG00000198056	PRIM1 PPI subnetwork	0.59
ENSG00000151148	UBE3B PPI subnetwork	0.59
ENSG00000054118	THRAP3 PPI subnetwork	0.59
ENSG00000120438	TCP1 PPI subnetwork	0.59
MP:0002335	decreased airway responsiveness	0.59
ENSG00000141027	NCOR1 PPI subnetwork	0.59
ENSG00000072952	MRVI1 PPI subnetwork	0.59
REACTOME_PI3KAKT_ACTIVATION	REACTOME_PI3KAKT_ACTIVATION	0.59
ENSG00000109846	CRYAB PPI subnetwork	0.59
REACTOME_REPAIR_SYNTHESIS_OF_PATCH_27:30_BASES_LONG__BY_DNA_PO	REACTOME_REPAIR_SYNTHESIS_OF_PATCH_27:30_BASES_LONG__BY_DNA_POLYM	0.59
REACTOME_REPAIR_SYNTHESIS_FOR_GAP:FILLING_BY_DNA_POLYMERASE_IN_	REACTOME_REPAIR_SYNTHESIS_FOR_GAP:FILLING_BY_DNA_POLYMERASE_IN_TC:N	0.59
REACTOME_SHC1_EVENTS_IN_ERBB4_SIGNALING	REACTOME_SHC1_EVENTS_IN_ERBB4_SIGNALING	0.59
GO:0030326	embryonic limb morphogenesis	0.59
GO:0035113	embryonic appendage morphogenesis	0.59
MP:0002628	hepatic steatosis	0.59
GO:0004653	polypeptide N-acetylgalactosaminyltransferase activity	0.6
GO:0072331	signal transduction by p53 class mediator	0.6
MP:0006069	abnormal retinal neuronal layer morphology	0.6
MP:0008699	increased interleukin-4 secretion	0.6
GO:0019724	B cell mediated immunity	0.6
KEGG_NON_HOMOLOGOUS_END_JOINING	KEGG_NON_HOMOLOGOUS_END_JOINING	0.6
REACTOME_PHASE_II_CONJUGATION	REACTOME_PHASE_II_CONJUGATION	0.6
ENSG00000120063	GNA13 PPI subnetwork	0.6

Original gene set ID	Original gene set description	Nominal P value
KEGG_PENTOSE_PHOSPHATE_PATHWAY	KEGG_PENTOSE_PHOSPHATE_PATHWAY	0.6
ENSG00000177426	TGIF1 PPI subnetwork	0.6
ENSG00000015153	YAF2 PPI subnetwork	0.6
REACTOME_XENOBIOTICS	REACTOME_XENOBIOTICS	0.6
ENSG00000137673	MMP7 PPI subnetwork	0.6
REACTOME_CDK:MEDIATED_PHOSPHORYLATION_AND_REMOVAL_OF_CDC6	REACTOME_CDK:MEDIATED_PHOSPHORYLATION_AND_REMOVAL_OF_CDC6	0.6
GO:0007631	feeding behavior	0.6
ENSG00000134287	ARF3 PPI subnetwork	0.6
GO:0043297	apical junction assembly	0.6
ENSG00000206274	ENSG00000206274 PPI subnetwork	0.6
ENSG00000206383	HSPA1L PPI subnetwork	0.6
ENSG00000124299	PEPD PPI subnetwork	0.6
ENSG00000162191	UBXN1 PPI subnetwork	0.6
ENSG00000139180	NDUFA9 PPI subnetwork	0.6
ENSG00000183049	CAMK1D PPI subnetwork	0.6
ENSG00000139549	DHH PPI subnetwork	0.6
GO:0042579	microbody	0.6
GO:0005777	peroxisome	0.6
REACTOME_SIGNALING_BY_INSULIN_RECEPTOR	REACTOME_SIGNALING_BY_INSULIN_RECEPTOR	0.6
ENSG00000155657	TTN PPI subnetwork	0.6
REACTOME_UBIQUITIN:DEPENDENT_DEGRADATION_OF_CYCLIN_D	REACTOME_UBIQUITIN:DEPENDENT_DEGRADATION_OF_CYCLIN_D	0.6
REACTOME_UBIQUITIN:DEPENDENT_DEGRADATION_OF_CYCLIN_D1	REACTOME_UBIQUITIN:DEPENDENT_DEGRADATION_OF_CYCLIN_D1	0.6
ENSG00000134759	ELP2 PPI subnetwork	0.6
GO:0030031	cell projection assembly	0.6
ENSG00000100867	DHRS2 PPI subnetwork	0.6
GO:0030433	ER-associated protein catabolic process	0.6
MP:0001491	unresponsive to tactile stimuli	0.6
ENSG00000106245	BUD31 PPI subnetwork	0.6
KEGG_SELENOAMINO_ACID_METABOLISM	KEGG_SELENOAMINO_ACID_METABOLISM	0.6
GO:0043535	regulation of blood vessel endothelial cell migration	0.6
GO:0015300	solute:solute antiporter activity	0.6
GO:0046364	monosaccharide biosynthetic process	0.6
GO:0050907	detection of chemical stimulus involved in sensory perception	0.6
KEGG_PURINE_METABOLISM	KEGG_PURINE_METABOLISM	0.6
MP:0001417	decreased exploration in new environment	0.6
ENSG00000114698	PLSCR4 PPI subnetwork	0.6
ENSG00000153922	CHD1 PPI subnetwork	0.6
MP:0000188	abnormal circulating glucose level	0.6
GO:0001763	morphogenesis of a branching structure	0.6
REACTOME_NEPNS2_INTERACTS_WITH_THE_CELLULAR_EXPORT_MACHINERY	REACTOME_NEPNS2_INTERACTS_WITH_THE_CELLULAR_EXPORT_MACHINERY	0.6
ENSG00000091428	RAPGEF4 PPI subnetwork	0.6
ENSG00000087460	GNAS PPI subnetwork	0.6
GO:0045214	sarcomere organization	0.6
MP:0001431	abnormal eating behavior	0.6
GO:0071174	mitotic cell cycle spindle checkpoint	0.6
ENSG00000196235	SUPT5H PPI subnetwork	0.6
GO:0031669	cellular response to nutrient levels	0.6
ENSG00000101856	PGRMC1 PPI subnetwork	0.6
ENSG00000173692	PSMD1 PPI subnetwork	0.6

Original gene set ID	Original gene set description	Nominal P value
MP:0000097	short maxilla	0.6
MP:0002064	seizures	0.6
MP:0000807	abnormal hippocampus morphology	0.6
GO:0018149	peptide cross-linking	0.6
ENSG00000120616	EPC1 PPI subnetwork	0.6
GO:0006303	double-strand break repair via nonhomologous end joining	0.6
GO:0030855	epithelial cell differentiation	0.6
GO:0005249	voltage-gated potassium channel activity	0.6
ENSG00000135100	HNF1A PPI subnetwork	0.6
ENSG00000163435	ELF3 PPI subnetwork	0.6
MP:0000880	decreased Purkinje cell number	0.6
ENSG00000082074	FYB PPI subnetwork	0.6
MP:0005221	abnormal rostral-caudal axis patterning	0.6
KEGG_NON_SMALL_CELL_LUNG_CANCER	KEGG_NON_SMALL_CELL_LUNG_CANCER	0.6
ENSG00000108443	RPS6KB1 PPI subnetwork	0.6
REACTOME_SIGNAL_TRANSDUCTION_BY_L1	REACTOME_SIGNAL_TRANSDUCTION_BY_L1	0.6
ENSG00000150991	UBC PPI subnetwork	0.6
REACTOME_REGULATION_OF_DNA_REPLICATION	REACTOME_REGULATION_OF_DNA_REPLICATION	0.6
ENSG00000133710	SPINK5 PPI subnetwork	0.6
GO:0033059	cellular pigmentation	0.6
MP:0008539	decreased susceptibility to induced colitis	0.6
ENSG00000166407	LMO1 PPI subnetwork	0.6
GO:0047485	protein N-terminus binding	0.6
KEGG_AXON_GUIDANCE	KEGG_AXON_GUIDANCE	0.6
ENSG00000086758	HUWE1 PPI subnetwork	0.6
ENSG00000092470	WDR76 PPI subnetwork	0.6
GO:0060606	tube closure	0.6
ENSG00000137561	TTPA PPI subnetwork	0.6
GO:0005496	steroid binding	0.6
GO:0071230	cellular response to amino acid stimulus	0.6
GO:0033365	protein localization to organelle	0.6
GO:0005798	Golgi-associated vesicle	0.6
REACTOME_NOREPINEPHRINE_NEUROTRANSMITTER_RELEASE_CYCLE	REACTOME_NOREPINEPHRINE_NEUROTRANSMITTER_RELEASE_CYCLE	0.6
GO:0004601	peroxidase activity	0.6
GO:0016684	oxidoreductase activity, acting on peroxide as acceptor	0.6
GO:0042274	ribosomal small subunit biogenesis	0.6
ENSG00000154473	BUB3 PPI subnetwork	0.6
GO:2000045	regulation of G1/S transition of mitotic cell cycle	0.6
GO:0008064	regulation of actin polymerization or depolymerization	0.6
GO:0048588	developmental cell growth	0.6
MP:0005668	decreased circulating leptin level	0.6
ENSG00000086827	ZW10 PPI subnetwork	0.6
GO:0002009	morphogenesis of an epithelium	0.6
GO:0020037	heme binding	0.6
MP:0001475	reduced long term depression	0.6
GO:0006164	purine nucleotide biosynthetic process	0.6
ENSG00000065978	YBX1 PPI subnetwork	0.6
ENSG00000130816	DNMT1 PPI subnetwork	0.6
ENSG00000168078	PBK PPI subnetwork	0.6

Original gene set ID	Original gene set description	Nominal P value
ENSG00000171824	EXOSC10 PPI subnetwork	0.6
GO:0009312	oligosaccharide biosynthetic process	0.6
MP:0002398	abnormal bone marrow cell morphology/development	0.61
GO:0006325	chromatin organization	0.61
GO:0006361	transcription initiation from RNA polymerase I promoter	0.61
GO:0006112	energy reserve metabolic process	0.61
KEGG_BUTANOATE_METABOLISM	KEGG_BUTANOATE_METABOLISM	0.61
REACTOME_ION_CHANNEL_TRANSPORT	REACTOME_ION_CHANNEL_TRANSPORT	0.61
GO:0021915	neural tube development	0.61
ENSG00000213658	LAT PPI subnetwork	0.61
KEGG_CYSTEINE_AND_METHIONINE_METABOLISM	KEGG_CYSTEINE_AND_METHIONINE_METABOLISM	0.61
ENSG00000099783	HNRNPM PPI subnetwork	0.61
MP:0003690	abnormal glial cell physiology	0.61
ENSG00000196331	HIST1H2BO PPI subnetwork	0.61
GO:0007498	mesoderm development	0.61
ENSG00000172680	MOS PPI subnetwork	0.61
ENSG00000110367	DDX6 PPI subnetwork	0.61
ENSG00000173175	ADCY5 PPI subnetwork	0.61
MP:0003209	abnormal pulmonary elastic fiber morphology	0.61
MP:0011427	mesangial cell hyperplasia	0.61
GO:0005262	calcium channel activity	0.61
MP:0008332	decreased lactotroph cell number	0.61
MP:0010103	small thoracic cage	0.61
ENSG00000095319	NUP188 PPI subnetwork	0.61
GO:0051289	protein homotetramerization	0.61
ENSG00000198722	UNC13B PPI subnetwork	0.61
GO:0048048	embryonic eye morphogenesis	0.61
ENSG00000113368	LMNB1 PPI subnetwork	0.61
MP:0008173	increased follicular B cell number	0.61
ENSG00000100346	CACNA1I PPI subnetwork	0.61
ENSG00000171311	EXOSC1 PPI subnetwork	0.61
ENSG00000095139	ARCN1 PPI subnetwork	0.61
ENSG00000101367	MAPRE1 PPI subnetwork	0.61
GO:0022600	digestive system process	0.61
ENSG00000008018	PSMB1 PPI subnetwork	0.61
GO:0032816	positive regulation of natural killer cell activation	0.61
ENSG00000156076	WIF1 PPI subnetwork	0.61
GO:0001959	regulation of cytokine-mediated signaling pathway	0.61
REACTOME_ADP_SIGNALLING_THROUGH_P2Y_PURINOCEPTOR_1	REACTOME_ADP_SIGNALLING_THROUGH_P2Y_PURINOCEPTOR_1	0.61
GO:0050434	positive regulation of viral transcription	0.61
ENSG00000100926	TM9SF1 PPI subnetwork	0.61
ENSG00000006634	DBF4 PPI subnetwork	0.61
GO:0042102	positive regulation of T cell proliferation	0.61
ENSG00000089048	ESF1 PPI subnetwork	0.61
ENSG00000110244	APOA4 PPI subnetwork	0.61
ENSG00000128908	INO80 PPI subnetwork	0.61
ENSG00000182872	RBM10 PPI subnetwork	0.61
MP:0009643	abnormal urine homeostasis	0.61
ENSG00000154342	WNT3A PPI subnetwork	0.61

Original gene set ID	Original gene set description	Nominal P value
ENSG00000163002	NUP35 PPI subnetwork	0.61
ENSG00000187266	EPOR PPI subnetwork	0.61
REACTOME_HOMOLOGOUS_RECOMBINATION_REPAIR	REACTOME_HOMOLOGOUS_RECOMBINATION_REPAIR	0.61
REACTOME_HOMOLOGOUS_RECOMBINATION_REPAIR_OF_REPLICATION:INDEI	REACTOME_HOMOLOGOUS_RECOMBINATION_REPAIR_OF_REPLICATION:INDEPENI	0.61
GO:0019200	carbohydrate kinase activity	0.61
ENSG00000085276	MECOM PPI subnetwork	0.61
ENSG00000187239	FNBP1 PPI subnetwork	0.61
GO:0006352	transcription initiation, DNA-dependent	0.61
ENSG00000109917	ZNF259 PPI subnetwork	0.61
MP:0002913	abnormal PNS synaptic transmission	0.61
ENSG00000113594	LIFR PPI subnetwork	0.61
MP:0000781	decreased corpus callosum size	0.61
GO:0006833	water transport	0.61
ENSG00000100285	NEFH PPI subnetwork	0.61
GO:0003746	translation elongation factor activity	0.61
ENSG00000125482	TTF1 PPI subnetwork	0.61
MP:0004799	increased susceptibility to experimental autoimmune encephalomyelitis	0.61
ENSG00000148468	FAM171A1 PPI subnetwork	0.61
ENSG00000112992	NNT PPI subnetwork	0.61
REACTOME_OLFACTORY_SIGNALING_PATHWAY	REACTOME_OLFACTORY_SIGNALING_PATHWAY	0.61
GO:0006821	chloride transport	0.61
MP:0003703	abnormal vestibulocochlear ganglion morphology	0.61
GO:0045076	regulation of interleukin-2 biosynthetic process	0.61
GO:0009069	serine family amino acid metabolic process	0.61
REACTOME_ORC1_REMOVAL_FROM_CHROMATIN	REACTOME_ORC1_REMOVAL_FROM_CHROMATIN	0.61
REACTOME_SWITCHING_OF_ORIGINS_TO_A_POST:REPLICATIVE_STATE	REACTOME_SWITCHING_OF_ORIGINS_TO_A_POST:REPLICATIVE_STATE	0.61
ENSG00000164742	ADCY1 PPI subnetwork	0.61
ENSG00000178409	BEND3 PPI subnetwork	0.61
REACTOME_CELL_CYCLE	REACTOME_CELL_CYCLE	0.61
ENSG00000198900	TOP1 PPI subnetwork	0.61
GO:2000146	negative regulation of cell motility	0.61
GO:0090066	regulation of anatomical structure size	0.61
ENSG00000170315	UBB PPI subnetwork	0.61
GO:0001673	male germ cell nucleus	0.61
REACTOME_REGULATION_OF_APOPTOSIS	REACTOME_REGULATION_OF_APOPTOSIS	0.61
MP:0001407	short stride length	0.61
ENSG00000119392	GLE1 PPI subnetwork	0.61
ENSG00000016402	IL20RA PPI subnetwork	0.61
MP:0011186	abnormal visceral endoderm morphology	0.61
MP:0000890	thin cerebellar molecular layer	0.61
GO:0000930	gamma-tubulin complex	0.61
ENSG00000110324	IL10RA PPI subnetwork	0.61
MP:0001340	abnormal eyelid morphology	0.61
ENSG00000173020	ADRBK1 PPI subnetwork	0.61
REACTOME_GAB1_SIGNALOSOME	REACTOME_GAB1_SIGNALOSOME	0.61
REACTOME_CYCLIN_AB1_ASSOCIATED_EVENTS_DURING_G2M_TRANSITION	REACTOME_CYCLIN_AB1_ASSOCIATED_EVENTS_DURING_G2M_TRANSITION	0.61
GO:0048008	platelet-derived growth factor receptor signaling pathway	0.61
ENSG00000167088	SNRPD1 PPI subnetwork	0.61
GO:0055013	cardiac muscle cell development	0.61

Original gene set ID	Original gene set description	Nominal P value
GO:0055006	cardiac cell development	0.61
ENSG00000133477	FAM83F PPI subnetwork	0.61
ENSG00000143119	CD53 PPI subnetwork	0.62
GO:0001840	neural plate development	0.62
ENSG00000115935	WIPF1 PPI subnetwork	0.62
ENSG00000196411	EPHB4 PPI subnetwork	0.62
ENSG00000169891	REPS2 PPI subnetwork	0.62
ENSG00000111530	CAND1 PPI subnetwork	0.62
ENSG00000142867	BCL10 PPI subnetwork	0.62
GO:0015695	organic cation transport	0.62
GO:0032735	positive regulation of interleukin-12 production	0.62
ENSG00000175416	CLTB PPI subnetwork	0.62
GO:0007188	adenylate cyclase-modulating G-protein coupled receptor signaling pathway	0.62
ENSG00000177156	TALDO1 PPI subnetwork	0.62
ENSG00000102226	USP11 PPI subnetwork	0.62
ENSG00000144283	PKP4 PPI subnetwork	0.62
GO:0043968	histone H2A acetylation	0.62
MP:0009747	impaired behavioral response to xenobiotic	0.62
GO:0000307	cyclin-dependent protein kinase holoenzyme complex	0.62
GO:0030800	negative regulation of cyclic nucleotide metabolic process	0.62
GO:0030803	negative regulation of cyclic nucleotide biosynthetic process	0.62
GO:0030809	negative regulation of nucleotide biosynthetic process	0.62
GO:0046888	negative regulation of hormone secretion	0.62
MP:0000373	belly spot	0.62
ENSG00000212645	ENSG00000212645 PPI subnetwork	0.62
ENSG00000157388	CACNA1D PPI subnetwork	0.62
ENSG00000163510	CWC22 PPI subnetwork	0.62
ENSG00000206300	ENSG00000206300 PPI subnetwork	0.62
ENSG00000204435	CSNK2B PPI subnetwork	0.62
ENSG00000206406	CSNK2B PPI subnetwork	0.62
KEGG_ERBB_SIGNALING_PATHWAY	KEGG_ERBB_SIGNALING_PATHWAY	0.62
REACTOME_REGULATION_OF_MITOTIC_CELL_CYCLE	REACTOME_REGULATION_OF_MITOTIC_CELL_CYCLE	0.62
REACTOME_APCC:MEDIATED_DEGRADATION_OF_CELL_CYCLE_PROTEINS	REACTOME_APCC:MEDIATED_DEGRADATION_OF_CELL_CYCLE_PROTEINS	0.62
MP:0011423	kidney cortex atrophy	0.62
ENSG00000038427	VCAN PPI subnetwork	0.62
MP:0008700	decreased interleukin-4 secretion	0.62
ENSG00000084733	RAB10 PPI subnetwork	0.62
ENSG00000103942	HOMER2 PPI subnetwork	0.62
MP:0003953	abnormal hormone level	0.62
MP:0009289	decreased epididymal fat pad weight	0.62
MP:0000034	abnormal vestibule morphology	0.62
ENSG00000173110	HSPA6 PPI subnetwork	0.62
ENSG00000090615	GOLGA3 PPI subnetwork	0.62
MP:0001405	impaired coordination	0.62
GO:0045132	meiotic chromosome segregation	0.62
GO:0031344	regulation of cell projection organization	0.62
GO:0090207	regulation of triglyceride metabolic process	0.62
ENSG00000066654	THUMPD1 PPI subnetwork	0.62
ENSG00000055130	CUL1 PPI subnetwork	0.62

Original gene set ID	Original gene set description	Nominal P value
MP:0008705	increased interleukin-6 secretion	0.62
MP:0009434	paraparesis	0.62
ENSG00000076555	ACACB PPI subnetwork	0.62
KEGG_OLFACTORY_TRANSDUCTION	KEGG_OLFACTORY_TRANSDUCTION	0.62
ENSG00000112983	BRD8 PPI subnetwork	0.62
GO:0010883	regulation of lipid storage	0.62
ENSG00000175334	BANF1 PPI subnetwork	0.62
ENSG00000171560	FGA PPI subnetwork	0.62
MP:0002989	small kidney	0.62
ENSG00000138757	G3BP2 PPI subnetwork	0.62
MP:0008024	absent lymph nodes	0.62
MP:0002446	abnormal macrophage morphology	0.62
ENSG00000130725	UBE2M PPI subnetwork	0.62
REACTOME_NUCLEAR_IMPORT_OF_REV_PROTEIN	REACTOME_NUCLEAR_IMPORT_OF_REV_PROTEIN	0.62
MP:0001386	abnormal maternal nurturing	0.62
ENSG00000162594	IL23R PPI subnetwork	0.62
GO:0032272	negative regulation of protein polymerization	0.62
ENSG00000206281	TAPBP PPI subnetwork	0.62
ENSG00000112493	TAPBP PPI subnetwork	0.62
ENSG00000206208	TAPBP PPI subnetwork	0.62
ENSG00000186298	PPP1CC PPI subnetwork	0.62
MP:0000968	abnormal sensory neuron innervation pattern	0.62
REACTOME_AXON_GUIDANCE	REACTOME_AXON_GUIDANCE	0.62
GO:0048407	platelet-derived growth factor binding	0.62
REACTOME_PROLACTIN_RECEPTOR_SIGNALING	REACTOME_PROLACTIN_RECEPTOR_SIGNALING	0.62
MP:0004190	abnormal direction of embryo turning	0.62
ENSG00000023734	STRAP PPI subnetwork	0.62
GO:0031225	anchored to membrane	0.62
GO:0046906	tetrapyrrole binding	0.62
GO:0031272	regulation of pseudopodium assembly	0.62
MP:0001680	abnormal mesoderm development	0.62
MP:0000522	kidney cortex cysts	0.62
REACTOME_SPHINGOLIPID_DE_NOVO_BIOSYNTHESIS	REACTOME_SPHINGOLIPID_DE_NOVO_BIOSYNTHESIS	0.62
GO:0035567	non-canonical Wnt receptor signaling pathway	0.62
GO:0031513	nonmotile primary cilium	0.62
GO:0070603	SWI/SNF-type complex	0.62
ENSG00000140307	GTF2A2 PPI subnetwork	0.62
GO:0042462	eye photoreceptor cell development	0.62
REACTOME_POST_NMDA_RECEPTOR_ACTIVATION_EVENTS	REACTOME_POST_NMDA_RECEPTOR_ACTIVATION_EVENTS	0.62
GO:0000018	regulation of DNA recombination	0.62
GO:0072507	divalent inorganic cation homeostasis	0.62
MP:0000832	abnormal thalamus morphology	0.62
GO:0046782	regulation of viral transcription	0.62
MP:0008058	abnormal DNA repair	0.62
ENSG00000167414	GNG8 PPI subnetwork	0.62
ENSG00000092841	MYL6 PPI subnetwork	0.62
GO:0030425	dendrite	0.62
GO:0002063	chondrocyte development	0.62
GO:0048741	skeletal muscle fiber development	0.62

Original gene set ID	Original gene set description	Nominal P value
REACTOME_TRANSPORT_OF_GLUCOSE_AND_OTHER_SUGARS_BILE_SALTS_ANI	REACTOME_TRANSPORT_OF_GLUCOSE_AND_OTHER_SUGARS_BILE_SALTS_AND_O	0.62
MP:0008533	abnormal anterior visceral endoderm morphology	0.62
ENSG00000177542	SLC25A22 PPI subnetwork	0.62
MP:0003111	abnormal cell nucleus morphology	0.62
MP:0004672	short ribs	0.62
ENSG00000116830	TTF2 PPI subnetwork	0.62
MP:0005545	abnormal lens development	0.62
GO:0045622	regulation of T-helper cell differentiation	0.62
MP:0000031	abnormal cochlea morphology	0.62
GO:0032814	regulation of natural killer cell activation	0.62
MP:0004620	cervical vertebral fusion	0.62
GO:0044448	cell cortex part	0.62
GO:0030513	positive regulation of BMP signaling pathway	0.62
ENSG00000171453	POLR1C PPI subnetwork	0.62
REACTOME_RNA_POLYMERASE_III_TRANSCRIPTION_INITIATION_FROM_TYPE_3	REACTOME_RNA_POLYMERASE_III_TRANSCRIPTION_INITIATION_FROM_TYPE_3_PI	0.62
MP:0009814	increased prostaglandin level	0.62
GO:0000084	S phase of mitotic cell cycle	0.62
GO:0047555	3',5'-cyclic-GMP phosphodiesterase activity	0.62
REACTOME_FACTORS_INVOLVED_IN_MEGAKARYOCYTE_DEVELOPMENT_AND_	REACTOME_FACTORS_INVOLVED_IN_MEGAKARYOCYTE_DEVELOPMENT_AND_PLAT	0.62
GO:0002027	regulation of heart rate	0.62
MP:0000430	absent maxillary shelf	0.62
GO:0006809	nitric oxide biosynthetic process	0.62
ENSG00000144908	ALDH1L1 PPI subnetwork	0.62
GO:0035136	forelimb morphogenesis	0.62
ENSG00000159023	EPB41 PPI subnetwork	0.62
ENSG00000169062	UPF3A PPI subnetwork	0.62
REACTOME_AUTODEGRADATION_OF_THE_E3_UBIQUITIN_LIGASE_COP1	REACTOME_AUTODEGRADATION_OF_THE_E3_UBIQUITIN_LIGASE_COP1	0.63
ENSG00000083093	PALB2 PPI subnetwork	0.63
ENSG00000120500	ARR3 PPI subnetwork	0.63
ENSG00000134001	EIF2S1 PPI subnetwork	0.63
REACTOME_DESTABILIZATION_OF_MRNA_BY_AUF1_HNRNP_D0	REACTOME_DESTABILIZATION_OF_MRNA_BY_AUF1_HNRNP_D0	0.63
GO:0014032	neural crest cell development	0.63
GO:0016591	DNA-directed RNA polymerase II, holoenzyme	0.63
GO:0071346	cellular response to interferon-gamma	0.63
GO:0014033	neural crest cell differentiation	0.63
MP:0004814	reduced linear vestibular evoked potential	0.63
GO:0032722	positive regulation of chemokine production	0.63
ENSG00000139719	VPS33A PPI subnetwork	0.63
ENSG00000124334	IL9R PPI subnetwork	0.63
GO:0030330	DNA damage response, signal transduction by p53 class mediator	0.63
ENSG00000103152	MPG PPI subnetwork	0.63
ENSG00000126785	RHOJ PPI subnetwork	0.63
ENSG00000107263	RAPGEF1 PPI subnetwork	0.63
GO:0007091	mitotic metaphase/anaphase transition	0.63
GO:0003203	endocardial cushion morphogenesis	0.63
ENSG00000166900	STX3 PPI subnetwork	0.63
ENSG00000123562	MORF4L2 PPI subnetwork	0.63
MP:0001963	abnormal hearing physiology	0.63
MP:0004100	abnormal spinal cord interneuron morphology	0.63

Original gene set ID	Original gene set description	Nominal P value
ENSG00000158169	FANCC PPI subnetwork	0.63
MP:0004189	abnormal alveolar process morphology	0.63
KEGG_PENTOSE_AND_GLUCURONATE_INTERCONVERSIONS	KEGG_PENTOSE_AND_GLUCURONATE_INTERCONVERSIONS	0.63
ENSG00000005249	PRKAR2B PPI subnetwork	0.63
ENSG00000078369	GNB1 PPI subnetwork	0.63
ENSG00000088305	DNMT3B PPI subnetwork	0.63
ENSG00000176884	GRIN1 PPI subnetwork	0.63
GO:0006893	Golgi to plasma membrane transport	0.63
ENSG00000122966	CIT PPI subnetwork	0.63
ENSG00000137486	ARRB1 PPI subnetwork	0.63
REACTOME_REGULATION_OF_ACTIVATED_PAK:2P34_BY_PROTEASOME_MEDI/	REACTOME_REGULATION_OF_ACTIVATED_PAK:2P34_BY_PROTEASOME_MEDIATEL	0.63
GO:0031069	hair follicle morphogenesis	0.63
ENSG00000204390	HSPA1L PPI subnetwork	0.63
ENSG00000034713	GABARAPL2 PPI subnetwork	0.63
ENSG00000126351	THRA PPI subnetwork	0.63
GO:0051320	S phase	0.63
GO:0046849	bone remodeling	0.63
GO:0006376	mRNA splice site selection	0.63
GO:0016840	carbon-nitrogen lyase activity	0.63
ENSG00000106400	ZNHIT1 PPI subnetwork	0.63
ENSG00000167880	EVPL PPI subnetwork	0.63
GO:0015669	gas transport	0.63
GO:0044306	neuron projection terminus	0.63
GO:0060330	regulation of response to interferon-gamma	0.63
GO:0060334	regulation of interferon-gamma-mediated signaling pathway	0.63
GO:0070838	divalent metal ion transport	0.63
GO:0006302	double-strand break repair	0.63
ENSG00000113812	ACTR8 PPI subnetwork	0.63
REACTOME_REGULATION_OF_APCC_ACTIVATORS_BETWEEN_G1S_AND_EARLY	REACTOME_REGULATION_OF_APCC_ACTIVATORS_BETWEEN_G1S_AND_EARLY_AN	0.63
MP:0008965	increased basal metabolism	0.63
MP:0001663	abnormal digestive system physiology	0.63
GO:0001539	ciliary or flagellar motility	0.63
ENSG00000025293	PHF20 PPI subnetwork	0.63
ENSG00000067900	ROCK1 PPI subnetwork	0.63
REACTOME_APC:CDC20_MEDIATED_DEGRADATION_OF_NEK2A	REACTOME_APC:CDC20_MEDIATED_DEGRADATION_OF_NEK2A	0.63
ENSG00000142684	ZNF593 PPI subnetwork	0.63
GO:0030336	negative regulation of cell migration	0.63
GO:0001947	heart looping	0.63
GO:0061371	determination of heart left/right asymmetry	0.63
MP:0002906	increased susceptibility to pharmacologically induced seizures	0.63
GO:0070979	protein K11-linked ubiquitination	0.63
ENSG00000103043	VAC14 PPI subnetwork	0.63
ENSG00000132002	DNAJB1 PPI subnetwork	0.63
GO:0048814	regulation of dendrite morphogenesis	0.63
ENSG00000172020	GAP43 PPI subnetwork	0.63
GO:0016209	antioxidant activity	0.63
ENSG00000113194	FAF2 PPI subnetwork	0.63
GO:0090103	cochlea morphogenesis	0.63
GO:0030101	natural killer cell activation	0.63

Original gene set ID	Original gene set description	Nominal P value
REACTOME_P53:DEPENDENT_G1S_DNA_DAMAGE_CHECKPOINT	REACTOME_P53:DEPENDENT_G1S_DNA_DAMAGE_CHECKPOINT	0.63
REACTOME_P53:DEPENDENT_G1_DNA_DAMAGE_RESPONSE	REACTOME_P53:DEPENDENT_G1_DNA_DAMAGE_RESPONSE	0.63
REACTOME_PKB:MEDIATED_EVENTS	REACTOME_PKB:MEDIATED_EVENTS	0.63
ENSG00000077348	EXOSC5 PPI subnetwork	0.63
ENSG00000152270	PDE3B PPI subnetwork	0.63
GO:0034765	regulation of ion transmembrane transport	0.63
MP:0002494	increased IgM level	0.63
ENSG00000100504	PYGL PPI subnetwork	0.63
ENSG00000130787	HIP1R PPI subnetwork	0.63
REACTOME_SCF:BETA:TRCP_MEDIATED_DEGRADATION_OF_EMI1	REACTOME_SCF:BETA:TRCP_MEDIATED_DEGRADATION_OF_EMI1	0.63
ENSG00000110448	CD5 PPI subnetwork	0.63
GO:0043623	cellular protein complex assembly	0.63
GO:0030137	COPI-coated vesicle	0.63
ENSG00000143771	CNIH4 PPI subnetwork	0.63
ENSG00000136108	CKAP2 PPI subnetwork	0.63
ENSG00000196084	ENSG00000196084 PPI subnetwork	0.63
MP:0005269	abnormal occipital bone morphology	0.63
REACTOME_INTERLEUKIN:1_SIGNALING	REACTOME_INTERLEUKIN:1_SIGNALING	0.63
ENSG00000163918	RFC4 PPI subnetwork	0.63
ENSG00000087258	GNAO1 PPI subnetwork	0.63
GO:0034220	ion transmembrane transport	0.63
ENSG00000135945	REV1 PPI subnetwork	0.63
GO:0010950	positive regulation of endopeptidase activity	0.63
GO:0042267	natural killer cell mediated cytotoxicity	0.63
GO:0002228	natural killer cell mediated immunity	0.63
GO:0030125	clathrin vesicle coat	0.63
ENSG00000133706	LARS PPI subnetwork	0.63
ENSG00000157916	RER1 PPI subnetwork	0.63
GO:0048754	branching morphogenesis of a tube	0.63
ENSG00000167085	PHB PPI subnetwork	0.63
MP:0000885	ectopic Purkinje cell	0.63
ENSG00000056558	TRAF1 PPI subnetwork	0.63
GO:0016861	intramolecular oxidoreductase activity, interconverting aldoses and ketoses	0.63
ENSG00000166851	PLK1 PPI subnetwork	0.64
GO:0042734	presynaptic membrane	0.64
ENSG00000169139	UBE2V2 PPI subnetwork	0.64
GO:0006575	cellular modified amino acid metabolic process	0.64
GO:0031970	organelle envelope lumen	0.64
GO:0072503	cellular divalent inorganic cation homeostasis	0.64
ENSG00000140694	PARN PPI subnetwork	0.64
ENSG00000196510	ANAPC7 PPI subnetwork	0.64
GO:0000049	tRNA binding	0.64
GO:0019751	polyol metabolic process	0.64
GO:0048871	multicellular organismal homeostasis	0.64
GO:0032660	regulation of interleukin-17 production	0.64
GO:0032620	interleukin-17 production	0.64
MP:0008027	abnormal spinal cord white matter morphology	0.64
REACTOME_REGULATION_OF_GLUCOKINASE_BY_GLUCOKINASE_REGULATORY_	REACTOME_REGULATION_OF_GLUCOKINASE_BY_GLUCOKINASE_REGULATORY_PR	0.64
GO:0002699	positive regulation of immune effector process	0.64

Original gene set ID	Original gene set description	Nominal P value
ENSG00000070501	POLB PPI subnetwork	0.64
ENSG00000129675	ARHGEF6 PPI subnetwork	0.64
MP:0009642	abnormal blood homeostasis	0.64
ENSG00000073756	PTGS2 PPI subnetwork	0.64
ENSG00000117650	NEK2 PPI subnetwork	0.64
ENSG00000131153	GINS2 PPI subnetwork	0.64
GO:0001727	lipid kinase activity	0.64
MP:0000159	abnormal xiphoid process morphology	0.64
ENSG00000135390	ATP5G2 PPI subnetwork	0.64
ENSG00000198626	RYR2 PPI subnetwork	0.64
GO:0015932	nucleobase-containing compound transmembrane transporter activity	0.64
GO:0071013	catalytic step 2 spliceosome	0.64
ENSG00000125166	GOT2 PPI subnetwork	0.64
GO:0045429	positive regulation of nitric oxide biosynthetic process	0.64
GO:0042472	inner ear morphogenesis	0.64
GO:0042491	auditory receptor cell differentiation	0.64
GO:0045995	regulation of embryonic development	0.64
GO:0051085	chaperone mediated protein folding requiring cofactor	0.64
GO:0007015	actin filament organization	0.64
ENSG00000139269	INHBE PPI subnetwork	0.64
ENSG00000181222	POLR2A PPI subnetwork	0.64
ENSG00000126583	PRKCG PPI subnetwork	0.64
ENSG00000185721	DRG1 PPI subnetwork	0.64
ENSG00000211456	SACM1L PPI subnetwork	0.64
ENSG00000168490	PHYHIP PPI subnetwork	0.64
GO:0050729	positive regulation of inflammatory response	0.64
GO:0030032	lamellipodium assembly	0.64
GO:0034702	ion channel complex	0.64
ENSG00000135624	CCT7 PPI subnetwork	0.64
GO:0001619	lysosphingolipid and lysophosphatidic acid receptor activity	0.64
GO:0004402	histone acetyltransferase activity	0.64
MP:0002423	abnormal mast cell physiology	0.64
ENSG00000089289	IGBP1 PPI subnetwork	0.64
ENSG00000182578	CSF1R PPI subnetwork	0.64
MP:0004395	increased cochlear inner hair cell number	0.64
MP:0005565	increased blood urea nitrogen level	0.64
GO:0045841	negative regulation of mitotic metaphase/anaphase transition	0.64
ENSG00000134597	RBMX2 PPI subnetwork	0.64
ENSG00000206466	GABBR1 PPI subnetwork	0.64
ENSG00000204681	GABBR1 PPI subnetwork	0.64
ENSG00000206511	GABBR1 PPI subnetwork	0.64
GO:0048592	eye morphogenesis	0.64
ENSG00000134470	IL15RA PPI subnetwork	0.64
ENSG00000125630	POLR1B PPI subnetwork	0.64
MP:0009750	impaired behavioral response to addictive substance	0.64
GO:0048384	retinoic acid receptor signaling pathway	0.64
GO:0043240	Fanconi anaemia nuclear complex	0.64
MP:0003451	absent olfactory bulb	0.64
ENSG00000133216	EPHB2 PPI subnetwork	0.64

Original gene set ID	Original gene set description	Nominal P value
GO:0016712	oxidoreductase activity, acting on paired donors, with incorporation or reduction of	0.64
ENSG00000214528	ENSG00000214528 PPI subnetwork	0.64
GO:0005758	mitochondrial intermembrane space	0.64
GO:0006900	membrane budding	0.64
GO:0010878	cholesterol storage	0.64
ENSG00000109103	UNC119 PPI subnetwork	0.64
GO:0019933	cAMP-mediated signaling	0.64
GO:0004889	acetylcholine-activated cation-selective channel activity	0.64
MP:0000371	diluted coat color	0.64
REACTOME_INTEGRATION_OF_ENERGY_METABOLISM	REACTOME_INTEGRATION_OF_ENERGY_METABOLISM	0.64
ENSG00000145623	OSMR PPI subnetwork	0.64
ENSG00000137070	IL11RA PPI subnetwork	0.64
GO:0016874	ligase activity	0.64
MP:0002102	abnormal ear morphology	0.64
GO:0090092	regulation of transmembrane receptor protein serine/threonine kinase signaling pat	0.64
KEGG_CHEMOKINE_SIGNALING_PATHWAY	KEGG_CHEMOKINE_SIGNALING_PATHWAY	0.64
GO:0042094	interleukin-2 biosynthetic process	0.64
GO:0009164	nucleoside catabolic process	0.64
GO:0021795	cerebral cortex cell migration	0.64
GO:0000780	condensed nuclear chromosome, centromeric region	0.64
GO:0045666	positive regulation of neuron differentiation	0.64
ENSG00000092853	CLSPN PPI subnetwork	0.64
ENSG00000129354	AP1M2 PPI subnetwork	0.64
ENSG00000141380	SS18 PPI subnetwork	0.64
GO:0002089	lens morphogenesis in camera-type eye	0.64
REACTOME_RNA_POLYMERASE_I_PROMOTER_ESCAPE	REACTOME_RNA_POLYMERASE_I_PROMOTER_ESCAPE	0.64
REACTOME_REGULATORY_RNA_PATHWAYS	REACTOME_REGULATORY_RNA_PATHWAYS	0.64
REACTOME_MICRORNA_MIRNA_BIOGENESIS	REACTOME_MICRORNA_MIRNA_BIOGENESIS	0.64
MP:0004765	decreased brainstem auditory evoked potential	0.64
REACTOME_FORMATION_OF_TUBULIN_FOLDING_INTERMEDIATES_BY_CCTTRI	REACTOME_FORMATION_OF_TUBULIN_FOLDING_INTERMEDIATES_BY_CCTTRIC	0.64
GO:0022890	inorganic cation transmembrane transporter activity	0.64
MP:0001297	microphthalmia	0.65
MP:0001183	overexpanded pulmonary alveoli	0.65
MP:0004046	abnormal mitosis	0.65
GO:0043204	perikaryon	0.65
ENSG00000143093	FAM40A PPI subnetwork	0.65
GO:0045667	regulation of osteoblast differentiation	0.65
GO:0001708	cell fate specification	0.65
GO:0007616	long-term memory	0.65
ENSG00000129465	RIPK3 PPI subnetwork	0.65
MP:0005558	decreased creatinine clearance	0.65
GO:0007501	mesodermal cell fate specification	0.65
ENSG00000007171	NOS2 PPI subnetwork	0.65
KEGG_VASCULAR_SMOOTH_MUSCLE_CONTRACTION	KEGG_VASCULAR_SMOOTH_MUSCLE_CONTRACTION	0.65
MP:0004324	vestibular hair cell degeneration	0.65
KEGG_ALLOGRAFT_REJECTION	KEGG_ALLOGRAFT_REJECTION	0.65
MP:0002023	B cell derived lymphoma	0.65
ENSG00000166592	RRAD PPI subnetwork	0.65
MP:0001783	decreased white adipose tissue amount	0.65

Original gene set ID	Original gene set description	Nominal P value
MP:0002746	abnormal semilunar valve morphology	0.65
GO:0048332	mesoderm morphogenesis	0.65
REACTOME_G_BETAGAMMA_SIGNALLING_THROUGH_PI3KGAMMA	REACTOME_G_BETAGAMMA_SIGNALLING_THROUGH_PI3KGAMMA	0.65
MP:0005551	abnormal eye electrophysiology	0.65
GO:0046459	short-chain fatty acid metabolic process	0.65
MP:0005407	hyperalgesia	0.65
MP:0004077	abnormal striatum morphology	0.65
ENSG00000002822	MAD1L1 PPI subnetwork	0.65
ENSG00000105486	LIG1 PPI subnetwork	0.65
ENSG00000125885	MCM8 PPI subnetwork	0.65
ENSG00000120659	TNFSF11 PPI subnetwork	0.65
ENSG00000115289	PCGF1 PPI subnetwork	0.65
ENSG00000164091	WDR82 PPI subnetwork	0.65
ENSG00000164077	MON1A PPI subnetwork	0.65
GO:0006944	cellular membrane fusion	0.65
ENSG00000073910	FRY PPI subnetwork	0.65
GO:0051223	regulation of protein transport	0.65
ENSG00000023445	BIRC3 PPI subnetwork	0.65
ENSG00000197958	RPL12 PPI subnetwork	0.65
GO:0008207	C21-steroid hormone metabolic process	0.65
REACTOME_APCCCDC20_MEDIATED_DEGRADATION_OF_CYCLIN_B	REACTOME_APCCCDC20_MEDIATED_DEGRADATION_OF_CYCLIN_B	0.65
ENSG00000163283	ALPP PPI subnetwork	0.65
GO:2001056	positive regulation of cysteine-type endopeptidase activity	0.65
GO:0043280	positive regulation of cysteine-type endopeptidase activity involved in apoptotic pro	0.65
MP:0008456	abnormal retinal rod cell outer segment morphology	0.65
KEGG_RNA_POLYMERASE	KEGG_RNA_POLYMERASE	0.65
GO:0001077	RNA polymerase II core promoter proximal region sequence-specific DNA binding tr	0.65
ENSG00000111907	TPD52L1 PPI subnetwork	0.65
ENSG00000175575	PAAF1 PPI subnetwork	0.65
ENSG00000100138	NHP2L1 PPI subnetwork	0.65
ENSG00000100395	L3MBTL2 PPI subnetwork	0.65
MP:0005619	increased urine potassium level	0.65
GO:0070661	leukocyte proliferation	0.65
ENSG00000176386	CDC26 PPI subnetwork	0.65
ENSG00000020426	MNAT1 PPI subnetwork	0.65
ENSG00000135829	DHX9 PPI subnetwork	0.65
ENSG00000181072	CHRM2 PPI subnetwork	0.65
ENSG00000104812	GYS1 PPI subnetwork	0.65
GO:0000779	condensed chromosome, centromeric region	0.65
ENSG00000135775	COG2 PPI subnetwork	0.65
GO:0030139	endocytic vesicle	0.65
ENSG00000120805	ARL1 PPI subnetwork	0.65
GO:0042100	B cell proliferation	0.65
MP:0001406	abnormal gait	0.65
MP:0001689	incomplete somite formation	0.65
MP:0004985	decreased osteoclast cell number	0.65
ENSG00000100462	PRMT5 PPI subnetwork	0.65
REACTOME_S_PHASE	REACTOME_S_PHASE	0.65
ENSG00000115884	SDC1 PPI subnetwork	0.65

Original gene set ID	Original gene set description	Nominal P value
ENSG00000182492	BGN PPI subnetwork	0.65
GO:0019843	rRNA binding	0.65
GO:0000301	retrograde transport, vesicle recycling within Golg	0.65
GO:0060395	SMAD protein signal transduction	0.65
GO:0071804	cellular potassium ion transport	0.65
GO:0071805	potassium ion transmembrane transport	0.65
GO:0048562	embryonic organ morphogenesis	0.65
GO:0048661	positive regulation of smooth muscle cell proliferatior	0.65
MP:0002971	abnormal brown adipose tissue morphology	0.65
GO:0061025	membrane fusion	0.65
ENSG00000119772	DNMT3A PPI subnetwork	0.65
ENSG00000130255	RPL36 PPI subnetwork	0.65
REACTOME_REMOVAL_OF_LICENSING_FACTORS_FROM_ORIGINS	REACTOME_REMOVAL_OF_LICENSING_FACTORS_FROM_ORIGINS	0.65
MP:0002810	microcytic anemia	0.65
GO:0019866	organelle inner membrane	0.65
ENSG00000092010	PSME1 PPI subnetwork	0.65
GO:0045786	negative regulation of cell cycle	0.65
ENSG00000008952	SEC62 PPI subnetwork	0.65
ENSG00000185436	IL28RA PPI subnetwork	0.65
GO:0043028	cysteine-type endopeptidase regulator activity involved in apoptotic proces	0.65
GO:0004114	3',5'-cyclic-nucleotide phosphodiesterase activity	0.65
GO:0008083	growth factor activity	0.65
ENSG00000116678	LEPR PPI subnetwork	0.65
ENSG00000100813	ACIN1 PPI subnetwork	0.65
GO:0015026	coreceptor activity	0.65
KEGG_ARACHIDONIC_ACID_METABOLISM	KEGG_ARACHIDONIC_ACID_METABOLISM	0.65
MP:0003503	decreased activity of thyroid	0.65
ENSG00000110321	EIF4G2 PPI subnetwork	0.65
ENSG00000133818	RRAS2 PPI subnetwork	0.65
ENSG00000143995	MEIS1 PPI subnetwork	0.65
MP:0008497	decreased IgG2b level	0.65
GO:0000315	organellar large ribosomal subunit	0.65
GO:0005762	mitochondrial large ribosomal subunit	0.65
GO:0060389	pathway-restricted SMAD protein phosphorylatior	0.65
GO:0002011	morphogenesis of an epithelial sheet	0.65
ENSG00000169282	KCNAB1 PPI subnetwork	0.65
GO:0034341	response to interferon-gamma	0.65
ENSG00000132467	UTP3 PPI subnetwork	0.65
ENSG00000100941	PNN PPI subnetwork	0.65
GO:0048593	camera-type eye morphogenesis	0.65
ENSG00000174371	EXO1 PPI subnetwork	0.65
GO:0010885	regulation of cholesterol storage	0.65
ENSG00000093217	XYLB PPI subnetwork	0.65
ENSG00000163904	SENP2 PPI subnetwork	0.65
ENSG00000100568	VTI1B PPI subnetwork	0.65
GO:0009267	cellular response to starvation	0.65
GO:0035587	purinergic receptor signaling pathway	0.65
MP:0008587	short photoreceptor outer segment	0.65
ENSG00000171549	ENSG00000171549 PPI subnetwork	0.65

Original gene set ID	Original gene set description	Nominal P value
ENSG00000182520	ENSG00000182520 PPI subnetwork	0.65
MP:0000967	abnormal sensory neuron projections	0.66
GO:0051186	cofactor metabolic process	0.66
ENSG00000058668	ATP2B4 PPI subnetwork	0.66
ENSG00000161547	SRSF2 PPI subnetwork	0.66
MP:0004784	abnormal anterior cardinal vein morphology	0.66
ENSG00000096401	CDC5L PPI subnetwork	0.66
GO:0042026	protein refolding	0.66
ENSG00000163191	S100A11 PPI subnetwork	0.66
ENSG00000159113	ENSG00000159113 PPI subnetwork	0.66
REACTOME_APCCCDH1_MEDIATED_DEGRADATION_OF_CDC20_AND_OTHER_A	REACTOME_APCCCDH1_MEDIATED_DEGRADATION_OF_CDC20_AND_OTHER_APCC	0.66
ENSG00000139343	SNRPF PPI subnetwork	0.66
GO:0090102	cochlea development	0.66
GO:0008565	protein transporter activity	0.66
GO:0001664	G-protein coupled receptor binding	0.66
ENSG00000183691	NOG PPI subnetwork	0.66
GO:0019005	SCF ubiquitin ligase complex	0.66
GO:0042474	middle ear morphogenesis	0.66
ENSG00000004700	RECQL PPI subnetwork	0.66
REACTOME_PYRIMIDINE_METABOLISM	REACTOME_PYRIMIDINE_METABOLISM	0.66
MP:0003084	abnormal skeletal muscle fiber morphology	0.66
GO:0009952	anterior/posterior pattern specification	0.66
GO:0016072	rRNA metabolic process	0.66
GO:0031331	positive regulation of cellular catabolic process	0.66
ENSG00000127314	RAP1B PPI subnetwork	0.66
GO:0048839	inner ear development	0.66
GO:0050767	regulation of neurogenesis	0.66
ENSG00000125730	C3 PPI subnetwork	0.66
GO:0010035	response to inorganic substance	0.66
GO:0009615	response to virus	0.66
ENSG00000108883	EFTUD2 PPI subnetwork	0.66
GO:0018345	protein palmitoylation	0.66
MP:0002664	decreased circulating adrenocorticotropin leve	0.66
ENSG00000112186	CAP2 PPI subnetwork	0.66
ENSG00000159459	UBR1 PPI subnetwork	0.66
ENSG00000131652	THOC6 PPI subnetwork	0.66
ENSG00000065548	ZC3H15 PPI subnetwork	0.66
GO:0009798	axis specification	0.66
ENSG00000027697	IFNGR1 PPI subnetwork	0.66
GO:0009314	response to radiation	0.66
GO:0000777	condensed chromosome kinetochore	0.66
MP:0002835	abnormal cranial suture morphology	0.66
MP:0005205	abnormal eye anterior chamber morphology	0.66
ENSG00000179950	PUF60 PPI subnetwork	0.66
GO:0071779	G1/S transition checkpoint	0.66
GO:0043087	regulation of GTPase activity	0.66
GO:0072372	primary cilium	0.66
GO:0001841	neural tube formation	0.66
ENSG00000169306	IL1RAPL1 PPI subnetwork	0.66

Original gene set ID	Original gene set description	Nominal P value
GO:0031072	heat shock protein binding	0.66
MP:0009456	impaired cued conditioning behavior	0.66
REACTOME_CRMPS_IN_SEMA3A_SIGNALING	REACTOME_CRMPS_IN_SEMA3A_SIGNALING	0.66
MP:0000154	rib fusion	0.66
GO:0007389	pattern specification process	0.66
MP:0008500	increased IgG2a level	0.66
ENSG00000165731	RET PPI subnetwork	0.66
GO:0010952	positive regulation of peptidase activity	0.66
GO:0021515	cell differentiation in spinal cord	0.66
GO:0002440	production of molecular mediator of immune response	0.66
ENSG00000136631	VPS45 PPI subnetwork	0.66
GO:0050873	brown fat cell differentiation	0.66
ENSG00000138190	EXOC6 PPI subnetwork	0.66
ENSG00000173744	AGFG1 PPI subnetwork	0.66
GO:0043073	germ cell nucleus	0.66
GO:0042552	myelination	0.66
ENSG00000069345	DNAJA2 PPI subnetwork	0.66
REACTOME_G_ALPHA_S_SIGNALLING_EVENTS	REACTOME_G_ALPHA_S_SIGNALLING_EVENTS	0.66
ENSG00000003436	TFPI PPI subnetwork	0.66
GO:0042044	fluid transport	0.66
ENSG00000138430	OLA1 PPI subnetwork	0.66
MP:0000194	hypercalcemia	0.66
GO:0000062	fatty-acyl-CoA binding	0.66
ENSG00000205220	PSMB10 PPI subnetwork	0.66
GO:0000038	very long-chain fatty acid metabolic process	0.66
GO:0018208	peptidyl-proline modification	0.66
GO:0032981	mitochondrial respiratory chain complex I assembly	0.66
GO:0097031	mitochondrial respiratory chain complex I biogenesis	0.66
GO:0010257	NADH dehydrogenase complex assembly	0.66
ENSG00000115561	CHMP3 PPI subnetwork	0.66
MP:0004022	abnormal cone electrophysiology	0.66
ENSG00000092199	HNRNPC PPI subnetwork	0.66
ENSG00000106070	GRB10 PPI subnetwork	0.66
GO:0007093	mitotic cell cycle checkpoint	0.66
ENSG00000072958	AP1M1 PPI subnetwork	0.66
ENSG00000142677	IL22RA1 PPI subnetwork	0.66
ENSG00000132361	KIAA0664 PPI subnetwork	0.66
MP:0011501	increased glomerular capsule space	0.66
ENSG00000103342	GSPT1 PPI subnetwork	0.66
GO:0030983	mismatched DNA binding	0.66
REACTOME_CELL:CELL_JUNCTION_ORGANIZATION	REACTOME_CELL:CELL_JUNCTION_ORGANIZATION	0.66
GO:0010171	body morphogenesis	0.66
MP:0006358	absent pinna reflex	0.66
ENSG00000088247	KHSRP PPI subnetwork	0.66
ENSG00000134871	COL4A2 PPI subnetwork	0.66
MP:0001463	abnormal spatial learning	0.66
GO:0031432	titin binding	0.66
GO:0008021	synaptic vesicle	0.66
GO:0015464	acetylcholine receptor activity	0.66

Original gene set ID	Original gene set description	Nominal P value
MP:0002703	abnormal renal tubule morphology	0.66
GO:0002062	chondrocyte differentiation	0.67
ENSG00000100644	HIF1A PPI subnetwork	0.67
GO:0051258	protein polymerization	0.67
ENSG00000114745	GORASP1 PPI subnetwork	0.67
ENSG00000144381	HSPD1 PPI subnetwork	0.67
GO:0009124	nucleoside monophosphate biosynthetic process	0.67
ENSG00000159128	IFNGR2 PPI subnetwork	0.67
GO:0000216	M/G1 transition of mitotic cell cycle	0.67
ENSG00000154277	UCHL1 PPI subnetwork	0.67
ENSG00000170624	SGCD PPI subnetwork	0.67
GO:0033151	V(D)J recombination	0.67
GO:0016254	preassembly of GPI anchor in ER membrane	0.67
ENSG00000151693	ASAP2 PPI subnetwork	0.67
GO:0030216	keratinocyte differentiation	0.67
MP:0002823	abnormal rib development	0.67
ENSG00000095261	PSMD5 PPI subnetwork	0.67
GO:0030041	actin filament polymerization	0.67
GO:0021903	rostrocaudal neural tube patterning	0.67
ENSG00000070831	CDC42 PPI subnetwork	0.67
ENSG00000165912	PACSIN3 PPI subnetwork	0.67
ENSG00000187741	FANCA PPI subnetwork	0.67
GO:0006766	vitamin metabolic process	0.67
GO:0070167	regulation of biomineral tissue development	0.67
GO:0051597	response to methylmercury	0.67
KEGG_TASTE_TRANSDUCTION	KEGG_TASTE_TRANSDUCTION	0.67
ENSG00000175582	RAB6A PPI subnetwork	0.67
KEGG_TYROSINE_METABOLISM	KEGG_TYROSINE_METABOLISM	0.67
GO:0048736	appendage development	0.67
GO:0060173	limb development	0.67
ENSG00000100380	ST13 PPI subnetwork	0.67
GO:0030663	COPI coated vesicle membrane	0.67
REACTOME_CDC20PHOSPHO:APCC_MEDIATED_DEGRADATION_OF_CYCLIN_A	REACTOME_CDC20PHOSPHO:APCC_MEDIATED_DEGRADATION_OF_CYCLIN_A	0.67
GO:0055074	calcium ion homeostasis	0.67
GO:0042471	ear morphogenesis	0.67
GO:0007067	mitosis	0.67
GO:0000280	nuclear division	0.67
ENSG00000186051	TAL2 PPI subnetwork	0.67
GO:0009066	aspartate family amino acid metabolic process	0.67
GO:0009950	dorsal/ventral axis specification	0.67
ENSG00000074800	ENO1 PPI subnetwork	0.67
GO:0016811	hydrolase activity, acting on carbon-nitrogen (but not peptide) bonds, in linear amid	0.67
MP:0000733	abnormal muscle development	0.67
GO:0007595	lactation	0.67
KEGG_AMINO_SUGAR_AND_NUCLEOTIDE_SUGAR_METABOLISM	KEGG_AMINO_SUGAR_AND_NUCLEOTIDE_SUGAR_METABOLISM	0.67
ENSG00000074211	PPP2R2C PPI subnetwork	0.67
ENSG00000147889	CDKN2A PPI subnetwork	0.67
MP:0001944	abnormal pancreas morphology	0.67
ENSG00000147684	NDUFB9 PPI subnetwork	0.67

Original gene set ID	Original gene set description	Nominal P value
ENSG00000068654	POLR1A PPI subnetwork	0.67
REACTOME_METABOLISM_OF_POLYAMINES	REACTOME_METABOLISM_OF_POLYAMINES	0.67
GO:0000087	M phase of mitotic cell cycle	0.67
GO:0007158	neuron cell-cell adhesion	0.67
GO:0032452	histone demethylase activity	0.67
GO:0046519	sphingoid metabolic process	0.67
MP:0002591	decreased mean corpuscular volume	0.67
ENSG00000120149	MSX2 PPI subnetwork	0.67
MP:0004772	abnormal bile secretion	0.67
GO:0032844	regulation of homeostatic process	0.67
MP:0002916	increased synaptic depression	0.67
MP:0004321	short sternum	0.67
ENSG00000172137	CALB2 PPI subnetwork	0.67
ENSG00000101400	SNTA1 PPI subnetwork	0.67
GO:0016597	amino acid binding	0.67
ENSG00000069248	NUP133 PPI subnetwork	0.67
REACTOME_G:PROTEIN_BETAGAMMA_SIGNALLING	REACTOME_G:PROTEIN_BETAGAMMA_SIGNALLING	0.67
GO:0005778	peroxisomal membrane	0.67
GO:0031903	microbody membrane	0.67
GO:0004950	chemokine receptor activity	0.67
GO:0001637	G-protein coupled chemoattractant receptor activity	0.67
ENSG00000137936	BCAR3 PPI subnetwork	0.67
GO:0051890	regulation of cardioblast differentiation	0.67
ENSG00000057608	GDI2 PPI subnetwork	0.67
GO:0035326	enhancer binding	0.67
GO:0060627	regulation of vesicle-mediated transport	0.67
MP:0003604	single kidney	0.67
REACTOME_SIGNALING_BY_EGFR_IN_CANCER	REACTOME_SIGNALING_BY_EGFR_IN_CANCER	0.67
GO:0001659	temperature homeostasis	0.67
ENSG00000076924	XAB2 PPI subnetwork	0.67
MP:0005344	increased circulating bilirubin level	0.67
ENSG00000082458	DLG3 PPI subnetwork	0.67
ENSG00000169016	E2F6 PPI subnetwork	0.67
GO:0005164	tumor necrosis factor receptor binding	0.67
GO:0000407	pre-autophagosomal structure	0.67
MP:0001176	abnormal lung development	0.67
GO:0000041	transition metal ion transport	0.67
GO:0007194	negative regulation of adenylate cyclase activity	0.67
GO:0031280	negative regulation of cyclase activity	0.67
ENSG00000055163	CYFIP2 PPI subnetwork	0.67
GO:0001707	mesoderm formation	0.67
GO:0030832	regulation of actin filament length	0.67
ENSG00000114942	EEF1B2 PPI subnetwork	0.67
ENSG00000094914	AAAS PPI subnetwork	0.67
GO:0002717	positive regulation of natural killer cell mediated immunity	0.67
GO:0045954	positive regulation of natural killer cell mediated cytotoxicity	0.67
ENSG00000123496	IL13RA2 PPI subnetwork	0.67
ENSG00000164485	IL22RA2 PPI subnetwork	0.67
ENSG00000103522	IL21R PPI subnetwork	0.67

Original gene set ID	Original gene set description	Nominal P value
ENSG00000076944	STXBP2 PPI subnetwork	0.67
MP:0008392	decreased primordial germ cell number	0.67
MP:0003921	abnormal heart left ventricle morphology	0.67
MP:0003446	renal hypoplasia	0.67
ENSG00000136603	SKIL PPI subnetwork	0.67
ENSG00000147162	OGT PPI subnetwork	0.67
GO:0048286	lung alveolus development	0.67
GO:0033003	regulation of mast cell activation	0.67
GO:0055007	cardiac muscle cell differentiation	0.67
REACTOME_STABILIZATION_OF_P53	REACTOME_STABILIZATION_OF_P53	0.67
MP:0003797	abnormal compact bone morphology	0.67
ENSG00000105204	DYRK1B PPI subnetwork	0.67
GO:0071214	cellular response to abiotic stimulus	0.67
REACTOME_NITRIC_OXIDE_STIMULATES_GUANYLATE_CYCLASE	REACTOME_NITRIC_OXIDE_STIMULATES_GUANYLATE_CYCLASE	0.67
REACTOME_POSTSYNAPTIC_NICOTINIC_ACETYLCHOLINE_RECEPTORS	REACTOME_POSTSYNAPTIC_NICOTINIC_ACETYLCHOLINE_RECEPTORS	0.67
REACTOME_ACETYLCHOLINE_BINDING_AND_DOWNSTREAM_EVENTS	REACTOME_ACETYLCHOLINE_BINDING_AND_DOWNSTREAM_EVENTS	0.67
REACTOME_ACTIVATION_OF_NICOTINIC_ACETYLCHOLINE_RECEPTORS	REACTOME_ACTIVATION_OF_NICOTINIC_ACETYLCHOLINE_RECEPTORS	0.67
GO:0003170	heart valve development	0.67
GO:0010817	regulation of hormone levels	0.67
REACTOME_REGULATION_OF_MRNA_STABILITY_BY_PROTEINS_THAT_BIND_AU	REACTOME_REGULATION_OF_MRNA_STABILITY_BY_PROTEINS_THAT_BIND_AU:RI	0.67
ENSG00000104325	DECR1 PPI subnetwork	0.67
GO:0033124	regulation of GTP catabolic process	0.67
GO:0051350	negative regulation of lyase activity	0.67
KEGG_RENIN_ANGIOTENSIN_SYSTEM	KEGG_RENIN_ANGIOTENSIN_SYSTEM	0.67
GO:0010038	response to metal ion	0.67
ENSG00000163631	ALB PPI subnetwork	0.67
ENSG00000104897	SF3A2 PPI subnetwork	0.67
ENSG00000163806	SPDYA PPI subnetwork	0.67
MP:0004404	cochlear outer hair cell degeneration	0.67
REACTOME_PLATELET_ADHESION_TO_EXPOSED_COLLAGEN	REACTOME_PLATELET_ADHESION_TO_EXPOSED_COLLAGEN	0.67
GO:0030530	heterogeneous nuclear ribonucleoprotein complex	0.67
ENSG00000066117	SMARCD1 PPI subnetwork	0.67
GO:0008094	DNA-dependent ATPase activity	0.67
GO:0006013	mannose metabolic process	0.67
GO:0043200	response to amino acid stimulus	0.67
MP:0005553	increased circulating creatinine level	0.67
ENSG00000011052	NME2 PPI subnetwork	0.67
ENSG00000179051	RCC2 PPI subnetwork	0.68
MP:0001077	abnormal spinal nerve morphology	0.68
GO:0035107	appendage morphogenesis	0.68
GO:0035108	limb morphogenesis	0.68
GO:0005942	phosphatidylinositol 3-kinase complex	0.68
GO:0072384	organelle transport along microtubule	0.68
GO:0006520	cellular amino acid metabolic process	0.68
GO:0090068	positive regulation of cell cycle process	0.68
MP:0008498	decreased IgG3 level	0.68
ENSG00000182979	MTA1 PPI subnetwork	0.68
GO:0048285	organelle fission	0.68
ENSG00000101161	PRPF6 PPI subnetwork	0.68

Original gene set ID	Original gene set description	Nominal P value
REACTOME_PLATELET_HOMEOSTASIS	REACTOME_PLATELET_HOMEOSTASIS	0.68
MP:0000752	dystrophic muscle	0.68
GO:0045165	cell fate commitment	0.68
ENSG00000184486	POU3F2 PPI subnetwork	0.68
GO:0008514	organic anion transmembrane transporter activity	0.68
GO:0015672	monovalent inorganic cation transport	0.68
GO:0045766	positive regulation of angiogenesis	0.68
MP:0006359	absent startle reflex	0.68
ENSG00000150672	DLG2 PPI subnetwork	0.68
ENSG00000137076	TLN1 PPI subnetwork	0.68
ENSG00000153147	SMARCA5 PPI subnetwork	0.68
GO:0015804	neutral amino acid transport	0.68
ENSG00000173867	ENSG00000173867 PPI subnetwork	0.68
MP:0003059	decreased insulin secretion	0.68
GO:0007006	mitochondrial membrane organization	0.68
GO:0002763	positive regulation of myeloid leukocyte differentiation	0.68
ENSG00000072849	DERL2 PPI subnetwork	0.68
ENSG00000179036	ENSG00000179036 PPI subnetwork	0.68
GO:0016830	carbon-carbon lyase activity	0.68
ENSG00000053900	ANAPC4 PPI subnetwork	0.68
GO:0072522	purine-containing compound biosynthetic process	0.68
ENSG00000101199	ARFGAP1 PPI subnetwork	0.68
ENSG00000171533	MAP6 PPI subnetwork	0.68
MP:0009766	increased sensitivity to xenobiotic induced morbidity/mortality	0.68
ENSG00000136045	PWP1 PPI subnetwork	0.68
GO:0055001	muscle cell development	0.68
ENSG00000143761	ARF1 PPI subnetwork	0.68
GO:0017144	drug metabolic process	0.68
MP:0000043	organ of Corti degeneration	0.68
REACTOME_APCCCDC20_MEDIATED_DEGRADATION_OF_SECURIN	REACTOME_APCCCDC20_MEDIATED_DEGRADATION_OF_SECURIN	0.68
ENSG00000197822	OCLN PPI subnetwork	0.68
ENSG00000134852	CLOCK PPI subnetwork	0.68
GO:0001573	ganglioside metabolic process	0.68
ENSG00000039537	C6 PPI subnetwork	0.68
ENSG00000213585	VDAC1 PPI subnetwork	0.68
ENSG00000025770	NCAPH2 PPI subnetwork	0.68
ENSG00000146007	ZMAT2 PPI subnetwork	0.68
GO:0005741	mitochondrial outer membrane	0.68
GO:0006874	cellular calcium ion homeostasis	0.68
MP:0008410	increased cellular sensitivity to ultraviolet irradiation	0.68
MP:0002642	anisocytosis	0.68
GO:0021543	pallium development	0.68
REACTOME_EARLY_PHASE_OF_HIV_LIFE_CYCLE	REACTOME_EARLY_PHASE_OF_HIV_LIFE_CYCLE	0.68
REACTOME_CYCLIN_E_ASSOCIATED_EVENTS_DURING_G1S_TRANSITION	REACTOME_CYCLIN_E_ASSOCIATED_EVENTS_DURING_G1S_TRANSITION	0.68
GO:0019320	hexose catabolic process	0.68
REACTOME_LAGGING_STRAND_SYNTHESIS	REACTOME_LAGGING_STRAND_SYNTHESIS	0.68
REACTOME_DESTABILIZATION_OF_MRNA_BY_TRISTETRAPROLIN_TTP	REACTOME_DESTABILIZATION_OF_MRNA_BY_TRISTETRAPROLIN_TTP	0.68
ENSG00000167815	PRDX2 PPI subnetwork	0.68
MP:0008535	enlarged lateral ventricles	0.68

Original gene set ID	Original gene set description	Nominal P value
GO:0043648	dicarboxylic acid metabolic process	0.68
ENSG00000140795	MYLK3 PPI subnetwork	0.68
GO:0030512	negative regulation of transforming growth factor beta receptor signaling pathway	0.68
ENSG00000133243	BTBD2 PPI subnetwork	0.68
GO:0015077	monovalent inorganic cation transmembrane transporter activity	0.68
GO:0003151	outflow tract morphogenesis	0.68
ENSG00000085840	ORC1 PPI subnetwork	0.68
GO:0009123	nucleoside monophosphate metabolic process	0.68
ENSG00000130204	TOMM40 PPI subnetwork	0.68
ENSG00000120708	TGFBI PPI subnetwork	0.68
ENSG00000165392	WRN PPI subnetwork	0.68
ENSG00000081985	IL12RB2 PPI subnetwork	0.68
ENSG00000175029	CTBP2 PPI subnetwork	0.68
MP:0002813	microcytosis	0.68
GO:0019898	extrinsic to membrane	0.68
ENSG00000206267	ENSG00000206267 PPI subnetwork	0.68
ENSG00000204351	SKIV2L PPI subnetwork	0.68
ENSG00000076242	MLH1 PPI subnetwork	0.68
ENSG00000076554	TPD52 PPI subnetwork	0.68
ENSG00000006451	RALA PPI subnetwork	0.68
GO:0007076	mitotic chromosome condensation	0.68
GO:0033762	response to glucagon stimulus	0.68
ENSG00000173805	HAP1 PPI subnetwork	0.68
ENSG00000206495	TRIM39 PPI subnetwork	0.68
ENSG00000206419	ENSG00000206419 PPI subnetwork	0.68
ENSG00000204599	TRIM39 PPI subnetwork	0.68
GO:0019905	syntaxin binding	0.68
GO:0061351	neural precursor cell proliferation	0.68
ENSG00000079950	STX7 PPI subnetwork	0.68
MP:0000242	impaired fertilization	0.68
REACTOME_GABA_RECEPTOR_ACTIVATION	REACTOME_GABA_RECEPTOR_ACTIVATION	0.68
ENSG00000206407	ENSG00000206407 PPI subnetwork	0.68
ENSG00000204569	PPP1R10 PPI subnetwork	0.68
ENSG00000206489	PPP1R10 PPI subnetwork	0.68
GO:0006266	DNA ligation	0.68
GO:2000179	positive regulation of neural precursor cell proliferation	0.68
GO:0044439	peroxisomal part	0.69
GO:0044438	microbody part	0.69
MP:0002626	increased heart rate	0.69
ENSG00000007392	LUC7L PPI subnetwork	0.69
GO:0060562	epithelial tube morphogenesis	0.69
ENSG00000101811	CSTF2 PPI subnetwork	0.69
ENSG00000121741	ZMYM2 PPI subnetwork	0.69
GO:0009896	positive regulation of catabolic process	0.69
GO:0031341	regulation of cell killing	0.69
GO:0005759	mitochondrial matrix	0.69
GO:0030659	cytoplasmic vesicle membrane	0.69
ENSG00000131724	IL13RA1 PPI subnetwork	0.69
KEGG_GLUTATHIONE_METABOLISM	KEGG_GLUTATHIONE_METABOLISM	0.69

Original gene set ID	Original gene set description	Nominal P value
MP:0008663	increased interleukin-12 secretion	0.69
ENSG00000173418	NAA20 PPI subnetwork	0.69
GO:0010573	vascular endothelial growth factor production	0.69
GO:0010574	regulation of vascular endothelial growth factor production	0.69
ENSG00000206353	SKIV2L PPI subnetwork	0.69
ENSG00000138814	PPP3CA PPI subnetwork	0.69
MP:0004096	abnormal midbrain-hindbrain boundary development	0.69
GO:0009880	embryonic pattern specification	0.69
ENSG00000127616	SMARCA4 PPI subnetwork	0.69
ENSG00000153046	CDYL PPI subnetwork	0.69
MP:0006036	abnormal mitochondrial physiology	0.69
GO:0007214	gamma-aminobutyric acid signaling pathway	0.69
GO:0021987	cerebral cortex development	0.69
MP:0005608	cardiac interstitial fibrosis	0.69
MP:0004532	abnormal inner hair cell stereociliary bundle morphology	0.69
ENSG00000079102	RUNX1T1 PPI subnetwork	0.69
GO:0042490	mechanoreceptor differentiation	0.69
GO:0016045	detection of bacterium	0.69
GO:0015985	energy coupled proton transport, down electrochemical gradient	0.69
GO:0015986	ATP synthesis coupled proton transport	0.69
MP:0002797	increased thigmotaxis	0.69
GO:0055003	cardiac myofibril assembly	0.69
KEGG_O_GLYCAN_BIOSYNTHESIS	KEGG_O_GLYCAN_BIOSYNTHESIS	0.69
ENSG00000011304	PTBP1 PPI subnetwork	0.69
ENSG00000130177	CDC16 PPI subnetwork	0.69
GO:0051247	positive regulation of protein metabolic process	0.69
GO:0048596	embryonic camera-type eye morphogenesis	0.69
MP:0005318	decreased triglyceride level	0.69
ENSG00000091129	NRCAM PPI subnetwork	0.69
ENSG00000112062	MAPK14 PPI subnetwork	0.69
ENSG00000156374	PCGF6 PPI subnetwork	0.69
ENSG00000142192	APP PPI subnetwork	0.69
ENSG00000116754	SRSF11 PPI subnetwork	0.69
ENSG00000173598	NUDT4 PPI subnetwork	0.69
GO:0030833	regulation of actin filament polymerization	0.69
GO:0060560	developmental growth involved in morphogenesis	0.69
ENSG00000005156	LIG3 PPI subnetwork	0.69
ENSG00000124422	USP22 PPI subnetwork	0.69
MP:0005403	abnormal nerve conduction	0.69
ENSG00000178896	EXOSC4 PPI subnetwork	0.69
ENSG00000132535	DLG4 PPI subnetwork	0.69
GO:0045649	regulation of macrophage differentiation	0.69
GO:0090307	spindle assembly involved in mitosis	0.69
GO:0043279	response to alkaloid	0.69
MP:0000432	abnormal head morphology	0.69
REACTOME_AUTODEGRADATION_OF_CDH1_BY_CDH1APCC	REACTOME_AUTODEGRADATION_OF_CDH1_BY_CDH1APCC	0.69
ENSG00000172943	PHF8 PPI subnetwork	0.69
GO:0031674	I band	0.69
ENSG00000139436	GIT2 PPI subnetwork	0.69

Original gene set ID	Original gene set description	Nominal P value
ENSG00000029363	BCLAF1 PPI subnetwork	0.69
GO:0010575	positive regulation vascular endothelial growth factor productior	0.69
ENSG00000130382	MLLT1 PPI subnetwork	0.69
ENSG00000177733	HNRNPA0 PPI subnetwork	0.69
ENSG00000111802	TDP2 PPI subnetwork	0.69
ENSG00000157601	MX1 PPI subnetwork	0.69
ENSG00000141959	PFKL PPI subnetwork	0.69
GO:0030018	Z disc	0.69
MP:0006264	decreased systemic arterial systolic blood pressure	0.69
GO:0021885	forebrain cell migration	0.69
ENSG00000139842	CUL4A PPI subnetwork	0.69
GO:0006898	receptor-mediated endocytosis	0.69
MP:0005026	decreased susceptibility to parasitic infectior	0.69
ENSG00000164418	GRIK2 PPI subnetwork	0.69
ENSG00000094631	HDAC6 PPI subnetwork	0.69
GO:0005892	acetylcholine-gated channel complex	0.69
GO:0044440	endosomal part	0.69
ENSG00000117748	RPA2 PPI subnetwork	0.69
ENSG00000164611	PTTG1 PPI subnetwork	0.69
GO:0035064	methylated histone residue binding	0.69
MP:0002914	abnormal endplate potential	0.69
ENSG00000138795	LEF1 PPI subnetwork	0.69
GO:0010810	regulation of cell-substrate adhesion	0.69
MP:0000748	progressive muscle weakness	0.69
MP:0002929	abnormal bile duct development	0.69
GO:0048485	sympathetic nervous system development	0.69
GO:0034703	cation channel complex	0.69
ENSG00000067225	PKM2 PPI subnetwork	0.69
ENSG00000142453	CARM1 PPI subnetwork	0.69
REACTOME_PIP3_ACTIVATES_AKT_SIGNALING	REACTOME_PIP3_ACTIVATES_AKT_SIGNALING	0.69
GO:0045921	positive regulation of exocytosis	0.69
GO:0016747	transferase activity, transferring acyl groups other than amino-acyl groups	0.69
MP:0000279	ventricular hypoplasia	0.69
MP:0005298	abnormal clavicle morphology	0.69
ENSG00000108344	PSMD3 PPI subnetwork	0.69
GO:0061041	regulation of wound healing	0.69
MP:0004355	short radius	0.69
MP:0005166	decreased susceptibility to injury	0.69
GO:0046320	regulation of fatty acid oxidation	0.69
MP:0008687	increased interleukin-2 secretion	0.69
MP:0003632	abnormal nervous system morphology	0.69
GO:0051480	cytosolic calcium ion homeostasis	0.69
KEGG_MISMATCH_REPAIR	KEGG_MISMATCH_REPAIR	0.69
ENSG00000185345	PARK2 PPI subnetwork	0.69
GO:0045686	negative regulation of glial cell differentiatior	0.69
REACTOME_TRANSPORT_OF_RIBONUCLEOPROTEINS_INTO_THE_HOST_NUCLEU	REACTOME_TRANSPORT_OF_RIBONUCLEOPROTEINS_INTO_THE_HOST_NUCLEUS	0.69
ENSG00000117318	ID3 PPI subnetwork	0.69
GO:0046651	lymphocyte proliferation	0.69
ENSG00000166206	GABRB3 PPI subnetwork	0.7

Original gene set ID	Original gene set description	Nominal P value
GO:0006919	activation of cysteine-type endopeptidase activity involved in apoptotic proces	0.7
GO:0002052	positive regulation of neuroblast proliferation	0.7
GO:0046635	positive regulation of alpha-beta T cell activation	0.7
GO:0030278	regulation of ossification	0.7
ENSG00000080503	SMARCA2 PPI subnetwork	0.7
ENSG00000175203	DCTN2 PPI subnetwork	0.7
ENSG00000118495	PLAGL1 PPI subnetwork	0.7
GO:0050830	defense response to Gram-positive bacterium	0.7
GO:0032570	response to progesterone stimulus	0.7
GO:0042451	purine nucleoside biosynthetic process	0.7
GO:0042455	ribonucleoside biosynthetic process	0.7
GO:0046129	purine ribonucleoside biosynthetic process	0.7
ENSG00000173465	SSSCA1 PPI subnetwork	0.7
GO:0022836	gated channel activity	0.7
ENSG00000168005	C11orf84 PPI subnetwork	0.7
GO:0015459	potassium channel regulator activity	0.7
ENSG00000039068	CDH1 PPI subnetwork	0.7
GO:0002495	antigen processing and presentation of peptide antigen via MHC class I	0.7
REACTOME_DAG_AND_IP3_SIGNALING	REACTOME_DAG_AND_IP3_SIGNALING	0.7
ENSG00000136026	CKAP4 PPI subnetwork	0.7
ENSG00000112640	PPP2R5D PPI subnetwork	0.7
ENSG00000145332	KLHL8 PPI subnetwork	0.7
GO:0051146	striated muscle cell differentiation	0.7
ENSG00000142945	KIF2C PPI subnetwork	0.7
ENSG00000148180	GSN PPI subnetwork	0.7
ENSG00000107295	SH3GL2 PPI subnetwork	0.7
ENSG00000059378	PARP12 PPI subnetwork	0.7
GO:0048488	synaptic vesicle endocytosis	0.7
ENSG00000163440	PDCL2 PPI subnetwork	0.7
MP:0005542	corneal vascularization	0.7
ENSG00000173702	MUC13 PPI subnetwork	0.7
ENSG00000186879	ENSG00000186879 PPI subnetwork	0.7
GO:0050829	defense response to Gram-negative bacterium	0.7
GO:2000736	regulation of stem cell differentiation	0.7
GO:0030516	regulation of axon extension	0.7
GO:0051301	cell division	0.7
GO:0006942	regulation of striated muscle contraction	0.7
GO:0004112	cyclic-nucleotide phosphodiesterase activity	0.7
MP:0005333	decreased heart rate	0.7
GO:0022029	telencephalon cell migration	0.7
MP:0001924	infertility	0.7
MP:0011228	abnormal vitamin D level	0.7
ENSG00000147130	ZMYM3 PPI subnetwork	0.7
ENSG00000082258	CCNT2 PPI subnetwork	0.7
GO:0009416	response to light stimulus	0.7
REACTOME_AQUAPORIN:MEDIATED_TRANSPORT	REACTOME_AQUAPORIN:MEDIATED_TRANSPORT	0.7
MP:0004113	abnormal aortic arch morphology	0.7
MP:0004398	cochlear inner hair cell degeneration	0.7
MP:0004505	decreased renal glomerulus number	0.7

Original gene set ID	Original gene set description	Nominal P value
GO:0007187	G-protein coupled receptor signaling pathway, coupled to cyclic nucleotide second r	0.7
GO:0032039	integrator complex	0.7
ENSG00000128340	RAC2 PPI subnetwork	0.7
GO:0003001	generation of a signal involved in cell-cell signaling	0.7
GO:0023061	signal release	0.7
MP:0008502	increased IgG3 level	0.7
GO:0000421	autophagic vacuole membrane	0.7
GO:0014014	negative regulation of gliogenesis	0.7
MP:0002492	decreased IgE level	0.7
ENSG00000132182	NUP210 PPI subnetwork	0.7
MP:0002663	failure to form blastocele	0.7
GO:0031575	mitotic cell cycle G1/S transition checkpoint	0.7
GO:0046488	phosphatidylinositol metabolic process	0.7
ENSG00000196911	KPNA5 PPI subnetwork	0.7
MP:0001426	polydipsia	0.7
ENSG00000148229	POLE3 PPI subnetwork	0.7
ENSG00000183684	ALYREF PPI subnetwork	0.7
KEGG_BASE_EXCISION_REPAIR	KEGG_BASE_EXCISION_REPAIR	0.7
GO:0051960	regulation of nervous system development	0.7
REACTOME_ASSOCIATION_OF_LICENSING_FACTORS_WITH_THE_PRE:REPLICAT	REACTOME_ASSOCIATION_OF_LICENSING_FACTORS_WITH_THE_PRE:REPLICATIVE_	0.7
MP:0006030	abnormal otic vesicle development	0.7
ENSG00000166971	AKTIP PPI subnetwork	0.7
REACTOME_SIGNALING_BY_PDGF	REACTOME_SIGNALING_BY_PDGF	0.7
MP:0008181	increased marginal zone B cell number	0.7
GO:0010874	regulation of cholesterol efflux	0.7
ENSG00000085063	CD59 PPI subnetwork	0.7
GO:0008076	voltage-gated potassium channel complex	0.7
GO:0034705	potassium channel complex	0.7
GO:0035137	hindlimb morphogenesis	0.7
GO:0014048	regulation of glutamate secretion	0.7
ENSG00000163875	MEAF6 PPI subnetwork	0.7
ENSG00000156299	TIAM1 PPI subnetwork	0.7
ENSG00000188229	TUBB4B PPI subnetwork	0.7
GO:0060415	muscle tissue morphogenesis	0.7
ENSG00000100368	CSF2RB PPI subnetwork	0.7
ENSG00000134717	BTF3L4 PPI subnetwork	0.7
KEGG_DILATED_CARDIOMYOPATHY	KEGG_DILATED_CARDIOMYOPATHY	0.7
GO:0008234	cysteine-type peptidase activity	0.7
GO:0045216	cell-cell junction organization	0.7
MP:0008734	decreased susceptibility to endotoxin shock	0.7
ENSG00000103343	ZNF174 PPI subnetwork	0.7
ENSG00000120875	DUSP4 PPI subnetwork	0.7
MP:0002941	increased circulating alanine transaminase leve	0.7
GO:0009791	post-embryonic development	0.7
GO:0050804	regulation of synaptic transmission	0.7
GO:0030136	clathrin-coated vesicle	0.7
GO:0050994	regulation of lipid catabolic process	0.7
ENSG00000013561	RNF14 PPI subnetwork	0.7
GO:0030808	regulation of nucleotide biosynthetic process	0.7

Original gene set ID	Original gene set description	Nominal P value
GO:0030802	regulation of cyclic nucleotide biosynthetic process	0.7
MP:0000480	increased rib number	0.7
ENSG00000127588	GNG13 PPI subnetwork	0.7
GO:0033116	endoplasmic reticulum-Golgi intermediate compartment membrane	0.7
ENSG00000126226	PCID2 PPI subnetwork	0.7
GO:0032642	regulation of chemokine production	0.7
REACTOME_SIGNALING_BY_EGFR	REACTOME_SIGNALING_BY_EGFR	0.7
GO:0032943	mononuclear cell proliferation	0.7
GO:0030162	regulation of proteolysis	0.7
GO:0008154	actin polymerization or depolymerization	0.7
REACTOME_DCC_MEDIATED_ATTRACTIVE_SIGNALING	REACTOME_DCC_MEDIATED_ATTRACTIVE_SIGNALING	0.71
REACTOME_RNA_POLYMERASE_I_TRANSCRIPTION	REACTOME_RNA_POLYMERASE_I_TRANSCRIPTION	0.71
MP:0000520	absent kidney	0.71
REACTOME_POTASSIUM_CHANNELS	REACTOME_POTASSIUM_CHANNELS	0.71
GO:0048486	parasympathetic nervous system development	0.71
GO:0005884	actin filament	0.71
ENSG00000173120	KDM2A PPI subnetwork	0.71
REACTOME_G1S_TRANSITION	REACTOME_G1S_TRANSITION	0.71
GO:0006007	glucose catabolic process	0.71
MP:0002608	increased hematocrit	0.71
GO:0010720	positive regulation of cell development	0.71
GO:0030521	androgen receptor signaling pathway	0.71
GO:0072531	pyrimidine-containing compound transmembrane transport	0.71
KEGG_B_CELL_RECEPTOR_SIGNALING_PATHWAY	KEGG_B_CELL_RECEPTOR_SIGNALING_PATHWAY	0.71
MP:0000823	abnormal lateral ventricle morphology	0.71
MP:0002910	abnormal excitatory postsynaptic currents	0.71
REACTOME_GAP:FILLING_DNA_REPAIR_SYNTHESIS_AND_LIGATION_IN_TC:NER	REACTOME_GAP:FILLING_DNA_REPAIR_SYNTHESIS_AND_LIGATION_IN_TC:NER	0.71
REACTOME_GAP:FILLING_DNA_REPAIR_SYNTHESIS_AND_LIGATION_IN_GG:NEI	REACTOME_GAP:FILLING_DNA_REPAIR_SYNTHESIS_AND_LIGATION_IN_GG:NER	0.71
ENSG00000104164	PLDN PPI subnetwork	0.71
ENSG00000102312	PORCN PPI subnetwork	0.71
MP:0005176	eyelids fail to open	0.71
ENSG00000122585	NPY PPI subnetwork	0.71
REACTOME_TAT:MEDIATED_HIV:1_ELONGATION_ARREST_AND_RECOVERY	REACTOME_TAT:MEDIATED_HIV:1_ELONGATION_ARREST_AND_RECOVERY	0.71
REACTOME_PAUSING_AND_RECOVERY_OF_TAT:MEDIATED_HIV:1_ELONGATIO	REACTOME_PAUSING_AND_RECOVERY_OF_TAT:MEDIATED_HIV:1_ELONGATION	0.71
GO:0000776	kinetochore	0.71
ENSG00000111664	GNB3 PPI subnetwork	0.71
MP:0008034	enhanced lipolysis	0.71
GO:0002237	response to molecule of bacterial origin	0.71
GO:0004536	deoxyribonuclease activity	0.71
ENSG00000163017	ACTG2 PPI subnetwork	0.71
GO:0060048	cardiac muscle contraction	0.71
GO:0009410	response to xenobiotic stimulus	0.71
GO:0071466	cellular response to xenobiotic stimulus	0.71
ENSG00000164105	SAP30 PPI subnetwork	0.71
GO:0016042	lipid catabolic process	0.71
GO:0032496	response to lipopolysaccharide	0.71
GO:0012506	vesicle membrane	0.71
ENSG00000152413	HOMER1 PPI subnetwork	0.71
REACTOME_G_ALPHA_Q_SIGNALLING_EVENTS	REACTOME_G_ALPHA_Q_SIGNALLING_EVENTS	0.71

Original gene set ID	Original gene set description	Nominal P value
REACTOME_INWARDLY_RECTIFYING_K_CHANNELS	REACTOME_INWARDLY_RECTIFYING_K_CHANNELS	0.71
ENSG00000104142	VPS18 PPI subnetwork	0.71
ENSG00000153827	TRIP12 PPI subnetwork	0.71
ENSG00000183741	CBX6 PPI subnetwork	0.71
GO:0030177	positive regulation of Wnt receptor signaling pathway	0.71
MP:0002961	abnormal axon guidance	0.71
ENSG00000111481	COPZ1 PPI subnetwork	0.71
REACTOME_DOUBLE:STRAND_BREAK_REPAIR	REACTOME_DOUBLE:STRAND_BREAK_REPAIR	0.71
GO:0010594	regulation of endothelial cell migration	0.71
MP:0000952	abnormal CNS glial cell morphology	0.71
ENSG00000130165	ELOF1 PPI subnetwork	0.71
REACTOME_SYNTHESIS_OF_SUBSTRATES_IN_N:GLYCAN_BIOSYTHESIS	REACTOME_SYNTHESIS_OF_SUBSTRATES_IN_N:GLYCAN_BIOSYTHESIS	0.71
MP:0000343	altered response to myocardial infarction	0.71
ENSG00000119421	NDUFA8 PPI subnetwork	0.71
ENSG00000104221	BRF2 PPI subnetwork	0.71
MP:0001585	hemolytic anemia	0.71
ENSG00000100321	SYNGR1 PPI subnetwork	0.71
GO:0031333	negative regulation of protein complex assembly	0.71
ENSG00000152578	GRIA4 PPI subnetwork	0.71
ENSG00000163882	POLR2H PPI subnetwork	0.71
GO:0001105	RNA polymerase II transcription coactivator activity	0.71
GO:0001190	RNA polymerase II transcription factor binding transcription factor activity involved	0.71
GO:0010822	positive regulation of mitochondrion organization	0.71
REACTOME_VIF:MEDIATED_DEGRADATION_OF_APOBEC3G	REACTOME_VIF:MEDIATED_DEGRADATION_OF_APOBEC3G	0.71
GO:0031668	cellular response to extracellular stimulus	0.71
ENSG00000121774	KHDRBS1 PPI subnetwork	0.71
GO:0017017	MAP kinase tyrosine/serine/threonine phosphatase activity	0.71
ENSG00000175595	ERCC4 PPI subnetwork	0.71
ENSG00000115844	DLX2 PPI subnetwork	0.71
GO:0002200	somatic diversification of immune receptors	0.71
MP:0009038	decreased inferior colliculus size	0.71
GO:0001704	formation of primary germ layer	0.71
MP:0001764	abnormal homeostasis	0.71
MP:0000749	muscle degeneration	0.71
MP:0001360	abnormal social investigation	0.71
ENSG00000101096	NFATC2 PPI subnetwork	0.71
ENSG00000126432	PRDX5 PPI subnetwork	0.71
GO:0008380	RNA splicing	0.71
GO:0048644	muscle organ morphogenesis	0.71
ENSG00000116761	CTH PPI subnetwork	0.71
GO:0002088	lens development in camera-type eye	0.71
GO:0031960	response to corticosteroid stimulus	0.71
MP:0000787	abnormal telencephalon morphology	0.71
GO:0034754	cellular hormone metabolic process	0.71
MP:0005028	abnormal trophectoderm morphology	0.71
GO:0042287	MHC protein binding	0.71
GO:0046165	alcohol biosynthetic process	0.71
REACTOME_G1S_DNA_DAMAGE_CHECKPOINTS	REACTOME_G1S_DNA_DAMAGE_CHECKPOINTS	0.71
GO:0004091	carboxylesterase activity	0.71

Original gene set ID	Original gene set description	Nominal P value
GO:0048489	synaptic vesicle transport	0.71
MP:0005617	increased susceptibility to type IV hypersensitivity reactior	0.71
MP:0005459	decreased percent body fat	0.71
ENSG00000168439	STIP1 PPI subnetwork	0.71
GO:0002793	positive regulation of peptide secretior	0.71
GO:0006378	mRNA polyadenylation	0.71
MP:0000298	absent atrioventricular cushions	0.71
GO:0010008	endosome membrane	0.71
MP:0004204	absent stapes	0.71
ENSG00000185008	ROBO2 PPI subnetwork	0.71
GO:0044427	chromosomal part	0.71
REACTOME_HIV:1_ELONGATION_ARREST_AND_RECOVERY	REACTOME_HIV:1_ELONGATION_ARREST_AND_RECOVERY	0.71
REACTOME_PAUSING_AND_RECOVERY_OF_HIV:1_ELONGATION	REACTOME_PAUSING_AND_RECOVERY_OF_HIV:1_ELONGATION	0.71
REACTOME_PAUSING_AND_RECOVERY_OF_ELONGATION	REACTOME_PAUSING_AND_RECOVERY_OF_ELONGATION	0.71
REACTOME_ELONGATION_ARREST_AND_RECOVERY	REACTOME_ELONGATION_ARREST_AND_RECOVERY	0.71
MP:0010551	abnormal coronary vessel morphology	0.71
GO:0070330	aromatase activity	0.71
ENSG00000138709	LARP1B PPI subnetwork	0.71
ENSG00000033122	LRRC7 PPI subnetwork	0.71
MP:0008536	enlarged third ventricle	0.71
GO:0002562	somatic diversification of immune receptors via germline recombination within a sir	0.71
GO:0016444	somatic cell DNA recombination	0.71
MP:0011353	expanded mesangial matrix	0.71
ENSG00000158769	F11R PPI subnetwork	0.71
GO:0046058	cAMP metabolic process	0.71
ENSG00000017797	RALBP1 PPI subnetwork	0.71
GO:0006354	transcription elongation, DNA-dependent	0.71
REACTOME_GABA_B_RECEPTOR_ACTIVATION	REACTOME_GABA_B_RECEPTOR_ACTIVATION	0.71
REACTOME_ACTIVATION_OF_GABAB_RECEPTORS	REACTOME_ACTIVATION_OF_GABAB_RECEPTORS	0.71
GO:0035116	embryonic hindlimb morphogenesis	0.71
GO:0000784	nuclear chromosome, telomeric region	0.71
ENSG00000129562	DAD1 PPI subnetwork	0.71
MP:0000536	hydroureter	0.71
ENSG00000122756	CNTFR PPI subnetwork	0.72
ENSG00000124642	ENSG00000124642 PPI subnetwork	0.72
ENSG00000184825	HIST1H2AH PPI subnetwork	0.72
GO:0005487	nucleocytoplasmic transporter activity	0.72
ENSG00000162231	NXF1 PPI subnetwork	0.72
GO:0051241	negative regulation of multicellular organismal process	0.72
ENSG00000133119	RFC3 PPI subnetwork	0.72
MP:0003728	abnormal retinal photoreceptor layer morphology	0.72
GO:0031683	G-protein beta/gamma-subunit complex binding	0.72
ENSG00000180573	HIST1H2AC PPI subnetwork	0.72
GO:0051262	protein tetramerization	0.72
GO:0010927	cellular component assembly involved in morphogenesis	0.72
ENSG00000118515	SGK1 PPI subnetwork	0.72
ENSG00000101306	MYLK2 PPI subnetwork	0.72
ENSG00000174720	LARP7 PPI subnetwork	0.72
GO:0019935	cyclic-nucleotide-mediated signaling	0.72

Original gene set ID	Original gene set description	Nominal P value
GO:0045777	positive regulation of blood pressure	0.72
REACTOME_PRESYNAPTIC_NICOTINIC_ACETYLCHOLINE_RECEPTORS	REACTOME_PRESYNAPTIC_NICOTINIC_ACETYLCHOLINE_RECEPTORS	0.72
ENSG00000137403	HLA-F PPI subnetwork	0.72
ENSG00000213066	FGFR1OP PPI subnetwork	0.72
ENSG00000157766	ACAN PPI subnetwork	0.72
GO:0032451	demethylase activity	0.72
REACTOME_CROSS:PRESENTATION_OF_SOLUBLE_EXOGENOUS_ANTIGENS_END	REACTOME_CROSS:PRESENTATION_OF_SOLUBLE_EXOGENOUS_ANTIGENS_ENDOS(	0.72
ENSG00000120837	NFYB PPI subnetwork	0.72
GO:0000226	microtubule cytoskeleton organization	0.72
GO:0051145	smooth muscle cell differentiation	0.72
ENSG00000111052	LIN7A PPI subnetwork	0.72
ENSG00000151247	EIF4E PPI subnetwork	0.72
ENSG00000130640	TUBGCP2 PPI subnetwork	0.72
GO:0010001	glial cell differentiation	0.72
MP:0005587	abnormal Meckel's cartilage morphology	0.72
GO:0015074	DNA integration	0.72
GO:0048715	negative regulation of oligodendrocyte differentiation	0.72
ENSG00000164975	SNAPC3 PPI subnetwork	0.72
ENSG00000158290	CUL4B PPI subnetwork	0.72
ENSG00000125743	SNRPD2 PPI subnetwork	0.72
ENSG00000138685	FGF2 PPI subnetwork	0.72
GO:0006364	rRNA processing	0.72
GO:0042092	type 2 immune response	0.72
REACTOME_RNA_POLYMERASE_I_PROMOTER_OPENING	REACTOME_RNA_POLYMERASE_I_PROMOTER_OPENING	0.72
GO:0033180	proton-transporting V-type ATPase, V1 domain	0.72
GO:0030509	BMP signaling pathway	0.72
GO:0035115	embryonic forelimb morphogenesis	0.72
ENSG00000115594	IL1R1 PPI subnetwork	0.72
GO:0048747	muscle fiber development	0.72
GO:0010744	positive regulation of macrophage derived foam cell differentiation	0.72
MP:0002196	absent corpus callosum	0.72
REACTOME_SYNTHESIS_SECRETION_AND_INACTIVATION_OF_GLUCAGON:LIKE_	REACTOME_SYNTHESIS_SECRETION_AND_INACTIVATION_OF_GLUCAGON:LIKE_PEF	0.72
REACTOME_INCRETIN_SYNTHESIS_SECRETION_AND_INACTIVATION	REACTOME_INCRETIN_SYNTHESIS_SECRETION_AND_INACTIVATION	0.72
GO:0006643	membrane lipid metabolic process	0.72
MP:0001921	reduced fertility	0.72
GO:0010463	mesenchymal cell proliferation	0.72
MP:0001685	abnormal endoderm development	0.72
ENSG00000104388	RAB2A PPI subnetwork	0.72
MP:0001065	abnormal trigeminal nerve morphology	0.72
ENSG00000104320	NBN PPI subnetwork	0.72
MP:0005656	decreased aggression	0.72
ENSG00000215902	ENSG00000215902 PPI subnetwork	0.72
REACTOME_G:PROTEIN_MEDIATED_EVENTS	REACTOME_G:PROTEIN_MEDIATED_EVENTS	0.72
ENSG00000137672	TRPC6 PPI subnetwork	0.72
GO:0009190	cyclic nucleotide biosynthetic process	0.72
GO:0010833	telomere maintenance via telomere lengthening	0.72
MP:0001701	incomplete embryo turning	0.72
GO:0046365	monosaccharide catabolic process	0.72
ENSG00000149782	PLCB3 PPI subnetwork	0.72

Original gene set ID	Original gene set description	Nominal P value
GO:0008080	N-acetyltransferase activity	0.72
MP:0003354	astrocytosis	0.72
GO:0007041	lysosomal transport	0.72
ENSG00000082898	XPO1 PPI subnetwork	0.72
GO:0030119	AP-type membrane coat adaptor complex	0.72
GO:0090277	positive regulation of peptide hormone secretion	0.72
ENSG00000106399	RPA3 PPI subnetwork	0.72
ENSG00000164867	NOS3 PPI subnetwork	0.72
ENSG00000177700	POLR2L PPI subnetwork	0.72
ENSG00000079805	DNM2 PPI subnetwork	0.72
ENSG00000113196	HAND1 PPI subnetwork	0.72
ENSG00000104879	CKM PPI subnetwork	0.72
GO:0007492	endoderm development	0.72
ENSG00000110148	CCKBR PPI subnetwork	0.72
ENSG00000033050	ABCF2 PPI subnetwork	0.72
GO:0072132	mesenchyme morphogenesis	0.72
MP:0002020	increased tumor incidence	0.72
REACTOME_PROCESSIVE_SYNTHESIS_ON_THE_C:STRAND_OF_THE_TELOMERE	REACTOME_PROCESSIVE_SYNTHESIS_ON_THE_C:STRAND_OF_THE_TELOMERE	0.72
GO:0031406	carboxylic acid binding	0.72
GO:0031901	early endosome membrane	0.72
ENSG00000153767	GTF2E1 PPI subnetwork	0.72
ENSG00000118491	C6orf94 PPI subnetwork	0.72
ENSG00000181789	COPG PPI subnetwork	0.72
GO:0004549	tRNA-specific ribonuclease activity	0.72
ENSG00000024048	UBR2 PPI subnetwork	0.72
GO:0021532	neural tube patterning	0.72
MP:0003070	increased vascular permeability	0.72
GO:0008589	regulation of smoothened signaling pathway	0.72
ENSG00000100836	PABPN1 PPI subnetwork	0.72
GO:0030199	collagen fibril organization	0.72
REACTOME_RNA_POLYMERASE_I_PROMOTER_CLEARANCE	REACTOME_RNA_POLYMERASE_I_PROMOTER_CLEARANCE	0.72
GO:0032602	chemokine production	0.72
MP:0005165	increased susceptibility to injury	0.72
ENSG00000104626	ERI1 PPI subnetwork	0.72
MP:0001566	hyperphosphatemia	0.73
GO:0043679	axon terminus	0.73
GO:2000677	regulation of transcription regulatory region DNA binding	0.73
GO:0032319	regulation of Rho GTPase activity	0.73
ENSG00000204086	RPA4 PPI subnetwork	0.73
ENSG00000113318	MSH3 PPI subnetwork	0.73
ENSG00000103994	ZFP106 PPI subnetwork	0.73
GO:0006805	xenobiotic metabolic process	0.73
MP:0010769	abnormal survival	0.73
ENSG00000215727	ENSG00000215727 PPI subnetwork	0.73
ENSG00000065518	NDUFB4 PPI subnetwork	0.73
MP:0004543	abnormal sperm physiology	0.73
GO:0004693	cyclin-dependent protein kinase activity	0.73
ENSG00000186184	POLR1D PPI subnetwork	0.73
REACTOME_ACTIVATION_OF_NMDA_RECEPTOR_UPON_GLUTAMATE_BINDING	REACTOME_ACTIVATION_OF_NMDA_RECEPTOR_UPON_GLUTAMATE_BINDING_AN	0.73

Original gene set ID	Original gene set description	Nominal P value
GO:0048291	isotype switching to IgG isotypes	0.73
ENSG00000088367	EPB41L1 PPI subnetwork	0.73
GO:0030917	midbrain-hindbrain boundary development	0.73
GO:2001252	positive regulation of chromosome organization	0.73
REACTOME_TRANSCRIPTION	REACTOME_TRANSCRIPTION	0.73
GO:0006541	glutamine metabolic process	0.73
GO:0051051	negative regulation of transport	0.73
GO:0060429	epithelium development	0.73
MP:0002757	decreased vertical activity	0.73
ENSG00000153774	CFDP1 PPI subnetwork	0.73
GO:0010464	regulation of mesenchymal cell proliferation	0.73
ENSG00000023318	ERP44 PPI subnetwork	0.73
GO:0060322	head development	0.73
GO:0031954	positive regulation of protein autophosphorylation	0.73
MP:0004965	inner cell mass degeneration	0.73
GO:0044460	flagellum part	0.73
GO:0044442	microtubule-based flagellum part	0.73
ENSG00000167986	DDB1 PPI subnetwork	0.73
GO:0007193	adenylate cyclase-inhibiting G-protein coupled receptor signaling pathway	0.73
GO:0048302	regulation of isotype switching to IgG isotypes	0.73
KEGG_SYSTEMIC_LUPUS_ERYTHEMATOSUS	KEGG_SYSTEMIC_LUPUS_ERYTHEMATOSUS	0.73
GO:0004930	G-protein coupled receptor activity	0.73
ENSG00000168539	CHRM1 PPI subnetwork	0.73
ENSG00000125977	EIF2S2 PPI subnetwork	0.73
GO:0072511	divalent inorganic cation transport	0.73
ENSG00000104637	ENSG00000104637 PPI subnetwork	0.73
GO:0048663	neuron fate commitment	0.73
GO:0042165	neurotransmitter binding	0.73
ENSG00000154917	RAB6B PPI subnetwork	0.73
ENSG00000154016	GRAP PPI subnetwork	0.73
ENSG00000185518	SV2B PPI subnetwork	0.73
REACTOME_HOST_INTERACTIONS_OF_HIV_FACTORS	REACTOME_HOST_INTERACTIONS_OF_HIV_FACTORS	0.73
ENSG00000214133	ENSG00000214133 PPI subnetwork	0.73
ENSG00000158623	COPG2 PPI subnetwork	0.73
MP:0003656	abnormal erythrocyte physiology	0.73
ENSG00000152234	ATP5A1 PPI subnetwork	0.73
GO:0048675	axon extension	0.73
ENSG00000163539	CLASP2 PPI subnetwork	0.73
ENSG00000111653	ING4 PPI subnetwork	0.73
ENSG00000084463	WBP11 PPI subnetwork	0.73
GO:0048551	metalloenzyme inhibitor activity	0.73
GO:0008191	metalloendopeptidase inhibitor activity	0.73
MP:0001008	abnormal sympathetic ganglion morphology	0.73
MP:0003047	abnormal thoracic vertebrae morphology	0.73
MP:0005153	abnormal B cell proliferation	0.73
MP:0005225	abnormal vertebrae development	0.73
ENSG00000147202	DIAPH2 PPI subnetwork	0.73
ENSG00000049541	RFC2 PPI subnetwork	0.73
ENSG00000101608	MYL12A PPI subnetwork	0.73

Original gene set ID	Original gene set description	Nominal P value
GO:0061136	regulation of proteasomal protein catabolic process	0.73
MP:0003658	abnormal capillary morphology	0.73
ENSG00000177963	RIC8A PPI subnetwork	0.73
ENSG00000129991	TNNI3 PPI subnetwork	0.73
ENSG00000111581	NUP107 PPI subnetwork	0.73
MP:0009243	hairpin sperm flagellum	0.73
ENSG00000205542	TMSB4X PPI subnetwork	0.73
MP:0004811	abnormal neuron physiology	0.73
GO:0030888	regulation of B cell proliferation	0.73
ENSG00000099817	POLR2E PPI subnetwork	0.73
MP:0008566	increased interferon-gamma secretion	0.73
REACTOME_EGFR_INTERACTS_WITH_PHOSPHOLIPASE_C:GAMMA	REACTOME_EGFR_INTERACTS_WITH_PHOSPHOLIPASE_C:GAMMA	0.73
GO:0006779	porphyrin-containing compound biosynthetic process	0.73
GO:0033014	tetrapyrrole biosynthetic process	0.73
ENSG00000162188	GNG3 PPI subnetwork	0.73
REACTOME_ACTIVATION_OF_ATR_IN_RESPONSE_TO_REPLICATION_STRESS	REACTOME_ACTIVATION_OF_ATR_IN_RESPONSE_TO_REPLICATION_STRESS	0.73
MP:0005578	teratozoospermia	0.73
MP:0003043	hypoalgesia	0.73
ENSG00000174437	ATP2A2 PPI subnetwork	0.73
GO:0016859	cis-trans isomerase activity	0.73
GO:0051260	protein homooligomerization	0.73
ENSG00000103035	PSMD7 PPI subnetwork	0.73
GO:2000826	regulation of heart morphogenesis	0.73
MP:0008044	increased NK cell number	0.73
ENSG00000177380	PPFIA3 PPI subnetwork	0.73
MP:0001625	cardiac hypertrophy	0.73
MP:0011110	partial preweaning lethality	0.73
GO:0048515	spermatid differentiation	0.73
MP:0002128	abnormal blood circulation	0.73
GO:0000387	spliceosomal snRNP assembly	0.73
MP:0001900	impaired synaptic plasticity	0.73
GO:0032154	cleavage furrow	0.73
ENSG00000055732	MCOLN3 PPI subnetwork	0.73
GO:0007603	phototransduction, visible light	0.73
MP:0002163	abnormal gland morphology	0.73
ENSG00000160224	AIRE PPI subnetwork	0.73
GO:0044455	mitochondrial membrane part	0.73
REACTOME_BOTULINUM_NEUROTOXICITY	REACTOME_BOTULINUM_NEUROTOXICITY	0.73
ENSG00000143256	PFDN2 PPI subnetwork	0.73
GO:0030131	clathrin adaptor complex	0.73
GO:0007029	endoplasmic reticulum organization	0.73
ENSG00000067182	TNFRSF1A PPI subnetwork	0.74
GO:0051918	negative regulation of fibrinolysis	0.74
REACTOME_MRNA_DECAY_BY_5_TO_3_EXORIBONUCLEASE	REACTOME_MRNA_DECAY_BY_5_TO_3_EXORIBONUCLEASE	0.74
MP:0009548	abnormal platelet aggregation	0.74
GO:0007034	vacuolar transport	0.74
GO:0001505	regulation of neurotransmitter levels	0.74
MP:0005012	decreased eosinophil cell number	0.74
MP:0011049	impaired adaptive thermogenesis	0.74

Original gene set ID	Original gene set description	Nominal P value
GO:0055117	regulation of cardiac muscle contraction	0.74
GO:0010543	regulation of platelet activation	0.74
MP:0000519	hydronephrosis	0.74
ENSG00000096996	IL12RB1 PPI subnetwork	0.74
ENSG00000196792	STRN3 PPI subnetwork	0.74
GO:0031343	positive regulation of cell killing	0.74
GO:0003755	peptidyl-prolyl cis-trans isomerase activity	0.74
GO:0009187	cyclic nucleotide metabolic process	0.74
MP:0003312	abnormal locomotor coordination	0.74
ENSG00000136937	NCBP1 PPI subnetwork	0.74
MP:0003014	abnormal kidney medulla morphology	0.74
GO:0000184	nuclear-transcribed mRNA catabolic process, nonsense-mediated decay	0.74
KEGG_HEMATOPOIETIC_CELL_LINEAGE	KEGG_HEMATOPOIETIC_CELL_LINEAGE	0.74
ENSG00000105248	CCDC94 PPI subnetwork	0.74
GO:0031571	mitotic cell cycle G1/S transition DNA damage checkpoint	0.74
ENSG00000164162	ANAPC10 PPI subnetwork	0.74
GO:0022843	voltage-gated cation channel activity	0.74
ENSG00000104863	LIN7B PPI subnetwork	0.74
ENSG00000115306	SPTBN1 PPI subnetwork	0.74
GO:0007338	single fertilization	0.74
ENSG00000101144	BMP7 PPI subnetwork	0.74
KEGG_ETHER_LIPID_METABOLISM	KEGG_ETHER_LIPID_METABOLISM	0.74
ENSG00000196367	TRRAP PPI subnetwork	0.74
ENSG00000075151	EIF4G3 PPI subnetwork	0.74
MP:0003056	abnormal hyoid bone morphology	0.74
ENSG00000114450	GNB4 PPI subnetwork	0.74
GO:0016614	oxidoreductase activity, acting on CH-OH group of donors	0.74
GO:0008301	DNA binding, bending	0.74
GO:0004520	endodeoxyribonuclease activity	0.74
REACTOME_PLC:GAMMA1_SIGNALLING	REACTOME_PLC:GAMMA1_SIGNALLING	0.74
ENSG00000101745	ANKRD12 PPI subnetwork	0.74
GO:0042063	gliogenesis	0.74
ENSG00000136146	MED4 PPI subnetwork	0.74
ENSG00000143384	MCL1 PPI subnetwork	0.74
GO:0042645	mitochondrial nucleoid	0.74
MP:0002885	abnormal AMPA-mediated synaptic currents	0.74
GO:0031570	DNA integrity checkpoint	0.74
ENSG00000102554	KLF5 PPI subnetwork	0.74
REACTOME_RNA_POLYMERASE_I_CHAIN_ELONGATION	REACTOME_RNA_POLYMERASE_I_CHAIN_ELONGATION	0.74
ENSG00000172850	LSM2 PPI subnetwork	0.74
ENSG00000204392	LSM2 PPI subnetwork	0.74
ENSG00000111987	ENSG00000111987 PPI subnetwork	0.74
ENSG00000106367	AP1S1 PPI subnetwork	0.74
ENSG00000198223	CSF2RA PPI subnetwork	0.74
GO:0007600	sensory perception	0.74
GO:0000288	nuclear-transcribed mRNA catabolic process, deadenylation-dependent decay	0.74
GO:0009206	purine ribonucleoside triphosphate biosynthetic process	0.74
GO:0005740	mitochondrial envelope	0.74
GO:0030282	bone mineralization	0.74

Original gene set ID	Original gene set description	Nominal P value
GO:0030500	regulation of bone mineralization	0.74
ENSG00000104824	HNRNPL PPI subnetwork	0.74
ENSG00000166793	YPEL4 PPI subnetwork	0.74
ENSG00000091140	DLD PPI subnetwork	0.74
GO:0015101	organic cation transmembrane transporter activity	0.74
GO:0050796	regulation of insulin secretion	0.74
ENSG00000141759	TXNL4A PPI subnetwork	0.74
MP:0001322	abnormal iris morphology	0.74
MP:0000372	irregular coat pigmentation	0.74
GO:0033121	regulation of purine nucleotide catabolic process	0.74
GO:0030811	regulation of nucleotide catabolic process	0.74
ENSG00000140939	NOL3 PPI subnetwork	0.74
ENSG00000087365	SF3B2 PPI subnetwork	0.74
GO:0051050	positive regulation of transport	0.74
GO:0032279	asymmetric synapse	0.74
GO:0043195	terminal button	0.74
GO:0019233	sensory perception of pain	0.74
REACTOME_NCAM_SIGNALING_FOR_NEURITE_OUT:GROWTH	REACTOME_NCAM_SIGNALING_FOR_NEURITE_OUT:GROWTH	0.74
GO:0030532	small nuclear ribonucleoprotein complex	0.74
ENSG00000185825	BCAP31 PPI subnetwork	0.74
ENSG00000156973	PDE6D PPI subnetwork	0.74
ENSG00000136717	BIN1 PPI subnetwork	0.74
MP:0001606	impaired hematopoiesis	0.74
ENSG00000176406	RIMS2 PPI subnetwork	0.74
GO:0051917	regulation of fibrinolysis	0.74
GO:0050773	regulation of dendrite development	0.74
GO:0050885	neuromuscular process controlling balance	0.74
GO:0060065	uterus development	0.74
GO:0001938	positive regulation of endothelial cell proliferation	0.74
ENSG00000153071	DAB2 PPI subnetwork	0.74
GO:0048841	regulation of axon extension involved in axon guidance	0.74
GO:0042269	regulation of natural killer cell mediated cytotoxicity	0.74
GO:0002715	regulation of natural killer cell mediated immunity	0.74
GO:0006457	protein folding	0.74
GO:0003209	cardiac atrium morphogenesis	0.74
ENSG00000131187	F12 PPI subnetwork	0.74
MP:0004008	abnormal GABA-mediated receptor currents	0.74
MP:0000813	abnormal hippocampus layer morphology	0.74
ENSG00000183431	SF3A3 PPI subnetwork	0.74
ENSG00000114127	XRN1 PPI subnetwork	0.74
MP:0009379	abnormal foot pigmentation	0.74
REACTOME_CLASS_B2_SECRETIN_FAMILY_RECEPTORS	REACTOME_CLASS_B2_SECRETIN_FAMILY_RECEPTORS	0.74
ENSG00000161040	FBXL13 PPI subnetwork	0.74
MP:0002237	abnormal nasal cavity morphology	0.74
ENSG00000115540	MOB4 PPI subnetwork	0.74
GO:0040018	positive regulation of multicellular organism growth	0.74
ENSG00000126456	IRF3 PPI subnetwork	0.74
ENSG00000186842	ENSG00000186842 PPI subnetwork	0.74
ENSG00000101333	PLCB4 PPI subnetwork	0.74

Original gene set ID	Original gene set description	Nominal P value
MP:0001807	decreased IgA level	0.74
GO:0009145	purine nucleoside triphosphate biosynthetic process	0.74
ENSG00000134480	CCNH PPI subnetwork	0.74
REACTOME_DNA_REPLICATION	REACTOME_DNA_REPLICATION	0.74
ENSG00000151067	CACNA1C PPI subnetwork	0.74
ENSG00000169429	IL8 PPI subnetwork	0.74
GO:0007286	spermatid development	0.74
MP:0001402	hypoactivity	0.74
GO:0050808	synapse organization	0.74
ENSG00000166930	MS4A5 PPI subnetwork	0.74
ENSG00000077312	SNRPA PPI subnetwork	0.75
GO:0019002	GMP binding	0.75
ENSG00000065526	SPEN PPI subnetwork	0.75
ENSG00000184672	RALYL PPI subnetwork	0.75
ENSG00000136560	TANK PPI subnetwork	0.75
MP:0004947	skin inflammation	0.75
GO:0004690	cyclic nucleotide-dependent protein kinase activity	0.75
ENSG00000198569	SLC34A3 PPI subnetwork	0.75
ENSG00000123268	ATF1 PPI subnetwork	0.75
GO:0006184	GTP catabolic process	0.75
GO:0051385	response to mineralocorticoid stimulus	0.75
ENSG00000106628	POLD2 PPI subnetwork	0.75
GO:0055065	metal ion homeostasis	0.75
MP:0000538	abnormal urinary bladder morphology	0.75
REACTOME_BIOSYNTHESIS_OF_THE_N:GLYCAN_PRECURSOR_DOLICHOL_LIPID:	REACTOME_BIOSYNTHESIS_OF_THE_N:GLYCAN_PRECURSOR_DOLICHOL_LIPID:LINK	0.75
GO:0050714	positive regulation of protein secretion	0.75
ENSG00000215320	ENSG00000215320 PPI subnetwork	0.75
ENSG00000143977	SNRPG PPI subnetwork	0.75
MP:0004986	abnormal osteoblast morphology	0.75
ENSG00000163453	IGFBP7 PPI subnetwork	0.75
MP:0011290	decreased nephron number	0.75
ENSG00000141552	ANAPC11 PPI subnetwork	0.75
GO:0006672	ceramide metabolic process	0.75
GO:0004386	helicase activity	0.75
ENSG00000014641	MDH1 PPI subnetwork	0.75
MP:0000150	abnormal rib morphology	0.75
ENSG00000102001	CACNA1F PPI subnetwork	0.75
ENSG00000162290	ENSG00000162290 PPI subnetwork	0.75
GO:0043547	positive regulation of GTPase activity	0.75
MP:0002972	abnormal cardiac muscle contractility	0.75
GO:0006040	amino sugar metabolic process	0.75
ENSG00000116062	MSH6 PPI subnetwork	0.75
ENSG00000126821	SGPP1 PPI subnetwork	0.75
MP:0000230	abnormal systemic arterial blood pressure	0.75
ENSG00000048052	HDAC9 PPI subnetwork	0.75
GO:0051588	regulation of neurotransmitter transport	0.75
GO:0001676	long-chain fatty acid metabolic process	0.75
ENSG00000187514	PTMA PPI subnetwork	0.75
ENSG00000030066	NUP160 PPI subnetwork	0.75

Original gene set ID	Original gene set description	Nominal P value
GO:0040013	negative regulation of locomotion	0.75
ENSG00000099800	TIMM13 PPI subnetwork	0.75
ENSG00000155897	ADCY8 PPI subnetwork	0.75
ENSG00000111716	LDHB PPI subnetwork	0.75
GO:0046883	regulation of hormone secretion	0.75
ENSG00000106305	AIMP2 PPI subnetwork	0.75
ENSG00000108518	PFN1 PPI subnetwork	0.75
ENSG00000087586	AURKA PPI subnetwork	0.75
GO:0040036	regulation of fibroblast growth factor receptor signaling pathway	0.75
MP:0000005	increased brown adipose tissue amount	0.75
GO:0030072	peptide hormone secretion	0.75
ENSG00000178999	AURKB PPI subnetwork	0.75
GO:0042176	regulation of protein catabolic process	0.75
GO:0003230	cardiac atrium development	0.75
MP:0003235	abnormal alisphenoid bone morphology	0.75
ENSG00000172613	RAD9A PPI subnetwork	0.75
ENSG00000165629	ATP5C1 PPI subnetwork	0.75
MP:0000455	abnormal maxilla morphology	0.75
MP:0005297	spina bifida occulta	0.75
MP:0001410	head bobbing	0.75
ENSG00000160789	LMNA PPI subnetwork	0.75
ENSG00000126602	TRAP1 PPI subnetwork	0.75
GO:0019439	aromatic compound catabolic process	0.75
GO:0002312	B cell activation involved in immune response	0.75
ENSG00000079819	EPB41L2 PPI subnetwork	0.75
GO:0007050	cell cycle arrest	0.75
GO:0044349	DNA excision	0.75
GO:0000718	nucleotide-excision repair, DNA damage removal	0.75
ENSG00000141404	GNAL PPI subnetwork	0.75
GO:0030799	regulation of cyclic nucleotide metabolic process	0.75
GO:0010742	macrophage derived foam cell differentiation	0.75
GO:0090077	foam cell differentiation	0.75
ENSG00000100142	POLR2F PPI subnetwork	0.75
GO:0008237	metallopeptidase activity	0.75
ENSG00000127337	YEATS4 PPI subnetwork	0.75
GO:0032543	mitochondrial translation	0.75
GO:0016624	oxidoreductase activity, acting on the aldehyde or oxo group of donors, disulfide as	0.75
MP:0002878	abnormal corticospinal tract morphology	0.75
ENSG00000145649	GZMA PPI subnetwork	0.75
GO:0033293	monocarboxylic acid binding	0.75
ENSG00000125970	RALY PPI subnetwork	0.75
ENSG00000168412	MTNR1A PPI subnetwork	0.75
ENSG00000070061	IKBKAP PPI subnetwork	0.75
MP:0005461	abnormal dendritic cell morphology	0.75
GO:0046209	nitric oxide metabolic process	0.75
MP:0002639	micrognathia	0.75
GO:0071496	cellular response to external stimulus	0.75
ENSG00000169031	COL4A3 PPI subnetwork	0.75
GO:0006283	transcription-coupled nucleotide-excision repair	0.75

Original gene set ID	Original gene set description	Nominal P value
ENSG00000171403	KRT9 PPI subnetwork	0.75
GO:0055002	striated muscle cell development	0.75
GO:0001158	enhancer sequence-specific DNA binding	0.75
ENSG00000128266	GNAZ PPI subnetwork	0.75
GO:0006725	cellular aromatic compound metabolic process	0.75
REACTOME_PLCG1_EVENTS_IN_ERBB2_SIGNALING	REACTOME_PLCG1_EVENTS_IN_ERBB2_SIGNALING	0.75
REACTOME_THROMBOXANE_SIGNALLING_THROUGH_TP_RECEPTOR	REACTOME_THROMBOXANE_SIGNALLING_THROUGH_TP_RECEPTOR	0.75
ENSG00000101150	TPD52L2 PPI subnetwork	0.75
KEGG_HYPERTROPHIC_CARDIOMYOPATHY_HCM	KEGG_HYPERTROPHIC_CARDIOMYOPATHY_HCM	0.75
ENSG00000147669	POLR2K PPI subnetwork	0.75
MP:0008023	abnormal styloid process morphology	0.75
GO:0014069	postsynaptic density	0.75
GO:0044327	dendritic spine head	0.75
GO:0006310	DNA recombination	0.75
REACTOME_P53:INDEPENDENT_G1S_DNA_DAMAGE_CHECKPOINT	REACTOME_P53:INDEPENDENT_G1S_DNA_DAMAGE_CHECKPOINT	0.75
REACTOME_P53:INDEPENDENT_DNA_DAMAGE_RESPONSE	REACTOME_P53:INDEPENDENT_DNA_DAMAGE_RESPONSE	0.75
REACTOME_UBIQUITIN_MEDIATED_DEGRADATION_OF_PHOSPHORYLATED_CD	REACTOME_UBIQUITIN_MEDIATED_DEGRADATION_OF_PHOSPHORYLATED_CDC25	0.75
ENSG00000149016	TUT1 PPI subnetwork	0.75
ENSG00000126767	ELK1 PPI subnetwork	0.75
ENSG00000108854	SMURF2 PPI subnetwork	0.75
ENSG00000126267	COX6B1 PPI subnetwork	0.75
ENSG00000092054	MYH7 PPI subnetwork	0.75
MP:0004131	abnormal embryonic cilium morphology	0.75
GO:0030071	regulation of mitotic metaphase/anaphase transition	0.75
GO:0004437	inositol or phosphatidylinositol phosphatase activity	0.75
MP:0003345	decreased rib number	0.75
GO:0051340	regulation of ligase activity	0.75
ENSG00000146535	GNA12 PPI subnetwork	0.75
ENSG00000105373	GLTSCR2 PPI subnetwork	0.75
REACTOME_EICOSANOID_LIGAND:BINDING_RECEPTORS	REACTOME_EICOSANOID_LIGAND:BINDING_RECEPTORS	0.76
ENSG00000170348	TMED10 PPI subnetwork	0.76
ENSG00000187953	ENSG00000187953 PPI subnetwork	0.76
ENSG00000122512	PMS2 PPI subnetwork	0.76
ENSG00000169249	ZRSR2 PPI subnetwork	0.76
ENSG00000136824	SMC2 PPI subnetwork	0.76
GO:0043567	regulation of insulin-like growth factor receptor signaling pathway	0.76
ENSG00000145736	GTF2H2 PPI subnetwork	0.76
GO:0032271	regulation of protein polymerization	0.76
ENSG00000116584	ARHGEF2 PPI subnetwork	0.76
ENSG00000168438	CDC40 PPI subnetwork	0.76
ENSG00000130024	PHF10 PPI subnetwork	0.76
GO:0060393	regulation of pathway-restricted SMAD protein phosphorylation	0.76
GO:0016866	intramolecular transferase activity	0.76
GO:0003002	regionalization	0.76
ENSG00000100028	SNRPD3 PPI subnetwork	0.76
ENSG00000169057	MECP2 PPI subnetwork	0.76
ENSG00000119335	SET PPI subnetwork	0.76
MP:0001052	abnormal muscle innervation	0.76
MP:0002206	abnormal CNS synaptic transmission	0.76

Original gene set ID	Original gene set description	Nominal P value
MP:0004021	abnormal rod electrophysiology	0.76
ENSG00000075711	DLG1 PPI subnetwork	0.76
ENSG00000197170	PSMD12 PPI subnetwork	0.76
GO:0030553	cGMP binding	0.76
ENSG00000119138	KLF9 PPI subnetwork	0.76
REACTOME_NETRIN:1_SIGNALING	REACTOME_NETRIN:1_SIGNALING	0.76
ENSG00000154429	C1orf96 PPI subnetwork	0.76
ENSG00000131149	KIAA0182 PPI subnetwork	0.76
MP:0008898	abnormal acrosome morphology	0.76
ENSG00000175895	PLEKHF2 PPI subnetwork	0.76
MP:0002752	abnormal somatic nervous system morphology	0.76
ENSG00000151065	DCP1B PPI subnetwork	0.76
GO:0005791	rough endoplasmic reticulum	0.76
ENSG00000112081	SRSF3 PPI subnetwork	0.76
GO:0045263	proton-transporting ATP synthase complex, coupling factor F(o)	0.76
GO:0006939	smooth muscle contraction	0.76
ENSG00000134574	DDB2 PPI subnetwork	0.76
ENSG00000125676	THOC2 PPI subnetwork	0.76
ENSG00000197780	TAF13 PPI subnetwork	0.76
GO:0010827	regulation of glucose transport	0.76
GO:0004385	guanylate kinase activity	0.76
MP:0010386	abnormal urinary bladder physiology	0.76
GO:0000076	DNA replication checkpoint	0.76
GO:0060441	epithelial tube branching involved in lung morphogenesis	0.76
ENSG00000113555	PCDH12 PPI subnetwork	0.76
ENSG00000111087	GLI1 PPI subnetwork	0.76
GO:0030814	regulation of cAMP metabolic process	0.76
ENSG00000137054	POLR1E PPI subnetwork	0.76
ENSG00000159461	AMFR PPI subnetwork	0.76
GO:0030817	regulation of cAMP biosynthetic process	0.76
ENSG00000177951	BET1L PPI subnetwork	0.76
ENSG00000198846	TOX PPI subnetwork	0.76
ENSG00000124097	ENSG00000124097 PPI subnetwork	0.76
ENSG00000103460	TOX3 PPI subnetwork	0.76
MP:0002882	abnormal neuron morphology	0.76
ENSG00000007168	PAFAH1B1 PPI subnetwork	0.76
GO:0050686	negative regulation of mRNA processing	0.76
GO:0006875	cellular metal ion homeostasis	0.76
ENSG00000168477	TNXB PPI subnetwork	0.76
GO:0042787	protein ubiquitination involved in ubiquitin-dependent protein catabolic proces	0.76
MP:0001688	abnormal somite development	0.76
GO:0072528	pyrimidine-containing compound biosynthetic process	0.76
MP:0003233	prolonged QT interval	0.76
REACTOME_SULFUR_AMINO_ACID_METABOLISM	REACTOME_SULFUR_AMINO_ACID_METABOLISM	0.76
GO:0002377	immunoglobulin production	0.76
GO:0006665	sphingolipid metabolic process	0.76
ENSG00000115241	PPM1G PPI subnetwork	0.76
ENSG00000064961	HMG20B PPI subnetwork	0.76
GO:0016616	oxidoreductase activity, acting on the CH-OH group of donors, NAD or NADP as acce	0.76

Original gene set ID	Original gene set description	Nominal P value
ENSG00000041357	PSMA4 PPI subnetwork	0.76
ENSG00000198910	L1CAM PPI subnetwork	0.76
GO:0015833	peptide transport	0.76
ENSG00000103671	TRIP4 PPI subnetwork	0.76
KEGG_NOD_LIKE_RECEPTOR_SIGNALING_PATHWAY	KEGG_NOD_LIKE_RECEPTOR_SIGNALING_PATHWAY	0.76
GO:0051384	response to glucocorticoid stimulus	0.76
ENSG00000153044	CENPH PPI subnetwork	0.76
ENSG00000160916	ENSG00000160916 PPI subnetwork	0.76
GO:0005743	mitochondrial inner membrane	0.76
GO:0005770	late endosome	0.76
GO:0051428	peptide hormone receptor binding	0.76
GO:0002790	peptide secretion	0.76
GO:0043161	proteasomal ubiquitin-dependent protein catabolic process	0.76
ENSG00000072682	P4HA2 PPI subnetwork	0.76
GO:0030279	negative regulation of ossification	0.76
GO:0002792	negative regulation of peptide secretion	0.76
ENSG00000110436	SLC1A2 PPI subnetwork	0.76
MP:0005517	decreased liver regeneration	0.76
ENSG00000121931	LRIF1 PPI subnetwork	0.76
ENSG00000198576	ARC PPI subnetwork	0.76
ENSG00000174231	PRPF8 PPI subnetwork	0.76
MP:0010404	ostium primum atrial septal defect	0.76
GO:0007204	elevation of cytosolic calcium ion concentration	0.76
ENSG00000128708	HAT1 PPI subnetwork	0.76
MP:0002135	abnormal kidney morphology	0.76
GO:0010576	metalloenzyme regulator activity	0.76
GO:0001510	RNA methylation	0.76
ENSG00000076053	RBM7 PPI subnetwork	0.76
GO:0031329	regulation of cellular catabolic process	0.76
ENSG00000106541	AGR2 PPI subnetwork	0.76
ENSG00000136875	PRPF4 PPI subnetwork	0.76
GO:0021782	glial cell development	0.76
GO:0051047	positive regulation of secretion	0.76
GO:0008144	drug binding	0.76
GO:0008227	G-protein coupled amine receptor activity	0.76
GO:0043392	negative regulation of DNA binding	0.76
GO:0021602	cranial nerve morphogenesis	0.76
GO:0090090	negative regulation of canonical Wnt receptor signaling pathway	0.77
GO:0006298	mismatch repair	0.77
ENSG00000133083	DCLK1 PPI subnetwork	0.77
GO:0010332	response to gamma radiation	0.77
GO:0009142	nucleoside triphosphate biosynthetic process	0.77
ENSG00000135486	HNRNPA1 PPI subnetwork	0.77
GO:0007369	gastrulation	0.77
REACTOME_M_PHASE	REACTOME_M_PHASE	0.77
ENSG00000174021	GNG5 PPI subnetwork	0.77
ENSG00000063244	U2AF2 PPI subnetwork	0.77
ENSG00000103194	USP10 PPI subnetwork	0.77
GO:0032412	regulation of ion transmembrane transporter activity	0.77

Original gene set ID	Original gene set description	Nominal P value
ENSG00000149554	CHEK1 PPI subnetwork	0.77
REACTOME_GPCR_LIGAND_BINDING	REACTOME_GPCR_LIGAND_BINDING	0.77
MP:0002812	spherocytosis	0.77
GO:0045191	regulation of isotype switching	0.77
MP:0003148	decreased cochlear coiling	0.77
MP:0010903	abnormal pulmonary alveolus wall morphology	0.77
ENSG00000115694	STK25 PPI subnetwork	0.77
ENSG00000164244	PRRC1 PPI subnetwork	0.77
ENSG00000146731	CCT6A PPI subnetwork	0.77
ENSG00000135336	ORC3 PPI subnetwork	0.77
ENSG00000122122	SASH3 PPI subnetwork	0.77
ENSG00000174718	C12orf35 PPI subnetwork	0.77
GO:0044309	neuron spine	0.77
GO:0043197	dendritic spine	0.77
MP:0008482	decreased spleen germinal center number	0.77
ENSG00000114315	HES1 PPI subnetwork	0.77
GO:0060216	definitive hemopoiesis	0.77
MP:0005202	lethargy	0.77
GO:0046933	hydrogen ion transporting ATP synthase activity, rotational mechanism	0.77
GO:0044456	synapse part	0.77
ENSG00000140350	ANP32A PPI subnetwork	0.77
GO:0050661	NADP binding	0.77
GO:0005109	frizzled binding	0.77
ENSG00000101843	PSMD10 PPI subnetwork	0.77
GO:0021761	limbic system development	0.77
ENSG00000109332	UBE2D3 PPI subnetwork	0.77
REACTOME_NEURONAL_SYSTEM	REACTOME_NEURONAL_SYSTEM	0.77
ENSG00000183763	TRAIP PPI subnetwork	0.77
REACTOME_PI3K_EVENTS_IN_ERBB4_SIGNALING	REACTOME_PI3K_EVENTS_IN_ERBB4_SIGNALING	0.77
GO:0006171	cAMP biosynthetic process	0.77
GO:0046879	hormone secretion	0.77
GO:0016471	vacuolar proton-transporting V-type ATPase complex	0.77
MP:0002919	enhanced paired-pulse facilitation	0.77
GO:0045335	phagocytic vesicle	0.77
MP:0000521	abnormal kidney cortex morphology	0.77
ENSG00000147439	BIN3 PPI subnetwork	0.77
MP:0001890	anencephaly	0.77
GO:0009894	regulation of catabolic process	0.77
REACTOME_TELOMERE_MAINTENANCE	REACTOME_TELOMERE_MAINTENANCE	0.77
ENSG00000143379	SETDB1 PPI subnetwork	0.77
GO:0007368	determination of left/right symmetry	0.77
GO:0060992	response to fungicide	0.77
ENSG00000163960	UBXN7 PPI subnetwork	0.77
REACTOME_FANCONI_ANEMIA_PATHWAY	REACTOME_FANCONI_ANEMIA_PATHWAY	0.77
MP:0000644	dextrocardia	0.77
MP:0001044	abnormal enteric nervous system morphology	0.77
GO:0032589	neuron projection membrane	0.77
ENSG00000180209	MYLPF PPI subnetwork	0.77
ENSG00000087274	ADD1 PPI subnetwork	0.77

Original gene set ID	Original gene set description	Nominal P value
MP:0003702	abnormal chromosome morphology	0.77
REACTOME_CLASS_A1_RHODOPSIN:LIKE_RECEPTORS	REACTOME_CLASS_A1_RHODOPSIN:LIKE_RECEPTORS	0.77
ENSG00000116459	ATP5F1 PPI subnetwork	0.77
GO:0001533	cornified envelope	0.77
MP:0002998	abnormal bone remodeling	0.77
MP:0002682	decreased mature ovarian follicle number	0.77
ENSG00000205937	RNPS1 PPI subnetwork	0.77
ENSG00000101444	AHCY PPI subnetwork	0.77
GO:0030017	sarcomere	0.77
ENSG00000164109	MAD2L1 PPI subnetwork	0.77
GO:0010389	regulation of G2/M transition of mitotic cell cycle	0.77
ENSG00000108528	SLC25A11 PPI subnetwork	0.77
ENSG00000196284	SUPT3H PPI subnetwork	0.77
REACTOME_TANDEM_PORE_DOMAIN_POTASSIUM_CHANNELS	REACTOME_TANDEM_PORE_DOMAIN_POTASSIUM_CHANNELS	0.77
GO:0017091	AU-rich element binding	0.77
ENSG00000127928	GNGT1 PPI subnetwork	0.77
ENSG00000011007	TCEB3 PPI subnetwork	0.77
ENSG00000128609	NDUFA5 PPI subnetwork	0.77
ENSG00000182180	MRPS16 PPI subnetwork	0.77
GO:0006699	bile acid biosynthetic process	0.77
GO:0009954	proximal/distal pattern formation	0.77
REACTOME_PI3K_EVENTS_IN_ERBB2_SIGNALING	REACTOME_PI3K_EVENTS_IN_ERBB2_SIGNALING	0.77
ENSG00000136273	HUS1 PPI subnetwork	0.77
ENSG00000042832	TG PPI subnetwork	0.77
REACTOME_POLYMERASE_SWITCHING_ON_THE_C:STRAND_OF_THE_TELOMER	REACTOME_POLYMERASE_SWITCHING_ON_THE_C:STRAND_OF_THE_TELOMERE	0.77
REACTOME_LEADING_STRAND_SYNTHESIS	REACTOME_LEADING_STRAND_SYNTHESIS	0.77
REACTOME_POLYMERASE_SWITCHING	REACTOME_POLYMERASE_SWITCHING	0.77
MP:0010856	dilated respiratory conducting tubes	0.77
KEGG_EPITHELIAL_CELL_SIGNALING_IN_HELICOBACTER_PYLORI_INFECTION	KEGG_EPITHELIAL_CELL_SIGNALING_IN_HELICOBACTER_PYLORI_INFECTION	0.77
GO:0008278	cohesin complex	0.77
ENSG00000213611	ENSG00000213611 PPI subnetwork	0.77
ENSG00000177889	UBE2N PPI subnetwork	0.77
REACTOME_PLC_BETA_MEDIATED_EVENTS	REACTOME_PLC_BETA_MEDIATED_EVENTS	0.77
MP:0002016	ovary cysts	0.77
ENSG00000147869	CER1 PPI subnetwork	0.77
ENSG00000108294	PSMB3 PPI subnetwork	0.77
GO:0042742	defense response to bacterium	0.77
GO:0008093	cytoskeletal adaptor activity	0.77
REACTOME_DUAL_INCISION_REACTION_IN_GG:NER	REACTOME_DUAL_INCISION_REACTION_IN_GG:NER	0.77
REACTOME_FORMATION_OF_INCISION_COMPLEX_IN_GG:NER	REACTOME_FORMATION_OF_INCISION_COMPLEX_IN_GG:NER	0.77
MP:0000757	herniated abdominal wall	0.77
ENSG00000164270	HTR4 PPI subnetwork	0.77
GO:0002293	alpha-beta T cell differentiation involved in immune response	0.77
GO:0002287	alpha-beta T cell activation involved in immune response	0.77
ENSG00000170558	CDH2 PPI subnetwork	0.77
ENSG00000117592	PRDX6 PPI subnetwork	0.77
ENSG00000197818	SLC9A8 PPI subnetwork	0.77
MP:0001636	irregular heartbeat	0.78
ENSG00000163082	SGPP2 PPI subnetwork	0.78

Original gene set ID	Original gene set description	Nominal P value
MP:0001071	abnormal facial nerve morphology	0.78
ENSG00000002016	RAD52 PPI subnetwork	0.78
ENSG00000120253	NUP43 PPI subnetwork	0.78
GO:0051899	membrane depolarization	0.78
GO:0021766	hippocampus development	0.78
GO:0006836	neurotransmitter transport	0.78
GO:0042384	cilium assembly	0.78
ENSG00000179915	NRXN1 PPI subnetwork	0.78
GO:0010743	regulation of macrophage derived foam cell differentiatior	0.78
MP:0005431	decreased oocyte number	0.78
ENSG00000115233	PSMD14 PPI subnetwork	0.78
ENSG00000108272	DHRS11 PPI subnetwork	0.78
MP:0003896	prolonged PR interval	0.78
ENSG00000104852	SNRNP70 PPI subnetwork	0.78
ENSG00000067334	DNTTIP2 PPI subnetwork	0.78
MP:0000534	abnormal ureter morphology	0.78
MP:0008146	asymmetric rib-sternum attachment	0.78
MP:0000729	abnormal myogenesis	0.78
GO:0048167	regulation of synaptic plasticity	0.78
ENSG00000183023	SLC8A1 PPI subnetwork	0.78
ENSG00000153162	BMP6 PPI subnetwork	0.78
GO:0046039	GTP metabolic process	0.78
GO:0005504	fatty acid binding	0.78
ENSG00000156970	BUB1B PPI subnetwork	0.78
GO:0031966	mitochondrial membrane	0.78
ENSG00000111358	GTF2H3 PPI subnetwork	0.78
GO:0003207	cardiac chamber formation	0.78
GO:0000077	DNA damage checkpoint	0.78
ENSG00000114026	OGG1 PPI subnetwork	0.78
REACTOME_INHIBITION__OF_VOLTAGE_GATED_CA2_CHANNELS_VIA_GBETAG/	REACTOME_INHIBITION__OF_VOLTAGE_GATED_CA2_CHANNELS_VIA_GBETAGAMI	0.78
REACTOME_ACTIVATION_OF_G_PROTEIN_GATED_POTASSIUM_CHANNELS	REACTOME_ACTIVATION_OF_G_PROTEIN_GATED_POTASSIUM_CHANNELS	0.78
REACTOME_G_PROTEIN_GATED_POTASSIUM_CHANNELS	REACTOME_G_PROTEIN_GATED_POTASSIUM_CHANNELS	0.78
ENSG00000186852	ENSG00000186852 PPI subnetwork	0.78
GO:0007409	axonogenesis	0.78
GO:0019003	GDP binding	0.78
REACTOME_SEROTONIN_RECEPTORS	REACTOME_SEROTONIN_RECEPTORS	0.78
MP:0001093	small trigeminal ganglion	0.78
ENSG00000131876	SNRPA1 PPI subnetwork	0.78
MP:0008267	abnormal hippocampus CA3 region morphology	0.78
GO:0006595	polyamine metabolic process	0.78
GO:0007173	epidermal growth factor receptor signaling pathway	0.78
ENSG00000112559	MDFI PPI subnetwork	0.78
MP:0003657	abnormal erythrocyte osmotic lysis	0.78
GO:0090278	negative regulation of peptide hormone secretior	0.78
ENSG00000067369	TP53BP1 PPI subnetwork	0.78
MP:0002286	cryptorchism	0.78
ENSG00000181218	HIST3H2A PPI subnetwork	0.78
ENSG00000212868	ENSG00000212868 PPI subnetwork	0.78
ENSG00000198727	MT-CYB PPI subnetwork	0.78

Original gene set ID	Original gene set description	Nominal P value
ENSG00000166508	MCM7 PPI subnetwork	0.78
ENSG00000100412	ACO2 PPI subnetwork	0.78
GO:0045202	synapse	0.78
REACTOME_JNK_C:JUN_KINASES_PHOSPHORYLATION_AND__ACTIVATION_MEI	REACTOME_JNK_C:JUN_KINASES_PHOSPHORYLATION_AND__ACTIVATION_MEDIAT	0.78
KEGG_NICOTINATE_AND_NICOTINAMIDE_METABOLISM	KEGG_NICOTINATE_AND_NICOTINAMIDE_METABOLISM	0.78
ENSG00000075651	PLD1 PPI subnetwork	0.78
ENSG00000171848	RRM2 PPI subnetwork	0.78
GO:0030501	positive regulation of bone mineralization	0.78
MP:0006301	abnormal mesenchyme morphology	0.78
GO:0006684	sphingomyelin metabolic process	0.78
ENSG00000135823	STX6 PPI subnetwork	0.78
GO:0022898	regulation of transmembrane transporter activity	0.78
GO:0006397	mRNA processing	0.78
MP:0001096	abnormal glossopharyngeal ganglion morphology	0.78
ENSG00000168243	GNG4 PPI subnetwork	0.78
REACTOME_G2M_CHECKPOINTS	REACTOME_G2M_CHECKPOINTS	0.78
MP:0005637	abnormal iron homeostasis	0.78
ENSG00000141446	ESCO1 PPI subnetwork	0.78
ENSG00000183454	GRIN2A PPI subnetwork	0.78
GO:0019228	regulation of action potential in neuron	0.78
ENSG00000059769	DNAJC25 PPI subnetwork	0.78
ENSG00000162419	GMEB1 PPI subnetwork	0.78
GO:0009295	nucleoid	0.78
MP:0002229	neurodegeneration	0.78
ENSG00000132872	SYT4 PPI subnetwork	0.78
MP:0001525	impaired balance	0.78
REACTOME_PROTEOLYTIC_CLEAVAGE_OF_SNARE_COMPLEX_PROTEINS	REACTOME_PROTEOLYTIC_CLEAVAGE_OF_SNARE_COMPLEX_PROTEINS	0.78
REACTOME_REGULATION_OF_INSULIN_SECRETION_BY_ACETYLCHOLINE	REACTOME_REGULATION_OF_INSULIN_SECRETION_BY_ACETYLCHOLINE	0.78
REACTOME_SIGNALING_BY_ERBB2	REACTOME_SIGNALING_BY_ERBB2	0.78
ENSG00000213639	PPP1CB PPI subnetwork	0.78
ENSG00000117399	CDC20 PPI subnetwork	0.78
ENSG00000047315	POLR2B PPI subnetwork	0.78
KEGG_TYPE_I_DIABETES_MELLITUS	KEGG_TYPE_I_DIABETES_MELLITUS	0.78
ENSG00000005194	CIAPIN1 PPI subnetwork	0.78
MP:0004158	right aortic arch	0.78
MP:0003635	abnormal synaptic transmission	0.78
ENSG00000198788	MUC2 PPI subnetwork	0.78
ENSG00000198932	GPRASP1 PPI subnetwork	0.78
GO:0002292	T cell differentiation involved in immune response	0.78
ENSG00000125845	BMP2 PPI subnetwork	0.78
ENSG00000136807	CDK9 PPI subnetwork	0.78
MP:0006074	abnormal retinal rod bipolar cell morphology	0.78
REACTOME_ORGANIC_CATIONANIONZWITTERION_TRANSPORT	REACTOME_ORGANIC_CATIONANIONZWITTERION_TRANSPORT	0.78
GO:0005326	neurotransmitter transporter activity	0.78
GO:0010092	specification of organ identity	0.79
ENSG00000152822	GRM1 PPI subnetwork	0.79
ENSG00000071894	CPSF1 PPI subnetwork	0.79
MP:0000269	abnormal heart looping	0.79
REACTOME_BETA_DEFENSINS	REACTOME_BETA_DEFENSINS	0.79

Original gene set ID	Original gene set description	Nominal P value
GO:0070301	cellular response to hydrogen peroxide	0.79
ENSG00000213465	ARL2 PPI subnetwork	0.79
ENSG00000212874	ENSG00000212874 PPI subnetwork	0.79
ENSG00000198712	MT-CO2 PPI subnetwork	0.79
MP:0005329	abnormal myocardium layer morphology	0.79
ENSG00000196092	PAX5 PPI subnetwork	0.79
MP:0003232	abnormal forebrain development	0.79
GO:0019321	pentose metabolic process	0.79
MP:0001596	hypotension	0.79
ENSG00000182621	PLCB1 PPI subnetwork	0.79
GO:0030195	negative regulation of blood coagulation	0.79
ENSG00000173575	CHD2 PPI subnetwork	0.79
ENSG00000162704	ARPC5 PPI subnetwork	0.79
MP:0008788	abnormal fetal cardiomyocyte morphology	0.79
GO:0055080	cation homeostasis	0.79
ENSG00000164330	EBF1 PPI subnetwork	0.79
ENSG00000058272	PPP1R12A PPI subnetwork	0.79
ENSG00000142655	PEX14 PPI subnetwork	0.79
GO:0031576	G2/M transition checkpoint	0.79
GO:0008173	RNA methyltransferase activity	0.79
GO:0016849	phosphorus-oxygen lyase activity	0.79
GO:0016831	carboxy-lyase activity	0.79
ENSG00000100911	PSME2 PPI subnetwork	0.79
ENSG00000167863	ATP5H PPI subnetwork	0.79
MP:0001522	impaired swimming	0.79
GO:0001653	peptide receptor activity	0.79
MP:0000955	abnormal spinal cord morphology	0.79
GO:0031163	metallo-sulfur cluster assembly	0.79
GO:0016226	iron-sulfur cluster assembly	0.79
MP:0004252	abnormal direction of heart looping	0.79
ENSG00000181090	EHMT1 PPI subnetwork	0.79
REACTOME_ASSEMBLY_OF_THE_PRE:REPLICATIVE_COMPLEX	REACTOME_ASSEMBLY_OF_THE_PRE:REPLICATIVE_COMPLEX	0.79
REACTOME_APCCCDC20_MEDIATED_DEGRADATION_OF_MITOTIC_PROTEINS	REACTOME_APCCCDC20_MEDIATED_DEGRADATION_OF_MITOTIC_PROTEINS	0.79
GO:0016769	transferase activity, transferring nitrogenous groups	0.79
GO:0001935	endothelial cell proliferation	0.79
MP:0002988	decreased urine osmolality	0.79
ENSG00000100764	PSMC1 PPI subnetwork	0.79
ENSG00000165525	NEMF PPI subnetwork	0.79
MP:0005584	abnormal enzyme/coenzyme activity	0.79
MP:0001958	emphysema	0.79
GO:0009060	aerobic respiration	0.79
GO:0051707	response to other organism	0.79
GO:0051969	regulation of transmission of nerve impulse	0.79
ENSG00000177189	RPS6KA3 PPI subnetwork	0.79
MP:0003222	increased cardiomyocyte apoptosis	0.79
GO:0004518	nuclease activity	0.79
ENSG00000100567	PSMA3 PPI subnetwork	0.79
GO:0046164	alcohol catabolic process	0.79
REACTOME_DEPOLARIZATION_OF_THE_PRESYNAPTIC_TERMINAL_TRIGGERS_T	REACTOME_DEPOLARIZATION_OF_THE_PRESYNAPTIC_TERMINAL_TRIGGERS_THE_	0.79

Original gene set ID	Original gene set description	Nominal P value
ENSG00000069329	VPS35 PPI subnetwork	0.79
MP:0000937	abnormal motor neuron morphology	0.79
REACTOME_ACTIVATED_AMPK_STIMULATES_FATTY:ACID_OXIDATION_IN_MUS	REACTOME_ACTIVATED_AMPK_STIMULATES_FATTY:ACID_OXIDATION_IN_MUSCLE	0.79
ENSG00000124535	WRNIP1 PPI subnetwork	0.79
ENSG00000134371	CDC73 PPI subnetwork	0.79
ENSG00000085872	CHERP PPI subnetwork	0.79
GO:0046580	negative regulation of Ras protein signal transductior	0.79
MP:0001380	reduced male mating frequency	0.79
ENSG00000124333	VAMP7 PPI subnetwork	0.79
MP:0003313	abnormal locomotor activation	0.79
MP:0002183	gliosis	0.79
ENSG00000168522	FNTA PPI subnetwork	0.79
GO:0045669	positive regulation of osteoblast differentiatior	0.79
GO:0010498	proteasomal protein catabolic process	0.79
KEGG_FATTY_ACID_METABOLISM	KEGG_FATTY_ACID_METABOLISM	0.79
REACTOME_LYSOSOME_VESICLE_BIOGENESIS	REACTOME_LYSOSOME_VESICLE_BIOGENESIS	0.79
GO:0009084	glutamine family amino acid biosynthetic process	0.79
ENSG00000117758	STX12 PPI subnetwork	0.79
GO:0021510	spinal cord development	0.79
ENSG00000178950	GAK PPI subnetwork	0.79
ENSG00000104835	FBXO17 PPI subnetwork	0.79
GO:0004745	retinol dehydrogenase activity	0.79
GO:0051537	2 iron, 2 sulfur cluster binding	0.79
ENSG00000125450	NUP85 PPI subnetwork	0.79
GO:0009201	ribonucleoside triphosphate biosynthetic process	0.79
ENSG00000104884	ERCC2 PPI subnetwork	0.79
ENSG00000003756	RBM5 PPI subnetwork	0.79
ENSG00000127922	SHFM1 PPI subnetwork	0.79
ENSG00000134899	ERCC5 PPI subnetwork	0.79
MP:0000297	abnormal atrioventricular cushion morphology	0.79
GO:0016607	nuclear speck	0.79
MP:0008221	abnormal hippocampal commissure morphology	0.79
ENSG00000173163	COMMD1 PPI subnetwork	0.79
ENSG00000139970	RTN1 PPI subnetwork	0.79
ENSG00000189283	FHIT PPI subnetwork	0.79
GO:0010578	regulation of adenylate cyclase activity involved in G-protein coupled receptor signa	0.79
GO:0007189	adenylate cyclase-activating G-protein coupled receptor signaling pathway	0.79
GO:0010579	positive regulation of adenylate cyclase activity involved in G-protein coupled recep	0.79
GO:0007156	homophilic cell adhesion	0.79
ENSG00000101158	TH1L PPI subnetwork	0.79
GO:0030003	cellular cation homeostasis	0.79
ENSG00000143870	PDIA6 PPI subnetwork	0.79
REACTOME_SIGNALING_BY_BMP	REACTOME_SIGNALING_BY_BMP	0.79
GO:0090087	regulation of peptide transport	0.79
GO:0002791	regulation of peptide secretion	0.79
ENSG00000035862	TIMP2 PPI subnetwork	0.79
GO:0007224	smoothened signaling pathway	0.79
GO:0042133	neurotransmitter metabolic process	0.79
GO:0000460	maturation of 5.8S rRNA	0.79

Original gene set ID	Original gene set description	Nominal P value
ENSG00000183207	RUVBL2 PPI subnetwork	0.79
MP:0005404	abnormal axon morphology	0.79
ENSG00000068323	TFE3 PPI subnetwork	0.79
GO:0051438	regulation of ubiquitin-protein ligase activity	0.79
ENSG00000117533	VAMP4 PPI subnetwork	0.79
GO:0005681	spliceosomal complex	0.79
ENSG00000140829	DHX38 PPI subnetwork	0.79
GO:0051052	regulation of DNA metabolic process	0.8
GO:0008277	regulation of G-protein coupled receptor protein signaling pathway	0.8
MP:0004966	abnormal inner cell mass proliferation	0.8
MP:0003008	enhanced long term potentiation	0.8
ENSG00000175054	ATR PPI subnetwork	0.8
ENSG00000114107	CEP70 PPI subnetwork	0.8
GO:0008430	selenium binding	0.8
ENSG00000113522	RAD50 PPI subnetwork	0.8
ENSG00000168061	SAC3D1 PPI subnetwork	0.8
ENSG00000128534	NAA38 PPI subnetwork	0.8
ENSG00000168067	MAP4K2 PPI subnetwork	0.8
GO:0031649	heat generation	0.8
ENSG00000075188	NUP37 PPI subnetwork	0.8
GO:0005865	striated muscle thin filament	0.8
ENSG00000111788	ENSG00000111788 PPI subnetwork	0.8
ENSG00000214826	ENSG00000214826 PPI subnetwork	0.8
ENSG00000137834	SMAD6 PPI subnetwork	0.8
ENSG00000172175	MALT1 PPI subnetwork	0.8
MP:0002675	asthenozoospermia	0.8
REACTOME_MITOTIC_M:MG1_PHASES	REACTOME_MITOTIC_M:MG1_PHASES	0.8
REACTOME_ACTIVATION_OF_APCC_AND_APCCCDC20_MEDIATED_DEGRADATI	REACTOME_ACTIVATION_OF_APCC_AND_APCCCDC20_MEDIATED_DEGRADATION_	0.8
ENSG00000159720	ATP6V0D1 PPI subnetwork	0.8
REACTOME_NEF:MEDIATES_DOWN_MODULATION_OF_CELL_SURFACE_RECEPT	REACTOME_NEF:MEDIATES_DOWN_MODULATION_OF_CELL_SURFACE_RECEPTORS	0.8
GO:0000956	nuclear-transcribed mRNA catabolic process	0.8
MP:0006316	increased urine sodium level	0.8
ENSG00000140262	TCF12 PPI subnetwork	0.8
GO:0004691	cAMP-dependent protein kinase activity	0.8
GO:0031396	regulation of protein ubiquitination	0.8
ENSG00000189091	SF3B3 PPI subnetwork	0.8
REACTOME_GRB2_EVENTS_IN_ERBB2_SIGNALING	REACTOME_GRB2_EVENTS_IN_ERBB2_SIGNALING	0.8
MP:0000029	abnormal malleus morphology	0.8
GO:0044106	cellular amine metabolic process	0.8
ENSG00000151461	UPF2 PPI subnetwork	0.8
GO:0032561	guanyl ribonucleotide binding	0.8
GO:0019001	guanyl nucleotide binding	0.8
GO:0006977	DNA damage response, signal transduction by p53 class mediator resulting in cell cy	0.8
GO:0072401	signal transduction involved in DNA integrity checkpoint	0.8
GO:0072431	signal transduction involved in mitotic cell cycle G1/S transition DNA damage checkp	0.8
GO:0072413	signal transduction involved in mitotic cell cycle checkpoint	0.8
GO:0072422	signal transduction involved in DNA damage checkpoint	0.8
GO:0072474	signal transduction involved in mitotic cell cycle G1/S checkpoint	0.8
ENSG00000183558	HIST2H2AA3 PPI subnetwork	0.8

Original gene set ID	Original gene set description	Nominal P value
ENSG00000203812	HIST2H2AA4 PPI subnetwork	0.8
ENSG00000128692	ENSG00000128692 PPI subnetwork	0.8
GO:0007411	axon guidance	0.8
ENSG00000072415	MPP5 PPI subnetwork	0.8
MP:0000936	small telencephalic vesicles	0.8
MP:0000611	jaundice	0.8
GO:0043254	regulation of protein complex assembly	0.8
ENSG00000213246	SUPT4H1 PPI subnetwork	0.8
ENSG00000100056	DGCR14 PPI subnetwork	0.8
GO:0031672	A band	0.8
MP:0000286	abnormal mitral valve morphology	0.8
GO:0002700	regulation of production of molecular mediator of immune response	0.8
GO:0007274	neuromuscular synaptic transmission	0.8
KEGG_SPLICEOSOME	KEGG_SPLICEOSOME	0.8
GO:0016667	oxidoreductase activity, acting on a sulfur group of donors	0.8
MP:0006380	abnormal spermatid morphology	0.8
MP:0003141	cardiac fibrosis	0.8
GO:0048846	axon extension involved in axon guidance	0.8
ENSG00000136813	KIAA0368 PPI subnetwork	0.8
MP:0002777	absent ovarian follicles	0.8
GO:0030510	regulation of BMP signaling pathway	0.8
GO:0008536	Ran GTPase binding	0.8
ENSG00000130779	CLIP1 PPI subnetwork	0.8
GO:0071377	cellular response to glucagon stimulus	0.8
ENSG00000135916	ITM2C PPI subnetwork	0.8
ENSG00000215476	ENSG00000215476 PPI subnetwork	0.8
ENSG00000206476	ENSG00000206476 PPI subnetwork	0.8
ENSG00000213780	GTF2H4 PPI subnetwork	0.8
ENSG00000122218	COPA PPI subnetwork	0.8
MP:0001932	abnormal spermiogenesis	0.8
MP:0009757	impaired behavioral response to morphine	0.8
ENSG00000157483	MYO1E PPI subnetwork	0.8
GO:0070169	positive regulation of biomineral tissue development	0.8
GO:0007623	circadian rhythm	0.8
GO:0032331	negative regulation of chondrocyte differentiation	0.8
MP:0000278	abnormal myocardial fiber morphology	0.8
ENSG00000137992	DBT PPI subnetwork	0.8
GO:0044062	regulation of excretion	0.8
GO:0050664	oxidoreductase activity, acting on NADH or NADPH, oxygen as acceptor	0.8
GO:0060324	face development	0.8
ENSG00000172315	TP53RK PPI subnetwork	0.8
GO:0033178	proton-transporting two-sector ATPase complex, catalytic domain	0.8
ENSG00000109107	ALDOC PPI subnetwork	0.8
GO:0006353	transcription termination, DNA-dependent	0.8
GO:0060479	lung cell differentiation	0.8
MP:0010090	increased circulating creatine kinase level	0.8
GO:0015036	disulfide oxidoreductase activity	0.8
GO:0060795	cell fate commitment involved in formation of primary germ layer	0.8
REACTOME_PEPTIDE_LIGAND:BINDING_RECEPTORS	REACTOME_PEPTIDE_LIGAND:BINDING_RECEPTORS	0.8

Original gene set ID	Original gene set description	Nominal P value
GO:0046633	alpha-beta T cell proliferation	0.8
GO:0008556	potassium-transporting ATPase activity	0.8
GO:0070663	regulation of leukocyte proliferation	0.8
ENSG00000152208	GRID2 PPI subnetwork	0.8
GO:0002053	positive regulation of mesenchymal cell proliferation	0.8
ENSG00000065609	SNAP91 PPI subnetwork	0.8
GO:0046676	negative regulation of insulin secretion	0.8
ENSG00000143228	NUF2 PPI subnetwork	0.8
ENSG00000170606	HSPA4 PPI subnetwork	0.8
ENSG00000108175	ZMIZ1 PPI subnetwork	0.8
ENSG00000172201	ID4 PPI subnetwork	0.8
GO:0051953	negative regulation of amine transport	0.8
GO:0042147	retrograde transport, endosome to Golgi	0.81
ENSG00000172572	PDE3A PPI subnetwork	0.81
ENSG00000198478	SH3BGRL2 PPI subnetwork	0.81
GO:0050819	negative regulation of coagulation	0.81
MP:0003862	decreased aggression towards males	0.81
ENSG00000102981	PARD6A PPI subnetwork	0.81
MP:0010264	increased hepatoma incidence	0.81
GO:0003211	cardiac ventricle formation	0.81
ENSG00000095002	MSH2 PPI subnetwork	0.81
REACTOME_TRANSMISSION_ACROSS_CHEMICAL_SYNAPSES	REACTOME_TRANSMISSION_ACROSS_CHEMICAL_SYNAPSES	0.81
ENSG00000170296	GABARAP PPI subnetwork	0.81
GO:0008528	G-protein coupled peptide receptor activity	0.81
GO:0006913	nucleocytoplasmic transport	0.81
GO:0031644	regulation of neurological system process	0.81
ENSG00000174243	DDX23 PPI subnetwork	0.81
GO:0032467	positive regulation of cytokinesis	0.81
ENSG00000169621	APLF PPI subnetwork	0.81
GO:0030816	positive regulation of cAMP metabolic process	0.81
GO:0030819	positive regulation of cAMP biosynthetic process	0.81
ENSG00000166579	NDEL1 PPI subnetwork	0.81
REACTOME_NCAM1_INTERACTIONS	REACTOME_NCAM1_INTERACTIONS	0.81
GO:0051046	regulation of secretion	0.81
ENSG00000106299	WASL PPI subnetwork	0.81
ENSG00000206505	HLA-A PPI subnetwork	0.81
GO:0097061	dendritic spine organization	0.81
GO:0060997	dendritic spine morphogenesis	0.81
ENSG00000129514	FOXA1 PPI subnetwork	0.81
ENSG00000211895	ENSG00000211895 PPI subnetwork	0.81
ENSG00000180353	HCLS1 PPI subnetwork	0.81
ENSG00000144231	POLR2D PPI subnetwork	0.81
MP:0001433	polyphagia	0.81
MP:0002106	abnormal muscle physiology	0.81
GO:0070717	poly-purine tract binding	0.81
ENSG00000099995	SF3A1 PPI subnetwork	0.81
ENSG00000163132	MSX1 PPI subnetwork	0.81
ENSG00000060069	CTDP1 PPI subnetwork	0.81
GO:0044243	multicellular organismal catabolic process	0.81

Original gene set ID	Original gene set description	Nominal P value
MP:0001544	abnormal cardiovascular system physiology	0.81
ENSG00000163041	H3F3A PPI subnetwork	0.81
ENSG00000132475	H3F3B PPI subnetwork	0.81
ENSG00000196285	ENSG00000196285 PPI subnetwork	0.81
ENSG00000079739	PGM1 PPI subnetwork	0.81
GO:0018209	peptidyl-serine modification	0.81
MP:0005642	decreased mean corpuscular hemoglobin concentration	0.81
MP:0001473	reduced long term potentiation	0.81
ENSG00000154582	TCEB1 PPI subnetwork	0.81
ENSG00000186318	BACE1 PPI subnetwork	0.81
GO:0009309	amine biosynthetic process	0.81
REACTOME_GABA_A_RECEPTOR_ACTIVATION	REACTOME_GABA_A_RECEPTOR_ACTIVATION	0.81
ENSG00000108840	HDAC5 PPI subnetwork	0.81
GO:0060968	regulation of gene silencing	0.81
GO:0051351	positive regulation of ligase activity	0.81
GO:0046640	regulation of alpha-beta T cell proliferation	0.81
ENSG00000064995	TAF11 PPI subnetwork	0.81
ENSG00000185049	WHSC2 PPI subnetwork	0.81
GO:0003012	muscle system process	0.81
GO:0005776	autophagic vacuole	0.81
GO:0070507	regulation of microtubule cytoskeleton organization	0.81
MP:0003130	anal atresia	0.81
ENSG00000070950	RAD18 PPI subnetwork	0.81
GO:0050662	coenzyme binding	0.81
GO:0030514	negative regulation of BMP signaling pathway	0.81
ENSG00000175166	PSMD2 PPI subnetwork	0.81
GO:0072207	metanephric epithelium development	0.81
GO:0045494	photoreceptor cell maintenance	0.81
ENSG00000105894	PTN PPI subnetwork	0.81
MP:0000690	absent spleen	0.81
ENSG00000141570	CBX8 PPI subnetwork	0.81
GO:0051606	detection of stimulus	0.81
GO:0050769	positive regulation of neurogenesis	0.81
GO:0019825	oxygen binding	0.81
ENSG00000130713	EXOSC2 PPI subnetwork	0.81
ENSG00000072501	SMC1A PPI subnetwork	0.81
REACTOME_CA:DEPENDENT_EVENTS	REACTOME_CA:DEPENDENT_EVENTS	0.81
MP:0004145	abnormal muscle electrophysiology	0.81
ENSG00000174622	ENSG00000174622 PPI subnetwork	0.81
GO:0043954	cellular component maintenance	0.81
GO:0009065	glutamine family amino acid catabolic process	0.81
ENSG00000100084	HIRA PPI subnetwork	0.81
ENSG00000153201	RANBP2 PPI subnetwork	0.81
MP:0008484	decreased spleen germinal center size	0.81
GO:0060740	prostate gland epithelium morphogenesis	0.81
ENSG00000134640	MTNR1B PPI subnetwork	0.81
MP:0005264	glomerulosclerosis	0.81
GO:0090276	regulation of peptide hormone secretion	0.81
GO:0005876	spindle microtubule	0.81

Original gene set ID	Original gene set description	Nominal P value
ENSG00000075856	SART3 PPI subnetwork	0.81
MP:0000953	abnormal oligodendrocyte morphology	0.81
MP:0003920	abnormal heart right ventricle morphology	0.81
ENSG00000166501	PRKCB PPI subnetwork	0.82
GO:0030670	phagocytic vesicle membrane	0.82
ENSG00000162923	WDR26 PPI subnetwork	0.82
ENSG00000138293	NCOA4 PPI subnetwork	0.82
KEGG_GLYCINE_SERINE_AND_THREONINE_METABOLISM	KEGG_GLYCINE_SERINE_AND_THREONINE_METABOLISM	0.82
GO:0006826	iron ion transport	0.82
GO:0022010	central nervous system myelination	0.82
GO:0032291	axon ensheathment in central nervous system	0.82
MP:0005620	abnormal muscle contractility	0.82
GO:0002042	cell migration involved in sprouting angiogenesis	0.82
GO:0005048	signal sequence binding	0.82
GO:0071248	cellular response to metal ion	0.82
ENSG00000078668	VDAC3 PPI subnetwork	0.82
ENSG00000108691	CCL2 PPI subnetwork	0.82
ENSG00000104177	MYEF2 PPI subnetwork	0.82
ENSG00000186810	CXCR3 PPI subnetwork	0.82
MP:0000153	rib bifurcation	0.82
ENSG00000155959	VBP1 PPI subnetwork	0.82
ENSG00000102683	SGCG PPI subnetwork	0.82
ENSG00000121552	CSTA PPI subnetwork	0.82
ENSG00000126215	XRCC3 PPI subnetwork	0.82
ENSG00000145494	NDUFS6 PPI subnetwork	0.82
MP:0006032	abnormal ureteric bud morphology	0.82
ENSG00000104915	STX10 PPI subnetwork	0.82
MP:0005599	increased cardiac muscle contractility	0.82
ENSG00000206328	ENSG00000206328 PPI subnetwork	0.82
ENSG00000204490	TNF PPI subnetwork	0.82
ENSG00000206439	TNF PPI subnetwork	0.82
GO:0007610	behavior	0.82
REACTOME_RNA_POLYMERASE_II_PRE:TRANSCRIPTION_EVENTS	REACTOME_RNA_POLYMERASE_II_PRE:TRANSCRIPTION_EVENTS	0.82
REACTOME_MITOTIC_PROMETAPHASE	REACTOME_MITOTIC_PROMETAPHASE	0.82
MP:0003161	absent lateral semicircular canal	0.82
GO:0032391	photoreceptor connecting cilium	0.82
ENSG00000137815	RTF1 PPI subnetwork	0.82
ENSG00000154174	TOMM70A PPI subnetwork	0.82
ENSG00000170260	ZNF212 PPI subnetwork	0.82
GO:0006406	mRNA export from nucleus	0.82
GO:0015802	basic amino acid transport	0.82
GO:0045761	regulation of adenylate cyclase activity	0.82
GO:0060198	clathrin sculpted vesicle	0.82
GO:0050755	chemokine metabolic process	0.82
ENSG00000115685	PPP1R7 PPI subnetwork	0.82
ENSG00000083312	TNPO1 PPI subnetwork	0.82
GO:0032964	collagen biosynthetic process	0.82
GO:0033205	cell cycle cytokinesis	0.82
GO:0009914	hormone transport	0.82

Original gene set ID	Original gene set description	Nominal P value
ENSG00000129170	CSRP3 PPI subnetwork	0.82
GO:0042776	mitochondrial ATP synthesis coupled proton transport	0.82
MP:0001968	abnormal touch/ nociception	0.82
KEGG_GAP_JUNCTION	KEGG_GAP_JUNCTION	0.82
REACTOME_FORMATION_OF_RNA_POL_II_ELONGATION_COMPLEX	REACTOME_FORMATION_OF_RNA_POL_II_ELONGATION_COMPLEX	0.82
REACTOME_RNA_POLYMERASE_II_TRANSCRIPTION_ELONGATION	REACTOME_RNA_POLYMERASE_II_TRANSCRIPTION_ELONGATION	0.82
REACTOME_FORMATION_OF_HIV:1_ELONGATION_COMPLEX_IN_THE_ABSENC	REACTOME_FORMATION_OF_HIV:1_ELONGATION_COMPLEX_IN_THE_ABSENCE_O	0.82
MP:0008661	decreased interleukin-10 secretion	0.82
ENSG00000143702	CEP170 PPI subnetwork	0.82
GO:0046961	proton-transporting ATPase activity, rotational mechanism	0.82
MP:0005449	abnormal food intake	0.82
ENSG00000090273	NUDC PPI subnetwork	0.82
GO:0045259	proton-transporting ATP synthase complex	0.82
GO:0021511	spinal cord patterning	0.82
ENSG00000111361	EIF2B1 PPI subnetwork	0.82
GO:0051932	synaptic transmission, GABAergic	0.82
GO:0009306	protein secretion	0.82
GO:0042744	hydrogen peroxide catabolic process	0.82
ENSG00000136152	COG3 PPI subnetwork	0.82
ENSG00000196961	AP2A1 PPI subnetwork	0.82
ENSG00000102096	PIM2 PPI subnetwork	0.82
ENSG00000169439	SDC2 PPI subnetwork	0.82
GO:0048645	organ formation	0.82
ENSG00000185624	P4HB PPI subnetwork	0.82
ENSG00000080986	NDC80 PPI subnetwork	0.82
GO:0097060	synaptic membrane	0.82
GO:0007632	visual behavior	0.82
MP:0004462	small basisphenoid bone	0.82
GO:0003785	actin monomer binding	0.82
REACTOME_GLUCAGON_SIGNALING_IN_METABOLIC_REGULATION	REACTOME_GLUCAGON_SIGNALING_IN_METABOLIC_REGULATION	0.82
GO:0050432	catecholamine secretion	0.82
GO:0031267	small GTPase binding	0.82
MP:0005352	small cranium	0.82
MP:0009712	impaired conditioned place preference behavior	0.82
ENSG00000175115	PACS1 PPI subnetwork	0.82
ENSG00000091651	ORC6 PPI subnetwork	0.82
GO:0005525	GTP binding	0.82
ENSG00000089280	FUS PPI subnetwork	0.82
GO:0034704	calcium channel complex	0.82
ENSG00000214122	ENSG00000214122 PPI subnetwork	0.82
ENSG00000145241	CENPC1 PPI subnetwork	0.82
ENSG00000117713	ARID1A PPI subnetwork	0.82
ENSG00000139546	TARBP2 PPI subnetwork	0.82
ENSG00000213672	NCKIPSD PPI subnetwork	0.82
ENSG00000170927	PKHD1 PPI subnetwork	0.82
ENSG00000080345	RIF1 PPI subnetwork	0.82
GO:0001660	fever generation	0.82
ENSG00000131795	RBM8A PPI subnetwork	0.82
ENSG00000015285	WAS PPI subnetwork	0.82

Original gene set ID	Original gene set description	Nominal P value
GO:0006103	2-oxoglutarate metabolic process	0.82
ENSG00000116350	SRSF4 PPI subnetwork	0.83
GO:0030073	insulin secretion	0.83
GO:0007602	phototransduction	0.83
GO:0032281	alpha-amino-3-hydroxy-5-methyl-4-isoxazolepropionic acid selective glutamate rece	0.83
GO:0032153	cell division site	0.83
GO:0032155	cell division site part	0.83
MP:0005027	increased susceptibility to parasitic infectior	0.83
ENSG00000125651	GTF2F1 PPI subnetwork	0.83
REACTOME_TAT:MEDIATED_ELONGATION_OF_THE_HIV:1_TRANSCRIPT	REACTOME_TAT:MEDIATED_ELONGATION_OF_THE_HIV:1_TRANSCRIPT	0.83
REACTOME_HIV:1_TRANSCRIPTION_ELONGATION	REACTOME_HIV:1_TRANSCRIPTION_ELONGATION	0.83
REACTOME_FORMATION_OF_HIV:1_ELONGATION_COMPLEX_CONTAINING_HI\	REACTOME_FORMATION_OF_HIV:1_ELONGATION_COMPLEX_CONTAINING_HIV:1_	0.83
ENSG00000163541	SUCLG1 PPI subnetwork	0.83
MP:0010053	decreased grip strength	0.83
ENSG00000077420	APBB1IP PPI subnetwork	0.83
GO:0051168	nuclear export	0.83
GO:0006997	nucleus organization	0.83
ENSG00000167110	GOLGA2 PPI subnetwork	0.83
GO:0009581	detection of external stimulus	0.83
ENSG00000159131	GART PPI subnetwork	0.83
ENSG00000168148	HIST3H3 PPI subnetwork	0.83
ENSG00000165916	PSMC3 PPI subnetwork	0.83
ENSG00000073792	IGF2BP2 PPI subnetwork	0.83
ENSG00000072315	TRPC5 PPI subnetwork	0.83
GO:0030574	collagen catabolic process	0.83
ENSG00000158869	FCER1G PPI subnetwork	0.83
MP:0003861	abnormal nervous system development	0.83
ENSG00000013573	DDX11 PPI subnetwork	0.83
GO:0001508	regulation of action potential	0.83
MP:0003924	herniated diaphragm	0.83
GO:0031076	embryonic camera-type eye development	0.83
ENSG00000122126	OCRL PPI subnetwork	0.83
ENSG00000168291	PDHB PPI subnetwork	0.83
ENSG00000183395	PMCH PPI subnetwork	0.83
ENSG00000182054	IDH2 PPI subnetwork	0.83
REACTOME_GLOBAL_GENOMIC_NER_GG:NER	REACTOME_GLOBAL_GENOMIC_NER_GG:NER	0.83
MP:0005107	abnormal stapes morphology	0.83
GO:0005484	SNAP receptor activity	0.83
MP:0003992	increased mortality induced by ionizing radiatior	0.83
GO:0019829	cation-transporting ATPase activity	0.83
ENSG00000167283	ATP5L PPI subnetwork	0.83
GO:0042730	fibrinolysis	0.83
GO:0034654	nucleobase-containing compound biosynthetic process	0.83
REACTOME_HIV:1_TRANSCRIPTION_INITIATION	REACTOME_HIV:1_TRANSCRIPTION_INITIATION	0.83
REACTOME_RNA_POLYMERASE_II_TRANSCRIPTION_PRE:INITIATION_AND_PRO	REACTOME_RNA_POLYMERASE_II_TRANSCRIPTION_PRE:INITIATION_AND_PROMO	0.83
REACTOME_RNA_POLYMERASE_II_TRANSCRIPTION_INITIATION	REACTOME_RNA_POLYMERASE_II_TRANSCRIPTION_INITIATION	0.83
REACTOME_RNA_POLYMERASE_II_HIV:1_PROMOTER_ESCAPE	REACTOME_RNA_POLYMERASE_II_HIV:1_PROMOTER_ESCAPE	0.83
REACTOME_RNA_POLYMERASE_II_TRANSCRIPTION_INITIATION_AND_PROMOT	REACTOME_RNA_POLYMERASE_II_TRANSCRIPTION_INITIATION_AND_PROMOTER_	0.83
REACTOME_RNA_POLYMERASE_II_PROMOTER_ESCAPE	REACTOME_RNA_POLYMERASE_II_PROMOTER_ESCAPE	0.83

Original gene set ID	Original gene set description	Nominal P value
GO:0051437	positive regulation of ubiquitin-protein ligase activity involved in mitotic cell cycle	0.83
ENSG00000185532	PRKG1 PPI subnetwork	0.83
ENSG00000089169	RPH3A PPI subnetwork	0.83
GO:0015980	energy derivation by oxidation of organic compounds	0.83
ENSG00000160062	ZBTB8A PPI subnetwork	0.83
MP:0000933	abnormal rhombomere morphology	0.83
ENSG00000178952	TUFM PPI subnetwork	0.83
GO:0033044	regulation of chromosome organization	0.83
GO:0016701	oxidoreductase activity, acting on single donors with incorporation of molecular oxy	0.83
ENSG00000160803	UBQLN4 PPI subnetwork	0.83
ENSG00000100519	PSMC6 PPI subnetwork	0.83
ENSG00000073050	XRCC1 PPI subnetwork	0.83
GO:0018130	heterocycle biosynthetic process	0.83
ENSG00000078140	UBE2K PPI subnetwork	0.83
ENSG00000171475	WIPF2 PPI subnetwork	0.83
MP:0001666	abnormal intestinal absorption	0.83
ENSG00000146047	HIST1H2BA PPI subnetwork	0.83
GO:0016445	somatic diversification of immunoglobulins	0.83
ENSG00000150753	CCT5 PPI subnetwork	0.83
GO:0009074	aromatic amino acid family catabolic process	0.83
ENSG00000171195	MUC7 PPI subnetwork	0.83
GO:0006901	vesicle coating	0.83
KEGG_RETINOL_METABOLISM	KEGG_RETINOL_METABOLISM	0.83
GO:0007271	synaptic transmission, cholinergic	0.83
GO:0006278	RNA-dependent DNA replication	0.83
MP:0004756	abnormal proximal convoluted tubule morphology	0.83
REACTOME_THE_ROLE_OF_NEF_IN_HIV:1_REPLICATION_AND_DISEASE_PATHO	REACTOME_THE_ROLE_OF_NEF_IN_HIV:1_REPLICATION_AND_DISEASE_PATHOGEN	0.83
ENSG00000129152	MYOD1 PPI subnetwork	0.83
MP:0001693	failure of primitive streak formation	0.83
GO:0000209	protein polyubiquitination	0.83
ENSG00000198211	TUBB3 PPI subnetwork	0.83
ENSG00000176788	BASP1 PPI subnetwork	0.83
ENSG00000094804	CDC6 PPI subnetwork	0.83
ENSG00000068796	KIF2A PPI subnetwork	0.83
MP:0010868	increased bone trabecula number	0.83
GO:0016675	oxidoreductase activity, acting on a heme group of donors	0.83
GO:0051339	regulation of lyase activity	0.83
ENSG00000176248	ANAPC2 PPI subnetwork	0.83
ENSG00000156467	UQCRB PPI subnetwork	0.83
GO:0005813	centrosome	0.83
ENSG00000135213	POM121C PPI subnetwork	0.83
ENSG00000119383	PPP2R4 PPI subnetwork	0.83
ENSG00000184381	PLA2G6 PPI subnetwork	0.83
ENSG00000138778	CENPE PPI subnetwork	0.83
ENSG00000167549	CORO6 PPI subnetwork	0.83
GO:0005126	cytokine receptor binding	0.83
ENSG00000188170	ENSG00000188170 PPI subnetwork	0.83
REACTOME_FORMATION_OF_ATP_BY_CHEMIOSMOTIC_COUPLING	REACTOME_FORMATION_OF_ATP_BY_CHEMIOSMOTIC_COUPLING	0.83
REACTOME_EGFR_DOWNREGULATION	REACTOME_EGFR_DOWNREGULATION	0.83

Original gene set ID	Original gene set description	Nominal P value
ENSG00000130340	SNX9 PPI subnetwork	0.83
GO:0005849	mRNA cleavage factor complex	0.83
GO:0048305	immunoglobulin secretion	0.83
REACTOME_PKA_ACTIVATION	REACTOME_PKA_ACTIVATION	0.83
REACTOME_PROCESSING_OF_CAPPED_INTRON:CONTAINING_PRE:MRNA	REACTOME_PROCESSING_OF_CAPPED_INTRON:CONTAINING_PRE:MRNA	0.83
ENSG00000177084	POLE PPI subnetwork	0.83
ENSG00000163161	ERCC3 PPI subnetwork	0.83
ENSG00000133116	KL PPI subnetwork	0.83
ENSG00000141034	C17orf39 PPI subnetwork	0.83
ENSG00000110801	PSMD9 PPI subnetwork	0.83
ENSG00000150086	GRIN2B PPI subnetwork	0.83
ENSG00000159164	SV2A PPI subnetwork	0.83
GO:0050670	regulation of lymphocyte proliferation	0.83
GO:0051169	nuclear transport	0.84
ENSG00000091181	IL5RA PPI subnetwork	0.84
GO:0009070	serine family amino acid biosynthetic process	0.84
MP:0003311	aminoaciduria	0.84
REACTOME_MRNA_SPLICING_:_MINOR_PATHWAY	REACTOME_MRNA_SPLICING_:_MINOR_PATHWAY	0.84
ENSG00000112379	KIAA1244 PPI subnetwork	0.84
MP:0001415	increased exploration in new environment	0.84
GO:0006260	DNA replication	0.84
GO:0009584	detection of visible light	0.84
ENSG00000100292	HMOX1 PPI subnetwork	0.84
ENSG00000178741	COX5A PPI subnetwork	0.84
GO:0090101	negative regulation of transmembrane receptor protein serine/threonine kinase sig	0.84
GO:0031279	regulation of cyclase activity	0.84
MP:0006108	abnormal hindbrain development	0.84
GO:0031401	positive regulation of protein modification process	0.84
MP:0002753	dilated heart left ventricle	0.84
GO:0008188	neuropeptide receptor activity	0.84
ENSG00000132464	ENAM PPI subnetwork	0.84
ENSG00000075785	RAB7A PPI subnetwork	0.84
GO:0006206	pyrimidine base metabolic process	0.84
GO:0016879	ligase activity, forming carbon-nitrogen bonds	0.84
ENSG00000133226	SRRM1 PPI subnetwork	0.84
GO:0016918	retinal binding	0.84
ENSG00000111786	SRSF9 PPI subnetwork	0.84
GO:0004222	metalloendopeptidase activity	0.84
GO:0048037	cofactor binding	0.84
REACTOME_DEADENYLATION:DEPENDENT_MRNA_DECAY	REACTOME_DEADENYLATION:DEPENDENT_MRNA_DECAY	0.84
GO:0006091	generation of precursor metabolites and energy	0.84
GO:0009607	response to biotic stimulus	0.84
ENSG00000204356	RDBP PPI subnetwork	0.84
ENSG00000206268	RDBP PPI subnetwork	0.84
ENSG00000206357	RDBP PPI subnetwork	0.84
ENSG00000183625	CCR3 PPI subnetwork	0.84
ENSG00000160201	U2AF1 PPI subnetwork	0.84
ENSG00000100650	SRSF5 PPI subnetwork	0.84
ENSG00000156261	CCT8 PPI subnetwork	0.84

Original gene set ID	Original gene set description	Nominal P value
ENSG00000169750	RAC3 PPI subnetwork	0.84
GO:0008333	endosome to lysosome transport	0.84
GO:0016180	snRNA processing	0.84
GO:0032270	positive regulation of cellular protein metabolic process	0.84
MP:0004777	abnormal phospholipid level	0.84
GO:0006816	calcium ion transport	0.84
ENSG00000085415	SEH1L PPI subnetwork	0.84
MP:0003484	abnormal channel response	0.84
GO:0005501	retinoid binding	0.84
GO:0005689	U12-type spliceosomal complex	0.84
GO:0030804	positive regulation of cyclic nucleotide biosynthetic process	0.84
GO:0030810	positive regulation of nucleotide biosynthetic process	0.84
ENSG00000137337	MDC1 PPI subnetwork	0.84
ENSG00000206385	ENSG00000206385 PPI subnetwork	0.84
GO:0035148	tube formation	0.84
REACTOME_CHEMOKINE_RECEPTORS_BIND_CHEMOKINES	REACTOME_CHEMOKINE_RECEPTORS_BIND_CHEMOKINES	0.84
ENSG00000117560	FASLG PPI subnetwork	0.84
ENSG00000120910	PPP3CC PPI subnetwork	0.84
ENSG00000103740	ACSBG1 PPI subnetwork	0.84
ENSG00000149295	DRD2 PPI subnetwork	0.84
REACTOME_LATE_PHASE_OF_HIV_LIFE_CYCLE	REACTOME_LATE_PHASE_OF_HIV_LIFE_CYCLE	0.84
ENSG00000141367	CLTC PPI subnetwork	0.84
ENSG00000152700	SAR1B PPI subnetwork	0.84
GO:2000177	regulation of neural precursor cell proliferation	0.84
REACTOME_DEFENSINS	REACTOME_DEFENSINS	0.84
ENSG00000215305	VPS16 PPI subnetwork	0.84
ENSG00000105926	MPP6 PPI subnetwork	0.84
GO:0007416	synapse assembly	0.84
GO:0031902	late endosome membrane	0.84
GO:0032673	regulation of interleukin-4 production	0.84
MP:0002893	ketoaciduria	0.84
ENSG00000168530	MYL1 PPI subnetwork	0.84
GO:0021537	telencephalon development	0.84
GO:0031397	negative regulation of protein ubiquitination	0.84
ENSG00000143727	ACP1 PPI subnetwork	0.84
REACTOME_PRESYNAPTIC_FUNCTION_OF_KAINATE_RECEPTORS	REACTOME_PRESYNAPTIC_FUNCTION_OF_KAINATE_RECEPTORS	0.84
GO:0009617	response to bacterium	0.84
GO:0030903	notochord development	0.84
GO:0006979	response to oxidative stress	0.84
ENSG00000158864	NDUFS2 PPI subnetwork	0.84
GO:0004033	aldo-keto reductase (NADP) activity	0.84
ENSG00000176986	SEC24C PPI subnetwork	0.84
GO:0006368	transcription elongation from RNA polymerase II promoter	0.84
REACTOME_HIV_LIFE_CYCLE	REACTOME_HIV_LIFE_CYCLE	0.84
GO:0048484	enteric nervous system development	0.84
MP:0010810	increased type II pneumocyte number	0.84
GO:0072079	nephron tubule formation	0.84
MP:0002084	abnormal developmental patterning	0.84
ENSG00000160551	TAOK1 PPI subnetwork	0.84

Original gene set ID	Original gene set description	Nominal P value
ENSG00000122180	MYOG PPI subnetwork	0.84
ENSG00000144642	RBMS3 PPI subnetwork	0.84
GO:0005125	cytokine activity	0.84
ENSG00000180190	C8orf42 PPI subnetwork	0.84
ENSG00000151224	MAT1A PPI subnetwork	0.84
GO:0032633	interleukin-4 production	0.84
ENSG00000164815	ORC5 PPI subnetwork	0.84
ENSG00000101413	RPRD1B PPI subnetwork	0.84
ENSG00000156049	GNA14 PPI subnetwork	0.84
ENSG00000125351	UPF3B PPI subnetwork	0.84
GO:0051054	positive regulation of DNA metabolic process	0.84
MP:0000877	abnormal Purkinje cell morphology	0.84
MP:0003730	abnormal photoreceptor inner segment morphology	0.84
GO:0032784	regulation of transcription elongation, DNA-dependent	0.84
GO:0002474	antigen processing and presentation of peptide antigen via MHC class	0.84
ENSG00000149311	ATM PPI subnetwork	0.84
GO:0060512	prostate gland morphogenesis	0.84
MP:0000929	open neural tube	0.84
GO:0042613	MHC class II protein complex	0.84
GO:0000375	RNA splicing, via transesterification reactions	0.84
REACTOME_E2F_MEDIATED_REGULATION_OF_DNA_REPLICATION	REACTOME_E2F_MEDIATED_REGULATION_OF_DNA_REPLICATION	0.84
ENSG00000134602	ENSG00000134602 PPI subnetwork	0.84
ENSG00000085662	AKR1B1 PPI subnetwork	0.84
GO:0009635	response to herbicide	0.84
GO:0008353	RNA polymerase II carboxy-terminal domain kinase activity	0.84
REACTOME_MUSCLE_CONTRACTION	REACTOME_MUSCLE_CONTRACTION	0.84
GO:0006220	pyrimidine nucleotide metabolic process	0.84
GO:0043395	heparan sulfate proteoglycan binding	0.84
GO:0007033	vacuole organization	0.84
MP:0004073	caudal body truncation	0.84
REACTOME_PROCESSING_OF_INTRONLESS_PRE:MRNAS	REACTOME_PROCESSING_OF_INTRONLESS_PRE:MRNAS	0.84
REACTOME_MRNA_PROCESSING	REACTOME_MRNA_PROCESSING	0.84
GO:0006511	ubiquitin-dependent protein catabolic process	0.84
GO:0000152	nuclear ubiquitin ligase complex	0.85
GO:0004993	serotonin receptor activity	0.85
ENSG00000138385	SSB PPI subnetwork	0.85
ENSG00000172409	CLP1 PPI subnetwork	0.85
GO:0009165	nucleotide biosynthetic process	0.85
ENSG00000163737	PF4 PPI subnetwork	0.85
REACTOME_DNA_REPAIR	REACTOME_DNA_REPAIR	0.85
ENSG00000169020	ATP5I PPI subnetwork	0.85
GO:0006813	potassium ion transport	0.85
REACTOME_TRANSCRIPTION_OF_THE_HIV_GENOME	REACTOME_TRANSCRIPTION_OF_THE_HIV_GENOME	0.85
GO:0030312	external encapsulating structure	0.85
GO:0006140	regulation of nucleotide metabolic process	0.85
ENSG00000100804	PSMB5 PPI subnetwork	0.85
MP:0005650	abnormal limb bud morphology	0.85
MP:0002718	abnormal inner cell mass morphology	0.85
REACTOME_MYOGENESIS	REACTOME_MYOGENESIS	0.85

Original gene set ID	Original gene set description	Nominal P value
REACTOME_CDO_IN_MYOGENESIS	REACTOME_CDO_IN_MYOGENESIS	0.85
GO:0048813	dendrite morphogenesis	0.85
MP:0009336	increased splenocyte proliferation	0.85
ENSG00000060339	CCAR1 PPI subnetwork	0.85
GO:0030838	positive regulation of actin filament polymerization	0.85
GO:0050807	regulation of synapse organization	0.85
MP:0003290	intestinal hypoperistalsis	0.85
REACTOME_TRANSPORT_OF_MATURE_MRNA_DERIVED_FROM_AN_INTRONLE	REACTOME_TRANSPORT_OF_MATURE_MRNA_DERIVED_FROM_AN_INTRONLESS_1	0.85
GO:0009799	specification of symmetry	0.85
ENSG00000115145	STAM2 PPI subnetwork	0.85
GO:0032944	regulation of mononuclear cell proliferation	0.85
MP:0000272	abnormal aorta morphology	0.85
GO:0015812	gamma-aminobutyric acid transport	0.85
ENSG00000115875	SRSF7 PPI subnetwork	0.85
GO:0042220	response to cocaine	0.85
GO:0014073	response to tropane	0.85
ENSG00000171724	VAT1L PPI subnetwork	0.85
MP:0002575	increased circulating ketone body level	0.85
GO:0004842	ubiquitin-protein ligase activity	0.85
ENSG00000109111	SUPT6H PPI subnetwork	0.85
ENSG00000144028	SNRNP200 PPI subnetwork	0.85
MP:0000266	abnormal heart morphology	0.85
ENSG00000142039	CCDC97 PPI subnetwork	0.85
GO:0005815	microtubule organizing center	0.85
GO:0048701	embryonic cranial skeleton morphogenesis	0.85
ENSG00000198523	PLN PPI subnetwork	0.85
GO:0051443	positive regulation of ubiquitin-protein ligase activity	0.85
ENSG00000136450	SRSF1 PPI subnetwork	0.85
GO:0042743	hydrogen peroxide metabolic process	0.85
REACTOME_SIGNALLING_TO_P38_VIA_RIT_AND_RIN	REACTOME_SIGNALLING_TO_P38_VIA_RIT_AND_RIN	0.85
ENSG00000205531	NAP1L4 PPI subnetwork	0.85
ENSG00000088256	GNA11 PPI subnetwork	0.85
ENSG00000157020	SEC13 PPI subnetwork	0.85
REACTOME_PKA_ACTIVATION_IN_GLUCAGON_SIGNALLING	REACTOME_PKA_ACTIVATION_IN_GLUCAGON_SIGNALLING	0.85
REACTOME_CALCITONIN:LIKE_LIGAND_RECEPTORS	REACTOME_CALCITONIN:LIKE_LIGAND_RECEPTORS	0.85
ENSG00000110717	NDUFS8 PPI subnetwork	0.85
ENSG00000102387	TAF7L PPI subnetwork	0.85
ENSG00000129084	PSMA1 PPI subnetwork	0.85
ENSG00000100280	AP1B1 PPI subnetwork	0.85
GO:0006360	transcription from RNA polymerase I promoter	0.85
GO:0051536	iron-sulfur cluster binding	0.85
GO:0051540	metal cluster binding	0.85
ENSG00000134444	KIAA1468 PPI subnetwork	0.85
GO:0016032	viral reproduction	0.85
ENSG00000141232	TOB1 PPI subnetwork	0.85
GO:0030239	myofibril assembly	0.85
MP:0006089	abnormal vestibular saccule morphology	0.85
ENSG00000105705	SUGP1 PPI subnetwork	0.85
MP:0003149	abnormal tectorial membrane morphology	0.85

Original gene set ID	Original gene set description	Nominal P value
ENSG00000142949	PTPRF PPI subnetwork	0.85
GO:0019882	antigen processing and presentation	0.85
REACTOME_REGULATION_OF_WATER_BALANCE_BY_RENAL_AQUAPORINS	REACTOME_REGULATION_OF_WATER_BALANCE_BY_RENAL_AQUAPORINS	0.85
GO:0015035	protein disulfide oxidoreductase activity	0.85
GO:0016627	oxidoreductase activity, acting on the CH-CH group of donors	0.85
GO:0016836	hydro-lyase activity	0.85
GO:0001906	cell killing	0.85
ENSG00000159186	ENSG00000159186 PPI subnetwork	0.85
ENSG00000122565	CBX3 PPI subnetwork	0.85
ENSG00000182004	SNRPE PPI subnetwork	0.85
REACTOME_TRANSPORT_OF_THE_SLBP_INDEPENDENT_MATURE_MRNA	REACTOME_TRANSPORT_OF_THE_SLBP_INDEPENDENT_MATURE_MRNA	0.85
GO:0006986	response to unfolded protein	0.85
GO:0051865	protein autoubiquitination	0.85
GO:0061326	renal tubule development	0.85
ENSG00000101138	CSTF1 PPI subnetwork	0.85
GO:0015850	organic alcohol transport	0.85
KEGG_BETA_ALANINE_METABOLISM	KEGG_BETA_ALANINE_METABOLISM	0.85
ENSG00000141200	KIF2B PPI subnetwork	0.85
GO:0046034	ATP metabolic process	0.85
ENSG00000039123	SKIV2L2 PPI subnetwork	0.85
GO:0021516	dorsal spinal cord development	0.85
ENSG00000154839	SKA1 PPI subnetwork	0.85
GO:0006941	striated muscle contraction	0.85
MP:0002894	abnormal otolith morphology	0.85
GO:0016410	N-acyltransferase activity	0.85
ENSG00000147274	RBMX PPI subnetwork	0.85
ENSG00000165659	DACH1 PPI subnetwork	0.85
GO:0019400	alditol metabolic process	0.85
ENSG00000137177	KIF13A PPI subnetwork	0.85
ENSG00000121542	SEC22A PPI subnetwork	0.85
KEGG_ALANINE_ASPARTATE_AND_GLUTAMATE_METABOLISM	KEGG_ALANINE_ASPARTATE_AND_GLUTAMATE_METABOLISM	0.85
GO:0043394	proteoglycan binding	0.85
GO:0046135	pyrimidine nucleoside catabolic process	0.85
KEGG_VIRAL_MYOCARDITIS	KEGG_VIRAL_MYOCARDITIS	0.85
ENSG00000198961	PJA2 PPI subnetwork	0.85
ENSG00000167005	NUDT21 PPI subnetwork	0.85
GO:0000398	nuclear mRNA splicing, via spliceosome	0.85
GO:0000377	RNA splicing, via transesterification reactions with bulged adenosine as nucleophile	0.85
ENSG00000123360	PDE1B PPI subnetwork	0.85
ENSG00000198730	CTR9 PPI subnetwork	0.85
ENSG00000115524	SF3B1 PPI subnetwork	0.85
GO:0006402	mRNA catabolic process	0.85
GO:0007004	telomere maintenance via telomerase	0.85
GO:0018105	peptidyl-serine phosphorylation	0.85
GO:0051444	negative regulation of ubiquitin-protein ligase activity	0.86
GO:0051352	negative regulation of ligase activity	0.86
GO:0021545	cranial nerve development	0.86
GO:0072498	embryonic skeletal joint development	0.86
GO:0031400	negative regulation of protein modification process	0.86

Original gene set ID	Original gene set description	Nominal P value
GO:0003382	epithelial cell morphogenesis	0.86
GO:0008608	attachment of spindle microtubules to kinetochore	0.86
GO:0042354	L-fucose metabolic process	0.86
ENSG00000133812	SBF2 PPI subnetwork	0.86
ENSG00000124507	PACSIN1 PPI subnetwork	0.86
MP:0004738	abnormal brainstem auditory evoked potentia	0.86
REACTOME_PROSTACYCLIN_SIGNALLING_THROUGH_PROSTACYCLIN_RECEPTOI	REACTOME_PROSTACYCLIN_SIGNALLING_THROUGH_PROSTACYCLIN_RECEPTOR	0.86
ENSG00000143079	CTTNBP2NL PPI subnetwork	0.86
GO:0006887	exocytosis	0.86
ENSG00000152056	AP1S3 PPI subnetwork	0.86
REACTOME_CAM_PATHWAY	REACTOME_CAM_PATHWAY	0.86
REACTOME_CALMODULIN_INDUCED_EVENTS	REACTOME_CALMODULIN_INDUCED_EVENTS	0.86
GO:0000781	chromosome, telomeric region	0.86
REACTOME_AMINE_LIGAND:BINDING_RECEPTORS	REACTOME_AMINE_LIGAND:BINDING_RECEPTORS	0.86
ENSG00000107371	EXOSC3 PPI subnetwork	0.86
REACTOME_SIGNALING_BY_ROBO_RECEPTOR	REACTOME_SIGNALING_BY_ROBO_RECEPTOR	0.86
ENSG00000188986	COBRA1 PPI subnetwork	0.86
REACTOME_ADHERENS_JUNCTIONS_INTERACTIONS	REACTOME_ADHERENS_JUNCTIONS_INTERACTIONS	0.86
ENSG00000122566	HNRNPA2B1 PPI subnetwork	0.86
MP:0000359	abnormal mast cell morphology	0.86
MP:0004722	abnormal platelet dense granule number	0.86
ENSG00000188342	GTF2F2 PPI subnetwork	0.86
GO:0001774	microglial cell activation	0.86
ENSG00000127920	GNG11 PPI subnetwork	0.86
ENSG00000112079	STK38 PPI subnetwork	0.86
GO:0009953	dorsal/ventral pattern formation	0.86
GO:0031032	actomyosin structure organization	0.86
GO:0050912	detection of chemical stimulus involved in sensory perception of taste	0.86
ENSG00000132383	RPA1 PPI subnetwork	0.86
ENSG00000086102	NFX1 PPI subnetwork	0.86
ENSG00000182185	RAD51B PPI subnetwork	0.86
ENSG00000183856	IQGAP3 PPI subnetwork	0.86
GO:0048483	autonomic nervous system development	0.86
GO:0023021	termination of signal transduction	0.86
ENSG00000169813	HNRNPF PPI subnetwork	0.86
ENSG00000100503	NIN PPI subnetwork	0.86
ENSG00000159377	PSMB4 PPI subnetwork	0.86
GO:0016702	oxidoreductase activity, acting on single donors with incorporation of molecular oxy	0.86
GO:0009582	detection of abiotic stimulus	0.86
MP:0009760	abnormal mitotic spindle morphology	0.86
MP:0004568	fusion of glossopharyngeal and vagus nerve	0.86
MP:0008148	abnormal rib-sternum attachment	0.86
ENSG00000160199	PKNOX1 PPI subnetwork	0.86
MP:0004970	kidney atrophy	0.86
ENSG00000077063	CTTNBP2 PPI subnetwork	0.86
ENSG00000124193	SRSF6 PPI subnetwork	0.86
MP:0004163	abnormal adenohypophysis morphology	0.86
GO:0034622	cellular macromolecular complex assembly	0.86
ENSG00000145321	GC PPI subnetwork	0.86

Original gene set ID	Original gene set description	Nominal P value
REACTOME_G_ALPHA_I_SIGNALLING_EVENTS	REACTOME_G_ALPHA_I_SIGNALLING_EVENTS	0.86
ENSG00000175550	DRAP1 PPI subnetwork	0.86
MP:0002115	abnormal limb bone morphology	0.86
GO:0044242	cellular lipid catabolic process	0.86
ENSG00000176102	CSTF3 PPI subnetwork	0.86
GO:0016073	snRNA metabolic process	0.86
GO:0004065	arylsulfatase activity	0.86
REACTOME_MRNA_SPLICING	REACTOME_MRNA_SPLICING	0.86
REACTOME_MRNA_SPLICING_:_MAJOR_PATHWAY	REACTOME_MRNA_SPLICING_:_MAJOR_PATHWAY	0.86
ENSG00000185291	IL3RA PPI subnetwork	0.86
GO:0050908	detection of light stimulus involved in visual perception	0.86
GO:0050962	detection of light stimulus involved in sensory perception	0.86
GO:0006200	ATP catabolic process	0.86
MP:0001906	increased dopamine level	0.86
GO:0032228	regulation of synaptic transmission, GABAergic	0.86
ENSG00000156304	SCAF4 PPI subnetwork	0.86
ENSG00000114554	PLXNA1 PPI subnetwork	0.86
ENSG00000132561	MATN2 PPI subnetwork	0.86
ENSG00000100632	ERH PPI subnetwork	0.86
GO:0050660	flavin adenine dinucleotide binding	0.86
GO:0009855	determination of bilateral symmetry	0.86
GO:0045211	postsynaptic membrane	0.86
GO:0019941	modification-dependent protein catabolic process	0.86
ENSG00000137767	SQRDL PPI subnetwork	0.86
GO:0048706	embryonic skeletal system development	0.86
GO:0031201	SNARE complex	0.86
GO:0030258	lipid modification	0.86
GO:0030170	pyridoxal phosphate binding	0.86
GO:0070279	vitamin B6 binding	0.86
ENSG00000139433	GLTP PPI subnetwork	0.86
ENSG00000063601	MTMR1 PPI subnetwork	0.86
ENSG00000148773	MKI67 PPI subnetwork	0.86
REACTOME_SYNTHESIS_OF_BILE_ACIDS_AND_BILE_SALTS_VIA_7ALPHA:HYDRO	REACTOME_SYNTHESIS_OF_BILE_ACIDS_AND_BILE_SALTS_VIA_7ALPHA:HYDROXYC	0.86
MP:0001883	mammary adenocarcinoma	0.86
ENSG00000135744	AGT PPI subnetwork	0.86
MP:0004936	impaired branching involved in ureteric bud morphogenesis	0.86
ENSG00000162385	MAGOH PPI subnetwork	0.86
MP:0004835	abnormal miniature endplate potential	0.86
GO:0031398	positive regulation of protein ubiquitination	0.86
ENSG00000127527	EPS15L1 PPI subnetwork	0.86
ENSG00000103507	BCKDK PPI subnetwork	0.86
GO:0006071	glycerol metabolic process	0.86
KEGG_LONG_TERM_POTENTIATION	KEGG_LONG_TERM_POTENTIATION	0.86
MP:0001970	abnormal pain threshold	0.86
GO:0007420	brain development	0.86
ENSG00000116852	KIF21B PPI subnetwork	0.86
GO:0017016	Ras GTPase binding	0.87
GO:0042594	response to starvation	0.87
ENSG00000071794	HLTF PPI subnetwork	0.87

Original gene set ID	Original gene set description	Nominal P value
MP:0002920	decreased paired-pulse facilitation	0.87
MP:0008272	abnormal endochondral bone ossification	0.87
MP:0002066	abnormal motor capabilities/coordination/movement	0.87
MP:0009133	decreased white fat cell size	0.87
ENSG00000120254	MTHFD1L PPI subnetwork	0.87
GO:0005328	neurotransmitter:sodium symporter activity	0.87
GO:0033108	mitochondrial respiratory chain complex assembly	0.87
ENSG00000206503	HLA-A PPI subnetwork	0.87
ENSG00000013583	HEBP1 PPI subnetwork	0.87
MP:0005171	absent coat pigmentation	0.87
ENSG00000170310	STX8 PPI subnetwork	0.87
REACTOME_CLATHRIN_DERIVED_VESICLE_BUDDING	REACTOME_CLATHRIN_DERIVED_VESICLE_BUDDING	0.87
REACTOME_TRANS:GOLGI_NETWORK_VESICLE_BUDDING	REACTOME_TRANS:GOLGI_NETWORK_VESICLE_BUDDING	0.87
GO:0016790	thiolester hydrolase activity	0.87
ENSG00000151923	TIAL1 PPI subnetwork	0.87
MP:0005193	abnormal anterior eye segment morphology	0.87
ENSG00000137259	HIST1H2AB PPI subnetwork	0.87
ENSG00000168274	HIST1H2AE PPI subnetwork	0.87
REACTOME_NEUROTRANSMITTER_RECEPTOR_BINDING_AND_DOWNSTREAM_	REACTOME_NEUROTRANSMITTER_RECEPTOR_BINDING_AND_DOWNSTREAM_TRA	0.87
GO:0050803	regulation of synapse structure and activity	0.87
ENSG00000163159	VPS72 PPI subnetwork	0.87
ENSG00000111245	MYL2 PPI subnetwork	0.87
ENSG00000155130	MARCKS PPI subnetwork	0.87
GO:0021953	central nervous system neuron differentiation	0.87
GO:0006213	pyrimidine nucleoside metabolic process	0.87
ENSG00000125354	SEPT6 PPI subnetwork	0.87
GO:0043632	modification-dependent macromolecule catabolic process	0.87
ENSG00000090060	PAPOLA PPI subnetwork	0.87
REACTOME_ADP_SIGNALLING_THROUGH_P2Y_PURINOCEPTOR_12	REACTOME_ADP_SIGNALLING_THROUGH_P2Y_PURINOCEPTOR_12	0.87
MP:0002633	persistent truncus arteriosis	0.87
ENSG00000134690	CDCA8 PPI subnetwork	0.87
ENSG00000116329	OPRD1 PPI subnetwork	0.87
GO:0051028	mRNA transport	0.87
MP:0002332	abnormal exercise endurance	0.87
GO:0019787	small conjugating protein ligase activity	0.87
GO:0051966	regulation of synaptic transmission, glutamatergic	0.87
ENSG00000167977	KCTD5 PPI subnetwork	0.87
ENSG00000168298	HIST1H1E PPI subnetwork	0.87
ENSG00000173636	ENSG00000173636 PPI subnetwork	0.87
ENSG00000166477	LEO1 PPI subnetwork	0.87
ENSG00000069424	KCNAB2 PPI subnetwork	0.87
ENSG00000085365	ENSG00000085365 PPI subnetwork	0.87
MP:0010107	abnormal renal reabsorbtion	0.87
ENSG00000089053	ANAPC5 PPI subnetwork	0.87
MP:0005445	abnormal neurotransmitter secretion	0.87
GO:0019363	pyridine nucleotide biosynthetic process	0.87
GO:0072525	pyridine-containing compound biosynthetic process	0.87
GO:0009064	glutamine family amino acid metabolic process	0.87
GO:0007190	activation of adenylate cyclase activity	0.87

Original gene set ID	Original gene set description	Nominal P value
GO:0005523	tropomyosin binding	0.87
MP:0003331	hepatocellular carcinoma	0.87
GO:0016881	acid-amino acid ligase activity	0.87
ENSG00000090470	PDCD7 PPI subnetwork	0.87
ENSG00000102901	CENPT PPI subnetwork	0.87
ENSG00000113649	TCERG1 PPI subnetwork	0.87
GO:0051020	GTPase binding	0.87
GO:0072175	epithelial tube formation	0.87
REACTOME_PKA:MEDIATED_PHOSPHORYLATION_OF_CREB	REACTOME_PKA:MEDIATED_PHOSPHORYLATION_OF_CREB	0.87
ENSG00000070367	EXOC5 PPI subnetwork	0.87
GO:0060571	morphogenesis of an epithelial fold	0.87
GO:0044087	regulation of cellular component biogenesis	0.87
GO:0072404	signal transduction involved in G1/S transition checkpoint	0.87
GO:0072395	signal transduction involved in cell cycle checkpoint	0.87
ENSG00000165178	ENSG00000165178 PPI subnetwork	0.87
ENSG00000033627	ATP6V0A1 PPI subnetwork	0.87
MP:0000106	abnormal basisphenoid bone morphology	0.87
ENSG00000186871	ERCC6L PPI subnetwork	0.87
MP:0004249	abnormal crista ampullaris morphology	0.87
ENSG00000120688	WBP4 PPI subnetwork	0.87
ENSG00000106615	RHEB PPI subnetwork	0.87
REACTOME_G_BETAGAMMA_SIGNALLING_THROUGH_PLC_BETA	REACTOME_G_BETAGAMMA_SIGNALLING_THROUGH_PLC_BETA	0.87
GO:0032409	regulation of transporter activity	0.87
MP:0001293	anophthalmia	0.87
ENSG00000129810	SGOL1 PPI subnetwork	0.87
ENSG00000136810	TXN PPI subnetwork	0.87
GO:0006044	N-acetylglucosamine metabolic process	0.87
GO:0006041	glucosamine metabolic process	0.87
REACTOME_RNA_POLYMERASE_II_TRANSCRIPTION	REACTOME_RNA_POLYMERASE_II_TRANSCRIPTION	0.87
ENSG00000102978	POLR2C PPI subnetwork	0.87
MP:0002151	abnormal neural tube morphology/development	0.87
GO:0007210	serotonin receptor signaling pathway	0.87
GO:0006914	autophagy	0.87
GO:0006536	glutamate metabolic process	0.87
GO:0035966	response to topologically incorrect protein	0.87
MP:0001454	abnormal cued conditioning behavior	0.87
ENSG00000005075	POLR2J PPI subnetwork	0.87
ENSG00000196872	C2orf55 PPI subnetwork	0.87
ENSG00000051180	RAD51 PPI subnetwork	0.87
GO:0008038	neuron recognition	0.87
ENSG00000138308	PLA2G12B PPI subnetwork	0.87
ENSG00000182287	AP1S2 PPI subnetwork	0.87
MP:0002825	abnormal notochord morphology	0.87
ENSG00000135446	CDK4 PPI subnetwork	0.87
MP:0009745	abnormal behavioral response to xenobiotic	0.87
ENSG00000139998	RAB15 PPI subnetwork	0.87
MP:0003036	vertebral transformation	0.87
GO:0043631	RNA polyadenylation	0.87
GO:0002544	chronic inflammatory response	0.87

Original gene set ID	Original gene set description	Nominal P value
ENSG00000165494	PCF11 PPI subnetwork	0.87
ENSG00000083857	FAT1 PPI subnetwork	0.87
ENSG00000070961	ATP2B1 PPI subnetwork	0.87
ENSG00000138668	HNRNPD PPI subnetwork	0.87
ENSG00000068878	PSME4 PPI subnetwork	0.87
MP:0003864	abnormal midbrain development	0.87
MP:0005191	head tilt	0.87
MP:0000189	hypoglycemia	0.87
ENSG00000171497	PPID PPI subnetwork	0.87
ENSG00000125266	EFNB2 PPI subnetwork	0.87
GO:0070403	NAD+ binding	0.87
ENSG00000149532	CPSF7 PPI subnetwork	0.87
GO:0021527	spinal cord association neuron differentiatior	0.87
GO:0032813	tumor necrosis factor receptor superfamily binding	0.88
ENSG00000174851	YIF1A PPI subnetwork	0.88
GO:0060487	lung epithelial cell differentiation	0.88
ENSG00000141503	MINK1 PPI subnetwork	0.88
ENSG00000080603	SRCAP PPI subnetwork	0.88
ENSG00000099622	CIRBP PPI subnetwork	0.88
GO:0051213	dioxygenase activity	0.88
REACTOME_REGULATION_OF_INSULIN_SECRETION_BY_GLUCAGON:LIKE_PEPTI	REACTOME_REGULATION_OF_INSULIN_SECRETION_BY_GLUCAGON:LIKE_PEPTIDE:1	0.88
ENSG00000105011	ASF1B PPI subnetwork	0.88
MP:0004819	decreased skeletal muscle mass	0.88
REACTOME_AMYLOIDS	REACTOME_AMYLOIDS	0.88
MP:0005075	abnormal melanosome morphology	0.88
KEGG_TRYPTOPHAN_METABOLISM	KEGG_TRYPTOPHAN_METABOLISM	0.88
ENSG00000031691	CENPQ PPI subnetwork	0.88
GO:2000516	positive regulation of CD4-positive, alpha-beta T cell activatior	0.88
GO:0043372	positive regulation of CD4-positive, alpha-beta T cell differentiatior	0.88
ENSG00000120334	CENPL PPI subnetwork	0.88
ENSG00000116095	PLEKHA3 PPI subnetwork	0.88
GO:0045762	positive regulation of adenylate cyclase activity	0.88
GO:0031281	positive regulation of cyclase activity	0.88
ENSG00000089685	BIRC5 PPI subnetwork	0.88
GO:0021781	glial cell fate commitment	0.88
ENSG00000120699	EXOSC8 PPI subnetwork	0.88
ENSG00000103363	TCEB2 PPI subnetwork	0.88
MP:0010984	abnormal metanephric mesenchyme morphology	0.88
ENSG00000169213	RAB3B PPI subnetwork	0.88
ENSG00000179899	ENSG00000179899 PPI subnetwork	0.88
ENSG00000196531	NACA PPI subnetwork	0.88
GO:0072080	nephron tubule development	0.88
MP:0004066	abnormal primitive node morphology	0.88
GO:0051439	regulation of ubiquitin-protein ligase activity involved in mitotic cell cycle	0.88
ENSG00000148835	TAF5 PPI subnetwork	0.88
ENSG00000095585	BLNK PPI subnetwork	0.88
GO:0001838	embryonic epithelial tube formation	0.88
ENSG00000184408	KCND2 PPI subnetwork	0.88
ENSG00000171793	CTPS PPI subnetwork	0.88

Original gene set ID	Original gene set description	Nominal P value
ENSG00000197905	TEAD4 PPI subnetwork	0.88
ENSG00000140379	BCL2A1 PPI subnetwork	0.88
MP:0002058	neonatal lethality	0.88
GO:0032934	sterol binding	0.88
MP:0005157	holoprosencephaly	0.88
GO:0048002	antigen processing and presentation of peptide antigen	0.88
GO:0051058	negative regulation of small GTPase mediated signal transduction	0.88
MP:0004599	abnormal vertebral arch morphology	0.88
GO:0051349	positive regulation of lyase activity	0.88
GO:0031145	anaphase-promoting complex-dependent proteasomal ubiquitin-dependent protein	0.88
GO:0007223	Wnt receptor signaling pathway, calcium modulating pathway	0.88
GO:0007218	neuropeptide signaling pathway	0.88
GO:0032835	glomerulus development	0.88
GO:0000236	mitotic prometaphase	0.88
REACTOME_AMINO_ACID_SYNTHESIS_AND_INTERCONVERSION_TRANSAMINAT	REACTOME_AMINO_ACID_SYNTHESIS_AND_INTERCONVERSION_TRANSAMINATION	0.88
GO:0035249	synaptic transmission, glutamatergic	0.88
GO:0005753	mitochondrial proton-transporting ATP synthase complex	0.88
ENSG00000184575	XPOT PPI subnetwork	0.88
ENSG00000137947	GTF2B PPI subnetwork	0.88
GO:0010972	negative regulation of G2/M transition of mitotic cell cycle	0.88
ENSG00000110768	GTF2H1 PPI subnetwork	0.88
ENSG00000117153	KLHL12 PPI subnetwork	0.88
ENSG00000168002	POLR2G PPI subnetwork	0.88
ENSG00000138663	COPS4 PPI subnetwork	0.88
MP:0003120	abnormal tracheal cartilage morphology	0.88
ENSG00000172660	TAF15 PPI subnetwork	0.88
ENSG00000119203	CPSF3 PPI subnetwork	0.88
ENSG00000164076	CAMKV PPI subnetwork	0.88
MP:0000783	abnormal forebrain morphology	0.88
ENSG00000132334	PTPRE PPI subnetwork	0.88
MP:0002841	impaired skeletal muscle contractility	0.88
ENSG00000132692	BCAN PPI subnetwork	0.88
ENSG00000095380	NANS PPI subnetwork	0.88
MP:0000270	abnormal heart tube morphology	0.88
GO:0003009	skeletal muscle contraction	0.88
GO:0034614	cellular response to reactive oxygen species	0.88
GO:0060590	ATPase regulator activity	0.88
REACTOME_MEMBRANE_TRAFFICKING	REACTOME_MEMBRANE_TRAFFICKING	0.88
ENSG00000102384	CENPI PPI subnetwork	0.88
GO:0032465	regulation of cytokinesis	0.88
GO:0006284	base-excision repair	0.88
ENSG00000137642	SORL1 PPI subnetwork	0.88
ENSG00000106100	NOD1 PPI subnetwork	0.88
GO:0045981	positive regulation of nucleotide metabolic process	0.88
ENSG00000197111	PCBP2 PPI subnetwork	0.88
ENSG00000177302	TOP3A PPI subnetwork	0.88
ENSG00000165934	CPSF2 PPI subnetwork	0.88
GO:0042088	T-helper 1 type immune response	0.88
GO:0047496	vesicle transport along microtubule	0.88

Original gene set ID	Original gene set description	Nominal P value
ENSG00000100554	ATP6V1D PPI subnetwork	0.88
GO:0051539	4 iron, 4 sulfur cluster binding	0.88
MP:0000966	decreased sensory neuron number	0.88
GO:0009409	response to cold	0.88
ENSG00000137055	PLAA PPI subnetwork	0.88
GO:0016567	protein ubiquitination	0.88
GO:0016903	oxidoreductase activity, acting on the aldehyde or oxo group of donors	0.88
GO:0019674	NAD metabolic process	0.88
ENSG00000206258	TNXB PPI subnetwork	0.88
ENSG00000198692	EIF1AY PPI subnetwork	0.88
ENSG00000197558	ENSG00000197558 PPI subnetwork	0.88
ENSG00000127388	ENSG00000127388 PPI subnetwork	0.88
ENSG00000161888	SPC24 PPI subnetwork	0.88
ENSG00000181790	BAI1 PPI subnetwork	0.88
ENSG00000166451	CENPN PPI subnetwork	0.88
ENSG00000020922	MRE11A PPI subnetwork	0.89
GO:0046641	positive regulation of alpha-beta T cell proliferation	0.89
GO:0042391	regulation of membrane potential	0.89
GO:0017157	regulation of exocytosis	0.89
GO:0006405	RNA export from nucleus	0.89
ENSG00000100151	PICK1 PPI subnetwork	0.89
GO:0032729	positive regulation of interferon-gamma production	0.89
ENSG00000114812	VIPR1 PPI subnetwork	0.89
GO:0006403	RNA localization	0.89
GO:0030684	preribosome	0.89
ENSG00000169976	SF3B5 PPI subnetwork	0.89
ENSG00000105323	HNRNPUL1 PPI subnetwork	0.89
GO:0045624	positive regulation of T-helper cell differentiation	0.89
ENSG00000127481	UBR4 PPI subnetwork	0.89
MP:0001793	altered susceptibility to infection	0.89
ENSG00000129757	CDKN1C PPI subnetwork	0.89
ENSG00000118194	TNNT2 PPI subnetwork	0.89
GO:0021675	nerve development	0.89
GO:0046356	acetyl-CoA catabolic process	0.89
GO:0048193	Golgi vesicle transport	0.89
GO:0044449	contractile fiber part	0.89
ENSG00000113456	RAD1 PPI subnetwork	0.89
ENSG00000157404	KIT PPI subnetwork	0.89
GO:0030801	positive regulation of cyclic nucleotide metabolic process	0.89
MP:0000774	decreased brain size	0.89
ENSG00000111275	ALDH2 PPI subnetwork	0.89
ENSG00000146963	LUC7L2 PPI subnetwork	0.89
MP:0000137	abnormal vertebrae morphology	0.89
ENSG00000204133	ENSG00000204133 PPI subnetwork	0.89
GO:0051313	attachment of spindle microtubules to chromosome	0.89
MP:0009907	decreased tongue size	0.89
GO:0005003	ephrin receptor activity	0.89
ENSG00000163527	STT3B PPI subnetwork	0.89
GO:0051023	regulation of immunoglobulin secretion	0.89

Original gene set ID	Original gene set description	Nominal P value
ENSG00000196747	HIST1H2AI PPI subnetwork	0.89
ENSG00000196787	HIST1H2AG PPI subnetwork	0.89
ENSG00000196866	HIST1H2AD PPI subnetwork	0.89
ENSG00000184348	HIST1H2AK PPI subnetwork	0.89
ENSG00000198374	HIST1H2AL PPI subnetwork	0.89
ENSG00000198728	LDB1 PPI subnetwork	0.89
GO:0000910	cytokinesis	0.89
ENSG00000103496	STX4 PPI subnetwork	0.89
REACTOME_PLATELET_CALCIUM_HOMEOSTASIS	REACTOME_PLATELET_CALCIUM_HOMEOSTASIS	0.89
MP:0005478	decreased circulating thyroxine level	0.89
MP:0003172	abnormal lysosome physiology	0.89
GO:0016620	oxidoreductase activity, acting on the aldehyde or oxo group of donors, NAD or NAD	0.89
ENSG00000184886	PIGW PPI subnetwork	0.89
ENSG00000159251	ACTC1 PPI subnetwork	0.89
ENSG00000169564	PCBP1 PPI subnetwork	0.89
REACTOME_TRANSPORT_OF_MATURE_MRNA_DERIVED_FROM_AN_INTRON:C	REACTOME_TRANSPORT_OF_MATURE_MRNA_DERIVED_FROM_AN_INTRON:CONT	0.89
GO:0050671	positive regulation of lymphocyte proliferation	0.89
ENSG00000100162	CENPM PPI subnetwork	0.89
ENSG00000198938	MT-CO3 PPI subnetwork	0.89
GO:0008374	O-acyltransferase activity	0.89
MP:0000940	abnormal motor neuron innervation	0.89
GO:0046605	regulation of centrosome cycle	0.89
MP:0001422	abnormal drinking behavior	0.89
GO:0021889	olfactory bulb interneuron differentiation	0.89
REACTOME_FORMATION_OF_THE_HIV:1_EARLY_ELONGATION_COMPLEX	REACTOME_FORMATION_OF_THE_HIV:1_EARLY_ELONGATION_COMPLEX	0.89
REACTOME_FORMATION_OF_THE_EARLY_ELONGATION_COMPLEX	REACTOME_FORMATION_OF_THE_EARLY_ELONGATION_COMPLEX	0.89
ENSG00000156052	GNAQ PPI subnetwork	0.89
GO:0030016	myofibril	0.89
ENSG00000152253	SPC25 PPI subnetwork	0.89
GO:0003156	regulation of organ formation	0.89
ENSG00000087250	MT3 PPI subnetwork	0.89
ENSG00000188312	CENPP PPI subnetwork	0.89
GO:0060428	lung epithelium development	0.89
MP:0002231	abnormal primitive streak morphology	0.89
ENSG00000165119	HNRNPK PPI subnetwork	0.89
REACTOME_ENDOSOMAL_SORTING_COMPLEX_REQUIRED_FOR_TRANSPORT_E	REACTOME_ENDOSOMAL_SORTING_COMPLEX_REQUIRED_FOR_TRANSPORT_ESCR	0.89
GO:0008542	visual learning	0.89
ENSG00000114503	NCBP2 PPI subnetwork	0.89
ENSG00000115163	CENPA PPI subnetwork	0.89
MP:0003324	increased liver adenoma incidence	0.89
GO:0000149	SNARE binding	0.89
REACTOME_CDC6_ASSOCIATION_WITH_THE_ORCORIGIN_COMPLEX	REACTOME_CDC6_ASSOCIATION_WITH_THE_ORCORIGIN_COMPLEX	0.89
ENSG00000110955	ATP5B PPI subnetwork	0.89
GO:0005891	voltage-gated calcium channel complex	0.89
ENSG00000105669	COPE PPI subnetwork	0.89
GO:0001936	regulation of endothelial cell proliferation	0.89
KEGG_PEROXISOME	KEGG_PEROXISOME	0.89
ENSG00000013455	ENSG00000013455 PPI subnetwork	0.89
GO:0050997	quaternary ammonium group binding	0.89

Original gene set ID	Original gene set description	Nominal P value
MP:0004084	abnormal cardiac muscle relaxation	0.89
ENSG00000155974	GRIP1 PPI subnetwork	0.89
GO:0016331	morphogenesis of embryonic epithelium	0.89
ENSG00000115953	ENSG00000115953 PPI subnetwork	0.89
MP:0005192	increased motor neuron number	0.89
MP:0001286	abnormal eye development	0.89
MP:0005106	abnormal incus morphology	0.89
ENSG00000115252	PDE1A PPI subnetwork	0.89
GO:0005834	heterotrimeric G-protein complex	0.89
GO:0006000	fructose metabolic process	0.89
MP:0003271	abnormal duodenum morphology	0.89
GO:0045333	cellular respiration	0.89
ENSG00000136709	WDR33 PPI subnetwork	0.89
KEGG_SNARE_INTERACTIONS_IN_VESICULAR_TRANSPORT	KEGG_SNARE_INTERACTIONS_IN_VESICULAR_TRANSPORT	0.89
GO:0006873	cellular ion homeostasis	0.89
GO:0002208	somatic diversification of immunoglobulins involved in immune response	0.89
GO:0045190	isotype switching	0.89
GO:0002204	somatic recombination of immunoglobulin genes involved in immune response	0.89
GO:0021513	spinal cord dorsal/ventral patterning	0.89
REACTOME_TRANSPORT_OF_MATURE_MRNAS_DERIVED_FROM_INTRONLESS_	REACTOME_TRANSPORT_OF_MATURE_MRNAS_DERIVED_FROM_INTRONLESS_TRA	0.89
REACTOME_THROMBIN_SIGNALLING_THROUGH_PROTEINASE_ACTIVATED_REC	REACTOME_THROMBIN_SIGNALLING_THROUGH_PROTEINASE_ACTIVATED_RECEPT	0.89
KEGG_ARGININE_AND_PROLINE_METABOLISM	KEGG_ARGININE_AND_PROLINE_METABOLISM	0.9
ENSG00000108828	VAT1 PPI subnetwork	0.9
GO:0051082	unfolded protein binding	0.9
GO:0007215	glutamate receptor signaling pathway	0.9
MP:0002766	situs inversus	0.9
GO:0050806	positive regulation of synaptic transmission	0.9
GO:0042288	MHC class I protein binding	0.9
ENSG00000138674	SEC31A PPI subnetwork	0.9
ENSG00000105379	ETFB PPI subnetwork	0.9
ENSG00000187735	TCEA1 PPI subnetwork	0.9
MP:0002696	decreased circulating glucagon level	0.9
MP:0004566	myocardial fiber degeneration	0.9
GO:0007200	phospholipase C-activating G-protein coupled receptor signaling pathway	0.9
MP:0002279	abnormal diaphragm morphology	0.9
GO:0032880	regulation of protein localization	0.9
ENSG00000130066	SAT1 PPI subnetwork	0.9
MP:0005317	increased triglyceride level	0.9
GO:0022904	respiratory electron transport chain	0.9
GO:0006308	DNA catabolic process	0.9
ENSG00000112526	ENSG00000112526 PPI subnetwork	0.9
ENSG00000204256	BRD2 PPI subnetwork	0.9
ENSG00000215077	BRD2 PPI subnetwork	0.9
ENSG00000126698	DNAJC8 PPI subnetwork	0.9
ENSG00000002834	LASP1 PPI subnetwork	0.9
GO:0045063	T-helper 1 cell differentiation	0.9
ENSG00000169371	SNUPN PPI subnetwork	0.9
GO:0048710	regulation of astrocyte differentiation	0.9
GO:0060191	regulation of lipase activity	0.9

Original gene set ID	Original gene set description	Nominal P value
REACTOME_REGULATION_OF_INSULIN_SECRETION	REACTOME_REGULATION_OF_INSULIN_SECRETION	0.9
REACTOME_REGULATION_OF_KIT_SIGNALING	REACTOME_REGULATION_OF_KIT_SIGNALING	0.9
GO:0016460	myosin II complex	0.9
ENSG00000123219	CENPK PPI subnetwork	0.9
GO:0032946	positive regulation of mononuclear cell proliferatior	0.9
ENSG00000198804	MT-CO1 PPI subnetwork	0.9
ENSG00000212875	ENSG00000212875 PPI subnetwork	0.9
GO:0055029	nuclear DNA-directed RNA polymerase complex	0.9
GO:0000428	DNA-directed RNA polymerase complex	0.9
ENSG00000012061	ERCC1 PPI subnetwork	0.9
ENSG00000130429	ARPC1B PPI subnetwork	0.9
GO:0090009	primitive streak formation	0.9
GO:0007212	dopamine receptor signaling pathway	0.9
ENSG00000168172	HOOK3 PPI subnetwork	0.9
GO:0019840	isoprenoid binding	0.9
ENSG00000060688	SNRNP40 PPI subnetwork	0.9
GO:0030850	prostate gland development	0.9
GO:0002827	positive regulation of T-helper 1 type immune response	0.9
ENSG00000181817	LSM10 PPI subnetwork	0.9
GO:0071260	cellular response to mechanical stimulus	0.9
ENSG00000175279	APITD1 PPI subnetwork	0.9
GO:0032273	positive regulation of protein polymerizatior	0.9
ENSG00000052723	SIKE1 PPI subnetwork	0.9
GO:0048038	quinone binding	0.9
ENSG00000138758	SEPT11 PPI subnetwork	0.9
GO:0050906	detection of stimulus involved in sensory perceptior	0.9
ENSG00000183918	SH2D1A PPI subnetwork	0.9
ENSG00000099956	SMARCB1 PPI subnetwork	0.9
MP:0003333	liver fibrosis	0.9
GO:0008307	structural constituent of muscle	0.9
GO:0000045	autophagic vacuole assembly	0.9
GO:0030118	clathrin coat	0.9
GO:0021895	cerebral cortex neuron differentiatior	0.9
REACTOME_TRANSPORT_OF_THE_SLBP_DEPENDANT_MATURE_MRNA	REACTOME_TRANSPORT_OF_THE_SLBP_DEPENDANT_MATURE_MRNA	0.9
GO:0072078	nephron tubule morphogenesis	0.9
MP:0000527	abnormal kidney development	0.9
ENSG00000137812	CASC5 PPI subnetwork	0.9
ENSG00000151725	MLF1IP PPI subnetwork	0.9
ENSG00000143858	SYT2 PPI subnetwork	0.9
ENSG00000183020	AP2A2 PPI subnetwork	0.9
GO:0006612	protein targeting to membrane	0.9
GO:0061333	renal tubule morphogenesis	0.9
ENSG00000100796	SMEK1 PPI subnetwork	0.9
GO:0044282	small molecule catabolic process	0.9
ENSG00000163462	TRIM46 PPI subnetwork	0.9
ENSG00000163468	CCT3 PPI subnetwork	0.9
KEGG_PRIMARY_BILE_ACID_BIOSYNTHESIS	KEGG_PRIMARY_BILE_ACID_BIOSYNTHESIS	0.9
REACTOME_MEIOTIC_RECOMBINATION	REACTOME_MEIOTIC_RECOMBINATION	0.9
GO:0048709	oligodendrocyte differentiation	0.9

Original gene set ID	Original gene set description	Nominal P value
GO:0010243	response to organic nitrogen	0.9
GO:0017085	response to insecticide	0.9
ENSG00000134109	EDEM1 PPI subnetwork	0.9
GO:0046467	membrane lipid biosynthetic process	0.9
ENSG00000114573	ATP6V1A PPI subnetwork	0.9
GO:0072593	reactive oxygen species metabolic process	0.9
KEGG_SULFUR_METABOLISM	KEGG_SULFUR_METABOLISM	0.9
GO:0050881	musculoskeletal movement	0.9
GO:0050879	multicellular organismal movement	0.9
GO:0050953	sensory perception of light stimulus	0.9
ENSG00000169045	HNRNPH1 PPI subnetwork	0.9
ENSG00000096717	SIRT1 PPI subnetwork	0.9
GO:0007601	visual perception	0.9
GO:0005814	centriole	0.9
ENSG00000090989	EXOC1 PPI subnetwork	0.9
GO:0030163	protein catabolic process	0.9
ENSG00000181029	TRAPPC5 PPI subnetwork	0.9
GO:0032993	protein-DNA complex	0.9
ENSG00000184983	NDUFA6 PPI subnetwork	0.9
ENSG00000185658	BRWD1 PPI subnetwork	0.9
REACTOME_TRANSCRIPTION:COUPLED_NER_TC:NER	REACTOME_TRANSCRIPTION:COUPLED_NER_TC:NER	0.9
KEGG_CITRATE_CYCLE_TCA_CYCLE	KEGG_CITRATE_CYCLE_TCA_CYCLE	0.9
ENSG00000057468	MSH4 PPI subnetwork	0.9
ENSG00000147443	DOK2 PPI subnetwork	0.9
GO:0050690	regulation of defense response to virus by virus	0.9
ENSG00000105656	ELL PPI subnetwork	0.9
GO:0051983	regulation of chromosome segregation	0.9
GO:0034440	lipid oxidation	0.9
ENSG00000157344	ENSG00000157344 PPI subnetwork	0.9
ENSG00000105289	TJP3 PPI subnetwork	0.9
ENSG00000198648	STK39 PPI subnetwork	0.9
ENSG00000028137	TNFRSF1B PPI subnetwork	0.9
GO:0015949	nucleobase-containing small molecule interconversion	0.9
REACTOME_G:PROTEIN_ACTIVATION	REACTOME_G:PROTEIN_ACTIVATION	0.9
ENSG00000165023	DIRAS2 PPI subnetwork	0.9
ENSG00000092201	SUPT16H PPI subnetwork	0.9
GO:0051018	protein kinase A binding	0.9
MP:0003463	abnormal single cell response	0.9
ENSG00000128524	ATP6V1F PPI subnetwork	0.9
MP:0000841	abnormal hindbrain morphology	0.9
GO:0033013	tetrapyrrole metabolic process	0.9
GO:0006778	porphyrin-containing compound metabolic process	0.9
KEGG_NUCLEOTIDE_EXCISION_REPAIR	KEGG_NUCLEOTIDE_EXCISION_REPAIR	0.9
ENSG00000213619	NDUFS3 PPI subnetwork	0.91
KEGG_GLYCOSAMINOGLYCAN_BIOSYNTHESIS_KERATAN_SULFATE	KEGG_GLYCOSAMINOGLYCAN_BIOSYNTHESIS_KERATAN_SULFATE	0.91
GO:0048713	regulation of oligodendrocyte differentiation	0.91
GO:0060425	lung morphogenesis	0.91
GO:0015030	Cajal body	0.91
ENSG00000138092	CENPO PPI subnetwork	0.91

Original gene set ID	Original gene set description	Nominal P value
ENSG00000140740	UQCRC2 PPI subnetwork	0.91
GO:0051087	chaperone binding	0.91
MP:0005164	abnormal response to injury	0.91
ENSG00000067836	ROGDI PPI subnetwork	0.91
GO:0060078	regulation of postsynaptic membrane potential	0.91
ENSG00000198744	ENSG00000198744 PPI subnetwork	0.91
ENSG00000072134	EPN2 PPI subnetwork	0.91
ENSG00000101004	NINL PPI subnetwork	0.91
REACTOME_DESTABILIZATION_OF_MRNA_BY_KSRP	REACTOME_DESTABILIZATION_OF_MRNA_BY_KSRP	0.91
ENSG00000166136	NDUFB8 PPI subnetwork	0.91
ENSG00000131100	ATP6V1E1 PPI subnetwork	0.91
ENSG00000145375	SPATA5 PPI subnetwork	0.91
ENSG00000149294	NCAM1 PPI subnetwork	0.91
ENSG00000161270	NPHS1 PPI subnetwork	0.91
ENSG00000100150	DEPDC5 PPI subnetwork	0.91
GO:0051928	positive regulation of calcium ion transport	0.91
GO:0048265	response to pain	0.91
GO:0033177	proton-transporting two-sector ATPase complex, proton-transporting domain	0.91
ENSG00000037965	HOXC8 PPI subnetwork	0.91
ENSG00000113300	CNOT6 PPI subnetwork	0.91
GO:0030730	sequestering of triglyceride	0.91
ENSG00000138041	SMEK2 PPI subnetwork	0.91
GO:0072009	nephron epithelium development	0.91
ENSG00000038274	MAT2B PPI subnetwork	0.91
REACTOME_TRANSPORT_OF_MATURE_TRANSCRIPT_TO_CYTOPLASM	REACTOME_TRANSPORT_OF_MATURE_TRANSCRIPT_TO_CYTOPLASM	0.91
MP:0009767	decreased sensitivity to xenobiotic induced morbidity/mortality	0.91
ENSG00000170144	HNRNPA3 PPI subnetwork	0.91
GO:0045744	negative regulation of G-protein coupled receptor protein signaling pathway	0.91
GO:0006650	glycerophospholipid metabolic process	0.91
ENSG00000196557	CACNA1H PPI subnetwork	0.91
ENSG00000166747	AP1G1 PPI subnetwork	0.91
GO:0038032	termination of G-protein coupled receptor signaling pathway	0.91
GO:0006892	post-Golgi vesicle-mediated transport	0.91
GO:0009948	anterior/posterior axis specification	0.91
GO:0051603	proteolysis involved in cellular protein catabolic process	0.91
ENSG00000094916	CBX5 PPI subnetwork	0.91
GO:0055067	monovalent inorganic cation homeostasis	0.91
ENSG00000167842	MIS12 PPI subnetwork	0.91
ENSG00000149636	DSN1 PPI subnetwork	0.91
ENSG00000099624	ATP5D PPI subnetwork	0.91
GO:0022624	proteasome accessory complex	0.91
KEGG_ASTHMA	KEGG_ASTHMA	0.91
ENSG00000155980	KIF5A PPI subnetwork	0.91
GO:0005802	trans-Golgi network	0.91
GO:0051345	positive regulation of hydrolase activity	0.91
ENSG00000106290	TAF6 PPI subnetwork	0.91
GO:0004890	GABA-A receptor activity	0.91
GO:0034332	adherens junction organization	0.91
ENSG00000139116	KIF21A PPI subnetwork	0.91

Original gene set ID	Original gene set description	Nominal P value
MP:0010392	prolonged QRS complex duration	0.91
ENSG00000008869	HEATR5B PPI subnetwork	0.91
ENSG00000171759	PAH PPI subnetwork	0.91
ENSG00000151834	GABRA2 PPI subnetwork	0.91
GO:0043292	contractile fiber	0.91
GO:0001823	mesonephros development	0.91
GO:0042311	vasodilation	0.91
ENSG00000205339	IPO7 PPI subnetwork	0.91
MP:0003817	abnormal pituitary diverticulum morphology	0.91
MP:0005332	abnormal amino acid level	0.91
GO:0030667	secretory granule membrane	0.91
MP:0000087	absent mandible	0.91
ENSG00000177455	CD19 PPI subnetwork	0.91
GO:0016447	somatic recombination of immunoglobulin gene segments	0.91
GO:0008484	sulfuric ester hydrolase activity	0.91
ENSG00000149503	INCENP PPI subnetwork	0.91
GO:0032886	regulation of microtubule-based process	0.91
ENSG00000113282	CLINT1 PPI subnetwork	0.91
MP:0005402	abnormal action potential	0.91
REACTOME_OPIOID_SIGNALLING	REACTOME_OPIOID_SIGNALLING	0.91
GO:0050708	regulation of protein secretion	0.91
ENSG00000145335	SNCA PPI subnetwork	0.91
MP:0006254	thin cerebral cortex	0.91
ENSG00000115091	ACTR3 PPI subnetwork	0.91
MP:0001292	abnormal lens vesicle development	0.91
GO:0008408	3'-5' exonuclease activity	0.91
GO:0045073	regulation of chemokine biosynthetic process	0.91
GO:0051233	spindle midzone	0.91
REACTOME_NUCLEOTIDE_EXCISION_REPAIR	REACTOME_NUCLEOTIDE_EXCISION_REPAIR	0.91
GO:0002381	immunoglobulin production involved in immunoglobulin mediated immune respons	0.91
REACTOME_GOLGI_ASSOCIATED_VESICLE_BIOGENESIS	REACTOME_GOLGI_ASSOCIATED_VESICLE_BIOGENESIS	0.91
ENSG00000143799	PARP1 PPI subnetwork	0.91
ENSG00000174442	ZWILCH PPI subnetwork	0.91
MP:0008874	decreased physiological sensitivity to xenobiotic	0.91
MP:0004754	abnormal kidney collecting duct morphology	0.91
ENSG00000100401	RANGAP1 PPI subnetwork	0.91
ENSG00000189060	H1F0 PPI subnetwork	0.91
REACTOME_RESOLUTION_OF_ABASIC_SITES_AP_SITES	REACTOME_RESOLUTION_OF_ABASIC_SITES_AP_SITES	0.92
REACTOME_BASE_EXCISION_REPAIR	REACTOME_BASE_EXCISION_REPAIR	0.92
ENSG00000117697	NSL1 PPI subnetwork	0.92
GO:0072243	metanephric nephron epithelium development	0.92
GO:0072234	metanephric nephron tubule development	0.92
GO:0000175	3'-5'-exoribonuclease activity	0.92
REACTOME_REGULATION_OF_ORNITHINE_DECARBOXYLASE_ODC	REACTOME_REGULATION_OF_ORNITHINE_DECARBOXYLASE_ODC	0.92
MP:0001684	abnormal axial mesoderm	0.92
GO:0030880	RNA polymerase complex	0.92
GO:0042402	cellular biogenic amine catabolic process	0.92
ENSG00000159082	SYNJ1 PPI subnetwork	0.92
REACTOME_BILE_ACID_AND_BILE_SALT_METABOLISM	REACTOME_BILE_ACID_AND_BILE_SALT_METABOLISM	0.92

Original gene set ID	Original gene set description	Nominal P value
GO:0006613	cotranslational protein targeting to membrane	0.92
ENSG00000126945	HNRNPH2 PPI subnetwork	0.92
ENSG00000171747	LGALS4 PPI subnetwork	0.92
GO:0002828	regulation of type 2 immune response	0.92
ENSG00000004487	KDM1A PPI subnetwork	0.92
ENSG00000158022	TRIM63 PPI subnetwork	0.92
REACTOME_ABORTIVE_ELONGATION_OF_HIV:1_TRANSCRIPT_IN_THE_ABSENC	REACTOME_ABORTIVE_ELONGATION_OF_HIV:1_TRANSCRIPT_IN_THE_ABSENCE_O	0.92
GO:0051436	negative regulation of ubiquitin-protein ligase activity involved in mitotic cell cycle	0.92
GO:0030317	sperm motility	0.92
ENSG00000198824	CHAMP1 PPI subnetwork	0.92
GO:0048013	ephrin receptor signaling pathway	0.92
GO:0001706	endoderm formation	0.92
ENSG00000100241	SBF1 PPI subnetwork	0.92
GO:0048708	astrocyte differentiation	0.92
ENSG00000184432	COPB2 PPI subnetwork	0.92
GO:0016594	glycine binding	0.92
ENSG00000070182	SPTB PPI subnetwork	0.92
GO:0050657	nucleic acid transport	0.92
GO:0051236	establishment of RNA localization	0.92
GO:0050658	RNA transport	0.92
ENSG00000010818	HIVEP2 PPI subnetwork	0.92
GO:0071158	positive regulation of cell cycle arrest	0.92
ENSG00000122705	CLTA PPI subnetwork	0.92
ENSG00000105258	POLR2I PPI subnetwork	0.92
GO:0042588	zymogen granule	0.92
GO:0070665	positive regulation of leukocyte proliferation	0.92
GO:0005932	microtubule basal body	0.92
GO:0031594	neuromuscular junction	0.92
GO:0016862	intramolecular oxidoreductase activity, interconverting keto- and enol-groups	0.92
GO:0006370	mRNA capping	0.92
ENSG00000132639	SNAP25 PPI subnetwork	0.92
GO:0030900	forebrain development	0.92
GO:0070972	protein localization in endoplasmic reticulum	0.92
ENSG00000115128	ENSG00000115128 PPI subnetwork	0.92
GO:0060079	regulation of excitatory postsynaptic membrane potentia	0.92
GO:0070647	protein modification by small protein conjugation or remova	0.92
KEGG_VIBRIO_CHOLERAE_INFECTION	KEGG_VIBRIO_CHOLERAE_INFECTION	0.92
GO:0016676	oxidoreductase activity, acting on a heme group of donors, oxygen as acceptor	0.92
GO:0015002	heme-copper terminal oxidase activity	0.92
GO:0004129	cytochrome-c oxidase activity	0.92
ENSG00000154710	RABGEF1 PPI subnetwork	0.92
GO:0044257	cellular protein catabolic process	0.92
GO:0016917	GABA receptor activity	0.92
ENSG00000048828	FAM120A PPI subnetwork	0.92
ENSG00000166226	CCT2 PPI subnetwork	0.92
GO:0009264	deoxyribonucleotide catabolic process	0.92
GO:0030313	cell envelope	0.92
GO:0044462	external encapsulating structure part	0.92
ENSG00000154723	ATP5J PPI subnetwork	0.92

Original gene set ID	Original gene set description	Nominal P value
ENSG00000137825	ITPKA PPI subnetwork	0.92
MP:0009838	abnormal sperm axoneme morphology	0.92
ENSG00000164402	SEPT8 PPI subnetwork	0.92
GO:0002637	regulation of immunoglobulin production	0.92
GO:0031334	positive regulation of protein complex assembly	0.92
REACTOME_ACTIVATION_OF_KAINATE_RECEPTORS_UPON_GLUTAMATE_BINDI	REACTOME_ACTIVATION_OF_KAINATE_RECEPTORS_UPON_GLUTAMATE_BINDING	0.92
GO:0001759	organ induction	0.92
ENSG00000109519	GRPEL1 PPI subnetwork	0.92
ENSG00000147099	HDAC8 PPI subnetwork	0.92
ENSG00000100410	PHF5A PPI subnetwork	0.92
ENSG00000160783	PMF1 PPI subnetwork	0.92
ENSG00000142856	ITGB3BP PPI subnetwork	0.92
GO:0072210	metanephric nephron development	0.92
GO:0050433	regulation of catecholamine secretion	0.92
ENSG00000065150	IPO5 PPI subnetwork	0.92
GO:0010959	regulation of metal ion transport	0.92
REACTOME_AMINE_COMPOUND_SLC_TRANSPORTERS	REACTOME_AMINE_COMPOUND_SLC_TRANSPORTERS	0.92
ENSG00000102109	PCSK1N PPI subnetwork	0.92
ENSG00000122952	ZWINT PPI subnetwork	0.92
GO:0043034	costamere	0.92
REACTOME_TRAFFICKING_OF_AMPA_RECEPTORS	REACTOME_TRAFFICKING_OF_AMPA_RECEPTORS	0.92
REACTOME_GLUTAMATE_BINDING_ACTIVATION_OF_AMPA_RECEPTORS_AND_	REACTOME_GLUTAMATE_BINDING_ACTIVATION_OF_AMPA_RECEPTORS_AND_SYN	0.92
GO:0019395	fatty acid oxidation	0.92
GO:0042436	indole-containing compound catabolic process	0.92
GO:0046218	indolalkylamine catabolic process	0.92
GO:0006569	tryptophan catabolic process	0.92
ENSG00000188386	PPP3R2 PPI subnetwork	0.92
MP:0004615	cervical vertebral transformation	0.92
ENSG00000121621	KIF18A PPI subnetwork	0.92
ENSG00000119318	RAD23B PPI subnetwork	0.92
ENSG00000115254	ENSG00000115254 PPI subnetwork	0.92
ENSG00000167136	ENDOG PPI subnetwork	0.92
GO:0016197	endosomal transport	0.92
GO:0016469	proton-transporting two-sector ATPase complex	0.92
ENSG00000060558	GNA15 PPI subnetwork	0.92
MP:0001905	abnormal dopamine level	0.92
ENSG00000129473	BCL2L2 PPI subnetwork	0.92
ENSG00000168496	FEN1 PPI subnetwork	0.92
ENSG00000167083	GNGT2 PPI subnetwork	0.92
REACTOME_GLUCAGON:TYPE_LIGAND_RECEPTORS	REACTOME_GLUCAGON:TYPE_LIGAND_RECEPTORS	0.92
ENSG00000118680	MYL12B PPI subnetwork	0.92
GO:0046503	glycerolipid catabolic process	0.92
ENSG00000124610	HIST1H1A PPI subnetwork	0.92
REACTOME_INSULIN_RECEPTOR_RECYCLING	REACTOME_INSULIN_RECEPTOR_RECYCLING	0.92
GO:0006281	DNA repair	0.92
REACTOME_STRIATED_MUSCLE_CONTRACTION	REACTOME_STRIATED_MUSCLE_CONTRACTION	0.92
GO:0030148	sphingolipid biosynthetic process	0.92
GO:0016054	organic acid catabolic process	0.92
GO:0046395	carboxylic acid catabolic process	0.92

Original gene set ID	Original gene set description	Nominal P value
REACTOME_DARPP:32_EVENTS	REACTOME_DARPP:32_EVENTS	0.92
GO:0006521	regulation of cellular amino acid metabolic process	0.92
MP:0004101	abnormal brain interneuron morphology	0.93
ENSG00000129990	SYT5 PPI subnetwork	0.93
ENSG00000184702	SEPT5 PPI subnetwork	0.93
ENSG00000183474	GTF2H2C PPI subnetwork	0.93
ENSG00000124795	DEK PPI subnetwork	0.93
ENSG00000112290	WASF1 PPI subnetwork	0.93
GO:0009583	detection of light stimulus	0.93
GO:0072073	kidney epithelium development	0.93
GO:0016236	macroautophagy	0.93
MP:0003672	abnormal ureter development	0.93
ENSG00000159259	CHAF1B PPI subnetwork	0.93
ENSG00000143368	SF3B4 PPI subnetwork	0.93
GO:0006721	terpenoid metabolic process	0.93
REACTOME_IRON_UPTAKE_AND_TRANSPORT	REACTOME_IRON_UPTAKE_AND_TRANSPORT	0.93
ENSG00000153207	AHCTF1 PPI subnetwork	0.93
GO:0032446	protein modification by small protein conjugation	0.93
GO:0044447	axoneme part	0.93
GO:0007626	locomotory behavior	0.93
GO:0042033	chemokine biosynthetic process	0.93
GO:0005245	voltage-gated calcium channel activity	0.93
REACTOME_INHIBITION_OF_INSULIN_SECRETION_BY_ADRENALINENORADREN/	REACTOME_INHIBITION_OF_INSULIN_SECRETION_BY_ADRENALINENORADRENALIN	0.93
ENSG00000124172	ATP5E PPI subnetwork	0.93
ENSG00000025800	KPNA6 PPI subnetwork	0.93
REACTOME_TRYPTOPHAN_CATABOLISM	REACTOME_TRYPTOPHAN_CATABOLISM	0.93
ENSG00000184357	HIST1H1B PPI subnetwork	0.93
GO:0050905	neuromuscular process	0.93
ENSG00000138071	ACTR2 PPI subnetwork	0.93
REACTOME_ENERGY_DEPENDENT_REGULATION_OF_MTOR_BY_LKB1:AMPK	REACTOME_ENERGY_DEPENDENT_REGULATION_OF_MTOR_BY_LKB1:AMPK	0.93
MP:0001898	abnormal long term depression	0.93
KEGG_GLYOXYLATE_AND_DICARBOXYLATE_METABOLISM	KEGG_GLYOXYLATE_AND_DICARBOXYLATE_METABOLISM	0.93
GO:0006289	nucleotide-excision repair	0.93
ENSG00000163636	PSMD6 PPI subnetwork	0.93
ENSG00000168556	ING2 PPI subnetwork	0.93
GO:0030594	neurotransmitter receptor activity	0.93
GO:0000775	chromosome, centromeric region	0.93
ENSG00000166337	TAF10 PPI subnetwork	0.93
REACTOME_SYNTHESIS_AND_INTERCONVERSION_OF_NUCLEOTIDE_DI:_AND_T	REACTOME_SYNTHESIS_AND_INTERCONVERSION_OF_NUCLEOTIDE_DI:_AND_TRIPI	0.93
GO:0007413	axonal fasciculation	0.93
ENSG00000143850	PLEKHA6 PPI subnetwork	0.93
REACTOME_INSULIN_SYNTHESIS_AND_PROCESSING	REACTOME_INSULIN_SYNTHESIS_AND_PROCESSING	0.93
GO:0032838	cell projection cytoplasm	0.93
ENSG00000106588	PSMA2 PPI subnetwork	0.93
MP:0000750	abnormal muscle regeneration	0.93
ENSG00000198947	DMD PPI subnetwork	0.93
GO:0034453	microtubule anchoring	0.93
GO:0042573	retinoic acid metabolic process	0.93
ENSG00000133059	DSTYK PPI subnetwork	0.93

Original gene set ID	Original gene set description	Nominal P value
GO:0016829	lyase activity	0.93
GO:0009055	electron carrier activity	0.93
GO:0009620	response to fungus	0.93
MP:0002239	abnormal nasal septum morphology	0.93
GO:0004221	ubiquitin thiolesterase activity	0.93
GO:0010888	negative regulation of lipid storage	0.93
GO:0051287	NAD binding	0.93
MP:0004452	abnormal pterygoid process morphology	0.93
GO:0006120	mitochondrial electron transport, NADH to ubiquinone	0.93
ENSG00000088832	FKBP1A PPI subnetwork	0.93
GO:0051298	centrosome duplication	0.93
REACTOME_ACTIVATION_OF_CA:PERMEABLE_KAINATE_RECEPTOR	REACTOME_ACTIVATION_OF_CA:PERMEABLE_KAINATE_RECEPTOR	0.93
REACTOME_IONOTROPIC_ACTIVITY_OF_KAINATE_RECEPTORS	REACTOME_IONOTROPIC_ACTIVITY_OF_KAINATE_RECEPTORS	0.93
ENSG00000087263	OGFOD1 PPI subnetwork	0.93
ENSG00000100985	MMP9 PPI subnetwork	0.93
REACTOME_METABOLISM_OF_AMINO_ACIDS_AND_DERIVATIVES	REACTOME_METABOLISM_OF_AMINO_ACIDS_AND_DERIVATIVES	0.93
ENSG00000103423	DNAJA3 PPI subnetwork	0.93
ENSG00000172977	KAT5 PPI subnetwork	0.93
REACTOME_CELL_CYCLE_CHECKPOINTS	REACTOME_CELL_CYCLE_CHECKPOINTS	0.93
ENSG00000105726	ATP13A1 PPI subnetwork	0.93
ENSG00000089818	NECAP1 PPI subnetwork	0.93
GO:0006081	cellular aldehyde metabolic process	0.94
ENSG00000165868	HSPA12A PPI subnetwork	0.94
GO:0072283	metanephric renal vesicle morphogenesis	0.94
MP:0003054	spina bifida	0.94
ENSG00000105514	RAB3D PPI subnetwork	0.94
GO:0072170	metanephric tubule development	0.94
GO:0016706	oxidoreductase activity, acting on paired donors, with incorporation or reduction of	0.94
ENSG00000137575	SDCBP PPI subnetwork	0.94
ENSG00000185973	TMLHE PPI subnetwork	0.94
GO:0009063	cellular amino acid catabolic process	0.94
MP:0003137	abnormal impulse conducting system conduction	0.94
ENSG00000173366	ENSG00000173366 PPI subnetwork	0.94
GO:0030878	thyroid gland development	0.94
GO:0010043	response to zinc ion	0.94
GO:0006568	tryptophan metabolic process	0.94
GO:0016796	exonuclease activity, active with either ribo- or deoxyribonucleic acids and producin	0.94
GO:0007611	learning or memory	0.94
KEGG_RNA_DEGRADATION	KEGG_RNA_DEGRADATION	0.94
GO:0000783	nuclear telomere cap complex	0.94
GO:0000782	telomere cap complex	0.94
ENSG00000100167	SEPT3 PPI subnetwork	0.94
GO:0016574	histone ubiquitination	0.94
MP:0002090	abnormal vision	0.94
ENSG00000006740	ARHGAP44 PPI subnetwork	0.94
ENSG00000143106	PSMA5 PPI subnetwork	0.94
ENSG00000101654	RNMT PPI subnetwork	0.94
ENSG00000125814	NAPB PPI subnetwork	0.94
ENSG00000185104	FAF1 PPI subnetwork	0.94

Original gene set ID	Original gene set description	Nominal P value
GO:0031023	microtubule organizing center organization	0.94
GO:0030968	endoplasmic reticulum unfolded protein response	0.94
GO:0034620	cellular response to unfolded protein	0.94
ENSG00000120265	PCMT1 PPI subnetwork	0.94
ENSG00000169217	CD2BP2 PPI subnetwork	0.94
GO:0055093	response to hyperoxia	0.94
ENSG00000197299	BLM PPI subnetwork	0.94
REACTOME_METABOLISM_OF_PORPHYRINS	REACTOME_METABOLISM_OF_PORPHYRINS	0.94
GO:0001655	urogenital system development	0.94
ENSG00000107554	DNMBP PPI subnetwork	0.94
GO:0021954	central nervous system neuron development	0.94
ENSG00000125870	SNRPB2 PPI subnetwork	0.94
ENSG00000105509	HAS1 PPI subnetwork	0.94
REACTOME_HIV_INFECTION	REACTOME_HIV_INFECTION	0.94
REACTOME_REGULATION_OF_AMPK_ACTIVITY_VIA_LKB1	REACTOME_REGULATION_OF_AMPK_ACTIVITY_VIA_LKB1	0.94
REACTOME_ACTIVATION_OF_GENES_BY_ATF4	REACTOME_ACTIVATION_OF_GENES_BY_ATF4	0.94
MP:0000830	abnormal diencephalon morphology	0.94
ENSG00000181555	SETD2 PPI subnetwork	0.94
GO:0048168	regulation of neuronal synaptic plasticity	0.94
ENSG00000066248	NGEF PPI subnetwork	0.94
GO:0048557	embryonic digestive tract morphogenesis	0.94
ENSG00000172732	MUS81 PPI subnetwork	0.94
ENSG00000072864	NDE1 PPI subnetwork	0.94
GO:0031646	positive regulation of neurological system process	0.94
ENSG00000188486	H2AFX PPI subnetwork	0.94
GO:0009310	amine catabolic process	0.94
GO:0008066	glutamate receptor activity	0.94
GO:0001822	kidney development	0.94
ENSG00000065833	ME1 PPI subnetwork	0.94
ENSG00000164687	FABP5 PPI subnetwork	0.94
GO:0051297	centrosome organization	0.94
MP:0005533	increased body temperature	0.94
ENSG00000161057	PSMC2 PPI subnetwork	0.94
ENSG00000115808	STRN PPI subnetwork	0.94
GO:0060193	positive regulation of lipase activity	0.94
ENSG00000205726	ITSN1 PPI subnetwork	0.94
KEGG_NEUROACTIVE_LIGAND_RECEPTOR_INTERACTION	KEGG_NEUROACTIVE_LIGAND_RECEPTOR_INTERACTION	0.94
GO:0045295	gamma-catenin binding	0.94
ENSG00000115760	BIRC6 PPI subnetwork	0.94
MP:0003384	abnormal ventral body wall morphology	0.94
REACTOME_PACKAGING_OF_TELOMERE_ENDS	REACTOME_PACKAGING_OF_TELOMERE_ENDS	0.94
ENSG00000183091	NEB PPI subnetwork	0.94
GO:0072001	renal system development	0.94
ENSG00000107611	CUBN PPI subnetwork	0.94
MP:0008789	abnormal olfactory epithelium morphology	0.94
GO:0055072	iron ion homeostasis	0.94
MP:0001787	pericardial edema	0.94
MP:0001777	abnormal body temperature homeostasis	0.94
MP:0003063	increased coping response	0.94

Original gene set ID	Original gene set description	Nominal P value
GO:0015931	nucleobase-containing compound transport	0.94
ENSG00000138413	IDH1 PPI subnetwork	0.94
ENSG00000152455	SUV39H2 PPI subnetwork	0.94
GO:0010470	regulation of gastrulation	0.94
GO:0007617	mating behavior	0.95
GO:0000786	nucleosome	0.95
GO:0009072	aromatic amino acid family metabolic process	0.95
GO:0033176	proton-transporting V-type ATPase complex	0.95
GO:0008328	ionotropic glutamate receptor complex	0.95
ENSG00000131558	EXOC4 PPI subnetwork	0.95
GO:0051653	spindle localization	0.95
GO:0051293	establishment of spindle localization	0.95
MP:0011448	decreased dopaminergic neuron number	0.95
ENSG00000162928	PEX13 PPI subnetwork	0.95
ENSG00000107819	SFXN3 PPI subnetwork	0.95
GO:0005234	extracellular-glutamate-gated ion channel activity	0.95
ENSG00000058262	SEC61A1 PPI subnetwork	0.95
MP:0001526	abnormal placing response	0.95
ENSG00000092108	SCFD1 PPI subnetwork	0.95
GO:0043523	regulation of neuron apoptotic process	0.95
ENSG00000108387	SEPT4 PPI subnetwork	0.95
ENSG00000100146	SOX10 PPI subnetwork	0.95
GO:0048019	receptor antagonist activity	0.95
GO:0030547	receptor inhibitor activity	0.95
REACTOME_CDT1_ASSOCIATION_WITH_THE_CDC6ORCORIGIN_COMPLEX	REACTOME_CDT1_ASSOCIATION_WITH_THE_CDC6ORCORIGIN_COMPLEX	0.95
GO:0008306	associative learning	0.95
REACTOME_NACL:_DEPENDENT_NEUROTRANSMITTER_TRANSPORTERS	REACTOME_NACL:_DEPENDENT_NEUROTRANSMITTER_TRANSPORTERS	0.95
GO:0051402	neuron apoptotic process	0.95
ENSG00000170734	POLH PPI subnetwork	0.95
ENSG00000025772	TOMM34 PPI subnetwork	0.95
GO:0050871	positive regulation of B cell activation	0.95
ENSG00000126457	PRMT1 PPI subnetwork	0.95
GO:0043178	alcohol binding	0.95
GO:0060601	lateral sprouting from an epithelium	0.95
GO:0009434	microtubule-based flagellum	0.95
GO:0048704	embryonic skeletal system morphogenesis	0.95
ENSG00000167641	PPP1R14A PPI subnetwork	0.95
GO:0005231	excitatory extracellular ligand-gated ion channel activity	0.95
GO:0016651	oxidoreductase activity, acting on NADH or NADPH	0.95
GO:0022408	negative regulation of cell-cell adhesion	0.95
GO:0031080	Nup107-160 complex	0.95
ENSG00000105402	NAPA PPI subnetwork	0.95
GO:0048368	lateral mesoderm development	0.95
GO:0070528	protein kinase C signaling cascade	0.95
ENSG00000184752	NDUFA12 PPI subnetwork	0.95
KEGG_HUNTINGTONS_DISEASE	KEGG_HUNTINGTONS_DISEASE	0.95
MP:0002842	increased systemic arterial blood pressure	0.95
ENSG00000085832	EPS15 PPI subnetwork	0.95
ENSG00000030304	MUSK PPI subnetwork	0.95

Original gene set ID	Original gene set description	Nominal P value
ENSG00000189043	NDUFA4 PPI subnetwork	0.95
ENSG00000180817	PPA1 PPI subnetwork	0.95
GO:0003678	DNA helicase activity	0.95
GO:0006775	fat-soluble vitamin metabolic process	0.95
ENSG00000088320	REM1 PPI subnetwork	0.95
ENSG00000022355	GABRA1 PPI subnetwork	0.95
ENSG00000167491	GATAD2A PPI subnetwork	0.95
GO:0014075	response to amine stimulus	0.95
ENSG00000183765	CHEK2 PPI subnetwork	0.95
ENSG00000169189	NSMCE1 PPI subnetwork	0.95
MP:0004736	abnormal distortion product otoacoustic emission	0.95
GO:0010518	positive regulation of phospholipase activity	0.95
ENSG00000149136	SSRP1 PPI subnetwork	0.95
ENSG00000169925	BRD3 PPI subnetwork	0.95
GO:0072087	renal vesicle development	0.95
REACTOME_SYNTHESIS_SECRETION_AND_INACTIVATION_OF_GLUCOSE:DEPEN	REACTOME_SYNTHESIS_SECRETION_AND_INACTIVATION_OF_GLUCOSE:DEPENDEN	0.95
GO:0031234	extrinsic to internal side of plasma membrane	0.95
GO:0032982	myosin filament	0.95
GO:0060249	anatomical structure homeostasis	0.95
ENSG00000182473	EXOC7 PPI subnetwork	0.95
ENSG00000179091	CYC1 PPI subnetwork	0.95
GO:0031123	RNA 3'-end processing	0.95
GO:0021772	olfactory bulb development	0.95
GO:0021988	olfactory lobe development	0.95
GO:0006099	tricarboxylic acid cycle	0.95
ENSG00000173660	UQCRH PPI subnetwork	0.95
GO:0048024	regulation of nuclear mRNA splicing, via spliceosome	0.95
ENSG00000172757	CFL1 PPI subnetwork	0.95
GO:0050890	cognition	0.95
GO:0070997	neuron death	0.95
GO:0014013	regulation of gliogenesis	0.95
GO:0016411	acylglycerol O-acyltransferase activity	0.95
MP:0006007	abnormal basal ganglion morphology	0.95
ENSG00000090372	STRN4 PPI subnetwork	0.95
GO:0017137	Rab GTPase binding	0.95
REACTOME_POST:ELONGATION_PROCESSING_OF_INTRON:CONTAINING_PRE:N	REACTOME_POST:ELONGATION_PROCESSING_OF_INTRON:CONTAINING_PRE:MRN	0.95
REACTOME_MRNA_3:END_PROCESSING	REACTOME_MRNA_3:END_PROCESSING	0.95
GO:0072088	nephron epithelium morphogenesis	0.95
REACTOME_G_ALPHA_Z_SIGNALLING_EVENTS	REACTOME_G_ALPHA_Z_SIGNALLING_EVENTS	0.95
REACTOME_DUAL_INCISION_REACTION_IN_TC:NER	REACTOME_DUAL_INCISION_REACTION_IN_TC:NER	0.95
REACTOME_FORMATION_OF_TRANSCRIPTION:COUPLED_NER_TC:NER_REPAIR_	REACTOME_FORMATION_OF_TRANSCRIPTION:COUPLED_NER_TC:NER_REPAIR_CO	0.95
ENSG00000087302	C14orf166 PPI subnetwork	0.95
GO:0001975	response to amphetamine	0.95
GO:0045454	cell redox homeostasis	0.95
GO:0072329	monocarboxylic acid catabolic process	0.95
ENSG00000130479	MAP1S PPI subnetwork	0.95
MP:0002938	white spotting	0.95
ENSG00000049245	VAMP3 PPI subnetwork	0.96
GO:0042168	heme metabolic process	0.96

Original gene set ID	Original gene set description	Nominal P value
REACTOME_TRANSFERRIN_ENDOCYTOSIS_AND_RECYCLING	REACTOME_TRANSFERRIN_ENDOCYTOSIS_AND_RECYCLING	0.96
MP:0004624	abnormal thoracic cage morphology	0.96
ENSG00000101182	PSMA7 PPI subnetwork	0.96
GO:0007270	neuron-neuron synaptic transmission	0.96
MP:0006054	spinal hemorrhage	0.96
GO:0051937	catecholamine transport	0.96
ENSG00000141543	EIF4A3 PPI subnetwork	0.96
ENSG00000134057	CCNB1 PPI subnetwork	0.96
GO:0019861	flagellum	0.96
REACTOME_RNA_POL_II_CTD_PHOSPHORYLATION_AND_INTERACTION_WITH_	REACTOME_RNA_POL_II_CTD_PHOSPHORYLATION_AND_INTERACTION_WITH_CE	0.96
GO:0030288	outer membrane-bounded periplasmic space	0.96
GO:0042597	periplasmic space	0.96
GO:0072077	renal vesicle morphogenesis	0.96
REACTOME_NEF_MEDIATED_DOWNREGULATION_OF_MHC_CLASS_I_COMPLEX	REACTOME_NEF_MEDIATED_DOWNREGULATION_OF_MHC_CLASS_I_COMPLEX_CE	0.96
ENSG00000163464	CXCR1 PPI subnetwork	0.96
GO:0042379	chemokine receptor binding	0.96
ENSG00000065057	NTHL1 PPI subnetwork	0.96
ENSG00000169021	UQCRFS1 PPI subnetwork	0.96
KEGG_VALINE_LEUCINE_AND_ISOLEUCINE_DEGRADATION	KEGG_VALINE_LEUCINE_AND_ISOLEUCINE_DEGRADATION	0.96
GO:0022900	electron transport chain	0.96
GO:0016358	dendrite development	0.96
GO:0072006	nephron development	0.96
ENSG00000166411	IDH3A PPI subnetwork	0.96
ENSG00000125356	NDUFA1 PPI subnetwork	0.96
GO:0072028	nephron morphogenesis	0.96
MP:0002804	abnormal motor learning	0.96
ENSG00000144848	ATG3 PPI subnetwork	0.96
ENSG00000125944	HNRNPR PPI subnetwork	0.96
KEGG_PARKINSONS_DISEASE	KEGG_PARKINSONS_DISEASE	0.96
GO:0014003	oligodendrocyte development	0.96
GO:0043269	regulation of ion transport	0.96
ENSG00000111445	RFC5 PPI subnetwork	0.96
GO:0031907	microbody lumen	0.96
GO:0005782	peroxisomal matrix	0.96
ENSG00000147133	TAF1 PPI subnetwork	0.96
GO:0031625	ubiquitin protein ligase binding	0.96
ENSG00000164683	HEY1 PPI subnetwork	0.96
GO:0042572	retinol metabolic process	0.96
ENSG00000145864	GABRB2 PPI subnetwork	0.96
ENSG00000170906	NDUFA3 PPI subnetwork	0.96
MP:0003964	abnormal noradrenaline level	0.96
GO:0048475	coated membrane	0.96
GO:0030117	membrane coat	0.96
MP:0000061	fragile skeleton	0.96
REACTOME_PROCESSING_OF_CAPPED_INTRONLESS_PRE:MRNA	REACTOME_PROCESSING_OF_CAPPED_INTRONLESS_PRE:MRNA	0.96
REACTOME_POST:ELONGATION_PROCESSING_OF_INTRONLESS_PRE:MRNA	REACTOME_POST:ELONGATION_PROCESSING_OF_INTRONLESS_PRE:MRNA	0.96
ENSG00000184270	HIST2H2AB PPI subnetwork	0.96
ENSG00000071564	TCF3 PPI subnetwork	0.96
GO:0045685	regulation of glial cell differentiation	0.96

Original gene set ID	Original gene set description	Nominal P value
GO:0008343	adult feeding behavior	0.96
GO:0051971	positive regulation of transmission of nerve impulse	0.96
GO:0022406	membrane docking	0.96
MP:0006090	abnormal utricle morphology	0.96
MP:0009453	enhanced contextual conditioning behavior	0.96
ENSG00000090266	NDUFB2 PPI subnetwork	0.96
ENSG00000115590	IL1R2 PPI subnetwork	0.96
GO:0090130	tissue migration	0.96
GO:0044450	microtubule organizing center part	0.96
GO:0034976	response to endoplasmic reticulum stress	0.96
ENSG00000112685	EXOC2 PPI subnetwork	0.96
GO:0048806	genitalia development	0.96
GO:0001756	somitogenesis	0.96
ENSG00000184445	KNTC1 PPI subnetwork	0.96
REACTOME_CITRIC_ACID_CYCLE_TCA_CYCLE	REACTOME_CITRIC_ACID_CYCLE_TCA_CYCLE	0.96
MP:0000285	abnormal heart valve morphology	0.96
GO:0007585	respiratory gaseous exchange	0.96
GO:0031124	mRNA 3'-end processing	0.96
MP:0001982	decreased chemically-elicited antinociception	0.96
GO:0033555	multicellular organismal response to stress	0.96
GO:0034599	cellular response to oxidative stress	0.96
GO:0006888	ER to Golgi vesicle-mediated transport	0.96
GO:0072075	metanephric mesenchyme development	0.96
GO:0010453	regulation of cell fate commitment	0.96
GO:0042775	mitochondrial ATP synthesis coupled electron transport	0.96
GO:0042773	ATP synthesis coupled electron transport	0.96
GO:0006733	oxidoreduction coenzyme metabolic process	0.96
ENSG00000107758	PPP3CB PPI subnetwork	0.96
GO:0031424	keratinization	0.96
GO:0004532	exoribonuclease activity	0.96
GO:0006936	muscle contraction	0.96
GO:0042162	telomeric DNA binding	0.96
GO:0072273	metanephric nephron morphogenesis	0.96
MP:0003425	abnormal optic vesicle formation	0.96
REACTOME_VIRAL_MESSENGER_RNA_SYNTHESIS	REACTOME_VIRAL_MESSENGER_RNA_SYNTHESIS	0.96
GO:0001657	ureteric bud development	0.96
GO:0003338	metanephros morphogenesis	0.96
GO:0009612	response to mechanical stimulus	0.96
ENSG00000169242	EFNA1 PPI subnetwork	0.96
GO:0030048	actin filament-based movement	0.96
ENSG00000103479	RBL2 PPI subnetwork	0.96
REACTOME_THE_CITRIC_ACID_TCA_CYCLE_AND_RESPIRATORY_ELECTRON_TRA	REACTOME_THE_CITRIC_ACID_TCA_CYCLE_AND_RESPIRATORY_ELECTRON_TRANSF	0.96
GO:0060688	regulation of morphogenesis of a branching structure	0.96
GO:0072524	pyridine-containing compound metabolic process	0.96
GO:0019362	pyridine nucleotide metabolic process	0.96
GO:0004970	ionotropic glutamate receptor activity	0.96
GO:0016646	oxidoreductase activity, acting on the CH-NH group of donors, NAD or NADP as acce	0.96
GO:0005859	muscle myosin complex	0.96
ENSG00000165288	BRWD3 PPI subnetwork	0.96

Original gene set ID	Original gene set description	Nominal P value
GO:0021984	adenohypophysis development	0.96
GO:0009713	catechol-containing compound biosynthetic process	0.97
GO:0042423	catecholamine biosynthetic process	0.97
GO:0034312	diol biosynthetic process	0.97
GO:0007202	activation of phospholipase C activity	0.97
GO:0010039	response to iron ion	0.97
ENSG00000036257	CUL3 PPI subnetwork	0.97
GO:0035967	cellular response to topologically incorrect protein	0.97
MP:0001961	abnormal reflex	0.97
MP:0004859	abnormal synaptic plasticity	0.97
GO:0030261	chromosome condensation	0.97
ENSG00000063245	EPN1 PPI subnetwork	0.97
GO:0005227	calcium activated cation channel activity	0.97
MP:0000149	abnormal scapula morphology	0.97
GO:0009452	RNA capping	0.97
GO:0043928	exonucleolytic nuclear-transcribed mRNA catabolic process involved in deadenylatio	0.97
GO:0000291	nuclear-transcribed mRNA catabolic process, exonucleolytic	0.97
ENSG00000023228	NDUFS1 PPI subnetwork	0.97
ENSG00000009413	REV3L PPI subnetwork	0.97
GO:0006004	fucose metabolic process	0.97
GO:0048169	regulation of long-term neuronal synaptic plasticity	0.97
MP:0001299	abnormal eye distance/ position	0.97
ENSG00000135940	COX5B PPI subnetwork	0.97
GO:0030219	megakaryocyte differentiation	0.97
ENSG00000151729	SLC25A4 PPI subnetwork	0.97
ENSG00000120656	TAF12 PPI subnetwork	0.97
MP:0002741	small olfactory bulb	0.97
GO:0045168	cell-cell signaling involved in cell fate commitment	0.97
ENSG00000124575	HIST1H1D PPI subnetwork	0.97
GO:0030145	manganese ion binding	0.97
GO:0030308	negative regulation of cell growth	0.97
GO:0005721	centromeric heterochromatin	0.97
GO:0007030	Golgi organization	0.97
REACTOME_PERK_REGULATED_GENE_EXPRESSION	REACTOME_PERK_REGULATED_GENE_EXPRESSION	0.97
MP:0004632	abnormal cochlear OHC efferent innervation pattern	0.97
GO:0051149	positive regulation of muscle cell differentiation	0.97
MP:0003031	acidosis	0.97
ENSG00000160075	SSU72 PPI subnetwork	0.97
MP:0000759	abnormal skeletal muscle morphology	0.97
MP:0000897	abnormal midbrain morphology	0.97
ENSG00000145216	FIP1L1 PPI subnetwork	0.97
GO:0035282	segmentation	0.97
ENSG00000175305	CCNE2 PPI subnetwork	0.97
ENSG00000163939	PBRM1 PPI subnetwork	0.97
GO:0048278	vesicle docking	0.97
MP:0001629	abnormal heart rate	0.97
ENSG00000120008	WDR11 PPI subnetwork	0.97
KEGG_HISTIDINE_METABOLISM	KEGG_HISTIDINE_METABOLISM	0.97
GO:0007379	segment specification	0.97

Original gene set ID	Original gene set description	Nominal P value
ENSG00000116903	EXOC8 PPI subnetwork	0.97
MP:0004180	failure of initiation of embryo turning	0.97
GO:0010863	positive regulation of phospholipase C activity	0.97
GO:0042375	quinone cofactor metabolic process	0.97
GO:0072074	kidney mesenchyme development	0.97
GO:0000132	establishment of mitotic spindle orientation	0.97
GO:0051294	establishment of spindle orientation	0.97
ENSG00000130288	ENSG00000130288 PPI subnetwork	0.97
GO:0033238	regulation of cellular amine metabolic process	0.97
KEGG_REGULATION_OF_AUTOPHAGY	KEGG_REGULATION_OF_AUTOPHAGY	0.97
ENSG00000197373	ENSG00000197373 PPI subnetwork	0.97
ENSG00000168255	POLR2J3 PPI subnetwork	0.97
GO:0060993	kidney morphogenesis	0.97
ENSG00000102977	ACD PPI subnetwork	0.97
ENSG00000139132	FGD4 PPI subnetwork	0.97
GO:0043270	positive regulation of ion transport	0.97
GO:0046496	nicotinamide nucleotide metabolic process	0.97
GO:0051925	regulation of calcium ion transport via voltage-gated calcium channel activity	0.97
ENSG00000100968	NFATC4 PPI subnetwork	0.97
GO:0031640	killing of cells of other organism	0.97
ENSG00000196628	TCF4 PPI subnetwork	0.97
GO:0042659	regulation of cell fate specification	0.97
ENSG00000145041	VPRBP PPI subnetwork	0.97
ENSG00000125968	ID1 PPI subnetwork	0.97
ENSG00000172288	CDY1 PPI subnetwork	0.97
ENSG00000172352	CDY1B PPI subnetwork	0.97
GO:0042417	dopamine metabolic process	0.97
GO:0070469	respiratory chain	0.97
GO:0007612	learning	0.97
GO:0010517	regulation of phospholipase activity	0.97
GO:0045778	positive regulation of ossification	0.97
GO:0005746	mitochondrial respiratory chain	0.97
GO:0000242	pericentriolar material	0.97
GO:0042745	circadian sleep/wake cycle	0.97
ENSG00000067829	IDH3G PPI subnetwork	0.97
GO:0006783	heme biosynthetic process	0.97
GO:0016896	exoribonuclease activity, producing 5'-phosphomonoesters	0.97
GO:0050832	defense response to fungus	0.98
ENSG00000119888	EPCAM PPI subnetwork	0.98
GO:0000940	condensed chromosome outer kinetochore	0.98
GO:0001656	metanephros development	0.98
REACTOME_MRNA_CAPPING	REACTOME_MRNA_CAPPING	0.98
ENSG00000206282	RGL2 PPI subnetwork	0.98
ENSG00000206210	ENSG00000206210 PPI subnetwork	0.98
ENSG00000164032	H2AFZ PPI subnetwork	0.98
GO:0009566	fertilization	0.98
GO:0031128	developmental induction	0.98
ENSG00000004779	NDUFAB1 PPI subnetwork	0.98
GO:0045471	response to ethanol	0.98

Original gene set ID	Original gene set description	Nominal P value
GO:0002825	regulation of T-helper 1 type immune response	0.98
MP:0002953	thick ventricular wall	0.98
ENSG00000204120	GIGYF2 PPI subnetwork	0.98
ENSG00000167792	NDUFV1 PPI subnetwork	0.98
REACTOME_DEPOSITION_OF_NEW_CENPA:CONTAINING_NUCLEOSOMES_AT_T	REACTOME_DEPOSITION_OF_NEW_CENPA:CONTAINING_NUCLEOSOMES_AT_THE_	0.98
REACTOME_NUCLEOSOME_ASSEMBLY	REACTOME_NUCLEOSOME_ASSEMBLY	0.98
ENSG00000164919	COX6C PPI subnetwork	0.98
GO:0040001	establishment of mitotic spindle localization	0.98
GO:0003401	axis elongation	0.98
GO:0043524	negative regulation of neuron apoptotic process	0.98
GO:0043176	amine binding	0.98
GO:0009062	fatty acid catabolic process	0.98
ENSG00000136888	ATP6V1G1 PPI subnetwork	0.98
ENSG00000010244	ZNF207 PPI subnetwork	0.98
GO:0016835	carbon-oxygen lyase activity	0.98
KEGG_CARDIAC_MUSCLE_CONTRACTION	KEGG_CARDIAC_MUSCLE_CONTRACTION	0.98
ENSG00000140416	TPM1 PPI subnetwork	0.98
GO:0060572	morphogenesis of an epithelial bud	0.98
ENSG00000065534	MYLK PPI subnetwork	0.98
ENSG00000167306	MYO5B PPI subnetwork	0.98
GO:0031593	polyubiquitin binding	0.98
MP:0004725	decreased platelet serotonin level	0.98
GO:0072527	pyrimidine-containing compound metabolic process	0.98
MP:0006065	abnormal heart position or orientation	0.98
ENSG00000164258	NDUFS4 PPI subnetwork	0.98
MP:0009232	abnormal sperm nucleus morphology	0.98
GO:0010632	regulation of epithelial cell migration	0.98
GO:0051924	regulation of calcium ion transport	0.98
MP:0004215	abnormal myocardial fiber physiology	0.98
ENSG00000186230	ZNF749 PPI subnetwork	0.98
GO:0010824	regulation of centrosome duplication	0.98
ENSG00000187555	USP7 PPI subnetwork	0.98
ENSG00000215697	ENSG00000215697 PPI subnetwork	0.98
ENSG00000168397	ATG4B PPI subnetwork	0.98
GO:0071241	cellular response to inorganic substance	0.98
GO:0015939	pantothenate metabolic process	0.98
MP:0008412	increased cellular sensitivity to oxidative stress	0.98
ENSG00000173894	CBX2 PPI subnetwork	0.98
GO:0060675	ureteric bud morphogenesis	0.98
REACTOME_CLEAVAGE_OF_GROWING_TRANSCRIPT_IN_THE_TERMINATION_RI	REACTOME_CLEAVAGE_OF_GROWING_TRANSCRIPT_IN_THE_TERMINATION_REGIC	0.98
REACTOME_RNA_POLYMERASE_II_TRANSCRIPTION_TERMINATION	REACTOME_RNA_POLYMERASE_II_TRANSCRIPTION_TERMINATION	0.98
REACTOME_POST:ELONGATION_PROCESSING_OF_THE_TRANSCRIPT	REACTOME_POST:ELONGATION_PROCESSING_OF_THE_TRANSCRIPT	0.98
ENSG00000032514	ENSG00000032514 PPI subnetwork	0.98
ENSG00000119013	NDUFB3 PPI subnetwork	0.98
ENSG00000120251	GRIA2 PPI subnetwork	0.98
GO:0006119	oxidative phosphorylation	0.98
ENSG00000099246	RAB18 PPI subnetwork	0.98
GO:0061053	somite development	0.98
GO:0033275	actin-myosin filament sliding	0.98

Original gene set ID	Original gene set description	Nominal P value
GO:0030049	muscle filament sliding	0.98
MP:0000433	microcephaly	0.98
ENSG00000167258	CDK12 PPI subnetwork	0.98
GO:0016634	oxidoreductase activity, acting on the CH-CH group of donors, oxygen as acceptor	0.98
GO:0021952	central nervous system projection neuron axonogenesis	0.98
ENSG00000124164	VAPB PPI subnetwork	0.98
GO:0009109	coenzyme catabolic process	0.98
MP:0004087	abnormal muscle fiber morphology	0.98
ENSG00000182117	NOP10 PPI subnetwork	0.98
GO:0051187	cofactor catabolic process	0.98
REACTOME_RESOLUTION_OF_AP_SITES_VIA_THE_MULTIPLE:NUCLEOTIDE_PAT	REACTOME_RESOLUTION_OF_AP_SITES_VIA_THE_MULTIPLE:NUCLEOTIDE_PATCH_	0.98
REACTOME_REMOVAL_OF_DNA_PATCH_CONTAINING_ABASIC_RESIDUE	REACTOME_REMOVAL_OF_DNA_PATCH_CONTAINING_ABASIC_RESIDUE	0.98
GO:0090183	regulation of kidney development	0.98
ENSG00000003096	KLHL13 PPI subnetwork	0.98
ENSG00000170515	PA2G4 PPI subnetwork	0.98
ENSG00000125447	GGA3 PPI subnetwork	0.98
ENSG00000077721	UBE2A PPI subnetwork	0.98
MP:0002674	abnormal sperm motility	0.98
MP:0001706	abnormal left-right axis patterning	0.98
MP:0004132	absent embryonic cilia	0.98
GO:0001658	branching involved in ureteric bud morphogenesis	0.98
GO:0008009	chemokine activity	0.98
GO:0060134	prepulse inhibition	0.98
ENSG00000163535	SGOL2 PPI subnetwork	0.98
GO:0006369	termination of RNA polymerase II transcription	0.98
GO:0009898	internal side of plasma membrane	0.98
ENSG00000105968	H2AFV PPI subnetwork	0.98
GO:0007098	centrosome cycle	0.98
ENSG00000092531	SNAP23 PPI subnetwork	0.98
GO:0006488	dolichol-linked oligosaccharide biosynthetic process	0.99
GO:0016101	diterpenoid metabolic process	0.99
ENSG00000171132	PRKCE PPI subnetwork	0.99
GO:0006513	protein monoubiquitination	0.99
GO:0042312	regulation of vasodilation	0.99
ENSG00000160194	NDUFV3 PPI subnetwork	0.99
GO:0009068	aspartate family amino acid catabolic process	0.99
GO:0006904	vesicle docking involved in exocytosis	0.99
ENSG00000010256	UQCRC1 PPI subnetwork	0.99
GO:0044236	multicellular organismal metabolic process	0.99
GO:0008045	motor axon guidance	0.99
REACTOME_TRAFFICKING_OF_GLUR2:CONTAINING_AMPA_RECEPTORS	REACTOME_TRAFFICKING_OF_GLUR2:CONTAINING_AMPA_RECEPTORS	0.99
ENSG00000139197	PEX5 PPI subnetwork	0.99
GO:0001523	retinoid metabolic process	0.99
MP:0002954	abnormal aerobic energy metabolism	0.99
MP:0011386	increased metanephric mesenchyme apoptosis	0.99
GO:0072163	mesonephric epithelium development	0.99
GO:0072164	mesonephric tubule development	0.99
ENSG00000180104	EXOC3 PPI subnetwork	0.99
ENSG00000212872	ENSG00000212872 PPI subnetwork	0.99

Original gene set ID	Original gene set description	Nominal P value
ENSG00000212871	ENSG00000212871 PPI subnetwork	0.99
ENSG00000198868	ENSG00000198868 PPI subnetwork	0.99
ENSG00000198840	MT-ND3 PPI subnetwork	0.99
GO:0042693	muscle cell fate commitment	0.99
ENSG00000100883	SRP54 PPI subnetwork	0.99
ENSG00000136521	NDUFB5 PPI subnetwork	0.99
ENSG00000164329	PAPD4 PPI subnetwork	0.99
GO:0006776	vitamin A metabolic process	0.99
ENSG00000132604	TERF2 PPI subnetwork	0.99
ENSG00000115286	NDUFS7 PPI subnetwork	0.99
ENSG00000104313	EYA1 PPI subnetwork	0.99
ENSG00000139112	GABARAPL1 PPI subnetwork	0.99
GO:0008527	taste receptor activity	0.99
MP:0008415	abnormal neurite morphology	0.99
ENSG00000105829	BET1 PPI subnetwork	0.99
GO:0043130	ubiquitin binding	0.99
ENSG00000197969	VPS13A PPI subnetwork	0.99
GO:0015844	monoamine transport	0.99
ENSG00000168653	NDUFS5 PPI subnetwork	0.99
GO:0005665	DNA-directed RNA polymerase II, core complex	0.99
GO:0045687	positive regulation of glial cell differentiation	0.99
ENSG00000106636	YKT6 PPI subnetwork	0.99
REACTOME_SYNTHESIS_OF_BILE_ACIDS_AND_BILE_SALTS	REACTOME_SYNTHESIS_OF_BILE_ACIDS_AND_BILE_SALTS	0.99
MP:0005399	increased susceptibility to fungal infection	0.99
ENSG00000198886	MT-ND4 PPI subnetwork	0.99
ENSG00000212869	ENSG00000212869 PPI subnetwork	0.99
ENSG00000198695	MT-ND6 PPI subnetwork	0.99
ENSG00000203811	HIST2H3C PPI subnetwork	0.99
ENSG00000183598	HIST2H3D PPI subnetwork	0.99
ENSG00000099795	NDUFB7 PPI subnetwork	0.99
ENSG00000159352	PSMD4 PPI subnetwork	0.99
ENSG00000204218	ENSG00000204218 PPI subnetwork	0.99
ENSG00000109911	ELP4 PPI subnetwork	0.99
ENSG00000180871	CXCR2 PPI subnetwork	0.99
GO:0004527	exonuclease activity	0.99
GO:0016645	oxidoreductase activity, acting on the CH-NH group of donors	0.99
GO:0050919	negative chemotaxis	0.99
GO:0001662	behavioral fear response	0.99
ENSG00000147601	TERF1 PPI subnetwork	0.99
GO:0030890	positive regulation of B cell proliferation	0.99
GO:0034311	diol metabolic process	0.99
GO:0006584	catecholamine metabolic process	0.99
GO:0009712	catechol-containing compound metabolic process	0.99
ENSG00000128513	POT1 PPI subnetwork	0.99
ENSG00000162129	CLPB PPI subnetwork	0.99
GO:0019783	small conjugating protein-specific protease activity	0.99
GO:0032182	small conjugating protein binding	0.99
ENSG00000006712	PAF1 PPI subnetwork	0.99
ENSG00000134014	ELP3 PPI subnetwork	0.99

Original gene set ID	Original gene set description	Nominal P value
GO:0042537	benzene-containing compound metabolic process	0.99
ENSG00000197579	TOPORS PPI subnetwork	0.99
ENSG00000054116	TRAPPC3 PPI subnetwork	0.99
GO:0071824	protein-DNA complex subunit organization	0.99
ENSG00000143614	GATAD2B PPI subnetwork	0.99
GO:0070252	actin-mediated cell contraction	0.99
GO:0021955	central nervous system neuron axonogenesis	0.99
GO:0046513	ceramide biosynthetic process	0.99
GO:0004843	ubiquitin-specific protease activity	0.99
ENSG00000213496	ENSG00000213496 PPI subnetwork	0.99
ENSG00000197265	GTF2E2 PPI subnetwork	0.99
GO:0005747	mitochondrial respiratory chain complex I	0.99
GO:0045271	respiratory chain complex I	0.99
GO:0030964	NADH dehydrogenase complex	0.99
GO:0045652	regulation of megakaryocyte differentiation	0.99
GO:0007099	centriole replication	0.99
MP:0001899	absent long term depression	0.99
ENSG00000197548	ATG7 PPI subnetwork	0.99
MP:0000819	abnormal olfactory bulb morphology	0.99
GO:0060174	limb bud formation	0.99
GO:0031055	chromatin remodeling at centromere	0.99
ENSG00000125798	FOXA2 PPI subnetwork	0.99
ENSG00000188459	ENSG00000188459 PPI subnetwork	0.99
ENSG00000197930	ERO1L PPI subnetwork	0.99
GO:0015872	dopamine transport	0.99
GO:0032963	collagen metabolic process	0.99
ENSG00000119048	UBE2B PPI subnetwork	0.99
ENSG00000168393	DTYMK PPI subnetwork	0.99
ENSG00000215694	ENSG00000215694 PPI subnetwork	0.99
GO:0043486	histone exchange	0.99
GO:0034502	protein localization to chromosome	0.99
GO:0005230	extracellular ligand-gated ion channel activity	0.99
ENSG00000147853	AK3 PPI subnetwork	0.99
GO:0009081	branched chain family amino acid metabolic process	0.99
GO:0007618	mating	0.99
GO:0030534	adult behavior	0.99
GO:0008137	NADH dehydrogenase (ubiquinone) activity	0.99
GO:0050136	NADH dehydrogenase (quinone) activity	0.99
GO:0003954	NADH dehydrogenase activity	0.99
ENSG00000163032	VSNL1 PPI subnetwork	0.99
GO:0004953	icosanoid receptor activity	0.99
GO:0004954	prostanoid receptor activity	0.99
GO:0033572	transferrin transport	0.99
GO:0015682	ferric iron transport	0.99
ENSG00000130176	CNN1 PPI subnetwork	0.99
ENSG00000183648	NDUFB1 PPI subnetwork	0.99
ENSG00000127184	COX7C PPI subnetwork	0.99
GO:0001964	startle response	0.99
REACTOME_MITOCHONDRIAL_FATTY_ACID_BETA:OXIDATION	REACTOME_MITOCHONDRIAL_FATTY_ACID_BETA:OXIDATION	0.99

Original gene set ID	Original gene set description	Nominal P value
GO:0007494	midgut development	0.99
ENSG00000124702	KLHDC3 PPI subnetwork	0.99
ENSG00000178127	NDUFV2 PPI subnetwork	0.99
ENSG00000212870	ENSG00000212870 PPI subnetwork	0.99
ENSG00000198786	MT-ND5 PPI subnetwork	0.99
GO:0042596	fear response	0.99
MP:0002243	abnormal vomeronasal organ morphology	0.99
GO:0072529	pyrimidine-containing compound catabolic process	0.99
ENSG00000147123	NDUFB11 PPI subnetwork	0.99
MP:0000747	muscle weakness	0.99
GO:0071103	DNA conformation change	0.99
ENSG00000185513	L3MBTL1 PPI subnetwork	0.99
GO:0050684	regulation of mRNA processing	1
GO:0006739	NADP metabolic process	1
GO:0044259	multicellular organismal macromolecule metabolic process	1
ENSG00000138029	HADHB PPI subnetwork	1
GO:0006740	NADPH regeneration	1
ENSG00000108587	GOSR1 PPI subnetwork	1
ENSG00000109390	NDUFC1 PPI subnetwork	1
ENSG00000172301	C17orf79 PPI subnetwork	1
GO:0002209	behavioral defense response	1
KEGG_PROTEIN_EXPORT	KEGG_PROTEIN_EXPORT	1
GO:0046520	sphingoid biosynthetic process	1
GO:0016655	oxidoreductase activity, acting on NADH or NADPH, quinone or similar compound as	1
ENSG00000112357	PEX7 PPI subnetwork	1
ENSG00000111875	ASF1A PPI subnetwork	1
ENSG00000185214	ENSG00000185214 PPI subnetwork	1
GO:0009262	deoxyribonucleotide metabolic process	1
ENSG00000155511	GRIA1 PPI subnetwork	1
GO:0065004	protein-DNA complex assembly	1
GO:0051952	regulation of amine transport	1
GO:0003924	GTPase activity	1
ENSG00000151366	NDUFC2 PPI subnetwork	1
ENSG00000147416	ATP6V1B2 PPI subnetwork	1
GO:0004869	cysteine-type endopeptidase inhibitor activity	1
MP:0010454	abnormal truncus arteriosus septation	1
GO:0034728	nucleosome organization	1
ENSG00000166963	MAP1A PPI subnetwork	1
ENSG00000115738	ID2 PPI subnetwork	1
ENSG00000187837	HIST1H1C PPI subnetwork	1
GO:0034080	CenH3-containing nucleosome assembly at centromere	1
GO:0006336	DNA replication-independent nucleosome assembly	1
GO:0034724	DNA replication-independent nucleosome organization	1
ENSG00000185621	LMLN PPI subnetwork	1
GO:0003995	acyl-CoA dehydrogenase activity	1
MP:0008531	increased chemical nociceptive threshold	1
ENSG00000121390	PSPC1 PPI subnetwork	1
GO:0021871	forebrain regionalization	1
ENSG00000206440	NFKBIL1 PPI subnetwork	1

Original gene set ID	Original gene set description	Nominal P value
ENSG00000168593	ENSG00000168593 PPI subnetwork	1
MP:0005480	increased circulating triiodothyronine leve	1
GO:0006098	pentose-phosphate shunt	1
GO:0042401	cellular biogenic amine biosynthetic process	1
GO:0000178	exosome (RNase complex)	1
ENSG00000198888	MT-ND1 PPI subnetwork	1
GO:0042136	neurotransmitter biosynthetic process	1
ENSG00000131747	TOP2A PPI subnetwork	1
ENSG00000131495	NDUFA2 PPI subnetwork	1
REACTOME_MRNA_DECAY_BY_3_TO_5_EXORIBONUCLEASE	REACTOME_MRNA_DECAY_BY_3_TO_5_EXORIBONUCLEASE	1
ENSG00000165264	NDUFB6 PPI subnetwork	1
MP:0002007	increased cellular sensitivity to gamma-irradiation	1
ENSG00000092330	TINF2 PPI subnetwork	1
ENSG00000212876	ENSG00000212876 PPI subnetwork	1
ENSG00000198763	MT-ND2 PPI subnetwork	1
GO:0006333	chromatin assembly or disassembly	1
GO:0031497	chromatin assembly	1
GO:0007628	adult walking behavior	1
GO:0016780	phosphotransferase activity, for other substituted phosphate groups	1
GO:0033540	fatty acid beta-oxidation using acyl-CoA oxidase	1
ENSG00000083896	YTHDC1 PPI subnetwork	1
GO:0032204	regulation of telomere maintenance	1
ENSG00000108671	PSMD11 PPI subnetwork	1
GO:0001504	neurotransmitter uptake	1
ENSG00000108468	CBX1 PPI subnetwork	1
ENSG00000079462	PAFAH1B3 PPI subnetwork	1
GO:0015992	proton transport	1
GO:0009378	four-way junction helicase activity	1
ENSG00000116288	PARK7 PPI subnetwork	1
REACTOME_RESPIRATORY_ELECTRON_TRANSPORT	REACTOME_RESPIRATORY_ELECTRON_TRANSPORT	1
GO:0021536	diencephalon development	1
REACTOME_RESPIRATORY_ELECTRON_TRANSPORT_ATP_SYNTHESIS_BY_CHEM	REACTOME_RESPIRATORY_ELECTRON_TRANSPORT_ATP_SYNTHESIS_BY_CHEMIOSM	1
ENSG00000013275	PSMC4 PPI subnetwork	1
GO:0006818	hydrogen transport	1
ENSG00000167774	NDUFA7 PPI subnetwork	1
GO:0006282	regulation of DNA repair	1
GO:0006323	DNA packaging	1
GO:0050922	negative regulation of chemotaxis	1
GO:0015991	ATP hydrolysis coupled proton transport	1
GO:0015988	energy coupled proton transport, against electrochemical gradient	1
GO:0015078	hydrogen ion transmembrane transporter activity	1
ENSG00000111880	RNGTT PPI subnetwork	1
KEGG_OXIDATIVE_PHOSPHORYLATION	KEGG_OXIDATIVE_PHOSPHORYLATION	1
GO:0008344	adult locomotory behavior	1
GO:0031290	retinal ganglion cell axon guidance	1
ENSG00000140990	NDUFB10 PPI subnetwork	1
ENSG00000153140	CETN3 PPI subnetwork	1
ENSG00000120696	KBTBD7 PPI subnetwork	1
GO:0006334	nucleosome assembly	1

Original gene set ID	Original gene set description	Nominal P value
GO:0006586	indolalkylamine metabolic process	1
GO:0042430	indole-containing compound metabolic process	1
GO:0018958	phenol-containing compound metabolic process	1
GO:0000381	regulation of alternative nuclear mRNA splicing, via spliceosome	1
ENSG00000108433	GOSR2 PPI subnetwork	1
GO:0001963	synaptic transmission, dopaminergic	1
REACTOME_E2F:ENABLED_INHIBITION_OF_PRE:REPLICATION_COMPLEX_FORM	REACTOME_E2F:ENABLED_INHIBITION_OF_PRE:REPLICATION_COMPLEX_FORMATI	1
GO:0006635	fatty acid beta-oxidation	1
ENSG00000203813	HIST1H3H PPI subnetwork	1
ENSG00000203852	HIST2H3A PPI subnetwork	1
ENSG00000131143	COX4I1 PPI subnetwork	1
ENSG00000166848	TERF2IP PPI subnetwork	1
MP:0000761	thin diaphragm muscle	1
GO:0021983	pituitary gland development	1
ENSG00000115677	HDLBP PPI subnetwork	1
ENSG00000113327	GABRG2 PPI subnetwork	1
GO:0006879	cellular iron ion homeostasis	1
GO:0021756	striatum development	1
GO:2001020	regulation of response to DNA damage stimulus	1
GO:0043044	ATP-dependent chromatin remodeling	1
GO:0006576	cellular biogenic amine metabolic process	1
GO:0009636	response to toxin	1
GO:0032200	telomere organization	1
GO:0009083	branched chain family amino acid catabolic process	1
REACTOME_BRANCHED:CHAIN_AMINO_ACID_CATABOLISM	REACTOME_BRANCHED:CHAIN_AMINO_ACID_CATABOLISM	1
REACTOME_PEROXISOMAL_LIPID_METABOLISM	REACTOME_PEROXISOMAL_LIPID_METABOLISM	1
ENSG00000179841	AKAP5 PPI subnetwork	1
GO:0000723	telomere maintenance	1
ENSG00000196532	HIST1H3C PPI subnetwork	1
ENSG00000198366	HIST1H3A PPI subnetwork	1
ENSG00000197153	HIST1H3J PPI subnetwork	1
ENSG00000197409	HIST1H3D PPI subnetwork	1
ENSG00000178458	ENSG00000178458 PPI subnetwork	1
ENSG00000112727	ENSG00000112727 PPI subnetwork	1
ENSG00000124693	HIST1H3B PPI subnetwork	1
ENSG00000196966	HIST1H3E PPI subnetwork	1
ENSG00000182572	HIST1H3I PPI subnetwork	1
ENSG00000180198	RCC1 PPI subnetwork	1
KEGG_ALZHEIMERS_DISEASE	KEGG_ALZHEIMERS_DISEASE	1
GO:0000380	alternative nuclear mRNA splicing, via spliceosome	1
GO:0017156	calcium ion-dependent exocytosis	1
