# Supplementary material for: Meta-Analysis of Genome-Wide Association Studies for Abdominal Aortic Aneurysm Identifies Four New Disease-Specific Risk Loci
Source: Circ Res. 2017 Jan 19;120(2):341–53. doi: 10.1161/CIRCRESAHA.116.308765 (PMC5253231; doi:10.1161/CIRCRESAHA.116.308765)
Supplement: Supplementary file 2 [file res-120-341-s002.pdf]

SUPPLEMENTAL MATERIAL

Jones et al.: Meta-analysis of genome-wide association studies for abdominal aortic aneurysm identifies four new disease specific risk loci

ONLINE METHODS AND DATA*

	Page
Discovery and validation cohort descriptions	2
Meta-analysis	9
SNP lookup in GWAS for traits associated with AAA	30
Search for other associated traits and diseases using GWAS databases	32
PheWAS analysis	37
Annotation of AAA associated SNPs using the UCSC Genome Browser	38
Pupasuite analysis	41
GWAS3D analysis	44
DEPICT analysis	49
Functional effects of SNPs at AAA loci	60
Validation of GWAS3D results using mRNA expression data	67
Look-up for transcription factor binding sites	70
Network analysis	72
Consortia contributing data	76
References	79

*for clarity and ease of use each section contains methods, results, figures and tables relevant to that section.

INDIVIDUAL GWAS STUDIES

All known studies with AAA genome-wide genotyping data were invited to join the International Aneurysm Consortium effort. All studies agreed to participate in the meta-GWAS, with cohort case control descriptions and inclusion/exclusion criteria having been previously reported[1-3] (**Online Table I**). All AAA cases shared a common definition of infra-renal aortic diameter ≥30 mm. Patients with connective tissue disease associated AAAs (e.g. Marfan, Ehlers-Danlos, Loeys-Dietz) were excluded from the study. Each GWAS was based on a case-control analysis of AAA modelled as a discrete trait. The statistical analysis of the Aneurysm Consortium and New Zealand GWAS datasets was repeated specifically for this study and therefore was harmonized using identical imputation and analysis methods. Data from the remaining cohorts consisted of summary data obtained from previously performed analyses.

The use of the samples in each study cohort was approved by the local Ethics Committees or Institutional Review Boards.

DISCOVERY AND VALIDATION COHORT DESCRIPTIONS (Online Tables I and II)

a) Discovery Cohorts

Aneurysm Consortium (AC) AAA GWAS dataset: The Aneurysm Consortium recruited cases of AAA from centres across the United Kingdom and Western Australia. Cases were defined as an infra-renal aortic diameter ≥ 30 mm proven on ultrasound or computerized tomography (CT) scan. Controls were taken from the WTCCC2 common control group[1, 4] and were therefore unscreened for AAA.

Data were from 1,866 cases with AAA and 5,435 unscreened controls from the Wellcome Trust Case Control Consortium 2 (WTCCC2) study consisting of samples from the 1958 British Birth Cohort and from the UK National Blood Service. DNA samples were processed at the Wellcome Trust Sanger Institute (WTSI). Genomic DNA was quantified by PicoGreen assay, and quality control (QC) assured by both agarose gel electrophoresis and Sequenom iPLEX genotyping of 29 SNPs and 4 sex-specific markers. Genotyping for the discovery study was performed using Illumina 1.2M (controls) or 670K (AAA) BeadChips. Raw intensity data were normalized using BeadStudio and genotypes were called concurrently from the combined case-control data set using the Illuminus algorithm[5].

As part of the original Aneurysm Consortium GWAS individual sample QC had been performed as follows. QC was first performed by exclusion of SNPs with call rates < 0.98 and those that demonstrated significant deviation from Hardy-Weinberg equilibrium in the control group ($P<5x10^{-4}$). Duplicate samples and those that failed genotyping (sample call rates <0.98) were also excluded from further analysis. Genotyping cluster plots for all SNPs with $P< 1x10^{-4}$ were visually inspected to exclude from further analysis positive associations generated by erroneous genotyping or calling. Checks for population stratification were performed by PLINK[6] identical by state clustering and extreme outliers were removed from the analysis.

Imputation was performed using IMPUTE 2.2 run on the BCISNPmax database platform (version 3.5, BCI Platforms, Espoo, Finland). The reference haplotypes were based on the 1000 Genomes June 2011 release. Imputed calls were filtered by quality score (excluding those <0.9) to restrict to higher quality imputed SNPs.

Following imputation further QC filtering was performed, excluding SNPs with call rates < 0.98 and those that demonstrated significant deviation from Hardy-Weinberg equilibrium in the control group ($P<5x10^{-4}$). Duplicate samples and those that failed genotyping (sample call rate <0.98) were also excluded from further analysis. Association testing was carried out in PLINK[6].

New Zealand (NZ) Vascular Genetics Study AAA GWAS dataset: The Vascular Research Consortium of New Zealand recruited New Zealand men and women with a proven history of AAA (infra-renal aortic diameter ≥ 30 mm proven on ultrasound or CT scan). Approximately 80% had undergone surgical AAA repair (typically AAA's > 50-55 mm in diameter). The vast majority of cases (>97%) were of Anglo-European ancestry. The control group underwent an abdominal ultrasound scan to exclude (>25 mm) concurrent AAA and Anglo-European ancestry was required for inclusion. Controls were also screened for peripheral artery disease (PAD; using ankle brachial index), carotid artery disease (ultrasound) and other cardiovascular risk factors.

Two separate GWAS were performed using New Zealand samples. NZ GWAS 1 consisted of 608 AAA patients (474 male) and 612 elderly controls (450 male), genotyped using the Affymetrix SNP 6 GeneChip array. All samples had call rates >0.95 (mean 0.992). NZ GWAS 2 consisted of 397 AAA patients (332 male) and 384 elderly controls (308 male), genotyped using the Illumina Infinium Omni2.5 BeadChip array. All samples had call rates >0.95 (mean 0.990). All NZ genomic DNA samples exceeded manufacturer's quality and quantity requirements having undergone pre-assessment by Nanophotometer (Implen GmbH, München, Germany) and agarose gel electrophoresis.

Imputation was conducted separately on NZ GWAS 1 and 2 data sets using the same methods as used for the Aneurysm Consortium datasets. IMPUTE 2.2 was run on the BCISNPmax database platform (version 3.5, BCI Platforms, Espoo, Finland). The reference haplotypes were based on the 1000 Genomes June 2011 release. Imputed calls were filtered by quality score (excluding those <0.9) to restrict to higher quality imputed SNPs. The genomic inflation factors (λ) were 1.07 and 1.05, respectively (MAF >0.05).

Both NZ GWAS 1 and 2 data sets underwent QC filtering, excluding SNPs with call rates < 0.98 and those that demonstrated significant deviation from Hardy-Weinberg equilibrium in the control group ($P<5x10^{-4}$). Duplicate samples and those that failed genotyping (sample call rate <0.98) were also excluded from further analysis. Association testing was carried out in PLINK[6].

US (PA) GWAS dataset: AAA patients were enrolled through the Department of Vascular Surgery at Geisinger Medical Center, Danville, Pennsylvania, USA as previously reported[2, 7]. To identify cases and controls from the electronic medical records, an ePhenotyping algorithm was developed[8]. Briefly, Structured Query Language (SQL) was used to script the algorithm utilizing "Current Procedural Terminology" (CPT) and "International Classification of Diseases" (ICD-9) codes as well as demographic and encounter data to classify individuals as case, control, or excluded. AAA cases were defined as having an AAA repair procedure (case Type 1), or at least one appropriate specialty encounter (vascular clinic) with a ruptured AAA (case Type 2), or at least two specialty encounters with an unruptured AAA (case Type 3). Controls were neither cases nor those excluded, had an encounter within the past 5 years, and had never been assigned an ICD9 code of 441.*, where * is a 1 or 2 digit code. Individuals were excluded if 1) they had a thoracic aortic aneurysm or a rare heritable disease with aortic manifestation; 2) they were younger than 40 or older than 89 years, 3) they had a single encounter with a code without mention of rupture (441.4), or 4) they had not had an encounter within the past 5 years. Rare heritable diseases were excluded because the goal of the current study was to identify non-syndromic AAA. Controls under 40 years might yet manifest an AAA, while cases under 40 years of age and without rare syndromic forms of aortic aneurysms are likely due to trauma. The AAA algorithm can be downloaded from www.PheKb.org. The algorithm was validated on a subset of individuals by manual chart review, and implemented at eMERGE network sites. The algorithm was implemented as a workflow in the Konstanz Information Miner (KNIME) (http://www.knime.org/).

AAA cases had infrarenal aortic diameter ≥ 30 mm as revealed by abdominal imaging. Approximately 20% of individuals with AAA had a family history of AAA. A control group was obtained through the Geisinger MyCode® Project, a cohort of Geisinger Clinic patients recruited for genomic studies. The MyCode® controls were matched for age distribution and sex to the Geisinger Vascular Clinic AAA cases. Based on electronic medical records, controls had no ICD-9 codes for AAA in their records, but they were not screened by ultrasonography for AAA. Both cases and controls from the Geisinger Clinic were of European descent.

The Geisinger cohort used for this study was a subset of a larger cohort comprising 3,264 samples from 3,149 individuals with three phenotypes: 922 putative AAA cases, 981 obesity cases and 1,246 controls. Samples were genotyped on the Illumina HumanOmniExpress-12v1.0 genotyping platform at the University of Pittsburgh Genomics and Proteomics Core Laboratories. Genotypes were called using the Illumina GenomeStudio v2010.3 software. QC consisted of a number of steps: identification of cross-contamination and removal of specimens, call rate of samples (> 0.98 SNPs called), sex consistency between annotated sex and genotyped sex, SNP discordance between replicate sample pairs, SNP call rate (> 0.95 calls in all specimens), SNP minor allele frequency (> 0.01), SNP Hardy-Weinberg equilibrium ($P > 1x\ 10^{-4}$), and selection of replicates to retain based on sex-specific Mahalanobis distance (< 4.1) and Illumina P10.GC (> 0.71). Cross-contamination of samples was detected by excess heterozygosity and excess relatedness (related to more than half of other samples at Pi-hat > 0.0625); four samples were removed prior to other QC steps. After the QC steps above, related individuals (pairwise Pi-hat > 0.15) were removed, retaining the individual and specimen with the highest call rate. A second round of QC was applied using the above SNP and sample criteria to ensure consistency after removal of SNPs and individuals. In addition, the SNP criteria were analyzed per chromosome to ensure that there were no systematic differences (no differences detected). Lastly, principle component analysis (PCA) was used to determine if there were any batch effects during genotyping (no evidence for batch effects). Of the 3,264 samples, 153 were removed for one or more of the QC reasons above. Of the 731,306 SNPs, 95,369 were removed; 2,012 were discordant, 13,107 had a low call rate, 78,086 had a MAF < 0.01, and 14,056 had a HWE $P < 1x\ 10^{-4}$ (9,047 SNPs were removed for more than one reason).

The final meta-analysis cohort comprised only those individuals who were identified as definitive AAA cases or controls using the rigorous ePhenotyping algorithm described above.

Imputation was performed as previously described[9]. Briefly: SNPs were re-mapped to the Genome Reference Consortium Human build 37 (GRCh37) and the program liftOver run to ensure mapping consistency. Subsequently all SNPs were mapped from the Illumina TOP notation to the plus (+) strand. Strand was checked using SHAPEIT2 (version r2.644)[10]. Next the data were phased using SHAPEIT2. Imputation was performed using IMPUTE2 (version 2.3.0)[11]. Chromosomes were divided into 6 MB segments with 250 kbp overlap between segments. A total of 5,719,283 SNPs with an info score of ≥ 0.9 were used for analysis.

Association analysis without adjustment was performed using PLINK (v1.09)[6] and the imputed SNPs.

The eMERGE Network Imputed GWAS for 41 Phenotypes (the dbGaP eMERGE Phase 1 and 2 Merged data Submission) accession number is: phs000888.v1.p1 which includes the Geisinger AAA data.

Iceland, deCODE Genetics AAA GWAS dataset: Icelandic individuals with AAA (defined as infra-renal aortic diameter ≥ 30 mm) were recruited from a registry of individuals who were admitted at Landspitali University Hospital, in Reykjavik, Iceland, 1980 – 2006. AAA patients were either followed up or treated by intervention for emergency repair of symptomatic or ruptured AAA or for an elective repair by surgery or endovascular intervention. In total, whole genome data from 557 subjects with AAA, enrolled as part of the cardiovascular disease (CVD) genetics program at deCODE,

were included in the metaGWAS. The Icelandic controls used (n=89,235) were selected from individuals who have participated in various GWA studies and who were recruited as part of genetic programs at deCODE. Individuals with known CVD were excluded as controls[2] but controls were unscreened for AAA.

The Icelandic case and control samples were assayed with the Illumina HumanHap300, HumanHapCNV370 or HumanHap610 bead chips (Illumina, SanDiego, CA, USA). Only SNPs present on all chips were included in the analysis and SNPs were excluded if they had (a) call rates < 95% in cases or controls, (b) MAF<0.01 in the controls, or (c) showed significant deviation from HWE in the controls ($P < 1x10^{-4}$). These criteria were applied separately to genotype data from each of the chip types used and SNPs that showed significant deviation (P < 0.0001 in an ANOVA test) in frequency between the chips were excluded from the analysis. Any samples with a call rate < 0.98 were excluded from the analysis. The final analysis included 293,677 SNPs present on all three chips.

For case-control association analysis, we used a standard likelihood ratio statistic, implemented in the NEMO software[12], to calculate two-sided P values and ORs for each individual allele, assuming a multiplicative model for risk[13].

Familial imputation: For the Icelandic data set, we extended the classical case-control association analysis to include *in silico* genotypes of affected individuals who were not genotyped but who had genotyped relatives[14] among the 40,000 Icelanders (about 13% of all living Icelanders) genotyped with the Illumina SNP chips at deCODE Genetics. For every ungenotyped affected individual, we calculated the probability distribution of the genotypes of his or her relatives, given his or her four possible phased genotypes. In practice, we included only genotypes of the affected individual's parents, children, siblings, half-siblings (and the half-sibling's parents), grandparents, grandchildren (and the grandchildren's parents) and spouses. The contribution of the ungenotyped affected individuals through this familial imputation to the effective sample size of the affected individuals, na,eff, was estimated using the Fisher information.

Genomic control: Some of the individuals in the Icelandic case-control groups are related to each other, causing the χ2 test statistic to have a mean >1 and median >0.455. We estimated the genome-wide inflation factor λg as the average of the 293,677 χ2 statistics to adjust for both relatedness and potential population stratification[14].

The Netherlands AAA GWAS dataset: The AAA sample set from Utrecht was recruited in 2007-2009 from 8 centres in The Netherlands[2], mainly when individuals visited their vascular surgeon in the clinic or, in rare cases, during hospital admission for elective or emergency AAA surgery. An AAA was defined as an infrarenal aorta ≥ 30 mm. The sample set comprised 89.9% males, with a mean AAA diameter of 58.4 mm, 61.7% had been operated on, of which 8.1 % were after rupture. The Dutch controls used in the AAA GWAS were recruited as part of the Nijmegen Biomedical Study and the Nijmegen Bladder Cancer Study (see http://dceg.cancer.gov/icbc/membership.html).

Genotyping was performed on Illumina HumanHap610 chips.[2] As controls, we included 2,791 Dutch subjects who were recruited as part of the Nijmegen Biomedical Study (n=1,832) and the Nijmegen Bladder Cancer Study (n=1,278)[15, 16] These controls were genotyped on Illumina CNV370 Duo BeadChips.

QC: We performed QC using PLINK version 1.07[6]. After removal of SNPs with A/T or C/G alleles and SNPs that were not called in any individual, we performed sample QC and SNP QC.

Sample QC was performed after merging cases and controls, using a subset of common, high-quality SNPs (as defined by SNPs without deviation from HWE (P>0.001), with high MA) (>0.2) and with low rate of missing genotypes (<0.01)). Linkage disequilibrium (LD) pruning (r^2>0.5) was performed. Subjects were removed based on the following three criteria: missing genotypes (subjects with call

rates < 0.95 were removed), heterozygosity (subjects were excluded if the inbreeding coefficient deviated more than 3 standard deviations from the mean) and cryptic relatedness (by calculating identity-by-descent (IBD) for each pair of individuals). In each pair with an IBD proportion of >20%, a subject was excluded, if it exhibited distant relatedness with more than one individual. For case-control pairs, we removed the control subject. In the case-case or control-control pairs, the subject with the lowest call rate was excluded.

Using these common, high-quality SNPs, we performed PCA using EIGENSTRAT on the remaining study subjects and HapMap-CEU subjects. We excluded SNPs from three regions with known long-distance LD: the major histocompatibility (MHC) region (chr6: 25.8-36 Mbp), the chromosome 8 inversion (chr8: 6-16 Mbp) and a chromosome 17 region (chr17: 40-45 Mbp). We created PC plots with the first four PCs, using R version 2.11.[17] Based on visual inspection of these plots, we excluded subjects that appeared to be outliers with respect to the CEU or the study population. After outlier removal, we recomputed PCs for them to be included as covariates in the logistic regression models.

After sample QC, we excluded SNPs with more than 2% missing genotypes, MAF < 0.01, missing genotype rate higher than MAF, and HWE deviation (P<0.001). Because cases and controls had been genotyped separately, we performed these QC steps in each study cohort separately and again after merging cases and controls. We also removed SNPs with a differential degree of missing genotypes between cases and controls ($P<1x10^{-5}$; chi-squared test).

Imputation: We performed genotype imputation using the pre-phasing/imputation stepwise approach implemented in IMPUTE2 and SHAPEIT (chunk size of 3 Mb and default parameters)[10, 18]. The imputation reference set consisted of 2,184 phased haplotypes from the full 1000 Genomes Project data set (February 2012; 40,318,253 variants). All genomic locations are given in NCBI Build 37/UCSC hg19 coordinates. After imputation, SNPs with an imputation accuracy score < 0.6 or MAF < 0.005 were excluded.

Association testing: Association testing was carried out in PLINK[6] using imputed SNP dosages. We included as covariates the first four PCs. We calculated genomic inflation factors (λ_{GC}), defined as the ratio of the median of the empirically observed distribution of the test statistic to the expected median[19].

b) Validation Cohorts

Aneurysm Consortium (AC) validation cohort: The same inclusion/exclusion criteria and recruitment sites were used as for the Aneurysm Consortium AAA GWAS. The lead SNPs (or their high LD proxies), identified in the discovery analysis, were genotyped at The Wellcome Trust Sanger Insitute, Cambridge, UK using Sequenom iPLEX platform. Allele frequency summary results (odds ratio and 95% confidence interval) were generated using Chi-squared tests as implemented in the SHEsis web-based software package[20] (available: http://analysis.bio-x.cn/SHEsisMain.htm). Deviation from HWE was estimated and results are shown in **Online Table VI.**

New Zealand (NZ) Validation cohort: NZ validation cohort participants were recruited from the same sites as those in the GWAS. Case and control inclusion/exclusion criteria were identical to that of the NZ AAA GWAS, with all controls having been screened by ultrasound.

The lead SNPs (or their high LD proxies), identified in the discovery analysis, were genotyped at the Vascular Research Group, University of Otago using the TaqMan (LifeTechnologies) platform. Allele frequency summary results (odds ratio and 95% confidence interval) were generated using Chi-squared tests as implemented in the SHEsis web-based software package[20]. Deviation from HWE was estimated and results are shown in **Online Table VI.**

Belgium and Canada validation cohorts: These sample-sets, in which all individuals were of European descent, included individuals with AAA who were admitted either for emergency repair of ruptured AAA or for an elective surgery to the University Hospital of Liege (Liege, Belgium) and to Dalhousie University Hospital (Halifax, Canada). AAA was defined as an infrarenal aortic diameter ≥ 30 mm. Details of these case-control sets have been reported previously[21, 22]. Approximately 40% of individuals with AAA had a family history of AAA. Control samples (51% males) were obtained from spouses of individuals with AAA or from individuals admitted to the same hospitals for reasons other than AAA. Controls had no known AAA, but they were not screened by ultrasonography for AAA.

The lead SNPs (or their high LD proxies), identified in the discovery analysis, were genotyped in the Tromp-Kuivaniemi Laboratory at Geisinger Health System using TaqMan (LifeTechnologies) platform. Allele frequency summary results (odds ratio and 95% confidence interval) were generated using Chi-squared tests as implemented in the SHEsis web-based software package[20]. Deviation from HWE was estimated and results are shown in **Online Table VI.**

eMERGE phase II (US) validation cohort: This cohort consisted of 338 AAA cases and 1,696 controls with GWAS data[9] available from Mayo Clinic, Marshfield Clinic, Mount Sinai School of Medicine , Vanderbilt University, Northwestern University and Group Health Research Institute. The cases and controls were ascertained from the electronic medical records[23] using an ePhenotyping algorithm[8] as described above. The samples had been genotyped in various GWAS and then imputed (see above). Allele frequency summary results (odds ratio and 95% confidence interval) were generated using Chi-squared tests as implemented in the SHEsis web-based software package[20]. Deviation from HWE was estimated and results are shown in **Online Table VI.** The eMERGE Network Imputed GWAS for 41 Phenotypes (the dbGaP eMERGE Phase 1 and 2 Merged data Submission) accession number is: phs000888.v1.p1 which includes these data.

US Validation 2 cohort: A second US case/control validation cohort was derived from the Mayo Vascular Disease Biorepository[24] (Mayo VDB; http://www.mayo.edu/research/labs/cardiovascular-biomarkers/vascular-diseases-biorepository), the Presbyterian University Hospital in Pittsburgh[25], Vanderbilt University (BioVU®)[26], Marshfield Clinic (Personalized Medicine Research Project®)[27], Mount Sinai School of Medicine (BioMe®)[28], and Northwestern University (NUgene®)[29]. The cases and controls at Mayo Clinic, Vanderbilt University, Marshfield Clinic, Mount Sinai School of Medicine, and Northwestern University were phenotypically ascertained using the same ePhenotyping algorithm[8] described above, whereas the AAA cases from the Presbyterian University Hospital in Pittsburgh were patients who had undergone elective or emergency surgery for AAA[25].

The lead SNPs (or their high LD proxies), identified in the discovery analysis, were genotyped in the Tromp-Kuivaniemi Laboratory at Geisinger Health System using TaqMan (LifeTechnologies) platform. Allele frequency summary results (odds ratio and 95% confidence interval) were generated using Chi-squared tests as implemented in the SHEsis web-based software package[20]. Deviation from HWE was estimated and results are shown in **Online Table VI.**

Italy validation cohort: This group consisted of 761 AAA cases and 520 controls. AAA cases were individuals referred to the Vascular Surgery Unit of the University of Florence. Familial and inflammatory AAAs were excluded from the study. All control subjects (n=520) had a negative personal and family history of AAA and were of comparable age and sex distribution to that of the AAA patients. A more detailed description of the study populations has been previously published[30].

The lead SNPs (or their high LD proxies), identified in the discovery analysis, were genotyped in the Giusti Laboratory at University of Florence using TaqMan (LifeTechnologies) platform. Allele frequency summary results (odds ratio and 95% confidence interval) were generated using Chi-squared tests as implemented in the SHEsis web-based software package[20]. Deviation from HWE was estimated and results are shown in **Online Table VI.**

Poland validation cohort: This group consisted of 481 AAA cases scheduled for surgery at the Department of General and Vascular Surgery of the Poznan University of Medical Sciences in the years 1999–2011. The control group, consisting of 487 subjects matched for age (±5 years) and sex to the AAA patients, was selected during the same time from the Poznan district[31]. The collection of samples was approved by the Bioethics Committee of the Poznan University of Medical Sciences. The diagnosis of AAA was evaluated by computed tomography angiography or magnetic resonance angiography. Based on physical examination supplemented with ultrasound duplex color scanning, the coexistence of PAD was recognized in 60.3% of the AAA patients. All patients were treated pharmacologically with statins, antiplatelet drugs and other drugs (antihypertensive or antidiabetic), depending on their clinical condition. The exclusion criteria for the controls included known aneurysms and PAD.

The lead SNPs (or their high LD proxies), identified in the discovery analysis, were genotyped in the Tromp-Kuivaniemi Laboratory at Geisinger Health System using TaqMan (LifeTechnologies) platform. Allele frequency summary results (odds ratio and 95% confidence interval) were generated using Chi-squared tests as implemented in the SHEsis web-based software package[20]. Deviation from HWE was estimated and results are shown in **Online Table VI.**

META-ANALYSIS of GWAS datasets

The discovery analysis consisted of the six cohorts with GWAS data detailed above, comprising 4,972 AAA cases and 99,858 controls, that were genotyped with a variety of genome-wide SNP arrays (**Online Table I**). All cohorts underwent QC filtering using the manufacturers' array-specific guidelines but with consistently applied inclusion criteria of SNP or sample call rates >95% and HWE $P>5x10^{-5}$ in controls[1-3, 7]. Each cohort then underwent imputation ([9]see above). Following imputation SNPs were quality controlled by quality score (Q>0.9) and MAF>0.05 in controls filtering, resulting in a common set of 5,363,770 SNPs across all discovery phase participants.

To obtain data for combination in the meta-analysis each case-control cohort was first analysed individually. Logistic regression models were used with AAA as a binary outcome in each cohort. Summary data [sample size, P-value, effect size (or log odds ratio), and the effect allele], unadjusted for covariates, for each SNP were combined in the meta-analysis.

The metaGWAS analysis was conducted using the METAL software package[32] on the BCISNPmax database platform (version 3.5, BCI Platforms, Espoo, Finland). METAL was implemented using the sample size scheme with weighting based on the effective sample size [$N_{eff}=4/(1/N_{cases} +1/N_{controls})$]. This approach was preferred over an inverse-variance weighted meta-analysis due to the disproportionate number of controls in some of the contributing cohorts and the fact that effect standard errors were not available in the data provided from Iceland and the United States (Geisinger) (**Online Table I**). The GWAS datasets from Iceland and the Netherlands were adjusted for genomic inflation prior to inclusion in the meta-analysis. The overall meta-GWAS analysis was adjusted for genomic inflation (λ) in each cohort (**Online Table I; Online Figure I**). An initial (λ-adjusted) discovery threshold of $P<5x10^{-6}$ was used to identify SNPs for subsequent validation genotyping.

The lead SNPs (or their high LD proxies), identified in the discovery analysis, were then genotyped in a further 8 independent cohorts (**Online Table II**). Each cohort's allele frequency summary results (odds ratio and 95% confidence interval) were generated using Chi-squared tests as implemented in the SHEsis web-based software package. Combined (discovery+validation) fixed effect meta-analysis was performed using a Maentel–Haenzel method with the genome-wide *P*-value significance threshold being set at $5x10^{-8}$. The Maentel-Haenzel method was chosen since SNPs from the discovery and validation studies that were being combined demonstrated effects in the same direction and with low/medium heterogeneity. A sensitivity analyses of the combined (discovery+validation) study data were also performed using a random-effects model[33]. The results from the discovery phase are presented in **Table 1; Online Tables III and IV; Figures 1 and 2**; and **Online Figures I and II.** The validation results are presented in **Table 1** and **Online Tables V and VI.** Results from the combined analyses are presented in **Table 1** and **Online Table VII**. Results from the sensitivity analysis are shown in **Online Table VIII.**

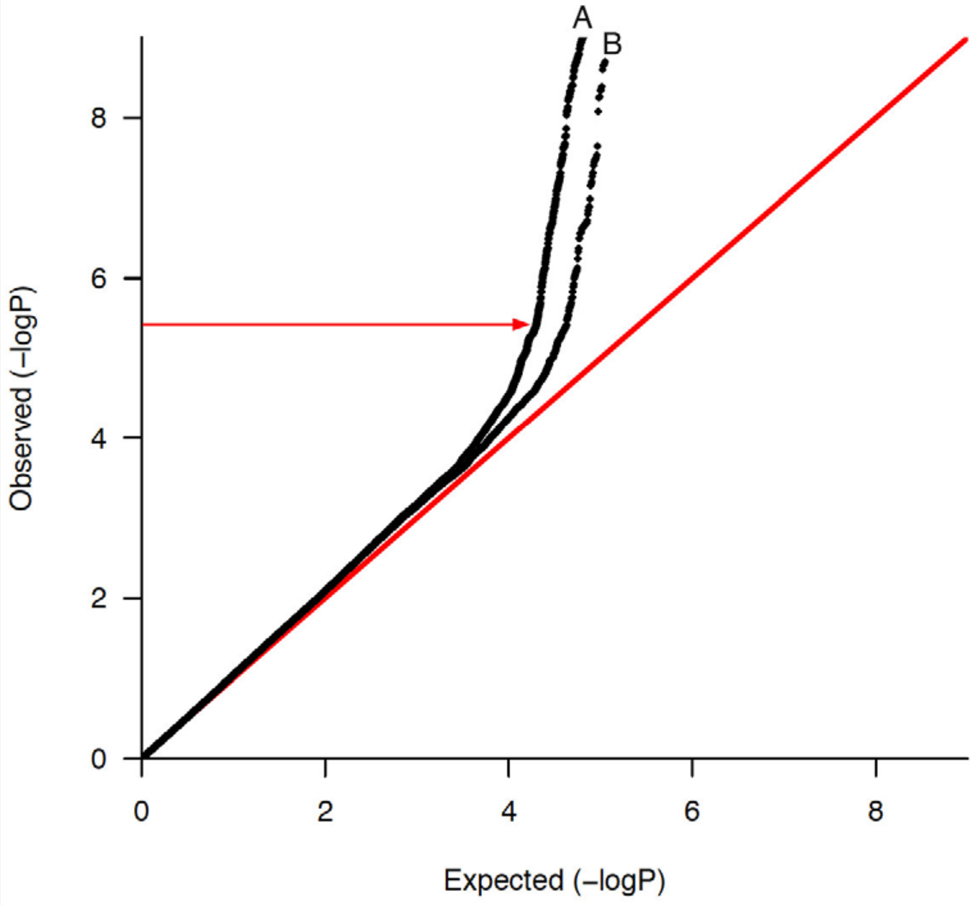

Online Figure I. Q-Q plot for the AAA meta-GWAS, showing (A) all 5.3 M SNPs with MAF>0.05 and (B) excluding the six previously identified loci (all SNPs within 100 kb of the peak variant associated with *SORT1, IL6R, CDKN2BAS1, DAB2IP, LDLR, LRP1*), generated from a comparison of 4,972 cases and 99,858 controls from 6 separate GWAS. The red arrow indicates the (λ-adjusted) $P<5x10^{-6}$ discovery threshold (362 SNPs in plot A).

Online Table I. Genome-wide association study (GWAS) cohort details. Individual level data were not available to calculate overall median age.

GWAS cohorts	Cases			Controls			GWAS Case weight (%)	Total* Case weight (%)	$N_{effective}$	Genotyping platform	Genomic inflation (λ) factor	Prior Adjustments
	Nn	%Male	Median Age (years)	N	% Male	Median Age (years)						
NZ 1	608	78	75	612	74	69	12.2	6.0	1,220	Affymetrix SNP6	1.07	None
NZ 2	397	84	77	384	80	67	8.0	3.9	781	Illumina Omni2.5	1.05	None
Aneurysm Consortium	1,846	98	72	5,605	49	52	37.1	18.1	5,555	Illumina 670	1.15	None
Netherlands	840	90	68	2,791	60	51	16.9	8.2	2,583	Illumina 300/370/610	1.11	Lambda
US (PA)	724	99	77	1,231	68	68	14.6	7.1	1,824	Illumina OmniExpress	1.06	None
Iceland deCODE	557	77	72	89,235	44	60	11.2	5.5	2,214	Illumina 300/370/610	0.70	Lambda
**Total**	**4,972**		**N/A**	**99,858**		**N/A**		**48.7%**	**14,176**			

Online Table II. Independent validation cohort details

Validation cohorts	Cases			Controls			Validation Case weight (%)	Total* Case weight (%)	$N_{effective}$	Genotyping platform
	N	% Male	Median Age (years)	N	% Male	Median Age (years)				
Aneurysm Consortium validation (AC)	1,236	84	72	2,196	93	68	23.6	12.1	3,163	Sequenom
NZ validation	753	81	77	1,237	67	68	14.4	7.4	1,872	Taqman
Italy validation	761	79	73	520	78	72	14.5	7.5	1,236	Taqman
Poland validation	481	86	69	487	72	59	9.2	4.7	968	Taqman
eMERGE US validation 1	338	80	82	1696	80	82	6.5	3.3	1127	Imputed data from various GWAS platforms
US validation 2	1,176	82	73	1,371	64	68	22.5	11.5	2,532	Taqman
Belgium validation	339	91	**N/A**	265	68	**N/A**	6.5	3.3	595	Taqman
Canada validation	148	75	**N/A**	136	79	**N/A**	2.8	1.5	283	Taqman
**Total**	**5,232**		**N/A**	**7,908**		**N/A**		**51.3%**		

*Combined (GWAS + Validation) analysis consisted of 10,204 AAA cases and 107,766 controls.

Online Table III: Summary of results for the lead SNPs at 19 putative AAA associated loci ($P<5x10^{-6}$) in the meta-analysis of 6 primary AAA GWAS datasets with a total of 4,972 AAA cases and 99,858 controls (see **Online Table I** for details on these cohorts). The results were based on an $N_{effective}$ weighted METAL analysis. The order of cohorts in the direction column is the same as that in **Online Table I** [NZ 1, NZ 2, Aneurysm Consortium, Netherlands, US (PA), Iceland (deCODE)]. See **Online Table IV** for MAF values for cases and controls separately.

							**$N_{effective}$ weighted analysis**		
**Chr**	**SNP**	**Position**	**Gene**	**Risk allele**	**Other allele**	**Direction**	**P**	**Phet**	**I^2**
1	rs602633	109821511	Near *PSRC1 CELSR2 SORT1*	T	G	------	$1.72x10^{-07}$	0.097	46.3
1	rs12133641	154428283	*IL6R*	A	G	++++++	$1.67x10^{-10}$	0.903	0.0
1	*rs4129267 proxySNP*	*154426264*	*IL6R*	*T*	*C*	------	$9.26x10^{-10}$	0.886	0.0
1	rs1795061	214409280	near *SMYD2*	T	C	++++++	$1.80x10^{-07}$	0.069	51.2
2	rs13382862	20882449	near *C2orf43* and *GDF7*	A	G	------	$3.03x10^{-08}$	0.878	0.0
4	rs10029392	5616048	*EVC2*	T	G	++++++	$4.60x10^{-06}$	0.147	38.8
5	rs12659791	74757758	*COL4A3BP*	T	C	-----+	$2.28x10^{-06}$	0.105	45.1
6	rs3176334	36648364	*CDKN1A*	C	T	------	$1.45x10^{-06}$	0.627	0.0
6	*rs733590 proxySNP*	*36645203*	*CDKN1A*	*T*	*C*	------	$8.74x10^{-06}$	0.584	0.0
8	rs3110425	107649626	*OXR1*	T	C	------	$3.25x10^{-06}$	0.895	0.0
9	rs10757274	22096055	*CDKN2BAS1/ANRIL*	A	G	------	$2.32x10^{-13}$	0.520	0.0
9	rs10985349	124425243	*DAB2IP*	T	C	++++++	$8.98x10^{-07}$	0.181	34.0
12	rs1385526	57532749	*LRP1*	C	G	------	$1.31x10^{-09}$	0.597	0.0
13	rs9316871	22861921	*LINC00540*	A	G	++++++	$5.95x10^{-06}$	0.143	39.4
15	rs17189674	89040591	*DET1*	A	G	++++++	$1.05x10^{-06}$	0.663	0.0
19	rs6511720	11202306	*LDLR*	T	G	------	$5.71x10^{-12}$	0.679	0.0
19	rs12980543	56096197	*ZNF579*	A	G	++++++	$2.30x10^{-06}$	0.301	17.4
19	*rs11084402 proxySNP*	*56093365*	*ZNF579*	*T*	*C*	++++++	$4.33x10^{-06}$	0.218	29.0
20	rs6516091	6050622	near *FERMT1*	A	G	+++++-	$3.82x10^{-09}$	0.027	60.5
20	rs58749629	44571317	near *PCIF1 ZNF335 MMP9*	A	G	++++++	$7.97x10^{-10}$	0.473	0.0
20	*rs3827066 proxySNP*	*44586023*	*near PCIF1 ZNF335 MMP9*	*T*	*C*	++++++	$9.18x10^{-10}$	0.729	0.0
21	rs2836411	39819830	*ERG*	T	C	+++++-	$1.53x10^{-07}$	0.103	45.5
X	rs5954362	140673423	*SPANXA1*	G	C	---	$2.73x10^{-07}$	0.271	23.2

Online Table IV: Individual cohort data from the 6 primary AAA GWAS studies, combined using the Maentel–Haenzel fixed effect method, for the lead SNPs at the 19 putative AAA loci identified in the meta-analysis of GWAS. See **Online Table I** for details on these cohorts. This table spans this and the following 6 pages.

CHR	SNP	POSITION	Region	Cohort	OR (95% CI)	P	Case/ Control	MAF$_{AAA}$	MAF$_{Control}$	
1	rs602633	109821511	Near P*SRC1 CELSR2 SORT1*	NZ GWAS 1	0.653 (0.535 - 0.797)	2.59E-05	608/ 612	0.1743	0.2442	
				NZ GWAS 2	0.874 (0.680 - 1.125)	0.2952	397/ 384	0.1870	0.2083	
				Aneurysm Consortium GWAS	0.864 (0.785 - 0.950)	0.002639	1846/ 5605	0.1942	0.2181	
				Netherlands GWAS	0.899 (0.778 - 1.038)	0.1458	840/ 2791	0.2151	0.2336	
				US (PA) GWAS	0.793 (0.680 - 0.925)	0.003166	724/ 1231	0.1843	0.2217	
				Iceland deCODE GWAS	0.916 (0.783 - 1.073)	0.2768	557/ 89235	0.1944	0.2085	
					**OR (95% CI)**	**Z-score**	**P-value**	**df (Q)**	**HetI^2**	**HetPval**
				**Combined**	0.845 (0.796 - 0.897)	-5.534	3.1E-08	5	29.4	0.215
1	rs12133641	154428283	*IL6R*	NZ GWAS 1	0.841 (0.715 - 0.989)	0.03596	608/ 612	0.3881	0.43	
	rs4129267 proxy	154426264		NZ GWAS 2	0.838 (0.683 - 1.027)	0.08904	397/ 384	0.3687	0.4108	
				Aneurysm Consortium GWAS	0.878 (0.813 - 0.948)	8.50E-04	1846/ 5605	0.3762	0.4072	
				Netherlands GWAS	0.818 (0.723 - 0.924)	0.001291	840/ 2791	0.3475	0.3936	
				US (PA) GWAS	0.817 (0.720 - 0.927)	0.001744	724/ 1231	0.3455	0.3925	
				Iceland deCODE GWAS	0.879 (0.775 - 0.997)	0.04487	557/ 89235	0.3857	0.4169	
					**OR (95% CI)**	**Z-score**	**P-value**	**df (Q)**	**HetI^2**	**HetPval**
				**Combined**	0.854 (0.813 - 0.896)	-6.382	1.7E-10	5	0	0.945
1	rs1795061	214409280	near *SMYD2*	NZ GWAS 1	1.033 (0.868 - 1.229)	0.716	608/ 612	0.3224	0.3153	
				NZ GWAS 2	1.364 (1.087 - 1.713)	0.007383	397/ 384	0.3456	0.2791	
				Aneurysm Consortium GWAS	1.135 (1.048 - 1.229)	0.001908	1846/ 5605	0.3342	0.3067	
				Netherlands GWAS	1.075 (0.943 - 1.225)	0.2806	840/ 2791	0.2952	0.2784	
				US (PA) GWAS	1.324 (1.163 - 1.507)	2.16E-05	724/ 1231	0.3517	0.2907	
				Iceland deCODE GWAS	1.133 (0.997 - 1.287)	0.0559	557/ 89235	0.4247	0.3954	
					**OR (95% CI)**	**Z-score**	**P-value**	**df (Q)**	**HetI^2**	**HetPval**
				**Combined**	1.154(1.097 - 1.214)	5.527	3.3E-07	5	47.9	0.087

CHR	SNP	POSITION	Region	Cohort	OR (95% CI)	P	Case/ Control	MAF_{AAA}	$MAF_{Control}$	
2	rs13382862	20882449	*C2orf43* and *GDF7*	NZ GWAS 1	0.886 (0.742 - 1.057)	0.1787	608/ 612	0.3424	0.3701	
				NZ GWAS 2	0.766 (0.618 - 0.948)	0.01432	397/ 384	0.3307	0.3922	
				Aneurysm Consortium GWAS	0.865 (0.799 - 0.937)	3.78E-04	1846/ 5605	0.3359	0.3689	
				Netherlands GWAS	0.862 (0.763 - 0.974)	0.0176	840/ 2791	0.3430	0.3761	
				US (PA) GWAS	0.855 (0.750 - 0.974)	0.01851	724/ 1231	0.3255	0.3609	
				Iceland deCODE GWAS	0.892 (0.782 - 1.016)	0.08629	557/ 89235	0.3420	0.3683	
					**OR (95% CI)**	**Z-score**	**P-value**	**df (Q)**	**HetI^2**	**HetPval**
				**Combined**	0.863 (0.820 - 0.907)	-5.75	8.8E-09	5	0	0.942
4	rs10029392	5616048	*EVC2*	NZ GWAS 1	2.071 (1.444 - 2.968)	5.47E-05	608/ 612	0.0801	0.0404	
				NZ GWAS 2	1.199 (0.733 - 1.961)	0.4692	397/ 384	0.0467	0.0393	
				Aneurysm Consortium GWAS	1.306 (1.081 - 1.578)	0.005588	1846/ 5605	0.0436	0.0338	
				Netherlands GWAS	1.180 (0.869 - 1.601)	0.2894	840/ 2791	0.0396	0.0358	
				US (PA) GWAS	1.166 (0.882 - 1.542)	0.28	724/ 1231	0.0517	0.0447	
				Iceland deCODE GWAS	1.402 (1.033 - 1.904)	0.03019	557/ 89235	0.0587	0.0445	
					**OR (95% CI)**	**Z-score**	**P-value**	**df (Q)**	**HetI^2**	**HetPval**
				**Combined**	1.331 (1.1851 - 1.495)	4.825	1.4E-06	5	32.1	0.195
5	rs12659791	74757758	*COL4A3BP*	NZ GWAS 1	1.284 (0.837 - 1.970)	0.2512	608/ 612	0.1763	0.1429	
				NZ GWAS 2	1.067 (0.808 - 1.409)	0.6466	397/ 384	0.1549	0.1466	
				Aneurysm Consortium GWAS	1.304 (1.181 - 1.440)	1.59E-07	1846/ 5605	0.1785	0.1428	
				Netherlands GWAS	1.216 (1.040 - 1.421)	0.01392	840/ 2791	0.1710	0.1480	
				US (PA) GWAS	1.077 (0.909 - 1.278)	0.3911	724/ 1231	0.1510	0.1417	
				Iceland deCODE GWAS	0.986 (0.819 - 1.188)	0.8844	557/ 89235	0.1264	0.1279	
					**OR (95% CI)**	**Z-score**	**P-value**	**df (Q)**	**HetI^2**	**HetPval**
				**Combined**	1.192 (1.115 - 1.274)	5.151	2.6E-07	5	45.5	0.102

CHR	SNP	POSITION	Region	Cohort	OR (95% CI)	P	Case/ Control	MAF_{AAA}	$MAF_{Control}$	
6	rs733590 proxy	36648364	*CDKN1A*	NZ GWAS 1	1.027 (0.870 - 1.213)	0.7498	608/ 612	0.3550	0.3489	
				NZ GWAS 2	1.108 (0.896 - 1.371)	0.3434	397/ 384	0.3405	0.3178	
				Aneurysm Consortium GWAS	1.128 (1.040 - 1.224)	0.003699	1846/ 5605	0.3612	0.3339	
				Netherlands GWAS	1.228 (1.085 - 1.391)	0.001181	840/ 2791	0.4027	0.3552	
				US (PA) GWAS	1.160 (1.024 - 1.315)	0.01962	724/ 1231	0.4052	0.3699	
				Iceland deCODE GWAS	1.056 (0.925 - 1.206)	0.42	557/ 89235	0.3449	0.3329	
					**OR (95% CI)**	**Z-score**	**P-value**	**df (Q)**	**$HetI^2$**	**HetPval**
				**Combined**	1.127 (1.072 - 1.186)	4.665	3.1E-06	5	0	0.546
8	rs3110425	107649626	*OXR1*	NZ GWAS 1	0.909 (0.769 - 1.075)	0.2639	608/ 612	0.3460	0.3679	
				NZ GWAS 2	0.852 (0.692 - 1.048)	0.1284	397/ 384	0.3469	0.3842	
				Aneurysm Consortium GWAS	0.883 (0.816 - 0.955)	0.001861	1846/ 5605	0.3310	0.3613	
				Netherlands GWAS	0.865 (0.764 - 0.980)	0.02225	840/ 2791	0.3340	0.3680	
				US (PA) GWAS	0.954 (0.840 - 1.085)	4.68E-01	724/ 1231	0.3650	0.3760	
				Iceland deCODE GWAS	0.849 (0.752 - 0.959)	0.012	557/ 89235	0.3480	0.3870	
					**OR (95% CI)**	**Z-score**	**P-value**	**df (Q)**	**$HetI^2$**	**HetPval**
				**Combined**	0.885 (0.843 - 0.93)	-4.873	1.1E-06	5	0	0.784
9	rs10757274	22096055	*ANRIL/ CDKN2B-AS1*	NZ GWAS 1	0.814 (0.694 - 0.955)	0.01152	608/ 612	0.4553	0.5066	
				NZ GWAS 2	0.922 (0.755 - 1.125)	0.4232	397/ 384	0.4484	0.4686	
				Aneurysm Consortium GWAS	0.842 (0.781 - 0.908)	7.91E-06	1846/ 5605	0.4707	0.5137	
				Netherlands GWAS	0.742 (0.660 - 0.834)	6.31E-07	840/ 2791	0.4746	0.5465	
				US (PA) GWAS	0.883 (0.782 - 0.997)	0.04425	724/ 1231	0.4793	0.5104	
				Iceland deCODE GWAS	0.838 (0.739 - 0.949)	0.005418	557/ 89235	0.5111	0.555	
					**OR (95% CI)**	**Z-score**	**P-value**	**df (Q)**	**$HetI^2$**	**HetPval**
				**Combined**	0.832 (0.793 - 0.872)	-7.612	2.7E-14	5	9.9	0.352

CHR	SNP	POSITION	Region	Cohort	OR (95% CI)	P	Case/ Control	MAF_{AAA}	$MAF_{Control}$	
9	rs10985349	124425243	*DAB2IP*	NZ GWAS 1	1.326 (1.078 - 1.630)	0.007382	608/ 612	0.2053	0.1631	
				NZ GWAS 2	1.443 (1.112 - 1.872)	0.005649	397/ 384	0.2091	0.1549	
				Aneurysm Consortium GWAS	1.159 (1.038 - 1.294)	0.008684	1846/ 5605	0.1666	0.1471	
				Netherlands GWAS	1.036 (0.890 - 1.206)	0.6491	840/ 2791	0.2192	0.2110	
				US (PA) GWAS	1.223 (1.050 - 1.425)	0.009782	724/ 1231	0.2055	0.1746	
				Iceland deCODE GWAS	1.212 (1.027 - 1.429)	0.02253	557/ 89235	0.2427	0.2130	
					**OR (95% CI)**	**Z-score**	**P-value**	**df (Q)**	**$HetI^2$**	**HetPval**
				**Combined**	1.185 (1.112 - 1.264)	5.198	2.0E-07	5	18.1	0.296
12	rs1385526	57532749	*LRP1*	NZ GWAS 1	0.863 (0.728 - 1.024)	0.09069	608/ 612	0.3165	0.3491	
				NZ GWAS 2	0.820 (0.659 - 1.021)	0.07616	397/ 384	0.3043	0.3478	
				Aneurysm Consortium GWAS	0.798 (0.737 - 0.865)	3.19E-08	1846/ 5605	0.3125	0.3629	
				Netherlands GWAS	0.893 (0.788 - 1.011)	0.07384	840/ 2791	0.3182	0.3452	
				US (PA) GWAS	0.902 (0.790 - 1.030)	0.1277	724/ 1231	0.3047	0.3269	
				Iceland deCODE GWAS	0.904 (0.796 - 1.028)	0.1233	557/ 89235	0.3945	0.4186	
					**OR (95% CI)**	**Z-score**	**P-value**	**df (Q)**	**$HetI^2$**	**HetPval**
				**Combined**	0.851 (0.809 - 0.895)	-6.295	3.1E-10	5	0	0.613
13	rs9316871	22861921	*LINC00540*	NZ GWAS 1	0.979 (0.804 - 1.190)	0.8279	608/ 612	0.2048	0.2083	
				NZ GWAS 2	0.678 (0.530 - 0.869)	0.002047	397/ 384	0.1755	0.2388	
				Aneurysm Consortium GWAS	0.823 (0.750 - 0.903)	3.92E-05	1846/ 5605	0.1923	0.2244	
				Netherlands GWAS	0.891 (0.770 - 1.030)	0.1191	840/ 2791	0.1931	0.2104	
				US (PA) GWAS	0.880 (0.760 - 1.020)	0.08905	724/ 1231	0.2103	0.2324	
				Iceland deCODE GWAS	0.969 (0.821 - 1.143)	0.7052	557/ 89235	0.1763	0.1809	
					**OR (95% CI)**	**Z-score**	**P-value**	**df (Q)**	**$HetI^2$**	**HetPval**
				**Combined**	0.864 (0.815 - 0.917)	-4.850	1.23E-06	5	33.2	0.187

CHR	SNP	POSITION	Region	Cohort	OR (95% CI)	P	Case/ Control	MAF_{AAA}	$MAF_{Control}$	
15	rs17189674	89040591	*DET1*	NZ GWAS 1	1.227 (0.966 - 1.559)	0.09398	608/ 612	0.1410	0.1180	
				NZ GWAS 2	1.595 (1.140 - 2.230)	0.006056	397/ 384	0.1273	0.0838	
				Aneurysm Consortium GWAS	1.241 (1.096 - 1.406)	6.51E-04	1846/ 5605	0.1123	0.0925	
				Netherlands GWAS	1.194 (0.991 - 1.438)	0.06165	840/ 2791	0.1314	0.1148	
				US (PA) GWAS	1.109 (0.916 - 1.344)	0.2889	724/ 1231	0.1167	0.1064	
				Iceland deCODE GWAS	1.181 (0.983 - 1.418)	0.07515	557/ 89235	0.1645	0.1448	
					**OR (95% CI)**	**Z-score**	**P-value**	**df (Q)**	**$HetI^2$**	**HetPval**
				**Combined**	1.216 (1.128 - 1.311)	5.091	3.6E-07	5	0	0.742
19	rs6511720	11202306	*LDLR*	NZ GWAS 1	0.611 (0.403 - 0.927)	0.01942	608/ 612	0.04043	0.06452	
				NZ GWAS 2	0.717 (0.514 - 1.001)	0.05007	397/ 384	0.08564	0.11550	
				Aneurysm Consortium GWAS	0.764 (0.676 - 0.863)	1.48E-05	1846/ 5605	0.09778	0.12430	
				Netherlands GWAS	0.650 (0.529 - 0.797)	3.74E-05	840/ 2791	0.08240	0.12150	
				US (PA) GWAS	0.742 (0.608 - 0.905)	0.003207	724/ 1231	0.09655	0.12590	
				Iceland deCODE GWAS	0.855 (0.684 - 1.068)	0.1668	557/ 89235	0.07480	0.08720	
					**OR (95% CI)**	**Z-score**	**P-value**	**df (Q)**	**$HetI^2$**	**HetPval**
				**Combined**	0.743 (0.685 - 0.806)	-7.151	8.6E-13	5	0	0.829
19	rs12980543	56096197	*ZNF579*	NZ GWAS 1	1.011 (0.832 - 1.227)	0.9147	608/ 612	0.2170	0.2152	
	rs11084402 proxy	56093365		NZ GWAS 2	1.280 (1.006 - 1.628)	0.04423	397/ 384	0.2418	0.1995	
				Aneurysm Consortium GWAS	1.077 (0.984 - 1.180)	0.1091	1846/ 5605	0.2153	0.2030	
				Netherlands GWAS	1.217 (1.051 - 1.409)	0.00854	840/ 2791	0.2159	0.1824	
				US (PA) GWAS	1.273 (1.094 - 1.481)	0.001733	724/ 1231	0.2131	0.1754	
				Iceland deCODE GWAS	1.281 (1.059 - 1.549)	0.01071	557/ 89235	0.1734	0.1430	
					**OR (95% CI)**	**Z-score**	**P-value**	**df (Q)**	**$HetI^2$**	**HetPval**
				**Combined**	1.152 (1.086 - 1.223)	4.669	3.0E-06	5	33.2	0.187

CHR	SNP	POSITION	Region	Cohort	OR (95% CI)	P	Case/ Control	MAF_{AAA}	$MAF_{Control}$	
20	rs6516091	6050622	near *FERMT1*	NZ GWAS 1	1.263 (0.991 - 1.609)	0.05848	608/ 612	0.1381	0.1126	
				NZ GWAS 2	1.271 (0.931 - 1.734)	0.13	397/ 384	0.1297	0.1050	
				Aneurysm Consortium GWAS	1.399 (1.261 - 1.551)	1.73E-10	1846/ 5605	0.1655	0.1242	
				Netherlands GWAS	1.187 (0.994 - 1.416)	0.05749	840/ 2791	0.1298	0.1150	
				US (PA) GWAS	1.186 (0.987 - 1.426)	0.06916	724/ 1231	0.1290	0.1110	
				Iceland deCODE GWAS	0.968 (0.783 - 1.197)	0.7619	557/ 89235	0.0938	0.0966	
					**OR (95% CI)**	**Z-score**	**P-value**	**df (Q)**	**$HetI^2$**	**HetPval**
				**Combined**	1.262 (1.177 - 1.354)	6.525	6.8E-11	5	56.2	0.044
20	rs58749629	44571317	Near *MMP9/ZNF335*	NZ GWAS 1	1.062 (0.846 - 1.333)	0.6044	608/ 612	0.1503	0.1427	
	rs3827066 proxy	44586023		NZ GWAS 2	1.101 (0.847 - 1.430)	0.4723	397/ 384	0.1827	0.1688	
				Aneurysm Consortium GWAS	1.237 (1.119 - 1.368)	2.97E-05	1846/ 5605	0.1743	0.1457	
				Netherlands GWAS	1.287 (1.099 - 1.506)	0.001752	840/ 2791	0.1761	0.1414	
				US (PA) GWAS	1.246 (1.067 - 1.456)	0.005513	724/ 1231	0.1972	0.1647	
				Iceland deCODE GWAS	1.325 (1.094 - 1.606)	0.003994	557/ 89235	0.1492	0.1193	
					**OR (95% CI)**	**Z-score**	**P-value**	**df (Q)**	**$HetI^2$**	**HetPval**
				**Combined**	1.233 (1.156 - 1.314)	6.371	1.9E-10	5	0	0.444
21	rs2836411	39819830	*ERG*	NZ GWAS 1	1.313 (1.113 - 1.548)	0.001228	608/ 612	0.3980	0.3350	
				NZ GWAS 2	1.076 (0.876 - 1.321)	0.4862	397/ 384	0.3823	0.3652	
				Aneurysm Consortium GWAS	1.132 (1.048 - 1.223)	0.00155	1846/ 5605	0.3814	0.3525	
				Netherlands GWAS	1.204 (1.065 - 1.361)	0.002983	840/ 2791	0.4016	0.3611	
				US (PA) GWAS	1.232 (1.086 - 1.397)	0.001164	724/ 1231	0.3821	0.3342	
				Iceland deCODE GWAS	1.000 (0.878 - 1.139)	0.9964	557/ 89235	0.3330	0.3331	
					**OR (95% CI)**	**Z-score**	**P-value**	**df (Q)**	**$HetI^2$**	**HetPval**
				**Combined**	1.149 (1.095 - 1.207)	5.573	2.5E-08	5	30.1	0.209

CHR	SNP	POSITION	Region	Cohort	OR (95% CI)	P	Case/ Control	MAF_{AAA}	$MAF_{Control}$	
X	RS5954362	140673423	*SPANXA1*	NZ GWAS 1	0.750 (0.556 - 1.007)	0.05496	474/ 450	0.2372	0.2930	
				NZ GWAS 2	0.862 (0.586 - 1.269)	0.4522	332/ 308	0.2310	0.2584	
				Aneurysm Consortium GWAS	0.584 (0.499 - 0.685)	1.81E-11	1815/ 2736	0.1894	0.2856	
					**OR (95% CI)**	**Z-score**	**P-value**	**df (Q)**	**$HetI^2$**	**HetPval**
				**Combined**	0.642 (0.563 - 0.732)	-6.105	1.0E-09	2	3.91	0.142

Online Table V: Summary of results for the combined (using the Maentel–Haenzel fixed effect method) validation study cohorts for the lead SNPs at putative AAA associated loci. The SNPs with $P<5x10^{-6}$ in the meta-analysis of 6 primary AAA GWAS datasets were genotyped in 8 different validation cohorts for a total of 5,232 AAA cases and 7,908 controls (see **Online Table II** for details on these cohorts). Results including MAFs for cases and controls in each individual cohort are shown in **Online Table VI**. Where a proxy SNP was typed the original lead SNP from the discovery study is shown above the proxy SNP typed in the validation study.

Chr	SNP	Position	Gene	Risk allele	Other allele	P	Direction	Phet	I^2
1	rs602633	109821511	Near *PSRC1 CELSR2 SORT1*	T	G	0.01	---++---	0.027	55.8
1	rs12133641	154428283	*IL6R*	A	G				
	rs4129267 proxySNP	*154426264*	*IL6R*	*T*	*C*	$1.81x10^{-4}$	+-------	0.294	17.2
1	rs1795061	214409280	near *SMYD2*	T	C	$3.49x10^{-4}$	+-++++++	0	70.3
2	rs13382862	20882449	near *C2orf43* and *GDF7*	A	G	0.360	---++++-	0.278	19.2
4	rs10029392	5616048	*EVC2*	T	G	0.267	+-+---++	0.195	29.2
5	rs12659791	74757758	*COL4A3BP*	T	C	0.966	-+-+---+	0.749	0.0
6	rs3176334	36648364	*CDKN1A*	C	T				
	rs733590 proxySNP	*36645203*	*CDKN1A*	*T*	*C*	0.789	+++-+-+-	0.754	0.0
8	*rs3110425*	107649626	*OXR1*	*T*	*C*	0.261	+-+++--+	0.268	20.4
9	rs10757274	22096055	*CDKN2BAS1/ANRIL*	A	G	$1.02x10^{-21}$	--------	0.001	64.2
9	rs10985349	124425243	*DAB2IP*	T	C	$2.30x10^{-5}$	-+++++++	0.4	3.9
12	rs1385526	57532749	*LRP1*	C	G	0.622	---+++-+	0.33	12.8
13	rs9316871	22861921	*LINC00540*	A	G	$8.28x10^{-5}$	-----+--	0.795	0.0
15	rs17189674	89040591	*DET1*	A	G	0.744	--++-++-	0.102	41.5
19	rs6511720	11202306	*LDLR*	T	G	$6.02x10^{-4}$	------++	0.003	68.2
19	rs12980543	56096197	*ZNF579*	A	G				
	rs11084402 proxySNP	*56093365*	*ZNF579*	*T*	*C*	0.364	+-+++++-	0.12	38.9
20	rs6516091	6050622	near *FERMT1*	A	G	0.867	+-+---++	0.104	41.1
20	rs58749629	44571317	near *PCIF1 ZNF335 MMP9*	A	G				
	rs3827066 proxySNP	*44586023*	*near PCIF1 ZNF335 MMP9*	*T*	*C*	$2.00x10^{-8}$	++++++++	0.3	16.5
21	rs2836411	39819830	*ERG*	T	C	0.011	+++-++++	0.203	28.3
X	rs5954362	140673423	*SPANXA1*	G	C	0.172	-+-+--	0.005	73.2

Online Table VI: Results of validation for the lead SNPs (combined using the Maentel–Haenzel fixed effect method) at putative AAA loci identified in the meta-analysis of GWAS. The SNPs with $P<5x10^{-6}$ in the meta-analysis of 6 primary AAA GWAS datasets were genotyped in 8 different validation cohorts for a total of up to 5,232 AAA cases and 7,908 controls (see **Online Table II** for details on these cohorts). This table spans this and the following 5 pages. Where a proxy SNP is indicated the results are for that proxy SNP.

Chromosome	SNP	POSITION	Region	Cohort	OR (95% CI)	Case/control	MAFAAA	MAFControl	HWEControl	HWEAAA
1	rs602633	109821511	Near PSRC1 CELSR2 SORT1	AC	0.885 (0.781-1.002)	1236/2196	0.191	0.211	0.079	0.799
				US2	0.864 (0.757-0.987)	1157/1374	0.211	0.236	0.724	0.248
				NZ	0.933 (0.794-1.095)	753/1237	0.201	0.213	0.473	0.183
				Italy	1.201 (0.995-1.449)	718/636	0.220	0.190	0.200	0.210
				Poland	1.133 (0.904-1.421)	443/474	0.218	0.197	0.652	0.995
				eMERGE	0.784 (0.639-0.963)	330/1648	0.203	0.245	0.005	0.248
				Belgium	0.8 (0.591-1.082)	302/216	0.192	0.229	0.198	0.959
				Canada	0.803 (0.533-1.211)	126/118	0.230	0.271	0.881	0.870
					**OR (95% CI)**	**Z-score**	**P-value**	**df (Q)**	**HetPVal**	**I-squared**
				**Combined:**	**0.92 (0.863-0.98)**	**-2.582**	**0.010**	**7**	**0.027**	**55.770**
1	rs12133641<br>rs4129267 proxy	154428283<br>154426264	IL6R	AC	1.001 (0.904-1.108)	1236/2196	0.386	0.386	0.706	0.293
				US2	0.879 (0.783-0.986)	1137/1324	0.369	0.400	0.798	0.689
				NZ	0.835 (0.732-0.954)	753/1237	0.377	0.420	0.155	0.022
				Italy	0.897 (0.765-1.052)	714/585	0.364	0.390	0.286	0.179
				Poland	0.925 (0.768-1.114)	480/481	0.353	0.371	0.731	0.000
				eMERGE	0.927 (0.781-1.099)	345/1724	0.354	0.371	0.137	0.840
				Belgium	0.767 (0.606-0.971)	334/256	0.361	0.424	0.440	0.410
				Canada	0.761 (0.541-1.072)	139/133	0.381	0.447	0.025	0.427
					**OR (95% CI)**	**Z-score**	**P-value**	**df (Q)**	**HetPVal**	**I-squared**
				**Combined:**	**0.904 (0.857-0.953)**	**-3.743**	**1.81E-04**	**7**	**0.294**	**17.200**
1	rs1795061	214409280	Near SMYD2	AC	1.171 (1.053-1.302)	1236/2196	0.332	0.298	0.869	0.360
				US2	0.91 (0.808-1.024)	1172/1386	0.301	0.321	0.469	0.892
				NZ	1.077 (0.939-1.236)	753/1237	0.336	0.319	0.060	0.026
				Italy	1.434 (1.21-1.698)	761/558	0.340	0.264	0.226	0.071
				Poland	1.025 (0.848-1.239)	470/487	0.332	0.326	0.823	0.025
				eMERGE	1.088 (0.915-1.294)	340/1679	0.347	0.328	0.216	0.991
				Belgium	1.28 (0.996-1.646)	335/260	0.327	0.275	0.605	0.583
				Canada	1.32 (0.916-1.905)	132/132	0.352	0.292	0.075	0.364
					**OR (95% CI)**	**Z-score**	**P-value**	**df (Q)**	**HetPVal**	**I-squared**
				**Combined:**	**1.105 (1.046-1.168)**	**3.576**	**3.49E-04**	**7**	**0.000**	**70.300**

Chromosome	SNP	POSITION	Region	Cohort	OR (95% CI)	Case/control	MAFAAA	MAFControl	HWEControl	HWEAAA
2	rs13382862	20882449	near C2orf43 and GDF7	AC	0.942 (0.85-1.045)	1236/2196	0.358	0.372	0.224	0.156
				US2	0.94 (0.836-1.057)	1109/1386	0.346	0.360	0.103	0.291
				NZ	0.87 (0.759-0.997)	753/1237	0.335	0.366	0.140	0.741
				Italy	1.109 (0.931-1.321)	727/558	0.287	0.266	0.158	0.345
				Poland	1.099 (0.906-1.332)	450/452	0.366	0.344	0.465	0.098
				eMERGE	1.083 (0.913-1.285)	335/1645	0.388	0.369	0.970	0.918
				Belgium	1.024 (0.794-1.319)	310/256	0.310	0.305	0.821	0.209
				Canada	0.998 (0.705-1.413)	142/131	0.370	0.370	0.720	0.021
					**OR (95% CI)**	**Z-score**	**P-value**	**df (Q)**	**HetPVal**	**I-squared**
				**Combined:**	**0.975 (0.923-1.029)**	**-0.915**	**0.360**	**7**	**0.278**	**19.156**
4	rs10029392	5616048	EVC2	AC	1.014 (0.814-1.263)	1698/2209	0.044	0.043	0.655	0.110
				US2*	0.876 (0.678-1.132)	1169/1387	0.046	0.052	0.217	0.088
				NZ*	1.233 (0.92-1.651)	753/1237	0.057	0.047	0.809	0.106
				Italy	0.767 (0.588-0.999)	678/556	0.088	0.112	0.423	0.709
				Poland*	0.809 (0.6-1.092)	472/481	0.091	0.110	0.074	0.963
				eMERGE	0.921 (0.642-1.321)	343/1707	0.054	0.058	0.998	0.006
				Belgium*	1.09 (0.545-2.18)	335/225	0.030	0.027	0.652	0.573
				Canada*	2.2 (0.754-6.422)	133/130	0.041	0.019	0.823	0.091
					**OR (95% CI)**	**Z-score**	**P-value**	**df (Q)**	**HetPVal**	**I-squared**
				**Combined:**	**0.94 (0.843-1.049)**	**-1.111**	**0.267**	**7**	**0.195**	**29.200**
5	rs12659791	74757758	COL4A3BP	AC	0.963 (0.839-1.105)	1236/2196	0.149	0.154	0.071	0.307
				US2	1.063 (0.91-1.242)	1151/1371	0.153	0.145	0.398	0.663
				NZ	0.959 (0.802-1.148)	753/1237	0.152	0.158	0.149	0.581
				Italy	1.051 (0.83-1.332)	732/439	0.151	0.145	0.484	0.177
				Poland	0.976 (0.771-1.236)	486/488	0.169	0.172	0.002	0.553
				eMERGE	0.979 (0.773-1.241)	345/1723	0.138	0.140	0.818	0.807
				Belgium	0.91 (0.669-1.237)	339/266	0.156	0.169	0.298	0.126
				Canada	1.62 (0.898-2.921)	105/91	0.167	0.110	0.239	0.953
					**OR (95% CI)**	**Z-score**	**P-value**	**df (Q)**	**HetPVal**	**I-squared**
				**Combined:**	**0.998 (0.929-1.073)**	**-0.043**	**0.966**	**7**	**0.749**	**0.000**

Chromosome	SNP	POSITION	Region	Cohort	OR (95% CI)	Case/control	MAFAAA	MAFControl	HWEControl	HWEAAA
6	rs3176334	36648364	CDKN1A	AC	1.042 (0.94-1.156)	1236/2196	0.356	0.347	0.523	0.412
	rs733590 proxy	36645203		US2	1.006 (0.897-1.127)	1157/1374	0.379	0.378	0.871	0.072
				NZ	1.026 (0.894-1.178)	753/1237	0.333	0.327	0.268	0.428
				Italy	0.978 (0.831-1.149)	733/546	0.371	0.376	0.163	0.992
				Poland	1.021 (0.846-1.232)	455/453	0.398	0.393	0.702	0.557
				eMERGE	0.893 (0.748-1.066)	318/1599	0.357	0.383	0.525	0.391
				Belgium	1.127 (0.888-1.43)	331/254	0.394	0.366	0.776	0.917
				Canada	0.84 (0.585-1.205)	142/132	0.296	0.333	0.361	0.150
					**OR (95% CI)**	**Z-score**	**P-value**	**df (Q)**	**HetPVal**	**I-squared**
				**Combined:**	**1.007 (0.955-1.063)**	**0.2678**	**0.789**	**7**	**0.754**	**0.000**
8	rs3110425	107649626	OXR1	AC	1.079 (0.974-1.196)	1225/2167	0.374	0.356	0.111	0.315
				US2	0.933 (0.826-1.053)	987/1323	0.355	0.371	0.568	0.248
				NZ	1.088 (0.949-1.247)	704/1174	0.382	0.361	0.107	0.279
				Italy	1.058 (0.878-1.275)	532/445	0.359	0.346	0.883	0.226
				Poland	1.214 (0.999-1.475	449/457	0.363	0.319	0.770	0.164
				eMERGE	0.942 (0.794-1.118)	342/1711	0.360	0.373	0.971	0.448
				Belgium	0.910 (0.713-1.163)	313/246	0.356	0.378	0.558	0.672
				Canada	1.036 (0.725-1.481)	126/118	0.230	0.271	0.881	0.870
					**OR (95% CI)**	**Z-score**	**P-value**	**df (Q)**	**HetPVal**	**I-squared**
				**Combined:**	**1.032 (0.977-1.090)**	**1.124**	**0.261**	**7**	**0.268**	**20.420**
9	rs10757274	22096055	ANRILCDKN2BAS1/	AC	0.831 (0.752-0.917)	1236/2196	0.456	0.502	0.273	0.163
				US	0.748 (0.67-0.836)	1162/1382	0.451	0.523	0.099	0.622
				NZ	0.885 (0.777-1.007)	753/1237	0.466	0.497	0.561	0.038
				Italy	0.63 (0.527-0.753)	540/464	0.371	0.484	0.236	0.052
				Poland	0.679 (0.565-0.816)	451/468	0.463	0.560	0.616	0.551
				eMERGE	0.68 (0.576-0.804)	336/1696	0.435	0.530	0.333	0.922
				Belgium	0.959 (0.757-1.214)	313/248	0.455	0.466	0.333	0.977
				Canada	0.674 (0.468-0.97)	117/117	0.427	0.526	0.535	0.320
					**OR (95% CI)**	**Z-score**	**P-value**	**df (Q)**	**HetPVal**	**I-squared**
				**Combined:**	**0.774 (0.735-0.816)**	**-9.575**	**1.02E-21**	**7**	**0.001**	**64.200**

Chromosome	SNP	POSITION	Region	Cohort	OR (95% CI)	Case/control	MAFAAA	MAFControl	HWEControl	HWEAAA
9	rs10985349	124425243	DAB2IP	AC	0.998 (0.876-1.135)	1236/2196	0.179	0.179	0.878	0.696
				US2	1.208 (1.051-1.387)	1171/1385	0.211	0.181	0.625	0.165
				NZ	1.266 (1.072-1.495)	753/1237	0.199	0.164	0.865	0.720
				Italy	1.227 (0.975-1.544)	729/620	0.138	0.115	0.061	0.327
				Poland	1.182 (0.947-1.476)	485/488	0.215	0.189	0.322	0.228
				eMERGE	1.215 (0.969-1.523)	299/1544	0.189	0.161	0.346	0.381
				Belgium	1.139 (0.848-1.53)	338/266	0.192	0.173	0.682	0.220
				Canada	1.304 (0.861-1.975)	149/134	0.221	0.179	0.177	0.116
					**OR (95% CI)**	**Z-score**	**P-value**	**df (Q)**	**HetPVal**	**I-squared**
				**Combined:**	**1.155 (1.081-1.235)**	**4.233**	**2.30E-05**	**7**	**0.400**	**3.900**
12	rs1385526	57532749	LRP1	AC	0.983 (0.886-1.092)	1236/2196	0.339	0.343	0.293	0.297
				US2	0.905 (0.804-1.019)	1161/1382	0.306	0.328	0.668	0.221
				NZ	0.918 (0.801-1.052)	753/1237	0.333	0.352	0.160	0.840
				Italy	1.135 (0.942-1.366)	509/493	0.351	0.323	0.072	0.392
				Poland	1.058 (0.875-1.28)	453/480	0.359	0.346	0.083	0.580
				eMERGE	1.124 (0.944-1.339)	342/1695	0.336	0.311	0.695	0.170
				Belgium	0.966 (0.749-1.244)	324/246	0.306	0.313	0.744	0.647
				Canada	1.031 (0.706-1.506)	132/120	0.311	0.304	0.181	0.607
					**OR (95% CI)**	**Z-score**	**P-value**	**df (Q)**	**HetPVal**	**I-squared**
				**Combined:**	**0.986 (0.933-1.042)**	**-0.493**	**0.622**	**7**	**0.330**	**12.800**
13	rs9316871	22861921	LINC00540	AC	0.905 (0.801-1.023)	1236/2196	0.199	0.216	0.094	0.193
				US2	0.845 (0.738-0.966)	1176/1384	0.202	0.231	0.624	0.052
				NZ	0.834 (0.711-0.978)	753/1237	0.195	0.225	0.210	0.727
				Italy	0.925 (0.776-1.102)	621/646	0.262	0.278	0.178	0.107
				Poland	0.884 (0.721-1.085)	469/483	0.251	0.274	0.222	0.038
				eMERGE	1.008 (0.828-1.226)	345/1724	0.223	0.222	0.049	0.954
				Belgium	0.79 (0.592-1.053)	330/251	0.185	0.223	0.854	0.920
				Canada	0.798 (0.515-1.235)	133/125	0.177	0.212	0.004	0.271
					**OR (95% CI)**	**Z-score**	**P-value**	**df (Q)**	**HetPVal**	**I-squared**
				**Combined:**	**0.883 (0.83-0.94)**	**-3.936**	**8.28E-05**	**7**	**0.795**	**0.000**

Chromosome	SNP	POSITION	Region	Cohort	OR (95% CI)	Case/control	MAFAAA	MAFControl	HWEControl	HWEAAA
15	rs17189674	89040591	DET1	AC	0.889 (0.763-1.036)	1236/2196	0.115	0.128	0.264	0.949
				US2	0.934 (0.781-1.116)	1170/1381	0.104	0.110	0.000	0.846
				NZ	1.301 (1.065-1.588)	753/1237	0.130	0.103	0.499	0.429
				Italy	1.115 (0.862-1.441)	616/872	0.127	0.116	0.113	0.276
				Poland	0.912 (0.686-1.212)	488/489	0.105	0.113	0.559	0.260
				eMERGE	1.072 (0.815-1.411)	320/1649	0.108	0.101	0.553	0.114
				Belgium	1.214 (0.849-1.737)	340/266	0.125	0.105	0.973	0.403
				Canada	0.927 (0.557-1.542)	201/137	0.113	0.120	0.105	0.944
					**OR (95% CI)**	**Z-score**	**P-value**	**df (Q)**	**HetPVal**	**I-squared**
				**Combined:**	**1.014 (0.935-1.099)**	**0.327**	**0.744**	**7**	**0.102**	**41.500**
19	rs6511720	11202306	LDLR	AC	0.969 (0.826-1.136)	1236/2196	0.107	0.110	0.144	0.384
				US2	0.93 (0.792-1.092)	1166/1383	0.132	0.141	0.000	0.000
				NZ	0.849 (0.69-1.045)	753/1237	0.103	0.119	0.737	0.454
				Italy	0.566 (0.436-0.736)	667/567	0.079	0.132	0.736	0.242
				Poland	0.607 (0.454-0.812)	477/479	0.087	0.136	0.944	0.132
				eMERGE	0.933 (0.709-1.227)	320/1639	0.108	0.101	0.553	0.114
				Belgium	1.162 (0.817-1.653)	336/260	0.129	0.113	0.102	0.079
				Canada	1.025 (0.63-1.67)	141/133	0.138	0.135	0.676	0.231
					**OR (95% CI)**	**Z-score**	**P-value**	**df (Q)**	**HetPVal**	**I-squared**
				**Combined:**	**0.868 (0.801-0.941)**	**-3.431**	**6.02E-04**	**7**	**0.003**	**68.200**
19	rs12980543	56096197	near ZNF579	AC	1.033 (0.913-1.169)	1217/2169	0.205	0.199	0.239	0.604
	rs11084402 proxy	56093365	near ZNF579	US2	0.889 (0.771-1.025)	1164/1386	0.176	0.193	0.216	0.988
				NZ	1.113 (0.953-1.3)	737/1217	0.230	0.212	0.665	0.832
				Italy	1.075 (0.871-1.326)	648/503	0.196	0.185	0.124	0.203
				Poland	1.014 (0.802-1.281)	474/485	0.177	0.176	0.737	0.780
				eMERGE	1.145 (0.935-1.402)	344/1723	0.209	0.188	0.967	0.200
				Belgium	1.293 (0.97-1.722)	337/262	0.223	0.181	0.068	0.140
				Canada	0.73 (0.486-1.096)	135/125	0.207	0.264	0.130	0.142
					**OR (95% CI)**	**Z-score**	**P-value**	**df (Q)**	**HetPVal**	**I-squared**
				**Combined:**	**1.03 (0.966-1.099)**	**0.908**	**0.364**	**7.000**	**0.120**	**38.900**

Chromosome	SNP	POSITION	Region	Cohort	OR (95% CI)	Case/control	MAFAAA	MAFControl	HWEControl	HWEAAA
20	rs6516091	6050622	near FERMT1	AC	1.112 (0.961-1.287)	1236/2196	0.136	0.124	0.911	0.431
				US2	0.901 (0.768-1.057)	1173/1384	0.132	0.144	0.625	0.149
				NZ	1.094 (0.9-1.33)	753/1237	0.131	0.121	0.682	0.424
				Italy	0.834 (0.667-1.043)	715/589	0.137	0.160	0.357	0.152
				Poland	0.824 (0.619-1.096)	485/488	0.100	0.119	0.363	0.002
				eMERGE	0.94 (0.741-1.192)	343/1716	0.137	0.145	0.047	0.511
				Belgium	1.355 (0.947-1.939)	339/266	0.133	0.102	0.065	0.153
				Canada	1.089 (0.671-1.768)	149/137	0.138	0.128	0.857	0.415
					**OR (95% CI)**	**Z-score**	**P-value**	**df (Q)**	**HetPVal**	**I-squared**
				**Combined:**	**0.994 (0.921-1.072)**	**-0.167**	**0.867**	**7**	**0.104**	**41.100**
20	rs58749629	44571317	near PCIF1 ZNF335 MMP9	AC	1.076 (0.94-1.233)	1236/2196	0.160	0.151	0.688	0.617
	rs3827066 proxy	44586023	near PCIF1 ZNF335 MMP9	US2	1.197 (1.035-1.385)	1171/1384	0.185	0.160	0.510	0.814
				NZ	1.372 (1.153-1.632)	753/1237	0.185	0.142	0.132	0.899
				Italy	1.263 (1.05-1.518)	709/579	0.256	0.214	0.380	0.275
				Poland	1.373 (1.092-1.728)	487/486	0.210	0.163	0.958	0.907
				eMERGE	1.077 (0.86-1.348)	345/1713	0.159	0.150	0.160	0.926
				Belgium	1.291 (0.956-1.743)	340/265	0.196	0.158	0.280	0.733
				Canada	1.469 (0.983-2.197)	146/132	0.260	0.193	0.023	0.028
					**OR (95% CI)**	**Z-score**	**P-value**	**df (Q)**	**HetPVal**	**I-squared**
				**Combined:**	**1.213 (1.134-1.298)**	**5.616**	**2.00E-08**	**7**	**0.300**	**16.500**
21	rs2836411	39819830	ERG	AC	1.05 (0.947-1.164)	1236/2196	0.367	0.355	0.409	0.483
				US2	1.085 (0.966-1.218)	1171/1382	0.350	0.331	0.073	0.877
				NZ	1.171 (1.023-1.339)	753/1237	0.387	0.351	0.733	0.258
				Italy	0.898 (0.757-1.065)	706/625	0.263	0.285	0.088	0.332
				Poland	1.125 (0.935-1.354)	482/484	0.380	0.352	0.990	0.769
				eMERGE	1.045 (0.88-1.241)	345/1724	0.349	0.339	0.485	0.797
				Belgium	1.01 (0.797-1.281)	339/264	0.358	0.356	0.224	0.715
				Canada	1.457 (1.04-2.041)	150/137	0.440	0.350	0.422	0.314
					**OR (95% CI)**	**Z-score**	**P-value**	**df (Q)**	**HetPVal**	**I-squared**
				**Combined:**	**1.072 (1.016-1.131)**	**2.533**	**0.011**	**7**	**0.203**	**28.300**
X	rs5954362	140673423	SPANXA1	Males only						
				NZ	0.816 (0.636-1.047)	585/762	0.248	0.288		
				Belgium	1.301 (0.972-1.741)	319/183	0.310	0.257		
				Canada	0.536 (0.294-0.978)	155/32	0.272	0.411		
				eMERGE	1.189 (1.044-1.353)	1145/1134	0.311	0.276		
				Poland	0.948 (0.759-1.186)	426/355	0.286	0.297		
					**OR (95% CI)**	**Z-score**	**P-value**	**df (Q)**	**HetPVal**	**I-squared**
				**Combined:**	**1.069 (0.972-1.175)**	**1.366**	**0.172**	**4**	**0.005**	**73.200**

Online Table VII: Combined results from GWAS meta-analysis and validation studies. Shaded rows indicate validated AAA risk loci that are also shown in Table 1 in the main text. A fixed effect meta-analysis was performed using a Maentel–Haenzel method with the genome-wide *P*-value significance threshold being set at 5x10^{-8}.

							Meta-GWAS (lambda adjusted)			Combined validation studies					Combined meta-analysis and validation							
Chr	SNP	Position	Gene	Minor allele	Other allele	MAF	P-value	Direction	HetPVal	OR	L95	U95	Z-score	P-value	OR	L95	U95	Z-score	P-value	df	HetPVal	
1*	rs602633	109821511	Near *PSRC1 CELSR2 SORT1*	T	**G**	0.199	1.72x10^{-07}	------	0.097	0.920	0.863	0.980	-2.582	9.83x10^{-03}	0.879	0.842	0.918	-5.801	6.58x10^{-09}	13	7.60x10^{-03}	[illegible]
*1**	*rs12133641*	*154428283*	*IL6R*	***A***	*G*		*1.67x10^{-10}*	*++++++*	*0.903*													
1	rs4129267 (proxy)	154426264	*IL6R*	T	**C**	0.370	9.26x10^{-10}	------	0.886	0.904	0.857	0.953	-3.743	1.81x10^{-04}	0.876	0.846	0.908	-7.232	4.76x10^{-13}	13	0.478	
1	rs1795061	214409280	near *SMYD2*	**T**	C	0.336	1.79x10^{-07}	++++++	0.069	1.105	1.046	1.168	3.576	3.49x10^{-04}	1.131	1.090	1.174	6.486	8.80x10^{-11}	13	1.14x10^{-03}	[illegible]
2	rs13382862	20882449	near *C2orf43* and *GDF7*	A	**G**	0.341	3.03x10^{-08}	------	0.878	0.975	0.923	1.029	-0.915	3.60x10^{-01}	0.913	0.880	0.947	-4.845	1.3x10^{-06}	13	8.01x10^{-02}	[illegible]
4	rs10029392	5616048	*EVC2*	**T**	G	0.052	4.60x10^{-06}	++++++	0.147	0.940	0.843	1.049	-1.111	2.67x10^{-01}	1.107	1.022	1.198	2.496	1.25x10^{-02}	13	6.51x10^{-04}	[illegible]
5	rs12659791	74757758	*COL4A3BP*	T	**C**	0.159	2.28x10^{-06}	--+---	0.105	0.998	0.929	1.073	-0.043	9.66x10^{-01}	1.098	1.046	1.153	3.752	1.8x10^{-04}	13	1.69x10^{-02}	[illegible]
6	*rs3176334*	*36648364*	*CDKN1A*	*T*	***C***		*1.50x10^{-06}*	------	*0.627*													
6	rs733590 (proxy)	36645203	*CDKN1A*	T	**C**	0.367	8.74x10^{-06}	------	0.584	1.007	0.955	1.063	0.2678	7.89x10^{-01}	1.070	1.031	1.110	3.588	3.33x10^{-04}	13	0.183	[illegible]
9*	rs10757274	22096055	*CDKN2BAS1/ANRIL*	A	**G**	0.351	2.32 x10^{-13}	------	0.520	0.774	0.735	0.816	-9.575	1.02x10^{-21}	0.806	0.778	0.834	-12.069	1.54x10^{-33}	13	5.94x10^{-03}	[illegible]
9*	rs10985349	124425243	*DAB2IP*	**T**	C	0.462	8.98x10^{-07}	++++++	0.181	1.155	1.081	1.235	4.233	2.30x10^{-05}	1.171	1.118	1.226	6.682	2.4x10^{-11}	13	3.52x10^{-01}	
12*	rs1385526	57532749	*LRP1*	C	**G**	0.195	1.31x10^{-09}	------	0.597	0.986	0.933	1.042	-0.493	6.22x10^{-01}	0.910	0.877	0.944	-4.980	6.4x10^{-07}	13	9.38x10^{-03}	[illegible]
13	rs9316871	22861921	*LINC00540*	**A**	G	0.328	5.95x10^{-06}	++++++	0.143	0.883	0.830	0.940	-3.936	8.28x10^{-05}	0.873	0.837	0.911	-6.227	4.8x10^{-10}	13	0.488	
15	rs17189674	89040591	*DET1*	**A**	G	0.201	1.05x10^{-06}	++++++	0.663	1.014	0.935	1.099	0.327	7.44x10^{-01}	1.118	1.058	1.181	3.957	7.59x10^{-05}	13	1.71x10^{-02}	[illegible]
19*	rs6511720	11202306	*LDLR*	T	**G**	0.122	5.71x10^{-12}	------	0.679	0.868	0.801	0.941	-3.431	6.02x10^{-04}	0.804	0.759	0.851	-7.472	7.9x10^{-14}	13	1.53x10^{-03}	[illegible]
19	rs12980543	56096197	*near ZNF579*	**A**	G		2.30x10^{-06}	++++++	0.301													
19	rs11084402 proxy	56093365	near *ZNF579*	**T**	C	0.206	4.33x10^{-06}	++++++	0.218	1.030	0.966	1.099	0.908	3.64x10^{-01}	1.095	1.048	1.144	4.050	5.1x10^{-05}	13	0.019	[illegible]
20	rs6516091	6050622	near *FERMT1*	**A**	G	0.135	3.82x10^{-09}	++-+++	0.027	0.994	0.921	1.072	-0.167	8.67x10^{-01}	1.131	1.074	1.190	4.680	2.9x10^{-06}	13	4.01x10^{-05}	[illegible]
20	rs58749629	44571317	near *PCIF1 ZNF335 MMP9*	***A***	*G*		*7.97x10^{-10}*	++++++	*0.760*													
20	rs3827066 proxy	44586023	near *PCIF1 ZNF335 MMP9*	**T**	C	0.179	9.18x10^{-10}	++++++	0.729	1.213	1.134	1.298	5.616	2.00x10^{-08}	1.223	1.168	1.281	8.486	2.1x10^{-17}	13	0.552	
21	rs2836411	39819830	*ERG*	**T**	C	0.369	1.53x10^{-07}	++-+++	0.103	1.072	1.016	1.131	2.533	1.13x10^{-02}	1.113	1.074	1.154	5.823	5.8x10^{-09}	13	4.83x10^{-02}	[illegible]
X	rs5954362	140673423	*SPANXA1*	C	**G**	0.241	1.0310^{-09}	---	0.142	1.069	0.972	1.175	1.366	1.72x10^{-01}	0.896	0.829	0.967	-2.807	5.0x10^{-03}	7	4.18x10^{-10}	[illegible]

*Loci previously identified as associated with AAA.

Online Table VIII: Sensitivity analysis comparing results from the combined GWAS meta-analysis and validation studies using a fixed effects model with a random-effects model. Results for loci surpassing the threshold for genome-wide significance are shown in bold. *Loci previously identified as associated with AAA

										Fixed effects model		Random effects model	
**Chr**	**SNP**	**Position**	**Gene**	**Minor allele**	**Other allele**	**MAF**	**df**	**HetPVal**	**I^2**	**OR (95% CI)**	**P-value**	**OR (95% CI)**	**P-value**
1*	rs602633	109821511	Near *PSRC1 CELSR2 SORT1*	T	**G**	0.199	13	7.60x10^{-03}	54.5	**0.879 (0.842 - 0.918)**	**6.58x10^{-09}**	**0.881 (0.822 - 0.943)**	**3.18x10^{-9}**
*1**	*rs12133641*	*154428283*	*IL6R*	***A***	*G*								
1	rs4129267 (proxy)	154426264	*IL6R*	T	**C**	0.370	13	0.478	0.0	**0.876 (0.846 - 0.908)**	**4.76x10^{-13}**	**0.876 (0.846 - 0.908)**	**1.03x10^{-12}**
1	rs1795061	214409280	near *SMYD2*	**T**	C	0.336	13	1.14x10^{-03}	61.9	**1.131 (1.090 - 1.174)**	**8.80x10^{-11}**	**1.142 (1.07 - 1.218)**	**3.47x10^{-12}**
2	rs13382862	20882449	near *C2orf43* and *GDF7*	A	**G**	0.341	13	8.01x10^{-02}	37.1	0.913 (0.880 - 0.947)	1.3x10^{-06}	0.921 (0.877 - 0.968)	2.05x10^{-6}
4	rs10029392	5616048	*EVC2*	**T**	G	0.052	13	6.51x10^{-04}	63.6	1.107 (1.022 - 1.198)	1.25x10^{-02}	1.120 (0.973 - 1.289)	1.88x10^{-4}
5	rs12659791	74757758	*COL4A3BP*	T	**C**	0.159	13	1.69x10^{-02}	50.1	1.098 (1.046 - 1.153)	1.8x10^{-04}	1.071 (0.993 - 1.156)	8.18x10^{-6}
6	*rs3176334*	*36648364*	*CDKN1A*	*T*	***C***								
6	rs733590 (proxy)	36645203	*CDKN1A*	T	**C**	0.367	13	0.183	25.2	1.070 (1.031 - 1.11)	3.33x10^{-04}	1.064 (1.017 - 1.112)	5.06x10^{-4}
9*	rs10757274	22096055	*CDKN2BAS1/ANRIL*	A	**G**	0.351	13	5.94x10^{-03}	55.6	**0.806 (0.778 - 0.834)**	**1.54x10^{-33}**	**0.797 (0.753 - 0.843)**	**1.21x10^{-33}**
9*	rs10985349	124425243	*DAB2IP*	**T**	C	0.462	13	3.52x10^{-01}	9.2	**1.171 (1.118 - 1.226)**	**2.4x10^{-11}**	**1.174 (1.117 - 1.233)**	**4.47x10^{-11}**
12*	rs1385526	57532749	*LRP1*	C	**G**	0.195	13	9.38x10^{-03}	53.4	0.910 (0.877 - 0.944)	6.4x10^{-07}	0.930 (0.877 - 0.986)	6.541x10^{-8}
13	rs9316871	22861921	*LINC00540*	**A**	G	0.328	13	0.488	0.0	**0.873 (0.837 - 0.911)**	**4.8x10^{-10}**	**0.873 (0.837 - 0.911)**	**9.98x10^{-10}**
15	rs17189674	89040591	*DET1*	**A**	G	0.201	13	1.71x10^{-02}	50.0	1.118 (1.058 - 1.181)	7.59x10^{-05}	1.120 (1.031 - 1.217)	1.26x10^{-5}
19*	rs6511720	11202306	*LDLR*	T	**G**	0.122	13	1.53x10^{-03}	61.0	**0.804 (0.759 - 0.851)**	**7.9x10^{-14}**	**0.795 (0.72 - 0.878)**	**7.19x10^{-15}**
19	rs12980543	56096197	*near ZNF579*	**A**	G								
19	rs11084402 (proxy)	56093365	near *ZNF579*	**T**	C	0.206	13	0.019	49.4	1.095 (1.048 - 1.144)	5.1x10^{-05}	1.101 (1.031 - 1.176)	2.51x10^{-5}
20	rs6516091	6050622	near *FERMT1*	**A**	G	0.135	13	4.01x10^{-05}	70.0	1.131 (1.074 - 1.19)	2.9x10^{-06}	**1.088 (0.983 - 1.203)**	**6.58x10^{-10}**
20	rs58749629	44571317	near *PCIF1 ZNF335 MMP9*	***A***	*G*								
20	rs3827066 (proxy)	44586023	near *PCIF1 ZNF335 MMP9*	**T**	C	0.179	13	0.552	0.0	**1.223 (1.168 - 1.281)**	**2.1x10^{-17}**	**1.223 (1.168 - 1.281)**	**6.12x10^{-17}**
21	rs2836411	39819830	*ERG*	**T**	C	0.369	13	4.83x10^{-02}	42.2	**1.113 (1.074 - 1.154)**	**5.8x10^{-09}**	**1.112 (1.057 - 1.17)**	**7.07x10^{-9}**
X	rs5954362	140673423	*SPANXA1*	C	**G**	0.241	7	4.18x10^{-10}	87.9	0.896 (0.829 - 0.967)	5.0x10^{-03}	**0.857 (0.672 - 1.092)**	**3.08x10^{-11}**

Online Figure II: Regional association plots for previously reported AAA risk loci.

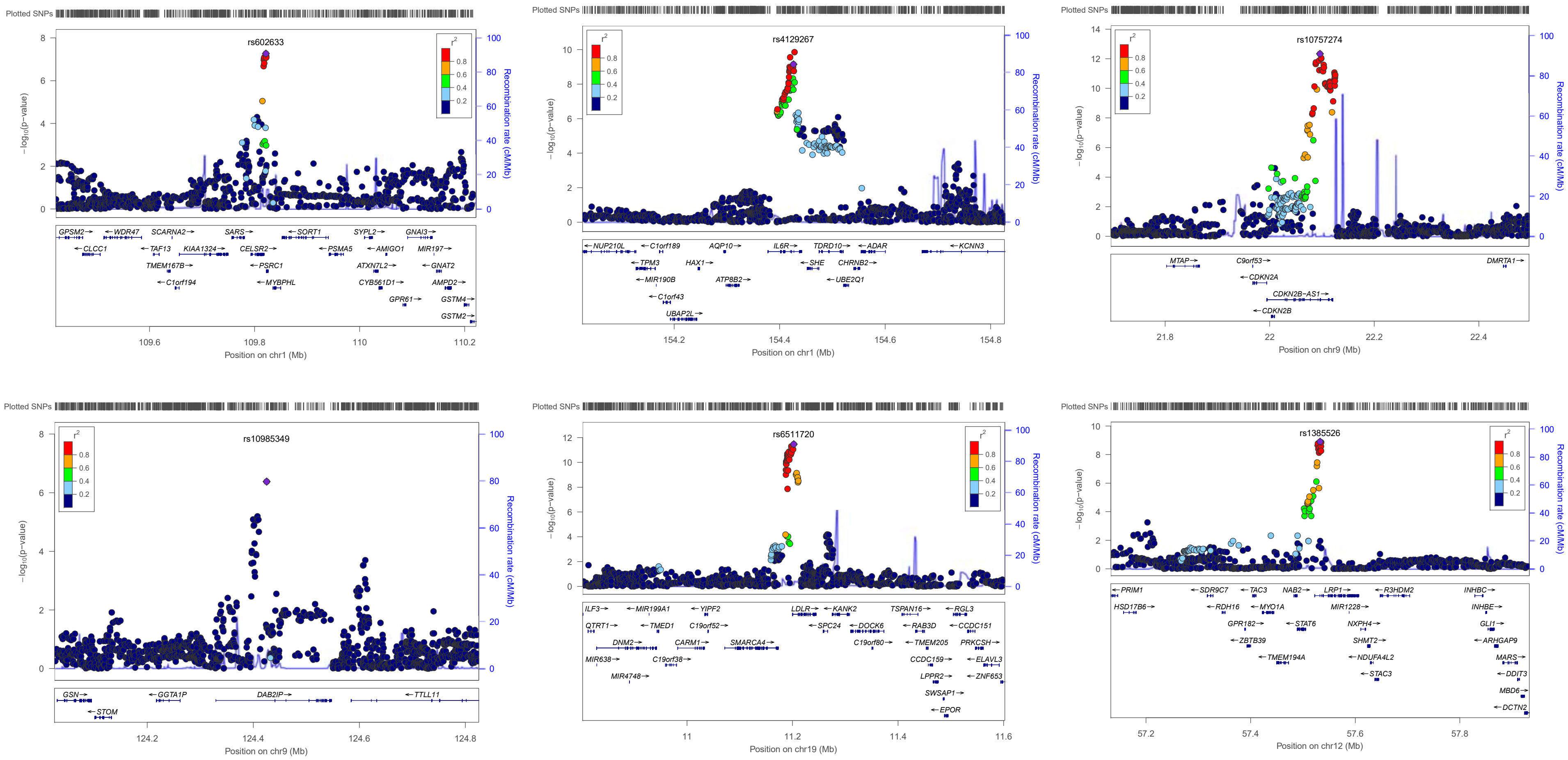

Five of the 6 previously identified AAA loci at 1p13.3 (*SORT1*), 1q21.3 (*IL6R*), 9p21 (*CDKN2BAS1/ANRIL*), 9q33 (*DAB2IP)* and 19p13.2 (*LDLR*) were replicated in this meta-GWAS and validation analysis. Although the previously reported[1] 12q13 (*LRP1*) locus (lower right panel) reached the discovery threshold (P=1.1x10^{-9}), it fell below the genome-wide threshold when combined with the validation cohorts (combined P=6.4x10^{-7}).

SNP LOOKUP IN GWAS FOR OTHER TRAITS ASSOCIATED WITH AAA

Data for AAA associated SNPs (those passing the genome-wide association threshold after combination of the results of the meta-analysis and validation studies) were obtained from GWAS datasets for other traits associated with AAA to determine if the associations were unique to AAA or related to generalized CVD (**Online Table IX and Figure 3)**. All results were from meta-analyses of multiple primary GWAS datasets for each trait. Summary results for each AAA associated SNP (P-value and effect size) were extracted. Results for type 2 diabetes[34] were obtained from the DIAGRAM consortium (http://www.diagram-consortium.org/index.html), CAD data from the CARDIoGRAM consortium[35] (www.CARDIOGRAMPLUSC4D.ORG), lipid trait data from the Global Lipids Genetics Consortium[36] (http://csg.sph.umich.edu/abecasis/public/lipids2013) and blood pressure data from the International Consortium for Blood Pressure[37] (http://www.ncbi.nlm.nih.gov/projects/gap/cgi-bin/study.cgi?study_id=phs000585.v1.p1).

Online Table IX: Results of lookup of AAA associated SNPs in GWAS of other cardiovascular traits (See also below). CAD: Coronary Artery Disease; HDL: High-density Lipoprotein; LDL: Low-density Lipoprotein; TG: Triglyceride; DBP: Diastolic Blood Pressure; SBP: Systolic Blood Pressure.

Known AAA risk loci

Trait	Chr	Position	SNP	Locus	Gene(s)	AAA risk allele	OR (95%CI)	n cases	n controls	P
Type 2 Diabetes	1	109821511	rs602633	1p13.3	CELSR2/*SORT1*	T	1.05 (1.01 - 1.1)	9580	53810	0.025
	1	154426264	rs4129267	1q21.3	*IL6R*	T	0.96 (0.93 - 1.00)	12171	56862	0.037
	9	22096055	rs10757274	9p21	*ANRIL*	A	0.96 (0.94 - 1.00)	12171	56862	0.041
	9	124425243	rs10985349	9q33.2	*DAB2IP*	T	1.03 (0.98 - 1.07)	12171	56862	0.210
	19	11202306	rs6511720	19p13.2	*LDLR*	T	1.02 (0.96 - 1.10)	8558	52735	0.480

Trait	Chr	Position	SNP	Locus	Gene(s)	AAA risk allele	OR	n cases	n controls	P
CAD	1	109821511	rs602633	1p13.3	CELSR2/*SORT1*	T	0.90 (0.87 - 0.93)	20375	61324	2.16x10^{-9}
	1	154426264	rs4129267	1q21.3	*IL6R*	T	0.95 (0.93 - 0.98)	20784	58718	0.001
	9	22096055	rs10757274	9p21	*ANRIL*	A	0.78 (0.74 - 0.82)	21932	62260	1.44x10^{-22}
	9	124425243	rs10985349	9q33.2	*DAB2IP*	T	1.04 (1.00 - 1.09)	14133	36016	0.036
	19	11202306	rs6511720	19p13.2	*LDLR*	T	0.88 (0.83 - 0.94)	8948	47471	1.61x10^{-04}

Trait	Chr	Position	SNP	Locus	Gene(s)	AAA risk allele	HDL beta (SE)	HDL n	HDL P	LDL beta (SE)	LDL n	LDL P	TG beta (SE)	TG n	TG P
Lipid traits	1	109821511	rs602633	1p13.3	CELSR2/*SORT1*	T	0.0073 (0.0051)	94311	0.077	-0.0066 (0.0057)	89888	0.230	-0.0129 (0.005)	91013	0.032
	1	154426264	rs4129267	1q21.3	*IL6R*	T	0.0328 (0.0041)	185599	3.50x10^{-14}	-0.1591 (0.0044)	171593	1.50x10^{-261}	-0.0121 (0.004)	176361	0.003
	9	22096055	rs10757274	9p21	*ANRIL*	A	-0.0047 (0.0048)	92706	0.371	0.0036 (0.0051)	83064	0.524	0.0012 (0.0047)	86702	0.907
	9	124425243	rs10985349	9q33.2	*DAB2IP*	T	-0.006 (0.0068)	86409	0.435	0.0067 (0.0075)	82099	0.557	-0.0039 (0.0066)	83111	0.944
	19	11202306	rs6511720	19p13.2	*LDLR*	T	0.0249 (0.0057)	184617	6.32x10^{-05}	-0.2209 (0.0061)	170608	3.85x10^{-262}	-0.0084 (0.0056)	175280	0.104

Trait	Chr	Position	SNP	Locus	Gene(s)	AAA risk allele	DBP beta (SE)	DBP n	DBP P	SBP beta (SE)	SBP n	SBP P
Blood Pressure	1	109821511	rs602633	1p13.3	CELSR2/*SORT1*	T	0.0704 (0.0665)	66347	0.289	-0.0568 (0.1044)	66352	0.586
	1	154426264	rs4129267	1q21.3	*IL6R*	T	0.0257 (0.0748)	66774	0.731	0.0100 (0.1180)	66781	0.932
	9	22096055	rs10757274	9p21	*ANRIL*	A	NA	NA	NA	NA	NA	NA
	9	124425243	rs10985349	9q33.2	*DAB2IP*	T	-0.0020 (0.0807)	58126	0.980	0.1229 (0.1283)	58171	0.338
	19	11202306	rs6511720	19p13.2	*LDLR*	T	-0.0171 (0.1134)	48183	0.880	-0.0963 (0.1792)	48153	0.591

New AAA risk loci

Trait	Chr	Position	SNP	Locus	Gene(s)	AAA risk allele	OR (95%CI)	n cases	n controls	P
Type 2 Diabetes	1	214409280	rs1795061	1q32.3	near *SMYD2*	T	1.04 (1.00 - 1.08)	9580	53810	0.044
	13	22861921	rs9316871	13q12.11	*LINC00540*	G	1 (0.96 - 1.04)	11902	53152	0.940
	20	44586023	rs3827066	20q13.12	Near *MMP9/ZNF335*	T	1.00 (0.95 - 1.06)	9580	53810	0.890
	21	39819830	rs2836411	21q22.2	*ERG*	T	1.01 (0.97 - 1.04)	12171	56862	0.720

Trait	Chr	Position	SNP	Locus	Gene(s)	AAA risk allele	OR	n cases	n controls	P
CAD	1	214409280	rs1795061	1q32.3	near *SMYD2*	T	0.99 (0.96 - 1.02)	20441	61399	0.533
	13	22861921	rs9316871	13q12.11	*LINC00540*	G	1.00 (0.97 - 1.03)	21588	59365	0.974
	20	44586023	rs3827066	20q13.12	Near *MMP9/ZNF335*	T	1.07 (1.03 - 1.12)	19108	59177	5.48x10^{-04}
	21	39819830	rs2836411	21q22.2	*ERG*	T	1.02 (0.99 - 1.05)	21424	59122	0.205

Trait	Chr	Position	SNP	Locus	Gene(s)	AAA risk allele	HDL beta (SE)	HDL n	HDL P	LDL beta (SE)	LDL n	LDL P	TG beta (SE)	TG n	TG P
Lipid traits	1	214409280	rs1795061	1q32.3	near *SMYD2*	T	0.0075 (0.0054)	94311	0.119	0.0023 (0.0059)	89888	0.572	-0.0047 (0.0052)	91013	0.501
	13	22861921	rs9316871	13q12.11	*LINC00540*	G	-0.0013 (0.0058)	90317	0.883	0.006 (0.0063)	85936	0.417	0.0018 (0.0058)	86976	0.563
	20	44586023	rs3827066	20q13.12	Near *MMP9/ZNF335*	T	0.0208 (0.0048)	185539	2.96x10^{-05}	-0.0092 (0.0052)	171507	0.103	-0.0156 (0.0047)	176203	0.003
	21	39819830	rs2836411	21q22.2	*ERG*	T	-0.0047 (0.005)	92801	0.402	-0.0082 (0.0055)	88414	0.269	0.0054 (0.0049)	89466	0.566

Trait	Chr	Position	SNP	Locus	Gene(s)	AAA risk allele	DBP beta (SE)	DBP n	DBP P	SBP beta (SE)	SBP n	SBP P
Blood pressure	1	214409280	rs1795061	1q32.3	near *SMYD2*	T	0.0320 (0.0691)	63232	0.643	0.0047 (0.1084)	63243	0.965
	13	22861921	rs9316871	13q12.11	*LINC00540*	G	0.0429 (0.0734)	69617	0.559	-0.0201 (0.1163)	69623	0.863
	20	44586023	rs3827066	20q13.12	Near *MMP9/ZNF335*	T	-0.0397 (0.0878)	59823	0.651	-0.0276 (0.1380)	59806	0.842
	21	39819830	rs2836411	21q22.2	*ERG*	T	0.1536 (0.0650)	67634	0.018	0.0487 (0.1024)	67631	0.635

SEARCH FOR OTHER ASSOCIATED TRAITS AND DISEASES USING GWAS DATABASES

The Phenotype-Genotype Integrator[38] (http://www.ncbi.nlm.nih.gov/gap/phegeni#GenomeView) and the GWAS catalog (http://www.gwascentral.org/index) were searched for diseases and traits associated with the lead SNPs at the AAA loci. In addition, we searched NHLBI GRASP catalog (GRASP v2.0; http://grasp.nhlbi.nih.gov/Overview.aspx)[39, 40] to find any further associations. The results obtained using the Phenotype-Genotype Integrator are shown in **Online Table X**, the search results from the GWAS catalog are presented in **Online Table XI,** and those using GRASP in **Online Table XII.**

PheGenI includes results from the NHGRI/EBI catalog. GRASP (Genome-Wide Repository of Associations Between SNPs and Phenotypes) is the largest GWAS results database in terms of coverage. It includes all available genetic association results from papers, their supplements and web-based content meeting the following guidelines:

- All associations with P<0.05 from GWAS defined as >= 25,000 markers tested for 1 or more traits.
- Study exclusion criteria: CNV-only studies, replication/follow-up studies testing <25K markers, non-human only studies, article not in English, gene-environment or gene-gene GWAS where single SNP main effects are not given, linkage only studies, aCGH/LOH only studies, heterozygosity/homozygosity (genome-wide or long run) studies, studies only presenting gene-based or pathway-based results, simulation-only studies, studies which we judge as redundant with prior studies since they do not provide significant inclusion of new samples or exposure of new results (e.g., many methodological papers on the WTCCC and FHS GWAS).

Online Table X: Results from the GWAS database search using the tool called Phenotype-Genotype Integrator (http://www.ncbi.nlm.nih.gov/gap/phegeni#GenomeView). All lead SNPs at the AAA loci were used for the search, but only two of the SNPs (rs4129267 and rs6511720) had hits.

Chr	Location	SNP	Gene	Trait	Location in gene	P-Value	Source	PubMed ID
1	154426264	rs4129267	*IL6R*	Receptors, Interleukin-6	intron	$2.00x10^{-57}$	NHGRI	18464913
				C-Reactive Protein	intron	$2.00x10^{-48}$	NHGRI	21300955
				Asthma	intron	$2.00x10^{-08}$	NHGRI	21907864
				Maximal Midexpiratory Flow Rate	intron	$7.00x10^{-06}$	NHGRI	17903307
19	11202306	rs6511720	*LDLR*	Cholesterol, LDL	intron	$4.00x10^{-11}7$	NHGRI	20686565
				Cholesterol	intron	$7.00x10^{-97}$	NHGRI	20686565
				Cholesterol, LDL	intron	$2.00x10^{-51}$	NHGRI	18193044
				Cholesterol, LDL	intron	$2.00x10^{-26}$	NHGRI	19060906
				Cholesterol, LDL	intron	$4.00x10^{-26}$	NHGRI	18193043
				1-Alkyl-2-acetylglycerophosphocholine Esterase	intron	$3.00x10^{-11}$	NHGRI	22003152
				Cholesterol, LDL	intron	$5.00x10^{-11}$	NHGRI	21943158
				Atherosclerosis	intron	$1.00x10^{-07}$	NHGRI	21909108

Online Table XI: Results of the dbGAP SNP lookup using the GWAS Catalog available at http://www.gwascentral.org/index. The total number of results in the GWAS Catalog ("n results in dbGAP") and the number of associations with P<1x10^{-3} (column labelled "n P<1x10^{-3}") are shown. Details on the associations with P<1x10^{-3} for each SNP are described.

SNP	Chromosome	Position	Gene(s)	n results in dbGAP	n P<1x10^{-3}	P-value	Phenotype	Study
rs602633	1	109821511	*PSRC1-CELSR2-SORT1*	16	3	4.80x10^{-14}	LDL cholesterol levels	Meta-analysis of plasma lipid concentrations (HGVST214)
						5.70x10^{-14}	Serum LDL cholesterol levels	GWAS of LDL-cholesterol concentrations (HGVST227)
						0.0001862	Height	GWAS of height (HGVST634)
rs4129267	1	154426264	*IL6R*	77	6	2.00x10^{-57}	Protein quantitative trait loci	GWAS of protein quantitative trait loci (HGVST264)
						2.00x10^{-48}	C-reactive protein level	GWAS of C-reactive protein levels (HGVST728)
						2.00x10^{-08}	Asthma	Unspecified analysis (HGVRS1753)
						7.39x10^{-06}	Percent predicted forced expiratory flow	GWAS of pulmonary function phenotypes in the Framingham Heart Study (HGVST212)
						1.92x10^{-05}	Asthma; Total asthma sample fixed effects (HGVRS1509)	GWAS of asthma (HGVST631)
						0.00014411	Asthma; Total asthma sample random effects(HGVRS1257)	GWAS of asthma (HGVST631)
rs1795061	1	214409280	*SMYD2*	19	0			
rs10757274	9	22096055	*ANRIL*	6	2	8.00x10^{-45}	Coronary heart disease	GWAS of Coronary heart disease (HGVST1380)
						3.70x10^{-06}	Coronary heart disease	GWAS of Coronary heart disease (HGVST57)
rs10985349	9	124425243	*DAB2IP*	19	0			
rs9316871	13	22861921	*LINC00540*	101	2	0.0007248	Schizophrenia	GWAS of schizophrenia (HGVST903)
						0.00084	Crohn's disease	GWAS of Crohn's disease (HGVST680)
rs6511720	19	11202306	*LDLR*	57	14	2x10^{-51}	LDL cholesterol	Meta-analysis of lipid concentrations (HGVST203)
						4.2x10^{-26}	LDL cholesterol levels	Meta-analysis of plasma lipid concentrations (HGVST214)
						2x10^{-26}	LDL cholesterol	GWAS of HDL cholesterol, triglycerides and LDL cholesterol (HGVST235)
						0.00026672	Serum cholesterol	GWAS of serum cholesterol levels in a British population (HGVST312)
						0.0005127	Height	GWAS of height (HGVST634)
						0.0000001	Carotid intima media thickness, plaque	GWAS of carotid intima media thickness (HGVST923)
						3x10^{-11}	Lipoprotein-associated phospholipase A2 activity and mass (Activity concentrations)	GWAS of lipoprotein-associated phospholipase A2 activity and mass (HGVST931)
						5x10^{-11}	Cardiovascular disease risk factors (LDL)	GWAS of cardiovascular disease risk factors (HGVST956)
						0.000000004	Metabolite levels	GWAS of Metabolite levels (HGVST1409)
						2x10^{-31}	Lipid metabolism phenotypes (LDL-C.assay, whole)	GWAS of Lipid metabolism phenotypes (HGVST1667)
						3x10^{-18}	Lipid metabolism phenotypes (APOB.assay, fasting)	GWAS of Lipid metabolism phenotypes (HGVST1667)
						1x10^{-25}	Lipid metabolism phenotypes (LDL-C.assay, fasting)	GWAS of Lipid metabolism phenotypes (HGVST1667)
						5x10^{-25}	Lipid metabolism phenotypes (APOB.assay, whole)	GWAS of Lipid metabolism phenotypes (HGVST1667)
rs3827066	20	44586023	*PCIF1-ZNF335-MMP9*	15	0			
rs2836411	21	39819830	*ERG*	94	1	0.0009289	Height	GWAS of height (HGVST634)

Online Table XII: Previously reported associations of the lead SNPs from AAA loci identified from an analysis of GRASP v2.0 (http://grasp.nhlbi.nih.gov/Overview.aspx). The Phenotypes, P values and sample sizes are those reported in the original publication that is referenced under 'Phenotype' in the table. The SNP, chromosome, position and genes are those from this analysis that were entered into GRASP v2.0 as a query. This table spans 3 pages.

SNP	CHR	Position	Gene	Phenotype	P value	Ancestry	Total	Total Replication	Total
rs602633	1	109821511	*PSRC1-CELSR2-SORT1*	LDL cholesterol[41]	$4.80x10^{-14}$	European	8656	11399	20055
				LDL cholesterol in serum[42]	$5.70x10^{-14}$	European	11685	5036	16721
				LDL cholesterol[43]	$7.60x10^{-41}$	European	19840	20623	40463
				LDL cholesterol[44]	$3.10x10^{-08}$	European	5059	0	5059
				APOB (apolipoprotein B)[44]	$2.20x10^{-07}$	European	5059	0	5059
				Coronary artery disease (CAD)[45]	$9.00x10^{-08}$	Unspecified	8319	10707	19026
				LDL cholesterol change with statins[46]	$8.40x10^{-08}$	European	3928	0	3928
				LDL cholesterol[46]	$8.40x10^{-08}$	European	3928	0	3928
				Total cholesterol change with statins[46]	$5.50x10^{-06}$	European	3928	0	3928
				Total cholesterol[46]	$5.50x10^{-06}$	European	3928	0	3928
				LDL cholesterol[47]	$4.90x10^{-16}4$	Mixed	100184	39875	140059
				Total cholesterol[47]	$3.90x10^{-12}7$	Mixed	100184	39875	140059
				HDL cholesterol[47]	$5.20x10^{-07}$	Mixed	100184	39875	140059
				Height[48]	$1.90x10^{-04}$	European	133653	50074	183727
				LDL cholesterol[49]	$2.90x10^{-06}$	African	8090	8849	16939
				LDL cholesterol[50]	$1.20x10^{-22}$	European	11683	0	11683
				LDL cholesterol (baseline)[51]	$5.00x10^{-08}$	European	5244	0	5244
				Lp-PLA2 activity[52]	$1.40x10^{-16}$	European	13664	0	13664
				Total cholesterol[53]	$5.90x10^{-65}$	European	66240	25282	91522
				LDL cholesterol[53]	$6.90x10^{-65}$	European	66240	25282	91522
				HDL cholesterol[53]	$1.30x10^{-07}$	European	66240	25282	91522
				Coronary artery disease (CAD)[54]	$4.70x10^{-26}$	Mixed	194427	15613	210040
				Coronary artery disease (CAD) age <=50[54]	$2.80x10^{-20}$	Mixed	194427	15613	210040
				Coronary artery disease (CAD) (males)[54]	$1.30x10^{-18}$	Mixed	194427	15613	210040
				Coronary artery disease (CAD) with myocardial infarction (MI)[54]	$2.20x10^{-16}$	Mixed	194427	15613	210040
				Coronary artery disease (CAD) age >50[54]	$5.00x10^{-08}$	Mixed	194427	15613	210040
				Coronary artery disease (CAD) (females) [54]	$3.40x10^{-05}$	Mixed	194427	15613	210040
rs4129267	1	154426264	*IL6R*	Lung function, predicted forced expiratory flow (FEF)[55]	$7.40x10^{-06}$	European	1222	0	1222
				Plasma C-reactive protein (female)[56]	$2.00x10^{-08}$	European	6345	0	6345
				Soluble IL6R (sIL6R)[57]	$2.50x10^{-76}$	European	1200	0	1200
				C-reactive protein [log (mg/l)][58]	$4.40x10^{-04}$	European	4763	0	4763
				Plasma fibrinogen (females)[59]	$1.80x10^{-11}$	European	17686	0	17686
				Asthma[60]	$1.90x10^{-05}$	European	26475	0	26475
				Fibrinogen[61]	$8.40x10^{-07}$	Mixed	30291	0	30291
				C-reactive protein (CRP)[62]	$2.10x10^{-48}$	European	66185	16540	82725
				Asthma[60]	$2.40x10^{-08}$	European	7197	57800	64997
				Interleukin-6 (IL-6) levels[63]	$2.40x10^{-08}$	European	4694	1392	6086
				C-reactive protein (CRP)[64]	$1.80x10^{-05}$	Mixed	11828	11991	23819
				Coronary artery disease (CAD)[54]	$1.70x10^{-08}$	Mixed	194427	15613	210040
				Interleukin-6 (IL-6) levels[65]	$1.60x10^{-21}$	European	8356	0	8356
				C-reactive protein (CRP)[65]	$8.80x10^{-12}$	European	8356	0	8356
rs1795061	1	214409280	*SMYD2*	None					
rs10757274	9	22096055	*ANRIL*	Coronary heart disease (CHD)[66]	$3.70x10^{-06}$	Mixed	634	28047	28681
				Coronary artery disease (CAD)[45]	$7.00x10^{-11}$	Unspecified	8319	10707	19026
				Coronary artery disease (CAD)[67]	$7.60x10^{-45}$	Asian	6534	26932	33466
				Coronary artery calcification (CAC)[68]	$8.00x10^{-10}$	European	4518	0	4518

SNP	CHR	Position	Gene	Phenotype	P value	Ancestry	Total	Total Replication	Total
				Coronary artery calcification (CAC)[69]	6.80×10^{-08}	Mixed	1509	3344	4853
rs10985349	9	124425243	*DAB2IP*	None					
rs9316871	13	22861921	*LINC00540*	Body mass index (BMI)[70]	6.90×10^{-04}	European	5217	0	5217
				Schizophrenia[71]	9.40×10^{-05}	European	16161	31375	47536
				HDL cholesterol change with statins[46]	7.70×10^{-04}	European	3928	0	3928
rs6511720	19	11202306	*LDLR*	LDL cholesterol[41]	4.20×10^{-26}	European	8656	11399	20055
				LDL cholesterol[72]	2.00×10^{-51}	Mixed	2758	22803	25561
				LDL cholesterol[43]	5.20×10^{-30}	European	19840	20623	40463
				LDL cholesterol exam 1 values[43]	3.00×10^{-04}	European	19840	20623	40463
				Total cholesterol (exam 1)[43]	3.40×10^{-04}	European	19840	20623	40463
				LDL cholesterol (mmol/l) [58]	1.50×10^{-09}	European	4763	0	4763
				Total cholesterol[73]	6.80×10^{-18}	European	22562	0	22562
				APOB (apolipoprotein B)[74]	7.00×10^{-18}	European	6382	970	7352
				LDL cholesterol[74]	5.20×10^{-15}	European	6382	970	7352
				LDL cholesterol[44]	7.30×10^{-24}	European	5059	0	5059
				APOB (apolipoprotein B) [44]	6.80×10^{-15}	European	5059	0	5059
				LDL cholesterol lipoprotein fraction concentration[75]	2.30×10^{-31}	European/Unspecified	17296	9472	26768
				LDL cholesterol lipoprotein fraction concentration in fasting sample[75]	1.50×10^{-25}	European/Unspecified	17296	9472	26768
				APOB assay lipoprotein fraction concentration[75]	4.80×10^{-25}	European/Unspecified	17296	9472	26768
				APOB assay lipoprotein fraction concentration in fasting sample[75]	2.80×10^{-18}	European/Unspecified	17296	9472	26768
				LDL cholesterol large lipoprotein fraction concentration[75]	4.30×10^{-15}	European/Unspecified	17296	9472	26768
				LDL cholesterol total lipoprotein fraction concentration[75]	1.90×10^{-13}	European/Unspecified	17296	9472	26768
				LDL cholesterol large lipoprotein fraction concentration in fasting sample[75]	3.00×10^{-12}	European/Unspecified	17296	9472	26768
				VLDL cholesterol small lipoprotein fraction concentration[75]	1.60×10^{-10}	European/Unspecified	17296	9472	26768
				LDL cholesterol total lipoprotein fraction concentration in fasting sample[75]	2.10×10^{-09}	European/Unspecified	17296	9472	26768
				VLDL cholesterol small lipoprotein fraction concentration in fasting sample[75]	1.90×10^{-08}	European/Unspecified	17296	9472	26768
				LDL cholesterol change with statins[46]	2.80×10^{-05}	European	3928	0	3928
				LDL cholesterol[46]	2.80×10^{-05}	European	3928	0	3928
				LDL cholesterol[47]	8.60×10^{-12}2	Mixed	100184	39875	140059
				Total cholesterol[47]	1.70×10^{-10}1	Mixed	100184	39875	140059
				Coronary artery disease (CAD) [47]	5.00×10^{-09}	Mixed	100184	39875	140059
				Height[48]	5.10×10^{-04}	European	133653	50074	183727
				LDL cholesterol[49]	2.00×10^{-51}	African	8090	8849	16939
				Presence of carotid artery plaque[76]	8.20×10^{-08}	European	31211	11273	42484
				Carotid artery plaque[76]	1.00×10^{-07}	European	31211	11273	42484
				Coronary artery disease (CAD) [76]	2.00×10^{-04}	European	31211	11273	42484
				LDL cholesterol[50]	5.00×10^{-11}	European	11683	0	11683
				Coronary artery disease (CAD)[77]	1.10×10^{-08}	Mixed	50587	57594	108181
				LDL cholesterol (baseline) [51]	5.20×10^{-15}	European	5244	0	5244
				Lp-PLA2 activity[52]	2.60×10^{-11}	European	13664	0	13664
				Lp-PLA2 mass[52]	5.50×10^{-05}	European	13664	0	13664
				Metabolic syndrome domains (Atherogenic Dyslipidemia - PC1)[78]	5.20×10^{-30}	Mixed	25755	0	25755
				Metabolic syndrome domains (Multivariate analysis) [78]	8.30×10^{-28}	Mixed	25755	0	25755
				LDL cholesterol[79]	1.40×10^{-49}	Mixed	44957	0	44957
				LDL cholesterol (female)[79]	7.50×10^{-29}	Mixed	44957	0	44957
				LDL cholesterol (male)[79]	3.10×10^{-25}	Mixed	44957	0	44957
				LDL cholesterol[53]	1.30×10^{-79}	European	66240	25282	91522
				Total cholesterol[53]	1.70×10^{-71}	European	66240	25282	91522
				APOB (apolipoprotein B)[80]	4.10×10^{-27}	European	3895	14810	18705
				LDL cholesterol[80]	3.80×10^{-25}	European	3895	14810	18705
				APOB (apolipoprotein B) response after 40mg daily simvastatin treatment[80]	1.40×10^{-07}	European	3895	14810	18705
				LDL cholesterol response after 40mg daily simvastatin treatment[80]	4.30×10^{-07}	European	3895	14810	18705
				LDL cholesterol[81]	2.60×10^{-17}	Mixed	9813	7000	16813
				Total cholesterol[81]	1.80×10^{-16}	Mixed	9813	7000	16813

SNP	CHR	Position	Gene	Phenotype	P value	Ancestry	Total	Total Replication	Total
				Total cholesterol[82]	$4.40x10^{-04}$	Hispanic	2240	2121	4361
rs3827066	20	44586023	*PCIF1-ZNF335-MMP9*	Coronary artery disease (CAD) [54]	$1.40x10^{-05}$	Mixed	194427	15613	210040
rs2836411	21	39819830	*ERG*	None					

PheWAS ANALYSIS

We performed a phenome-wide association study (PheWAS)[83, 84] exploring the association between the 9 AAA-associated SNPs and an extensive group of diagnoses to identify novel associations and uncover potential pleiotropy. For the PheWAS we used data from the electronic Medical Records and Genomics (eMERGE) Network[23] derived from 7 adult sites with a total of 27,077 unrelated patients of European ancestry above 19 years of age. We divided these samples into two datasets by proportional sampling based on eMERGE site, sex, and genotyping platform (13,559 and 13,518 individuals in sets 1 and 2, respectively). We calculated associations between the 9 AAA-associated SNPs and case or control status based on the extensive set of ICD-9 diagnoses, where for a specific diagnosis, individuals with the diagnosis are considered cases. Associations were adjusted for sex, site, genotyping platform and the first 3 principal components to account for global ancestry. We considered the identification of previously known associations, such as rs602633 associated with hyperglyceridemia and rs10757274 associated with CAD, to be indications that the PheWAS approach was robust. The PheWAS results are presented in **Online Table XIII**.

Online Table XIII: PheWAS Results

							PheWAS dataset 1			PheWAS dataset 2		
Chr	Position	SNP	Locus	Gene(s)	PheWAS associations	ICD-9 Description	n cases/controls	Beta(SE)	P	n cases/controls	Beta(SE)	P
1	214409280	rs1795061	1q32.3	near *SMYD2*	None							
1	154426264	rs4129267	1q21.3	*IL6R*	None							
1	109821511	rs602633	1p13.3	CELSR2/*SORT1*	6	Other and unspecified hyperlipidemia	5467/5722	-0.187 (0.035)	$7.86171x10^{-08}$	5539/5645	-0.151 (0.035)	$1.54x10^{-05}$
						Other and unspecified hyperlipidemia	5467/5722	-0.187 (0.0345)	$7.86171x10^{-08}$	2506/8687	-0.114 (0.042)	0.006402
						Pure hypercholesterolemia	2436/8780	-0.116 (0.042)	0.0055185	5539/5645	-0.151 (0.035)	$1.54x10^{-05}$
						Pure hypercholesterolemia	2436/8780	-0.116 (0.042)	0.0055185	2506/8687	-0.114 (0.042)	0.006402
						Mixed hyperlipidemia	1310/11032	-0.141 (0.054)	0.0083104	5539/5645	-0.151 (0.035)	$1.54x10^{-05}$
						Mixed hyperlipidemia	1310/11032	-0.141 (0.054)	0.0083104	2506/8687	-0.114 (0.042)	0.006402
9	22096055	rs10757274	9p21	*ANRIL*	2	Coronary atherosclerosis of native coronary artery	2332/9886	0.210 (0.034)	$3.70081x10^{-10}$	2141/9972	0.158 (0.035)	$6.9x10^{-06}$
						Coronary atherosclerosis of unspecified type of vessel, native or graft	2167/10004	0.202 (0.035)	$4.61186x10^{-09}$	2141/9972	0.158 (0.035)	$6.9x10^{-06}$
9	124425243	rs10985349	9q33.2	*DAB2IP*	None							
13	22861921	rs9316871	13q12.11	*LINC00540*	None							
19	11202306	rs6511720	19p13.2	*LDLR*	4	Other and unspecified hyperlipidemia	5638/5878	-0.201 (0.046)	$1.05931x10^{-05}$	2583/8928	-0.218 (0.055)	$4.95x10^{-05}$
						Other and unspecified hyperlipidemia	5638/5878	-0.201 (0.046)	$1.05931x10^{-05}$	5697/5803	-0.173 (0.045)	$9.53x10^{-05}$
						Pure hypercholesterolemia	2502/9017	-0.183 (0.056)	0.000948352	2583/8928	-0.218 (0.055)	$4.95x10^{-05}$
						Pure hypercholesterolemia	2502/9017	-0.183 (0.056)	0.000948352	5697/5803	-0.173 (0.045)	$9.53x10^{-05}$
20	44586023	rs3827066	20q13.12	Near PCIF1/*MMP9*/*ZNF335*	None							
21	39819830	rs2836411	21q22.2	*ERG*	None							

ANNOTATION OF AAA ASSOCIATED SNPs USING THE UCSC GENOME BROWSER

The 9 AAA-associated loci were manually annotated using the UCSC Genome Browser (http://genome.ucsc.edu/cgi-bin/hgGateway) on the hg19 human genome assembly. To annotate a gene, the SNP identification number (rs ID) was typed into the browser, and the genomic region centered on the SNP was examined. We noted genomic elements within 10 kbp of the SNP and on either side of the SNP. Within the browser, there were eleven main tracks that were used to annotate the SNP, which displayed gene locations, related literature, full-length public transcriptome data (mRNAs and ESTs), regulation, conservation, and repetitive elements. The results of this annotation are presented in **Online Table XIV**.

Gene Location

We used several UCSC Genome Browser tracks to determine whether a locus was exonic, intronic, or intergenic, as well as the identity and classification of the gene, if any, at the locus. One of the tracks used was the UCSC Known Genes track. This track displays information on genes and their location, including both protein-coding and non-coding RNA genes[85]. Within this track, NCBI Reference Sequence (RefSeq) genes and GenBank genes were aligned to the genome (by the UCSC Genome Bioinformatics team) using the BLAST-like alignment tool (BLAT)[86-88]. In order to be included, genes needed a 98% alignment. The track also included gene models from the Consensus CDS (CCDS) project. Predicted genes from tRNA and mouse genes from Rfam with synteny to the human genome are also included in the track[89, 90]. The track also reports whether the gene is coding or non-coding. In addition to using the NCBI RefSeq to search proteins, UniProt proteins are also reported[91].

Another UCSC track that describes the location of coding and non-coding genes is the NCBI RefSeq track. This displays genes that were aligned to genome with at least a 96% match[88]. We also used ENCODE Consortium's Gencode human gene catalog (v19)[92]. The track combines automatic annotations with manual and experimentally validated entries. Another track used to examine gene location is the Broad Institute lincRNA track. The long intergenic non-coding RNA (lincRNA) data were collected by RNA sequencing (RNA-seq)[93]. In addition to lincRNAs, the track also displays transcripts of uncertain coding potential (TUCP). For each of these gene types, expression was displayed across 22 different cell and tissue types[94].

Related Literature

The loci were also annotated according to their relationship to other SNPs. The National Human Genome Research Institute (NHGRI) has a UCSC track of manually curated loci from published Genome-Wide Association Studies (GWAS)[95]with $P < 1.0 \times 10^{-5}$. This track was used to check independent previously reported disease or phenotype association of each SNP, and to see if the locus of interest fell within a “SNP cloud”, which is an area with several SNPs all significantly associated with complementary or biologically similar quantitative traits.

mRNAs and ESTs

In addition to looking at genes, mRNAs and expressed sequence tags (ESTs) were examined. One track used was the human mRNA track. This is comprised of human mRNAs from GenBank aligned to the genome using BLAT[86, 87]. The Human ESTs track was compiled in the same manner, and included both spliced and un-spliced ESTs. Both of these tracks contain raw full-length public transcriptome data

captured through transcript-to-genome alignments and were used to confirm the presence of genes, to interrogate gene expression profiles, to derive comprehensive information on gene structures (promoters, splice junctions, 3'ends), and novel transcriptional units absent from gene databases.

Regulation

There were also several UCSC Browser tracks used to examine epigenetic and post-transcriptional regulation in the vicinity of a SNP. One track is the TS miRNA track. This shows 3' untranslated region (UTR) microRNA (miRNA) binding sites predicted using TargetScanHuman version 5.1. First, the UTRs were scanned for miRNA sites[96]. After all the matches were found, they were ranked[97]. Another track that displayed regulatory information was the ENCODE Regulation supertrack, which consists of 7 sub-tracks. All of the tracks were used in annotating the locus. The first of these sub-tracks is the transcription track, which displays ENCODE RNA-seq results from cells representing 9 different tissues[98].

Three of the sub-tracks show information on histone modifications. This was collected by ENCODE using chromatin immunoprecipitation sequencing (ChIP-seq) on cells representing seven different tissues[99]. One histone modification is the monomethylation of lysine 4 in the histone 3 protein, referred to as H3K4Me1. Another modification is the acetylation of lysine 27 in the same histone protein, referred to as H3K27Ac. The presence of either modification suggests an activating regulatory element, and the co-occurrence of the two modifications indicates a putative enhancer region. IThe third histone modification examined is a trimethylation of lysine 4 in the same histone protein, referred to as H3K4Me3. This signal indicates the presence of a promoter.

Another sub-track we used is the DNase hypersensitivity track version 3, which shows areas of open chromosome accessibility in 125 different cell lines[100]. The final two sub-tracks display transcription factor binding sites (TFBSs). Both were created using ChIP-Seq and have information for 161 different transcription factors in 91 different cell types[101]. The differentiating factor between the two tracks is that one includes information from Factorbook, which displays consensus motifs in binding sites[102].

Conservation

To examine the evolutionary conservation of a locus, the PhyloP Conservation track is used. This displays conservation across 100 different species in a human-centric multispecies alignment[103].

Repetitive Elements

The final track used is RepeatMaster, which searches the genome for 10 different forms of repeating elements, including long interspersed nuclear elements (LINE), short interspersed nuclear element (SINE), and retrotransposons. This track uses information from the Genetic Information Research Institute's (GIRI) Repbase Update library[104] and makes it possible to determine whether a SNP resides within a genomic repetitive element.

Online Table XIV. Annotation of AAA-Associated SNPs using the UCSC Genome Browser (http://genome.ucsc.edu/cgi-bin/hgGateway)

**SNP rs#**	**Information Available in UCSC Genome Browser**
rs1795061	Intron of one mRNA (AY343912), but no ESTs<br>~550 bp downstream of CEBP beta binding site with consensus motif
rs4129267	In a LINE repeat<br>NHGRI: associated with CAD, asthma, C-protein levels, and protein quantitative traits.<br>Intron of *IL6R* (involved in immune responses), H3K4Me1 expression<br>In a DNase hypersensitivity cluster (83/125) and 7 TFBSs, including one with a consensus site (MYC).<br>Low expression level.<br>Two ESTs are near the hypersensitivity site, but are not related to any gene/mRNAs.
rs602633	NHGRI: associated with stroke<br>850 bp downstream of 3' end of *PSRC1* (involved in mitosis)<br>900 bp downstream of large H3K4Me1 peak; high transcription levels<br>Associated with a DNase site (9/125) and 3 TFBSs, including a consensus site in *EGR1*.<br>Approximately 1 kb upstream of a DNase hypersensitivity region (125/125, 100% of cell types) with a large H3K4Me1 peak and mild H3K27Ac levels. There are 36 TFBSs, 6 with consensus motifs.<br>3 kb downstream of *CELSR2* (brain expressed cadherin like protein). This region also has high H3K4Me1 expression and moderate H3K27Ac levels, indicating an enhancer region. There are also high transcription levels. There is a DNase hypersensitivity region (62/125) that correlates with 50 TFBSs, 18 with consensus motifs.<br>5 kbp downstream of another DNase hypersensitivity site (20/125) with 6 TFBSs, 4 of which have consensus motifs.<br>SNP cloud with 9 other SNPs, which have been associated with cholesterol and lipid levels, as well as stroke, and CAD
rs10757274	NHGRI: associated with CAD<br>In a LINE and intron of *ANRIL*<br>1.6 kb upstream from high H3K4Me1 and moderate H3K27Ac<br>DNase hypersensitivity site (61) and 15 TFBSs (2 consensus motifs)<br>7 kbp upstream from a putative enhancer region (high H3K4Me1 and H3K27Ac), associated with DNase hypersensitivity (88) and over 50 TFBSs
rs10985349	Intron of *DAB2IP*<br>In a DNase hypersensitivity cluster (22) and H3K4Me1 peak
rs9316871	Intergenic
rs6511720	NHGRI: associated with CAD and aneurysm, as well as with lipid and cholesterol levels<br>Intronic to *LDLR*<br>Moderate transcription levels<br>High levels of H3K4Me1, H3K4Me3, and H3K27Ac (indicating an enhancer/promoter region)<br>In a DNase hypersensitivity cluster of 105/125 with approximately 40 TFBSs (9 consensus motifs)
rs3827066	Intron of *ZNF335*<br>Moderate transcription levels
rs2836411	Intron of *ERG*<br>High H3K4Me1 levels, mild H3K27Ac levels<br>Inside a DNase hypersensitivity cluster (11)

PUPASUITE ANALYSIS

The lead SNPs at the 4 novel AAA risk loci (**Table 1**) were identified in the 1000 Genomes phase 3 CEU panel. SNPs in LD (r^2>0.5) and the lead SNPs were extracted from the 1000 Genomes data and entered into Pupasuite v3.1[105] (**Online Table XV**). No non-synonymous, transcript structure, transcript processing, transcription factor (TF) binding site (Transfac/Jaspar/Oreganno), miRNA sequence, miRNA target, splice site or other functional results were identified.

Online Table XV: Pupasuite 3.1 output for SNPs in LD (r^2 > 0.5) with lead SNPs at novel AAA loci.
*Transcript IDs are shown without the full Ensembl Transcript ID (ENST number) for display purposes.
This table spans 2 pages.

Lead SNP	Chr	Position	Nearest gene(s)	LD SNPs	r^2	Location relative to transcript	Gene	Transcript(s)*
rs1795061	1	214409280	SMYD2	rs1795065	1	INTERGENIC		
				rs1660364	1	INTERGENIC		
				rs1660365	1	INTERGENIC		
				rs1795064	1	INTERGENIC		
				rs1795063	1	INTERGENIC		
				rs1795062	1	INTERGENIC		
				rs1660368	1	INTERGENIC		
				rs199679227	1	INTERGENIC		
				rs1660371	1	INTERGENIC		
				rs1795060	1	INTERGENIC		
				rs201675223	0.978	INTERGENIC		
				rs1147673	0.912	INTERGENIC		
				rs12745411	0.724	INTERGENIC		
				rs11585945	0.724	INTERGENIC		
				rs61819142	0.724	INTERGENIC		
				rs12754343	0.724	INTERGENIC		
				rs17784245	0.628	INTERGENIC		
				rs1021639	0.609	INTERGENIC		
rs9316871	13	22861921	LINC00540	rs9506822	0.85	INTERGENIC		
				rs9510086	0.763	INTERGENIC		
				rs12863716	0.763	INTERGENIC		
				rs7336555	0.763	INTERGENIC		
				rs12857403	0.763	INTERGENIC		
				rs12866004	0.763	INTERGENIC		
				rs11618858	0.763	INTERGENIC		
				rs7994761	0.763	INTERGENIC		
				rs9506820	0.696	INTERGENIC		
rs3827066	20	44586023	PCIF1-ZNF335-MMP9	rs73128528	0.922	INTRONIC	ZNF335	243961
						INTRONIC	ZNF335	322927
						INTRONIC	ZNF335	426788
				rs17448653	0.629	DOWNSTREAM	ZNF335	494955
						INTRONIC	ZNF335	243961
						INTRONIC	ZNF335	322927
						INTRONIC	ZNF335	426788
						UPSTREAM	ZNF335	475002
						WITHIN_NON_CODING_GENE	ZNF335	476822
rs2836411	21	39819830	ERG	rs2836399	0.555	INTRONIC	ERG	288319, 357391, 398897, 398899, 398905, 398907, 398910, 398911, 398916, 398919, 415743, 417133, 429727, 442448, 451178, 453032
						WITHIN_NON_CODING_GENE	ERG	468474, 473107, 481609, 492833
				rs2298336	0.53	INTRONIC	ERG	288319, 357391, 398897, 398899, 398905, 398907, 398910, 398911, 398916, 398919, 415743, 417133, 429727, 442448, 451178, 453032
						WITHIN_NON_CODING_GENE	ERG	468474, 473107, 481609, 492833
				rs2836402	0.53	INTRONIC	ERG	288319, 357391, 398897,

					398899, 398905, 398907, 398910, 398911, 398916, 398919, 415743, 417133, 429727, 442448, 451178, 453032
			WITHIN_NON_CODING_GENE	ERG	468474, 473107, 481609, 492833
	rs2836400	0.519	INTRONIC	ERG	288319, 357391, 398897, 398899, 398905, 398907, 398910, 398911, 398916, 398919, 415743, 417133, 429727, 442448, 451178, 453032
			WITHIN_NON_CODING_GENE	ERG	468474, 473107, 481609, 492833
	rs2836407	0.519	INTRONIC	ERG	288319, 357391, 398897, 398899, 398905, 398907, 398910, 398911, 398916, 398919, 415743, 417133, 429727, 442448, 451178, 453032
			UPSTREAM	ERG	492833
			WITHIN_NON_CODING_GENE	ERG	468474, 473107, 481609
	rs2836409	0.519	INTRONIC	ERG	288319, 398897, 398899, 398905, 398907, 398910, 398911, 398916, 398919, 417133, 442448, 451178, 453032
			UPSTREAM	ERG	357391, 415743, 429727, 492833
			WITHIN_NON_CODING_GENE	ERG	468474, 473107, 481609

GWAS3D ANALYSIS

The 9 AAA GWAS SNPs (LeadSNP) were entered into the GWAS3D[106] web-portal (http://jjwanglab.org/gwas3d), using the following settings: 1. SNP dataset: 1000 Genomes pilot 1, 2. Population: EUR, 3. LD threshold: R^2 >0.8, 4. Cell type: All; and 5. all ENCODE TF Family Motifs (binding site P-value 0.02).

The predicted lead functional SNP (Fn_SNPID) associations for the 9 AAA SNPs are shown in **Figure 4 and Online Table XVI**. For example, the AAA GWAS SNP rs602633 is in high LD with rs599839, which has previously been associated with AAA[3]. GWAS3D predicted the rs599839 variant to alter STAT, Ets, p300 and RFX5 binding affinities.

The extended list of potential functional variant associations within each locus is shown in **Online Table XVII**. All AAA SNPs were predicted to be associated with transcription factor binding site affinity variants and eight map to interactions with distal regions.

Online Table XVI: Lead functional associations for each of the 9 replicated AAA SNPs.

Fn_SNPID	Chr:Position	Locus	Combined P	LeadSNP	GWAS P	R^2	Status
rs4977575	9:22124744	9p21.3	$1.70x10^{-35}$	rs10757274	$1.5x10^{-33}$	0.87	
rs73128528	20:44582187	*ZNF335*	$3.72x10^{-19}$	rs3827066	$2.1x10^{-17}$	0.83	
rs73015013	19:11190873	19p13.2	$2.58x10^{-16}$	rs6511720	$7.9x10^{-14}$	0.94	
rs4845620	1:154406656	*IL6R*	$9.92x10^{-15}$	rs4129267	$4.8x10^{-13}$	0.87	
rs1660368	1:214407335	1q32.3	$4.64x10^{-14}$	rs1795061	$8.8x10^{-11}$	0.97	
rs599839	1:109822166	1p13.3	$3.07x10^{-13}$	rs602633	$6.6x10^{-09}$	0.92	
rs9510086	13:22862440	13q12.11	$8.33x10^{-12}$	rs9316871	$4.8x10^{-10}$	0.82	
rs10985349	9:124425243	*DAB2IP*	$7.46x10^{-11}$	rs10985349	$2.4x10^{-11}$	1	
rs2836411	21:39819830	*ERG*	$4.72x10^{-09}$	rs2836411	$5.8x10^{-09}$	1	

	Leading variant
	Significant TFBS
	Mapping on distal interaction
	Mapping on putative enhancer region
	Mapping on GERP++ conservation element

Online Table XVII: Significant regulatory variants detected by the GWAS3D algorithm. Status: distal interaction (td), transcription factor binding affinity (bda), chromatin modification state (chromhmm), sites under evolutionary constraint (gerp). This table spans 4 pages.

SNPID	CHRPOS	GENOTYPE	LOCUS	FINALP	LeadSNP	LEADSNP_P	RSQUARE	STATUS
rs4977575	9:22124744	C|G	9p21.3	$1.70x10^{-35}$	rs10757274	$1.54x10^{-33}$	0.87	td,bda,enhancer,gerp
rs1333049	9:22125503	G|C	9p21.3	$3.13x10^{-35}$	rs10757274	$1.54x10^{-33}$	0.88	td,bda,enhancer
rs1333046	9:22124123	T|A	9p21.3	$6.47x10^{-35}$	rs10757274	$1.54x10^{-33}$	0.93	td,bda,enhancer
rs10738610	9:22123766	A|C	9p21.3	$4.51x10^{-34}$	rs10757274	$1.54x10^{-33}$	0.93	td,bda,enhancer
rs7857118	9:22124140	A|T	9p21.3	$4.85x10^{-34}$	rs10757274	$1.54x10^{-33}$	0.92	td,bda,enhancer
rs7859362	9:22105927	T|C	*ANRIL*	$7.83x10^{-34}$	rs10757274	$1.54x10^{-33}$	0.93	td,bda,enhancer
rs10217586	9:22121349	A|T	9p21.3	$9.21x10^{-34}$	rs10757274	$1.54x10^{-33}$	0.81	td,bda,enhancer
rs7859727	9:22102165	C|T	*ANRIL*	$1.12x10^{-33}$	rs10757274	$1.54x10^{-33}$	0.97	td,bda,enhancer
rs10811656	9:22124472	C|T	9p21.3	$1.19x10^{-33}$	rs10757274	$1.54x10^{-33}$	0.85	td,bda,enhancer
rs1333043	9:22106731	T|A	*ANRIL*	$2.16x10^{-33}$	rs10757274	$1.54x10^{-33}$	0.93	td,bda,enhancer
rs2891168	9:22098619	A|G	*ANRIL*	$2.44x10^{-33}$	rs10757274	$1.54x10^{-33}$	0.99	td,bda,enhancer
rs10738608	9:22094796	A|C	*ANRIL*	$2.72x10^{-33}$	rs10757274	$1.54x10^{-33}$	0.95	td,bda,enhancer
rs10738607	9:22088094	A|G	*ANRIL*	$2.72x10^{-33}$	rs10757274	$1.54x10^{-33}$	0.95	td,bda,enhancer
rs6475609	9:22106271	A|G	*ANRIL*	$4.57x10^{-33}$	rs10757274	$1.54x10^{-33}$	0.93	td,bda,enhancer
rs2383207	9:22115959	A|G	*ANRIL*	$4.60x10^{-33}$	rs10757274	$1.54x10^{-33}$	0.90	td,bda,enhancer
rs1537370	9:22084310	C|T	*ANRIL*	$1.19x10^{-32}$	rs10757274	$1.54x10^{-33}$	0.84	td,bda,enhancer
rs10511701	9:22112599	T|C	*ANRIL*	$1.39x10^{-32}$	rs10757274	$1.54x10^{-33}$	0.90	td,bda,enhancer
rs1333047	9:22124504	A|T	9p21.3	$1.53x10^{-32}$	rs10757274	$1.54x10^{-33}$	0.87	td,bda,enhancer
rs10757275	9:22106225	G|A	*ANRIL*	$1.67x10^{-32}$	rs10757274	$1.54x10^{-33}$	0.94	td,bda,enhancer
rs1333048	9:22125347	A|C	9p21.3	$1.67x10^{-32}$	rs10757274	$1.54x10^{-33}$	0.94	td,bda,enhancer
rs4977574	9:22098574	A|G	*ANRIL*	$2.01x10^{-32}$	rs10757274	$1.54x10^{-33}$	0.99	td,bda,enhancer
rs10757278	9:22124477	A|G	9p21.3	$2.25x10^{-32}$	rs10757274	$1.54x10^{-33}$	0.88	td,bda,enhancer
rs1537374	9:22116046	A|G	*ANRIL*	$2.27x10^{-32}$	rs10757274	$1.54x10^{-33}$	0.90	td,bda,enhancer
rs1537375	9:22116071	T|C	*ANRIL*	$2.66x10^{-32}$	rs10757274	$1.54x10^{-33}$	0.91	td,bda,enhancer
rs7341791	9:22112427	A|G	*ANRIL*	$2.69x10^{-32}$	rs10757274	$1.54x10^{-33}$	0.89	td,bda,enhancer
rs10757279	9:22124630	A|G	9p21.3	$3.05x10^{-32}$	rs10757274	$1.54x10^{-33}$	0.88	td,bda,enhancer
rs2383206	9:22115026	A|G	*ANRIL*	$3.05x10^{-32}$	rs10757274	$1.54x10^{-33}$	0.90	td,bda,enhancer
rs7341786	9:22112241	A|C	*ANRIL*	$3.35x10^{-32}$	rs10757274	$1.54x10^{-33}$	0.89	td,bda,enhancer
rs10757272	9:22088260	C|T	*ANRIL*	$3.38x10^{-32}$	rs10757274	$1.54x10^{-33}$	0.96	td,bda,enhancer
rs1537376	9:22116220	T|C	*ANRIL*	$3.95x10^{-32}$	rs10757274	$1.54x10^{-33}$	0.90	td,bda,enhancer
rs1537373	9:22103341	T|G	*ANRIL*	$5.37x10^{-32}$	rs10757274	$1.54x10^{-33}$	0.96	td,bda,enhancer
rs10757274	9:22096055	A|G	*ANRIL*	$6.09x10^{-32}$	rs10757274	$1.54x10^{-33}$	1.00	td,bda,enhancer,self
rs2210538	9:22092257	G|A	*ANRIL*	$6.51x10^{-32}$	rs10757274	$1.54x10^{-33}$	0.85	td,bda,enhancer
rs1537371	9:22099568	C|A	*ANRIL*	$6.84x10^{-32}$	rs10757274	$1.54x10^{-33}$	0.96	td,bda,enhancer
rs10757277	9:22124450	A|G	9p21.3	$6.93x10^{-32}$	rs10757274	$1.54x10^{-33}$	0.88	td,bda,enhancer
rs10733376	9:22114469	G|C	*ANRIL*	$7.49x10^{-32}$	rs10757274	$1.54x10^{-33}$	0.90	td,bda,enhancer

SNPID	CHRPOS	GENOTYPE	LOCUS	FINALP	LeadSNP	LEADSNP_P	RSQUARE	STATUS
rs10738606	9:22088090	A|T	*ANRIL*	$9.11x10^{-32}$	rs10757274	$1.54x10^{-33}$	0.95	td,bda,enhancer
rs4977757	9:22094330	A|G	*ANRIL*	$9.23x10^{-32}$	rs10757274	$1.54x10^{-33}$	0.91	td,bda,enhancer
rs1004638	9:22115589	A|T	*ANRIL*	$9.85x10^{-32}$	rs10757274	$1.54x10^{-33}$	0.90	td,bda,enhancer
rs10738609	9:22114495	A|C,G,T	*ANRIL*	$1.05x10^{-31}$	rs10757274	$1.54x10^{-33}$	0.91	td,bda,enhancer
rs9644860	9:22090603	C|T	*ANRIL*	$1.08x10^{-31}$	rs10757274	$1.54x10^{-33}$	0.81	td,bda,enhancer
rs944797	9:22115286	T|C	*ANRIL*	$1.14x10^{-31}$	rs10757274	$1.54x10^{-33}$	0.90	td,bda,enhancer
rs1556516	9:22100176	G|C	*ANRIL*	$1.37x10^{-31}$	rs10757274	$1.54x10^{-33}$	0.96	td,bda,enhancer
rs10116277	9:22081397	G|T	*ANRIL*	$1.58x10^{-31}$	rs10757274	$1.54x10^{-33}$	0.85	td,bda,enhancer
rs1412834	9:22110131	T|C	*ANRIL*	$1.59x10^{-31}$	rs10757274	$1.54x10^{-33}$	0.91	td,bda,enhancer
rs1333042	9:22103813	A|G	*ANRIL*	$1.84x10^{-31}$	rs10757274	$1.54x10^{-33}$	0.95	td,bda,enhancer
rs6475606	9:22081850	C|T	*ANRIL*	$2.11x10^{-31}$	rs10757274	$1.54x10^{-33}$	0.85	td,bda,enhancer
rs1970112	9:22085598	T|C	*ANRIL*	$2.49x10^{-31}$	rs10757274	$1.54x10^{-33}$	0.86	td,bda,enhancer
rs73128528	20:44582187	A|T	*ZNF335*	$3.72x10^{-19}$	rs3827066	$2.10x10^{-17}$	0.83	bda,enhancer
rs7267295	20:44570683	C|T	*PCIF1*	$4.78x10^{-18}$	rs3827066	$2.10x10^{-17}$	0.84	td,bda,enhancer
rs58749629	20:44571317	G|A	*PCIF1*	$1.84x10^{-16}$	rs3827066	$2.10x10^{-17}$	0.91	td,bda,enhancer
rs73015013	19:11190873	C|T	19p13.2	$2.58x10^{-16}$	rs6511720	$7.90x10^{-14}$	0.94	td,bda,enhancer
rs8124182	20:44608901	G|A	20q13.12	$2.86x10^{-16}$	rs3827066	$2.10x10^{-17}$	0.84	td,bda,enhancer
rs7270354	20:44607661	G|A	20q13.12	$4.86x10^{-16}$	rs3827066	$2.10x10^{-17}$	0.91	td,bda,enhancer
chr19:11190074	19:11190074	G|A	19p13.2	$1.41x10^{-15}$	rs6511720	$7.90x10^{-14}$	0.96	td,bda,enhancer
chr19:11189272	19:11189272	T|C	19p13.2	$1.65x10^{-15}$	rs6511720	$7.90x10^{-14}$	0.94	td,bda,enhancer
chr19:11189937	19:11189937	T|A	19p13.2	$1.81x10^{-15}$	rs6511720	$7.90x10^{-14}$	0.96	td,bda,enhancer
rs6511720	19:11202306	G|T	*LDLR*	$2.06x10^{-15}$	rs6511720	$7.90x10^{-14}$	1	bda,enhancer,chromhmm,self
rs56289821	19:11188247	G|A	19p13.2	$3.84x10^{-15}$	rs6511720	$7.90x10^{-14}$	0.92	td,bda,enhancer
rs8106503	19:11196886	T|C	19p13.2	$6.27x10^{-15}$	rs6511720	$7.90x10^{-14}$	0.94	td,bda,enhancer,chromhmm
rs4845620	1:154406656	A|G	*IL6R*	$9.92x10^{-15}$	rs4129267	$4.76x10^{-13}$	0.87	td,bda,enhancer
rs17248720	19:11198187	C|T	*LDLR*	$1.87x10^{-14}$	rs6511720	$7.90x10^{-14}$	0.91	td,bda,enhancer,chromhmm
rs1660368	1:214407335	C|T	1q32.3	$4.64x10^{-14}$	rs1795061	$8.80x10^{-11}$	0.97	td,bda,enhancer,gerp
rs4537545	1:154418879	C|T	*IL6R*	$4.72x10^{-14}$	rs4129267	$4.76x10^{-13}$	0.87	td,bda,enhancer
chr19:11187358	19:11187358	T|G	19p13.2	$5.55x10^{-14}$	rs6511720	$7.90x10^{-14}$	0.92	td,bda,enhancer
rs56383622	1:154405024	A|G	*IL6R*	$5.67x10^{-14}$	rs4129267	$4.76x10^{-13}$	0.87	td,bda,enhancer
chr19:11191197	19:11191197	G|A	19p13.2	$7.26x10^{-14}$	rs6511720	$7.90x10^{-14}$	0.94	td,bda,enhancer
rs57217136	19:11201124	T|C	*LDLR*	$8.82x10^{-14}$	rs6511720	$7.90x10^{-14}$	1	bda,enhancer,chromhmm
rs4129267	1:154426264	C|T	*IL6R*	$9.12x10^{-14}$	rs4129267	$4.76x10^{-13}$	1	bda,enhancer,self
rs12151108	19:11197261	G|A	19p13.2	$1.10x10^{-13}$	rs6511720	$7.90x10^{-14}$	0.93	td,bda,enhancer
rs17248727	19:11198502	T|C	*LDLR*	$1.31x10^{-13}$	rs6511720	$7.90x10^{-14}$	0.92	td,bda,enhancer,chromhmm
rs2228145	1:154426970	A|C,T	*IL6R*	$1.39x10^{-13}$	rs4129267	$4.76x10^{-13}$	0.99	bda,enhancer
chr19:11189205	19:11189205	C|G	19p13.2	$1.54x10^{-13}$	rs6511720	$7.90x10^{-14}$	0.94	td,bda,enhancer
chr19:11189980	19:11189980	C|A	19p13.2	$1.73x10^{-13}$	rs6511720	$7.90x10^{-14}$	0.96	td,bda,enhancer
chr19:11188899	19:11188899	C|T	19p13.2	$1.74x10^{-13}$	rs6511720	$7.90x10^{-14}$	0.94	td,bda,enhancer
rs73015021	19:11192915	A|G	19p13.2	$1.91x10^{-13}$	rs6511720	$7.90x10^{-14}$	0.96	td,bda,enhancer

SNPID	CHRPOS	GENOTYPE	LOCUS	FINALP	LeadSNP	LEADSNP_P	RSQUARE	STATUS
chr19:11190544	19:11190544	C|T	19p13.2	$2.15x10^{-13}$	rs6511720	$7.90x10^{-14}$	0.88	td,bda,enhancer
rs6684439	1:154395839	C|T	*IL6R*	$2.51x10^{-13}$	rs4129267	$4.76x10^{-13}$	0.83	td,bda,enhancer
chr19:11191729	19:11191729	C|T	19p13.2	$2.95x10^{-13}$	rs6511720	$7.90x10^{-14}$	0.94	td,bda,enhancer
rs599839	1:109822166	G|A	1p13.3	$3.07x10^{-13}$	rs602633	$6.58x10^{-09}$	0.92	td,bda,enhancer,gerp
chr19:11190481	19:11190481	G|T	19p13.2	$3.15x10^{-13}$	rs6511720	$7.90x10^{-14}$	0.96	td,bda,enhancer
rs10412048	19:11193949	A|G	19p13.2	$3.58x10^{-13}$	rs6511720	$7.90x10^{-14}$	0.93	td,bda,enhancer
rs73015011	19:11189764	T|C	19p13.2	$3.78x10^{-13}$	rs6511720	$7.90x10^{-14}$	0.94	td,bda,enhancer
rs4845373	1:154417829	C|T	*IL6R*	$4.52x10^{-13}$	rs4129267	$4.76x10^{-13}$	0.87	td,bda,enhancer
rs55997232	19:11188117	C|T	19p13.2	$4.98x10^{-13}$	rs6511720	$7.90x10^{-14}$	0.94	td,bda,enhancer
rs56125973	19:11188164	T|C	19p13.2	$5.03x10^{-13}$	rs6511720	$7.90x10^{-14}$	0.91	td,bda,enhancer
chr19:11188850	19:11188850	T|C	19p13.2	$6.17x10^{-13}$	rs6511720	$7.90x10^{-14}$	0.93	td,bda,enhancer
rs12126142	1:154425456	G|A	*IL6R*	$6.17x10^{-13}$	rs4129267	$4.76x10^{-13}$	1	bda,enhancer
chr19:11187422	19:11187422	T|C	19p13.2	$6.25x10^{-13}$	rs6511720	$7.90x10^{-14}$	0.93	td,bda,enhancer
chr19:11190556	19:11190556	T|C	19p13.2	$6.99x10^{-13}$	rs6511720	$7.90x10^{-14}$	0.93	td,bda,enhancer
rs10402112	19:11191677	T|A	19p13.2	$7.26x10^{-13}$	rs6511720	$7.90x10^{-14}$	0.94	td,bda,enhancer
rs11265613	1:154418415	T|C	*IL6R*	$7.93x10^{-13}$	rs4129267	$4.76x10^{-13}$	0.88	td,bda,enhancer
chr19:11187324	19:11187324	C|G	19p13.2	$8.25x10^{-13}$	rs6511720	$7.90x10^{-14}$	0.93	td,bda,enhancer
rs55791371	19:11188153	A|C	19p13.2	$8.67x10^{-13}$	rs6511720	$7.90x10^{-14}$	0.93	td,bda,enhancer
rs1147673	1:214402313	A|G	1q32.3	$9.07x10^{-13}$	rs1795061	$8.80x10^{-11}$	0.93	td,bda,enhancer
rs61194703	19:11192193	A|T	19p13.2	$9.20x10^{-13}$	rs6511720	$7.90x10^{-14}$	0.94	td,bda,enhancer
chr19:11190292	19:11190292	T|C	19p13.2	$1.03x10^{-12}$	rs6511720	$7.90x10^{-14}$	0.94	td,bda,enhancer
rs4845622	1:154411419	A|C	*IL6R*	$1.13x10^{-12}$	rs4129267	$4.76x10^{-13}$	0.87	td,bda,enhancer
rs73015024	19:11197598	G|T	19p13.2	$1.13x10^{-12}$	rs6511720	$7.90x10^{-14}$	0.94	td,bda,enhancer
rs12133641	1:154428283	A|G	*IL6R*	$1.32x10^{-12}$	rs4129267	$4.76x10^{-13}$	0.97	bda,enhancer
chr19:11190534	19:11190534	G|A	19p13.2	$1.36x10^{-12}$	rs6511720	$7.90x10^{-14}$	0.93	td,bda,enhancer
chr19:11190110	19:11190110	A|G	19p13.2	$1.44x10^{-12}$	rs6511720	$7.90x10^{-14}$	0.96	td,bda,enhancer
chr19:11190549	19:11190549	G|A	19p13.2	$1.50x10^{-12}$	rs6511720	$7.90x10^{-14}$	0.93	td,bda,enhancer
rs73015016	19:11191300	G|A	19p13.2	$1.56x10^{-12}$	rs6511720	$7.90x10^{-14}$	0.94	td,bda,enhancer
rs12730935	1:154419892	G|A	*IL6R*	$1.60x10^{-12}$	rs4129267	$4.76x10^{-13}$	0.86	td,bda
chr19:11192831	19:11192831	A|G	19p13.2	$1.88x10^{-12}$	rs6511720	$7.90x10^{-14}$	0.94	td,bda,enhancer
rs73015020	19:11192550	G|A	19p13.2	$1.98x10^{-12}$	rs6511720	$7.90x10^{-14}$	0.93	td,bda,enhancer
rs4576655	1:154418749	C|T	*IL6R*	$2.56x10^{-12}$	rs4129267	$4.76x10^{-13}$	0.88	td,bda,enhancer
rs4845621	1:154409730	G|A	*IL6R*	$2.95x10^{-12}$	rs4129267	$4.76x10^{-13}$	0.87	td,bda
rs4393147	1:154414037	C|T	*IL6R*	$3.16x10^{-12}$	rs4129267	$4.76x10^{-13}$	0.87	td,bda,enhancer
rs12753254	1:154416935	G|A	*IL6R*	$3.49x10^{-12}$	rs4129267	$4.76x10^{-13}$	0.87	td,bda,enhancer
rs4845372	1:154415396	C|A	*IL6R*	$4.58x10^{-12}$	rs4129267	$4.76x10^{-13}$	0.83	td,bda,enhancer
rs6664201	1:154414296	C|T	*IL6R*	$5.08x10^{-12}$	rs4129267	$4.76x10^{-13}$	0.87	td,bda,enhancer
rs4845623	1:154415777	A|G	*IL6R*	$5.10x10^{-12}$	rs4129267	$4.76x10^{-13}$	0.83	td,bda,enhancer
rs7521458	1:154407713	T|C	*IL6R*	$5.55x10^{-12}$	rs4129267	$4.76x10^{-13}$	0.87	td,bda
rs12730036	1:154416969	C|T	*IL6R*	$6.86x10^{-12}$	rs4129267	$4.76x10^{-13}$	0.87	td,bda,enhancer

SNPID	CHRPOS	GENOTYPE	LOCUS	FINALP	LeadSNP	LEADSNP_P	RSQUARE	STATUS
rs9510086	13:22862440	G|C	13q12.11	$8.33x10^{-12}$	rs9316871	$4.80x10^{-10}$	0.81	td,bda,enhancer
rs7518199	1:154407419	A|C	*IL6R*	$8.49x10^{-12}$	rs4129267	$4.76x10^{-13}$	0.87	td,bda,enhancer
rs4453032	1:154414086	A|G	*IL6R*	$8.54x10^{-12}$	rs4129267	$4.76x10^{-13}$	0.87	td,bda,enhancer
rs1795065	1:214405194	G|A	1q32.3	$1.75x10^{-11}$	rs1795061	$8.80x10^{-11}$	0.97	td,bda,enhancer
rs1795060	1:214410021	C|T	1q32.3	$1.90x10^{-11}$	rs1795061	$8.80x10^{-11}$	1	bda,enhancer
rs12740374	1:109817590	G|T	*CELSR2*	$2.07x10^{-11}$	rs602633	$6.58x10^{-09}$	0.90	td,bda,enhancer,chromhmm,gerp
rs904320	1:214408457	A|T	1q32.3	$4.93x10^{-11}$	rs1795061	$8.80x10^{-11}$	0.98	td,bda,enhancer
rs1795061	1:214409280	T|C	1q32.3	$5.62x10^{-11}$	rs1795061	$8.80x10^{-11}$	1	td,bda,enhancer,self
rs629301	1:109818306	G|T	*CELSR2*	$7.32x10^{-11}$	rs602633	$6.58x10^{-09}$	0.90	td,bda,enhancer,chromhmm,gerp
rs10985349	9:124425243	C|T	*DAB2IP*	$7.46x10^{-11}$	rs10985349	$2.40x10^{-11}$	1	td,bda,enhancer,self
rs10985350	9:124429196	A|C	*DAB2IP*	$1.67x10^{-10}$	rs10985349	$2.40x10^{-11}$	0.81	td,bda,enhancer
rs660240	1:109817838	T|C	*CELSR2*	$1.88x10^{-10}$	rs602633	$6.58x10^{-09}$	0.96	td,bda,enhancer,chromhmm,gerp
rs7528419	1:109817192	A|G	*CELSR2*	$4.03x10^{-10}$	rs602633	$6.58x10^{-09}$	0.90	td,bda,enhancer,chromhmm,gerp
rs1795064	1:214406272	C|T	1q32.3	$5.07x10^{-10}$	rs1795061	$8.80x10^{-11}$	0.97	td,bda,enhancer
rs1795062	1:214406721	T|C	1q32.3	$5.73x10^{-10}$	rs1795061	$8.80x10^{-11}$	0.97	td,bda,enhancer
rs1660371	1:214409248	T|A	1q32.3	$9.29x10^{-10}$	rs1795061	$8.80x10^{-11}$	0.97	td,bda,enhancer
rs1795063	1:214406508	G|A	1q32.3	$9.72x10^{-10}$	rs1795061	$8.80x10^{-11}$	0.97	td,bda,enhancer
rs9316871	13:22861921	A|G	13q12.11	$1.26x10^{-09}$	rs9316871	$4.80x10^{-10}$	1	td,bda,enhancer,self
rs9506822	13:22862220	A|G	13q12.11	$1.89x10^{-09}$	rs9316871	$4.80x10^{-10}$	0.87	td,bda,enhancer
rs2836411	21:39819830	C|T	*ERG*	$4.72x10^{-09}$	rs2836411	$5.80x10^{-09}$	1	bda,enhancer,self

107

BIOINFORMATIC IDENTIFICATION OF CANDIDATE AAA GENES AND PATHWAYS USING DEPICT

An integrated gene function analysis was performed using the DEPICT version 1.1 tool[108]. DEPICT was installed, tested and run using meta-GWAS summary statistics following the recommended procedure outlined at https://github.com/perslab/depict. Two separate runs were performed using either all independent SNPs with discovery metaGWAS $P<5x10^{-6}$ or just those 10 SNPs which reached $P<1x10^{-6}$ in the combined analysis. Results are shown in **Online Table XVIII** and the full dataset is available in the online data supplement.

Online Table XVIII, DEPICT gene enrichment sets (nominal P<0.05) based on the top 10 validated loci.

There is a notable presence of descriptions associated with transforming growth factor beta regulation, lipoprotein metabolism, inflammation induced extracellular matrix remodelling (eg. RFX1), vascular smooth muscle cell function, vascular injury (including haemorrhage), immune cell function (particularly T & B cells), acute phase response (including IL6 secretion), apoptosis, hyperglycemia and the PIK3K, JNK and MAPK cascades. In addition, there are several descriptions associated with long bone size, an observation which may be consistent with previous reports linking height with cardiovascular disease risk. All gene sets had a false discovery rate > 0.2 with the exception of the most significant gene set, MP:0006396 (decreased long bone epiphyseal plate size), where the FDR was <0.2. This table spans 11 pages.

Original gene set ID	Original gene set description	DEPICT Nominal P-value
MP:0006396	decreased long bone epiphyseal plate size	$1.14x10^{-9}$
GO:0034381	plasma lipoprotein particle clearance	$5.22x10^{-7}$
ENSG00000205250	E2F4 PPI subnetwork	$1.27x10^{-6}$
ENSG00000132005	RFX1 PPI subnetwork	$2.28x10^{-6}$
MP:0000708	thymus hyperplasia	$6.32x10^{-6}$
ENSG00000167553	TUBA1C PPI subnetwork	$3.18x10^{-5}$
ENSG00000170421	KRT8 PPI subnetwork	$9.59x10^{-5}$
MP:0003645	increased pancreatic beta cell number	$1.12x10^{-4}$
ENSG00000166866	MYO1A PPI subnetwork	$2.32x10^{-4}$
REACTOME	REACTOME_apoptotic_execution__phase	$2.92x10^{-4}$
MP:0008182	decreased marginal zone B cell number	$3.06x10^{-4}$
GO:0008375	acetylglucosaminyltransferase activity	$3.62x10^{-4}$
ENSG00000131941	RHPN2 PPI subnetwork	$3.75x10^{-4}$
ENSG00000169710	FASN PPI subnetwork	$4.55x10^{-4}$
REACTOME	Reactome_apoptotic_cleavage_of_cellular_proteins	$5.46x10^{-4}$
ENSG00000013297	CLDN11 PPI subnetwork	$6.28x10^{-4}$
ENSG00000070159	PTPN3 PPI subnetwork	$6.56x10^{-4}$
ENSG00000091409	ITGA6 PPI subnetwork	$7.26x10^{-4}$
ENSG00000178209	PLEC PPI subnetwork	$8.57x10^{-4}$
REACTOME	Reactome_p75_ntr_receptor:mediated_signalling	$1.07x10^{-3}$
GO:0001890	placenta development	$1.08x10^{-3}$
ENSG00000164344	KLKB1 PPI subnetwork	$1.09x10^{-3}$
MP:0002136	abnormal kidney physiology	$1.17x10^{-3}$
MP:0002655	abnormal keratinocyte morphology	$1.45x10^{-3}$

Original gene set ID	Original gene set description	DEPICT Nominal P-value
ENSG00000143375	CGN PPI subnetwork	$1.48x10^{-3}$
MP:0005595	abnormal vascular smooth muscle physiology	$1.55x10^{-3}$
ENSG00000122641	INHBA PPI subnetwork	$1.79x10^{-3}$
MP:0002764	short tibia	$1.79x10^{-3}$
MP:0003662	abnormal long bone epiphyseal plate proliferative zone	$2.01x10^{-3}$
ENSG00000169047	IRS1 PPI subnetwork	$2.21x10^{-3}$
ENSG00000125503	PPP1R12C PPI subnetwork	$2.23x10^{-3}$
MP:0001179	thick pulmonary interalveolar septum	$2.30x10^{-3}$
GO:0043256	laminin complex	$2.33x10^{-3}$
ENSG00000116809	ZBTB17 PPI subnetwork	$2.46x10^{-3}$
GO:0050431	transforming growth factor beta binding	$2.47x10^{-3}$
ENSG00000039560	RAI14 PPI subnetwork	$2.60x10^{-3}$
ENSG00000164733	CTSB PPI subnetwork	$2.64x10^{-3}$
ENSG00000139567	ACVRL1 PPI subnetwork	$2.75x10^{-3}$
MP:0005590	increased vasodilation	$3.45x10^{-3}$
GO:0071813	lipoprotein particle binding	$3.51x10^{-3}$
GO:0071814	protein-lipid complex binding	$3.51x10^{-3}$
MP:0002082	postnatal lethality	$3.53x10^{-3}$
GO:0071902	positive regulation of protein serine/threonine kinase activity	$3.87x10^{-3}$
ENSG00000130147	SH3BP4 PPI subnetwork	$3.91x10^{-3}$
GO:0005178	integrin binding	$4.08x10^{-3}$
ENSG00000133056	PIK3C2B PPI subnetwork	$4.40x10^{-3}$
ENSG00000172725	CORO1B PPI subnetwork	$4.45x10^{-3}$
ENSG00000136286	MYO1G PPI subnetwork	$4.65x10^{-3}$
ENSG00000078142	PIK3C3 PPI subnetwork	$4.72x10^{-3}$
MP:0005095	decreased T cell proliferation	$4.84x10^{-3}$
ENSG00000145715	RASA1 PPI subnetwork	$4.96x10^{-3}$
ENSG00000104725	ENSG00000104725 PPI subnetwork	$5.08x10^{-3}$
KEGG_PATHWAYS	KEGG_PATHWAYS_IN_CANCER	$5.17x10^{-3}$
GO:0008194	UDP-glycosyltransferase activity	$5.46x10^{-3}$
ENSG00000078747	ITCH PPI subnetwork	$5.48x10^{-3}$
ENSG00000149257	SERPINH1 PPI subnetwork	$5.79x10^{-3}$
ENSG00000114062	UBE3A PPI subnetwork	$5.85x10^{-3}$
ENSG00000139144	PIK3C2G PPI subnetwork	$5.85x10^{-3}$
ENSG00000143393	PI4KB PPI subnetwork	$5.87x10^{-3}$
ENSG00000148498	PARD3 PPI subnetwork	$6.00x10^{-3}$
ENSG00000196455	PIK3R4 PPI subnetwork	$6.19x10^{-3}$
ENSG00000148660	CAMK2G PPI subnetwork	$6.48x10^{-3}$
ENSG00000034152	MAP2K3 PPI subnetwork	$6.58x10^{-3}$
ENSG00000123124	WWP1 PPI subnetwork	$6.95x10^{-3}$
MP:0008813	decreased common myeloid progenitor cell number	$7.37x10^{-3}$
ENSG00000204175	GPRIN2 PPI subnetwork	$7.39x10^{-3}$
GO:0001772	immunological synapse	$7.40x10^{-3}$
REACTOME	Reactome_caspase:mediated_cleavage_of_cytoskeletal_proteins	$7.46x10^{-3}$
ENSG00000017427	IGF1 PPI subnetwork	$7.51x10^{-3}$
MP:0001954	respiratory distress	$7.56x10^{-3}$
GO:0016051	carbohydrate biosynthetic process	$7.65x10^{-3}$

Original gene set ID	Original gene set description	DEPICT Nominal P-value
GO:0043406	positive regulation of MAP kinase activity	$7.65x10^{-3}$
REACTOME	Reactome_cell_death_signalling_via_nrage_nrif_and_nade	$7.67x10^{-3}$
MP:0000180	abnormal circulating cholesterol level	$7.71x10^{-3}$
ENSG00000170759	KIF5B PPI subnetwork	$7.79x10^{-3}$
ENSG00000180530	NRIP1 PPI subnetwork	$7.86x10^{-3}$
ENSG00000138771	SHROOM3 PPI subnetwork	$7.89x10^{-3}$
ENSG00000065882	TBC1D1 PPI subnetwork	$7.97x10^{-3}$
ENSG00000138592	USP8 PPI subnetwork	$7.99x10^{-3}$
MP:0001915	intracranial hemorrhage	$8.00x10^{-3}$
ENSG00000131746	TNS4 PPI subnetwork	$8.01x10^{-3}$
MP:0004883	abnormal vascular wound healing	$8.15x10^{-3}$
ENSG00000091073	ENSG00000091073 PPI subnetwork	$8.22x10^{-3}$
ENSG00000081189	MEF2C PPI subnetwork	$8.24x10^{-3}$
ENSG00000154415	PPP1R3A PPI subnetwork	$8.33x10^{-3}$
ENSG00000188313	PLSCR1 PPI subnetwork	$8.55x10^{-3}$
MP:0004933	abnormal epididymis epithelium morphology	$8.61x10^{-3}$
ENSG00000147065	MSN PPI subnetwork	$8.64x10^{-3}$
ENSG00000165409	TSHR PPI subnetwork	$8.64x10^{-3}$
ENSG00000106992	AK1 PPI subnetwork	$8.69x10^{-3}$
GO:0007292	female gamete generation	$8.93x10^{-3}$
ENSG00000144061	NPHP1 PPI subnetwork	$8.95x10^{-3}$
MP:0003419	delayed endochondral bone ossification	$8.98x10^{-3}$
ENSG00000110880	CORO1C PPI subnetwork	$9.04x10^{-3}$
ENSG00000197879	MYO1C PPI subnetwork	$9.22x10^{-3}$
ENSG00000176476	CCDC101 PPI subnetwork	$9.31x10^{-3}$
ENSG00000176108	CHMP6 PPI subnetwork	$9.44x10^{-3}$
REACTOME	Reactome_integrin_cell_surface_interactions	$9.47x10^{-3}$
GO:0016758	transferase activity, transferring hexosyl groups	$9.49x10^{-3}$
ENSG00000103197	TSC2 PPI subnetwork	$9.49x10^{-3}$
MP:0003909	increased eating behavior	$9.57x10^{-3}$
MP:0000716	abnormal immune system cell morphology	$9.63x10^{-3}$
MP:0008803	abnormal placental labyrinth vasculature morphology	$9.67x10^{-3}$
ENSG00000137801	THBS1 PPI subnetwork	$9.71x10^{-3}$
ENSG00000130522	JUND PPI subnetwork	$9.72x10^{-3}$
GO:0005088	Ras guanyl-nucleotide exchange factor activity	$9.78x10^{-3}$
ENSG00000170581	STAT2 PPI subnetwork	$9.79x10^{-3}$
ENSG00000173757	STAT5B PPI subnetwork	$9.84x10^{-3}$
GO:0043277	apoptotic cell clearance	$9.98x10^{-3}$
GO:0006917	induction of apoptosis	0.01
MP:0001828	abnormal T cell activation	0.01
ENSG00000171241	SHCBP1 PPI subnetwork	0.01
REACTOME	REACTOME_apoptosis	0.01
MP:0011106	partial embryonic lethality before somite formation	0.01
GO:0030169	low-density lipoprotein particle binding	0.01
MP:0003731	abnormal retinal outer nuclear layer morphology	0.01
MP:0009400	decreased skeletal muscle fiber size	0.01
ENSG00000137693	YAP1 PPI subnetwork	0.01

Original gene set ID	Original gene set description	DEPICT Nominal P-value
REACTOME	Reactome_nrage_signals_death_through_jnk	0.01
ENSG00000145794	MEGF10 PPI subnetwork	0.01
MP:0008478	increased spleen white pulp amount	0.01
MP:0005079	defective cytotoxic T cell cytolysis	0.01
ENSG00000141506	PIK3R5 PPI subnetwork	0.01
GO:0000989	transcription factor binding transcription factor activity	0.01
MP:0002161	abnormal fertility/fecundity	0.01
GO:0040029	regulation of gene expression, epigenetic	0.01
ENSG00000115963	RND3 PPI subnetwork	0.01
GO:0043236	laminin binding	0.01
ENSG00000211660	ENSG00000211660 PPI subnetwork	0.01
ENSG00000211653	ENSG00000211653 PPI subnetwork	0.01
ENSG00000160310	PRMT2 PPI subnetwork	0.01
ENSG00000127688	GAN PPI subnetwork	0.01
ENSG00000167711	SERPINF2 PPI subnetwork	0.01
GO:0043491	protein kinase B signaling cascade	0.01
ENSG00000136068	FLNB PPI subnetwork	0.01
GO:0002020	protease binding	0.01
ENSG00000198053	SIRPA PPI subnetwork	0.01
ENSG00000182319	SGK223 PPI subnetwork	0.01
ENSG00000174292	TNK1 PPI subnetwork	0.01
ENSG00000132825	PPP1R3D PPI subnetwork	0.01
GO:0051015	actin filament binding	0.01
ENSG00000140443	IGF1R PPI subnetwork	0.01
MP:0000281	abnormal interventricular septum morphology	0.01
ENSG00000067560	RHOA PPI subnetwork	0.01
MP:0006094	increased fat cell size	0.01
ENSG00000197555	SIPA1L1 PPI subnetwork	0.01
ENSG00000183386	FHL3 PPI subnetwork	0.01
MP:0003229	abnormal vitelline vasculature morphology	0.01
MP:0001231	abnormal epidermis stratum basale morphology	0.01
MP:0000511	abnormal intestinal mucosa morphology	0.01
KEGG	KEGG_ACUTE_MYELOID_LEUKEMIA	0.01
GO:0007254	JNK cascade	0.01
GO:0008624	induction of apoptosis by extracellular signals	0.01
GO:0000988	protein binding transcription factor activity	0.02
MP:0002452	abnormal antigen presenting cell physiology	0.02
ENSG00000185950	IRS2 PPI subnetwork	0.02
KEGG	KEGG_leukocyte_transendothelial_migration	0.02
GO:0051568	histone H3-K4 methylation	0.02
ENSG00000165516	KLHDC2 PPI subnetwork	0.02
REACTOME	Reactome_cell_surface_interactions_at_the_vascular_wall	0.02
ENSG00000198838	RYR3 PPI subnetwork	0.02
MP:0001711	abnormal placenta morphology	0.02
GO:0014910	regulation of smooth muscle cell migration	0.02
ENSG00000104960	PTOV1 PPI subnetwork	0.02
GO:0001968	fibronectin binding	0.02

Original gene set ID	Original gene set description	DEPICT Nominal P-value
GO:0012502	induction of programmed cell death	0.02
ENSG00000100364	KIAA0930 PPI subnetwork	0.02
ENSG00000165410	CFL2 PPI subnetwork	0.02
MP:0004994	abnormal brain wave pattern	0.02
MP:0001552	increased circulating triglyceride level	0.02
ENSG00000116141	MARK1 PPI subnetwork	0.02
GO:0032403	protein complex binding	0.02
ENSG00000179364	PACS2 PPI subnetwork	0.02
MP:0001559	hyperglycemia	0.02
ENSG00000105851	PIK3CG PPI subnetwork	0.02
MP:0004031	insulitis	0.02
MP:0010124	decreased bone mineral content	0.02
ENSG00000165197	FIGF PPI subnetwork	0.02
ENSG00000126561	STAT5A PPI subnetwork	0.02
ENSG00000136156	ITM2B PPI subnetwork	0.02
ENSG00000164327	RICTOR PPI subnetwork	0.02
ENSG00000159166	LAD1 PPI subnetwork	0.02
MP:0001716	abnormal placenta labyrinth morphology	0.02
MP:0002427	disproportionate dwarf	0.02
ENSG00000105371	ICAM4 PPI subnetwork	0.02
ENSG00000165476	REEP3 PPI subnetwork	0.02
GO:0046625	sphingolipid binding	0.02
MP:0000585	kinked tail	0.02
MP:0000889	abnormal cerebellar molecular layer	0.02
ENSG00000105699	LSR PPI subnetwork	0.02
GO:0005545	1-phosphatidylinositol binding	0.02
MP:0001134	absent corpus luteum	0.02
ENSG00000100097	LGALS1 PPI subnetwork	0.02
MP:0002079	increased circulating insulin level	0.02
ENSG00000150093	ITGB1 PPI subnetwork	0.02
GO:0001871	pattern binding	0.02
GO:0030247	polysaccharide binding	0.02
ENSG00000126934	MAP2K2 PPI subnetwork	0.02
ENSG00000110395	CBL PPI subnetwork	0.02
ENSG00000179151	EDC3 PPI subnetwork	0.02
ENSG00000154162	CDH12 PPI subnetwork	0.02
ENSG00000184363	PKP3 PPI subnetwork	0.02
ENSG00000020577	SAMD4A PPI subnetwork	0.02
MP:0004139	abnormal gastric parietal cell morphology	0.02
ENSG00000168476	REEP4 PPI subnetwork	0.02
ENSG00000110651	CD81 PPI subnetwork	0.02
ENSG00000134184	GSTM1 PPI subnetwork	0.02
ENSG00000105376	ICAM5 PPI subnetwork	0.02
ENSG00000196954	CASP4 PPI subnetwork	0.02
MP:0003704	abnormal hair follicle development	0.02
ENSG00000050820	BCAR1 PPI subnetwork	0.02
ENSG00000151748	SAV1 PPI subnetwork	0.02

Original gene set ID	Original gene set description	DEPICT Nominal P-value
GO:0003714	transcription corepressor activity	0.02
ENSG00000115904	SOS1 PPI subnetwork	0.02
ENSG00000175793	SFN PPI subnetwork	0.02
ENSG00000100345	MYH9 PPI subnetwork	0.02
GO:0035091	phosphatidylinositol binding	0.02
ENSG00000149930	TAOK2 PPI subnetwork	0.02
GO:0042054	histone methyltransferase activity	0.02
MP:0000689	abnormal spleen morphology	0.02
GO:0001892	embryonic placenta development	0.02
ENSG00000130294	KIF1A PPI subnetwork	0.02
ENSG00000148965	SAA4 PPI subnetwork	0.02
GO:0034774	secretory granule lumen	0.02
ENSG00000166483	WEE1 PPI subnetwork	0.02
ENSG00000110237	ARHGEF17 PPI subnetwork	0.02
GO:0032608	interferon-beta production	0.02
ENSG00000152518	ZFP36L2 PPI subnetwork	0.02
MP:0010792	abnormal stomach mucosa morphology	0.02
ENSG00000189319	FAM53B PPI subnetwork	0.02
ENSG00000117461	PIK3R3 PPI subnetwork	0.02
GO:0034362	low-density lipoprotein particle	0.02
ENSG00000134072	CAMK1 PPI subnetwork	0.02
ENSG00000163362	C1orf106 PPI subnetwork	0.02
MP:0002816	colitis	0.02
GO:0050900	leukocyte migration	0.03
GO:0044304	main axon	0.03
ENSG00000071909	MYO3B PPI subnetwork	0.03
ENSG00000100714	MTHFD1 PPI subnetwork	0.03
ENSG00000198836	OPA1 PPI subnetwork	0.03
ENSG00000197442	MAP3K5 PPI subnetwork	0.03
ENSG00000206306	HLA-DRB1 PPI subnetwork	0.03
ENSG00000206240	HLA-DRB1 PPI subnetwork	0.03
GO:0031983	vesicle lumen	0.03
KEGG	KEGG_regulation_of_actin_cytoskeleton	0.03
GO:0004713	protein tyrosine kinase activity	0.03
GO:0006953	acute-phase response	0.03
GO:0003712	transcription cofactor activity	0.03
MP:0000295	trabecula carnea hypoplasia	0.03
ENSG00000105647	PIK3R2 PPI subnetwork	0.03
GO:0060205	cytoplasmic membrane-bounded vesicle lumen	0.03
ENSG00000107566	ERLIN1 PPI subnetwork	0.03
ENSG00000114270	COL7A1 PPI subnetwork	0.03
ENSG00000135930	EIF4E2 PPI subnetwork	0.03
MP:0006413	increased T cell apoptosis	0.03
ENSG00000211949	ENSG00000211949 PPI subnetwork	0.03
ENSG00000125731	SH2D3A PPI subnetwork	0.03
MP:0000414	alopecia	0.03
ENSG00000160691	SHC1 PPI subnetwork	0.03

Original gene set ID	Original gene set description	DEPICT Nominal P-value
MP:0001282	short vibrissae	0.03
MP:0003996	clonic seizures	0.03
ENSG00000019991	HGF PPI subnetwork	0.03
MP:0010025	decreased total body fat amount	0.03
GO:0007568	aging	0.03
GO:0042809	vitamin D receptor binding	0.03
MP:0005331	insulin resistance	0.03
GO:0045682	regulation of epidermis development	0.03
MP:0001923	reduced female fertility	0.03
MP:0001219	thick epidermis	0.03
ENSG00000068615	REEP1 PPI subnetwork	0.03
ENSG00000171219	CDC42BPG PPI subnetwork	0.03
MP:0009583	increased keratinocyte proliferation	0.03
ENSG00000105810	CDK6 PPI subnetwork	0.03
ENSG00000105662	CRTC1 PPI subnetwork	0.03
MP:0003957	abnormal nitric oxide homeostasis	0.03
KEGG	KEGG_small_cell_lung_cancer	0.03
GO:0030669	clathrin-coated endocytic vesicle membrane	0.03
ENSG00000100030	MAPK1 PPI subnetwork	0.03
GO:0046328	regulation of JNK cascade	0.03
GO:0014070	response to organic cyclic compound	0.03
GO:0033500	carbohydrate homeostasis	0.03
GO:0042593	glucose homeostasis	0.03
REACTOME	Reactome_ptm_gamma_carboxylation_hypusine_formation_and_arylsulfatase_activation	0.03
REACTOME	Reactome_regulation_of_signaling_by_cbl	0.03
MP:0002418	increased susceptibility to viral infection	0.03
MP:0003721	increased tumor growth/size	0.03
GO:0071845	cellular component disassembly at cellular level	0.03
GO:0030518	intracellular steroid hormone receptor signaling pathway	0.03
ENSG00000116824	CD2 PPI subnetwork	0.03
MP:0003566	abnormal cell adhesion	0.03
GO:0034061	DNA polymerase activity	0.03
ENSG00000141968	VAV1 PPI subnetwork	0.03
GO:0001701	in utero embryonic development	0.03
MP:0000166	abnormal chondrocyte morphology	0.03
MP:0003400	kinked neural tube	0.03
GO:0000790	nuclear chromatin	0.03
ENSG00000197102	DYNC1H1 PPI subnetwork	0.03
GO:0043566	structure-specific DNA binding	0.03
ENSG00000075413	MARK3 PPI subnetwork	0.03
GO:0000271	polysaccharide biosynthetic process	0.03
REACTOME	Reactome_cell:cell_communication	0.03
MP:0000410	waved hair	0.03
ENSG00000154556	SORBS2 PPI subnetwork	0.03
ENSG00000104368	PLAT PPI subnetwork	0.03
GO:0043123	positive regulation of I-kappaB kinase/NF-kappaB cascade	0.03
ENSG00000156127	BATF PPI subnetwork	0.03

Original gene set ID	Original gene set description	DEPICT Nominal P-value
ENSG00000132470	ITGB4 PPI subnetwork	0.03
GO:0038024	cargo receptor activity	0.03
ENSG00000100014	SPECC1L PPI subnetwork	0.03
ENSG00000163083	INHBB PPI subnetwork	0.03
ENSG00000110931	CAMKK2 PPI subnetwork	0.03
MP:0002088	abnormal embryonic growth/weight/body size	0.03
GO:0016571	histone methylation	0.03
GO:0033559	unsaturated fatty acid metabolic process	0.03
MP:0005350	increased susceptibility to autoimmune disorder	0.03
ENSG00000136111	TBC1D4 PPI subnetwork	0.03
REACTOME	Reactome_nephrin_interactions	0.03
ENSG00000182195	LDOC1 PPI subnetwork	0.03
ENSG00000123685	BATF3 PPI subnetwork	0.03
ENSG00000215699	ENSG00000215699 PPI subnetwork	0.03
GO:0005720	nuclear heterochromatin	0.03
ENSG00000092969	TGFB2 PPI subnetwork	0.03
KEGG	KEGG_ECM_receptor_interaction	0.03
MP:0001201	translucent skin	0.03
GO:0016278	lysine N-methyltransferase activity	0.03
GO:0016279	protein-lysine N-methyltransferase activity	0.03
ENSG00000196586	MYO6 PPI subnetwork	0.03
GO:0004702	receptor signaling protein serine/threonine kinase activity	0.03
ENSG00000072518	MARK2 PPI subnetwork	0.03
ENSG00000165025	SYK PPI subnetwork	0.03
MP:0002109	abnormal limb morphology	0.03
ENSG00000157764	BRAF PPI subnetwork	0.03
ENSG00000152256	PDK1 PPI subnetwork	0.03
ENSG00000065618	COL17A1 PPI subnetwork	0.04
ENSG00000169220	RGS14 PPI subnetwork	0.04
ENSG00000100311	PDGFB PPI subnetwork	0.04
ENSG00000134202	GSTM3 PPI subnetwork	0.04
ENSG00000142515	KLK3 PPI subnetwork	0.04
MP:0002619	abnormal lymphocyte morphology	0.04
ENSG00000161395	PGAP3 PPI subnetwork	0.04
ENSG00000145431	PDGFC PPI subnetwork	0.04
ENSG00000170962	PDGFD PPI subnetwork	0.04
ENSG00000153879	CEBPG PPI subnetwork	0.04
ENSG00000077380	DYNC1I2 PPI subnetwork	0.04
ENSG00000197122	SRC PPI subnetwork	0.04
MP:0004399	abnormal cochlear outer hair cell morphology	0.04
ENSG00000174996	KLC2 PPI subnetwork	0.04
MP:0002376	abnormal dendritic cell physiology	0.04
MP:0000709	enlarged thymus	0.04
MP:0008706	decreased interleukin-6 secretion	0.04
MP:0004686	decreased length of long bones	0.04
GO:0050810	regulation of steroid biosynthetic process	0.04
ENSG00000138396	ENSG00000138396 PPI subnetwork	0.04

Original gene set ID	Original gene set description	DEPICT Nominal P-value
ENSG00000148400	NOTCH1 PPI subnetwork	0.04
ENSG00000137171	KLC4 PPI subnetwork	0.04
ENSG00000196396	PTPN1 PPI subnetwork	0.04
ENSG00000148672	GLUD1 PPI subnetwork	0.04
GO:0000975	regulatory region DNA binding	0.04
GO:0001067	regulatory region nucleic acid binding	0.04
GO:0022411	cellular component disassembly	0.04
ENSG00000026025	VIM PPI subnetwork	0.04
ENSG00000061273	HDAC7 PPI subnetwork	0.04
ENSG00000104067	TJP1 PPI subnetwork	0.04
MP:0004813	absent linear vestibular evoked potential	0.04
ENSG00000091136	LAMB1 PPI subnetwork	0.04
KEGG	KEGG_renal_cell_carcinoma	0.04
KEGG	KEGG_focal_adhesion	0.04
GO:0031581	hemidesmosome assembly	0.04
ENSG00000141068	KSR1 PPI subnetwork	0.04
MP:0004214	abnormal long bone diaphysis morphology	0.04
ENSG00000123836	PFKFB2 PPI subnetwork	0.04
ENSG00000168090	COPS6 PPI subnetwork	0.04
ENSG00000132356	PRKAA1 PPI subnetwork	0.04
GO:0031093	platelet alpha granule lumen	0.04
GO:0048545	response to steroid hormone stimulus	0.04
MP:0003109	short femur	0.04
ENSG00000113758	DBN1 PPI subnetwork	0.04
GO:0008276	protein methyltransferase activity	0.04
MP:0003383	abnormal gluconeogenesis	0.04
ENSG00000162614	NEXN PPI subnetwork	0.04
ENSG00000162614	NEXN PPI subnetwork	0.04
ENSG00000169641	LUZP1 PPI subnetwork	0.04
MP:0002152	abnormal brain morphology	0.04
ENSG00000204257	HLA-DMA PPI subnetwork	0.04
ENSG00000206229	ENSG00000206229 PPI subnetwork	0.04
ENSG00000206293	ENSG00000206293 PPI subnetwork	0.04
ENSG00000138439	FAM117B PPI subnetwork	0.04
GO:0006636	unsaturated fatty acid biosynthetic process	0.04
ENSG00000176444	CLK2 PPI subnetwork	0.04
MP:0000703	abnormal thymus morphology	0.04
REACTOME	Reactome_zinc_transporters	0.04
ENSG00000125952	MAX PPI subnetwork	0.04
GO:0046456	icosanoid biosynthetic process	0.04
ENSG00000132964	CDK8 PPI subnetwork	0.04
MP:0008688	decreased interleukin-2 secretion	0.04
ENSG00000196218	RYR1 PPI subnetwork	0.04
MP:0004770	abnormal synaptic vesicle recycling	0.04
ENSG00000121879	PIK3CA PPI subnetwork	0.04
ENSG00000196735	HLA-DQA1 PPI subnetwork	0.04
MP:0009355	increased liver triglyceride level	0.04

Original gene set ID	Original gene set description	DEPICT Nominal P-value
MP:0009399	increased skeletal muscle fiber size	0.04
ENSG00000160678	S100A1 PPI subnetwork	0.04
ENSG00000064999	ANKS1A PPI subnetwork	0.04
ENSG00000173327	MAP3K11 PPI subnetwork	0.04
GO:0051183	vitamin transporter activity	0.04
GO:0006690	icosanoid metabolic process	0.04
ENSG00000134363	FST PPI subnetwork	0.04
GO:0060053	neurofilament cytoskeleton	0.04
ENSG00000151914	DST PPI subnetwork	0.04
ENSG00000189079	ARID2 PPI subnetwork	0.04
ENSG00000065559	MAP2K4 PPI subnetwork	0.05
ENSG00000120709	FAM53C PPI subnetwork	0.05
MP:0002110	abnormal digit morphology	0.05
GO:0005976	polysaccharide metabolic process	0.05
ENSG00000054523	KIF1B PPI subnetwork	0.05
ENSG00000100906	NFKBIA PPI subnetwork	0.05
ENSG00000136518	ACTL6A PPI subnetwork	0.05
GO:0004709	MAP kinase kinase kinase activity	0.05
GO:0060711	labyrinthine layer development	0.05
KEGG_	KEGG_circadian_rhythm_mammal	0.05
REACTOME	Reactome_classical_antibody:mediated_complement_activation	0.05
ENSG00000211979	ENSG00000211979 PPI subnetwork	0.05
ENSG00000211973	ENSG00000211973 PPI subnetwork	0.05
ENSG00000172534	HCFC1 PPI subnetwork	0.05
ENSG00000136270	TBRG4 PPI subnetwork	0.05
GO:0032648	regulation of interferon-beta production	0.05
GO:0034375	high-density lipoprotein particle remodeling	0.05
ENSG00000185811	IKZF1 PPI subnetwork	0.05
ENSG00000198802	ENSG00000198802 PPI subnetwork	0.05
MP:0006262	testis tumor	0.05
ENSG00000171992	SYNPO PPI subnetwork	0.05
ENSG00000213341	CHUK PPI subnetwork	0.05
ENSG00000175197	DDIT3 PPI subnetwork	0.05
MP:0005150	cachexia	0.05
GO:0043122	regulation of I-kappaB kinase/NF-kappaB cascade	0.05
GO:0097006	regulation of plasma lipoprotein particle levels	0.05
ENSG00000162772	ATF3 PPI subnetwork	0.05
GO:0000122	negative regulation of transcription from RNA polymerase II promoter	0.05
GO:0005858	axonemal dynein complex	0.05
ENSG00000051382	PIK3CB PPI subnetwork	0.05
GO:0043405	regulation of MAP kinase activity	0.05
MP:0008722	abnormal chemokine secretion	0.05
KEGG	KEGG_chronic_myeloid_leukemia	0.05
REACTOME	Reactome_regulated_PROTEOLYSIS_OF_P75NTR	0.05
GO:0043588	skin development	0.05
GO:0010627	regulation of intracellular protein kinase cascade	0.05
GO:0044212	transcription regulatory region DNA binding	0.05

Original gene set ID	Original gene set description	DEPICT Nominal P-value
GO:0030027	lamellipodium	0.05
ENSG00000105976	MET PPI subnetwork	0.05
MP:0002792	abnormal retinal vasculature morphology	0.05
MP:0000069	kyphoscoliosis	0.05
GO:0034339	regulation of transcription from RNA polymerase II promoter by nuclear hormone receptor	0.05
ENSG00000141551	CSNK1D PPI subnetwork	0.05
MP:0005108	abnormal ulna morphology	0.05
MP:0002419	abnormal innate immunity	0.05
GO:0016757	transferase activity, transferring glycosyl groups	0.05
ENSG00000161800	RACGAP1 PPI subnetwork	0.05
MP:0006387	abnormal T cell number	0.05
GO:0005089	Rho guanyl-nucleotide exchange factor activity	0.05
ENSG00000117984	CTSD PPI subnetwork	0.05
ENSG00000105971	CAV2 PPI subnetwork	0.05
ENSG00000115085	ZAP70 PPI subnetwork	0.05
MP:0004609	vertebral fusion	0.05
ENSG00000135862	LAMC1 PPI subnetwork	0.05
MP:0003449	abnormal intestinal goblet cell morphology	0.05
MP:0002687	oligozoospermia	0.05
MP:0000714	increased thymocyte number	0.05
ENSG00000133030	MPRIP PPI subnetwork	0.05
ENSG00000079841	RIMS1 PPI subnetwork	0.05
ENSG00000130638	ATXN10 PPI subnetwork	0.05
MP:0002656	abnormal keratinocyte differentiation	0.05
ENSG00000129691	ASH2L PPI subnetwork	0.05
MP:0002650	abnormal ameloblast morphology	0.05
ENSG00000135503	ACVR1B PPI subnetwork	0.05
GO:0004715	non-membrane spanning protein tyrosine kinase activity	0.05
ENSG00000001497	LAS1L PPI subnetwork	0.05
GO:0018024	histone-lysine N-methyltransferase activity	0.05
GO:0000792	heterochromatin	0.05
ENSG00000111961	SASH1 PPI subnetwork	0.05
MP:0008840	abnormal spike wave discharge	0.05
ENSG00000139514	SLC7A1 PPI subnetwork	0.05
GO:0007249	I-kappaB kinase/NF-kappaB cascade	0.05

Functional effects of SNPs at AAA loci

1: Expression SNP database lookup (Online Table XIX)
Evidence for functional effects of AAA associated SNPs/loci was sought in two eQTL datasets curated by Andrew Johnson at the NIH National Heart Lung and Blood Institute, Framingham, USA. Firstly, index and proxy SNPs were queried in a collected database of expression SNP (eSNP) results. The collected eSNP results met criteria for statistical thresholds for association with gene transcript levels as described in the original papers. A general overview of a subset of >50 eQTL studies has been published[109], with specific citations for >100 studies included in the current query following here:

Blood cell related eQTL studies included fresh lymphocytes[110], fresh leukocytes[111] , leukocyte samples in individuals with Celiac disease[112], whole blood samples[113-126], lymphoblastoid cell lines (LCL) derived from asthmatic children[127, 128], HapMap LCL from 3 populations[129], a separate study on HapMap CEU LCL[130], additional LCL population samples[131-136], $CD19^+$ B cells[137], primary PHA-stimulated T cells[133, 135], $CD4^+$ T cells[138], peripheral blood monocytes[137, 139, 140] and $CD14^+$ monocytes before and after stimulation with LPS or interferon-gamma[141], $CD11^+$ dendritic cells before and after *Mycobacterium tuberculosis* infection[142] and a separate study of dendritic cells before or after stimulation with LPS, influenza or interferon-beta[143]. Micro-RNA QTLs[144] and DNase-I QTLs[145] were also queried for LCL.

Non-blood cell tissue eQTLs searched included omental and subcutaneous adipose[115, 134, 146, 147], stomach[147], endometrial carcinomas[148], ER+ and ER- breast cancer tumor cells[149], liver[147, 150-153], osteoblasts[154], intestine[155] and normal and cancerous colon[156], skeletal muscle[157], breast tissue (normal and cancer)[158, 159], lung[146, 160, 161], skin[134, 146, 162], primary fibroblasts[133, 135, 163], sputum[164], pancreatic islet cells[165] and heart tissue from left ventricles[146, 166] and left and right atria[167]. Micro-RNA QTLs were also queried for gluteal and abdominal adipose[168] and liver[169]. Further mRNA and micro-RNA QTLs were queried from ER+ invasive breast cancer samples, colon-, kidney renal clear-, lung- and prostate-adenocarcinoma samples[170].

Brain eQTL studies included brain cortex[139, 171, 172], cerebellar cortex[173], cerebellum[172, 174-177], frontal cortex[173, 175, 176], gliomas[178], hippocampus[173, 176], inferior olivary nucleus (from medulla)[173], intralobular white matter[173], occiptal cortex[173], parietal lobe[174], pons[175], pre-frontal cortex[176, 177, 179, 180], putamen (at the level of anterior commussure)[173], substantia nigra[173], temporal cortex[172, 173, 175, 176], thalamus[176] and visual cortex[177].

Secondly, additional eQTL data were integrated from online sources including ScanDB, the Broad Institute GTex browser, and the Pritchard Lab (eqtl.uchicago.edu). Cerebellum, parietal lobe and liver eQTL data was downloaded from ScanDB and cis-eQTLs were limited to those with $P<1.0x10^{-6}$ and trans-eQTLs with $P<5.0x10^{-8}$. The top 1000 eQTL results were downloaded from the GTex Browser at the Broad Institute for 9 tissues on 11/26/2013: thyroid, leg skin (sun exposed), tibial nerve, tibial artery, skeletal muscle, lung, heart (left ventricle), whole blood, and subcutaneous adipose[146]. All GTex results had associations with $P<8.4x10^{-7}$.

2: eQTL lookup in the Advanced Study of Aortic Pathology (Online Table XX and Online Figure III)
eQTL data were obtained from the Advanced Study of Aortic Pathology (ASAP) dataset which has previously been described[181]. Tissue samples were selected from individuals undergoing aortic valve surgery. Five tissue types were collected from each patient: mammary artery, liver, aorta intima-media, aorta adventitia, and heart. RNA was extracted from tissues and hybridised to Affymetrix ST 1.0 exon arrays (Santa Clara, CA, USA) and data were robust multiarray average normalised before log2 transformation. DNA extracted from whole blood was genotyped on the Illumina 610w-Quad

bead array (San Diego, CA, USA) platform. SNPs with >95% call rate were used for imputation, and imputed SNPs with quality scores of MACH <0.3 were excluded from analysis. An additive model for associations between SNPs and gene expression was assumed. Genotypes for 5 of the 10 lead SNPs at AAA risk loci were directly genotyped on Illumina 610wQuad arrays.

3: RNA-seq (Online Table XXI)

RNA-seq data were obtained from the Stockholm-Tartu Atherosclerosis Reverse Network Engineering Task (STARNET) database[182] (http://www.mountsinai.org/profiles/johan-bjorkegren). These consist of RNA-seq data from 9 cardiovascular tissues from up to 600 CAD patients obtained during coronary artery by-pass grafting surgery. Gene expression was measured with a standard RNA-seq protocol, followed by normalization of raw read counts to adjust for library size and batch effects. Adjusted read counts were subsequently log_2-transformed, and the association between genotype and expression was tested using a linear model. Permutation was used to assess the statistical significance. Significant results for the lead SNPs at each AAA risk locus are shown in **Online Table XX.**

4: Peripheral blood monocyte eQTL analysis (Online Table XXII)

Data from an eQTL analysis of peripheral blood monocytes was obtained from the Cardiogenics Consortium [183, 184]. The description of the cohort sample collection and processing and the eQTL analysis have previously been described in detail. Briefly, genome-wide expression and genotype data were obtained from peripheral blood monocytes from 363 patients with CAD or myocardial infarction and 395 healthy individuals. Expression profiling was performed using the Illumina HumanRef-8 v3 beadchip array (Illumina Inc., San Diego, CA) containing 24,516 probes corresponding to 18,311 distinct genes and 21,793 Ref Seq annotated transcripts. Genome-wide genotyping was carried out using two Illumina arrays, the Sentrix Human Custom 1.2M array and the Human 610 Quad Custom array. SNP analysis was restricted to autosomal SNPs with MAF >0.01, call rate >0.95 and HWE testing $P>1x10^{-5}$. After quality control, 522,603 SNPs were used for association analyses with expression. All replicated AAA associated SNPs were tested for association with regional gene expression. Significant results are shown in **Online Table XXII**.

Online Table XIX: eQTL data (1). This table spans 2 pages.

Locus	Lead AAA SNP	Gene	Tissue	Transcript	Proxy SNP looked up in eQTL database			Peak regional SNP in eQTL database		
					Proxy SNP	r2 to AAA SNP	eQTL P-Value for lead AAA SNP or proxy	Peak SNP	r2 (Peak SNP to AAA SNP)	eQTL P-Value for peak SNP
**1q21.3**	rs4129267	***IL6R***	Average in 10 brain regions [PMID 25174004]	*INTS3*	rs4576655	1	5.59x10^{-06}	rs12068901	NA	3.32x10^{-42}
			**Average in 10 brain regions [PMID 25174004]**	***SLC39A1***	**rs4845372**	**0.965**	**4.75x10^{-06}**	**rs4845372**	**0.965**	**4.75x10^{-06}**
			**Average in 10 brain regions [PMID 25174004]**	***PYGO2***	**rs6684439**	**0.839**	**2.96x10^{-06}**	**rs6684439**	**0.839**	**2.96x10^{-06}**
			**CD14+ monocytes (untreated) [PMID 24604202]**	***IL6R***			**6.64x10^{-04}**	**rs7518199**	**0.965**	**5.27x10^{-04}**
			Intestine (normal ileum) [PMID 23474282]	*IL6R*			8.81x10^{-06}	rs7553796	0.49	8.20x10^{-11}
			**Lymph [PMID 17873875]**	***IL6R***	**rs4537545**	**1**	**2.63x10^{-03}**	**rs4845623**	**0.965**	**1.88x10^{-03}**
			**Prefrontal cortex (all samples) [PMID 23622250]**	***MUC1***	**rs8192284**	**0.982**	**6.33x10^{-05}**	**rs8192284**	**0.982**	**6.33x10^{-05}**
			**Whole blood (Battle) [PMID 24092820]**	***IL6R***	**rs4537545**	**1**	**2.62x10^{-20}**	**rs4537545**	**1**	**2.62x10^{-20}**
			**Whole blood (CHARGE) [PMID 24013639]**	***IL6R***			**3.15x10^{-27}**	**rs4537545**	**1**	**2.02x10^{-29}**
			Whole blood (CHARGE) [PMID 24013639]	*UBE2Q1*			9.75x10^{-08}	rs6660775	0.058	3.93x10^{-21}
**1p13.3**	**rs602633**	**CELSR2/*SORT1***	**CD14+ monocytes (24h LPS stimulated) [PMID 24604202]**	***PSRC1***	**rs599839**	**1**	**9.50x10^{-05}**	**rs646776**	**0.895**	**3.53x10^{-05}**
			**CD14+ monocytes (IFNg stimulated) [PMID 24604202]**	***PSRC1***	**rs599839**	**1**	**4.25x10^{-13}**	**rs646776**	**0.895**	**7.40x10^{-14}**
			**CD14+ monocytes (untreated) [PMID 24604202]**	***PSRC1***	**rs599839**	**1**	**7.31x10^{-44}**	**rs599839**	**1**	**7.31x10^{-44}**
			**Cerebellum (all samples) [PMID 23622250]**	***PSRC1***			**9.12x10^{-06}**	**rs602633**	**Same SNP**	**9.12x10^{-06}**
			**Cerebellum (Huntington's) [PMID 23622250]**	***PSRC1***	**rs646776**	**0.895**	**5.19x10^{-05}**	**rs646776**	**0.895**	**5.19x10^{-05}**
			**Liver (ScanDB)**	***SORT1***	**rs646776**	**0.895**	**3.18x10^{-43}**	**rs646776**	**0.895**	**3.18x10^{-43}**
			**Liver (ScanDB)**	***PSRC1***	**rs646776**	**0.895**	**2.92x10^{-37}**	**rs646776**	**0.895**	**2.92x10^{-37}**
			**Liver (ScanDB)**	***CELSR2***	**rs646776**	**0.895**	**4.48x10^{-24}**	**rs646776**	**0.895**	**4.48x10^{-24}**
			**Liver(Greenawalt) [PMID 21602305]**	***SORT1***	**rs646776**	**0.895**	**5.20x10^{-88}**	**rs646776**	**0.895**	**5.20x10^{-88}**
			**Liver(Greenawalt) [PMID 21602305]**	***PSRC1***	**rs646776**	**0.895**	**3.05x10^{-86}**	**rs646776**	**0.895**	**3.05x10^{-86}**
			**Liver(Greenawalt) [PMID 21602305]**	***CELSR2***	**rs646776**	**0.895**	**6.27x10^{-68}**	**rs646776**	**0.895**	**6.27x10^{-68}**
			**Liver(Schroder) [PMID 22006096]**	***SORT1***	**rs646776**	**0.895**	**2.14x10^{-27}**	**rs646776**	**0.895**	**2.14x10^{-27}**
			**Liver(Schroder) [PMID 22006096]**	***CELSR2***	**rs646776**	**0.895**	**3.66x10^{-22}**	**rs646776**	**0.895**	**3.66x10^{-22}**
			**Liver(Schroder) [PMID 22006096]**	***PSRC1***	**rs646776**	**0.895**	**8.72x10^{-17}**	**rs646776**	**0.895**	**8.72x10^{-17}**
			**Liver(UChicago) [PMID 21637794]**	***CELSR2***	**rs12740374**	**0.895**	**<1x10^{-16}**	**rs12740374**	**0.895**	**<1e^{-16}**
			**Liver(UChicago) [PMID 21637794]**	***SORT1***	**rs12740374**	**0.895**	**<1x10^{-16}**	**rs12740374**	**0.895**	**<1e^{-16}**
			**Liver(UWash) [PMID 21637794]**	***SORT1***	**rs12740374**	**0.895**	**2.86x10^{-22}**	**rs12740374**	**0.895**	**2.86x10^{-22}**
			**Liver(UWash) [PMID 21637794]**	***CELSR2***	**rs12740374**	**0.895**	**5.31x10^{-11}**	**rs12740374**	**0.895**	**5.31x10^{-11}**
			**Lymph [PMID 17873875]**	***PSRC1***	**rs646776**	**0.895**	**2.10x10^{-08}**	**rs646776**	**0.895**	**2.10x10^{-08}**
			**Monocytes (CD14+) [PMID 22446964]**	***PSRC1***	**rs599839**	**1**	**6.65x10^{-18}**	**rs599839**	**1**	**6.65x10^{-18}**
			**Monocytes [PMID 20502693]**	***PSRC1***	**rs599839**	**1**	**5.30x10^{-55}**	**rs629301**	**0.895**	**2.34x10^{-56}**
			**Muscle_Skeletal [PMID 23715323]**	***CELSR2***	**rs12740374**	**0.895**	**1.40x10^{-08}**	**rs12740374**	**0.895**	**1.40x10^{-08}**
			**Prefrontal cortex (all samples) [PMID 23622250]**	***CELSR2***	**rs646776**	**0.895**	**4.10x10^{-10}**	**rs646776**	**0.895**	**4.10x10^{-10}**
			**Prefrontal cortex (all samples) [PMID 23622250]**	***PSRC1***	**rs646776**	**0.895**	**1.67x10^{-09}**	**rs646776**	**0.895**	**1.67x10^{-09}**
			**Prefrontal cortex (Alzheimer's) [PMID 23622250]**	***PSRC1***	**rs646776**	**0.895**	**7.93x10^{-08}**	**rs646776**	**0.895**	**7.93x10^{-08}**
			**Prefrontal cortex (Alzheimer's) [PMID 23622250]**	***CELSR2***	**rs646776**	**0.895**	**3.24x10^{-05}**	**rs646776**	**0.895**	**3.24x10^{-05}**
			**Prefrontal cortex (Huntington's) [PMID 23622250]**	***CELSR2***			**1.52x10^{-06}**	**rs602633**	**Same SNP**	**1.52x10^{-06}**
			**PrefrontalCortex [PMID 20351726]**	***PSRC1***	**rs599839**	**1**	**2.62x10^{-06}**	**rs599839**	**1**	**2.62x10^{-06}**
			**SchadtLiver [PMID 18462017]**	***SORT1***	**rs599839**	**1**	**1.52x10^{-56}**	**rs599839**	**1**	**1.52x10^{-56}**
			**SchadtLiver [PMID 18462017]**	***CELSR2***	**rs646776**	**0.895**	**3.09x10^{-24}**	**rs646776**	**0.895**	**3.09x10^{-24}**
			**SubCutAdipose(Greenawalt) [PMID 21602305]**	***CELSR2***			**2.93x10^{-08}**	**rs602633**	**Same SNP**	**2.93x10^{-08}**

			**Visual cortex (all samples) [PMID 23622250]**	***PSRC1***	**rs646776**	**0.895**	**7.66x10^{-11}**	**rs646776**	**0.895**	**7.66x10^{-11}**
			**Visual cortex (Alzheimer's) [PMID 23622250]**	***PSRC1***	**rs646776**	**0.895**	**1.44x10^{-09}**	**rs646776**	**0.895**	**1.44x10^{-09}**
			**Whole blood (Battle) [PMID 24092820]**	***PSRC1***	**rs599839**	**1**	**4.93x10^{-87}**	**rs599839**	**1**	**4.93x10^{-87}**
			**Whole blood (Schramm et al.) [PMID 24740359]**	***PSRC1***	**rs599839**	**1**	**1.23x10^{-24}**	**rs599839**	**1**	**1.23x10^{-24}**
			**Whole blood (Wright, n=4,647) [PMID 24728292]**	***CELSR2***	**rs629301**	**0.895**	**6.73x10^{-18}**	**rs629301**	**0.895**	**6.73x10^{-18}**
9p21	**rs10757274**	***ANRIL***	**SubCutAdipose(Greenawalt) [PMID 21602305]**	***CDKN2B***	**rs1537370**	**0.901**	**1.48x10^{-04}**	**rs1537370**	**Same SNP**	**1.48x10^{-04}**
			**Omental adipose [PMID 21602305]**	***CDKN2B***	**rs2383207**	**0.846**	**3.10x10^{-07}**	**rs2383207**	**Same SNP**	**3.10x10^{-07}**
**9q33.2**	**rs10985349**	***DAB2IP***	**CD14+ monocytes (2h LPS stimulated) [PMID 24604202]**	***GGTA1***			**6.64x10^{-04}**	**rs10985349**	**Same SNP**	**6.64x10^{-04}**
**20q13.12**	rs3827066	**Near PCIF1/*MMP9*/*ZNF335***	Bcells (CD19+) [PMID 22446964]	*PLTP*			2.98x10^{-09}	rs394643	0.229	9.89x10^{-40}
			**CD14+ monocytes (24h LPS stimulated) [PMID 24604202]**	***PLTP***			**2.46x10^{-11}**	**rs3827066**	**Same SNP**	**2.46x10^{-11}**
			CD14+ monocytes (24h LPS stimulated) [PMID 24604202]	*DNTTIP1*			8.41x10^{-09}	rs2664529	0.108	1.02x10^{-63}
			CD14+ monocytes (2h LPS stimulated) [PMID 24604202]	*PLTP*			3.89x10^{-11}	rs3843763	0.506	2.46x10^{-13}
			CD14+ monocytes (2h LPS stimulated) [PMID 24604202]	*DNTTIP1*			9.56x10^{-10}	rs2664529	0.108	1.60x10^{-63}
			**CD14+ monocytes (IFNg stimulated) [PMID 24604202]**	***PLTP***			**2.20x10^{-43}**	**rs3827066**	**Same SNP**	**2.20x10^{-43}**
			CD14+ monocytes (IFNg stimulated) [PMID 24604202]	*DNTTIP1*			2.68x10^{-10}	rs6032531	0.148	8.85x10^{-76}
			**CD14+ monocytes (IFNg stimulated) [PMID 24604202]**	***PLTP***			**8.25x10^{-07}**	**rs3827066**	**Same SNP**	**2.20x10^{-43}**
			CD14+ monocytes (untreated) [PMID 24604202]	*DNTTIP1*			3.69x10^{-09}	rs6032531	0.148	1.11x10^{-49}
			CD14+ monocytes (untreated) [PMID 24604202]	*CD40*			4.03x10^{-04}	rs745307	0.086	4.24x10^{-88}
			LCL (MuTHER) [PMID 22941192]	*PLTP*			1.20x10^{-06}	rs441346	0.214	1.64x10^{-37}
			**Liver(UChicago) [PMID 21637794]**	***NEURL2***			**2.29x10^{-06}**	**rs3827066**	**Same SNP**	**2.29x10^{-06}**
			Liver(UChicago) [PMID 21637794]	*C20orf165*	rs7270354	1	1.22x10^{-03}	rs7270354	1	1.22x10^{-03}
			**Liver(UWash) [PMID 21637794]**	***NEURL2***			**1.02x10^{-03}**	**rs3827066**	**Same SNP**	**1.02x10^{-03}**
			Peripheral artery plaque [PMID 24973796]	*NEURL2*	rs7270354	1	2.51x10^{-08}	rs7270354	1	2.51x10^{-08}
			Skin (MuTHER) [PMID 22941192]	*WFDC3*			2.60x10^{-12}	rs2664529	0.108	5.47x10^{-74}
			Subc adipose (MuTHER) [PMID 22941192]	*PLTP*			6.67x10^{-11}	rs6104410	0.486	2.62x10^{-11}
			**Subc adipose (MuTHER) [PMID 22941192]**	***NEURL2***			**9.51x10^{-09}**	**rs3827066**	**Same SNP**	**9.51x10^{-09}**
			Subc adipose (MuTHER) [PMID 22941192]	*WFDC3*			1.26x10^{-05}	rs6032544	0.11	5.99x10^{-36}
			Whole blood (CHARGE) [PMID 24013639]	*TNNC2*			3.98x10^{-19}	rs6104350	0.11	3.33x10^{-63}
			Whole blood (CHARGE) [PMID 24013639]	*DNTTIP1*			2.52x10^{-14}	rs6104350	0.11	7.89x10^{-71}

Online Table XX: eQTL from the Advanced Study of Aortic Pathology[181].

Chr	SNP	Position	Imputed (quality score)	Genes	Aortic Adventitia (Effect, P, Quartile)	Aortic Media (Effect, P, Quartile)	Heart (Effect, P, Quartile)	Liver (Effect, P, Quartile)	LIMA (Effect, P, Quartile)
1	rs1795061	214409280	Yes (0.7908)	*PROX1*	0.0264, 0.764, 2	-0.008, 0.82, 1	-0.0534, 0.449, 4	0.0132, 0.801, 4	0.0565, 0.234, 1
				SMYD2	0.0597, 0.237, 2	-0.0871, 0.0694, 2	0.0254, 0.643, 4	-0.0408, 0.285, 3	-0.0632, 0.302, 2
				PTPN14	-0.071, 0.116, 4	-0.0158, 0.736, 4	0.024, 0.663, 3	0.0299, 0.251, 2	0.00447, 0.933, 4
1	rs4129267	154426264		*UBAP2L*	0.00819, 0.814, 4	-0.000356, 0.993, 4	0.0361, 0.579, 4	0.0467, 0.106, 4	0.046, 0.3, 4
				HAX1	**0.115, 0.0484, 3**	-0.0725, 0.223, 4	0.0377, 0.547, 4	0.0398, 0.291, 4	-0.0667, 0.253, 4
				RNU6-239P	nd	nd	nd	nd	nd
				RNU6-121P	nd	nd	nd	nd	nd
				AQP10	-0.00947, 0.769, 1	-0.0213, 0.494, 1	-0.00645, 0.832, 2	-0.00193, 0.931, 1	0.0217, 0.579, 1
				ATP8B2	0.0769, 0.161, 4	0.0443, 0.32, 4	-0.0216, 0.588, 3	0.0223, 0.446, 3	0.08, 0.161, 4
				IL6R	-0.0261, 0.55, 2	-0.0277, 0.45, 2	-0.028, 0.294, 2	0.0292, 0.49, 4	-0.0128, 0.698, 2
				PSMD8P1	nd	nd	nd	nd	nd
				SHE	nd	nd	nd	nd	nd
				TDRD10	-0.0646, 0.146, 2	-0.0249, 0.465, 2	-0.0246, 0.49, 2	-0.00838, 0.779, 2	0.0332, 0.429, 2
				UBE2Q1	**0.0798, 0.0377, 4**	-0.0194, 0.636, 4	-0.0343, 0.483, 4	**0.0769, 0.0323, 4**	0.0562, 0.198, 4
				UBE2Q1-AS1	nd	nd	nd	nd	nd
				CHRNB2	0.0103, 0.758, 2	0.0322, 0.367, 2	-0.0525, 0.17, 2	0.00361, 0.898, 2	0.00785, 0.856, 2
				ADAR	0.0195, 0.674, 4	0.000942, 0.986, 4	0.0382, 0.559, 4	0.055, 0.107, 4	0.0846, 0.146, 4
1	rs602633	109821511	Yes (0.51863)	*TMEM167B*	nd	nd	nd	nd	nd
				SCARNA2	nd	nd	nd	nd	nd
				C1orf194	nd	nd	nd	nd	nd
				KIAA1324	0.0254, 0.3, 1	0.00596, 0.762, 1	0.0279, 0.189, 1	-0.0172, 0.26, 1	-0.00434, 0.868, 1
				SARS	-0.0594, 0.2, 4	-0.0245, 0.546, 4	-0.0825, 0.2, 4	0.0736, 0.0725, 4	0.0937, 0.087, 4
				CELSR2	0.00668, 0.806, 3	-0.0101, 0.643, 3	0.0198, 0.46, 3	**0.0522, 0.0139, 3**	-0.0384, 0.208, 3
				PSRC1	0.00324, 0.91, 2	-0.0272, 0.36, 2	0.0288, 0.343, 2	**0.281, 9.67×10^{-13}, 2**	0.0057, 0.877, 2
				MYBPHL	nd	nd	nd	Nd	nd
				SORT1	-0.145, 0.135, 4	-0.0409, 0.532, 4	**-0.203, 0.009, 4**	**1.25, 2.35×10^{-24}, 3**	0.0304, 0.648, 4
				PSMA5	-0.137, 0.0753, 3	-0.0474, 0.489, 3	-0.145, 0.0884, 4	0.102, 0.0705, 4	0.131, 0.191, 3
				SYPL2	**-0.0904, 0.0412, 1**	-0.0385, 0.461, 2	**-0.211, 0.0312, 2**	**0.0977, 0.0336, 2**	-0.0657, 0.28, 2
9	rs10757274	22096055	Yes (0.78299)	*MTAP*	0.0276, 0.416, 3	0.0231, 0.403, 3	0.0554, 0.142, 3	-0.0111, 0.668, 3	0.0137, 0.702, 3
				ERVFRD-3	nd	nd	nd	nd	nd
				CDKN2A-AS1	nd	nd	nd	nd	nd
				CDKN2A	0.0379, 0.215, 2	0.0337, 0.296, 2	-0.0227, 0.449, 2	-0.00872, 0.68, 2	0.022, 0.515, 2
				ANRIL	nd	nd	nd	nd	nd
				CDKN2B	0.0101, 0.728, 3	-0.0237, 0.405, 3	0.000812, 0.979, 3	0.0047, 0.82, 3	0.0369, 0.325, 3
				UBA52P6	nd	nd	nd	nd	nd
9	rs10985349	124425243	Yes (0.58069)	*GGTA1P*	nd	nd	nd	nd	nd
				RN7SL187P	nd	nd	nd	nd	nd
				HMGB1P37	nd	nd	nd	nd	nd
				DAB2IP	0.0586, 0.13, 3	0.0653, 0.11, 3	-0.0288, 0.571, 3	-0.0159, 0.547, 3	-0.0644, 0.0833, 3
				TTLL11	0.0213, 0.64, 3	0.0515, 0.163, 3	**-0.0772, 0.0168, 3**	0.0161, 0.445, 3	**-0.0992, 0.00289, 4**
13	rs9316871	22861921		*LINC00540*	nd	nd	nd	nd	nd
				NME1P1	nd	nd	nd	nd	nd
				MTND3P1	nd	nd	nd	nd	nd
19	rs6511720	11202306		*CARM1*	0.00983, 0.861, 3	-0.0297, 0.577, 3	-0.00125, 0.987, 3	-0.0163, 0.662, 3	0.0162, 0.744, 3
				YIPF2	0.00883, 0.881, 3	-0.00788, 0.863, 3	-0.0105, 0.871, 3	-0.0461, 0.237, 3	0.0308, 0.624, 3
				C19orf52	-0.0462, 0.458, 3	0.0269, 0.58, 3	-0.023, 0.717, 3	-0.0214, 0.559, 3	-0.0201, 0.772, 3
				SMARCA4	0.0288, 0.569, 3	**-0.105, 0.0407, 3**	-0.018, 0.787, 3	-0.0268, 0.393, 3	-0.0647, 0.323, 3
				LDLR	0.164, 0.118, 3	0.19, 0.112, 3	-0.0297, 0.634, 3	0.0024, 0.982, 4	0.221, 0.177, 3
				MIR6886	nd	nd	nd	nd	nd
				SPC24	-0.0229, 0.651, 1	0.0159, 0.721, 2	0.0378, 0.507, 2	-0.0246, 0.459, 1	0.0153, 0.781, 2
				KANK2	-0.0233, 0.851, 4	-0.00958, 0.911, 4	0.0425, 0.71, 4	-0.0695, 0.141, 3	-0.0716, 0.395, 4
				DOCK6	-0.018, 0.749, 3	-0.0289, 0.469, 3	-0.0386, 0.526, 3	-0.0229, 0.523, 3	-0.0288, 0.513, 3
				C19orf80	nd	nd	nd	nd	nd
20	rs3827066	44586023	Yes (0.6275)	*WFDC3*	0.0626, 0.15, 2	**0.0772, 0.0433, 2**	-0.00286, 0.961, 2	-0.0196, 0.542, 2	0.0407, 0.375, 2
				RNU6ATAC38P	nd	nd	nd	nd	nd
				DNTTIP1	-0.0666, 0.132, 3	-0.0711, 0.0606, 3	-0.0573, 0.316, 3	**-0.0781, 0.00627, 3**	**-0.0715, 0.0459, 3**
				UBE2C	0.0147, 0.729, 2	0.0206, 0.616, 2	-0.0565, 0.219, 2	0.0164, 0.538, 2	**0.0859, 0.025, 2**
				TNNC2	-0.0322, 0.621, 2	0.059, 0.304, 2	0.0675, 0.431, 2	0.007, 0.872, 2	0.121, 0.086, 2
				SNX21	-0.0545, 0.228, 3	0.00543, 0.875, 3	-0.0359, 0.544, 3	-0.00772, 0.773, 3	0.0266, 0.539, 3
				ACOT8	-0.00969, 0.816, 3	0.0458, 0.17, 3	-0.1, 0.277, 3	0.0303, 0.313, 3	-0.024, 0.53, 3
				ZSWIM3	0.0582, 0.209, 1	0.0493, 0.187, 1	-0.0382, 0.563, 1	0.0258, 0.402, 1	-0.00447, 0.922, 1
				ZSWIM1	-0.0458, 0.427, 2	-0.0743, 0.123, 2	-0.0861, 0.374, 2	-0.0159, 0.655, 2	0.0164, 0.78, 2
				SPATA25	nd	nd	nd	nd	nd
				NEURL2	nd	nd	nd	nd	nd
				CTSA	0.0231, 0.616, 4	-0.0716, 0.321, 4	0.0481, 0.487, 4	**0.105, 0.0111, 4**	-0.141, 0.101, 4
				PLTP	**0.513, 0.0118, 4**	-0.00838, 0.958, 4	0.0306, 0.832, 4	0.0375, 0.587, 4	-0.0865, 0.561, 3
				PCIF1	-0.0435, 0.361, 3	-0.0633, 0.0975, 3	-0.000238, 0.998, 3	-0.0277, 0.278, 3	-0.042, 0.389, 3
				ZNF335	-0.0467, 0.107, 3	0.0401, 0.123, 3	0.0213, 0.631, 3	0.0105, 0.608, 3	-0.0174, 0.579, 3
				FTLP1	nd	nd	nd	nd	nd
				MMP9	0.107, 0.333, 2	0.168, 0.0796, 2	0.0236, 0.623, 2	0.0107, 0.79, 2	0.0262, 0.566, 2
				SLC12A5	-0.0409, 0.175, 2	-0.00016, 0.995, 2	0.0189, 0.654, 2	-0.00367, 0.866, 1	-0.0206, 0.544, 2
				NCOA5	-0.00203, 0.962, 3	-0.0311, 0.449, 4	-0.0941, 0.29, 3	-0.0339, 0.424, 3	-0.0258, 0.597, 4
				RPL13P2	nd	nd	nd	nd	nd
				CD40	-0.0309, 0.531, 2	-0.0095, 0.813, 2	-0.0416, 0.391, 2	0.0104, 0.681, 2	0.0118, 0.755, 2
21	rs2836411	39819830		*KCNJ15*	-0.00282, 0.958, 2	-0.0161, 0.634, 1	-0.00692, 0.809, 1	-0.0181, 0.635, 2	-0.0407, 0.236, 1
				LINC01423	nd	nd	nd	nd	nd
				ERG	-0.0351, 0.636, 4	-0.0324, 0.573, 4	0.0247, 0.648, 3	0.0276, 0.309, 3	-0.106, 0.0762, 4
				SNRPGP13	nd	nd	nd	nd	nd

Online Figure III: Significant eQTL plots for data from ASAP study. Each column of plots represents data for the SNP and tissue stated at the bottom of each column. The gene for which expression has been assessed in each plot is shown to the left of each plot.

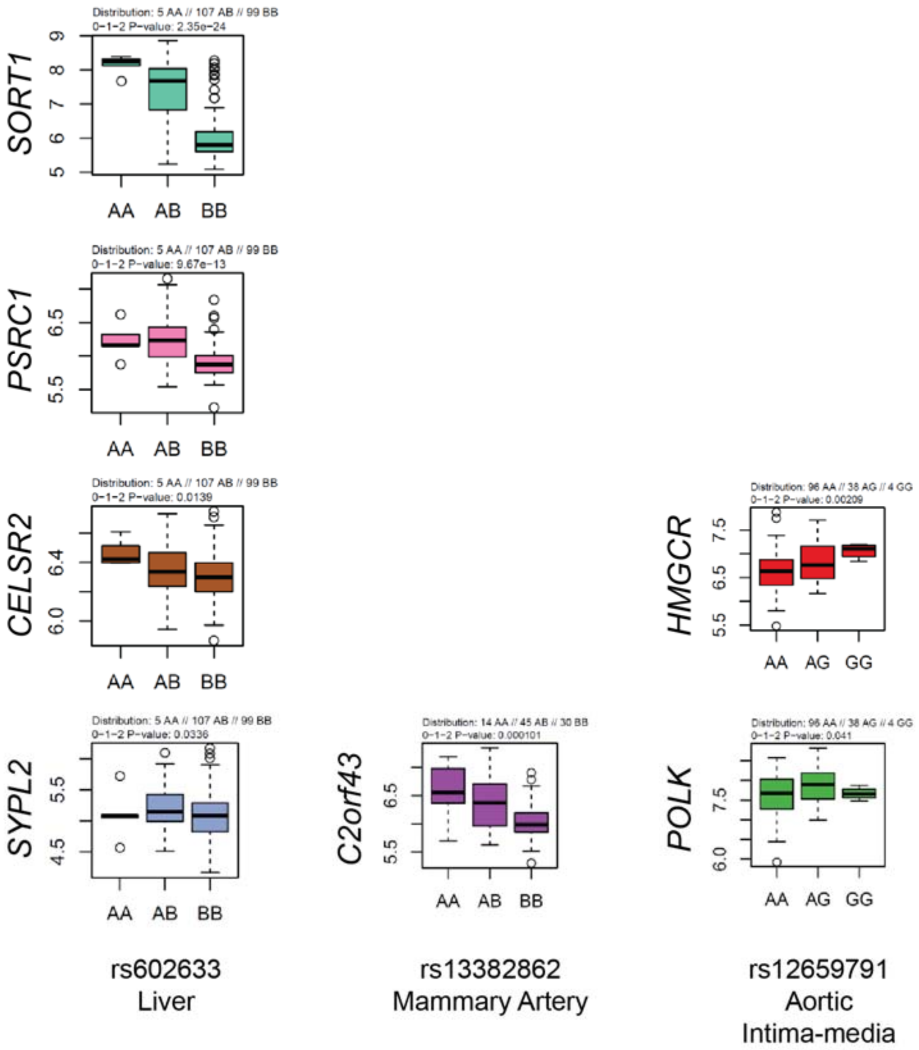

Online Table XXI: eQTL results based on RNA seq data

Chr	Position	SNP	Locus	Gene(s)	Tissue	gene for RNA-Seq	index SNP	index SNP beta	index SNP P	Lead SNP in region	Lead SNP beta	Lead SNP P	index SNP effect independent
1	109821511	rs602633	1p13.3	CELSR2/*SORT1*	Blood	*PSRC1*	rs602633	0.640465606	4.49x10^{-17}	rs629301	0.61412198	1.03x10^{-15}	Yes
					LIV	*PSRC1*	rs602633	-1.292449889	9.43x10^{-10}8	rs7528419	1.26797503	4.33x10^{-103}	Yes
					LIV	*SARS*	rs602633	0.495537791	2.37x10^{-12}	rs1277930	0.49333558	2.97x10^{-12}	Yes
					LIV	*SORT1*	rs602633	-1.365654087	2.98x10^{-13}0	rs7528419	1.33525433	1.79x10^{-122}	Yes
					LIV	*CELSR2*	rs602633	1.298741331	1.79x10^{-10}9	rs629301	1.27447629	8.75x10^{-105}	Yes
1	154426264	rs4129267	1q21.3	*IL6R*	MAM	*IL6R*	rs4129267	0.652344491	1.97x10^{-26}	rs7518199	0.63498045	3.78x10^{-24}	Yes
13	22861921	rs9316871	13q12.11	*LINC00540*	MAM	*FGF9*	rs9316871	-0.699526462	1.76x10^{-23}	rs9506822	-0.7027035	2.44x10^{-23}	Yes
20	44586023	rs3827066	20q13.12	Near PCIF1/*MMP9*/*ZNF335*	AOR	*PLTP*	rs3827066	-0.756619944	1.65x10^{-21}	rs7267295	-0.7193854	5.40x10^{-18}	Yes
					SF	*PLTP*	rs3827066	-0.905646216	4.93x10^{-29}	rs7270354	-0.8474133	2.10x10^{-26}	Yes
					SKLM	*PLTP*	rs3827066	-0.484882963	2.76x10^{-09}	rs7267295	-0.4860579	1.40x10^{-08}	Yes
					VAF	*PLTP*	rs3827066	-0.610415837	1.39x10^{-12}	rs8124182	-0.5513566	1.48x10^{-10}	Yes
21	39819830	rs2836411	21q22.2	*ERG*	MAM	*ERG*	rs386574671	0.271607451	1.88x10^{-05}	rs386574671	0.27160745	1.88x10^{-05}	Yes

Online Table XXII: Peripheral blood monocyte eQTLs

Chr	Position	SNP	Locus	Gene(s)	Probe_ID	ILMN_Gene	beta	beta_se	P	FDR	Cell type
1	109821511	rs602633	1p13.3	CELSR2/*SORT1*	ILMN_1671843	*PSRC1*	-0.2202	0.0195	7.91x10^{-27}	1.08x10^{-23}	Macrophage
					ILMN_2315964	*PSRC1*	-0.0763	0.0168	6.32x10^{-06}	8.65x10^{-04}	Macrophage
					ILMN_1671843	*PSRC1*	-0.1512	0.0121	1.96x10^{-32}	2.33x10^{-29}	Monocyte
					ILMN_2315964	*PSRC1*	-0.1261	0.0158	4.72x10^{-15}	1.83x10^{-12}	Monocyte
					ILMN_1711208	*CELSR2*	-0.0696	0.0139	7.41x10^{-07}	1.23x10^{-04}	Macrophage
					ILMN_1707077	*SORT1*	-0.0752	0.0183	4.38x10^{-05}	4.95x10^{-03}	Macrophage
					ILMN_1671843	*PSRC1*	-0.2202	0.0195	7.91x10^{-27}	1.08x10^{-23}	Macrophage
					ILMN_1711208	*CELSR2*	-0.0696	0.0139	7.41x10^{-07}	1.23x10^{-04}	Macrophage
					ILMN_2315964	*PSRC1*	-0.0763	0.0168	6.32x10^{-06}	8.65x10^{-04}	Macrophage
					ILMN_1707077	*SORT1*	-0.0752	0.0183	4.38x10^{-05}	4.95x10^{-03}	Macrophage
					ILMN_1671843	*PSRC1*	-0.1512	0.0121	1.96x10^{-32}	2.33x10^{-29}	Monocyte
					ILMN_2315964	*PSRC1*	-0.1261	0.0158	4.72x10^{-15}	1.83x10^{-12}	Monocyte
20	44586023	rs3827066	20q13.12	Near *PCIF1/MMP9/ZNF335*	ILMN_1777113	*NEURL2*	-0.1058	0.0255	3.86x10^{-05}	3.11x10^{-03}	Macrophage
					ILMN_1773389	*PLTP*	-0.3127	0.0796	9.57x10^{-05}	6.86x10^{-03}	Macrophage
					ILMN_1711748	*PLTP*	-0.2266	0.0587	1.27x10^{-04}	8.82x10^{-03}	Macrophage
					ILMN_2367818	*CD40*	0.0828	0.0245	7.75x10^{-04}	4.08x10^{-02}	Macrophage
					ILMN_1691117	*DNTTIP1*	0.0741	0.0109	1.95x10^{-11}	3.13x10^{-09}	Monocyte
					ILMN_2367818	*CD40*	0.0752	0.0176	2.20x10^{-05}	1.45x10^{-03}	Monocyte
					ILMN_1779257	*CD40*	0.1136	0.0317	3.63x10^{-04}	1.74x10^{-02}	Monocyte
21	39819830	rs2836411	21q22.2	*ERG*	ILMN_1757074	*GNG10*	-0.0626	0.0138	6.84x10^{-06}	5.28x10^{-01}	Monocyte

VALIDATION OF GWAS3D RESULTS USING mRNA EXPRESSION DATA FOR AAA AND CONTROL AORTA

To determine the potential utility of the GWAS3D chromatin state analysis to identify trans interactions, a validation analysis was performed comparing mRNA expression of putative genes in abdominal aortic tissue. Relative mRNA expression profiles of candidate genes, indicated by either SNP proximity (cis-acting regulatory variant) or GWAS3D predicted distal interaction, was derived using the Biros *et al* 2015 (GSE57691) dataset (**Online Table XXIII)**. The composition and analysis of this dataset has been previously described[107]. All genes at AAA loci (Table 1) were included in this analysis. Case (49 AAA, including 29 large and 20 small) and control (10 organ donor) abdominal aortic samples were compared using data generated from the Illumina HumanHT-12 V4.0 expression beadchip (GPL10558).

Several of the GWAS3D predicted distal gene interactions appeared to have differential case/ control gene expression (**Online Table XXIII and Online Figure IV)**. For example, not only was the mRNA expression of *SORT1* (which is within the locus suggested by rs602633) significantly different between cases and controls, but predicted distal interactions in *BCAR3* and *NOTCH2* also had altered expression. The predicted distal interaction between rs4129267 (*IL6R* locus) and *TDRD10* also appeared concordant with an observed differential gene expression profile. The intergenic SNP rs9316871 (closest gene *LINC0540*) had a predicted interaction with *FGF9* which also had increased expression in AAA versus control aortic tissue.

It should be noted that absence of differential gene expression in this analysis does not specifically preclude a role in AAA pathogenesis. Many genes will have temporal expression and may, for example, only be differentially expressed in specific phases of the pathology. In addition, other genes may have significantly altered expression in other tissues (such as the liver, kidney or circulating leukocytes) the results of which may have indirect effects on the aortic wall. Nevertheless, these results appear to, at least in part, validate the potential utility of chromatin state-based analysis to identify functional mechanisms underlying SNP associations.

Online Table XXIII: AAA tissue mRNA expression for genes in close proximity to validated SNPs or having predicted distal gene interactions based on GWAS3D analysis.

Gene selection criteria	Gene	Locus	Entrez Gene ID	mRNA p-value	AAA mRNA expression
**metaGWAS SNP proximity**	**ANRIL**	**9p21.3**	**1030**	**0.0025**	**increased**
**GWAS3D predicted distal interaction (SORT1)**	**BCAR3**	**1p22.1**	**8412**	**$1.8x10^{-4}$**	**decreased**
**metaGWAS SNP proximity**	CDKN2A	9p21.3	1029	0.217	
**metaGWAS SNP proximity**	CDKN2BAS1	9p21.3	100048912	0.266	
**metaGWAS SNP proximity**	CELSR2	1p13.3	1952	0.479	
**metaGWAS SNP proximity**	CHRNB2	1q21.3	1141	0.829	
**metaGWAS SNP proximity**	CTSA	20q13.12	5476	0.174	
**metaGWAS SNP proximity**	DAB2IP	9q33.1	153090	0.213	
**metaGWAS SNP proximity**	ERG	21q21.3	2078	0.095	
**GWAS3D predicted distal interaction (LINC00540)**	**FGF9**	**13q11**	**2254**	**0.002**	**increased**
**metaGWAS SNP proximity**	IL6R	1q21	3570	0.087	
**metaGWAS SNP proximity**	KANK2	19p13.2	25959	0.132	
**metaGWAS SNP proximity**	LDLR	19p13.2	3949	0.197	
**metaGWAS SNP proximity**	**LRP1**	**12q13.3**	**4035**	**0.0084**	**decreased**
**metaGWAS SNP proximity**	MMP9	20q13.12	4318	0.132	
**metaGWAS SNP proximity**	MYBPHL	1p13.3	343263	0.897	
**metaGWAS SNP proximity**	**NAB2**	**12q13.3**	**4665**	**$1.1x10^{-5}$**	**decreased**
**metaGWAS SNP proximity**	NEURL2	20q13.12	140825	0.576	
**GWAS3D predicted distal interaction (LDLR)**	NEUROG1	5q23	4762	0.138	
**GWAS3D predicted distal interaction (SORT1)**	**NOTCH2**	**1p12**	**4853**	**$4.6x10^{-7}$**	**increased**
**metaGWAS SNP proximity**	PCIF1	20q13.12	63935	0.968	
**metaGWAS SNP proximity**	**PLTP**	**20q13.12**	**5360**	**0.011**	**increased**
**metaGWAS SNP proximity**	PSRC1	1p13.3	84722	0.440	
**metaGWAS SNP proximity**	SARS	1p13.3	6301	0.095	
**GWAS3D predicted distal interaction (LDLR)**	SHANK1	19p13.3	50944	0.567	
**metaGWAS SNP proximity**	SHE	1q21.3	126669	0.396	
**metaGWAS SNP proximity**	SLC12A5	20q13.12	57468	0.329	
**metaGWAS SNP proximity**	SMYD2	1q41	56950	0.317	
**metaGWAS SNP proximity**	**SORT1**	**1p13.3**	**6272**	**$1.1x10^{-4}$**	**decreased**
**metaGWAS SNP proximity**	SPC24	19p13.2	147841	0.211	
**metaGWAS SNP proximity**	STAT6	**12q13.3**	6778	0.423	
**GWAS3D predicted distal interaction (IL6R)**	**TDRD10**	**1q21.3**	**126668**	**0.006**	**increased**
**GWAS3D predicted distal interaction (IL6R)**	TYW1	7q11.21	55253	0.320	
**metaGWAS SNP proximity**	UBE2Q1	1q21.3	55585	0.157	
**GWAS3D predicted distal interaction (CDKN2B-AS1)**	**UBE2W**	**8q21.11**	**55284**	**0.0292**	**increased**
**metaGWAS SNP proximity**	ZNF335	20q13.12	63925	0.205	
**GWAS3D predicted distal interaction (LDLR)**	ZNF440	19p13.2	126070	0.406	
**metaGWAS SNP proximity**	ZSWIM3	20q13.12	140831	0.235	

Online Figure IV: Box and whiskers plots of gene expression in AAA tissue and control tissue for genes in close proximity to validated SNPs or having predicted distal gene interactions based on GWAS3D analysis. Significant differences between AAA and controls are highlighted. Gene expression is log base 2, normalized to the 75th percentile.

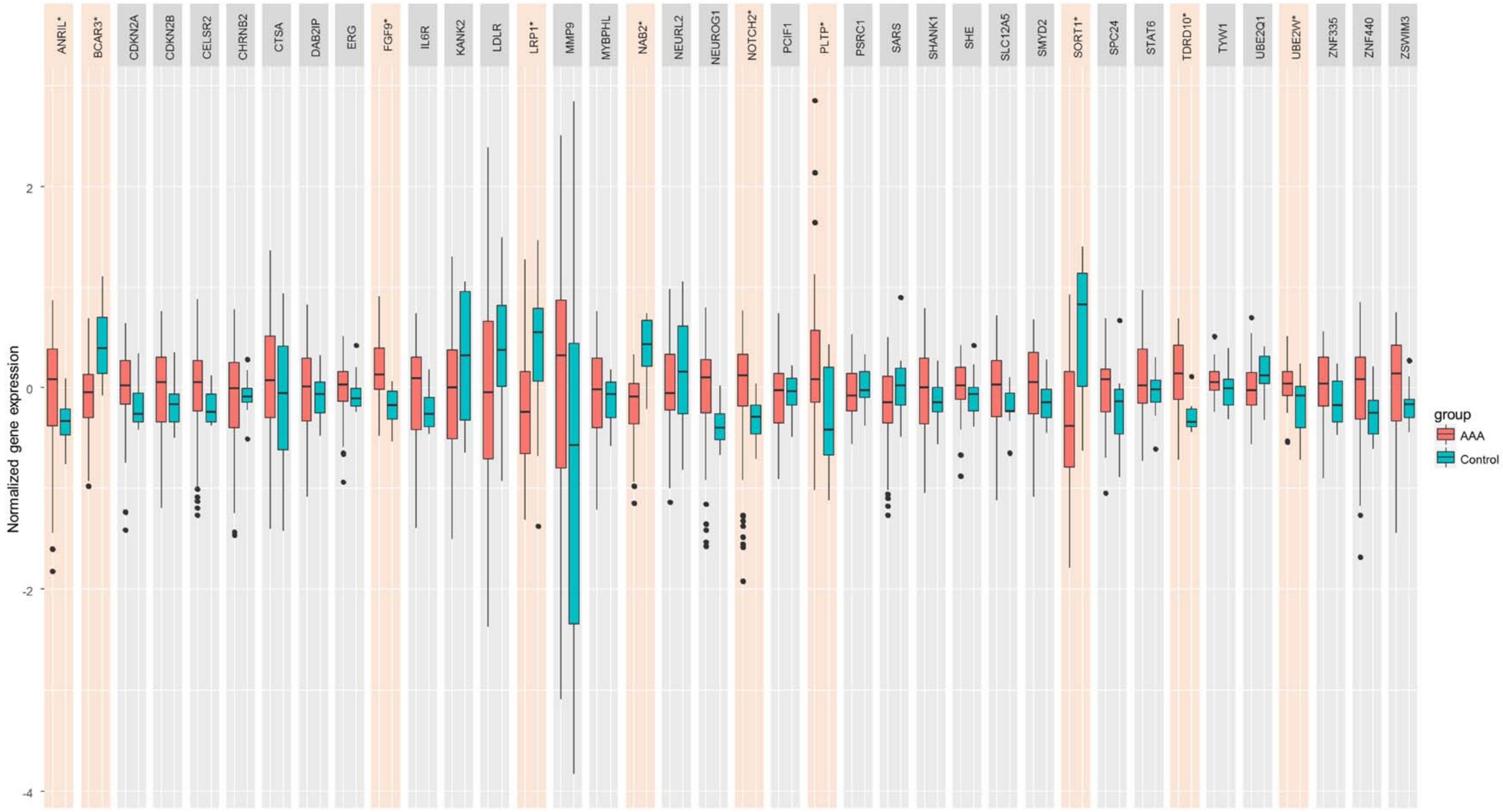

LOOK-UP FOR TRANSCRIPTION FACTOR BINDING SITES IN GENES HARBORING AAA-ASSOCIATED VARIANTS

We previously performed a chromatin-immunoprecipation (ChIP) study using AAA and control aorta tissue for the TFs ELF1, ETS2, RUNX1 and STAT5[185]. These TFs were chosen because they were enriched in genes differentially expressed between AAA and control aorta; ELF1, ETS2, and RUNX1 were identified as relevant to most upregulated genes[186] and STAT5 was a driver for genes in the complement cascade[187].

The TF binding data were obtained from tables published in a paper by Pahl et al.[185], which describes ChIP-chip for TFs ELF1, ETS2, RUNX1 and STAT5 using human aortic tissue (AAA and control aorta). We performed a lookup in these data for evidence supporting that the genes near the SNPs identified by the meta-GWAS are relevant to AAA pathobiology. Lack of evidence in these data does not preclude involvement in AAA, but presence of evidence is a useful indicator that the gene is likely involved. This is especially useful for genes with little or no annotation in the major databases such as *SMYD2* and *ERG*. The results are summarized in **Online Table XXIV**. Chromatin enriched regions (cher) with binding sites for the TF ETS2 were found in *SMYD2* and *SORT1*. TF STAT5 had binding sites with chers in *CDKN2B-AS1ANRIL*, *ERG* and *DAB2IP*, and TF ELF1 had multiple binding sites in *ERG*. None of the TF binding sites in these genes contained the lead SNPs identified at the AAA risk loci.

Online Table XXIV: ChIP-chip data on human aortic tissue for the genes harbouring AAA-associated SNPs. Genome-wide ChIP-chip data were available on 4 transcription factors: ETS2, ELF1, STAT5 and RUNX1. For details, see Pahl et al. 2015[185]. AAA, aortic tissue from abdominal aortic aneurysm; cher, chromatin enriched region; CTL, control abdominal aorta; TFBS, transcription factor binding site as defined by Transfac®.

SNP	Chr	Position	Gene(s)	Gene Symbol	Cher for TF (tissue source)	TFBS in cher
rs602633	1	109821511	*PSRC1-CELSR2-SORT1*	*CELSR2*	None	
				SORT1	ETS2 (CTL)	ETS2 (1 site)
				PSRC	None	
rs4129267	1	154426264	*IL6R*	*IL6R*	None	
rs1795061	1	214409280	*SMYD2*	*SMYD2*	ETS2 (AAA)	ETS2 (4 sites)
rs10757274	9	22096055	ANRIL	*ANRIL*	STAT5 (CTL)	STAT5 (1 site)
rs10985349	9	124425243	*DAB2IP*	*DAB2IP*	STAT5 (CTL)	STAT5 (1 site)
rs9316871	13	22861921	*LINC00540*	*LINC00540*	None	
rs6511720	19	11202306	*LDLR*	*LDLR*	None	
rs3827066	20	44586023	*PCIF1-ZNF335-MMP9*	*PCIF1*	None	
				MMP9	None	
				ZNF335	None	
rs2836411	21	39819830	*ERG*	*ERG*	STAT5 (AAA)	STAT5 (1 site)
					ELF1 (AAA)	ELF1 (4 sites)
					ELF1 (CTL)	

NETWORK ANALYSIS

We investigated whether most of the loci could be connected into a single network through intermediate nodes and interactions. A network integrating most of the loci would suggest mechanisms by which the loci could act in concert, whether synergistically or antagonistically, to affect the phenotype. The network(s) would also provide hypotheses for future investigation. Potential interactions between molecules encoded by genes harboring AAA-associated SNPs were analyzed using two independent analysis tools: Ingenuity Pathway Analysis® (IPA) tool version 9.0 (Qiagen's Ingenuity Systems, Redwood City, CA, USA; www.ingenuity.com) and ConsensusPathDB (http://cpdb.molgen.mpg.de/CPDB)[188-191]. The analyzed gene set had 14 genes: 2 loci identified by the 9 AAA-associated SNPs included clusters of 3 genes (see **Online Table XIV** for SNP annotations), we also included TNF since recent literature indicated that SMYD2 suppresses IL6 and TNF production[192, 193] and this had been published since the latest database update for each pathway analysis tool used. The gene symbols included in the network analyses were: *CDKN2BAS1, CELSR2, DAB2IP, ERG, IL6R, LDLR, LINC00540, MMP9, PCIF1, PSRC1, SMYD2, SORT1, ZNF335, and TNF.*

The parameters for the IPA were: 1. the Ingenuity Knowledge Base was used as a reference set; 2. both direct and indirect relationships were considered; and 3. only relationships that were either experimentally observed or had predictions with high confidence were considered. The relationships displayed as direct interactions mean that the two molecules make physical contact with each other such as binding or phosphorylation, and those displayed as indirect interactions do not require physical contact between the two molecules, such as signaling events. The IPA network generation algorithm has been described previously[194]. The IPA's Core Analysis generated 2 networks, (1) "cardiovascular disease, cellular movement, developmental disorders" ($P=1x10^{-21}$; 8/12 molecules), and (2) "cell signalling, nucleic acid metabolism, small molecular biochemistry" ($P=1x10^{-7}$; 3/12 molecules). We merged the two networks into an interaction figure (**Online Figure V**). This identified that *ERG, IL6R* and *LDLR* were predicted modifiers of *MMP9*, with a direct interaction between *ERG* and *MMP9*. *SORT* and *LDLR* appear coupled via *ERK* and *LDL*. *IL6R* affects *DAB2IP* which in turn regulates *NFKB*. Several gene products, such as *ANRIL, CELSR2, ZNF335* and *SMYD2* have poorly defined functions at present, and *LINC00540*, a long non-coding RNA expressed in the hippocampus and lacking annotation information, did not belong to either of the 2 networks. The long non-coding RNA ANRIL, our strongest hit in the genome (**Figure 1**), has been reported in numerous studies as a GWAS hotspot and a candidate gene for CAD, intracranial aneurysms, and diverse cardiometabolic disorders[195].

The same gene list was submitted to the ConsensusPathDB web-based tool for generating an inferred network. ConsensusPathDB-human integrates interaction networks in *Homo sapiens* including binary and complex protein-protein, genetic, metabolic, signaling, gene regulatory and drug-target interactions, as well as biochemical pathways. Data currently originate from 32 public resources for interactions and interactions that have been curated from the literature. The interaction data are integrated in a manner to avoid redundancies, resulting in an interaction network containing different types of interactions. When the analysis was carried out, the database contained the following annotations: 158,523 unique physical entities; 458,570 unique interactions (17,098 gene regulation, 261,085 protein interaction, 443 genetic, 21,070 biochemical reactions, and 158,874 drug-target interactions), and 4,593 pathways. ConsensusPathDB infers a network to include proteins or metabolites that are not in the user-supplied input list, but associate two or more nodes (gene/protein/metabolite) on the input list with each other. These nodes are termed intermediate nodes and are ranked according to the significance of association with the input nodes given their overall connectivity in the background network. This is quantified by a z-score calculated for each intermediate node with the binomial

proportions test. The default z-score was used. The network was visualized using CytoScape (version 3.4.0).

Four genes from the input list did not map to known entities in ConsensusPathDB: ANRIL and LINC00540 are long non-coding RNAs and not represented; similarly PCIF1 and ZNF335 are poorly annotated and not currently represented in source databases (**Online Figure VI).** The inferred network generated by ConsensusPathDB is largely similar to that produced by IPA, although it lacks the interaction between SMYD2 and IL6R, and SMYD2 and TNF. The absence of these interactions could be due to the recent elucidation as well as the unknown mechanism by which the SMYD2 suppression of TNF and IL6 occurs. The number of interactions of a node is a function of the true number of interactions as well as how well studied the protein or gene is. In the network (**Online Figure VI**) MMP9 and TNF have a large number of interactions. Interestingly LDLR and SMYD2 both have indirect interactions with MMP9 and TNF through CREBP, and could have synergistic effects on the AAA phenotype. Additionally CREBP has an interaction with NFKB complex and ETS1. Inhibition of NFKB and ETS1 was shown to reduce AAA in a rat model[196] and their promoter binding sites were enriched in the promoters of genes upregulated in human AAA[197].

Online Figure V. Potential interactions between gene products of AAA related genes. This figure shows IPA networks 1 and 2 merged together. Molecules are represented as nodes, and the biological relationship between two nodes as a line. Solid lines represent direct and dashed lines indirect interactions. All lines are supported by at least one literature citation or are from canonical information stored in the Ingenuity Pathways Knowledge Base (Qiagen's Ingenuity Systems). Nodes are displayed using various shapes that represent the functional class of the gene product. Molecules in orange are encoded by genes harboring AAA-associated variants. Red dashed lines indicate new information on SMYD2, IL6 and TNF found in recent literature[192, 193]

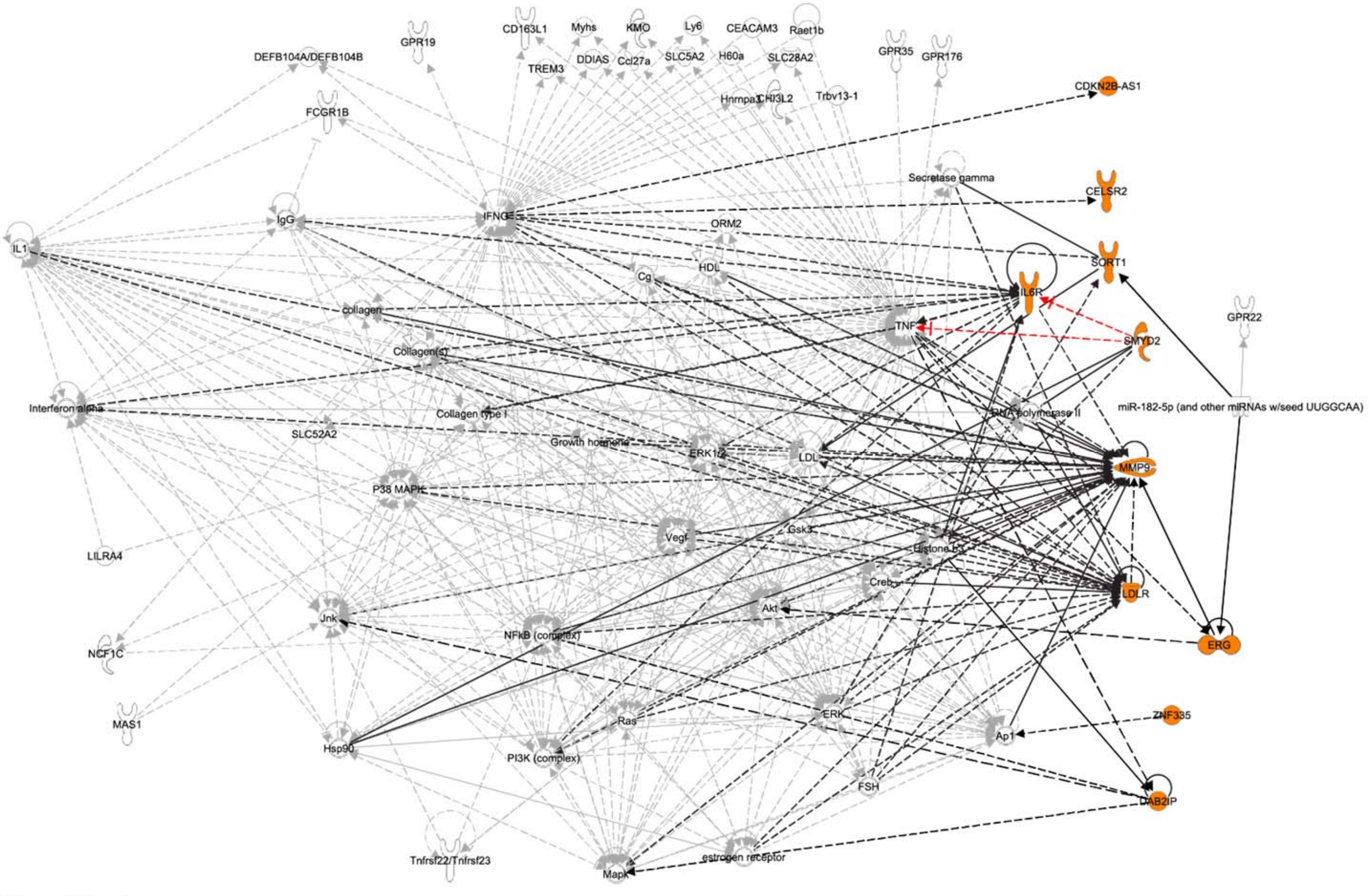

© 2000-2015 QIAGEN. All rights reserved.

Online Figure VI: Induced network generated by ConsensusPathDB from 14 input genes. The network comprises, genes, gene products (proteins), protein complexes, and metabolites, represented as nodes of different colors: pink, protein; light-blue, gene; medium blue, protein complexes; yellow, metabolite; and light green, unknown complex. Node shape indicates whether the node was on the input list: octagons, input list; rhomboids, induced nodes. Interactions are represented as edges, with color indicating interaction type: blue, protein interaction; red, gene regulatory interaction; and green, biochemical interaction.

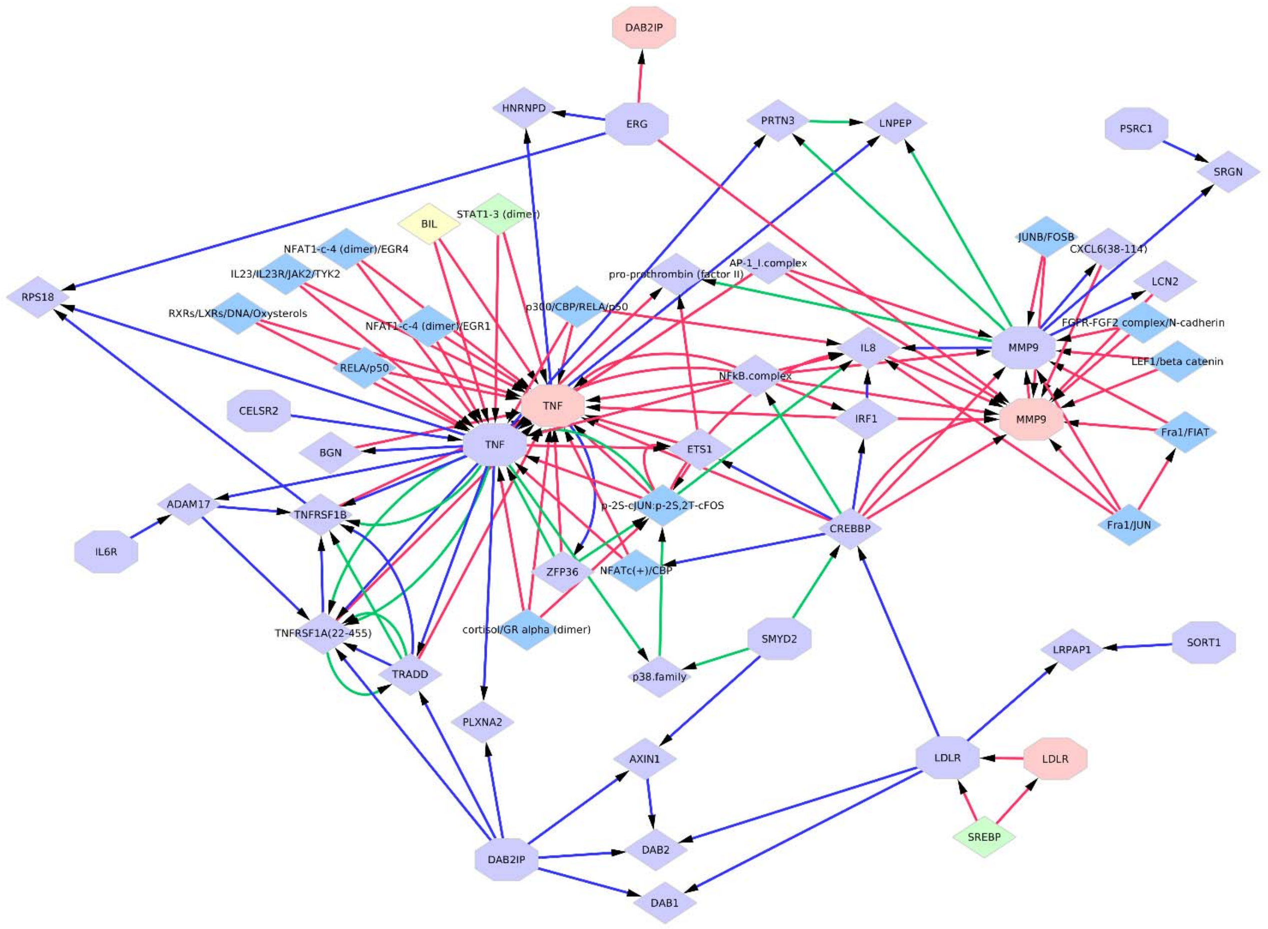

CONSORTIA CONTRIBUTING DATA

List of members of the Cardiogenics consortium

Tony Attwood[1], Stephanie Belz[2], Peter Braund[3], Jessy Brocheton[4], François Cambien[4], Jason Cooper[5], Abi Crisp-Hihn[1], Patrick Diemert (formerly Linsel-Nitschke)[2], Panos Deloukas[6], Jeanette Eardman[2], Nicola Foad[1], Tiphaine Godefroy[4], Alison H Goodall[3,11], Jay Gracey[3], Emma Gray[6],, Rhian Gwilliams[6], Susanne Heimerl[7], Christian Hengstenberg[7], Jennifer Jolley[1], Unni Krishnan[3], Heather Lloyd-Jones[1], Ulrika Liljedahl[8], Ingrid Lugauer[7], Per Lundmark[8], Seraya Maouche[2,4], Jasbir S Moore[3], Gilles Montalescot[4], David Muir[1], Elizabeth Murray[1], Chris P Nelson[3], Jessica Neudert[9], David Niblett[6], Karen O'Leary[1], Willem H Ouwehand[1,6], Helen Pollard[3], Carole Proust[4], Angela Rankin$_1$, Augusto Rendon[12], Catherine M Rice[6], Hendrik B Sager[2], Nilesh J Samani[3,11], Jennifer Sambrook[1], Gerd Schmitz[10], Michael Scholz[9], Laura Schroeder[2], Heribert Schunkert[2], Jonathan Stephens[1], Ann-Christine Syvannen[8], Stefanie Tennstedt (formerly Gulde)[2], Chris Wallace[5].

[1]*Department of Haematology, University of Cambridge, Long Road, Cambridge, CB2 2PT, UK and National Health Service Blood and Transplant, Cambridge Centre, Long Road, Cambridge, CB2 2PT, UK;*

[2]*Medizinische Klinik 2, Universität zu Lübeck, Lübeck Germany*

[3]*Department of Cardiovascular Sciences, University of Leicester, Glenfield Hospital, Groby Road, Leicester, LE3 9QP, UK*

[4] *INSERM UMRS 937, Pierre and Marie Curie University (UPMC, Paris 6) and Medical School, 91 Bd de l'Hôpital 75013, Paris, France*

[5]*Juvenile Diabetes Research Foundation/Wellcome Trust Diabetes and Inflammation Laboratory, Department of Medical Genetics, Cambridge Institute for Medical Research, University of Cambridge, Wellcome Trust/MRC Building, Cambridge, CB2 0XY, UK*

[6]*The Wellcome Trust Sanger Institute, Wellcome Trust Genome Campus, Hinxton, Cambridge CB10 1SA, UK*

[7]*Klinik und Poliklinik für Innere Medizin II, Universität Regensburg, Germany*

[8]*Molecular Medicine, Department of Medical Sciences, Uppsala University, Uppsala, Sweden*

[9]*Trium, Analysis Online GmbH, Hohenlindenerstr. 1, 81677, München, Germany*

[10]*Institut für Klinische Chemie und Laboratoriumsmedizin, Universität, Regensburg, D-93053 Regensburg, Germany*

[11] *Leicester NIHR Biomedical Research Unit in Cardiovascular Disease, Glenfield Hospital, Leicester, LE3 9QP, UK*

[12] *European Bioinformatics Institute, Wellcome Trust Genome Campus, Hinxton, Cambridge, CB10 1SD, UK*

List of members of ICBP: The International Consortium for Blood Pressure Genome-Wide Association Studies[198]

Georg B. Ehret, Patricia B. Munroe, Kenneth M. Rice, Murielle Bochud, Andrew D. Johnson, Daniel I. Chasman, Albert V. Smith, Martin D. Tobin, Germaine C. Verwoert, Shih-Jen Hwang, Vasyl Pihur, Peter Vollenweider, Paul F. O'Reilly, Najaf Amin, Jennifer L. Bragg-Gresham, Alexander Teumer, Nicole L. Glazer, Lenore Launer, Jing Hua Zhao, Yurii Aulchenko, Simon Heath, Siim Sõber, Afshin Parsa, Jian'an Luan, Pankaj Arora, Abbas Dehghan, Feng Zhang, Gavin Lucas, Andrew A. Hicks, Anne U. Jackson, John F Peden, Toshiko Tanaka, Sarah H. Wild, Igor Rudan, Wilmar Igl, Yuri Milaneschi, Alex N. Parker, Cristiano Fava, John C. Chambers, Ervin R. Fox, Meena Kumari, Min Jin Go, Pim van der Harst, Wen Hong Linda Kao, Marketa Sjögren, D. G. Vinay, Myriam Alexander, Yasuharu Tabara, Sue Shaw-Hawkins, Peter H. Whincup, Yongmei Liu, Gang Shi, Johanna Kuusisto, Bamidele Tayo, Mark Seielstad, Xueling Sim, Khanh-Dung Hoang Nguyen, Terho Lehtimäki, Giuseppe Matullo, Ying Wu, Tom R. Gaunt, N. Charlotte Onland-

Moret, Matthew N. Cooper, Carl G. P. Platou, Elin Org, Rebecca Hardy, Santosh Dahgam, Jutta Palmen, Veronique Vitart, Peter S. Braund, Tatiana Kuznetsova, Cuno S. P. M. Uiterwaal, Adebowale Adeyemo, Walter Palmas, Harry Campbell, Barbara Ludwig, Maciej Tomaszewski, Ioanna Tzoulaki, Nicholette D. Palmer, CARDIoGRAM consortium, CKDGen Consortium, KidneyGen Consortium, EchoGen consortium, CHARGE-HF consortium, Thor Aspelund, Melissa Garcia, Yen-Pei C. Chang, Jeffrey R. O'Connell, Nanette I. Steinle, Diederick E. Grobbee, Dan E. Arking, Sharon L. Kardia, Alanna C. Morrison, Dena Hernandez, Samer Najjar, Wendy L. McArdle, David Hadley, Morris J. Brown, John M. Connell, Aroon D. Hingorani, Ian N.M. Day, Debbie A. Lawlor, John P. Beilby, Robert W. Lawrence, Robert Clarke, Jemma C. Hopewell, Halit Ongen, Albert W. Dreisbach, Yali Li, J. Hunter Young, Joshua C. Bis, Mika Kähönen, Jorma Viikari, Linda S. Adair, Nanette R. Lee, Ming-Huei Chen, Matthias Olden, Cristian Pattaro, Judith A. Hoffman Bolton, Anna Köttgen, Sven Bergmann, Vincent Mooser, Nish Chaturvedi, Timothy M. Frayling, Muhammad Islam, Tazeen H. Jafar, Jeanette Erdmann, Smita R. Kulkarni, Stefan R. Bornstein, Jürgen Grässler, Leif Groop, Benjamin F. Voight, Johannes Kettunen, Philip Howard, Andrew Taylor, Simonetta Guarrera, Fulvio Ricceri, Valur Emilsson, Andrew Plump, Inês Barroso, Kay-Tee Khaw, Alan B. Weder, Steven C. Hunt, Yan V. Sun, Richard N. Bergman, Francis S. Collins, Lori L. Bonnycastle, Laura J. Scott, Heather M. Stringham, Leena Peltonen, Markus Perola, Erkki Vartiainen, Stefan-Martin Brand, Jan A. Staessen, Thomas J. Wang, Paul R. Burton, Maria Soler Artigas, Yanbin Dong, Harold Snieder, Xiaoling Wang, Haidong Zhu, Kurt K. Lohman, Megan E. Rudock, Susan R. Heckbert, Nicholas L. Smith, Kerri L. Wiggins, Ayo Doumatey, Daniel Shriner, Gudrun Veldre, Margus Viigimaa, Sanjay Kinra, Dorairaj Prabhakaran, Vikal Tripathy, Carl D. Langefeld, Annika Rosengren, Dag S. Thelle, Anna Maria Corsi, Andrew Singleton, Terrence Forrester, Gina Hilton, Colin A. McKenzie, Tunde Salako, Naoharu Iwai, Yoshikuni Kita, Toshio Ogihara, Takayoshi Ohkubo, Tomonori Okamura, Hirotsugu Ueshima, Satoshi Umemura, Susana Eyheramendy, Thomas Meitinger, H.-Erich Wichmann, Yoon Shin Cho, Hyung-Lae Kim, Jong-Young Lee, James Scott, Joban S. Sehmi, Weihua Zhang, Bo Hedblad, Peter Nilsson, George Davey Smith, Andrew Wong, Narisu Narisu, Alena Stančáková, Leslie J. Raffel, Jie Yao, Sekar Kathiresan, Christopher J. O'Donnell, Stephen M. Schwartz, M. Arfan Ikram, W. T. Longstreth Jr, Thomas H. Mosley, Sudha Seshadri, Nick R.G. Shrine, Louise V. Wain, Mario A. Morken, Amy J. Swift, Jaana Laitinen, Inga Prokopenko, Paavo Zitting, Jackie A. Cooper, Steve E. Humphries, John Danesh, Asif Rasheed, Anuj Goel, Anders Hamsten, Hugh Watkins, Stephan J. L. Bakker, Wiek H. van Gilst, Charles S. Janipalli, K. Radha Mani, Chittaranjan S. Yajnik, Albert Hofman, Francesco U. S. Mattace-Raso, Ben A. Oostra, Ayse Demirkan, Aaron Isaacs, Fernando Rivadeneira, Edward G. Lakatta, Marco Orru, Angelo Scuteri, Mika Ala-Korpela, Antti J. Kangas, Leo-Pekka Lyytikäinen, Pasi Soininen, Taru Tukiainen, Peter Würtz, Rick Twee-Hee Ong, Marcus Dörr, Heyo K. Kroemer, Uwe Völker, Henry Völzke, Pilar Galan, Serge Hercberg, Mark Lathrop, Diana Zelenika, Panos Deloukas, Massimo Mangino, Tim D. Spector, Guangju Zhai, James F. Meschia, Michael A. Nalls, Pankaj Sharma, Janos Terzic, M. V. Kranthi Kumar, Matthew Denniff, Ewa Zukowska-Szczechowska, Lynne E. Wagenknecht, F. Gerald R. Fowkes, Fadi J. Charchar, Peter E. H. Schwarz, Caroline Hayward, Xiuqing Guo, Charles Rotimi, Michiel L. Bots, Eva Brand, Nilesh J. Samani, Ozren Polasek, Philippa J. Talmud, Fredrik Nyberg, Diana Kuh, Maris Laan, Kristian Hveem, Lyle J. Palmer, Yvonne T. van der Schouw, Juan P. Casas, Karen L. Mohlke, Paolo Vineis, Olli Raitakari, Santhi K. Ganesh, Tien Y. Wong, E Shyong Tai, Richard S. Cooper, Markku Laakso, Dabeeru C. Rao, Tamara B. Harris, Richard W. Morris, Anna F. Dominiczak, Mika Kivimaki, Michael G. Marmot, Tetsuro Miki, Danish Saleheen, Giriraj R. Chandak, Josef Coresh, Gerjan Navis, Veikko Salomaa, Bok-Ghee Han, Xiaofeng Zhu, Jaspal S. Kooner, Olle Melander, Paul M Ridker, Stefania Bandinelli, Ulf B. Gyllensten, Alan F. Wright, James F. Wilson, Luigi Ferrucci, Martin Farrall, Jaakko Tuomilehto, Peter P. Pramstaller, Roberto Elosua, Nicole Soranzo, Eric J. G. Sijbrands, David Altshuler, Ruth J. F. Loos, Alan R. Shuldiner, Christian Gieger, Pierre Meneton, Andre G. Uitterlinden, Nicholas J. Wareham, Vilmundur Gudnason, Jerome I. Rotter, Rainer Rettig, Manuela Uda, David P. Strachan, Jacqueline C. M. Witteman, Anna-Liisa Hartikainen, Jacques S. Beckmann, Eric Boerwinkle, Ramachandran S. Vasan, Michael Boehnke, Martin G. Larson,

Marjo-Riitta Järvelin, Bruce M. Psaty, Gonçalo R. Abecasis, Aravinda Chakravarti, Paul Elliott, Cornelia M. van Duijn, Christopher Newton-Cheh, Daniel Levy, Mark J. Caulfield & Toby Johnson.

REFERENCES

1. Bown MJ, Jones GT, Harrison SC, et al. Abdominal aortic aneurysm is associated with a variant in low-density lipoprotein receptor-related protein 1. *Am J Hum Genet*. 2011;89:619-627
2. Gretarsdottir S, Baas AF, Thorleifsson G, et al. Genome-wide association study identifies a sequence variant within the dab2ip gene conferring susceptibility to abdominal aortic aneurysm. *Nature Genet*. 2010;42:692-U671
3. Jones GT, Bown MJ, Gretarsdottir S, et al. A sequence variant associated with sortilin-1 (sort1) on 1p13.3 is independently associated with abdominal aortic aneurysm. *Hum Mol Genet*. 2013
4. Harrison SC, Zabaneh D, Asselbergs FW, et al. A gene-centric study of common carotid artery remodelling. *Atherosclerosis*. 2013;226:440-446
5. Teo YY. Genotype calling for the illumina platform. *Methods Mol Biol*. 2012;850:525-538
6. Purcell S, Neale B, Todd-Brown K, Thomas L, Ferreira MA, Bender D, Maller J, Sklar P, de Bakker PI, Daly MJ, Sham PC. Plink: A tool set for whole-genome association and population-based linkage analyses. *Am J Hum Genet*. 2007;81:559-575
7. Elmore JR, Obmann MA, Kuivaniemi H, Tromp G, Gerhard GS, Franklin DP, Boddy AM, Carey DJ. Identification of a genetic variant associated with abdominal aortic aneurysms on chromosome 3p12.3 by genome wide association. *J Vasc Surg*. 2009;49:1525-1531
8. Borthwick KM, Smelser DT, Bock JA, et al. Ephenotyping for abdominal aortic aneurysm in the electronic medical records and genomics (emerge) network: Algorithm development and konstanz information miner workflow. *Int J Biomed Data Mining*. 2015;4
9. Verma SS, de Andrade M, Tromp G, et al. Imputation and quality control steps for combining multiple genome-wide datasets. *Front Genet*. 2014;5:370
10. Delaneau O, Marchini J, Zagury JF. A linear complexity phasing method for thousands of genomes. *Nat Methods*. 2012;9:179-181
11. Howie B, Fuchsberger C, Stephens M, Marchini J, Abecasis GR. Fast and accurate genotype imputation in genome-wide association studies through pre-phasing. *Nat Genet*. 2012;44:955-959
12. Gretarsdottir S, Thorleifsson G, Reynisdottir ST, et al. The gene encoding phosphodiesterase 4d confers risk of ischemic stroke. *Nat Genet*. 2003;35:131-138
13. Rice JA. Generalized likelihood ratio tests. In: Rice ja, editor. Mathematical statistics and data analysis. Vol. 1. International thomson publishing; 1995. P. 308–310.
14. Rafnar T, Sulem P, Stacey SN, et al. Sequence variants at the tert-clptm1l locus associate with many cancer types. *Nat Genet*. 2009;41:221-227
15. Kiemeney LA, Thorlacius S, Sulem P, et al. Sequence variant on 8q24 confers susceptibility to urinary bladder cancer. *Nat Genet*. 2008;40:1307-1312
16. Wetzels JF, Kiemeney LA, Swinkels DW, Willems HL, den Heijer M. Age- and gender-specific reference values of estimated gfr in caucasians: The nijmegen biomedical study. *Kidney Int*. 2007;72:632-637

17. Bown MJ, Braund PS, Thompson J, London NJ, Samani NJ, Sayers RD. Association between the coronary artery disease risk locus on chromosome 9p21.3 and abdominal aortic aneurysm. *Circ Cardiovasc Genet*. 2008;1:39-42
18. Howie B, Marchini J, Stephens M. Genotype imputation with thousands of genomes. *G3 (Bethesda)*. 2011;1:457-470
19. Devlin B, Roeder K. Genomic control for association studies. *Biometrics*. 1999;55:997-1004
20. Shi YY, He L. Shesis, a powerful software platform for analyses of linkage disequilibrium, haplotype construction, and genetic association at polymorphism loci. *Cell Res*. 2005;15:97-98
21. Helgadottir A, Thorleifsson G, Magnusson KP, et al. The same sequence variant on 9p21 associates with myocardial infarction, abdominal aortic aneurysm and intracranial aneurysm. *Nat Genet*. 2008;40:217-224
22. Ogata T, Shibamura H, Tromp G, Sinha M, Goddard KA, Sakalihasan N, Limet R, MacKean GL, Arthur C, Sueda T, Land S, Kuivaniemi H. Genetic analysis of polymorphisms in biologically relevant candidate genes in patients with abdominal aortic aneurysms. *J Vasc Surg*. 2005;41:1036-1042
23. Gottesman O, Kuivaniemi H, Tromp G, et al. The electronic medical records and genomics (emerge) network: Past, present, and future. *Genet Med*. 2013;15:761-771
24. Ye Z, Kalloo FS, Dalenberg AK, Kullo IJ. An electronic medical record-linked biorepository to identify novel biomarkers for atherosclerotic cardiovascular disease. *Glob Cardiol Sci Pract*. 2013;2013:82-90
25. St Jean PL, Zhang XC, Hart BK, Lamlum H, Webster MW, Steed DL, Henney AM, Ferrell RE. Characterization of a dinucleotide repeat in the 92 kda type iv collagenase gene (clg4b), localization of clg4b to chromosome 20 and the role of clg4b in aortic aneurysmal disease. *Ann Hum Genet*. 1995;59:17-24
26. Pulley J, Clayton E, Bernard GR, Roden DM, Masys DR. Principles of human subjects protections applied in an opt-out, de-identified biobank. *Clin Transl Sci*. 2010;3:42-48
27. McCarty CA, Wilke RA, Giampietro PF, Wesbrook SD, Caldwell MD. Marshfield clinic personalized medicine research project (pmrp): Design, methods and recruitment for a large population-based biobank. *Personalized Medicine*. 2005;2:49-79
28. Tayo BO, Teil M, Tong L, Qin H, Khitrov G, Zhang W, Song Q, Gottesman O, Zhu X, Pereira AC, Cooper RS, Bottinger EP. Genetic background of patients from a university medical center in manhattan: Implications for personalized medicine. *PLoS One*. 2011;6:e19166
29. Kho AN, Hayes MG, Rasmussen-Torvik L, et al. Use of diverse electronic medical record systems to identify genetic risk for type 2 diabetes within a genome-wide association study. *J Am Med Inform Assoc*. 2012;19:212-218
30. Galora S, Saracini C, Pratesi G, Sticchi E, Pulli R, Pratesi C, Abbate R, Giusti B. Association of rs1466535 lrp1 but not rs3019885 slc30a8 and rs6674171 tdrd10 gene polymorphisms with abdominal aortic aneurysm in italian patients. *J Vasc Surg*. 2014
31. Strauss E, Waliszewski K, Oszkinis G, Staniszewski R. Polymorphisms of genes involved in the hypoxia signaling pathway and the development of abdominal aortic aneurysms or large-artery atherosclerosis. *J Vasc Surg*. 2015;61:1105-1113.e1103

32. Willer CJ, Li Y, Abecasis GR. Metal: Fast and efficient meta-analysis of genomewide association scans. *Bioinformatics*. 2010;26:2190-2191
33. Han B, Eskin E. Random-effects model aimed at discovering associations in meta-analysis of genome-wide association studies. *Am J Hum Genet*. 2011;88:586-598
34. Morris AP, Voight BF, Teslovich TM, et al. Large-scale association analysis provides insights into the genetic architecture and pathophysiology of type 2 diabetes. *Nat Genet*. 2012;44:981-990
35. Schunkert H, Konig IR, Kathiresan S, et al. Large-scale association analysis identifies 13 new susceptibility loci for coronary artery disease. *Nat Genet*. 2011;43:333-338
36. Willer CJ, Schmidt EM, Sengupta S, et al. Discovery and refinement of loci associated with lipid levels. *Nat Genet*. 2013;45:1274-1283
37. Wain LV, Verwoert GC, O'Reilly PF, et al. Genome-wide association study identifies six new loci influencing pulse pressure and mean arterial pressure. *Nat Genet*. 2011;43:1005-1011
38. Ramos EM, Hoffman D, Junkins HA, Maglott D, Phan L, Sherry ST, Feolo M, Hindorff LA. Phenotype-genotype integrator (phegeni): Synthesizing genome-wide association study (gwas) data with existing genomic resources. *Eur J Hum Genet*. 2014;22:144-147
39. Eicher JD, Landowski C, Stackhouse B, Sloan A, Chen W, Jensen N, Lien JP, Leslie R, Johnson AD. Grasp v2.0: An update on the genome-wide repository of associations between snps and phenotypes. *Nucleic Acids Res*. 2015;43:D799-804
40. Leslie R, O'Donnell CJ, Johnson AD. Grasp: Analysis of genotype-phenotype results from 1390 genome-wide association studies and corresponding open access database. *Bioinformatics*. 2014;30:i185-194
41. Willer CJ, Sanna S, Jackson AU, et al. Newly identified loci that influence lipid concentrations and risk of coronary artery disease. *Nat Genet*. 2008;40:161-169
42. Sandhu MS, Waterworth DM, Debenham SL, et al. Ldl-cholesterol concentrations: A genome-wide association study. *Lancet*. 2008;371:483-491
43. Kathiresan S, Willer CJ, Peloso GM, et al. Common variants at 30 loci contribute to polygenic dyslipidemia. *Nat Genet*. 2009;41:56-65
44. Talmud PJ, Drenos F, Shah S, et al. Gene-centric association signals for lipids and apolipoproteins identified via the humancvd beadchip. *Am J Hum Genet*. 2009;85:628-642
45. Clarke R, Peden JF, Hopewell JC, et al. Genetic variants associated with lp(a) lipoprotein level and coronary disease. *N Engl J Med*. 2009;361:2518-2528
46. Barber MJ, Mangravite LM, Hyde CL, et al. Genome-wide association of lipid-lowering response to statins in combined study populations. *PLoS One*. 2010;5:e9763
47. Teslovich TM, Musunuru K, Smith AV, et al. Biological, clinical and population relevance of 95 loci for blood lipids. *Nature*. 2010;466:707-713
48. Lango Allen H, Estrada K, Lettre G, et al. Hundreds of variants clustered in genomic loci and biological pathways affect human height. *Nature*. 2010;467:832-838
49. Lettre G, Palmer CD, Young T, et al. Genome-wide association study of coronary heart disease and its risk factors in 8,090 african americans: The nhlbi care project. *PLoS Genet*. 2011;7:e1001300

50. Middelberg RP, Ferreira MA, Henders AK, Heath AC, Madden PA, Montgomery GW, Martin NG, Whitfield JB. Genetic variants in lpl, oasl and tomm40/apoe-c1-c2-c4 genes are associated with multiple cardiovascular-related traits. *BMC Med Genet*. 2011;12:123
51. Trompet S, de Craen AJ, Postmus I, Ford I, Sattar N, Caslake M, Stott DJ, Buckley BM, Sacks F, Devlin JJ, Slagboom PE, Westendorp RG, Jukema JW. Replication of ldl gwas hits in prosper/phase as validation for future (pharmaco)genetic analyses. *BMC Med Genet*. 2011;12:131
52. Grallert H, Dupuis J, Bis JC, et al. Eight genetic loci associated with variation in lipoprotein-associated phospholipase a2 mass and activity and coronary heart disease: Meta-analysis of genome-wide association studies from five community-based studies. *Eur Heart J*. 2012;33:238-251
53. Asselbergs FW, Guo Y, van Iperen EP, et al. Large-scale gene-centric meta-analysis across 32 studies identifies multiple lipid loci. *Am J Hum Genet*. 2012;91:823-838
54. Deloukas P, Kanoni S, Willenborg C, et al. Large-scale association analysis identifies new risk loci for coronary artery disease. *Nat Genet*. 2013;45:25-33
55. Wilk JB, Walter RE, Laramie JM, Gottlieb DJ, O'Connor GT. Framingham heart study genome-wide association: Results for pulmonary function measures. *BMC Med Genet*. 2007;8 Suppl 1:S8
56. Ridker PM, Pare G, Parker A, Zee RY, Danik JS, Buring JE, Kwiatkowski D, Cook NR, Miletich JP, Chasman DI. Loci related to metabolic-syndrome pathways including lepr,hnf1a, il6r, and gckr associate with plasma c-reactive protein: The women's genome health study. *Am J Hum Genet*. 2008;82:1185-1192
57. Melzer D, Perry JR, Hernandez D, et al. A genome-wide association study identifies protein quantitative trait loci (pqtls). *PLoS Genet*. 2008;4:e1000072
58. Sabatti C, Service SK, Hartikainen AL, et al. Genome-wide association analysis of metabolic traits in a birth cohort from a founder population. *Nat Genet*. 2009;41:35-46
59. Danik JS, Pare G, Chasman DI, Zee RY, Kwiatkowski DJ, Parker A, Miletich JP, Ridker PM. Novel loci, including those related to crohn disease, psoriasis, and inflammation, identified in a genome-wide association study of fibrinogen in 17 686 women: The women's genome health study. *Circ Cardiovasc Genet*. 2009;2:134-141
60. Ferreira MA, Matheson MC, Duffy DL, et al. Identification of il6r and chromosome 11q13.5 as risk loci for asthma. *Lancet*. 2011;378:1006-1014
61. Wassel CL, Lange LA, Keating BJ, et al. Association of genomic loci from a cardiovascular gene snp array with fibrinogen levels in european americans and african-americans from six cohort studies: The candidate gene association resource (care). *Blood*. 2011;117:268-275
62. Dehghan A, Dupuis J, Barbalic M, et al. Meta-analysis of genome-wide association studies in >80 000 subjects identifies multiple loci for c-reactive protein levels. *Circulation*. 2011;123:731-738
63. Naitza S, Porcu E, Steri M, et al. A genome-wide association scan on the levels of markers of inflammation in sardinians reveals associations that underpin its complex regulation. *PLoS Genet*. 2012;8:e1002480

64. Reiner AP, Beleza S, Franceschini N, Auer PL, Robinson JG, Kooperberg C, Peters U, Tang H. Genome-wide association and population genetic analysis of c-reactive protein in african american and hispanic american women. *Am J Hum Genet*. 2012;91:502-512
65. Shah T, Zabaneh D, Gaunt T, et al. Gene-centric analysis identifies variants associated with interleukin-6 levels and shared pathways with other inflammation markers. *Circ Cardiovasc Genet*. 2013;6:163-170
66. McPherson R, Pertsemlidis A, Kavaslar N, Stewart A, Roberts R, Cox DR, Hinds DA, Pennacchio LA, Tybjaerg-Hansen A, Folsom AR, Boerwinkle E, Hobbs HH, Cohen JC. A common allele on chromosome 9 associated with coronary heart disease. *Science*. 2007;316:1488-1491
67. Lu X, Wang L, Chen S, et al. Genome-wide association study in han chinese identifies four new susceptibility loci for coronary artery disease. *Nat Genet*. 2012;44:890-894
68. Pechlivanis S, Muhleisen TW, Mohlenkamp S, Schadendorf D, Erbel R, Jockel KH, Hoffmann P, Nothen MM, Scherag A, Moebus S. Risk loci for coronary artery calcification replicated at 9p21 and 6q24 in the heinz nixdorf recall study. *BMC Med Genet*. 2013;14:23
69. Ferguson JF, Matthews GJ, Townsend RR, et al. Candidate gene association study of coronary artery calcification in chronic kidney disease: Findings from the cric study (chronic renal insufficiency cohort). *J Am Coll Cardiol*. 2013;62:789-798
70. Saxena R, Voight BF, Lyssenko V, et al. Genome-wide association analysis identifies loci for type 2 diabetes and triglyceride levels. *Science*. 2007;316:1331-1336
71. Stefansson H, Ophoff RA, Steinberg S, et al. Common variants conferring risk of schizophrenia. *Nature*. 2009;460:744-747
72. Kathiresan S, Melander O, Guiducci C, et al. Six new loci associated with blood low-density lipoprotein cholesterol, high-density lipoprotein cholesterol or triglycerides in humans. *Nat Genet*. 2008;40:189-197
73. Aulchenko YS, Ripatti S, Lindqvist I, et al. Loci influencing lipid levels and coronary heart disease risk in 16 european population cohorts. *Nat Genet*. 2009;41:47-55
74. Chasman DI, Pare G, Zee RY, et al. Genetic loci associated with plasma concentration of low-density lipoprotein cholesterol, high-density lipoprotein cholesterol, triglycerides, apolipoprotein a1, and apolipoprotein b among 6382 white women in genome-wide analysis with replication. *Circ Cardiovasc Genet*. 2008;1:21-30
75. Chasman DI, Pare G, Mora S, et al. Forty-three loci associated with plasma lipoprotein size, concentration, and cholesterol content in genome-wide analysis. *PLoS Genet*. 2009;5:e1000730
76. Bis JC, Kavousi M, Franceschini N, et al. Meta-analysis of genome-wide association studies from the charge consortium identifies common variants associated with carotid intima media thickness and plaque. *Nat Genet*. 2011;43:940-947
77. Large-scale gene-centric analysis identifies novel variants for coronary artery disease. *PLoS Genet*. 2011;7:e1002260
78. Avery CL, He Q, North KE, et al. A phenomics-based strategy identifies loci on apoc1, brap, and plcg1 associated with metabolic syndrome phenotype domains. *PLoS Genet*. 2011;7:e1002322

79. Musunuru K, Romaine SP, Lettre G, et al. Multi-ethnic analysis of lipid-associated loci: The nhlbi care project. *PLoS One*. 2012;7:e36473
80. Hopewell JC, Parish S, Offer A, Link E, Clarke R, Lathrop M, Armitage J, Collins R. Impact of common genetic variation on response to simvastatin therapy among 18 705 participants in the heart protection study. *Eur Heart J*. 2013;34:982-992
81. Elbers CC, Guo Y, Tragante V, et al. Gene-centric meta-analysis of lipid traits in african, east asian and hispanic populations. *PLoS One*. 2012;7:e50198
82. Weissglas-Volkov D, Aguilar-Salinas CA, Nikkola E, et al. Genomic study in mexicans identifies a new locus for triglycerides and refines european lipid loci. *J Med Genet*. 2013;50:298-308
83. Denny JC, Ritchie MD, Basford MA, Pulley JM, Bastarache L, Brown-Gentry K, Wang D, Masys DR, Roden DM, Crawford DC. Phewas: Demonstrating the feasibility of a phenome-wide scan to discover gene-disease associations. *Bioinformatics*. 2010;26:1205-1210
84. Pendergrass SA, Brown-Gentry K, Dudek S, et al. Phenome-wide association study (phewas) for detection of pleiotropy within the population architecture using genomics and epidemiology (page) network. *PLoS Genet*. 2013;9:e1003087
85. Hsu F, Kent WJ, Clawson H, Kuhn RM, Diekhans M, Haussler D. The ucsc known genes. *Bioinformatics*. 2006;22:1036-1046
86. Benson DA, Karsch-Mizrachi I, Lipman DJ, Ostell J, Wheeler DL. Genbank: Update. *Nucleic Acids Res*. 2004;32:D23-26
87. Kent WJ. Blat--the blast-like alignment tool. *Genome Res*. 2002;12:656-664
88. Pruitt KD, Tatusova T, Maglott DR. Ncbi reference sequence (refseq): A curated non-redundant sequence database of genomes, transcripts and proteins. *Nucleic Acids Res*. 2005;33:D501-504
89. Gardner PP, Daub J, Tate J, Moore BL, Osuch IH, Griffiths-Jones S, Finn RD, Nawrocki EP, Kolbe DL, Eddy SR, Bateman A. Rfam: Wikipedia, clans and the "decimal" release. *Nucleic Acids Res*. 2011;39:D141-145
90. Lowe TM, Eddy SR. Trnascan-se: A program for improved detection of transfer rna genes in genomic sequence. *Nucleic Acids Res*. 1997;25:955-964
91. Reorganizing the protein space at the universal protein resource (uniprot). *Nucleic Acids Res*. 2012;40:D71-75
92. Harrow J, Frankish A, Gonzalez JM, et al. Gencode: The reference human genome annotation for the encode project. *Genome Res*. 2012;22:1760-1774
93. Cabili MN, Trapnell C, Goff L, Koziol M, Tazon-Vega B, Regev A, Rinn JL. Integrative annotation of human large intergenic noncoding rnas reveals global properties and specific subclasses. *Genes Dev*. 2011;25:1915-1927
94. Trapnell C, Williams BA, Pertea G, Mortazavi A, Kwan G, van Baren MJ, Salzberg SL, Wold BJ, Pachter L. Transcript assembly and quantification by rna-seq reveals unannotated transcripts and isoform switching during cell differentiation. *Nat Biotechnol*. 2010;28:511-515
95. Kent WJ, Sugnet CW, Furey TS, Roskin KM, Pringle TH, Zahler AM, Haussler D. The human genome browser at ucsc. *Genome Res*. 2002;12:996-1006

96. Friedman RC, Farh KK, Burge CB, Bartel DP. Most mammalian mrnas are conserved targets of micrornas. *Genome Res*. 2009;19:92-105
97. Grimson A, Farh KK, Johnston WK, Garrett-Engele P, Lim LP, Bartel DP. Microrna targeting specificity in mammals: Determinants beyond seed pairing. *Mol Cell*. 2007;27:91-105
98. Mortazavi A, Williams BA, McCue K, Schaeffer L, Wold B. Mapping and quantifying mammalian transcriptomes by rna-seq. *Nat Methods*. 2008;5:621-628
99. Ernst J, Kheradpour P, Mikkelsen TS, Shoresh N, Ward LD, Epstein CB, Zhang X, Wang L, Issner R, Coyne M, Ku M, Durham T, Kellis M, Bernstein BE. Mapping and analysis of chromatin state dynamics in nine human cell types. *Nature*. 2011;473:43-49
100. An integrated encyclopedia of DNA elements in the human genome. *Nature*. 2012;489:57-74
101. Wang J, Zhuang J, Iyer S, et al. Sequence features and chromatin structure around the genomic regions bound by 119 human transcription factors. *Genome Res*. 2012;22:1798-1812
102. Wang J, Zhuang J, Iyer S, Lin XY, Greven MC, Kim BH, Moore J, Pierce BG, Dong X, Virgil D, Birney E, Hung JH, Weng Z. Factorbook.Org: A wiki-based database for transcription factor-binding data generated by the encode consortium. *Nucleic Acids Res*. 2013;41:D171-176
103. Hubisz MJ, Pollard KS, Siepel A. Phast and rphast: Phylogenetic analysis with space/time models. *Brief Bioinform*. 2011;12:41-51
104. Jurka J. Repbase update: A database and an electronic journal of repetitive elements. *Trends Genet*. 2000;16:418-420
105. Reumers J, Conde L, Medina I, Maurer-Stroh S, Van Durme J, Dopazo J, Rousseau F, Schymkowitz J. Joint annotation of coding and non-coding single nucleotide polymorphisms and mutations in the snpeffect and pupasuite databases. *Nucleic Acids Res*. 2008;36:D825-829
106. Li MJ, Wang LY, Xia Z, Sham PC, Wang J. Gwas3d: Detecting human regulatory variants by integrative analysis of genome-wide associations, chromosome interactions and histone modifications. *Nucleic Acids Res*. 2013;41:W150-158
107. Biros E, Gabel G, Moran CS, Schreurs C, Lindeman JH, Walker PJ, Nataatmadja M, West M, Holdt LM, Hinterseher I, Pilarsky C, Golledge J. Differential gene expression in human abdominal aortic aneurysm and aortic occlusive disease. *Oncotarget*. 2015;6:12984-12996
108. Pers TH, Karjalainen JM, Chan Y, et al. Biological interpretation of genome-wide association studies using predicted gene functions. *Nat Commun*. 2015;6:5890
109. Zhang X, Gierman HJ, Levy D, Plump A, Dobrin R, Goring HH, Curran JE, Johnson MP, Blangero J, Kim SK, O'Donnell CJ, Emilsson V, Johnson AD. Synthesis of 53 tissue and cell line expression qtl datasets reveals master eqtls. *BMC Genomics*. 2014;15:532
110. Goring HH, Curran JE, Johnson MP, et al. Discovery of expression qtls using large-scale transcriptional profiling in human lymphocytes. *Nat Genet*. 2007;39:1208-1216
111. Idaghdour Y, Czika W, Shianna KV, Lee SH, Visscher PM, Martin HC, Miclaus K, Jadallah SJ, Goldstein DB, Wolfinger RD, Gibson G. Geographical genomics of human leukocyte gene expression variation in southern morocco. *Nat Genet*. 2010;42:62-67

112. Heap GA, Trynka G, Jansen RC, Bruinenberg M, Swertz MA, Dinesen LC, Hunt KA, Wijmenga C, Vanheel DA, Franke L. Complex nature of snp genotype effects on gene expression in primary human leucocytes. *BMC Med Genomics*. 2009;2:1
113. Battle A, Mostafavi S, Zhu X, Potash JB, Weissman MM, McCormick C, Haudenschild CD, Beckman KB, Shi J, Mei R, Urban AE, Montgomery SB, Levinson DF, Koller D. Characterizing the genetic basis of transcriptome diversity through rna-sequencing of 922 individuals. *Genome Res*. 2014;24:14-24
114. Benton MC, Lea RA, Macartney-Coxson D, Carless MA, Goring HH, Bellis C, Hanna M, Eccles D, Chambers GK, Curran JE, Harper JL, Blangero J, Griffiths LR. Mapping eqtls in the norfolk island genetic isolate identifies candidate genes for cvd risk traits. *Am J Hum Genet*. 2013;93:1087-1099
115. Emilsson V, Thorleifsson G, Zhang B, et al. Genetics of gene expression and its effect on disease. *Nature*. 2008;452:423-428
116. Fehrmann RS, Jansen RC, Veldink JH, et al. Trans-eqtls reveal that independent genetic variants associated with a complex phenotype converge on intermediate genes, with a major role for the hla. *PLoS Genet*. 2011;7:e1002197
117. Landmark-Hoyvik H, Dumeaux V, Nebdal D, Lund E, Tost J, Kamatani Y, Renault V, Borresen-Dale AL, Kristensen V, Edvardsen H. Genome-wide association study in breast cancer survivors reveals snps associated with gene expression of genes belonging to mhc class i and ii. *Genomics*. 2013;102:278-287
118. Mehta D, Heim K, Herder C, Carstensen M, Eckstein G, Schurmann C, Homuth G, Nauck M, Volker U, Roden M, Illig T, Gieger C, Meitinger T, Prokisch H. Impact of common regulatory single-nucleotide variants on gene expression profiles in whole blood. *Eur J Hum Genet*. 2013;21:48-54
119. Narahara M, Higasa K, Nakamura S, Tabara Y, Kawaguchi T, Ishii M, Matsubara K, Matsuda F, Yamada R. Large-scale east-asian eqtl mapping reveals novel candidate genes for ld mapping and the genomic landscape of transcriptional effects of sequence variants. *PLoS One*. 2014;9:e100924
120. Quinlan J, Idaghdour Y, Goulet JP, Gbeha E, de Malliard T, Bruat V, Grenier JC, Gomez S, Sanni A, Rahimy MC, Awadalla P. Genomic architecture of sickle cell disease in west african children. *Front Genet*. 2014;5:26
121. Sasayama D, Hori H, Nakamura S, Miyata R, Teraishi T, Hattori K, Ota M, Yamamoto N, Higuchi T, Amano N, Kunugi H. Identification of single nucleotide polymorphisms regulating peripheral blood mrna expression with genome-wide significance: An eqtl study in the japanese population. *PLoS One*. 2013;8:e54967
122. Schramm K, Marzi C, Schurmann C, et al. Mapping the genetic architecture of gene regulation in whole blood. *PLoS One*. 2014;9:e93844
123. van Eijk KR, de Jong S, Boks MP, Langeveld T, Colas F, Veldink JH, de Kovel CG, Janson E, Strengman E, Langfelder P, Kahn RS, van den Berg LH, Horvath S, Ophoff RA. Genetic analysis of DNA methylation and gene expression levels in whole blood of healthy human subjects. *BMC Genomics*. 2012;13:636
124. Westra HJ, Peters MJ, Esko T, et al. Systematic identification of trans eqtls as putative drivers of known disease associations. *Nat Genet*. 2013;45:1238-1243

125. Wright FA, Sullivan PF. Heritability and genomics of gene expression in peripheral blood. 2014;46:430-437
126. Zhernakova DV, de Klerk E, Westra HJ, et al. Deepsage reveals genetic variants associated with alternative polyadenylation and expression of coding and non-coding transcripts. *PLoS Genet*. 2013;9:e1003594
127. Dixon AL, Liang L, Moffatt MF, Chen W, Heath S, Wong KC, Taylor J, Burnett E, Gut I, Farrall M, Lathrop GM, Abecasis GR, Cookson WO. A genome-wide association study of global gene expression. *Nat Genet*. 2007;39:1202-1207
128. Liang L, Morar N, Dixon AL, Lathrop GM, Abecasis GR, Moffatt MF, Cookson WO. A cross-platform analysis of 14,177 expression quantitative trait loci derived from lymphoblastoid cell lines. *Genome Res*. 2013;23:716-726
129. Stranger BE, Nica AC, Forrest MS, Dimas A, Bird CP, Beazley C, Ingle CE, Dunning M, Flicek P, Koller D, Montgomery S, Tavare S, Deloukas P, Dermitzakis ET. Population genomics of human gene expression. *Nat Genet*. 2007;39:1217-1224
130. Kwan T, Benovoy D, Dias C, Gurd S, Provencher C, Beaulieu P, Hudson TJ, Sladek R, Majewski J. Genome-wide analysis of transcript isoform variation in humans. *Nat Genet*. 2008;40:225-231
131. Bryois J, Buil A, Evans DM, Kemp JP, Montgomery SB, Conrad DF, Ho KM, Ring S, Hurles M, Deloukas P, Davey Smith G, Dermitzakis ET. Cis and trans effects of human genomic variants on gene expression. *PLoS Genet*. 2014;10:e1004461
132. Cusanovich DA, Billstrand C, Zhou X, Chavarria C, De Leon S, Michelini K, Pai AA, Ober C, Gilad Y. The combination of a genome-wide association study of lymphocyte count and analysis of gene expression data reveals novel asthma candidate genes. *Hum Mol Genet*. 2012;21:2111-2123
133. Dimas AS, Deutsch S, Stranger BE, et al. Common regulatory variation impacts gene expression in a cell type-dependent manner. *Science*. 2009;325:1246-1250
134. Grundberg E, Small KS, Hedman AK, et al. Mapping cis- and trans-regulatory effects across multiple tissues in twins. *Nat Genet*. 2012;44:1084-1089
135. Gutierrez-Arcelus M, Lappalainen T, Montgomery SB, et al. Passive and active DNA methylation and the interplay with genetic variation in gene regulation. *Elife*. 2013;2:e00523
136. Mangravite LM, Engelhardt BE, Medina MW, et al. A statin-dependent qtl for gatm expression is associated with statin-induced myopathy. *Nature*. 2013;502:377-380
137. Fairfax BP, Makino S, Radhakrishnan J, Plant K, Leslie S, Dilthey A, Ellis P, Langford C, Vannberg FO, Knight JC. Genetics of gene expression in primary immune cells identifies cell type-specific master regulators and roles of hla alleles. *Nat Genet*. 2012;44:502-510
138. Murphy A, Chu JH, Xu M, et al. Mapping of numerous disease-associated expression polymorphisms in primary peripheral blood cd4+ lymphocytes. *Hum Mol Genet*. 2010;19:4745-4757
139. Heinzen EL, Ge D, Cronin KD, Maia JM, Shianna KV, Gabriel WN, Welsh-Bohmer KA, Hulette CM, Denny TN, Goldstein DB. Tissue-specific genetic control of splicing: Implications for the study of complex traits. *PLoS Biol*. 2008;6:e1
140. Zeller T, Wild P, Szymczak S, et al. Genetics and beyond--the transcriptome of human monocytes and disease susceptibility. *PLoS One*. 2010;5:e10693

141. Fairfax BP, Humburg P, Makino S, Naranbhai V, Wong D, Lau E, Jostins L, Plant K, Andrews R, McGee C, Knight JC. Innate immune activity conditions the effect of regulatory variants upon monocyte gene expression. *Science*. 2014;343:1246949
142. Barreiro LB, Tailleux L, Pai AA, Gicquel B, Marioni JC, Gilad Y. Deciphering the genetic architecture of variation in the immune response to mycobacterium tuberculosis infection. *Proc Natl Acad Sci U S A*. 2012;109:1204-1209
143. Lee MN, Ye C, Villani AC, et al. Common genetic variants modulate pathogen-sensing responses in human dendritic cells. *Science*. 2014;343:1246980
144. Huang RS, Gamazon ER, Ziliak D, Wen Y, Im HK, Zhang W, Wing C, Duan S, Bleibel WK, Cox NJ, Dolan ME. Population differences in microrna expression and biological implications. *RNA Biol*. 2011;8:692-701
145. Degner JF, Pai AA, Pique-Regi R, Veyrieras JB, Gaffney DJ, Pickrell JK, De Leon S, Michelini K, Lewellen N, Crawford GE, Stephens M, Gilad Y, Pritchard JK. Dnase i sensitivity qtls are a major determinant of human expression variation. *Nature*. 2012;482:390-394
146. The genotype-tissue expression (gtex) project. *Nat Genet*. 2013;45:580-585
147. Greenawalt DM, Dobrin R, Chudin E, et al. A survey of the genetics of stomach, liver, and adipose gene expression from a morbidly obese cohort. *Genome Res*. 2011;21:1008-1016
148. Kompass KS, Witte JS. Co-regulatory expression quantitative trait loci mapping: Method and application to endometrial cancer. *BMC Med Genomics*. 2011;4:6
149. Li Q, Seo JH, Stranger B, McKenna A, Pe'er I, Laframboise T, Brown M, Tyekucheva S, Freedman ML. Integrative eqtl-based analyses reveal the biology of breast cancer risk loci. *Cell*. 2013;152:633-641
150. Chen TH, D'Ambrosio D, Gallins P, et al. Mapping of hepatic expression quantitative trait loci (eqtls) in a han chinese population. *Nat Genet*. 2014;51:319-326
151. Innocenti F, Cooper GM, Stanaway IB, et al. Identification, replication, and functional fine-mapping of expression quantitative trait loci in primary human liver tissue. *PLoS Genet*. 2011;7:e1002078
152. Schadt EE, Molony C, Chudin E, et al. Mapping the genetic architecture of gene expression in human liver. *PLoS Biol*. 2008;6:e107
153. Schroder A, Klein K, Winter S, Schwab M, Bonin M, Zell A, Zanger UM. Genomics of adme gene expression: Mapping expression quantitative trait loci relevant for absorption, distribution, metabolism and excretion of drugs in human liver. *Pharmacogenomics J*. 2013;13:12-20
154. Grundberg E, Kwan T, Ge B, et al. Population genomics in a disease targeted primary cell model. *Genome Res*. 2009;19:1942-1952
155. Kabakchiev B, Silverberg MS. Expression quantitative trait loci analysis identifies associations between genotype and gene expression in human intestine. *Gastroenterology*. 2013;144:1488-1496, 1496.e1481-1483
156. Ongen H, Andersen CL, Bramsen JB, et al. Putative cis-regulatory drivers in colorectal cancer. *Nature*. 2014;512:87-90
157. Keildson S, Fadista J, Ladenvall C, et al. Expression of phosphofructokinase in skeletal muscle is influenced by genetic variation and associated with insulin sensitivity. *Diabetes*. 2014;63:1154-1165

158. Curtis C, Shah SP, Chin SF, et al. The genomic and transcriptomic architecture of 2,000 breast tumours reveals novel subgroups. *Nature*. 2012;486:346-352
159. Quigley DA, Fiorito E, Nord S, et al. The 5p12 breast cancer susceptibility locus affects mrps30 expression in estrogen-receptor positive tumors. *Mol Oncol*. 2014;8:273-284
160. Gao C, Tignor NL, Salit J, Strulovici-Barel Y, Hackett NR, Crystal RG, Mezey JG. Heft: Eqtl analysis of many thousands of expressed genes while simultaneously controlling for hidden factors. *Bioinformatics*. 2014;30:369-376
161. Hao K, Bosse Y, Nickle DC, et al. Lung eqtls to help reveal the molecular underpinnings of asthma. *PLoS Genet*. 2012;8:e1003029
162. Ding J, Gudjonsson JE, Liang L, Stuart PE, Li Y, Chen W, Weichenthal M, Ellinghaus E, Franke A, Cookson W, Nair RP, Elder JT, Abecasis GR. Gene expression in skin and lymphoblastoid cells: Refined statistical method reveals extensive overlap in cis-eqtl signals. *Am J Hum Genet*. 2010;87:779-789
163. Wagner JR, Busche S, Ge B, Kwan T, Pastinen T, Blanchette M. The relationship between DNA methylation, genetic and expression inter-individual variation in untransformed human fibroblasts. *Genome Biol*. 2014;15:R37
164. Qiu W, Cho MH, Riley JH, et al. Genetics of sputum gene expression in chronic obstructive pulmonary disease. *PLoS One*. 2011;6:e24395
165. Fadista J, Vikman P, Laakso EO, et al. Global genomic and transcriptomic analysis of human pancreatic islets reveals novel genes influencing glucose metabolism. *Proc Natl Acad Sci U S A*. 2014;111:13924-13929
166. Koopmann TT, Adriaens ME, Moerland PD, et al. Genome-wide identification of expression quantitative trait loci (eqtls) in human heart. *PLoS One*. 2014;9:e97380
167. Lin H, Dolmatova EV, Morley MP, Lunetta KL, McManus DD, Magnani JW, Margulies KB, Hakonarson H, del Monte F, Benjamin EJ, Cappola TP, Ellinor PT. Gene expression and genetic variation in human atria. *Heart Rhythm*. 2014;11:266-271
168. Rantalainen M, Herrera BM, Nicholson G, et al. Microrna expression in abdominal and gluteal adipose tissue is associated with mrna expression levels and partly genetically driven. *PLoS One*. 2011;6:e27338
169. Gamazon ER, Innocenti F, Wei R, Wang L, Zhang M, Mirkov S, Ramirez J, Huang RS, Cox NJ, Ratain MJ, Liu W. A genome-wide integrative study of micrornas in human liver. *BMC Genomics*. 2013;14:395
170. Li Q, Stram A, Chen C, Kar S, Gayther S, Pharoah P, Haiman C, Stranger B, Kraft P, Freedman ML. Expression qtl-based analyses reveal candidate causal genes and loci across five tumor types. *Hum Mol Genet*. 2014;23:5294-5302
171. Webster JA, Gibbs JR, Clarke J, et al. Genetic control of human brain transcript expression in alzheimer disease. *Am J Hum Genet*. 2009;84:445-458
172. Zou F, Chai HS, Younkin CS, et al. Brain expression genome-wide association study (egwas) identifies human disease-associated variants. *PLoS Genet*. 2012;8:e1002707
173. Ramasamy A, Trabzuni D, Guelfi S, Varghese V, Smith C, Walker R, De T, Coin L, de Silva R, Cookson MR, Singleton AB, Hardy J, Ryten M, Weale ME. Genetic variability in the regulation of gene expression in ten regions of the human brain. *Nat Neurosci*. 2014;17:1418-1428

174. Gamazon ER, Badner JA, Cheng L, et al. Enrichment of cis-regulatory gene expression snps and methylation quantitative trait loci among bipolar disorder susceptibility variants. *Mol Psychiatry*. 2013;18:340-346
175. Gibbs JR, van der Brug MP, Hernandez DG, et al. Abundant quantitative trait loci exist for DNA methylation and gene expression in human brain. *PLoS Genet*. 2010;6:e1000952
176. Kim S, Cho H, Lee D, Webster MJ. Association between snps and gene expression in multiple regions of the human brain. *Transl Psychiatry*. 2012;2:e113
177. Zhang B, Gaiteri C, Bodea LG, et al. Integrated systems approach identifies genetic nodes and networks in late-onset alzheimer's disease. *Cell*. 2013;153:707-720
178. Shpak M, Hall AW, Goldberg MM, Derryberry DZ, Ni Y, Iyer VR, Cowperthwaite MC. An eqtl analysis of the human glioblastoma multiforme genome. *Genomics*. 2014;103:252-263
179. Colantuoni C, Lipska BK, Ye T, Hyde TM, Tao R, Leek JT, Colantuoni EA, Elkahloun AG, Herman MM, Weinberger DR, Kleinman JE. Temporal dynamics and genetic control of transcription in the human prefrontal cortex. *Nature*. 2011;478:519-523
180. Liu C, Cheng L, Badner JA, Zhang D, Craig DW, Redman M, Gershon ES. Whole-genome association mapping of gene expression in the human prefrontal cortex. *Mol Psychiatry*. 2010;15:779-784
181. Folkersen L, van't Hooft F, Chernogubova E, Agardh HE, Hansson GK, Hedin U, Liska J, Syvanen AC, Paulsson-Berne G, Franco-Cereceda A, Hamsten A, Gabrielsen A, Eriksson P. Association of genetic risk variants with expression of proximal genes identifies novel susceptibility genes for cardiovascular disease. *Circ Cardiovasc Genet*. 2010;3:365-373
182. Björkegren JLM, Kovacic JC, Dudley JT, Schadt EE. Genome-wide significant loci: How important are they?: Systems genetics to understand heritability of coronary artery disease and other common complex disorders. *J Am Coll Cardiol*. 2015;65:830-845
183. Garnier S, Truong V, Brocheton J, et al. Genome-wide haplotype analysis of cis expression quantitative trait loci in monocytes. *PLoS Genet*. 2013;9:e1003240
184. Heinig M, Petretto E, Wallace C, et al. A trans-acting locus regulates an anti-viral expression network and type 1 diabetes risk. *Nature*. 2010;467:460-464
185. Pahl MC, Erdman R, Kuivaniemi H, Lillvis JH, Elmore JR, Tromp G. Transcriptional (chip-chip) analysis of elf1, ets2, runx1 and stat5 in human abdominal aortic aneurysm. *Int J Mol Sci*. 2015;16:11229-11258
186. Lenk GM, Tromp G, Weinsheimer S, Gatalica Z, Berguer R, Kuivaniemi H. Whole genome expression profiling reveals a significant role for immune function in human abdominal aortic aneurysms. *BMC Genomics*. 2007;8:237
187. Hinterseher I, Erdman R, Donoso LA, et al. Role of complement cascade in abdominal aortic aneurysms. *ATVB*. 2011;31:1653-1660
188. Kamburov A, Pentchev K, Galicka H, Wierling C, Lehrach H, Herwig R. Consensuspathdb: Toward a more complete picture of cell biology. *Nucleic Acids Res*. 2011;39:D712-717
189. Kamburov A, Stelzl U, Lehrach H, Herwig R. The consensuspathdb interaction database: 2013 update. *Nucleic Acids Res*. 2013;41:D793-800

190. Kamburov A, Wierling C, Lehrach H, Herwig R. Consensuspathdb--a database for integrating human functional interaction networks. *Nucleic Acids Res*. 2009;37:D623-628
191. Pentchev K, Ono K, Herwig R, Ideker T, Kamburov A. Evidence mining and novelty assessment of protein-protein interactions with the consensuspathdb plugin for cytoscape. *Bioinformatics*. 2010;26:2796-2797
192. Nguyen H, Allali-Hassani A, Antonysamy S, et al. Lly-507, a cell-active, potent, and selective inhibitor of protein-lysine methyltransferase smyd2. *J Biol Chem*. 2015;290:13641-13653
193. Xu G, Liu G, Xiong S, Liu H, Chen X, Zheng B. The histone methyltransferase smyd2 is a negative regulator of macrophage activation by suppressing interleukin 6 (il-6) and tumor necrosis factor alpha (tnf-alpha) production. *J Biol Chem*. 2015;290:5414-5423
194. Calvano SE, Xiao W, Richards DR, et al. A network-based analysis of systemic inflammation in humans. *Nature*. 2005;437:1032-1037
195. Hannou SA, Wouters K, Paumelle R, Staels B. Functional genomics of the cdkn2a/b locus in cardiovascular and metabolic disease: What have we learned from gwass? *Trends Endocrinol Metab*. 2015;26:176-184
196. Nakashima H, Aoki M, Miyake T, Kawasaki T, Iwai M, Jo N, Oishi M, Kataoka K, Ohgi S, Ogihara T, Kaneda Y, Morishita R. Inhibition of experimental abdominal aortic aneurysm in the rat by use of decoy oligodeoxynucleotides suppressing activity of nuclear factor kappab and ets transcription factors. *Circulation*. 2004;109:132-138
197. Nischan J, Gatalica Z, Curtis M, Lenk GM, Tromp G, Kuivaniemi H. Binding sites for ets family of transcription factors dominate the promoter regions of differentially expressed genes in abdominal aortic aneurysms. *Circ Cardiovasc Genet*. 2009;2:565-572
198. Genetic variants in novel pathways influence blood pressure and cardiovascular disease risk. *Nature*. 2011;478:103-109
